# Moderators of peer influence effects for adolescents’ smoking and vaping norms and outcomes in high and middle-income settings

**DOI:** 10.3389/fpsyg.2025.1655761

**Published:** 2025-11-03

**Authors:** Jennifer M. Murray, Sharon C. Sánchez-Franco, Olga L. Sarmiento, Erik O. Kimbrough, Christopher Tate, Shannon C. Montgomery, Rajnish Kumar, Laura Dunne, Abhijit Ramalingam, Erin L. Krupka, Felipe Montes, Huiyu Zhou, Laurence Moore, Linda Bauld, Blanca Llorente, Frank Kee, Ruth F. Hunter

**Affiliations:** ^1^Centre for Public Health, School of Medicine, Dentistry and Biomedical Sciences, Queen's University Belfast, Belfast, Antrim, United Kingdom; ^2^School of Medicine, Universidad de los Andes, Bogotá, Colombia; ^3^Smith Institute for Political Economy and Philosophy, Chapman University, Orange, CA, United States; ^4^College of Education, Health and Human Sciences, Florida State University, Tallahassee, FL, United States; ^5^Queen's Business School, Queen's University Belfast, Belfast, Antrim, United Kingdom; ^6^Centre for Evidence and Social Innovation, School of Social Sciences, Education and Social Work, Queen's University Belfast, Belfast, Antrim, United Kingdom; ^7^Department of Economics, Appalachian State University, Boone, NC, United States; ^8^School of Information, University of Michigan, Ann Arbor, MI, United States; ^9^Department of Industrial Engineering, Universidad de los Andes, Bogotá, Colombia; ^10^School of Informatics, University of Leicester, Leicester, Leicestershire, United Kingdom; ^11^MRC/CSO Social and Public Health Sciences Unit, University of Glasgow, Glasgow, United Kingdom; ^12^Usher Institute, College of Medicine and Veterinary Medicine, University of Edinburgh, Edinburgh, United Kingdom; ^13^Fundación Anáas, Bogotá, Colombia

**Keywords:** smoking, prevention, adolescents, norms, social influence, social networks, moderation analysis, low and middle-income countries

## Abstract

**Background:**

Peer influence is central to adolescent smoking initiation, yet its impact varies depending on individual and contextual factors. Understanding which moderators (personality, contextual, cultural, and environmental traits) shape these processes can inform more effective prevention strategies. We investigated hypothesized moderators of peer influence for adolescent smoking/vaping norms and other smoking-related outcomes in high and low-middle-income countries (LMICs): Northern Ireland and Bogotá.

**Methods:**

Across 12 schools (*n* = 1,344, age 12–13 years), participants completed novel behavioral economics experiments measuring social norms, and self-report surveys, before and after school-based prevention interventions (ASSIST and Dead Cool). We examined how peer influence effects were moderated by setting, intervention type, gender, school socio-economic status (SES), personality traits, social network positions, and self-efficacy. Moderation was examined using regressions with interactions between peer-group means (friends, school classes, school year groups) of the outcome variables and moderators (*p* ≤ 0.01).

**Results:**

Peer influence was moderated by study setting, intervention, gender, school SES, personality characteristics (pro-sociality, fear of negative evaluation, extraversion), and social network structure. Effects were stronger among girls and in schools with lower SES. ASSIST schools showed greater peer influence effects than Dead Cool, reflecting the programs’ distinct mechanisms, as ASSIST operates primarily through network diffusion and Dead Cool through teacher-led instruction and skills-building. Network measures highlighted that peer influence was stronger amongst more central individuals and more homogenous networks.

**Conclusion:**

Susceptibility to peer influence depends on contextual, individual, and network factors. Future social norms interventions should provide information on both injunctive and descriptive norms and highlight the social consequences of smoking, particularly in LMICs. Gender-tailored approaches are needed to address heightened susceptibility among girls. Future intervention research should combine peer-led diffusion approaches with teacher-led instruction to maximize reach and sustainability in different contexts. Social influence-based interventions may be particularly beneficial for schools with lower SES or in LMICs without tobacco control legislation, where smoking remains largely normalized. Network-based interventions like ASSIST could benefit from careful consideration of which network metrics are used to select peer leaders (e.g., eigenvector or closeness centralities) and exploring alternative approaches for more heterogeneous networks (e.g., ‘segmentation’, which targets clusters of individuals within social networks).

## 1 Introduction

The Mechanisms of Networks and Norms Influence on Smoking in Schools (MECHANISMS) study aimed to investigate how social norms about adolescent smoking and vaping spread through school social networks, comparing the results between two research settings with different norms, culture, smoking and vaping rates: Northern Ireland (NI), and Bogotá (Hunter et al., 2020). NI is a high-income country in the United Kingdom (UK) (The World Bank, 2020a), with current smoking rates of 2.2% and electronic cigarette (e-cigarette) consumption rates of 9.2% for adolescents aged 11–16 years (Northern Ireland Statistics and Research Agency, 2023). Bogotá is the capital city of Colombia, an upper-middle-income country (The World Bank, 2020b), with current smoking rates of 6.2% and e-cigarette consumption rates of 12.1% for adolescents aged 12–18 years (Ministry of Justice and Law – Colombian Drug Observatory, Ministry of National Education, 2022). Considering the growing popularity of e-cigarettes among adolescents, norms for smoking and vaping were both considered in the MECHANISMS study (Perikleous et al., 2018; Schneider and Diehl, 2016; Hartmann-Boyce et al., 2022). Adolescents who vape are also more likely to use e-cigarettes for experimentation, similar to how adolescents typically use conventional cigarettes, and are more likely to start smoking (Perikleous et al., 2018; Soneji et al., 2017). The study used transdisciplinary insights to compare the mechanisms of two school-based smoking prevention programs with proven effectiveness in previous cluster randomized trials in the UK: The A Stop Smoking in Schools Trial (ASSIST) and Dead Cool (Hunter et al., 2020; Campbell et al., 2008; Thurston et al., 2019). It is also the first study to apply experimental methods from behavioral economics and game theory to elicit social norms for adolescent smoking and vaping behaviors (Kimbrough and Vostroknutov, 2016; Kimbrough and Vostroknutov, 2018; Krupka and Weber, 2013).

### 1.1 Measuring social norms using behavioral economics experiments in the MECHANISMS study

The MECHANISMS study experimental protocol used financially incentivized ‘co-ordination’ games to elicit injunctive social norms – what people *ought* to do – as shared perceptions within whole school year groups on the social appropriateness of various smoking and vaping-related scenarios (Krupka and Weber, 2013). Descriptive norms – what people *actually* do – were elicited as shared perceptions of the rate of acceptance of smoking or vaping behaviors within the school year group (Krupka and Weber, 2013). To encourage participants to consider how most others in their year group would respond (i.e., the norm) instead of providing personal opinions, they were informed that they would receive a payment if their answer matched the most common answer provided in their school year group (Krupka and Weber, 2013; Murray et al., 2020). That is, we asked the pupils to guess how most of their classmates would respond to the smoking-related scenarios, rewarding them if they matched the majority answer. This way, we measured group perceptions rather than just individual opinions. One of the advantages of these experimental methods compared to the traditional self-reports used in public health research is that they mitigate social desirability biases since respondents must report their beliefs about others’ beliefs rather than answering personally (Murray et al., 2020; Mackie et al., 2015). Another advantage is that the method is theoretically intuitive since the existence of such shared “second-order” beliefs is a necessary pre-condition for the existence of a social norm (Bicchieri et al., 2018). On the other hand, self-report methods of measuring social norms have the advantages of simplicity, low cost, and ease of distribution (Murray et al., 2020). Furthermore, the experimental and self-report measures used in our study focus on different aspects of norms. The experiments inquire about the beliefs of the whole school year group whilst the self-report methods ask about influences amongst the respondent’s own family, friends and peers (Murray et al., 2020).

The experimental norms measures can also potentially provide richer insights into intervention mechanisms to better explain variation in individuals’ behaviors within and between different contexts (Kimbrough and Vostroknutov, 2016; Kimbrough and Vostroknutov, 2018; Krupka and Weber, 2013; Murray et al., 2020). For example, the experiments included a measure of individuals’ sensitivities to social norms, or rule-following propensity (Kimbrough and Vostroknutov, 2016; Kimbrough and Vostroknutov, 2018). The task instructed participants to follow an arbitrary rule when doing so imposed explicit monetary penalties directly proportional to the degree of rule-following. In principle, the more a participant cares intrinsically about rule-following the more willing he/she should be to incur the costs of doing so (Kimbrough and Vostroknutov, 2016). Put simply, the task measured how much individual pupils valued following rules and norms in terms of how willing they were to forgo a payment to obey the rule. This norm sensitivity measure has been shown to correlate with willingness to follow norms of cooperation, reciprocity, and pro-social behavior in different decision contexts (Kimbrough and Vostroknutov, 2016). According to the behavioral economics theory, individuals with higher norms sensitivities are more likely to conform to the norms within their social context (Kimbrough and Vostroknutov, 2016; Kimbrough and Vostroknutov, 2018; Krupka and Weber, 2013; Murray et al., 2025a).

The MECHANISMS study is the first to use coordination games to measure norms around adolescent smoking and vaping, and to investigate how individuals’ conformity to social norms varies with this norm-sensitivity measure (Hunter et al., 2020).

### 1.2 Tobacco consumption and social influence

Tobacco consumption is the leading preventable risk factor for chronic disease and mortality worldwide, responsible for over seven million annual deaths from direct consumption and 1.2 million from second-hand smoke (World Health Organization, 2025). Most adult smokers start smoking during adolescence (Institute of Medicine, 2015), a developmental stage when susceptibility to social influences is heightened (Foulkes and Blakemore, 2016). During adolescence, many young people take their cues consciously or subconsciously from observing the beliefs, actions, and attitudes of friends, peers and family (Littlecott et al., 2019; Allen and Feigl, 2017; Vitória et al., 2011). Whether it is due to peer influence – a social process where an individual’s behavior or attitudes are affected by observing peers within social networks – or through selection homophily processes – the tendency for individuals to form friendships with others who share similar characteristics and behaviors – research consistently shows that adolescent smokers usually have more smoking friends, whilst non-smokers have more non-smoking friends (Liu et al., 2017; Steglich et al., 2012; Krupka et al., 2016; Montgomery et al., 2020). In other words, smokers often end up surrounded by other smokers, either because they are drawn to friends who already smoke, or because they are influenced by smoker friends to start smoking.

Numerous studies have highlighted the importance of peer influence and peer selection homophily in shaping adolescent smoking outcomes, mostly focusing on smoking behavior, intentions, and susceptibility (Montgomery et al., 2020; Watts et al., 2024; Hoffman et al., 2007; Mercken et al., 2012; Lahiri et al., 2024; Trucco, 2020). Within the MECHANISMS study, we have contributed to this literature by investigating these social network processes with respect to our study’s novel experimental measures of adolescent smoking norms and a range of psychosocial antecedents of smoking (e.g., attitudes, self-efficacy, and perceived risks and benefits) (Montes et al., 2023; Murray et al., 2023). We also compared peer influence estimates derived from Simulation Investigation of Empirical Network Analysis (SIENA) models with conventional regression-based methods that are more common in public health and behavioral economics research (Hoffman et al., 2007; Mercken et al., 2012; Flashman and Gambetta, 2014; Fowler and Christakis, 2008; Go et al., 2012; Miething et al., 2016; Parkinson et al., 2018; Rohrer et al., 2021; Mercken et al., 2009; Ripley et al., 2025; Steglich et al., 2010; Snijders et al., 2010; Mercken et al., 2010; Ragan et al., 2019). One advantage of the regression-based approach was that it allowed us to distinguish the influence of proximal peers (e.g., close friends) from more distal peers (e.g., pupils in the same school class or year group), and to assess both lagged and contemporaneous peer influence effects (Murray et al., 2023). Specifically, we tested whether participants’ outcomes at follow-up were predicted by the average responses of their nominated friends, school classes, or year groups, finding positive peer influence effects across most study outcomes (Murray et al., 2023).

Given the overwhelming evidence that social influences are central in determining adolescent smoking uptake, many smoking prevention programs target young adolescents (typically aged 12–13 years) and use social norms or peer influence approaches (Campbell et al., 2008; Thomas et al., 2015; Ahmed et al., 2018). Social influence-based interventions have been effective for preventing adolescent smoking uptake in high-income settings, but reviews have highlighted a lack of evidence from low and middle-income countries (LMICs) (Thomas et al., 2015; Munabi-Babigumira et al., 2012; Huriah and Dwi, 2020; Ba-Break et al., 2023; Nishio et al., 2018; Macarthur et al., 2016; Brown et al., 2014). They recommend that high-quality studies should be conducted in LMICs, incorporating successful strategies from high income settings, appropriately adapted for local culture and conditions (Munabi-Babigumira et al., 2012). This is particularly important given that smoking rates are declining in high-income countries but remain high in LMICs, which now account for over 80% of the world’s 1.3 billion tobacco users (World Health Organization, 2025).

### 1.3 Investigating moderators of peer influences for adolescent smoking

Measuring individuals’ norm sensitivities as part of our experimental protocol reflects an important component of social influence theory. Namely, the extent of attitude or behavior change that occurs through social influence is largely due to variation in individuals’ susceptibilities to social influences (Stacy et al., 1992; McGuire, 1968). Social influences may only have a strong impact on the behavior of individuals and groups with characteristics (e.g., personality, contextual, cultural, and environmental traits) that make them susceptible to social influences (Stacy et al., 1992).

For ‘socially contagious’ behaviors like smoking (Littlecott et al., 2019; Allen and Feigl, 2017), it is important to establish how certain moderating variables may interact to vary the impact of social influence on behavior. Moderators are defined as qualitative (e.g., setting) or quantitative (e.g., rule-following) variables that affect the direction and/or strength of the relationship between a predictor variable and an outcome (Baron and Kenny, 1986). In the current study, a moderating effect implies that the effect of peer influence on smoking norms or outcomes varies at different values of the moderator and is indicated by a significant interaction effect between the moderator and predictor over and above the additive effects of the two variables (Cohen and Cohen, 1983). In simple terms, if individuals’ norm sensitivities act as a moderator, this means individuals who are more sensitive to social norms are more likely to be influenced by their peers’ smoking behaviors.

Previous studies have investigated factors like gender (McCoy et al., 2017; Lansford et al., 2009), social network positions and structure (Lansford et al., 2009; Haynie, 2015), personality traits (Slagt et al., 2015; Marschall-Lévesque et al., 2014), and context (Marschall-Lévesque et al., 2014), as potential moderators of peer influence in adolescent risk behaviors. One study conducted in the United States in 1992 investigated personal characteristics such as gender, self-efficacy, self-esteem, parental supervision, and perceived stress, as potential moderators of peer influence in adolescent smoking and found significant moderating effects of self-efficacy (Stacy et al., 1992). Having higher levels of self-efficacy (i.e., greater belief in your own ability to resist social influence for smoking) reduced the strength of social influence from friends on individuals’ smoking behavior (Stacy et al., 1992). A recent review of moderators of peer influence for adolescent substance use identified that ten of the 43 included studies investigated tobacco use (Rodríguez-Ruiz and Espejo-Siles, 2025). The authors found evidence that peer influences for adolescent substance use are moderated by a range of individual, family, school, peer, and community factors including emotional control and anxiety, peer proximity and reciprocity, closeness to parents, siblings’ willingness to use substances, school disapproval and school troubles, peer support, and neighborhood characteristics.

Moderation analyses can provide answers for important public health questions, including which participants benefit most from intervention strategies, and in what contexts (Sheeran et al., 2017). Investigating potential moderators of social influences for adolescent smoking could have important implications in terms of how smoking prevention programs are designed and implemented. For example, knowledge of effective moderating variables can help with identifying individuals and groups who are most susceptible to social influences for smoking or identifying the most efficient individuals within social networks to recruit as peer leaders to spread anti-smoking messages. Medical Research Council guidance on developing complex interventions highlights that the context within which an intervention operates is a critical factor that can act as a barrier or facilitator to its implementation or effectiveness (O’Cathain et al., 2019; Moore et al., 2015). Recent research also suggests that peer processes in adolescent smoking may vary as the tobacco control context and societal norms change when countries introduce tobacco control legislation (Littlecott et al., 2023; Littlecott et al., 2022). Having been designed to compare norms and social network-based intervention mechanisms between schools in a high-income setting (NI) with schools in a middle-income setting (Bogotá), the MECHANISMS study provides a unique opportunity to investigate the moderating effects of context on peer influences for adolescent smoking. The experimental protocol also proposes that peer influences should be moderated by individuals’ norms sensitivities (Hunter et al., 2020).

In the current paper, we investigated hypothesized moderators of peer influence effects for our adolescent smoking and vaping outcomes, by including interactions between peer-group means (nominated friends, school classes, and school year groups) of the outcome variables and the moderators in regression models (Hayes, 2013). Our outcomes included experimental smoking and vaping norms, self-report smoking norms, self-report and objectively measured smoking behavior, intentions, susceptibility, knowledge, attitudes, self-efficacy, perceived risks and benefits, and perceived behavioral control (PBC). Our hypothesized moderators included setting (NI versus Bogotá), intervention (ASSIST versus Dead Cool), gender, school socio-economic status (SES), individuals’ norm sensitivities and other socially oriented personality characteristics, the ‘Big Five’ personality traits (Morizot, 2014; Ortet et al., 2017), social network parameters, and self-efficacy to resist smoking.

### 1.4 Theoretical framework for the current study

#### 1.4.1 Theories of social influence

Several behavior change theories can help explain how peers shape adolescent smoking behaviors. As previously discussed, two competing theories describing how health-related attitudes and behaviors evolve and are transmitted in social networks include the peer socializing (influence) theory and the peer selection theory. The peer socializing theory states that peers’ smoking behaviors are important in explaining an individual’s future behavior, whilst the peer selection theory states that an individual’s own smoking behavior will determine which friends they choose. Empirical studies suggest that both theories are important for explaining adolescent smoking and other substance use (Hoffman et al., 2007; Mercken et al., 2012; Go et al., 2012; Marschall-Lévesque et al., 2014; Simons-Morton and Farhat, 2010; Kiuru et al., 2010; Fergusson et al., 2002; Duarte et al., 2011; Dishion and Owen, 2002; Mercken et al., 2012; Wills and Cleary, 1999).

Bronfenbrenner’s 1977 ecological and 1979 bioecological models have been central in organizing the socialization factors that contribute to adolescent smoking and other substance use into a coherent framework (Trucco, 2020; Marschall-Lévesque et al., 2014; Rodríguez-Ruiz and Espejo-Siles, 2025; Bronfenbrenner, 1974; Bronfenbrenner, 1979). The ecological model conceptualizes development and health behavior as being shaped by nested environmental systems. The microsystem includes immediate socialization environments that affect the child directly (e.g., peers, parents, siblings, school). The mesosystem includes connections and interactions between the microsystems (e.g., teacher-parent interactions). The exosystem includes larger social environments that influence the child through indirect effects on the microsystem (e.g., neighborhoods). The macrosystem represents the outermost layer of socialization, encompassing cultural values, politics, religion, and laws, which shape individual development through a cascading influence on all other levels. Whilst the ecological model viewed the child largely as a passive recipient of these environmental influences, the bioecological model introduced the role of individual factors and active human agency. In the bioecological model, development and health behaviors are understood as the product of ongoing, reciprocal interactions between the individual and the environment, with attributes such as temperament, health, and genetic predispositions shaping how these interactions unfold (Trucco, 2020; Marschall-Lévesque et al., 2014; Rodríguez-Ruiz and Espejo-Siles, 2025; Bronfenbrenner, 1974; Bronfenbrenner, 1979; Bronfenbrenner and Morris, 1998). Thus, the bioecological model recognizes that individuals differ in their susceptibility to peer influence. Similarly, the theory of triadic influence and other integrative models emphasize how individual, social, and cultural-environmental factors interact across multiple levels of influence to shape behavior (Marschall-Lévesque et al., 2014; Flay et al., 1995; Flay and Petraitis, 1994; Fishbein, 2000; Fishbein, 2009; Michie et al., 2014; Flay et al., 1983).

In social learning theory, modelling is the primary mechanism through which social environments shape behavior (Bandura and McClelland, 1977). Individuals observe and imitate the behaviors of others, as they anticipate social rewards like increased status or affection. Bandura (1977) proposed that favorable attitudes toward smoking and substance use are reinforced when a role model is perceived as rewarded for those behaviors, similar to the observer, and possessing higher social status (Watts et al., 2024; Trucco, 2020; Michie et al., 2014; Bandura and McClelland, 1977; Laninga-Wijnen and Veenstra, 2023). Complementing this, the perception-behavior link paradigm highlights that individuals often mimic others’ behaviors spontaneously, even without conscious intent (Laninga-Wijnen and Veenstra, 2023; Chartrand and Bargh, 1999). A common assumption is that young people engage in behaviors like smoking and substance use because their peers pressure them. However, these frameworks can help explain why adolescents are often influenced passively rather than actively. That is, they often adopt behaviors through observation, imitation, and conformity to perceived social norms. Indeed, research suggests that direct peer pressure is relatively rare, whereas perceived peer approval and exposure to peers’ substance use during early adolescence are particularly influential in shaping behavior (Trucco, 2020; Laninga-Wijnen and Veenstra, 2023; Bauman and Ennett, 1996). Furthermore, social norms theories propose that behaviors are influenced by inaccurate perceptions of the attitudes and behaviors of others within social groups (Michie et al., 2014; Perkins and Berkowitz, 1986; Berkowitz, 2004). Adolescents are particularly prone to overestimating peers’ engagement in health risk behaviors such that many adolescent smoking prevention programs invoke social norms approaches attempting to align perceptions of prevalence rates with actual prevalence (Trucco, 2020; Ahmed et al., 2018; Borsari and Carey, 2003; Helms et al., 2014; Chung and Rimal, 2016).

Social control theory posits that strong social bonds – characterized by attachment, commitment to norms, involvement in valued activities, and belief in societal rules – protect adolescents from engaging in antisocial behaviors like smoking (Trucco, 2020; Rodríguez-Ruiz and Espejo-Siles, 2025; Hirschi, 1969). Consistent with this view, research shows that adolescents’ openness to peer influence is shaped by the quality of their relationships, with higher-quality bonds predicting greater susceptibility to peers’ substance use behaviors (Allen et al., 2022). The social development model integrates social control and social learning theories, proposing that adolescents form bonds across family, school, community, and peer contexts based on anticipated rewards for prosocial or antisocial behaviors. Adolescents who anticipate rewards for prosocial actions are more likely to engage in prosocial activities, whereas those who anticipate rewards for antisocial actions are more likely to engage in behaviors like substance use. The model also adopts a developmental perspective, recognizing that the influence of socializing agents shifts with age, for example moving from parents to peers as adolescence progresses. Social development theory acknowledges that individual factors, such as temperament or self-regulation, can moderate the effects of social bonds and perceived rewards, buffering adolescents from negative peer influences and reducing engagement in risky behaviors (Watts et al., 2024; Trucco, 2020; Marschall-Lévesque et al., 2014; Michie et al., 2014; Hawkins and Weis, 1985; Catalano and Hawkins, 1996; Catalano et al., 1996).

The diathesis–stress and differential susceptibility models complement social development theory by offering a framework explicitly addressing why adolescents differ in their responsiveness to the same socializing influences. The diathesis-stress model proposes that individual vulnerabilities – such as low self-regulation, heightened impulsivity, or genetic risk factors – interact with adverse peer environments to increase the likelihood of smoking initiation. Individuals without such vulnerabilities are less affected by the same peer pressures (Zuckerman, 1999; Monroe and Simons, 1991; Wills and Dishion, 2004). The differential susceptibility model extends this vulnerability-based view to suggest that some adolescents are more broadly sensitive to environmental influences. The same individual characteristics may also amplify sensitivity to positive peer influences, such that highly susceptible adolescents are not only more vulnerable in risky peer contexts but also more likely to benefit from prosocial peer environments (Belsky, 1997; Belsky, 2005; Belsky et al., 2007; Belsky and Pluess, 2009b; Ellis et al., 2011; Belsky and Pluess, 2009a; Belsky, 2013) These models account for heterogeneity in adolescent responses to peer influence, supporting the idea that adolescent smoking behaviors may be determined by the interaction between individual predispositions and social environments.

#### 1.4.2 Hypothesized moderators in the MECHANISMS study

The MECHANISMS study was conducted in two diverse research settings with different smoking rates, norms, cultures, and tobacco control contexts. Adolescent smoking and vaping rates are 4 and 3% higher in Bogotá compared to NI, respectively (Northern Ireland Statistics and Research Agency, 2023; Ministry of Justice and Law – Colombian Drug Observatory, Ministry of National Education, 2022). The UK has comprehensive tobacco control legislation, and tobacco education is embedded in the school curriculum (UK Government Office for Health Improvement and Disparities, 2021; UK Government Department of Health, 2017; Action on Smoking and Health (ASH), 2017; National Institute for Health and Clinical Excellence (NICE), 2023). Colombia ratified the World Health Organization Framework Convention on Tobacco Control (WHO-FCTC) in 2009, and follow-up reports indicate high compliance with regulations banning sales to minors and tobacco advertising (Ministerio de Salud y Protección Social, 2009; Colombia Ombudsman Office, 2017). Nonetheless, adolescents in Colombia may still access tobacco products through informal means, such as contraband cigarettes or street vendors (Colombia Ombudsman Office, 2017). Like many Latin American countries, Colombia has historically been vulnerable to the tobacco epidemic, with smoking embedded in cultural practices (Müller and Wehbe, 2008). Implementing the WHO-FCTC was challenging due to opposition from tobacco companies, limited state capacity, and attempts to position tobacco as a post-conflict development strategy (Müller and Wehbe, 2008; Uang et al., 2018). Broader cultural characteristics can also help explain differences in smoking patterns across settings. For example, the degree of individualism–collectivism in a society can affect individuals’ sensitivity to peer influences and the value placed on norm conformity (Liu et al., 2017). Research shows that correlations between adolescents’ smoking and their peers’ smoking behaviors are stronger in collectivistic than in individualistic cultures (Liu et al., 2017). Collectivistic cultures also place greater emphasis on conformity to norms and social acceptance (Liu et al., 2017). Generally, high-income countries tend to be more individualistic, whereas LMICs including those in Latin America, tend to be more collectivistic (Weiss et al., 2019; Peng and Paletz, 2011; Hofstede Insights, 2022). Recent research further indicates that the UK is among the most individualistic cultures globally, while Colombia is among the most collectivistic (Hofstede Insights, 2022; Minkov and Kaasa, 2022). As a collectivistic culture that has historically been vulnerable to the tobacco epidemic, we hypothesized that peer influence effects for adolescent smoking would be stronger in Bogotá compared to NI.

One of the main objectives of the MECHANISMS study was to compare intervention mechanisms in two different school-based smoking prevention programs (Hunter et al., 2020). ASSIST is grounded in the diffusion of innovations theory and is designed to harness peer influence. It recruits the most influential pupils within each school year group to act as peer supporters, spreading prevention messages through informal conversations with their friends (i.e., peer education and diffusion) (Campbell et al., 2008; Rogers, 2003). According to diffusion theory, four components drive this process: the innovation (e.g., smoking prevention messages), communication channels (e.g., peer-to-peer conversations), time (e.g., the intervention period), and the social system (e.g., the school year group) (Rogers, 2003). Thus, peer influence is embedded in the intervention’s design. By contrast, Dead Cool is a classroom-based program, rooted in more conventional pedagogy and the theory of planned behavior. It is delivered to all pupils and provides accurate information on smoking, addresses influences from family, friends, and the media, and develops skills to resist smoking (Thurston et al., 2019; Ajzen, 1991). Previous MECHANISIMS research found that selection homophily and/or peer influence accounted for a greater proportion of smoking- and vaping-related similarity between friends in ASSIST schools compared to Dead Cool (Montes et al., 2023; Murray et al., 2023). Given its explicit focus on leveraging peer influence, we hypothesized stronger peer influence effects in ASSIST schools than in Dead Cool.

Research suggests there are gender differences in susceptibility to peer influences for adolescent smoking, with girls typically being more strongly influenced by peer smoking than boys (Mercken et al., 2010; Simons-Morton and Farhat, 2010; Hu et al., 1995; Hoving et al., 2007; McMillan et al., 2018; Barber et al., 1999; Michell and Amos, 1997). Previous MECHANISMS research similarly found that girls were more sensitive to norms in the experimental rule-following task (Tate et al., 2022), and were more likely to have descriptive norms favorable towards smoking (Montes et al., 2023). Adolescents tend to form same-sex friendships and gender differences in adolescent social networks may help explain why girls feel more social pressures to smoke (Mercken et al., 2009; Mercken et al., 2010; McMillan et al., 2018). Females are more likely to form selective and intimate friendships, place greater importance on social relationships, and seek support from peers (Mercken et al., 2010; McMillan et al., 2018; Thomas and Daubman, 2001). They may also be especially sensitive to social-evaluative concerns, relying on close friendships as an important source of self-worth and self-evaluation (McCoy et al., 2017; Thomas and Daubman, 2001; Rudolph and Conley, 2005), which increases opportunities for peer influence (Mercken et al., 2010). Consistent with these dynamics, patterns of adolescent tobacco use show similar gender differences in both NI and Colombia. Current cigarette use remains higher among boys (2.8% versus 1.6% in NI and 4.8% versus 4.3% in Colombia). However, current e-cigarette use is now more prevalent among girls (9.4% versus 8.9% in NI and 11.6% versus 10.8% in Colombia) (Northern Ireland Statistics and Research Agency, 2023; Ministry of Justice and Law – Colombian Drug Observatory, Ministry of National Education, 2022). These findings suggest that peer influence processes may be particularly pronounced for girls, leading us to hypothesize that we would observe stronger peer influence effects among girls compared to boys.

Research consistently shows that smoking prevalence is higher amongst more socially disadvantaged groups (Soteriades and DiFranza, 2011; Green et al., 2016; Hiscock et al., 2012). From a theoretical viewpoint, collective efficacy theory suggests that disadvantaged neighborhoods, which are often characterized by low social cohesion and weak informal social control, are less able to regulate adolescent behavior, contributing to higher rates of substance use (Trucco, 2020; Sampson, 1992; Leventhal and Brooks-Gunn, 2000; Handley et al., 2015; Mayberry et al., 2009). The integrative model of smoking behavior also emphasizes SES as an indirect determinant of adolescent smoking initiation, as it influences parental smoking patterns and peer environments (Michie et al., 2014; Flay et al., 1983). Recent reviews emphasize the importance of examining differences across socio-economic contexts – such as school-level SES – when designing interventions, particularly in societies where tobacco control legislation has advanced the denormalization of smoking, but health inequalities persist (Littlecott et al., 2023; Littlecott et al., 2022; Harper and McKinnon, 2012; Action on Smoking and Health (ASH), 2019; Moore and Littlecott, 2015). For instance, social influence–based interventions may be especially valuable in schools serving more deprived populations or in LMICs without strong tobacco control policies, where smoking remains widely normalized (Littlecott et al., 2023; Littlecott et al., 2022). Supporting this, the original ASSIST study in the UK reported greater intervention effectiveness in lower-SES schools (Campbell et al., 2008; Littlecott et al., 2023; Littlecott et al., 2022). Building on this evidence, we hypothesized that peer influence effects would be stronger in schools with lower SES.

According to behavioral economic theory, individuals’ sensitivity to norms – captured in our experimental rule-following measure – should moderate peer influence effects, as those who are more rule-following and experience greater ‘disutility’ from norm violations are more likely to conform to the prevailing norms in their social context (Kimbrough and Vostroknutov, 2016; Kimbrough and Vostroknutov, 2018). Similarly, the affective quality of peer relations might shape susceptibility to peer influence (Manzoni et al., 2011). That is, individuals who experience stronger negative affect from social exclusion or increased positive affect from social acceptance might be more likely to adjust their behavior in line with peers (Manzoni et al., 2011). To capture these socially oriented dispositions, we additionally measured self-reported pro-sociality (Goodman et al., 2003; Bevelander et al., 2018), fear of negative evaluation (FNE) (Bevelander et al., 2018; Leary, 1983; Collins et al., 2005), and need to belong (NTB) (Bevelander et al., 2018; Leary et al., 2013). Based on this framework, we hypothesized that pupils with higher levels of norm sensitivity, pro-sociality, FNE, and NTB would show stronger peer influence effects.

Research indicates that personality traits are important dispositional factors that can affect how individuals respond to social influences (Denissen and Penke, 2008). Prior studies have examined the ‘Big Five’ personality traits – openness, extraversion, agreeableness, conscientiousness, and emotional stability (Morizot, 2014; Ortet et al., 2017) – as potential moderators of peer influence in adolescent and young adult smoking, alcohol use, and delinquent behaviors, although the findings are varied across behaviors and datasets (Slagt et al., 2015; van Schoor et al., 2008; de Leeuw et al., 2010; Yu et al., 2013; Gallego et al., 2018; Theakston et al., 2004; Pocuca et al., 2018; Poelen et al., 2007). Within the MECHANISMS study, pupils with higher scores on each of the ‘Big Five’ traits reported stronger anti-smoking norms (Murray et al., 2020), and, greater openness and lower extraversion were linked with reduced odds of being susceptible to smoking initiation in NI (Tate et al., 2021). Given these mixed findings, we conducted an exploratory analysis to assess whether the ‘Big Five’ traits moderated the peer influence effects, without making pre-specified hypotheses about the direction of associations.

Social network structures affect how social influence operates and how social norms spread (Hunter et al., 2020; Panter-Brick et al., 2006). Previous research suggests that an adolescent’s ‘popularity’, as measured by their eigenvector centrality within high school social networks, affects the influence they exert on their peers’ smoking behavior (Robalino and Macy, 2018). Studies have also examined how individuals’ positions within networks moderate susceptibility to peer influence (Lansford et al., 2009), and how broader network structures determine adolescents’ vulnerability to peer influences for delinquent behaviors or cannabis use (Haynie, 2015; Torrejón-Guirado et al., 2023). Recent work emphasizes the importance of considering multiple network parameters when investigating diffusion processes (Badham et al., 2018; Badham et al., 2019; Badham et al., 2021). In the current study, we assessed moderation of peer influence effects using individuals’ clustering coefficients, eigenvector centralities, closeness centralities, betweenness centralities, and school-level Gini degree coefficients, which capture heterogeneity in network degree distributions (Badham, 2013). We hypothesized that peer influence would be stronger among pupils with higher clustering coefficients – indicating more tightly interconnected friends – and those more central in their school network according to their eigenvector, closeness, or betweenness measures. Additionally, we expected peer influence effects to be stronger in schools with lower Gini degree coefficients since heterogeneity is predicted to inhibit network diffusion under complex contagion processes (Badham et al., 2021; Pastor-Satorras et al., 2015).

Intervention logic models in the MECHANISMS study protocol identify self-efficacy as a mediator that is expected to be increased by the smoking prevention programs, and to lead on to reduced smoking behavior, susceptibility, and intentions to smoke (Hunter et al., 2020). However, some authors have conceptualized self-efficacy or refusal assertiveness as a more stable behavior-specific personality trait that may act as a moderator (Stacy et al., 1992; Rodríguez-Ruiz and Espejo-Siles, 2025; Schwarzer, 2015), and early research on moderators of peer influences in adolescent smoking found that having higher levels of self-efficacy can act as a buffer that protects adolescents against social influence (Stacy et al., 1992). Accordingly, we examined self-efficacy both as an outcome – subject to change between baseline and follow-up through peer influence mechanisms – and as a moderator, with baseline levels affecting susceptibility to peer influence for smoking and vaping outcomes. Consistent with self-efficacy theory and prior empirical findings, we hypothesized that pupils with lower self-efficacy to resist smoking in various emotional, social and environmental contexts would experience stronger peer influence effects (Stacy et al., 1992; Bandura, 1977; Bandura, 1986).

In summary, our tested moderators and hypotheses were as follows:

- Setting: We hypothesized that we would observe stronger peer influence effects for Bogotá compared to NI.
- Intervention: We hypothesized that we would observe stronger peer influence effects for ASSIST schools compared to Dead Cool schools.
- Gender: We hypothesized that we would observe stronger peer influence effects for girls compared to boys.
- School socio-economic status: We hypothesized that we would observe stronger peer influence effects in schools with lower SES.
- Norm sensitivities and related personality characteristics: We hypothesized that we would observe stronger peer influence effects for pupils with higher experimentally measured norm sensitivities, self-reported pro-sociality, FNE, and NTB.
- ‘Big Five’ personality traits: We conducted an exploratory analysis to investigate moderation of the peer influence effects according to the ‘Big Five’ personality traits of openness, extraversion, agreeableness, conscientiousness, and emotional stability.
- Social network parameters: We hypothesized that we would observe stronger peer influence effects for pupils with higher clustering coefficients, eigenvector centralities, closeness centralities, and betweenness centralities. We also hypothesized that we would observe stronger peer influence effects for school networks with lower Gini degree coefficients (i.e., less heterogeneous school networks).
- Self-efficacy to resist smoking: We hypothesized that we would observe stronger peer influence effects for pupils with lower self-efficacy to resist smoking.

**Figure 1:**
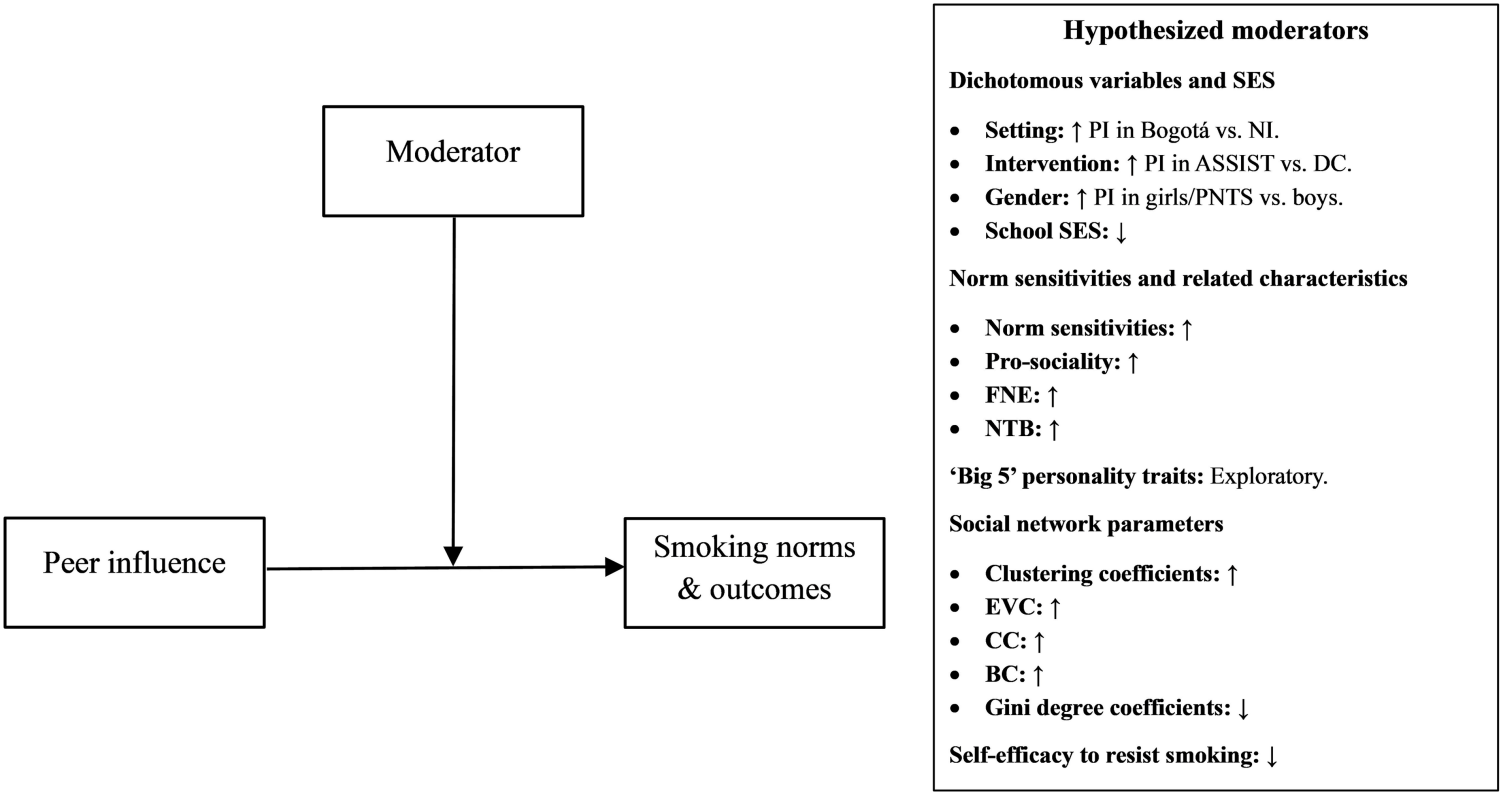
Conceptual diagram showing the hypothesized moderating effects. ASSIST: ‘A Stop Smoking in Schools Trial’, BC: betweenness centrality, CC: closeness centrality, DC: Dead Cool, EVC: eigenvector centrality, FNE: fear of negative evaluation, NI: Northern Ireland, NTB: need to belong, PI: peer influence. “↑ PI in Bogotá vs. NI” indicates we hypothesized that peer influence effects would be stronger in Bogotá compared to NI. “School SES: ↓” indicates we hypothesized that peer influence effects would be stronger in schools with lower SES. “Norm sensitivities: ↑” indicates we hypothesized that peer influence effects would be stronger for pupils with higher norm sensitivities.

Figure 1 shows a conceptual diagram of the hypothesized moderators and directions of the effects.

## 2 Methods

### 2.1 Study design

The MECHANISMS study is a pre-post quasi-experimental study (Hunter et al., 2020). Twelve schools (*N* = 6 NI, *N* = 6 Bogotá; *n* = 1344/1444 pupils, participation = 93.1%) participated in the MECHANISMS study between January and November 2019 (Hunter et al., 2020). Study procedures have previously been described (Hunter et al., 2020; Murray et al., 2020; Murray et al., 2023; Sánchez-Franco et al., 2021). Full school year groups were recruited in each school (NI Year 9, Bogotá Year 7; target age 12–13 years). Schools were assigned to the ASSIST or Dead Cool programs (Campbell et al., 2008; Thurston et al., 2019). During one school semester, pupils received the smoking prevention programs, and completed incentivized (monetary) norms elicitation experiments (Kimbrough and Vostroknutov, 2016; Kimbrough and Vostroknutov, 2018; Krupka and Weber, 2013) and self-report surveys, before and after the programs. All data collection was conducted via Qualtrics (Qualtrics, Provo, Utah, USA) on tablet computers. Prior to implementation in Bogotá, all study materials and intervention programs were thoroughly culturally adapted, including translation into Spanish language (Sánchez-Franco et al., 2021). Ethics approval was granted from Queen’s University Belfast (21 September 2018, reference 18:43) and Universidad de los Andes (30 July 2018, reference 937/2018). Participants and parents provided informed consent, and data collection procedures complied with institutional guidelines. Further details on study procedures are available in Supplementary File 1. The study flow diagram is shown in Supplementary File 1: Figure S1.1, and participants’ baseline characteristics are shown in Supplementary File 1: Table S1.1.

### 2.2 Settings

NI is a high-income country in the UK (The World Bank, 2020b), with around 2 million inhabitants (Northern Ireland Statistics and Research Agency, 2019). In 2022, current cigarette consumption rates were 2.2% for adolescents aged 11–16 years in NI (1.0% reported smoking at least once a week and 7.6% reported having smoked tobacco at least once before). Current e-cigarette consumption rates were 9.2% (6.3% reported vaping at least once a week and 21.3% reported having vaped at least once before) (Northern Ireland Statistics and Research Agency, 2023). In the UK, it is illegal to sell tobacco products to or buy tobacco products for minors under the age of 18 years (UK Government, 2014). Tobacco education is a formal part of the UK school curriculum (National Institute for Health and Clinical Excellence (NICE), 2023). The UK has comprehensive tobacco control legislation regulating tobacco advertising, sale to minors, packaging, smoke-free public places, and in-door smoking (UK Government Office for Health Improvement and Disparities, 2021; UK Government Department of Health, 2017; Action on Smoking and Health (ASH), 2017). It is also illegal to sell e-cigarettes to under 18s. Whilst the UK government has sought to maximize the potential of e-cigarettes as a smoking cessation aid for adults, they are currently introducing stricter legislation like the tobacco control measures, to regulate sale to minors and ban advertising and sponsorship of e-cigarettes and other nicotine products (UK Government Department of Health, 2024; UK Government Department of Health, 2022).

Bogotá is the capital city of Colombia, an upper-middle-income country (The World Bank, 2020b), with over 7 million inhabitants (National Administrative Department of Statistics, 2019). In 2022, current cigarette consumption rates were 4.5% for adolescents aged 12–18 years across Colombia (11.1% reported having used tobacco or cigarettes at least once before). In Bogotá, current cigarette consumption rates were 6.2%. Current e-cigarette consumption rates were 11.2% for adolescents aged 12–18 years across Colombia (22.7% reported having used e-cigarettes at least once before). In Bogotá, current e-cigarette consumption rates were 12.1% (Ministry of Justice and Law – Colombian Drug Observatory, Ministry of National Education, 2022). Colombia adopted the WHO-FCTC in 2009 which includes legislation regulating tobacco advertising, packaging, sale to minors, and smoke-free public places (Ministerio de Salud y Protección Social, 2009). In Colombia, selling tobacco products to minors under the age of 18 years is illegal. This has a high level of compliance within the regulatory sphere. The legislation also includes a complete ban on tobacco advertisements, sponsorships, and promotions, which is highly implemented in television, cinemas, and banners. However, adolescents can still access contraband cigarettes or purchase them from street vendors (Colombia Ombudsman Office, 2017). At the time of MECHANISMS data collection, Colombia’s public policy for tobacco control did not include e-cigarettes, which were unregulated until 2024 (Malagón-Rojas, 2024).

### 2.3 Interventions

The ASSIST and Dead Cool programs have previously been shown to effectively reduce rates of adolescent smoking initiation (Campbell et al., 2008; Thurston et al., 2019). The ASSIST program is based on the diffusion of innovations theory (Rogers, 2003), and works on the principles of peer education and diffusion. It is designed to train the most influential pupils in the school year group, nominated in a ‘Peer Questionnaire’ at baseline, to use informal contacts with their peers – other pupils in their school year group – to encourage them not to smoke (Campbell et al., 2008). Dead Cool is a skills-based program based on the theory of planned behavior (Ajzen, 1991). It includes training for schoolteachers and provision of program resources – lesson plans, pupil workbooks, fact sheets and a DVD – to enhance pupils’ knowledge of potential influences on smoking from family, friends, and the media (Thurston et al., 2019; Dunne et al., 2016). Pupils participate in eight classroom-based sessions during which they watch DVD clips of adolescents discussing smoking-related issues, and complete various workbook and group activities (Thurston et al., 2019; Dunne et al., 2016).

### 2.4 Incentivized experiments

The behavioral economics and game theory experiments included several tasks that used monetary incentives as part of the experimental design (Kimbrough and Vostroknutov, 2016; Kimbrough and Vostroknutov, 2018; Krupka and Weber, 2013). Part 1 included a rule-following task measuring individuals’ sensitivities to the effects of social norms (Kimbrough and Vostroknutov, 2016; Kimbrough and Vostroknutov, 2018). Participants were given 5 min to sequentially allocate 50 balls across two buckets (one blue and one yellow), following an arbitrary rule with explicit monetary costs. Participants were told that “The rule is to put the balls in the blue bucket” (Kimbrough and Vostroknutov, 2018). They were also informed that they would receive £0.05 (NI; *COP* $100 Bogotá) for every ball they put in the blue bucket and £0.10 (NI; *COP* $200 Bogotá) for every ball they put in the yellow bucket. Individuals’ norm sensitivities were elicited as the number of balls allocated to the rule-following bucket (‘rule-following’). Parts 2 and 3 included incentivized co-ordination games measuring injunctive and descriptive norms for smoking and vaping in whole school year groups (Krupka and Weber, 2013). Participants were informed they would receive a payment if their response to a randomly selected question matched the most common answer in their school year group. Injunctive norms, defined as shared beliefs about what actions people *ought* to take (Krupka and Weber, 2013), were assessed by asking participants to rate the social appropriateness of eight smoking and vaping-related scenarios (“extremely socially inappropriate” to “extremely socially appropriate”; P2S2–9). Descriptive norms, defined as shared beliefs about what actions people *actually do* take (Krupka and Weber, 2013), were assessed by asking participants to estimate the proportion of peers in the year group who would be accepting of a close friend smoking or vaping (“none of my peers” to “all of my peers”; P3Q1–2). Part 4 assessed participants’ willingness to pay to support prevention interventions that promote anti-smoking norms. Participants were given ten virtual tokens of equal monetary value, asked how many they wanted to donate to the smoking prevention program delivered in their school, and informed they would receive a payment equal to the amount not donated (‘Donation to ASSIST/Dead Cool’). Participants received participation fees of £5.00 (NI; *COP*$5.000 Bogotá) and could earn money in each part of the experiment (maximum £30 NI, *COP*$50.000 Bogotá) depending on their answers and answers provided by their peers. Payments were received after the follow-up experiment.

Further details on the experimental protocol and outcomes are available in the Supplementary File 1: Methods and Supplementary File 1: Table S1.2. Higher numerical values represented greater norm sensitivities (rule-following), more pro-smoking injunctive and descriptive norms, and higher donations to ASSIST/Dead Cool. Table 1 shows the smoking and vaping scenarios.

**Table 1:** Baseline and follow-up summary statistics.

Outcomes^a^	Northern Ireland (*N* = 6)		Bogotá (*N* = 6)		All schools (*N* = 12)	
	Baseline	Follow-up	Baseline	Follow-up	Baseline	Follow-up
Experiment, n	625	620	646	631	1,271	1,251
Survey, n	630	590	644	619	1,274	1,209
Carbon monoxide readings, n	591	591	648	620	1,239	1,211
Experiment Part 2 (injunctive social norms; *α* = 0.78; −1 = “extremely socially inappropriate” to +1 = “extremely socially appropriate”)						
P2S2: Parent smoking in their own home in front of children under the age of 5.	−0.8 (0.3)	−0.8 (0.4)	−0.9 (0.2)	−0.9 (0.2)	−0.9 (0.3) [−1]	−0.8 (0.3) [−1]
P2S3: An adult smoking in a car with children under the age of 16 in the car.	−0.7 (0.4)	−0.7 (0.4)	−0.7 (0.3)	−0.7 (0.3)	−0.7 (0.4) [−0.6]	−0.7 (0.3) [−0.6]
P2S4: Someone selling cigarettes to a teenager who looks younger than 16 without requesting proof of age.	−0.9 (0.3)	−0.8 (0.3)	−0.9 (0.3)	−0.8 (0.3)	−0.9 (0.3) [−1]	−0.8 (0.3) [−1]
P2S5: In a recent superhero movie the lead actor is seen smoking in the opening scene.	−0.3 (0.4)	−0.3 (0.4)	−0.4 (0.4)	−0.4 (0.4)	−0.4 (0.4) [−0.2]	−0.3 (0.4) [−0.2]
P2S6: An older student from school is smoking outside school, for example, at a bus stop.	−0.5 (0.4)	−0.5 (0.4)	−0.5 (0.4)	−0.5 (0.4)	−0.5 (0.4) [−0.6]	−0.5 (0.4) [−0.6]
P2S7: A pupil from school is using an e-cigarette while walking to school.	−0.5 (0.4)	−0.5 (0.4)	−0.5 (0.4)	−0.5 (0.4)	−0.5 (0.4) [−0.6]	−0.5 (0.4) [−0.6]
P2S8: A pupil from school shares a photograph of him/herself using an e-cigarette on social media.	−0.5 (0.4)	−0.5 (0.4)	−0.4 (0.4)	−0.4 (0.4)	−0.5 (0.4) [−0.6]	−0.5 (0.4) [−0.6]
P2S9: A pupil from school is chewing tobacco.	−0.8 (0.4)	−0.7 (0.4)	−0.8 (0.3)	−0.7 (0.3)	−0.8 (0.3) [−1]	−0.7 (0.4) [−1]
Experimental injunctive norms scale (average P2S2 to P2S9).	−0.6 (0.3)	−0.6 (0.3)	−0.7 (0.2)	−0.6 (0.2)	−0.6 (0.2) [−0.7]	−0.6 (0.3) [−0.6]
Experiment Part 3 (descriptive social norms; *α* = 0.85; *α* = 0.85; −1 = “none of my peers” to +1 = “all of my peers”)						
P3Q1: Proportion of school year group accepting of a close friend smoking.	−0.5 (0.5)	−0.3 (0.5)	−0.5 (0.5)	−0.3 (0.5)	−0.5 (0.5) [−0.6]	−0.3 (0.5) [−0.6]
P3Q2: Proportion of school year group accepting of a close friend vaping.	−0.3 (0.6)	−0.2 (0.6)	−0.4 (0.5)	−0.3 (0.6)	−0.4 (0.6) [−0.6]	−0.2 (0.6) [−0.2]
Experimental descriptive norms scale (average P3Q1 to P3Q2).	−0.4 (0.5)	−0.3 (0.5)	−0.5 (0.5)	−0.3 (0.5)	−0.4 (0.5) [−0.6]	−0.3 (0.5) [−0.4]
Experiment Part 4 (willingness to pay to support anti-smoking norms; 0 = “0 tokens donated to ASSIST/Dead Cool” to 10 = “10 tokens donated to ASSIST/Dead Cool”)						
Donation to ASSIST/Dead Cool (0 to 10)	3.5 (3.1)	3.0 (2.8)	3.9 (2.6)	3.6 (2.4)	3.7 (2.9) [4]	3.3 (2.6) [3]
Survey: Self-report injunctive social norms (*α* = 0.75; −2 = “think(s) that I definitely should smoke” to +2 = “think(s) that I definitely should not smoke”)						
IN1: Most of the people who are important to me.	1.7 (0.7)	1.7 (0.7)	1.8 (0.7)	1.7 (0.8)	1.7 (0.7) [2]	1.7 (0.7) [2]
IN2: Mother.	1.9 (0.3)	1.9 (0.4)	1.9 (0.4)	1.9 (0.5)	1.9 (0.4) [2]	1.9 (0.4) [2]
IN3: Father.	1.8 (0.6)	1.8 (0.6)	1.7 (0.7)	1.7 (0.7)	1.7 (0.7) [2]	1.7 (0.7) [2]
IN4: Brother(s).	1.4 (0.9)	1.4 (0.9)	1.4 (0.9)	1.5 (0.8)	1.4 (0.9) [2]	1.4 (0.9) [2]
IN5: Sister(s).	1.4 (0.9)	1.4 (0.9)	1.3 (0.9)	1.4 (0.9)	1.4 (0.9) [2]	1.4 (0.9) [2]
IN6: Friends.	1.5 (0.9)	1.5 (0.9)	1.3 (0.9)	1.3 (0.9)	1.4 (0.9) [2]	1.4 (0.9) [2]
IN7: Best friend.	1.7 (0.7)	1.7 (0.8)	1.5 (0.9)	1.5 (0.9)	1.6 (0.8) [2]	1.6 (0.8) [2]
Self-report injunctive norms scale (average IN1 to IN7).	1.6 (0.5)	1.6 (0.5)	1.5 (0.5)	1.6 (0.5)	1.6 (0.5) [1.7]	1.6 (0.5) [1.7]
Survey: Self-report descriptive social norms 1 (*α* = 0.54; 1 = “smoke(s) very often” to 5 = “never smoke(s)”/“do not know”)						
DN1.1: Best friend.	4.8 (0.8)	4.7 (0.8)	4.9 (0.6)	4.8 (0.6)	4.8 (0.7) [5]	4.8 (0.7) [5]
DN1.2: Mother.	4.2 (1.4)	4.3 (1.3)	4.6 (0.9)	4.6 (0.9)	4.4 (1.2) [5]	4.5 (1.1) [5]
DN1.3: Father.	4.1 (1.4)	4.2 (1.4)	4.4 (1.1)	4.5 (1.1)	4.3 (1.3) [5]	4.3 (1.3) [5]
DN1.4: Brother(s).	4.7 (0.9)	4.7 (0.9)	4.7 (0.8)	4.7 (0.9)	4.7 (0.8) [5]	4.7 (0.9) [5]
DN1.5: Sister(s).	4.8 (0.7)	4.8 (0.8)	4.8 (0.7)	4.8 (0.7)	4.8 (0.7) [5]	4.8 (0.7) [5]
Self-report descriptive norms scale 1 (average DN1.1 to DN1.5).	4.5 (0.7)	4.5 (0.7)	4.7 (0.5)	4.7 (0.5)	4.6 (0.6) [5]	4.6 (0.6) [5]
Survey: Self-report descriptive social norms 2 (*α* = 0.53; 1 = “almost all of them smoke” to 5 = “almost none of them smoke”/“do not know”)						
DN2.1: Friends.	4.7 (0.7)	4.6 (0.8)	4.7 (0.6)	4.7 (0.7)	4.7 (0.7) [5]	4.7 (0.8) [5]
DN2.2: Other family members.	4.1 (1.0)	4.1 (1.1)	4.4 (0.9)	4.5 (0.9)	4.3 (1.0) [5]	4.3 (1.0) [5]
DN2.3: Classmates.	4.7 (0.7)	4.6 (0.7)	4.8 (0.5)	4.8 (0.6)	4.8 (0.6) [5]	4.7 (0.7) [5]
Self-report descriptive norms scale 2 (average DN2.1 to DN2.3).	4.5 (0.6)	4.5 (0.7)	4.7 (0.5)	4.6 (0.5)	4.6 (0.5) [4.7]	4.6 (0.6) [4.7]
Survey: Self-report smoking behavior, intentions, and susceptibility						
Smoking behavior (1 = “sometimes smoke” to 4 = “never smoked”).	3.8 (0.6)	3.8 (0.7)	3.7 (0.7)	3.6 (0.7)	3.8 (0.6) [4]	3.7 (0.7) [4]
Intentions (1 = “I am a smoker” to 6 = “definitely remain a non-smoker”).	5.7 (0.8)	5.7 (0.9)	5.5 (1.1)	5.3 (1.3)	5.6 (1.0) [6]	5.5 (1.1) [6]
Susceptible to commencing smoking, n(%).	199 (28.8%)	199 (28.8%)	259 (39.7%)	315 (48.2%)	458 (34.1%)	514 (38.2%)
Survey: Self-report smoking knowledge and attitudes						
Knowledge (0 = “0 correct” to 6 = “6 correct”).	3.0 (1.5)	3.3 (1.5)	2.2 (1.4)	2.5 (1.5)	2.6 (1.5) [3]	2.9 (1.5) [3]
Attitudes (1 = “least anti-smoking” to 5 = “most anti-smoking”; α = 0.81).	4.0 (0.6)	4.0 (0.6)	3.9 (0.7)	3.9 (0.7)	3.9 (0.6) [4]	3.9 (0.7) [4]
Survey: Self-report psycho-social antecedents						
Self-efficacy (Emotional; 1 = “least self-efficacy to resist smoking” to 6 = “greatest self-efficacy to resist smoking”; α = 0.97).	5.7 (0.8)	5.7 (0.9)	5.6 (0.8)	5.4 (0.9)	5.6 (0.8) [6]	5.5 (0.9) [6]
Self-efficacy (Friends; 1 to 6; α = 0.96).	5.7 (0.8)	5.7 (0.8)	5.6 (0.7)	5.5 (0.9)	5.6 (0.8) [6]	5.6 (0.8) [6]
Self-efficacy (Opportunity; 1 to 6; α = 0.98).	5.8 (0.6)	5.8 (0.6)	5.7 (0.6)	5.6 (0.8)	5.8 (0.6) [6]	5.7 (0.7) [6]
Perceived physical risks (0% = “lowest perceived risk” to 100% = “highest perceived risk”; α = 0.87).	62.5 (21.6)	66.0 (20.4)	59.4 (26.5)	62.9 (25.3)	60.9 (24.2) [62.9]	64.4 (23.1) [67.4]
Perceived social risks (0 to 100%; α = 0.71).	75.1 (22.0)	75.9 (22.2)	61.5 (29.1)	63.8 (26.8)	68.1 (26.8) [72.7]	69.7 (25.4) [73.3]
Perceived addiction risks (0 to 100%; α = 0.49).	43.4 (24.9)	47.5 (24.0)	27.7 (24.9)	30.2 (25.1)	35.2 (26.1) [33.3]	38.9 (26.1) [37.3]
Perceived benefits (0% = “lowest perceived benefit” to 100% = “highest perceived benefit”; α = 0.79).	23.4 (22.1)	24.0 (20.9)	23.8 (21.1)	23.7 (22.0)	23.6 (21.5) [19.8]	23.8 (21.5) [20.0]
Perceived behavioral control (easy to quit; 1 = “strongly disagree” to 5 = “strongly agree”).	2.5 (1.4)	2.4 (1.4)	3.5 (1.3)	3.5 (1.3)	3.0 (1.4) [3]	3.0 (1.5) [3]
Perceived behavioral control (to avoid smoking; 1 = “strongly disagree” to 5 = “strongly agree”).	4.3 (1.1)	4.3 (1.0)	4.0 (1.3)	4.0 (1.3)	4.2 (1.2) [5]	4.2 (1.2) [5]
Objectively measured smoking behavior (expelled air carbon monoxide in parts per million)						
Carbon monoxide reading (0 to 30).	1.5 (1.4)	2.0 (1.0)	3.4 (1.5)	3.5 (1.7)	2.5 (1.7) [3]	2.8 (1.6) [2]
Moderators						
Setting						
*Number of schools, N*	6		6		12	
*Number of classes, N*	31		24		55	
*Number of pupils, n*	718		726		1,444	
Intervention, *N* (schools)/n (pupils)						
*ASSIST schools*	*N* = 3/*n* = 423		*N* = 3/*n* = 373		*N* = 6/*n* = 796	
*Dead Cool schools*	*N* = 3/*n* = 295		*N* = 3/*n* = 353		*N* = 6/*n* = 648	
Participation, *n* (%)	691 (96.2%)		653 (89.9%)		1,344 (93.1%)	
School socio-economic status (1 = lowest to 4 = highest)^b^.	1.9 (1.0)		2.5 (0.5)		2.2 (0.9) [2.0]	
*School MDM (NI only: 5.7 to 80.2)^c^*.	34.4 (23.3)		–		34.4 (23.3) [29.2]	
Gender, *n* (%)						
*Boys*	298 (43.1%)	–	327 (50.1%)	–	625 (46.5%)	–
*Girls*	321 (46.5%)	–	312 (47.8%)	–	633 (47.1%)	–
*Prefer not to say*	11 (1.6%)	–	5 (0.8%)	–	16 (1.2%)	–
Experiment Part 1 (Rule-following): Balls allocated to blue bucket (0 = “least rule-following” to 5 = “most rule-following”).	2.9 (1.9)	2.9 (2.0)	3.3 (1.7)	3.3 (1.8)	3.1 (1.8)	3.1 (1.9)
Pro-sociality (0 = “least pro-social” to 10 = “most pro-social”; *α* = 0.74).	8.1 (2.03)	–	7.3 (2.2)	–	7.7 (2.1) [8]	–
Fear of negative evaluation (1 = “least FNE” to 5 = “most FNE”; *α* = 0.89).	2.9 (0.7)	–	2.6 (0.6)	–	2.7 (0.7) [2.7]	–
Need to belong (1 = “least NTB” to 5 = “most NTB”; *α* = 0.81).	3.1 (0.6)	–	2.8 (0.6)	–	3.0 (0.6) [3]	–
‘Big Five’: Openness (0 = “least openness” to 4 = “most openness”; *α* = 0.80).	2.4 (0.6)	–	2.7 (0.7)	–	2.6 (0.7) [2.6]	–
‘Big Five’: Extraversion (0 = “least extraverted” to 4 = “most extraverted”; *α* = 0.78).	2.5 (0.8)	–	2.7 (0.7)	–	2.6 (0.7) [2.7]	–
‘Big Five’: Agreeableness (0 = “least agreeable” to 4 = “most agreeable”; *α* = 0.70).	2.5 (0.6)	–	2.6 (0.7)	–	2.6 (0.7) [2.5]	–
‘Big Five’: Conscientiousness (0 = “least conscientious” to 4 = “most conscientious”; α = 0.70).	2.3 (0.7)	–	2.4 (0.7)	–	2.3 (0.7) [2.2]	–
‘Big Five’: Emotional stability (0 = “least stable” to 4 = “most stable”; α = 0.74).	1.9 (0.8)	–	2.1 (0.7)	–	2.0 (0.8) [2]	–
Clustering coefficient (0 to 10)^d^.	3.5 (2.3)	3.2 (2.4)	3.3 (2.2)	3.5 (2.5)	3.4 (2.3) [3.3]	3.3 (2.4) [3.1]
Eigenvector centrality (Baseline: 0.008 to 3.13; Follow-up: 0.003 to 3.04)^d^.	0.7 (0.6)	0.7 (0.6)	0.7 (0.6)	0.7 (0.6)	0.7 (0.6) [0.6]	0.7 (0.6) [0.5]
Closeness centrality (Baseline: 2.24 to 5.10; Follow-up: 2.15 to 4.94)^d^.	3.6 (0.4)	3.5 (0.5)	3.7 (0.5)	3.5 (0.5)	3.7 (0.4) [3.7]	3.5 (0.5) [3.5]
Betweenness centrality (Baseline: 0 to 2.83; Follow-up: 0 to 5.49)^d^.	0.3 (0.3)	0.3 (0.3)	0.3 (0.3)	0.4 (0.5)	0.3 (0.3) [0.2]	0.3 (0.4) [0.2]
Gini degree coefficient (Baseline: 1.70 to 2.67; Follow-up: 1.91 to 2.91)^d^.	2.2 (0.3)	2.3 (0.3)	2.1 (0.3)	2.2 (0.3)	2.1 (0.3) [2.2]	2.2 (0.3) [2.2]
Other self-report socio-demographic variables included as covariates						
Age, *n* (%)						
*11 or 12 years old*	244 (35.3%)	–	223 (34.1%)	–	467 (34.7%)	–
*13 years old*	380 (55.0%)	–	257 (39.4%)	–	637 (47.4%)	–
*14 or 15 years old*	6 (0.9%)	–	174 (26.6%)	–	180 (13.4%)	–
Ethnicity, *n* (%)^e^						
*Ethnic minority*	49 (7.1%)	–	89 (13.6%)	–	138 (10.3%)	–
*No ethnic minority*	579 (83.8%)	–	555 (85.0%)	–	1,134 (84.4%)	–
Socio-economic status (individual pupils), *n* (%)^f^						
*1*	259 (37.5%)	–	343 (52.5%)	–	602 (44.8%)	–
*2*	170 (24.6%)	–	292 (44.7%)	–	462 (34.4%)	–
*3*	133 (19.2%)	–	2 (0.3%)	–	135 (10.0%)	–

Reported results are mean (standard deviation) [median] unless otherwise stated. ^a^Outcomes are defined in Supplementary File 1: Table S1.2. ^b^In Bogotá, school socio-economic status is based on the Socio-economic level index (1 = Lower; 2 = Middle-low; 3 = Middle-high; 4 = Higher). Calculated each year using a sample from each school, based on the characteristics of the home and its infrastructure, some household assets, the relationship of the children with their parents, among other characteristics. Schools are then classified into four levels according to the average of the responses of the pupils enrolled in them. Provided by the Instituto Colombiano para el Fomento de la Educación Superior (ICFES; “Colombian Institute for the Promotion of Higher Education”). In NI, school socio-economic status is based on the NIMDM2017 for each school’s postcode. A four-category variable was created, comparable with the Colombian school SES measure, to examine moderation of peer influence effects across all schools (in NI and Bogotá) in the same model (1 = NIMDM2017 ≤ 222.5; 2 = 222.5 < NIMDM2017 ≤ 445; 3 = 445 < NIMDM2017 ≤ 667.5; 4 = NIMDM2017 > 667.5). ^c^In NI, school socio-economic status is based on the NIMDM2017 for each school’s postcode. For the moderation analyses including NI schools only, the variable was scaled by 10: 5.7 [NIMDM2017 = 57] to 80.2 [NIMDM2017 = 802]. ^d^Higher numerical values for social network clustering coefficients, centrality measures, and Gini degree coefficients indicate greater clustering, centrality, and heterogeneity in network degree distributions. ^e^Northern Ireland: Ethnic minority (African, Asian, Chinese, Any other ethnic group); No ethnic minority (White British, White Irish). Bogotá: Ethnic minority (Indigenous, Gypsy/Roma, Archipelago Raizal, Palenquero of San Basilio, Black/Mulatto/Afro-Colombian/Afro-descendant); No ethnic minority (“None of the above,” i.e., Colombian no ethnic minority). ^f^Northern Ireland: 1 = NIMDM2017 ≤ 296.6; 2 = 296.6 < NIMDM2017 ≤ 593.2; 3 = NIMDM2017 > 593.2. Bogotá: 1 = Informal settlement/Lowest/Low; 2 = Middle-Low/Middle; 3 = Middle-High/High.

### 2.5 Self-report survey, social networks data, and carbon monoxide measurements

Our survey collected socio-demographic information (gender, age, ethnicity, SES), social networks, self-report smoking outcomes, and personality characteristics. In NI, SES for schools and individual pupils was based on the Northern Ireland Multiple Deprivation Measure (NIMDM2017), which ranks postcodes based on seven domains of deprivation (1 = most deprived to 890 = least deprived) (Northern Ireland Statistics and Research Agency, 2017). In Bogotá, SES for schools was based on the socio-economic level index for educational institutions provided by the Colombian Institute for the Promotion of Higher Education (1 = lower to 4 = higher) (Colombian Institute for the Evaluation of Education, 2017). In Bogotá, SES for individual pupils was determined as the socio-economic level index of the household provided by the Colombian National Administrative Department of Statistics (0 = informal settlement, 1 = lowest to 6 = high) (National Administrative Department of Statistics, 2021).

Social networks were assessed by asking pupils to name up to ten of their closest friends in their school year group (Murray et al., 2023; Dunne et al., 2016). Pupils were provided with class rosters and asked to provide the first name, surname, and form class of nominated friends (data were anonymized by the study team).

Self-report smoking outcomes included: injunctive norms (Cremers et al., 2012), descriptive norms (Cremers et al., 2012), past/current smoking behavior (Dunne et al., 2016; Fuller and Hawkins, 2012), intentions and susceptibility (Dunne et al., 2016; Mazanov and Byrne, 2007; Pierce et al., 1998), knowledge of the health effects of smoking (Cremers et al., 2012), attitudes towards smoking (Ganley and Rosario, 2013), self-efficacy to resist smoking (emotional, friends, and opportunity subscales) (Condiotte and Lichtenstein, 1981; Lawrance, 1989), perceived risks of smoking (physical, social, and addiction subscales) (Halpern-Felsher et al., 2004; Song et al., 2009; Aryal et al., 2013), perceived benefits of smoking (Halpern-Felsher et al., 2004; Song et al., 2009; Aryal et al., 2013), and PBC (‘easy to quit smoking’, and ‘to avoid smoking’) (Smith et al., 2006). Self-report injunctive norms were assessed with seven items enquiring about perceived approval of smoking from groups of important others (e.g., “most of the people who are important to me,” “mother,” “father”; IN1-7) (Cremers et al., 2012). Self-report descriptive norms were assessed with five items enquiring about how often groups of important others engaged in smoking behavior, and three items enquiring about the proportion of groups of important others who are smokers (DN1.1–1.5, DN2.1–2.3) (Cremers et al., 2012).

Personality variables collected at baseline included: pro-sociality (Goodman et al., 2003; Bevelander et al., 2018), FNE (Bevelander et al., 2018; Leary, 1983; Collins et al., 2005), NTB (Bevelander et al., 2018; Leary et al., 2013), and the ‘Big Five’ personality traits (Morizot, 2014; Ortet et al., 2017).

Smoking behavior in the last 24 h was objectively measured using hand-held carbon monoxide monitors measuring expelled air carbon monoxide in parts per million (PICOAdvantage Smokerlyzer, Bedfont) (Thurston et al., 2019; Bedfont Scientific Ltd, 2018). Supplementary File 1: Table S1.2 shows details of all measurement instruments. Higher numerical values represented more anti-smoking outcomes on the self-report survey (e.g., more anti-smoking behavior, intentions, attitudes, and higher knowledge of the effects of smoking), apart from perceived benefits and smoking susceptibility (0 = not susceptible, 1 = susceptible). Higher numerical values also represented greater objectively measured smoking behavior.

### 2.6 Moderators

The following variables were examined as moderators:

- Setting: A dichotomous variable representing NI versus Bogotá.
- Intervention: A dichotomous variable representing ASSIST versus Dead Cool.
- Gender: A dichotomous variable representing boys versus girls/prefer not to say (PNTS).
- School socio-economic status: Since SES was measured differently across the two settings, we created a four-category variable for NI based on quartiles of the NIMDM2017, ensuring comparability with the Colombian school SES measure. We also repeated the models examining peer influence from average friends’ responses separately in NI and Bogotá.
- Norm sensitivities and related personality characteristics: Individuals’ norm sensitivities/'rule-following’, pro-sociality, FNE, and NTB.
- ‘Big Five’ personality traits: Openness, extraversion, agreeableness, conscientiousness, and emotional stability.
- Social network parameters: Individuals’ clustering coefficients, eigenvector centralities, closeness centralities, betweenness centralities, and social network Gini degree coefficients.
- Self-efficacy to resist smoking: Emotional, friends, and opportunity self-efficacy.

All moderator variables were measured at baseline. Social network parameters at baseline and follow-up were both considered as moderators depending on whether the peer influence effects were measured at baseline or follow-up.

Higher numerical values represented greater SES and norms sensitivities, higher levels of the personality characteristics, and greater self-efficacy to resist smoking. They also represented higher clustering, centrality, and Gini degree coefficients, corresponding to more heterogeneous networks (Supplementary File 1: Table S1.2). Definitions of the social network parameters are provided in the ‘Social network parameter definitions’ subsection of Supplementary File 1.

### 2.7 Statistical analysis

Analyses were conducted using Stata 13 (StataCorp) (StataCorp, 2013). Descriptive statistics, and Cronbach’s alpha coefficients for individual scales were calculated (Table 1).

Peer influence effects were examined for the following smoking and vaping outcomes which were targeted by the ASSIST and Dead Cool interventions (1): experimentally measured injunctive norms, experimentally measured descriptive norms, experimental donations to ASSIST/Dead Cool, self-report injunctive norms, self-report descriptive norms, self-report smoking behavior, intentions, knowledge, attitudes, self-efficacy, perceived risks, perceived benefits, PBC, objectively measured smoking behavior, and smoking susceptibility.

To examine influence effects from friendship networks, school classes, and school year groups, variables were computed for each outcome at baseline and follow-up, containing: (1) the average responses of each focal participant’s (*i*) friendship network; (2) the average responses of *i*’s school class, excluding *i*; and (3) the average responses of *i*’s school year group, excluding *i* (Krupka et al., 2016). Ordinary least square (OLS) regressions with robust (Huber-White) standard errors (Huber, 1967; White, 1980) were used to examine influence effects, and moderation of influence effects. In addition to the ‘average peer’ predictor variable, the moderator variable and its interaction with the predictor variable were included in each model. We considered a model to show a significant moderating effect if the interaction term reached statistical significance at *p* ≤ 0.01.

Logistic regressions were run with focal participants’ smoking susceptibility at follow-up as the outcome, and robust (Huber White) standard errors (Huber, 1967; White, 1980). Variables were computed at baseline and follow-up containing the percentage of *i*’s friendship network, school class, and school year group, that were susceptible to commencing smoking. In addition to the ‘percentage peer’ predictor variable, the moderator and its interaction with the predictor were included.

Baseline covariates included in each model were gender, age, intervention, ethnicity, individuals’ SES, and baseline values (Murray et al., 2023). Continuous predictor variables, moderator variables, and baseline outcomes were mean-centered. The models were repeated to examine influence effects from average peer responses at baseline and average peer responses at follow-up (Murray et al., 2023).

Variance inflation factors (VIFs) were calculated to examine multi-collinearity (>5 or 10 usually indicates problematic amounts of collinearity). VIFs for ‘setting’ were high for many of the models with school class or year group responses as predictors (Murray et al., 2023). Where high VIFs affected one of the models with setting as a moderator, this is indicated in the footnotes of Supplementary Files 2: Table S2.1 and Supplementary File 3: Table S3.1. VIFs were satisfactory for all other analyses.

Significant interactions (*p* ≤ 0.01) were probed using the simple slopes and Johnson-Neyman techniques (Hayes, 2013; Johnson and Neyman, 1936). For dichotomous moderator variables, the simple slopes technique was used to calculate marginal effects at each level of the moderator (NI versus Bogotá, ASSIST versus Dead Cool, and boys versus girls/PNTS). For continuous moderator variables, the marginal effects were calculated at one standard deviation below (‘low’) and above (‘high’) the mean value of the moderator. Conditional effects were graphed with their 95% confidence intervals (CIs), showing the relationship between the predictor (average ‘peer’ variable) and the predicted value of the outcome at follow-up, as a function of the moderator. Regions of significance were calculated, using the Johnson-Neyman technique (Johnson and Neyman, 1936). These methods allowed us to visualize whether the strength or direction of the peer influence effects changed depending on the value of the moderator.

Due to the large number of tests for each moderator, we adopted a fourfold approach to adjusting for multiple testing. Firstly, we have discussed our results with reference to a significance criterion of *p* ≤ 0.01. Secondly, in the results tables we have highlighted which results would have attained statistical significance at the *p* ≤ 0.05 level after using the Holm-Bonferroni procedure to adjust the *p*-values for multiple testing (Holm, 1979). Thirdly, we used binomial tests to determine whether the number of significant interactions (p ≤ 0.01) observed per moderator was greater than expected by chance. Fourthly, we have used multiverse-style analyses to summarize the distribution of p-values and standardized regression coefficients for interaction effects across the models for each moderator (Steegen et al., 2016; Del and Gangestad, 2021).

For the multiverse-style analyses, we used histograms to show the distribution of *p*-values for interaction effects for each moderator. Volcano plots were constructed showing the relationship between the *p*-values and standardized regression coefficients. Figures were constructed to visualize the distribution of significant interaction effects according to outcome (experimental norms outcomes, self-report norms outcomes, or other smoking outcomes), peer group (friends, school class, or school year group), and peer influence measurement time-point (baseline or follow-up) (Steegen et al., 2016; Del and Gangestad, 2021). Finally, we constructed summary tables and heatmaps to facilitate the narrative synthesis of results for each moderator.

A more detailed description of the statistical methods is provided in the Supplementary File 1: Methods.

## 3 Results

Descriptive statistics for outcomes, moderators, and baseline covariates, are shown in Table 1. Our study sample consisted of 1,344 school pupils (691 in NI, 653 in Bogotá). The pupils were aged between 11 and 15 years (81.6% were aged 12–13 years), 46.5% were boys (47.1% were girls, 1.2% PNTS), 80.6% were self-reported “never smokers” and 34.1% were susceptible to commencing smoking at baseline.

The results section begins with a high-level summary of the results for each moderator. A more detailed description of the models with significant interactions (*p* ≤ 0.01) is then provided, organized by groups of moderators. Unstandardized regression coefficients for all models are reported in Supplementary File 2. Marginal effects and regions of significance for models with significant interactions are reported in Supplementary File 3.

Throughout the results section, we have presented examples of our graphs showing the conditional effects of peer influence on smoking and vaping outcomes at various levels of the moderator (one example for a dichotomous moderator and one example for a continuous moderator), and examples of our multiverse-style graphs for models with setting as the moderator. The remainder of the conditional effects and multiverse-style graphs are presented in Supplementary Files 4, 5.

**Figure 3:**
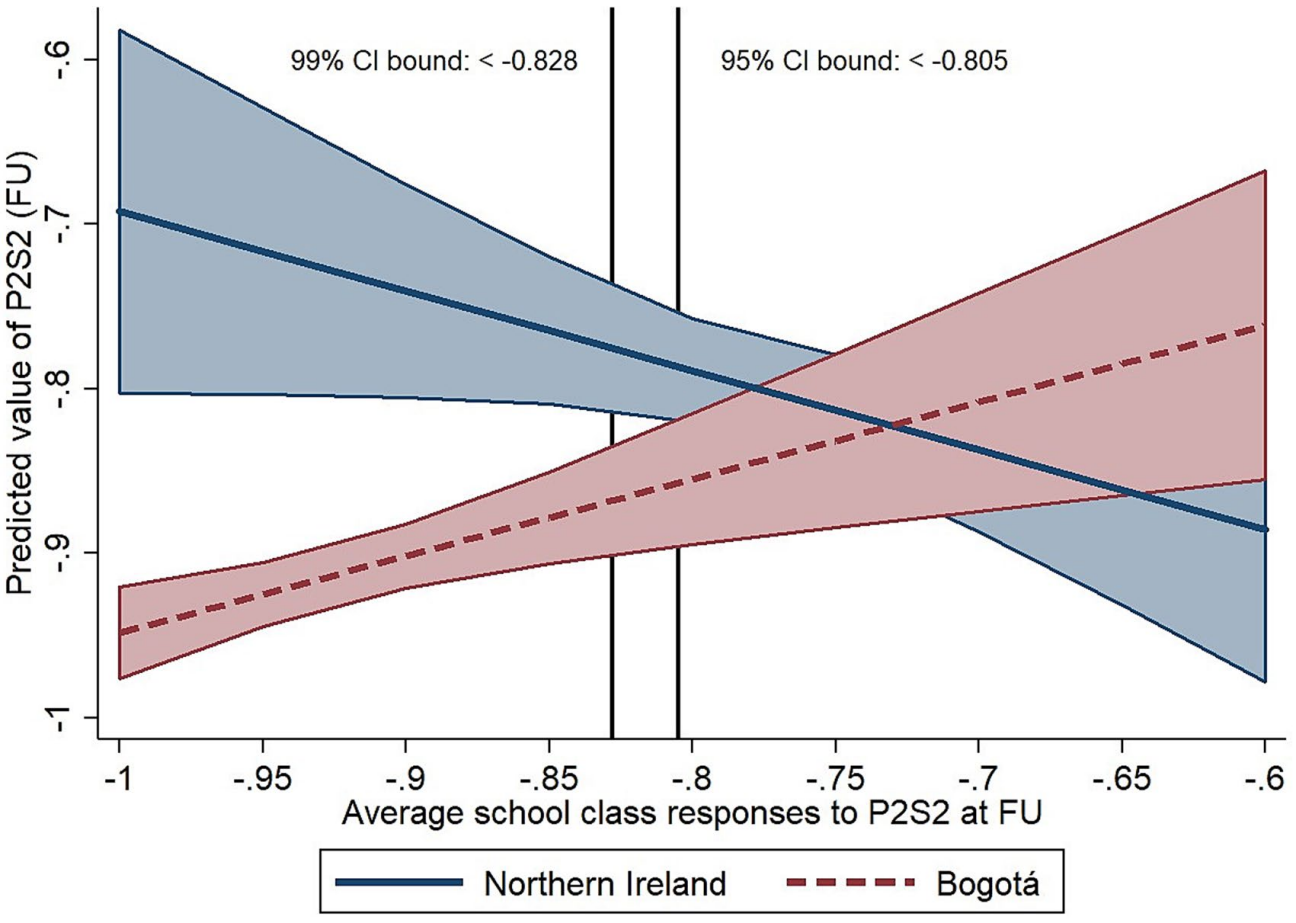
Example of a conditional effects graph for a dichotomous moderator. The graph shows the conditional effects of peer influence from average school class responses for experimental injunctive norms item P2S2 at follow-up (predictor) on focal participants’ values of P2S2 at follow-up (outcome) for participants in NI and Bogotá (moderator: Setting) with 95% CI limits for each slope, and bounds indicating regions of significance at the 95 and 99% levels (indicating values of the predictor for which the slopes differ significantly for NI and Bogotá). P2S2: Social appropriateness ratings for “a parent smoking in their own home in front of children under the age of 5″ (−1 = “extremely socially inappropriate” to +1 = “extremely socially appropriate”).

**Figure 4:**
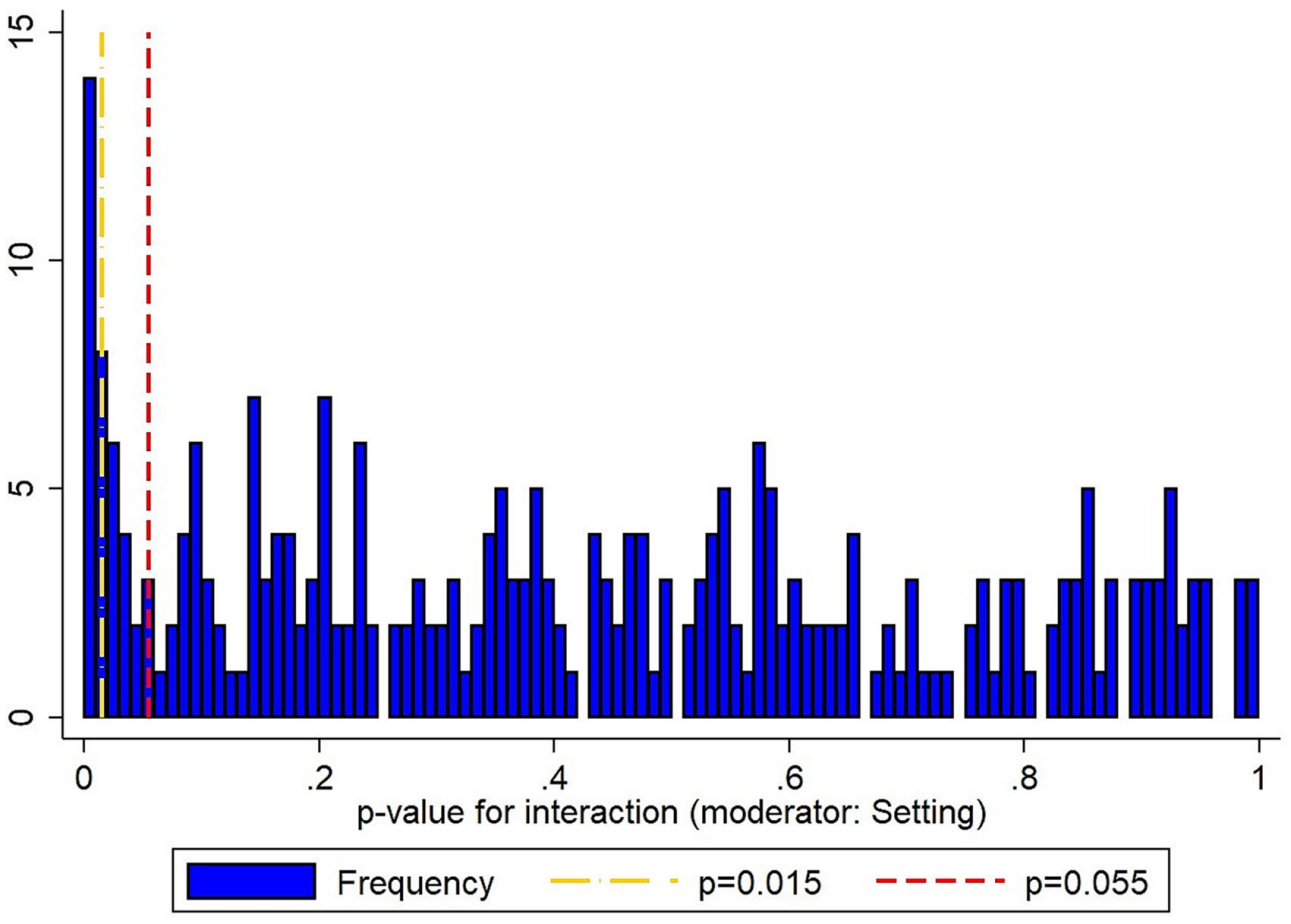
Histogram showing the distribution of *p*-values for interaction effects across the 276 models with setting as the moderator (mean = 0.43, median = 0.40, 20 [7.2%] at *p* ≤ 0.01, 35 [11.6%] at *p* ≤ 0.05). Figure 6 shows the differences between the 276 models (in terms of their outcome variables and predictor variables) with the individual *p*-values displayed in a grid format.

**Figure 5:**
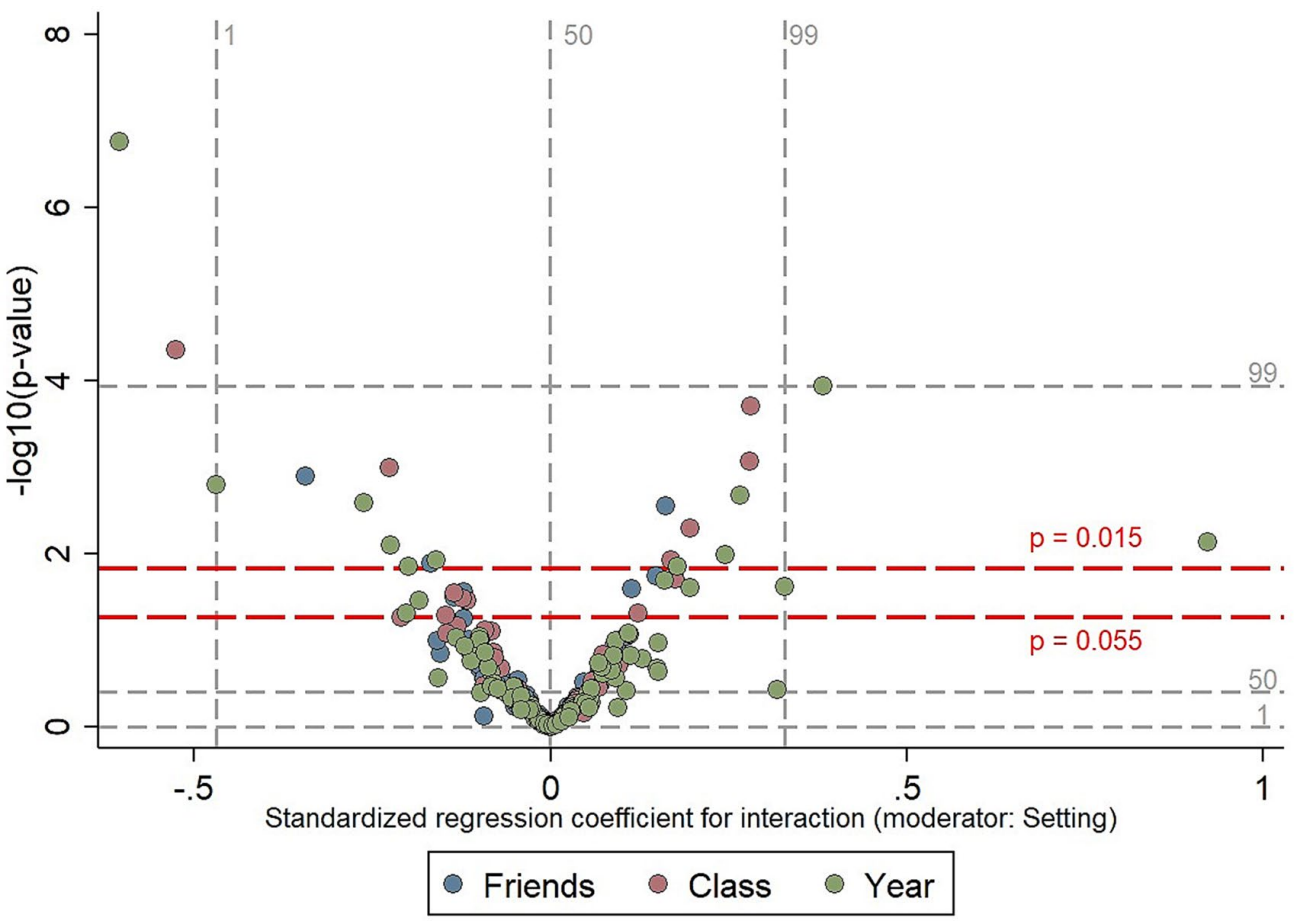
Volcano plot for the 270 models with setting as the moderator. The negative logs of the p-values for the interaction effects are shown on the *y*-axis (larger values on the *y*-axis correspond to smaller *p*-values). Standardized regression coefficients for the interaction effects are shown on the *x*-axis (the estimated difference in standard deviations of the outcome variable between two cases that differ by one standard deviation on the ‘peer group average’ predictor variable, for pupils in Bogotá compared to pupils in Northern Ireland). The red dashed lines show the cut-off points where *p* = 0.015 and *p* = 0.055, with observations lying above the lines attaining statistical significance at the *p* ≤ 0.01 and *p* ≤ 0.05 levels, respectively. The gray dashed lines show the 1st, 50th, and 99th percentiles of the distributions of *p*-values and the standardized regression coefficients (mean = −0.003, median = −0.001, 1st percentile = −0.47, 99th percentile = 0.33). The results of logistic regression models including smoking susceptibility as the outcome variable are not included in the plot.

**Figure 6:**
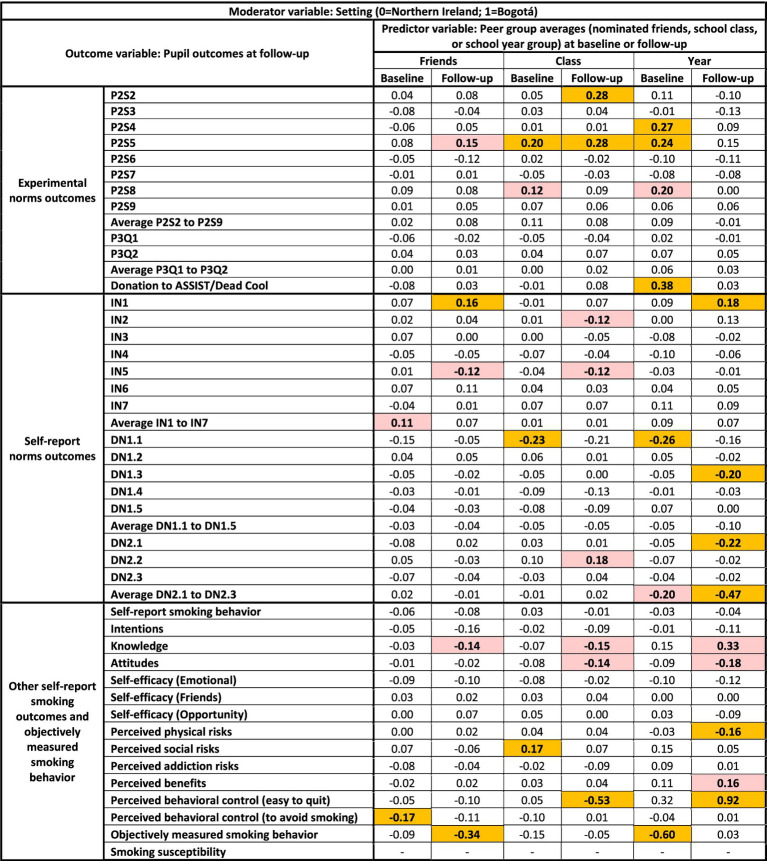
Visualization of the multiverse of standardized regression coefficients for interaction effects in the 270 models with setting as the moderator. Standardized regression coefficients can be interpreted as the estimated difference in standard deviations of the outcome variable between two cases that differ by one standard deviation on the ‘peer group average’ predictor variable, for pupils in Bogotá compared to pupils in Northern Ireland. Logistic regression models including smoking susceptibility as the outcome variable are not included. Cells highlighted in gold indicate *p* ≤ 0.01. Cells highlighted in red indicate *p* ≤ 0.05. For example, the result “0.04” in the top lefthand side of the figure indicates that the estimated difference in standard deviations of the outcome P2S2 at follow-up (outcome variable) between two pupils who differ by one standard deviation on the average of their nominated friends’ P2S2 scores at baseline (predictor variable) was 0.04 standard deviations higher for pupils in Bogotá compared to Northern Ireland.

**Figure 7:**
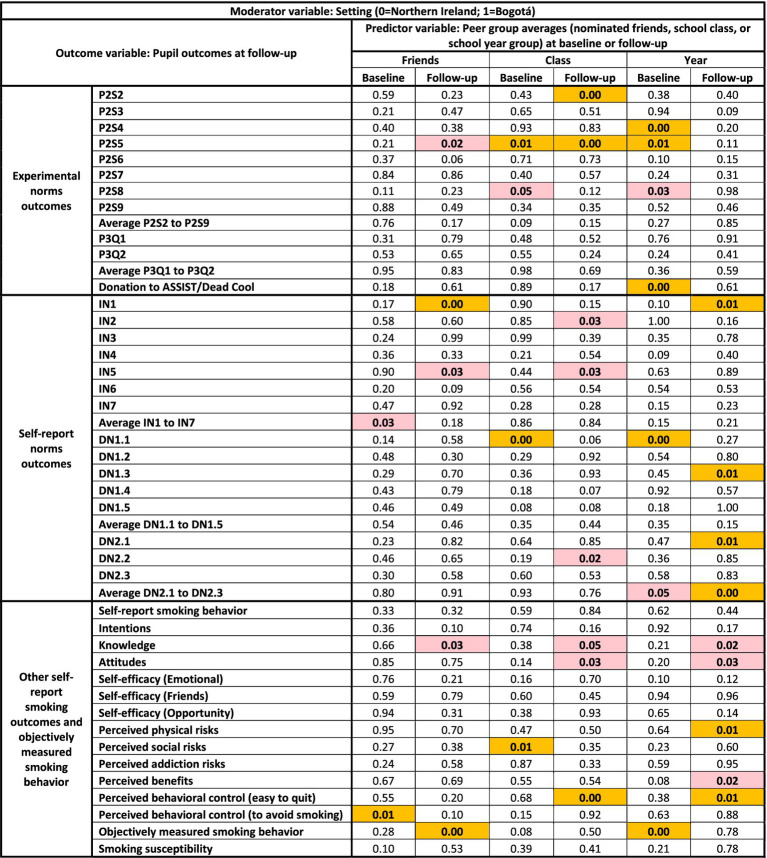
Visualization of the multiverse of p-values for interaction effects in the 276 models with setting as the moderator. Logistic regression models including smoking susceptibility as the outcome variable are included. Cells highlighted in gold indicate *p* ≤ 0.01. Cells highlighted in red indicate *p* ≤ 0.05.

**Figure 8:**
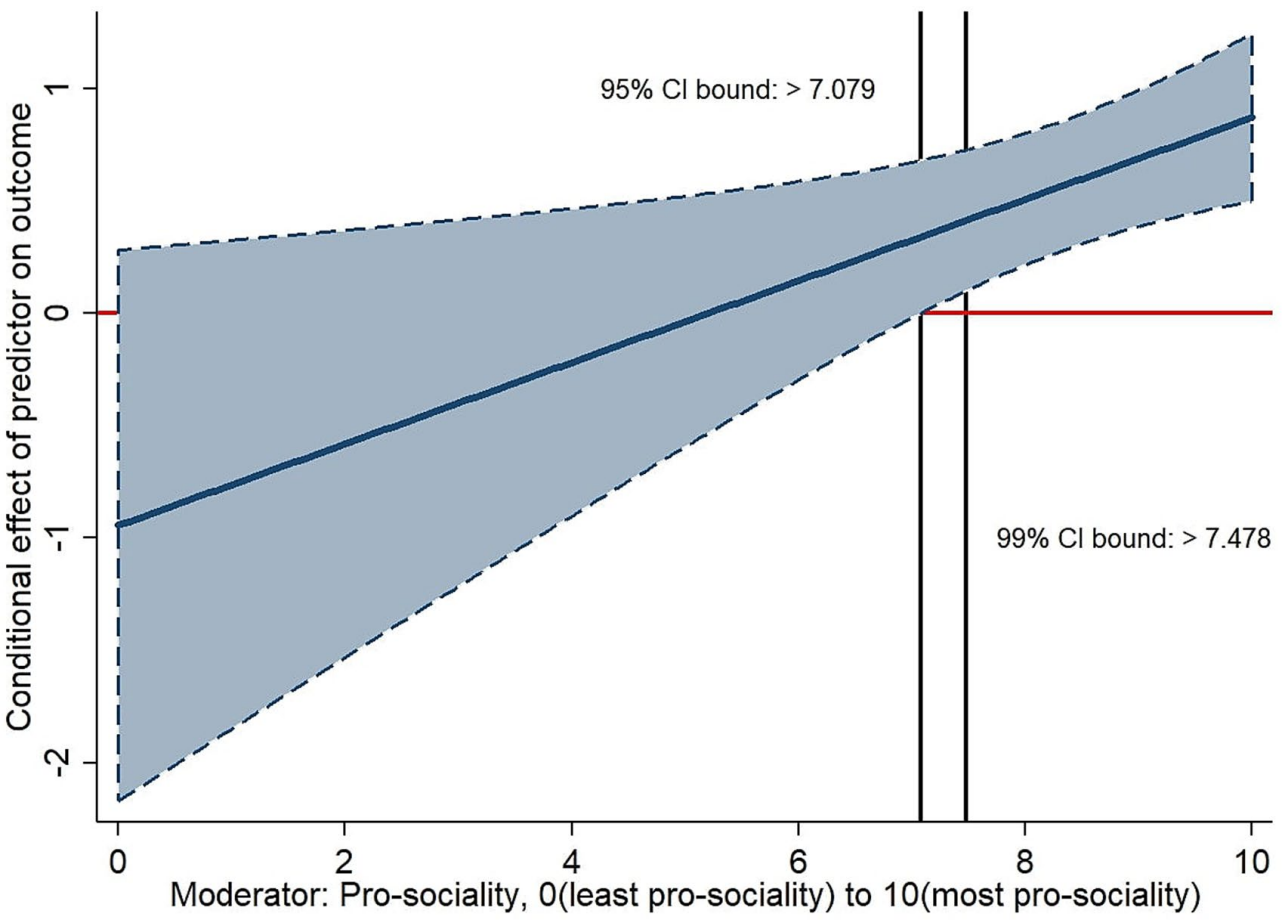
Example of a conditional effects graph for a continuous moderator. The graph shows the conditional effects of peer influence from average school year group responses for experimental injunctive norms item P2S6 at follow-up (predictor) on focal participants’ values of P2S6 at follow-up (outcome) by pro-sociality (moderator) with 95% CI limits for conditional effects, and bounds indicating regions of significance at the 95 and 99% levels (indicating values of the moderator for which conditional effects differ significantly from 0). P2S6: Social appropriateness ratings for “an older student from school is smoking outside school, for example, at a bus stop” (−1 = “extremely socially inappropriate” to +1 = “extremely socially appropriate”).

### 3.1 Summary of the main results

Table 2 shows a summary of the main results including the binomial tests and our overall conclusions about each moderator. The binomial tests showed evidence that setting was a significant moderator of the peer influence effects, but they provided no indication of whether peer influence was stronger in NI or Bogotá overall. Intervention was a significant moderator, and there was some indication that the peer influence effects were stronger in ASSIST schools compared to Dead Cool overall. Gender was a significant moderator and there was evidence that the peer influence effects were stronger for girls/PNTS compared to boys.

**Table 2:** Overview of results for each moderator.

Moderator^a^	Description of moderator^b^	Hypothesized direction^c^	Number of models	Number (%) of significant interactions (*p* ≤ 0.01)				Direction of results^d^	Outcome variables	Δ*R*^2e^	Binomial tests		
				Total	Friends	Class	Year				Overall^f^	Direction^g^	Conclusion
Setting	Dichotomous. 0 = NI, 1 = Bogotá.	Bogotá.	276	20 (7.2%)	3 (3.3%)	6 (6.5%)	11 (12.0%)	10 NI. 10 Bogotá.	NI: self-report descriptive norms, perceived physical risks, PBC, objectively measured smoking behavior. Bogotá: experimental injunctive norms, experimental donations to ASSIST/DC, self-report injunctive norms, perceived social risks, PBC.	0.0022–0.0128	B (276,20,0.01), *p* < 0.0001.	NI: B (276,10,0.01), *p* = 0.0005. Bogotá: B (276,10,0.01), *p* = 0.0005.	Significant moderator. No indication if PI is stronger in NI or Bogotá overall.
Intervention	Dichotomous. 1 = ASSIST, 2 = DC.	ASSIST.	276	11 (4.0%)	4 (4.3%)	4 (4.3%)	3 (3.3%)	7 ASSIST. 4 DC.	ASSIST: experimental donations to ASSIST/DC, self-report injunctive and descriptive norms, smoking susceptibility. DC: experimental injunctive norms, attitudes, objectively measured smoking behavior.	0.0039–0.0098	B (276,11,0.01), *p* = 0.0001.	ASSIST: B (276,7,0.01), *p* = 0.02. DC: B (276,4,0.01), *p* = 0.30.	Significant moderator. Some indication that PI is stronger for ASSIST vs. DC.
Gender	Dichotomous. 0 = boy, 1 = girl/PNTS.	Girls/PNTS.	276	18 (6.5%)	5 (5.4%)	6 (6.5%)	7 (7.6%)	6 boys. 12 girls/PNTS.	Boys: self-report injunctive norms, perceived physical risks. Girls/PNTS: experimental injunctive and descriptive norms, self-report descriptive norms, intentions, perceived addiction risks.	0.0039–0.0112	B (276,18,0.01), *p* < 0.0001.	Boys: B (276,6,0.01), *p* = 0.06. Girls/PNTS: B (276,12,0.01), *p* < 0.0001.	Significant moderator. PI is stronger for girls/PNTS vs. boys.
School SES	Continuous. 1 to 4.	↓	276	15 (8.7%)	5 (5.4%)	6 (6.5%)	4 (4.3%)	6 ↑ 9 ↓	↑: experimental injunctive norms (P2S4), self-report injunctive norms (IN1, scale), self-efficacy. ↓: experimental injunctive norms (P2S6), self-report injunctive norms (IN4, IN5), self-report descriptive norms.	0.0035–0.0160	B (276,15,0.01), *p* < 0.0001.	↑: B (276,6,0.01), *p* = 0.06. ↓: B (276,9,0.01), *p* = 0.002.	Significant moderator. PI is stronger in schools with lower SES.
School SES (NI)	Continuous. 5.7 to 80.2.	↓	92	8 (8.7%)	8 (8.7%)	N/A.	N/A.	1 ↑ 7 ↓	↑: self-report injunctive norms (IN1). ↓: self-report injunctive norms (IN5), self-report smoking behavior, self-efficacy, perceived physical risks.	0.0061–0.0188	B (92,8,0.01), *p* < 0.0001.	↑: B (92,1,0.01), *p* = 0.60. ↓: B (92,7,0.01), *p* < 0.0001.	Significant moderator. PI is stronger in NI schools with lower SES.
School SES (Bogotá)	Continuous. 1 to 4.	↓	92	4 (4.3%)	4 (4.3%)	N/A.	N/A.	3 ↑ 1 ↓	↑: objectively measured smoking behavior (FU), self-report injunctive and descriptive norms. ↓: objectively measured smoking behavior (baseline).	0.0058–0.0214	B (92,4,0.01), *p* = 0.01.	↑: B (92,3,0.01), *p* = 0.07. ↓: B (92,1,0.01), *p* = 0.60.	Some evidence that school SES is a significant moderator in Bogotá. No indication whether PI is stronger at higher or lower school SES.
Rule-following	Continuous. 0 to 5.	↑	276	5 (1.8%)	0 (0.0%)	2 (2.2%)	3 (3.3%)	2 ↑ 3 ↓	↑: experimental injunctive norms, PBC (easy to quit). ↓: perceived physical risks, PBC (to avoid).	0.0045–0.0067	B (276,5,0.01), *p* = 0.15.	N/A.	Not a significant moderator.
Pro-sociality	Continuous. 0 to 10.	↑	276	14 (5.1%)	5 (5.4%)	4 (4.3%)	5 (5.4%)	11 ↑ 3 ↓	↑: experimental injunctive norms (P2S6, P2S7, scale), self-report descriptive norms, intentions, perceived risks. ↓: experimental injunctive norms (P2S9), self-report injunctive norms, self-efficacy.	0.0038–0.0083	B (276,14,0.01), p < 0.0001.	↑: B (276,11,0.01), *p* = 0.0001. ↓: B (276,3,0.01), *p* = 0.52.	Significant moderator. PI is stronger at higher pro-sociality.
FNE	Continuous. 1 to 5.	↑	276	8 (2.9%)	4 (4.3%)	2 (2.2%)	2 (2.2%)	8 ↑ 0 ↓	↑: experimental injunctive norms, self-report descriptive norms, self-report smoking behavior, self-efficacy, PBC.	0.0027–0.0098	B (276,8,0.01), *p* = 0.007.	↑: B (276,8,0.01), *p* = 0.007. ↓: B (276,0,0.01), *p* = 1.00.	Significant moderator. PI is stronger at higher FNE.
NTB		↑	276	6 (2.2%)	5 (5.4%)	1 (1.1%)	0 (0.0%)	5 ↑ 1 ↓	↑: self-report injunctive and descriptive norms, self-report smoking behavior, self-efficacy. ↓: experimental injunctive norms.	0.0038–0.0099	B (276,6,0.01), *p* = 0.06.	N/A.	Not a significant moderator.
Openness	Continuous. 0 to 4.	Exploratory.	276	6 (2.2%)	4 (4.3%)	0 (0.0%)	2 (2.2%)	6 ↑ 0 ↓	↑: experimental injunctive norms.	0.0045–0.0063	B (276,6,0.01), *p* = 0.06.	N/A.	Not a significant moderator.
Extraversion			276	10 (3.6%)	2 (2.2%)	4 (4.3%)	4 (4.3%)	7 ↑ 3 ↓	↑: experimental injunctive norms. ↓: intentions, smoking susceptibility.	0.0049–0.0087	B (276,10,0.01), *p* = 0.0005.	↑: B (276,7,0.01), *p* = 0.02. ↓: B (276,3,0.01), *p* = 0.52.	Significant moderator. PI is stronger at higher extraversion.
Agreeableness			276	4 (1.4%)	1 (1.1%)	1 (1.1%)	2 (2.2%)	1 ↑ 3 ↓	↑: self-report injunctive norms. ↓: experimental injunctive norms, self-report smoking behavior.	0.0028–0.0203	B (276,4,0.01), *p* = 0.30.	N/A.	Not a significant moderator.
Conscientiousness			276	4 (1.4%)	0 (0.0%)	2 (2.2%)	2 (2.2%)	0 ↑ 4 ↓	↓: self-report smoking behavior, attitudes, self-efficacy.	0.0029–0.0093	B (276,4,0.01), *p* = 0.30.	N/A.	Not a significant moderator.
Emotional stability			276	1 (0.4%)	0 (0.0%)	0 (0.0%)	1 (1.1%)	0 ↑ 1 ↓	↓: attitudes.	0.0082	B (276,1,0.01), *p* = 0.94.	N/A.	Not a significant moderator.
Clustering coefficients	Continuous. 0 to 10.	↑	276	4 (1.4%)	2 (2.2%)	1 (1.1%)	1 (1.1%)	3 ↑ 1 ↓	↑: experimental injunctive norms, donation to ASSIST/DC, self-report injunctive norms. ↓: self-report descriptive norms.	0.0028–0.0080	B (276,4,0.01), *p* = 0.30.	N/A.	Not a significant moderator.
Eigenvector centralities	Continuous. Base: 0.008 to 3.13. FU: 0.003 to 3.04.	↑	276	11 (4.0%)	5 (5.4%)	2 (2.2%)	4 (4.3%)	11 ↑ 0 ↓	↑: experimental injunctive anddescriptive norms, self-report injunctive norms, perceived risks, objectively measured smoking behavior.	0.0036–0.0096	B (276,11,0.01), *p* = 0.0001.	↑: B (276,11,0.01), *p* = 0.0001. ↓: B (276,0,0.01), *p* = 1.00.	Significant moderator. PI is stronger at higher EVC.
Closeness centralities	Continuous. Base: 2.24 to 5.10. FU: 2.15 to 4.94.	↑	276	13 (4.7%)	5 (5.4%)	4 (4.3%)	4 (4.3%)	12 ↑ 1 ↓	↑: self-report descriptive norms, self-report smoking behavior, intentions, self-efficacy, perceived risks, objectively measured smoking behavior. ↓: self-report injunctive norms.	0.0032–0.0313	B (276,13,0.01), *p* < 0.0001.	↑: B (276,12,0.01), *p* < 0.0001. ↓: B (276,1,0.01), *p* = 0.94.	Significant moderator. PI is stronger at higher CC.
Betweenness centralities	Continuous. Base: 0 to 2.83. FU: 0 to 5.49.	↑	276	3 (1.1%)	2 (2.2%)	0 (0.0%)	1 (1.1%)	3 ↑ 0 ↓	↑: self-report descriptive norms, objectively measured smoking behavior.	0.0071–0.0260	B (276,3,0.01), *p* = 0.52.	N/A.	Not a significant moderator.
Gini degree coefficients	Continuous. Base: 1.70 to 2.67. FU: 1.91 to 2.91.	↓	276	11 (4.0%)	4 (4.3%)	1 (1.1%)	6 (6.5%)	2 ↑ 9 ↓	↑: experimental donations to ASSIST/DC. ↓: experimental injunctive norms, self-report injunctive and descriptive norms, PBC, objectively measured smoking behavior.	0.0032–0.0145	B (276,11,0.01), *p* = 0.0001.	↑: B (276,2,0.01), *p* = 0.76. ↓: B (276,9,0.01), *p* = 0.002.	Significant moderator. PI is stronger in schools with lower Gini coefficients.
Self-efficacy (emotional, friends, opportunity subscales)	Continuous. 1 to 6.	↓	774	19 (2.5%)	7 (2.7%)	3 (1.2%)	9 (3.5%)	12 ↑ 7 ↓	↑: experimental injunctive norms, self-report descriptive norms, perceived risks, objectively measured smoking behavior. ↓: self-report injunctive norms, self-report smoking behavior, intentions, self-report injunctive norms.	0.0036–0.0090	B (774,19,0.01), *p* = 0.0004.	↑: B (774,12,0.01), *p* = 0.09. ↓: B (774,7,0.01), *p* = 0.65.	Some evidence that self-efficacy is a significant moderator. No indication whether PI is stronger at higher or lower self-efficacy.

^a^ASSIST: ‘A Stop Smoking in Schools Trial’, Base: baseline, CC: closeness centrality, DC: Dead Cool, EVC: eigenvector centrality, FNE: fear of negative evaluation, FU: follow-up, N/A: not applicable, NI: Northern Ireland, NTB: need to belong, PBC: perceived behavioral control, PI: peer influence, PNTS: prefer not to say, SES: socio-economic status. ^b^For continuous moderators, higher numerical values indicate higher values of the moderator (e.g., higher SES, rule-following, etc.). ^c^For dichotomous moderators, e.g., “Bogotá” indicates we hypothesized that peer influence effects would be stronger in Bogotá compared to NI. For continuous moderators, e.g., ↑ indicates we hypothesized that peer influence effects would be stronger at higher values of the moderator, ↓ indicates we hypothesized that peer influence effects would be stronger at lower values of the moderator. “Exploratory” indicates we conducted exploratory analyses to investigate moderation but made no hypotheses about the direction of the moderated effects. ^d^For dichotomous moderators, e.g., “10 NI” indicates that 10 of the significant interaction effects indicated there were stronger peer influence effects in NI. For continuous moderators, e.g., “6 ↑” indicates that 6 of the significant interaction effects indicated there were stronger peer influence effects at higher values of the moderator. ^e^ΔR^2^ indicates change in the R-squared value when including the interaction term in the model (i.e., the change in the percentage of outcome variance explained by the model). This quantifies how much additional variance in outcomes could be explained after accounting for the interaction terms. This column shows the range of ΔR^2^ for the models with significant interactions. ^f^Binomial tests examining whether the total number of significant interactions (p ≤ 0.01) observed for each moderator is greater than expected by chance. For example, the test denoted as B (276,20,0.01) evaluates whether observing 20 significant interactions out of 276 models is greater than expected under a binomial distribution with *p* = 0.01. ^g^Binomial tests considering the direction of the significant interactions (*p* ≤ 0.01). For example, the test denoted as NI: B (276,10,0.01) evaluates whether observing 10/276 significant interactions indicating stronger peer influence effects in NI is greater than expected under a binomial distribution with *p* = 0.01. N/A indicates the test was not carried out because the binomial test examining the total number of significant interactions was non-significant.

School SES was a significant moderator across all schools, with stronger peer influence effects in schools with lower SES. When we repeated the models examining peer influence from friends separately in NI and Bogotá, we found the peer influence effects were stronger in NI schools with lower SES. Whilst there was some evidence that school SES was a significant moderator in Bogotá, there was little indication of whether the peer influence effects were stronger at higher or lower school SES overall.

Overall, we found evidence that peer influence effects were stronger for pupils with higher levels of pro-sociality, FNE, and extraversion. There was evidence that peer influence effects were stronger for pupils with higher eigenvector and closeness centralities. There were also stronger peer influence effects in school networks with less heterogeneous degree distributions, as defined by the school network Gini degree coefficient (i.e., networks in which individuals had similar numbers of connections).

There was some evidence that self-efficacy was a significant moderator, but little indication of whether the peer influence effects were stronger at higher or lower levels of self-efficacy overall.

We found no overall evidence that pupils’ experimentally measured norm sensitivities, NTB, openness, agreeableness, conscientiousness, emotional stability, clustering coefficients, or betweenness centralities were significant moderators.

The significant moderating effects were distributed evenly across models of peer influence from friends, school classes, and year groups, and across models measuring peer influence at baseline versus follow-up.

**Figure 2:**
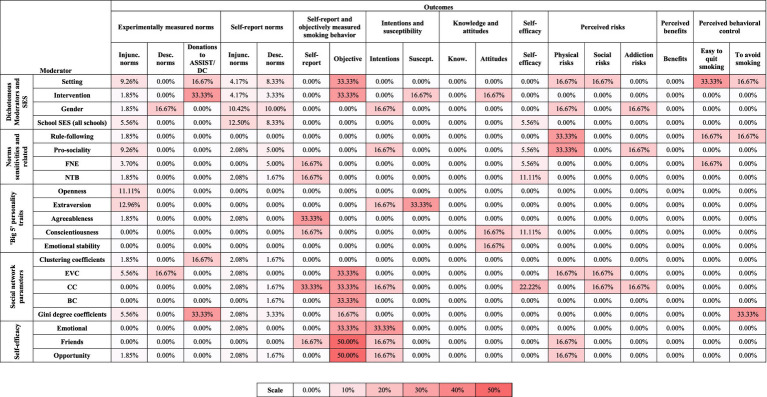
Heatmap showing a summary of the results for each moderator. Moderators are indicated along the lefthand side. Outcomes are indicated along the top. The percentages in each cell indicate the percentage of models with *p* ≤ 0.01 for the interaction effect. ASSIST: ‘A Stop Smoking in Schools Trial’, BC: betweenness centrality, CC: closeness centrality, DC: Dead Cool, Descript.: descriptive, EVC: eigenvector centrality, FNE: Fear of negative evaluation, Injunc.: injunctive, Know.: knowledge, NTB: need to belong, SES: socio-economic status, Suscept.: susceptibility.

Figure 2 shows a heatmap summarizing the results for each moderator according to smoking outcomes. The significant moderation effects appear dispersed across moderators and outcomes, with no single moderator consistently dominating. The strongest moderation signals were observed for setting, intervention, gender, pro-sociality, extraversion, network centrality parameters, Gini degree coefficients, and self-efficacy. These effects were most evident for models with objectively measured smoking behavior, intentions, and perceived physical risks as the outcomes. This suggests that the research context, gender, individual dispositions, and network structures all shape how peer influence impacts adolescent smoking.

### 3.2 Setting, intervention, and gender

The results for models with setting, intervention, and gender as moderators are reported in Figures 3–7, Supplementary File 2: Tables S2.1–S2.3, Supplementary File 3: Table S3.1, Supplementary File 4: Figures S4.1–S4.49, and Supplementary File 5: Figures S5.1–S5.12.

Setting was a statistically significant (*p* ≤ 0.01) moderator in 20/276 models. Three were for influence effects from friends, six were for school classes, and 11 were for school year groups. Ten models showed more positive peer influence effects in Bogotá and ten showed more positive peer influence effects in NI.

The mean standardized regression coefficient for the interaction effect across the 276 models with setting as the moderator was −0.003 (7.2% at *p* ≤ 0.01, 11.6% at *p* ≤ 0.05, Figures 4–7).

Intervention was a statistically significant moderator in 11/276 models. Four were for influence effects from friends, four were for school classes, and three were for school year groups. Seven models showed more positive peer influence effects in ASSIST schools and four showed more positive peer influence effects in Dead Cool schools. The mean standardized regression coefficient was 0.00008 (4.0% at *p* ≤ 0.01, 7.6% at *p* ≤ 0.05, Supplementary File 5: Figures S5.5–S5.8).

Gender was a statistically significant moderator in 18/276 models. Five were for influence effects from friends, six were for school classes, and seven were for school year groups. Twelve models showed more positive peer influence effects for girls/PNTS and six showed more positive peer influence effects for boys. The mean standardized regression coefficient was 0.02 (6.5% at *p* ≤ 0.01, 13.0% at *p* ≤ 0.05, Supplementary File 5: Figures S5.9–S5.12).

### 3.3 School socio-economic status

The results for models with school SES as the moderator are reported in Supplementary File 2: Tables S2.4–S2.6, Supplementary File 3: Table S3.2, Supplementary File 4: Figures S4.50–S4.76, and Supplementary File 5: Figures S5.13–S5.24.

School SES (all schools) was a statistically significant moderator in 15/276 models. Five were for influence effects from friends, six were for school classes, and four were for school year groups. Nine models showed decreasing peer influence effects as school SES increased. The mean standardized regression coefficient was −0.003 (5.4% at *p* ≤ 0.01, 8.3% at *p* ≤ 0.05).

In NI, School SES was a statistically significant moderator in 8/92 models examining peer influence effects from friends. Seven showed decreasing peer influence effects as school SES increased. The mean standardized regression coefficient was −0.02 (8.7% at *p* ≤ 0.01, 15.2% at *p* ≤ 0.05).

In Bogotá, School SES was a statistically significant moderator in 4/92 models examining peer influence effects from friends. One showed decreasing peer influence effects as school SES increased. The mean standardized regression coefficient was 0.02 (4.3% at *p* ≤ 0.01, 9.8% at *p* ≤ 0.05).

### 3.4 Norm sensitivities and related personality characteristics

The results for models with norm sensitivities and related personality characteristics as moderators are reported in Figure 8, Supplementary File 2: Tables S2.7–S2.10, Supplementary File 3: Table S3.3, Supplementary File 4: Figures S4.77–S4.109, and Supplementary File 5: Figures S5.25–S5.40.

Pro-sociality was a statistically significant moderator in 14/276 models. Five were for influence effects from friends, four were for school classes, and five were for school year groups. Eleven models showed increasing peer influence effects as pro-sociality increased. The mean standardized regression coefficient was 0.009 (5.1% at *p* ≤ 0.01, 12.7% at *p* ≤ 0.05).

FNE was a statistically significant moderator in 8/276 models. Four were for influence effects from friends, two were for school classes, and two were for school year groups. All eight models showed increasing peer influence effects as FNE increased. The mean standardized regression coefficient was 0.01 (2.9% at *p* ≤ 0.01, 16.3% at *p* ≤ 0.05).

We found little evidence that peer influence effects were significantly moderated by rule-following (individuals’ norm sensitivities) or NTB, which showed statistically significant interactions in only 5 and 6 out of 276 models, respectively.

### 3.5 ‘Big Five’ personality traits

The results for models with the ‘Big Five’ personality traits as moderators are reported in Supplementary File 2: Tables S2.11–S2.15, Supplementary File 3: Table S3.4, Supplementary File 4: Figures S4.110–S4.134, and Supplementary File 5: Figures S5.41–S5.60.

Extraversion was a statistically significant moderator in 10/276 models. Two were for influence effects from friends, four were for school classes, and four were for school year groups. Seven models showed increasing peer influence effects as extraversion increased. The mean standardized regression coefficient was −0.0001 (3.6% at *p* ≤ 0.01, 8.7% at *p* ≤ 0.05).

We found little evidence that peer influence effects were significantly moderated by the ‘Big Five’ personality traits of openness, agreeableness, conscientiousness, or emotional stability, which showed statistically significant interactions in only 6, 4, 4, and 1 out of 276 models, respectively.

### 3.6 Social network parameters

The results for models with social network parameters as moderators are reported in Supplementary File 2: Tables S2.16–S2.20, Supplementary File 3: Table S3.5, Supplementary File 4: Figures S4.135–S4.176, and Supplementary File 5: Figures S5.61–S5.80.

Individuals’ eigenvector centralities were statistically significant moderators in 11/276 models. Five were for influence effects from friends, two were for school classes, and four were for school year groups. All 11 models showed increasing peer influence effects as eigenvector centralities increased. The mean standardized regression coefficient was 0.009 (4.0% at *p* ≤ 0.01, 12.0% at *p* ≤ 0.05).

Individuals’ closeness centralities were statistically significant moderators in 13/276 models. Five were for influence effects from friends, four were for school classes, and four were for school year groups. Twelve models showed increasing peer influence effects as closeness centralities increased. The mean standardized regression was 0.02 (4.7% at *p* ≤ 0.01, 10.9% at *p* ≤ 0.05).

School network Gini degree coefficients were statistically significant moderators of peer influence effects in 11/276 models. Four were for influence effects from friends, one was for school classes, and six were for school year groups. Nine models showed decreasing peer influence effects as Gini degree coefficients increased. The mean standardized regression coefficient was −0.02 (4.0% at *p* ≤ 0.01, 7.6% at *p* ≤ 0.05).

We found little evidence that peer influence effects were significantly moderated by individuals’ clustering coefficients or betweenness centralities, which showed statistically significant interactions in only 4 and 3 out of 276 models, respectively.

### 3.7 Self-efficacy to resist smoking

The results for models with the self-efficacy subscales as moderators are reported in Supplementary File 2: Tables S2.21–S2.23, Supplementary File 3: Table S3.6, Supplementary File 4: Figures S4.177–S4.195, and Supplementary File 5: Figures S5.81–S5.92.

The self-efficacy subscales were statistically significant moderators in 19/774 models. Seven were for influence effects from friends, three were for school classes, and nine were for school year groups. Seven models showed increasing peer influence effects as self-efficacy decreased.

## 4 Discussion

This paper investigated moderators of peer influence effects (from nominated friends, school classes, and school year groups) for adolescent smoking and vaping outcomes in the MECHANISMS study (Hunter et al., 2020; Murray et al., 2023). Given that the MECHANISMS study was designed to compare results between NI (a high-income setting) and Bogotá (a middle-income setting), we were particularly interested in comparing peer influence processes between the settings. Furthermore, our novel experimental norms’ elicitation protocol proposes that peer influences within the same setting/context, and between settings/contexts should be moderated by individuals’ norm sensitivities (Hunter et al., 2020). In addition to setting, we examined moderation of peer influence effects by intervention program (ASSIST versus Dead Cool), gender, school SES, social norm sensitivities (experimentally measured) and related personality characteristics (self-reported), the ‘Big Five’ personality traits, social network parameters, and self-efficacy to resist smoking.

As a collectivistic culture historically vulnerable to the tobacco epidemic, we hypothesized that peer influence effects would be stronger among pupils in Bogotá compared to NI (hypothesis I) (Müller and Wehbe, 2008; Hofstede Insights, 2022). Overall, we found evidence the peer influence effects were significantly moderated by setting. However, the direction of the significant interactions varied, with stronger peer influence effects in NI for some outcomes and stronger effects in Bogotá for others. We found stronger peer influence effects in Bogotá for several experimentally measured and self-report injunctive norms outcomes, and pupils’ willingness to pay to support anti-smoking norms. In collectivistic societies, individuals tend to value social acceptance and conformity, identifying more strongly with normative referents (Liu et al., 2017). This helps explain why pupils in Bogotá were more strongly influenced by their peers when responding to outcomes designed to capture injunctive norms – collective perceptions about social appropriateness – or were more successful at gauging their peers’ responses in the experimental tasks. Supporting this interpretation, our earlier research found greater consensus in Bogotá that injunctive norms outcomes P2S2 (“a parent smoking in their own home in front of children under the age of 5″) and P2S5 (“in a recent superhero movie the lead actor is seen smoking in the opening scene”) were extremely socially inappropriate (Murray et al., 2020). We suggested this may reflect macro-level denormalization of indoor smoking and smoking advertisements following the 2009 implementation of the WHO-FCTC (Murray et al., 2020; Colombia Ombudsman Office, 2017; Otálvaro-Ramírez et al., 2019).

We also found some indication that perceived physical risks (e.g., getting a bad cough or lung cancer) were more strongly influenced by peers in NI, whereas perceived social risks (e.g., upsetting friends) were more strongly peer-influenced in Bogotá. In collectivistic contexts, there may be stronger social influence mechanisms inherent in how individuals evaluate potential social sanctions for violating socially driven behavioral norms like smoking (Eriksson et al., 2021). This aligns with literature conceptualizing sanctions as “metanorms,” or higher-level norms that establish how violations of lower-level norms should be punished (Axelrod, 1986). This pattern suggests that collectivistic orientations may heighten responsiveness to social sanctions but not necessarily to individually oriented health risks, where peer influence may be more salient in individualistic settings. Leveraging peer influence to highlight the social consequences of smoking may therefore be a particularly effective prevention strategy for adolescents in LMICs.

By contrast, peer influence effects were stronger in NI than Bogotá for several self-report descriptive norms outcomes asking how many peers smoke or how often they smoke. This suggests that pupils in Bogotá were more influenced by whether their peers thought smoking was socially acceptable (injunctive norms) but were less influenced in terms of whether they thought their peers actually smoked (descriptive norms). Despite the higher smoking prevalence (Northern Ireland Statistics and Research Agency, 2023; Ministry of Justice and Law – Colombian Drug Observatory, Ministry of National Education, 2022; Murray et al., 2020; Tate et al., 2021), pupils in Bogotá were less aware of their peers’ smoking behavior. For instance, when asked at follow-up “How many of your friends smoke?” (DN2.1), 47.7% of Bogotá pupils responded that they did not know, compared to only 20.4% in NI. This may indicate that adolescents in collectivistic cultures conform more strongly to normative peer influence, but only when aware of the descriptive norm. Alternatively, responses in Bogotá may have been affected by social desirability bias. Overall, collectivism seems to amplify peer influence primarily when norms are salient and consensual. When descriptive norms are less visible or more ambiguous, collectivistic tendencies do not necessarily translate into stronger peer effects.

Previous research shows that providing normative information, particularly on descriptive norms, can strongly influence behavior in collectivistic populations (Liu et al., 2017). Social norms interventions typically seek to correct common misperceptions that an unhealthy behavior is highly prevalent by providing accurate information on actual peer behavior (Ahmed et al., 2018). Our findings support using social norms-based intervention strategies that provide accurate information on descriptive norms in LMICs, since many adolescents may not have formed clear perceptions of peer smoking rates. However, prior studies caution that descriptive norm messages can sometimes backfire by inadvertently signaling that some people actually do engage in the undesirable behavior (Chung and Rimal, 2016; Cialdini, 2003). Since we have found stronger peer influence effects for injunctive norms in Bogotá but that pupils were less aware of the descriptive norms, social norms approaches that align descriptive norms with injunctive norms – providing accurate prevalence data while emphasizing that most peers disapprove of smoking and vaping – may be particularly effective in LMICs (Cialdini, 2003).

There was some indication that peer influence effects were stronger in ASSIST schools compared to Dead Cool, particularly for norms-related outcomes (willingness to pay to support anti-smoking norms, self-report injunctive norms, self-report descriptive norms), and smoking susceptibility. This finding accords with the theoretical underpinnings of the interventions, and hypothesis II. Importantly, this does not reflect overall program effectiveness, but rather that there are differences in the mechanisms through which the programs operate. ASSIST is based on the diffusion of innovations theory (Rogers, 2003), and is designed to leverage school friendship networks to propagate anti-smoking norms (Campbell et al., 2008). In contrast, Dead Cool follows a more traditional, teacher-led approach that directly targets pupils’ smoking intentions, knowledge and attitudes through classroom instruction, skills-building activities, and normative information dissemination (Hunter et al., 2020; Thurston et al., 2019). Our findings suggest that ASSIST primarily achieves change via peer influence and network-mediated processes, whereas Dead Cool influences outcomes through direct instruction. These differences have important implications for intervention design and implementation in LMICs. Peer-led programs like ASSIST may be particularly effective for shifting social norms and promoting behaviors that are sensitive to peer influence or in social contexts where the norms are particularly strong. However, teacher-led programs can still produce meaningful changes in knowledge, attitudes, and behaviors through direct instruction and skills-building (Murray et al., 2025b). Future research should explore how these mechanisms can be combined or adapted in different socio-cultural contexts. These mechanism-focused insights highlight that the translational value of interventions lies not only in overall effectiveness but in the pathways through which change occurs. Understanding these pathways allows policymakers and practitioners to design tailored interventions to maximize both reach and sustainability.

We found evidence that the peer influence effects were stronger for girls/PNTS compared to boys (hypothesis III). This is consistent with previous research suggesting that adolescent girls perceive more social pressures to smoke and are more susceptible to social influences (Mercken et al., 2010; Simons-Morton and Farhat, 2010; Tate et al., 2022; Grogan et al., 2009). Differences in friendship patterns between adolescent males and females may lead to differences in smoking-related peer influence processes. Females tend to have closer, more intimate friendships, and are more likely to turn to peers for support (Mercken et al., 2010; Thomas and Daubman, 2001). Females also may have a heightened sensitivity to social-evaluative concerns, which can lead them to rely on preserving close friendships as a source of self-evaluation and self-esteem (McCoy et al., 2017; Thomas and Daubman, 2001; Rudolph and Conley, 2005). This can lead to more opportunities for peer influence (Mercken et al., 2010). On the other hand, males may be more susceptible to deviant peer pressure for risk-taking behaviors, and some authors refer to gender role socialization theory as a possible theoretical explanation that sees male risk-takers as seeking alignment with masculine ideals (McCoy et al., 2017). In other words, girls may be more likely to smoke because they care about fitting in with their friends, while boys may be influenced by other kinds of peer pressures, like risk-taking or masculine ideals.

Adolescents tend to gravitate towards same-sex friends (Mercken et al., 2009; Mercken et al., 2010). This makes gender, and traditional gender role stereotypes that develop during adolescence, a potentially important source of group identity that can heighten perceptions of the potential consequences of deviating from masculine norms for males, or feminine norms for females (McCoy et al., 2017; Spears, 2021). Recent research has highlighted the role of group identity within the social influence process (Spears, 2021). The theory of normative social behavior proposes that the strength of group identity moderates the relationship between descriptive norms and behavior (Rimal and Real, 2005). Supporting empirical evidence from social norms intervention research shows that using more proximal referent groups, with which an individual may have a stronger social identification, strengthens the relation between perceived norms and behavior (Dempsey et al., 2018). For example, studies leveraging group identities by providing gender-specific social norms information have found stronger effects, compared to generic social norms information, for alcohol consumption (Lewis and Neighbors, 2015; Neighbors et al., 2010). Providing gender-tailored social norms information could be an effective technique to explore in future adolescent smoking prevention efforts.

Overall, there was evidence that the peer influence effects were stronger in schools with lower SES (hypothesis IV). When we repeated these analyses separately for NI and Bogotá, we found that the pattern was more prevalent in NI. This reflects findings in previous studies suggesting that the relationship between SES and smoking in LMICs may be more dynamic, and not necessarily conform to the historical pattern in higher-income countries (Harper and McKinnon, 2012; Rossouw, 2021; Chisha et al., 2019). Recent research suggests that with many countries like Colombia now adopting comprehensive tobacco control legislation, it is important to study how the relationships between social network structures and processes, SES, and adolescent smoking change over time (Littlecott et al., 2023; Littlecott et al., 2022). This is an important consideration for our study given the two contrasting research settings, with varying cultures, historical smoking rates and tobacco control contexts (Hunter et al., 2020; Murray et al., 2020; Murray et al., 2023). Countries which have introduced increasingly comprehensive tobacco control legislation over the past decade may have experienced widespread denormalization of smoking at the macro-systemic level whilst inequalities have remained. For example, the original ASSIST trial found higher levels of intervention effectiveness in schools with lower SES, higher smoking rates, and greater social network densities (Campbell et al., 2008; Littlecott et al., 2023; Littlecott et al., 2022). With reports showing high levels of implementation and acceptance since the introduction of the WHO-FCTC in 2009, arguably Colombia has experienced some level of denormalization of smoking (Murray et al., 2020; Ministerio de Salud y Protección Social, 2009; Colombia Ombudsman Office, 2017; Otálvaro-Ramírez et al., 2019). Social influence-based interventions like ASSIST may be particularly beneficial for schools in more deprived areas or in LMICs without tobacco control legislation where smoking remains largely normalized (Littlecott et al., 2023; Littlecott et al., 2022). We also ran our models investigating moderation of the peer influence effects according to the household SES of individual pupils but found no evidence that individuals’ SES was an important moderator (data not presented).

A wide body of literature has examined the relation between social identities and personality characteristics (Jenkins et al., 2012). Personality theorists conceptualize personality as a set of stable traits across the lifespan that can affect an individual’s behavior, group orientations, and how likely they are to follow norms (Jenkins et al., 2012; Weber et al., 2011). We found that peer influence effects were stronger amongst pupils with higher pro-sociality and FNE (hypothesis V). Pro-sociality has been defined as “voluntary behavior intended to benefit others” that includes a broad array of behaviors like altruism, helping, sharing, and cooperation (Van Hoorn et al., 2014; Padilla-Walker and Carlo, 2014). Individuals may engage in pro-social behaviors to avert negative affect (e.g., reducing the emotional impact of stress, self-conscious emotions, or averting feelings of guilt and shame) (Raposa et al., 2016; Chierchia et al., 2020; Somerville et al., 2013; Roos et al., 2014). FNE is a broad measure of social-evaluative anxiety that may include apprehension or distress about being negatively evaluated by others, avoiding evaluative situations, and having expectations that others would evaluate oneself negatively (Collins et al., 2005; Watson and Friend, 1969). Previous research investigating the affective dimensions of peer influence found that negative feelings were the main driving force behind conformity (Manzoni et al., 2011; Lashbrook, 2000). Conforming to peer expectations is often followed by positive reinforcement (e.g., admiration, or higher status), and adolescents may be more likely to conform due to the fear of being rejected, socially isolated, or ridiculed (Manzoni et al., 2011; Lashbrook, 2000). Adolescents are also more likely to fear social rejection and conform to peer pressures from proximal peers (e.g., close friends) (Paek and Gunther, 2007).

Contrary to our hypotheses, we found little evidence that peer influence effects were stronger for adolescents with higher ‘rule-following’ (our experimental measure of norm sensitivity) or NTB. The rule-following task has previously been validated as an empirical measure of general norm-following proclivity, which has been shown to correlate with willingness to follow more pro-social norms such as cooperation, reciprocity, and pro-social behavior (Kimbrough and Vostroknutov, 2016). One explanation for our null results could be that when it comes to peer influence for following adolescent smoking norms, rule-following may operate differently. For example, adolescent smoking can be viewed as an anti-social behavior that is often linked with anti-social deviance, rule-breaking and risk-taking (Weiss et al., 2019). In this context, pupils who have greater norm sensitivities may face a trade-off because they want to conform to the peer norms in their social group but also want to avoid engaging in an anti-social behavior like smoking. This suggests that the role of norm sensitivity in smoking-related peer influence amongst adolescents may be more nuanced and bi-directional.

We also found little evidence to support that peer influence effects were moderated by the ‘Big Five’ personality traits, apart from extraversion for our sample (hypothesis VI). This could suggest that broad dispositional traits are less relevant for predicting adolescents’ susceptibility to peer influence for smoking compared to traits with stronger social-evaluative aspects (e.g., pro-sociality and FNE). Adolescence is a period of development that is marked by heightened sensitivity to peer evaluation and social comparison (Lashbrook, 2000; Paek and Gunther, 2007). This developmental context may amplify the role of socially oriented characteristics and diminish the moderating role of more general personality traits which may still be developing and changing during adolescence (Litt et al., 2015; Borghuis et al., 2017; Tetzner et al., 2023). Among the ‘Big Five’ traits, extraversion could be the exception due to its inherent social orientation. For example, extraversion represents individual differences in sociability, social ascendancy, and the propensity to express positive emotions (Morizot, 2014; Ortet et al., 2017; Denissen and Penke, 2008). Previous studies have reported inconsistent findings for the moderating effects of the ‘Big Five’ personality traits on peer influence (Slagt et al., 2015; van Schoor et al., 2008; de Leeuw et al., 2010; Yu et al., 2013; Gallego et al., 2018; Theakston et al., 2004; Pocuca et al., 2018; Poelen et al., 2007). Slaght et al. and van Schoor et al. found that peer influence effects were significantly moderated by only one of the ‘Big Five’ traits for adolescent delinquent behaviors and young adults’ alcohol consumption, respectively (Slagt et al., 2015; van Schoor et al., 2008). Whilst de Leeuw et al., found some evidence that influences from parents and siblings for adolescent smoking were moderated by the ‘Big Five’ traits, they found no evidence for moderation of the effects of friends’ smoking (de Leeuw et al., 2010). In practical terms, these findings imply that smoking prevention interventions may benefit more from targeting adolescents’ social identity processes and sensitivity to peer evaluation, rather than tailoring approaches to broad personality profiles. They also provide further support for emphasizing the social consequences of smoking.

Our study provides further supporting evidence that social network structures affect how social influence operates, and how social norms spread (hypothesis VII) (Hunter et al., 2020; Lansford et al., 2009; Haynie, 2015; Panter-Brick et al., 2006; Robalino and Macy, 2018). Interventionists may wish to consider social network structures and properties when deciding on the most appropriate smoking prevention strategies to adopt for specific populations. Previous research has found that the most “popular” adolescents within school social networks (i.e., those with more highly inter-connected friends as defined by their eigenvector centralities), exerted the most peer influence for smoking (Robalino and Macy, 2018). For our sample, we found that peer influence effects were greater for pupils with higher eigenvector centralities, higher closeness centralities, and in school networks with less heterogeneous degree distributions as defined by the school network Gini degree coefficient (i.e., networks in which individuals had similar numbers of connections) (Gini, 1912; Dalton, 1920). We found no evidence that peer influence effects were moderated by pupils’ clustering coefficients or betweenness centralities. Pupils who are more central are not only the most influential within social networks, but also the most susceptible to peer influences for smoking. Smoking prevention programs based on peer education and diffusion of anti-smoking norms (e.g., the ASSIST program, which relies on trained ‘peer supporters’ having informal conversations about smoking with friends) may consider targeting the most central pupils within social networks – defined by eigenvector or closeness centralities – as peer leaders since they are more influential and are also likely to benefit from receiving tobacco education as their social network positions make them more susceptible to peer influences for smoking. Reviews of social network-based interventions have identified four broad categories of intervention approaches (Hunter et al., 2019; Valente, 2012). The ASSIST intervention, which identifies individuals to act as proponents of behavior change, falls under the ‘individuals’ category for which there is the strongest evidence of effectiveness in previous intervention studies (Hunter et al., 2019). Our results showed that peer influence effects were stronger for more homogeneous school networks in which pupils had similar numbers of connections. Future intervention studies may wish to consider whether some of the other network intervention approaches may be effective targets for adolescent smoking prevention in schools with more heterogeneous friendship networks (e.g., the ‘segmentation’ approach, which targets groups of individuals clustered within networks) (Hunter et al., 2019; Valente, 2012).

Self-efficacy, defined as confidence in one’s ability to perform a desired behavior or to resist an undesirable behavior (Bandura, 1977), has been widely researched as a predictor of various health behaviors, and often conceptualized as a mediator illustrating how a change in behavior takes place following an intervention (Hunter et al., 2020; Elshatarat et al., 2016). However, several authors have disputed the causal role of self-efficacy in health behavior change (French, 2013), or called for researchers to explore various complex models when incorporating self-efficacy into behavior change research (Schwarzer, 2015). Some authors have conceptualized self-efficacy as a more stable behavior-specific personality trait that may act as a moderator (Stacy et al., 1992; Schwarzer, 2015), and early research on moderators of peer influences in adolescent smoking has found that having higher levels of self-efficacy can act as a buffer that protects adolescents against social influence (Stacy et al., 1992). Overall, we found evidence that self-efficacy acted as a moderator of peer influence effects in our sample, but little indication of whether peer influence effects were stronger at higher or lower levels of self-efficacy. However, there was little variability in our study’s self-efficacy outcomes. Most pupils had high levels of self-efficacy at baseline with 82.0, 83.1, and 88.5% scoring values ≥5 for the emotional, friends, and opportunity subscales, respectively.

### 4.1 Strengths and limitations

Study strengths include the large sample size, and comparison of results between two settings (one high-income, one middle-income) that have different norms, cultural traits, regulatory contexts, network structures, and health behavior patterns. This is important given the lack of relevant research and high smoking rates in LMICs (World Health Organization, 2025; Thomas et al., 2015; Munabi-Babigumira et al., 2012; Huriah and Dwi, 2020). All study materials underwent a thorough cultural adaptation process at the start of the study in Bogotá (Sánchez-Franco et al., 2021). Our study includes a broad range of smoking and vaping-related outcomes to provide richer insights into the working mechanisms of the interventions. These include self-report and objective measures of smoking behavior, and smoking/vaping norms assessed by self-report and experimental methods. This is the first study to apply experimental methods from behavioral economics, which mitigate social desirability biases, to study norms for adolescent smoking and vaping (Hunter et al., 2020; Murray et al., 2020). We have also examined moderation of peer influence effects by a broad range of potential moderating variables.

Limitations include the relatively small sample of schools, exclusion of nominated friends with missing attribute data, and multiple testing. In each setting, we endeavored to recruit schools with a range of deprivation levels and mixed-gender. There was a high participation rate across the schools, and rates of completion for the experiments and survey were high at both time-points. Therefore, the impact of missing data should be minimal. We have accounted for multiple testing by discussing our results with reference to a significance criterion of *p* ≤ 0.01, highlighting which results would have attained statistical significance (*p* ≤ 0.05) after using the Holm-Bonferroni procedure to correct the *p*-values for multiple testing (Holm, 1979), and using multiverse-style analysis strategies (Steegen et al., 2016; Del and Gangestad, 2021). The issue of making adjustments for multiple testing within a study is a widely debated issue. Whilst there are no established rules or guidance, several prominent academics have made a strong case for why it is not always desirable, or even correct, to adjust for multiple testing (Feise, 2002; Perneger, 1998; Rothman, 1990). Whilst adjustments for multiple testing reduce type one error rates (the rate of falsely finding a significant result), they simultaneously increase type two error rates (the rate of falsely concluding a null result), which increases the likelihood of missing important findings. Given that our analyses aimed to test the theoretically justifiable hypotheses outlined in the introduction section, we adopted the approach of discussing all results meeting the *p* ≤ 0.01 criterion. This approach permitted us to discuss all of the potential implications for future norms-based intervention research, whilst highlighting which results would not have attained statistical significance using the stricter adjustment criterion. VIFs were high for several models with setting as a moderator. However, we have followed standard recommendations for conducting moderation analyses – examining interaction effects (Hayes, 2013) – and have indicated which models had high VIFs. Only one model with a significant interaction showed potentially problematic amounts of collinearity.

### 4.2 Implications for future research

Our results highlight important opportunities for refining future social norms and social network-based interventions, particularly for adolescent smoking prevention in LMICs. Social norms intervention approaches that align descriptive norms – providing accurate information on how many peers smoke – with injunctive norms – emphasizing that most peers disapprove of smoking and vaping – may be especially effective in LMICs (Cialdini, 2003). There may also be stronger social influence mechanisms in how individuals evaluate potential social sanctions for norm violations in LMICs (‘metanorms’) (Eriksson et al., 2021; Axelrod, 1986). Leveraging peer influences to highlight the social consequences of smoking could be an effective strategy in LMICs. Providing gender-tailored norms information should also be explored in future prevention efforts (Lewis and Neighbors, 2015; Neighbors et al., 2010). Our findings highlight that peer-led programs like ASSIST and teacher-led programs like Dead Cool operate through distinct mechanisms (network diffusion versus direct instruction). Recognizing these pathways can inform the design of tailored interventions that combine or adapt elements of each approach to maximize impact and sustainability in different settings. Social influence-based interventions like ASSIST may be particularly beneficial in more deprived areas or in LMICs lacking strong tobacco control legislation, where smoking remains largely normalized (Littlecott et al., 2023; Littlecott et al., 2022). Finally, our results underscore the importance of adolescents’ social identity processes, sensitivity to peer evaluation, and the structural properties of school networks in shaping peer influence. Selecting peer leaders based on eigenvector or closeness centralities may enhance diffusion effects, while alternative network intervention strategies like ‘segmentation’ may be beneficial for more heterogenous networks (Hunter et al., 2020; Lansford et al., 2009; Haynie, 2015; Panter-Brick et al., 2006; Robalino and Macy, 2018; Hunter et al., 2019).

## 5 Conclusion

This paper investigated moderators of peer influence effects around two different types of adolescent smoking prevention programs for participants in the MECHANISMS study (Hunter et al., 2020; Murray et al., 2023). Our results show how contextual factors (e.g., high-income versus middle-income settings), gender, intervention type, school SES, personality characteristics, social network structures, and individuals’ positions within social networks, can affect how susceptible individuals and groups are to peer influences for smoking/vaping. We also found that peer influence effects were stronger in ASSIST than in Dead Cool schools, reflecting the programs’ distinct mechanisms. ASSIST operates primarily through network diffusion whilst Dead Cool operates through direct instruction and skills-building. Recognizing these pathways can guide the design of interventions that combine peer-led diffusion and teacher-led instruction as complementary strategies to maximize impact in different settings. Future research on social norms and network-based interventions for adolescent smoking prevention in LMICs should incorporate both injunctive and descriptive norms approaches, highlighting the social consequences of smoking. Social influence-based intervention strategies may be particularly beneficial for schools with lower SES or in LMICs without tobacco control legislation where smoking remains largely normalized at the macro-level. Future directions for improving and refining norms and networks-based smoking-prevention intervention research include providing gender-tailored social norms information, paying greater attention to adolescents’ socially oriented personality traits that heighten sensitivity to peer evaluation, considering whether individuals with high eigenvector or closeness centralities might be better targets in peer-led programs, and exploring alternative network intervention approaches for more heterogeneous networks (e.g., ‘segmentation’, which involves targeting clusters of individuals within social networks). Overall, future research should focus on tailoring interventions to both socio-cultural and network contexts to strengthen their effectiveness and sustainability in high-income and LMIC settings.

## Data Availability

The datasets presented in this article are not readily available because participants were informed that no-one outside of the research team would have access to the research data when they signed their consent forms. For further information about the study datasets and analytic code, please contact the corresponding authors (Emails: jmurray39@qub.ac.uk; ruth.hunter@qub.ac.uk). Requests to access the datasets should be directed to Jennifer Murray, jmurray39@qub.ac.uk; Ruth Hunter, ruth.hunter@qub.ac.uk.

## Supplementary material

SUPPLEMENTARY File 1 Supplementary methods, social network parameter definitions, study flow diagram, participants’ baseline characteristics, and breakdown of outcome variables.

SUPPLEMENTARY File 2 Regression coefficients for predictors, moderators, and interactions.

SUPPLEMENTARY FILE 3 Marginal effects, regions of significance, and changes in R-squared values for models with significant interactions.

SUPPLEMENTARY FILE 4 Figures showing conditional effects of peer influence for smoking and vaping outcomes by varying levels of the moderators with bounds indicating regions of significance.

SUPPLEMENTARY FILE 5 Multiverse figures.
