# Supplementary material for: Moderators of peer influence effects for adolescents’ smoking and vaping norms and outcomes in high and middle-income settings
Source: Front Psychol. 2025 Nov 3;16:1655761. doi: 10.3389/fpsyg.2025.1655761 (PMC12620383; doi:10.3389/fpsyg.2025.1655761)
Supplement: SUPPLEMENTARY File 1 — Supplementary methods, social network parameter definitions, study flow diagram, participants’ baseline characteristics, and breakdown of outcome variables. [file Table_1.docx]

Supplementary File 1

Moderators of peer influence effects for adolescents’ smoking and vaping norms and outcomes in high and middle-income settings.

**Jennifer M. Murray*, Sharon C. Sánchez-Franco, Olga L. Sarmiento, Erik O. Kimbrough, Christopher Tate, Shannon C. Montgomery, Rajnish Kumar, Laura Dunne, Abhijit Ramalingam, Erin L. Krupka, Felipe Montes, Huiyu Zhou, Laurence Moore, Linda Bauld, Blanca Llorente, Frank Kee, Ruth F. Hunter***

*** Correspondence:** Corresponding Authors: [jmurray39@qub.ac.uk](mailto:jmurray39@qub.ac.uk), [ruth.hunter@qub.ac.uk](mailto:ruth.hunter@qub.ac.uk)

**This file includes:**

Supplementary Methods.

Social network parameter definitions.

Supplementary Figure S1.1. (study flow diagram).

Supplementary Table S1.1. (participants’ baseline characteristics).

Supplementary Table S1.2. (breakdown of outcome variables).

The information in the Supplementary Methods has been previously published as supplementary information to earlier papers related to the MECHANISMS study (1–3).

**
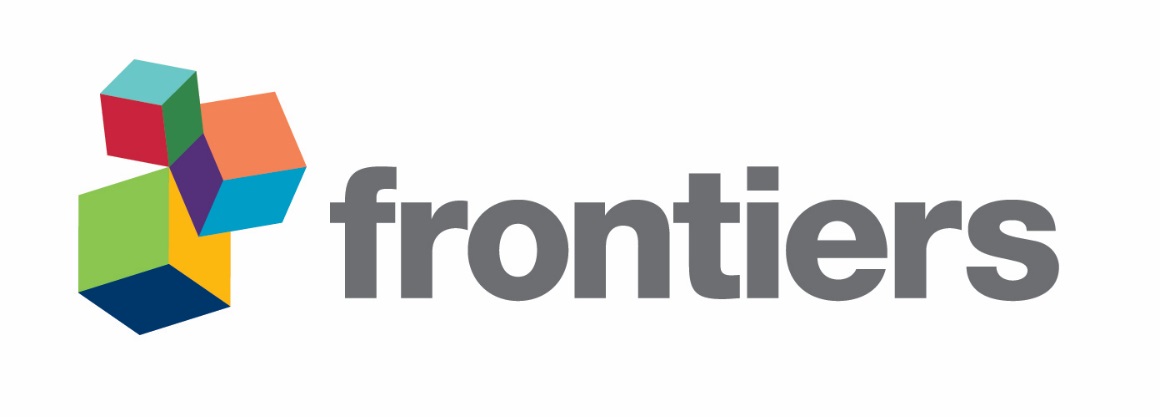
**

**Supplementary file 1: Supplementary methods, social network parameter definitions, study flow diagram, participants’ baseline characteristics, and breakdown of outcome variables.**

**Supplementary methods**

**Study procedures**

**Sample size calculation:** The study's power calculation was drawn from the work of Krupka et al., (2016) who studied ~200 Michigan university freshmen before and after a single semester (4). Using the basic Clauset community detection algorithm (5), with a sample size of ~200, and with data from two waves, a one standard deviation (SD) increase in risk preferences of an individual’s friends or social network was associated with an increase of 1/8th to 1/10th of a standard deviation (SD) of the same variable for the individual. Assuming that clustering of risk preferences is a reasonable proxy for clustering of social norms related to smoking that we have elicited in the MECHANISMS study, the power calculation estimated that a sample size of 300 would give over 80% power to detect, as statistically significant at the 5% level, a slope of 0.16 (i.e., an increase of 0.16 SDs in “y” [individual norm sensitivity], per SD increase in “x” [norm sensitivity of those in the individual’s social network]).

**School recruitment and assignment to intervention programs:** In Northern Ireland (NI), schools were recruited for the full phase of the MECHANISMS study between November 2018 and January 2019. Schools were recruited for the "A Stop Smoking in Schools Trial" (ASSIST) program as follows. Non-selective post-primary schools were selected from the list of secondary schools in NI as outlined by the Department of Education NI (6). Schools were prioritized if they were non-selective secondary education schools, mixed sex, had over 100 pupils in the year group, were of higher deprivation levels, and ranged in geographical location (urban, rural) and sector (controlled, maintained, integrated). Schools with over 100 pupils in Year 9 were identified using Year 8 enrolments during 2017 (7). Deprivation was measured using the Northern Ireland Multiple Deprivation Measure 2017 (NIMDM2017) (8). Invitations outlining the study procedures were sent to the Principals of nine schools who met the criteria outlined above. Follow-up emails and phone-calls were conducted by a member of the study team, and a total of three schools were recruited to the ASSIST arm.

Schools were recruited for the Dead Cool program in NI as follows. Schools were prioritized if they were in more deprived areas and had higher school enrolment numbers. The Cancer Focus NI (CFNI) Post Primary School database was searched and schools were eliminated if they were currently enrolled in the Dead Cool program or other CFNI smoking prevention programs. The remaining schools were contacted for expression of interest. After initial contact with the school was established, schools were eliminated if they had a class duration of less than 50 minutes (as this was deemed unsuitable for satisfactory completion of the Dead Cool program). The remaining schools were classified to ensure a good mix in terms of sex, geographical location (urban, rural) and sector (controlled, maintained, integrated). Detailed information regarding study procedures was sent to each school, resulting in a number of schools deselecting due to a range of issues (e.g., change of staff due to illness, unannounced school inspection, commitment to other initiatives). A total of three schools were enrolled to the Dead Cool arm of the study. The remaining schools were offered the Dead Cool program without taking part in the study.

In Bogotá, schools were recruited for the full phase of the MECHANISMS study between March and May 2019. The school recruitment strategy was as follows. First, a list of 40 private and public schools were prioritized based on health risks outlined by the Education and Health secretaries. Second, from this list, 13 schools were invited to participate according to the following inclusion criteria: (1) schools in urban area; (2) including boys and girls; (3) having enrolled between 90 and 150 students in 7th year (equivalent of Year 9 in NI). Third, only six schools accepted the invitation and were selected for the final sample. These schools were assigned randomly to each of the intervention arms by a member of the study team. Three schools were assigned to the ASSIST program, and the other three were assigned to the Dead Cool program.

**Ethical approval and study procedures:** Prior to the baseline assessment, each school was provided with Teacher information sheets, Pupil information sheets, Parent/guardian information sheets, Pupil consent forms, and Parent/guardian opt-out forms. All pupils were required to complete written consent forms indicating whether they agreed or declined to participate. Parents/guardians who did not wish their child to take part were asked to return completed opt-out forms. Pupils who consented to participate took part in a baseline assessment consisting of a series of game theory experiments and completion of a self-report survey. The fieldwork was conducted in NI schools between January and June 2019, and in Bogotá's schools between July and November 2019. Following the baseline assessment, each school took part in one of two previously tested smoking prevention interventions: ASSIST (N=3 schools in NI, N=3 Bogotá) or Dead Cool (N=3 NI, N=3 Bogotá) over a single school semester (approximately 10 weeks). Following intervention delivery in each school, all participants took part in a follow-up assessment, again completing the game theory experiments and a self-report survey. Prior to implementation in Bogotá, all study materials (experiments, surveys, intervention materials) underwent a 'cultural adaptation' process, including translation into Spanish language and back translation, using a previously published framework (9,10). Participation in the study's experiments required a monetary payment to be made to each individual pupil. In NI the payment was made in cash, however due to Colombian ethical regulations the payment was made using gift cards to pupils in Bogotá. Ethics approval was granted from the School of Medicine, Dentistry and Biomedical Sciences Ethics Committee at Queen's University Belfast (QUB) on September 21, 2018 (reference 18:43) and from the Research Ethics Committee at Universidad de los Andes (UniAndes) on July 30, 2018 (reference 937/2018). This study complies with all relevant ethical regulations. All study procedures, including the informed consent process, were conducted in accordance with the ethical standards of the responsible committee on human experimentation (institutional and national) and with the Helsinki Declaration of 1975, as revised in 2000.

**Data collection:** The baseline assessment consisted of two separate sessions, held approximately one week apart, with each class in the school year group in each school. Sessions lasted approximately 50 minutes. Participating pupils completed the game theory experiments during the first session and the self-report survey during the second session. Experiments and surveys were collected on tablet computers using the platform Qualtrics (web-based platform in NI and offline version in Bogotá) (Qualtrics, Provo, Utah, USA). At the start of each session, participants were assured that any information provided would be treated as confidential. They were also instructed not to communicate with other participants and to direct any questions to a researcher. In NI, poster boards were used at computer stations to discourage communication between participants. In both countries, instructions were delivered onscreen with key portions read aloud by the experimenter. The experimenter read out introductory instructions at the start of the experiment, and at the start of Parts 1, 2 and 4. Pupils were invited to ask any questions. Dummy screens were inserted at the end of Parts 1 and 3 instructing pupils to wait until all of their classmates were ready to proceed to the next part so that instructions could be read together. Parts 2 and 3 were otherwise self-paced, and pupils were invited to raise their hand to have any further questions answered privately. The same procedures were used during the follow-up assessment.

**Interventions:** The ASSIST and Dead Cool programs have previously been evaluated in separate cluster randomized trials, and shown to effectively reduce rates of adolescent smoking initiation (11,12). ASSIST is specifically designed to leverage peer influence whilst Dead Cool is based on conventional classroom pedagogy. The ASSIST program is based on the diffusion of innovations theory (13), and works on principles of peer education and diffusion. It is designed to train the most influential pupils in the school year group, nominated in a Peer Questionnaire completed by all participants prior to the baseline assessment, to use informal contacts with their peers (i.e., other pupils in their school year group) to encourage them not to smoke (11). The top 18% nominated pupils are invited to attend a two-day training course away from school premises to increase their knowledge of the effects of smoking and enhance their skills to enable them to take on the role of 'peer supporter'. Over the next ten weeks, they are asked to have informal conversations with their school friends about smoking, and take part in four follow-up sessions during which they receive on-going support from the ASSIST trainers (11). Dead Cool is a skills-based program based on the theory of planned behavior (14). It includes training of school teachers in program delivery and provision of program resources (lesson plans, pupil work books, fact sheets and a DVD) to enhance pupils' knowledge of potential influences on smoking behavior from family, friends and the media (12,15). Pupils receive eight class sessions during which they watch DVD clips of adolescents discussing smoking-related issues, and complete various workbook and group activities. At the end of the program, the pupils are asked to make group presentations about their new skills (12,15).

**Statistical analysis.**

Analyses were conducted using Stata 13 (StataCorp) (16). Descriptive statistics, and Cronbach's alpha coefficients for individual scales were calculated (Table 1).

Peer influence effects were examined for the following smoking and vaping outcomes which were targeted by the ASSIST and Dead Cool interventions (17): experimentally measured injunctive norms (P2S2-9), experimentally measured descriptive norms (P3Q1-2), experimental donations to ASSIST/Dead Cool, self-report injunctive norms (IN1-7), self-report descriptive norms (DN1.1-1.5, DN2.1-2.3), self-report smoking behavior, intentions, knowledge, attitudes, self-efficacy (emotional, friends, and opportunity subscales), perceived risks (physical, social, and addiction subscales), perceived benefits, PBC (easy to quit smoking; to avoid smoking), objectively measured smoking behavior (exhaled carbon monoxide), and smoking susceptibility (0=not susceptible, 1=susceptible).

To examine influence effects from friendship networks, school classes, and school year groups, variables were computed for each outcome at baseline and follow-up, containing: (1) the average responses of the focal participant's (*i*) friendship network; (2) the average responses of *i*'s school class, excluding *i*; and (3) the average responses of *i*'s school year group, excluding *i* (4). Ordinary least square (OLS) regressions with robust (Huber-White) standard errors (18,19) were used to examine influence effects, and moderation of influence effects, for focal participant outcomes at follow-up. In addition to the 'average peer' predictor variable, the moderator variable and its interaction with the predictor variable were included in each model. We considered a model to show a significant moderating effect if the interaction term reached statistical significance at p≤0.01.

Logistic regressions were run with focal participants' smoking susceptibility at follow-up as the outcome, and robust (Huber White) standard errors (18,19). Variables were computed at baseline and follow-up containing: (1) the percentage of *i*'s friendship network that were susceptible to commencing smoking; (2) the percentage of *i*'s school class that were susceptible to commencing smoking, excluding *i*; and (3) the percentage of *i*'s school year group that were susceptible to commencing smoking, excluding *i*. In addition to the 'percentage peer' predictor variable, the moderator and its interaction with the predictor were included. Baseline covariates adjusted for in all models included: gender, age, intervention, ethnicity, individuals' SES, and *i*'s baseline values of the outcome (2). Continuous predictor variables, moderator variables, and baseline outcomes were mean-centered.

The models were repeated to examine influence effects from average peer responses at baseline and average peer responses at follow-up (2). Apart from the social network parameters, all moderator variables were measured at baseline. Social network parameters were calculated at baseline and follow-up. For models examining influence effects from the baseline responses of peers, *i*’s baseline value of the social network parameter was examined as the moderator. For models examining influence effects from the follow-up responses of peers, *i*’s follow-up value of the parameter was examined as the moderator. We aimed to examine moderation of influence effects from average peer responses according to the focal participant's position within the network (or the network structure) at the time when the peer responses were measured. Furthermore, although we do not expect to see changes in network structures as a result of the smoking prevention interventions, we previously found that there were significant changes in friendship nominations (i.e., network structures) for 11 of the 12 schools between baseline and follow-up (2).

Unstandardized regression coefficients (*b*) were extracted. Odds ratios (ORs) and ratios of ratios were extracted for logistic regression models. Changes in the model R-squared (the proportion of variance in the outcome that is explained by the model (20)) before and after adding the interaction effects were calculated (∆R^2^). Variance inflation factors (VIFs) were calculated to examine multi-collinearity (>5 or 10 usually indicates problematic amounts of collinearity). VIFs for 'setting' were high for many of the models with school class or year group responses as predictors (2). Where high VIFs affected one of the models with setting as a moderator, this is indicated in the footnotes of Tables S2.1. and S3.1. in Supplementary files 2 and 3. VIFs were satisfactory for all other analyses.

Significant interactions (p≤0.01) were probed using the simple slopes and Johnson-Neyman techniques (21,22). For dichotomous moderator variables, the simple slopes technique was used to calculate marginal effects at each level of the moderator (NI versus Bogotá, ASSIST versus Dead Cool, and boys versus girls/PNTS). Marginal effects were calculated as unstandardized regression coefficients representing the average change in the outcome variable (focal participant outcomes at follow-up) for a one-unit increase in the predictor variable (average 'peer' variable) among participants who were at the specific level of the moderator variable. For the logistic regression models, marginal effects were the average change in the predicted probability of being classified as susceptible to commencing smoking for a 10% increase in the number of peers classified as susceptible (predictor variable) among participants who are at the specific level of the moderator. Conditional effects were graphed with their 95% confidence intervals (CIs), showing the relationship between the predictor and the predicted value of the outcome (or predicted probability of being classified as susceptible to commencing smoking) at follow-up, at each level of the moderator. Regions of significance were calculated, using the Johnson-Neyman technique (22), as values of the predictor for which the conditional effects significantly differed at different levels of the moderator at the 95% and 99% confidence levels.

For continuous moderator variables, the simple slopes technique was used to calculate marginal effects at one standard deviation below ('low') and above ('high') the mean value of the moderator. For models examining betweenness centralities as moderators, 'low' values were capped at the minimum value. For models examining self-efficacy as moderators, 'high' values were capped at the maximum value of the scale. Graphs were constructed showing the conditional effect of the predictor variable (average 'peer' variable) on the outcome variable (focal participant outcomes at follow-up), with 95% CIs, as a function of the moderator variable. For the logistic regression models, graphs were constructed showing the conditional effect of the predictor (percentage of peers classified as susceptible to commencing smoking) on the probability of being classified as susceptible at follow-up, with 95% CIs, as a function of the moderator. The Johnson-Neyman technique was used to calculate regions of significance in terms of values of the moderator for which the conditional effects differed significantly from 0 at the 95% and 99% confidence levels (22).

Due to the large number of tests for each moderator, we adopted a fourfold approach to adjusting for multiple testing: firstly, we have discussed our results with reference to a significance criterion of p≤0.01; secondly, in the results tables we have highlighted which results would have attained statistical significance (p≤0.05) after using the Holm-Bonferroni procedure to adjust the p-values for multiple testing (23); thirdly, we used binomial tests (B) to determine whether the number of significant interactions (p≤0.01) observed per moderator was greater than expected by chance; and fourthly, we have used multiverse-style analyses to summarize the distribution of p-values and standardized regression coefficients for interaction effects across the models for each moderator (24,25).

For the multiverse-style analyses, standardized regression coefficients for interaction effects were extracted for comparability across outcomes measured on different scales (representing the estimated difference in standard deviations of the outcome variable between two cases that differ by one standard deviation on the ‘peer group average’ predictor variable, for a one-unit increase on a dichotomous moderator, or an increase of one standard deviation on a continuous moderator). Histograms were constructed showing the distribution of p-values for interaction effects for each moderator. Volcano plots were constructed showing the relationship between the negative logs of p-values and standardized regression coefficients for interaction effects, and figures were constructed to visualize the distribution of significant interaction effects (p≤0.01 and p≤0.05) according to outcome (experimental norms outcomes, self-report norms outcomes, or other self-report smoking outcomes and objectively measured smoking behavior), peer group (friends, school class, or school year group), and measurement time-point for predictor variables (baseline or follow-up) (24,25). Most of the volcano plots showed a Janus effect (with standardized regression coefficients at the first and 99^th^ percentiles having opposite signs) (25). However, the plots depict associations between standardized regression coefficients and p-values for models with different outcomes, ‘peer group’ predictor variables, and measurement time-points for predictor variables (i.e., the differences between the observations are not arbitrary, as is usual with multiverse analyses) (24,25). Finally, we constructed summary tables and heatmaps to facilitate the narrative synthesis of results for each moderator.

**Game Theory Experiments**

The game theory experiments consisted of a series of incentivized tasks which were based on published works in the field of behavioral economics (26–28). There were four parts to the experiment: (1) a Rule-Following (RF) task measuring each individual participant's sensitivity to the effects of social norms; (2) a series of co-ordination games attempting to elicit *injunctive* social norms unrelated and related to smoking and vaping behaviors; (3) a series of co-ordination games attempting to elicit *descriptive* social norms related to smoking and vaping behaviors; (4) a willingness-to-pay task designed to measure each individual participant's support for cultivating anti-smoking norms. These are outlined in more detail below. The full experimental protocol is available below.

At the start of experimental sessions, participants were informed that they would receive a participation fee of £5.00 (NI; *COP* $5.000 in Bogotá), and that they could earn money in each part of the experiment (maximum £30 in NI, *COP* $50.000 in Bogotá) depending on the answers they provided and those provided by other pupils in their school year group. They were told that the researchers would determine their payment by performing two sets of randomizations for each part of the experiment: (1) to determine whether payment was based on answers provided at baseline or follow-up; (2) to determine which question of each part would result in payment.

**Part 1: Identifying individual-level norm sensitivity**

Part 1 of the experiment consisted of an individual decision task (a variant of the RF Task) measuring participants' preferences for following established rules and social norms, without peer interaction (26,27). The task instructs participants to follow an explicitly stated arbitrary rule when doing so imposes explicit monetary costs directly proportional to the degree of rule-following. We employed the version of the RF task introduced by Kimbrough & Vostroknutov (2018) (27). Specifically, participants were asked to sequentially allocate 50 balls across two buckets (one blue and one yellow). They were told that "The rule is to put the balls in the blue bucket". They were also informed that they would receive £0.05 (NI; *COP* $100 Bogotá) for every ball they put in the blue bucket and £0.10 (NI; *COP* $200 Bogotá) for every ball they put in the yellow bucket. Lastly, they were informed that they would be given five minutes to allocate the 50 balls between the two buckets and that any balls which were not allocated by the end of the five minutes were worth nothing.^[[1]](#footnote-1)^ No other information was provided. Therefore, assuming a participant allocated all 50 balls, the minimum amount that he/she could earn was £2.50 (NI; *COP* $5.000 Bogotá) if he/she followed the rule completely and allocated all 50 balls to the blue bucket. The maximum amount that could be earned was £5.00 (NI; *COP* $10.000 in Bogotá) if he/she completely ignored the rule and allocated all 50 balls to the yellow bucket. The central premise is that the more a participant cares intrinsically about rule-following the more willing he/she will be to incur the costs of doing so (26). The extent of rule-following in the RF task provides a measure of individual norm-following proclivity, and this norm sensitivity measure has been shown to correlate with willingness to follow norms of cooperation, reciprocity and pro-social behavior across decision contexts (26). To avoid introducing any potential biases due to preference for bucket placement, participants were randomized to a version of the RF task with the blue bucket on the left (n=621 baseline, n=616 follow-up), or a version with the blue bucket on the right (n=650 baseline, n=635 follow-up).

**Parts 2-3: Measuring injunctive and descriptive social norms**

Parts 2 and 3 of the experiment consisted of a series of incentivized co-ordination games which used methods employed by Krupka and Weber to elicit injunctive and descriptive social norms around smoking and vaping (28). Injunctive norms reflect shared beliefs among members of a population about what actions people *ought* *to* take; descriptive norms reflect shared beliefs among members of a population about what actions people *actually do* take (28).

In Part 2, participants were asked to rate the social appropriateness of various actions that others might take on a six-point Likert scale: "extremely socially inappropriate", "very socially inappropriate", "somewhat socially inappropriate", "somewhat socially appropriate", "very socially appropriate", "extremely socially appropriate". Situation 1 aimed to elicit pro-sociality injunctive norms by asking participants to co-ordinate with others in their school year group to rate the social appropriateness of a range of actions one might take in a standard Dictator game. The Dictator game is commonly used as a measure of social preferences, in particular, altruism. Such norms are unlikely to be affected by interventions targeted at altering smoking behavior. Eight items (situations 2-9) were asked to assess smoking- and vaping-related injunctive social norms. In Part 3, participants were asked to estimate the proportion of peers in their school year group who would be accepting of certain behaviors on a six-point Likert scale: "none of my peers", "only a few of my peers", "some of my peers", "a lot of my peers", "most of my peers", "all of my peers". Two items were asked to assess smoking- and vaping-related descriptive social norms.

The principal feature of this part of the experiment is that participants are provided with incentives to *match* their ratings/estimates to other participants' in their school year group on the day as opposed to providing personal opinions. For example, participants are informed that they will receive £10 (NI; *COP* $15.000 Bogotá) if the answer they provide for a randomly selected question matches the most common answer in the school year group. Assuming that a norm exists, and in the absence of peer interaction, participants attempting to match others' responses in order to win the incentive will anticipate the extent to which others will rate an action as socially appropriate or inappropriate (or anticipate the extent to which others will estimate that a large or small proportion of their peers would be accepting of certain behaviors), and respond accordingly.^[[2]](#footnote-2)^ Therefore, in Part 2 of the experiment participants play a co-ordination game in which the incentive elicits an empirical measure of injunctive social norms as collective perceptions of the social appropriateness of various behaviors. In Part 3 they play a co-ordination game in which the incentive elicits an empirical measure of descriptive social norms as collective perceptions of the rate of acceptance of certain behaviors.

The components elicited in Parts 1-3 of the experiment can be examined within the context of a norm-dependent utility framework to further our understanding of how the existence of social norms, and individuals' norm sensitivities, can influence behavior in social settings (26–28). Within this framework, behavioral heterogeneity in a given social context is proposed as being related to the fact that people suffer disutility from violating norms and that those individuals differ in sensitivity to own-norm violations.

u(a_k_) = V {ᴫ(a_k_)} + γN(a_k_) (1)

In the above framework (1), social norms are modelled quantitatively, such that a decision maker’s "pay-off", u(a_k_), from each action, V {ᴫ(a_k_)}, is traded off against the normative appropriateness of each action according to the parameter γ≥0, representing the degree to which the individual cares about adhering to social norms, with the function N capturing the social norm. N_g_(a_k_) denotes the social norms for group g, which are estimated from the co-ordination games in Parts 2 and 3 of the experiment. γ is the parameter reflecting individual sensitivity to the norm, estimated using the total number of balls allocated to the blue "rule-following" bucket in the RF task of Part 1.

**Part 4: Measuring willingness to pay to support anti-smoking norms**

Part 4 of the experiment gives us a chance to test the implications of this model. Participants are given an endowment of £5 (NI; *COP* $10.000 Bogotá) and asked to decide how much of the £5 to donate to the organizations responsible for ASSIST/Dead Cool, depending on which program their school was taking part in, and how much to keep for themselves. They are provided with a description of ASSIST/Dead Cool as "a smoking prevention program which aims to prevent the uptake of smoking among adolescents your age". Therefore, in the same way that a willingness to incur a cost to follow the rule in the RF task reveals a respect for following norms in general, the extent of a participant's willingness to incur a cost to make a higher donation to a program whose aim is to encourage smoking reduction by others reveals their support for anti-smoking norms. Since a donation may be taken as revealing a participant's belief that such smoking prevention programs are normatively appealing and effective, this task may be taken as providing evidence for the behavioral impact of an injunctive anti-smoking social norm.

To connect this to the model (1), we need only assume that V is increasing in the participant’s own payoff; then when facing the decision about whether to donate to the anti-smoking intervention charity, subjects trade off their own higher payoff from keeping the money for themselves against the normative appropriateness of donating to help prevent smoking. The theory implies that when norms are stronger, or an individual’s γ is larger, the amount donated will be higher.

Further details on the smoking- and vaping-related scenarios assessed in Parts 2 and 3 of the experiment and numerical coding of responses are provided in Table 1 of the manuscript. Responses to the experiment items from Part 2 were coded such that numerical responses ran between -1 (extremely socially inappropriate) and +1 (extremely socially appropriate) similar to Krupka & Weber (2013) (28). Similarly, responses to experiment items from Part 3 were coded such that numerical responses ran between -1 (none of my peers) and +1 (all of my peers).

**English and Spanish language versions of the experimental protocol.**


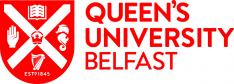


**Experimental Instructions**

**General information**

This is a study about decision-making. You will be paid a fee of £5 for taking part, as outlined below. In addition, you may receive some extra money based on your choices and the choices made by others during the study.

If you have any questions during the session, please raise your hand and wait for a researcher to come to you.  Please do not talk or try to communicate with other participants during the experiment.  It is important that everyone taking part makes his or her own decisions.

This is an on-going study, which has received funding from the UK Medical Research Council to cover all current and future costs. You can be certain that all participants who complete the study will be paid as described in the instructions. If you have any concerns, please contact:

**Dr. Ruth Hunter**
Centre for Public Health/UKCRC Centre of Excellence for Public Health (NI)
School of Medicine, Dentistry and Biomedical Sciences
Institute of Clinical Science B, Royal Victoria Hospital
Grosvenor Road, Belfast, BT 12 6BJ
E-mail: ruth.hunter@qub.ac.uk; 
Tel: +44 (0) 28 90978944
 
**There are four parts to today’s study.**

**You can earn money in each part.**

Your earnings from today will **not** be paid to you today. We will come back to your school at the end of the program in ten weeks’ time. At that time, we would like you to participate in another study. There will be four parts to that study, and you can earn money in each part of that study too.

After you have participated in the study at the end of the program we will determine for each part whether you receive earnings from today or from the study at the end of the program. For each part, we will toss a coin to determine this. We will record your choices in both today’s study and the study at the end of the program. You will be able to review your choices from both experiments when you learn your payment, if you wish.

**Part 1**

In Part 1 of this study, you will decide how to allocate 50 balls between two buckets. Your task is to put each of the balls, one-by-one, into one of the two buckets: the blue bucket or the yellow bucket. The balls will appear to the left-hand side of your screen, and you can allocate each ball by clicking and dragging it to the bucket of your choice. For each ball you put in the blue bucket, you will receive 5 pence, and for each ball you put in the yellow bucket, you will receive 10 pence.

The rule is to put the balls in the blue bucket.

Once the experiment begins, you will have 5 minutes to put the balls into the buckets. When you are finished, please click on the next button and wait quietly for further instructions from the experimenter. Any balls that have not been placed in a bucket at the end of the 5 minutes are worth nothing. Your earnings from Part 1 will be based on your decisions: it is the sum of earnings from the blue and yellow buckets.

This is the end of the instructions for Part 1. If you have any questions, please raise your hand and a researcher will answer them privately. Otherwise, please wait quietly until all of your classmates are ready and click on the next button to begin the experiment.


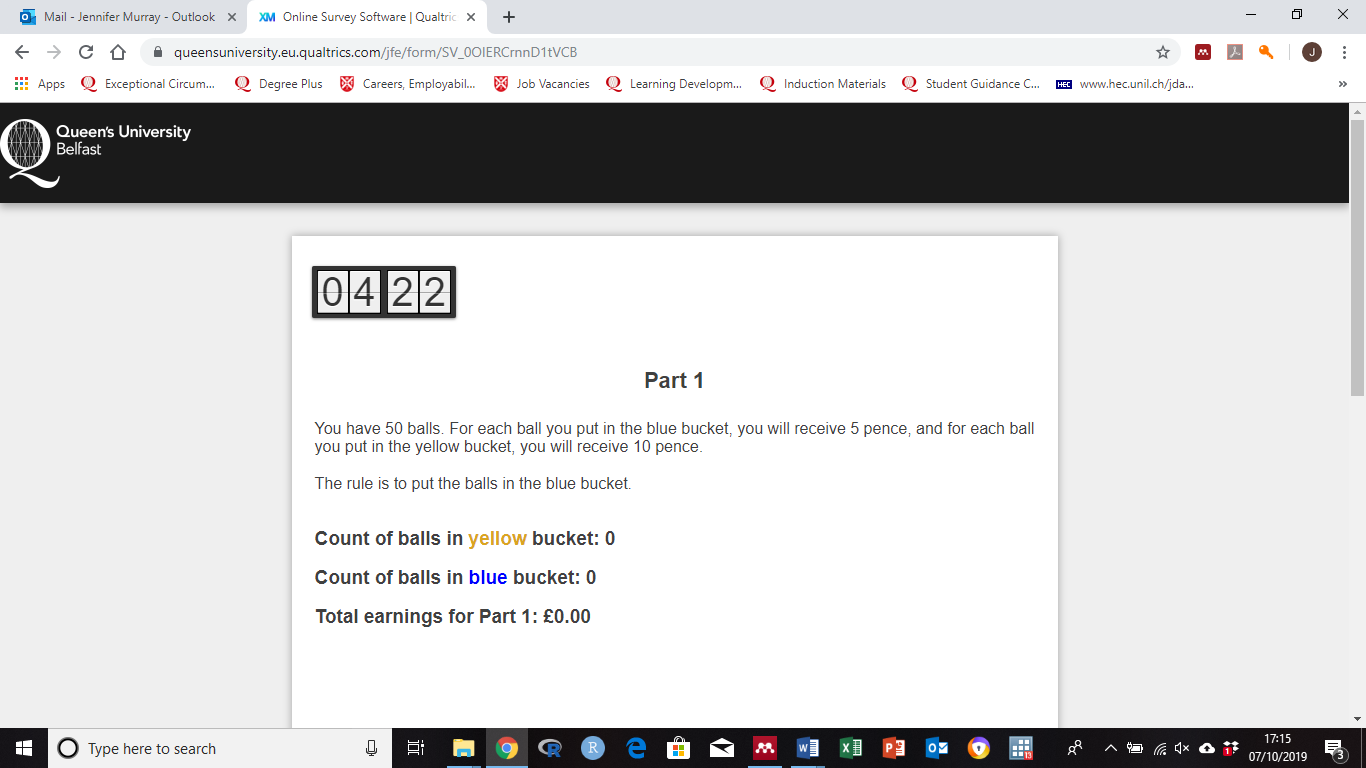


**The 50 balls can be re-located individually to either the blue or yellow bucket by mouse click and drag-and-drop.**

**Updated as balls are dragged in or out of the yellow bucket.**

**Updated as balls are dragged in or out of the blue bucket.**

**Timer indicating five-minute count-down for completing Rule-Following task.**


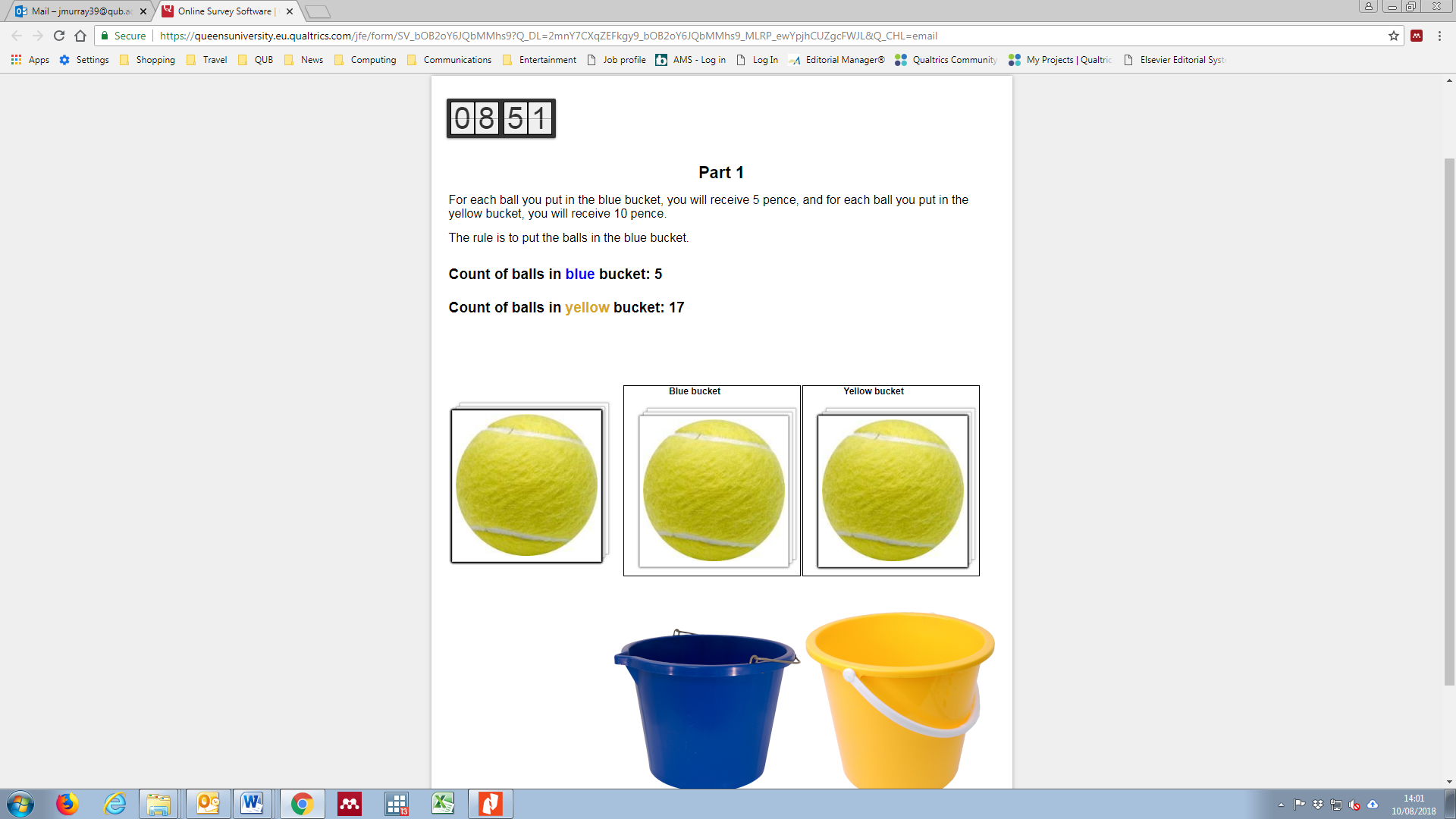


**N.B. Participants were randomized to this version of the experiment or to a version that had the buckets in reverse order to overcome any potential bias due to positioning of buckets.**

**Part 2**

On the following screens, you will read descriptions of a series of situations. These descriptions correspond to situations in which one person must make a decision or has taken an action. For each situation, you will be given a description of the decision faced or action taken by this person.

After you read the description of the situation, you will be asked to evaluate the decision or action taken. You will be asked to decide whether taking that decision or action would be "socially appropriate" and "consistent with moral or proper social behaviour" or "socially inappropriate" and "inconsistent with moral or proper social behaviour". By socially appropriate, we mean behaviour that most people in your school year group agree is the "correct" or "ethical" thing to do. Another way to think about what we mean is that if the person in the situation were to select a socially inappropriate choice, then someone else in your school year group might be angry with that person for doing so.

In each of your responses, we would like you to answer as truthfully as possible, based on your opinions of what constitutes socially appropriate or socially inappropriate behaviour.

To give you an idea of how the experiment will proceed, we will go through an example and show you how you will indicate your responses. On the next screen you will see an example of a situation.

**Part 2**

**Example Situation**

A person is at a local coffee shop near school. While there, the person notices that someone has left a wallet at one of the tables. The person must decide what to do. This person has four possible choices: take the wallet, ask others nearby if the wallet belongs to them, leave the wallet where it is, or give the wallet to the shop manager. The person can choose one of these four options.

The table below presents a list of the possible choices available to this person. For each of the choices, you will be asked to indicate whether you believe choosing that option is extremely socially inappropriate, very socially inappropriate, somewhat socially inappropriate, somewhat socially appropriate, very socially appropriate, or extremely socially appropriate. To indicate your response, you would select the corresponding option.

**The person's choice...**

|  | Extremely socially inappropriate | Very socially inappropriate | Somewhat socially inappropriate | Somewhat socially appropriate | Very socially appropriate | Extremely socially appropriate |
| --- | --- | --- | --- | --- | --- | --- |
| Take the wallet |  |  |  |  |  |  |
| Ask others nearby if the wallet belongs to them |  |  |  |  |  |  |
| Leave the wallet where it is |  |  |  |  |  |  |
| Give the wallet to the shop manager |  |  |  |  |  |  |

**Please make sure that you have placed one tick in each row.**

If this were one of the situations for this study, you would consider each of the possible choices above and, for that choice, indicate the extent to which you believe taking that action would be socially appropriate" and "consistent with moral or proper social behaviour" or "socially inappropriate" and "inconsistent with moral or proper social behaviour".  Recall that by socially appropriate we mean behaviour that most people agree is the "correct" or "ethical" thing to do.

**Part 2**

For example, suppose you thought that taking the wallet was extremely socially inappropriate, asking others nearby if the wallet belongs to them was somewhat socially appropriate, leaving the wallet where it is was somewhat socially inappropriate, and giving the wallet to the shop manager was extremely socially appropriate.  Then you would indicate your responses as follows:


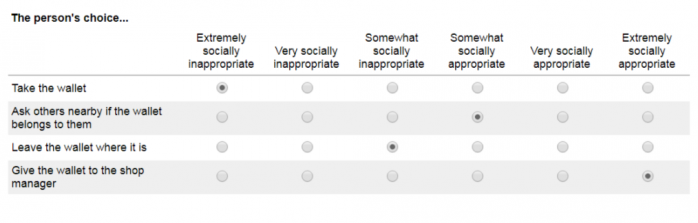
Are there any questions about this example situation or about how to indicate your responses?  On the following pages, there are several situations, all dealing with decisions that another person might have to make. 

You will indicate your appropriateness rating by selecting the corresponding option.

At the end of the experiment today, we will randomly select one of the situations. For this situation, we will also randomly select one of the possible choices that Individual A could make. Thus, we will select both a situation and one possible choice at random. For the choice selected, we will find out which response was selected by most people in your school year group today. 

If you give the same response as that most frequently given by other people in your school year group, then your earning from Part 2 will be £10.  This amount will be paid to you, in cash, at the conclusion of the study in ten weeks. For instance, if we were to select the example situation above and the possible choice "Leave the wallet where it is", and if your response had been "somewhat socially inappropriate", then your earning from Part 2 would be £10, if this was the response selected by most other people in your school year group today. Otherwise your earning from Part 2 would be £0.

You are now going to complete some similar questions to this example on your own. You can go at your own pace.

***If you have any questions from this point on, please raise your hand and wait for the researcher to come to you.***

**Part 2**

**Situation 1**

Consider two hypothetical individuals from your school year group – Individual A and Individual B. Suppose that Individual A is randomly paired with another person in your school year group, Individual B in an experiment. The pairing is anonymous, meaning that neither individual will ever know the identity of the other individual with whom he or she is paired.

In this hypothetical experiment, Individual A will make a choice, the researcher will record this choice, and then both individuals will be informed of the choice and paid money based on the choice made by Individual A, as well as a small participation fee. Suppose that neither individual will receive any other money for participating in the experiment.

In each pair, Individual A will receive £10.  Individual A will then have the opportunity to give any amount of his or her £10 to Individual B. That is, Individual A can give any of the £10 he or she receives to Individual B. For instance, Individual A may decide to give £0 to Individual B and keep £10 for him or herself. Or Individual A may decide to give £10 to Individual B and keep £0 for him or herself. Individual A may also choose to give any other amount between £0 and £10 to Individual B. This choice will determine how much money each will receive, privately and in cash, at the end of the experiment.

The table below gives a list of the possible choices available to Individual A. For each of the choices, please indicate whether you believe choosing that option is extremely socially inappropriate, very socially inappropriate, somewhat socially inappropriate, somewhat socially appropriate, very socially appropriate, or extremely socially appropriate. To indicate your response, please select the corresponding option.

**Remember that you will earn money (£10) if your response to a randomly selected question is the same as the most common response provided in your school year group today.**

**Individual A's choice...**

|  | Extremely socially inappropriate | Very socially inappropriate | Somewhat socially inappropriate | Somewhat socially appropriate | Very socially appropriate | Extremely socially appropriate |
| --- | --- | --- | --- | --- | --- | --- |
| Give £0 to Individual B (Individual A gets £10, Individual B gets £0) |  |  |  |  |  |  |
| Give £1 to Individual B (Individual A gets £9, Individual B gets £1) |  |  |  |  |  |  |
| Give £2 to Individual B (Individual A gets £8, Individual B gets £2) |  |  |  |  |  |  |
| Give £3 to Individual B (Individual A gets £7, Individual B gets £3) |  |  |  |  |  |  |
| Give £4 to Individual B (Individual A gets £6, Individual B gets £4) |  |  |  |  |  |  |
| Give £5 to Individual B (Individual A gets £5, Individual B gets £5) |  |  |  |  |  |  |
| Give £6 to Individual B (Individual A gets £4, Individual B gets £6) |  |  |  |  |  |  |
| Give £7 to Individual B (Individual A gets £3, Individual B gets £7) |  |  |  |  |  |  |
| Give £8 to Individual B (Individual A gets £2, Individual B gets £8) |  |  |  |  |  |  |
| Give £9 to Individual B (Individual A gets £1, Individual B gets £9) |  |  |  |  |  |  |
| Give £10 to Individual B (Individual A gets £0, Individual B gets £10) |  |  |  |  |  |  |

**If you have any questions, please raise your hand and wait for the experimenter.**

**Part 2**

**Situation 2**

**A parent is smoking in their own home in front of their children who are under the age of 5.**

Please indicate whether you believe the parent smoking at home in front of their young children is extremely socially inappropriate, very socially inappropriate, somewhat socially inappropriate, somewhat socially appropriate, very socially appropriate, or extremely socially appropriate. To indicate your response, please select the corresponding option.

**Remember that you will earn money (£10) if your response to a randomly selected question is the same as the most common response provided in your school year group today.**

**A parent smoking at home in front of their young children.**

- Extremely socially inappropriate
- Very socially inappropriate
- Somewhat socially inappropriate
- Somewhat socially appropriate
- Very socially appropriate
- Extremely socially appropriate

**Part 2**

**Situation 3**

**An adult is smoking in a car with children under the age of 16 in the car.**

Please indicate whether you believe the adult smoking in the car with children in the car is extremely socially inappropriate, very socially inappropriate, somewhat socially inappropriate, somewhat socially appropriate, very socially appropriate, or extremely socially appropriate. To indicate your response, please select the corresponding option.

**Remember that you will earn money (£10) if your response to a randomly selected question is the same as the most common response provided in your school year group today.**

**Adult smoking in a car with children on board.**

- Extremely socially inappropriate
- Very socially inappropriate
- Somewhat socially inappropriate
- Somewhat socially appropriate
- Very socially appropriate
- Extremely socially appropriate

**Part 2**

**Situation 4**

**Someone sells cigarettes to a teenager who looks younger than 16 without requesting proof of age.**

Please indicate whether you believe someone selling cigarettes without proof of age is extremely socially inappropriate, very socially inappropriate, somewhat socially inappropriate, somewhat socially appropriate, very socially appropriate, or extremely socially appropriate. To indicate your response, please select the corresponding option.

**Remember that you will earn money (£10) if your response to a randomly selected question is the same as the most common response provided in your school year group today.**

**Someone selling cigarettes without proof of age.**

- Extremely socially inappropriate
- Very socially inappropriate
- Somewhat socially inappropriate
- Somewhat socially appropriate
- Very socially appropriate
- Extremely socially appropriate

**Part 2**

**Situation 5**

**In a recent superhero movie the lead actor is seen smoking in the opening scene.**

Please indicate whether you believe the movie showing the lead character smoking is extremely socially inappropriate, very socially inappropriate, somewhat socially inappropriate, somewhat socially appropriate, very socially appropriate, or extremely socially appropriate. To indicate your response, please select the corresponding option.

**Remember that you will earn money (£10) if your response to a randomly selected question is the same as the most common response provided in your school year group today.**

**A movie showing the lead character smoking.**

- Extremely socially inappropriate
- Very socially inappropriate
- Somewhat socially inappropriate
- Somewhat socially appropriate
- Very socially appropriate
- Extremely socially appropriate

**Part 2**

**Situation 6**

**An older student in your school is smoking outside school, for example, at a bus stop.**

Please indicate whether you believe an older student smoking outside school is extremely socially inappropriate, very socially inappropriate, somewhat socially inappropriate, somewhat socially appropriate, very socially appropriate, or extremely socially appropriate. To indicate your response, please select the corresponding option.

**Remember that you will earn money (£10) if your response to a randomly selected question is the same as the most common response provided in your school year group today.**

**An older student from your school smoking outside school.**

- Extremely socially inappropriate
- Very socially inappropriate
- Somewhat socially inappropriate
- Somewhat socially appropriate
- Very socially appropriate
- Extremely socially appropriate

**Part 2**

**Situation 7**

**A pupil from your school is using an e-cigarette while walking to school.**

Please indicate whether you believe the pupil using an e-cigarette is extremely socially inappropriate, very socially inappropriate, somewhat socially inappropriate, somewhat socially appropriate, very socially appropriate, or extremely socially appropriate. To indicate your response, please select the corresponding option.

**Remember that you will earn money (£10) if your response to a randomly selected question is the same as the most common response provided in your school year group today.**

**A school student smoking an e-cigarette.**

- Extremely socially inappropriate
- Very socially inappropriate
- Somewhat socially inappropriate
- Somewhat socially appropriate
- Very socially appropriate
- Extremely socially appropriate

**Part 2**

**Situation 8**

**A pupil from your school shares a photograph of him/herself using an e-cigarette on social media (e.g. Facebook, Instagram).**

Please indicate whether you believe the pupil sharing an image of e-cigarette use is extremely socially inappropriate, very socially inappropriate, somewhat socially inappropriate, somewhat socially appropriate, very socially appropriate, or extremely socially appropriate. To indicate your response, please select the corresponding option.

**Remember that you will earn money (£10) if your response to a randomly selected question is the same as the most common response provided in your school year group today.**

**A student sharing a photo of his/her e-cigarette use.**

- Extremely socially inappropriate
- Very socially inappropriate
- Somewhat socially inappropriate
- Somewhat socially appropriate
- Very socially appropriate
- Extremely socially appropriate

**Part 2**

**Situation 9**

**A pupil from your school is chewing tobacco.**

Please indicate whether you believe the pupil chewing tobacco is extremely socially inappropriate, very socially inappropriate, somewhat socially inappropriate, somewhat socially appropriate, very socially appropriate, or extremely socially appropriate. To indicate your response, please select the corresponding option.

**Remember that you will earn money (£10) if your response to a randomly selected question is the same as the most common response provided in your school year group today.**

**A school pupil chewing tobacco.**

- Extremely socially inappropriate
- Very socially inappropriate
- Somewhat socially inappropriate
- Somewhat socially appropriate
- Very socially appropriate
- Extremely socially appropriate

**This is the end of Part 2 of the experiment.**

In Part 3 of today's experiment you will be asked some questions about the behaviour of your peers. By peers, we mean “other students in your school year group”. After today we will randomly select a question from part 3. If you give the same response as that most frequently given by other people in your school year group, then your earning from Part 3 will be £10. This amount will be paid to you, in cash, at the conclusion of the study in ten weeks. Please click on the next button when you are ready to proceed.

**Part 3**

**Question 1**

**What share of your school year group would be accepting of one of their close friends smoking?**

*Please indicate what proportion of students in your school year group (your peers) you believe would be accepting of one of their close friends smoking: All of my peers; most of my peers; a lot of my peers; some of my peers; only a few of my peers; none of my peers. To indicate your response, please select the corresponding option.*

**Remember that you will earn money (£10) if your response to a randomly selected question is the same as the most common response provided in your school year group today.**

**The proportion of my peers who would be accepting of a close friend smoking.**

- All of my peers
- Most of my peers
- A lot of my peers
- Some of my peers
- Only a few of my peers
- None of my peers

**Part 3**

**Question 2**

**What share of your school year group would be accepting of one of their close friends vaping (using an e-cigarette)?**

Please indicate what proportion of students *in your school year group (your peers)* you believe would be accepting of one of their close friends using an e-cigarette: All of my peers; most of my peers; a lot of my peers; some of my peers; only a few of my peers; none of my peers. To indicate your response, please select the corresponding option.

**Remember that you will earn money (£10) if your response to a randomly selected question is the same as the most common response provided in your school year group today.**

**The proportion of my peers who would be accepting of a close friend vaping.**

- All of my peers
- Most of my peers
- A lot of my peers
- Some of my peers
- Only a few of my peers
- None of my peers

**This is the end of Part 3 of the experiment.**

Please wait for the experimenter to tell you when to proceed to Part 4.

**Part 4**

You will be given 10 virtual tokens. Each token is worth 50 pence. That means you will receive tokens worth £5.

You will then have the opportunity to give any amount of your £5 to the ASSIST Program.

ASSIST is a smoking prevention program which aims to prevent the uptake of smoking among adolescents your age.

You can give any of the £5 you receive to ASSIST. For instance, you may decide to give £0 to ASSIST and keep £5 for yourself. Or you may decide to give £5 to ASSIST and keep £0 for yourself. You may also choose to give any other amount between £0 and £5 to ASSIST.

The value of any tokens you do not give to ASSIST will be your earnings for this Part. That is, each token that you do not give to ASSIST will increase your own payment for Part 4 by 50 pence.

**How many tokens do you want to give to ASSIST?**

- 0 (you earn £5.00)
- 1 (you earn £4.50)
- 2 (you earn £4.00)
- 3 (you earn £3.50)
- 4 (you earn £3.00)
- 5 (you earn £2.50)
- 6 (you earn £2.00)
- 7 (you earn £1.50)
- 8 (you earn £1.00)
- 9 (you earn £0.50)
- 10 (you earn £0.00)

**
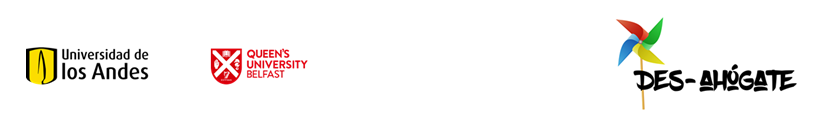
**

**Instrucciones para los experimentos**

**Información general**

Este es un estudio sobre toma de decisiones. Por participar te daremos un monto de $ 5.000 en una tarjeta de regalo. Además, puedes recibir un dinero extra en esta tarjeta en función de tus elecciones y las decisiones tomadas por otros durante el estudio.

Si tienes alguna pregunta durante la sesión, levanta la mano y espera a la persona encargada. Es muy importante que no hables ni trates de comunicarte con otros estudiantes durante el experimento. También es importante que todos los participantes tomen sus propias decisiones.

Este es un estudio que recibe financiación del Consejo de Investigación Médica del Reino Unido y cubrirá todos los gastos actuales y futuros. Por eso, puedes estar seguro de que a todos los estudiantes que participan se les pagará como se describe en las instrucciones en una tarjeta de regalo. Si tienes alguna duda, comunícate con el encargado en el salón o con Sharon Sánchez en la Facultad de Medicina de la Universidad de los Andes al teléfono 3394949 ext.3803 o al correo sc.sanchez@uniandes.edu.co.

**Hay cuatro partes en el estudio de hoy. Puedes ganar dinero en cada parte.**

Tus ganancias NO se pagarán hoy. La tarjeta de regalo será entregada al final del programa en 16 semanas. En ese momento volveremos para realizar otro estudio similar a este en el que podrás ganar dinero en cada parte. Tus ganancias dependen de tus respuestas en la sesión de experimentos de hoy y en la sesión del final del programa. Ten en cuenta que recibirás ganancias solo por una de las sesiones de experimentos, que se elegirá al azar lanzando una moneda. Para poder determinar tus ganancias, registraremos tus respuestas tanto en la sesión de hoy como en la sesión al final del programa. Si lo deseas, al final podrás verificar que el pago asignado corresponde a las respuestas que realizaste durante las dos sesiones.

**Parte 1**

En la parte 1 de este estudio, decidirás cómo encestar 50 pelotas entre dos baldes. Tu tarea es colocar cada una de las pelotas, una a una, en uno de los dos baldes: el balde azul o el balde amarillo. Las pelotas aparecerán en la parte izquierda de la pantalla, y puedes encestar cada pelota haciendo clic y arrastrándola al balde de tu elección. Por cada pelota que pongas en el balde azul, recibirás $100, y por cada pelota que pongas en el balde amarillo, recibirás $200.

La regla es poner las pelotas en el balde azul.

Una vez que comience el experimento, tendrás 5 minutos para poner las pelotas en los baldes. Cuando hayas terminado, espera en silencio hasta que el tiempo se acabe. Las bolas que no hayan sido encestadas en ningún balde no valen nada. Tus ganancias de la parte 1 se basarán en tus decisiones: es la suma de los pagos correspondientes a las pelotas que se encuentren en los baldes azules y amarillos.

Este es el final de las instrucciones para la parte 1. Si tienes alguna pregunta, levanta la mano y el encargado las contestará en privado. De lo contrario, espera en silencio hasta que todo el mundo haya terminado. **Solo cuando el encargado lo indique**, da clic en la siguiente pestaña para comenzar el experimento.

**Parte 1**

Tienes 50 pelotas. Por cada pelota que pongas en el balde azul, recibirás $100, y por cada bola que pongas en el balde amarillo, recibirás $200.


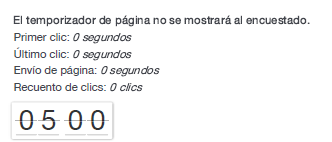


La regla es poner las bolas en el balde azul.

Arrastra y suelta cada pelota dentro del espacio correspondiente. Clasifique los elementos arrastrándolos y colocándolos en su lugar.


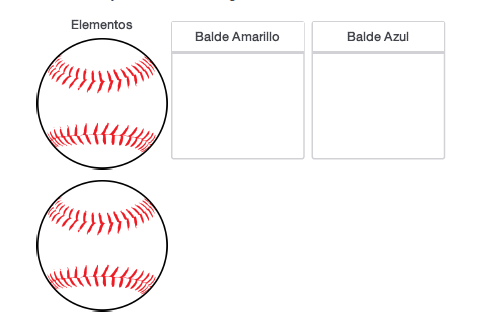


**Este es el final de la Parte 1 del experimento.**

Por favor, espera a que el encargado te indique cuándo empezar con la parte 2.

**Parte 2**

En las siguientes pantallas, se describirán varias situaciones. En estas situaciones una persona debe tomar una decisión o ha realizado una acción. Para cada situación, se describe la decisión o acción tomada por esta persona.

Después de leer la situación, se te pedirá que evalúes la decisión o acción tomada por esa persona. Debes decidir si lo que la persona hace sería “socialmente apropiado” y “consistente con un comportamiento moralmente aceptable o socialmente apropiado” o si el comportamiento de la persona sería “socialmente inapropiado” e “incompatible con una conducta moralmente aceptable o socialmente apropiada”. Por socialmente apropiado, nos referimos al comportamiento que la mayoría de las personas en tu grado escolar está de acuerdo que es lo “correcto” o “ético”. Otra forma de pensar sobre lo que queremos decir es que, si la persona en la situación descrita elije una opción socialmente inapropiada, alguien más en el grado escolar podría estar enojado con esa persona por hacerlo.

En cada una de tus respuestas, nos gustaría que respondas lo más sinceramente posible, en función de tus opiniones sobre lo que es un comportamiento socialmente apropiado o socialmente inapropiado.

Para explicarte cómo procederá el experimento, veremos un ejemplo y te mostraremos cómo podrías indicar tus respuestas. Recuerda que este es solo un ejemplo, tú podrás marcar cada respuesta de acuerdo con lo que opines. No hay respuestas buenas y malas. En la siguiente pantalla, verás un ejemplo de una situación y el encargado del salón lo explicará para todos.

**Parte 2**

**Ejemplo de situación.**

Una persona está en una cafetería cerca del colegio. Mientras está allí, la persona se da cuenta de que alguien ha dejado una billetera en una de las mesas. La persona debe decidir qué hacer. Esta persona tiene cuatro posibles opciones: llevarse la billetera, preguntar a otras personas que estén cerca si la billetera es suya, dejar la billetera donde está o darle la billetera al administrador de la tienda. La persona debe elegir una de las cuatro opciones.

*La siguiente tabla presenta una lista de las posibles opciones disponibles para esta persona. Para cada una de las opciones, debes indicar si crees que elegir esa opción es extremadamente inapropiado, socialmente muy inapropiado, socialmente algo inapropiado, socialmente algo apropiado, socialmente muy apropiado, extremadamente apropiado. Para indicar tu respuesta, marca la casilla correspondiente a la opción deseada:*

| **La persona elige:** | *Extremadamente inapropiado* | *Socialmente muy inapropiado* | *Socialmente algo inapropiado* | *Socialmente algo apropiado* | *Socialmente muy apropiado* | *Extremadamente apropiado* |
| --- | --- | --- | --- | --- | --- | --- |
| Llevarse la billetera | 🞆 | 🞆 | 🞆 | 🞆 | 🞆 | 🞆 |
| Preguntar a personas cercanas si la billetera es suya. | 🞆 | 🞆 | 🞆 | 🞆 | 🞆 | 🞆 |
| Dejar la billetera donde está | 🞆 | 🞆 | 🞆 | 🞆 | 🞆 | 🞆 |
| Darle la billetera al administrador de la tienda. | 🞆 | 🞆 | 🞆 | 🞆 | 🞆 | 🞆 |

**Por favor, asegúrate de seleccionar la opción deseada**

Si esta fuera una de las situaciones para este estudio, tú considerarías cada una de las posibles opciones anteriores y, para esa elección, indicarías hasta qué punto crees que tomar esa acción sería “socialmente apropiado” y “consistente con un comportamiento moralmente aceptable o socialmente apropiado” o si el comportamiento de la persona sería “socialmente inapropiado” e “incompatible con una conducta moralmente aceptable o socialmente apropiada. Recuerda que por “socialmente apropiado” nos referimos al comportamiento con el que la mayoría de las personas está de acuerdo, es lo “correcto” o “ético”.

**Parte 2**

Por ejemplo, supongamos que pensaste que tomar la billetera era *Extremadamente inapropiado*, preguntar a otras personas cercanas si la billetera era suya era *socialmente algo apropiado*, dejar la billetera donde estaba era *socialmente algo inapropiado* y darle la billetera al administrador de la tienda era *Extremadamente apropiado*. Entonces, tu habrías indicado tus respuestas de la siguiente manera:


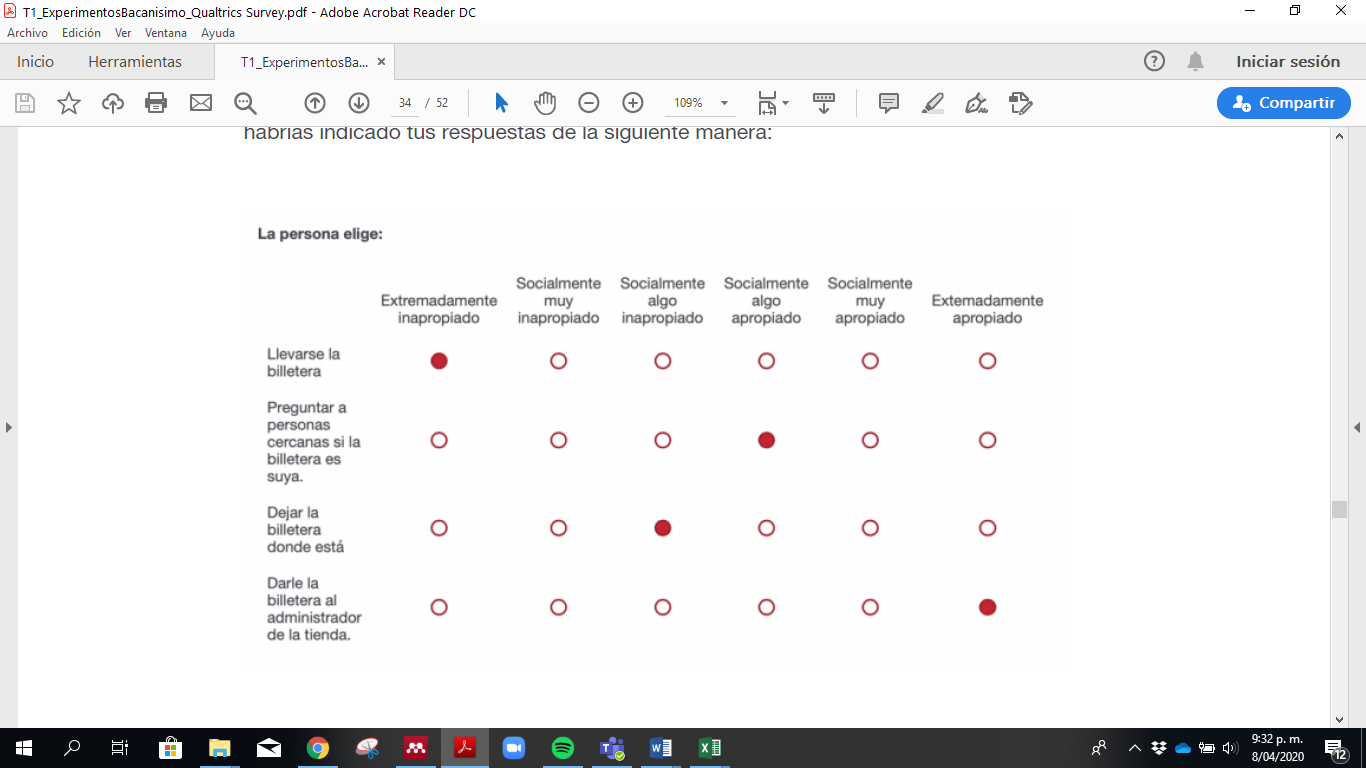


¿Hay alguna pregunta sobre este ejemplo o sobre cómo indicar tus respuestas? En las siguientes páginas, hay varias situaciones, todas relacionadas con decisiones que una persona podría tener que hacer.

Para cada situación, podrás marcar qué tan idóneo te parece cada acción en el recuadro correspondiente.

Al final del experimento de hoy, con un juego de lotería seleccionaremos al azar una de las situaciones. Para esta situación, también seleccionaremos al azar una de las opciones posibles que la persona podría hacer. Por lo tanto, seleccionaremos una situación y una posible elección al azar. Para la opción seleccionada, descubriremos qué respuesta fue seleccionada hoy por la mayoría de tus compañeros en el grado escolar.

Si tú escribes la misma respuesta que la mayoría de tus compañeros de grado, podrás recibir $15.000 por la parte 2 del experimento. Este premio se te entregará en la tarjeta de regalo al final del programa 16 semanas para EntreParceros. Por ejemplo, si en el ejemplo anterior, seleccionáramos al azar para premiar la opción “deje la billetera donde está”, y si tu respuesta fuese la misma que la mayoría de tus compañeros, entonces tu ganancia sería de $15.000, si esta fue la respuesta seleccionada por la mayoría de tus compañeros de grado el día de hoy. De lo contrario, su ganancia de la parte 2 sería $0.

Ahora, vas a completar algunas preguntas similares a este ejemplo por tu cuenta. Puedes ir a tu propio ritmo.

**Si tienes alguna pregunta a partir de este punto, levanta la mano y espera a que el encargado acuda.**

**Parte 2**

**Situación 1.**

Imagina dos personas hipotéticas de tu grado escolar que son ubicados en parejas al azar: Individuo A e Individuo B. El emparejamiento es anónimo, es decir, nadie sabe quién es la pareja de quién.

En este experimento hipotético, el individuo A hará una elección sobre dar dinero al otro, el encargado del curso registrará esta elección, y luego se la informará a ambas personas. Supongamos que ninguna persona recibirá ningún otro dinero diferente a lo que haga en esta situación hipotética.

En la pareja, el individuo A recibe $10.000. El individuo A tendrá entonces la oportunidad de dar cualquier cantidad de sus $10.000 al individuo B. Es decir, el individuo A puede darle al individuo B lo que quiera de los $10.000 que recibió. Por ejemplo, el individuo A puede decidir darle al individuo B $0 y mantener $10.000 para él o ella. O el individuo A puede decidir darle al individuo B los $10.000 y mantener $0 para él o ella. El individuo A también puede optar por dar cualquier otra cantidad entre $0 y $10.000 al individuo B.

Recuerda que tu respuesta sobre lo socialmente apropiado de las acciones del individuo A determinará la ganancia en tu tarjeta de regalo al final del estudio.

*La siguiente tabla presenta una lista de las posibles opciones disponibles para esta persona. Para cada una de las opciones, por favor, indica si crees que elegir esa opción es extremadamente inapropiado, socialmente muy inapropiado, socialmente algo inapropiado, socialmente algo apropiado, socialmente muy apropiado, extremadamente apropiado. Para indicar tu respuesta, marca la casilla correspondiente.*

Recuerda que ganarás un premio ($15.000) en la tarjeta de regalo si tu respuesta a una pregunta seleccionada al azar coincide con la respuesta más común brindada hoy por tus compañeros del grado escolar. Es decir, **para que sea más probable ganar el premio, debes responder según lo que tú crees que la mayoría de tus compañeros piensan.**

El individuo A elige:

| El individuo A elige: | *Extremadamente inapropiado* | *Socialmente muy inapropiado* | *Socialmente algo inapropiado* | *Socialmente algo apropiado* | *Socialmente muy apropiado* | *Extremadamente apropiado* |
| --- | --- | --- | --- | --- | --- | --- |
| Darle $0 al individuo B  (Individuo A obtiene $10.000, Individuo B obtiene $0) | 🞆 | 🞆 | 🞆 | 🞆 | 🞆 | 🞆 |
| Darle $1.000 al individuo B  (Individuo A obtiene $9.000, Individuo B obtiene $1.000 | 🞆 | 🞆 | 🞆 | 🞆 | 🞆 | 🞆 |
| Darle $2.000 al individuo B  (Individuo A obtiene $8.000, Individuo B obtiene $2.000) | 🞆 | 🞆 | 🞆 | 🞆 | 🞆 | 🞆 |
| Darle $3.000 al individuo B  (Individuo A obtiene $7.000, Individuo B obtiene $3.000) | 🞆 | 🞆 | 🞆 | 🞆 | 🞆 | 🞆 |
| Darle $4.000 al individuo B  (Individuo A obtiene $6.000, Individuo B obtiene $4.000) | 🞆 | 🞆 | 🞆 | 🞆 | 🞆 | 🞆 |
| Darle $5.000 al individuo B  (Individuo A obtiene $5.000, Individuo B obtiene $5.000) | 🞆 | 🞆 | 🞆 | 🞆 | 🞆 | 🞆 |
| Darle $6.000 al individuo B  (Individuo A obtiene $4.000, Individuo B obtiene $6.000) | 🞆 | 🞆 | 🞆 | 🞆 | 🞆 | 🞆 |
| Darle $5.000 al individuo B  (Individuo A obtiene $3.000, Individuo B obtiene $7.000) | 🞆 | 🞆 | 🞆 | 🞆 | 🞆 | 🞆 |
| Darle $8.000 al individuo B  (Individuo A obtiene $2.000, Individuo B obtiene $8.000) | 🞆 | 🞆 | 🞆 | 🞆 | 🞆 | 🞆 |
| Darle $9.000 al individuo B  (Individuo A obtiene $1.000, Individuo B obtiene $9.000) | 🞆 | 🞆 | 🞆 | 🞆 | 🞆 | 🞆 |
| Darle $10.000 al individuo B  (Individuo A obtiene $0, Individuo B obtiene $10.000) | 🞆 | 🞆 | 🞆 | 🞆 | 🞆 | 🞆 |

**Si tienes alguna pregunta, por favor levanta la mano y espera por el encargado del salón.**

**Parte 2**

**Situación 2.**

Un padre o una madre fuma en su propia casa frente a sus hijos menores de 5 años.

*Indica si crees que el padre fumando en la casa delante de sus hijos pequeños es extremadamente inapropiado, socialmente muy inapropiado, socialmente algo inapropiado, socialmente algo apropiado, socialmente muy apropiado, extremadamente apropiado. Para indicar tu respuesta marca la casilla correspondiente.*

Recuerda que ganarás un premio ($15.000) en la tarjeta de regalo si tu respuesta a una pregunta seleccionada al azar coincide con la respuesta más común brindada hoy por tus compañeros del grado escolar. Es decir, **para que sea más probable ganar el premio, debes responder según lo que tú crees que la mayoría de tus compañeros piensan.**

Un padre o una madre fuma en su propia casa frente a sus hijos menores de 5 años.

| *Extremadamente inapropiado* | *Socialmente muy inapropiado* | *Socialmente algo inapropiado* | *Socialmente algo apropiado* | *Socialmente muy apropiado* | *Extremadamente apropiado* |
| --- | --- | --- | --- | --- | --- |
| 🞆 | 🞆 | 🞆 | 🞆 | 🞆 | 🞆 |

**Parte 2**

**Situación 3.**

Un adulto fuma en un carro con personas menores de 16 años en el auto.

*Indica si crees que un adulto fumando en un carro con niños menores de 16 años en el carro es extremadamente inapropiado, socialmente muy inapropiado, socialmente algo inapropiado, socialmente algo apropiado, socialmente muy apropiado, extremadamente apropiado. Para indicar tu respuesta marca la casilla correspondiente.*

Recuerda que ganarás un premio ($15.000) en la tarjeta de regalo si tu respuesta a una pregunta seleccionada al azar coincide con la respuesta más común brindada hoy por tus compañeros del grado escolar. Es decir, **para que sea más probable ganar el premio, debes responder según lo que tú crees que la mayoría de tus compañeros piensan.**

Un adulto fuma en un carro con personas menores de 16 años en el auto.

| *Extremadamente inapropiado* | *Socialmente muy inapropiado* | *Socialmente algo inapropiado* | *Socialmente algo apropiado* | *Socialmente muy apropiado* | *Extremadamente apropiado* |
| --- | --- | --- | --- | --- | --- |
| 🞆 | 🞆 | 🞆 | 🞆 | 🞆 | 🞆 |

**Parte 2**

**Situación 4.**

Alguien vende cigarrillos a un adolescente que parece tener menos de 16 años sin pedirle la cédula para comprobar su edad.

*Indica si crees que alguien venda cigarrillos a un adolescente que parece tener menos de 16 años sin solicitar prueba de edad es extremadamente inapropiado, socialmente muy inapropiado, socialmente algo inapropiado, socialmente algo apropiado, socialmente muy apropiado, extremadamente apropiado.*

Recuerda que ganarás un premio ($15.000) en la tarjeta de regalo si tu respuesta a una pregunta seleccionada al azar coincide con la respuesta más común brindada hoy por tus compañeros del grado escolar. Es decir, **para que sea más probable ganar el premio, debes responder según lo que tú crees que la mayoría de tus compañeros piensan.**

Alguien vende cigarrillos a un adolescente que parece tener menos de 16 años sin pedirle la cédula para comprobar su edad.

| *Extremadamente inapropiado* | *Socialmente muy inapropiado* | *Socialmente algo inapropiado* | *Socialmente algo apropiado* | *Socialmente muy apropiado* | *Extremadamente apropiado* |
| --- | --- | --- | --- | --- | --- |
| 🞆 | 🞆 | 🞆 | 🞆 | 🞆 | 🞆 |

**Parte 2**

**Situación 5.**

En una película reciente de superhéroes, se ve al actor principal fumando en la primera escena.

*Indica si crees que en una película mostrando al actor principal fumando en la primera escena* *es extremadamente inapropiado, socialmente muy inapropiado, socialmente algo inapropiado, socialmente algo apropiado, socialmente muy apropiado, extremadamente apropiado.*

Recuerda que ganarás un premio ($15.000) en la tarjeta de regalo si tu respuesta a una pregunta seleccionada al azar coincide con la respuesta más común brindada hoy por tus compañeros del grado escolar. Es decir, **para que sea más probable ganar el premio, debes responder según lo que tú crees que la mayoría de tus compañeros piensan.**

En una película reciente de superhéroes, se ve al actor principal fumando en la primera escena.

| *Extremadamente inapropiado* | *Socialmente muy inapropiado* | *Socialmente algo inapropiado* | *Socialmente algo apropiado* | *Socialmente muy apropiado* | *Extremadamente apropiado* |
| --- | --- | --- | --- | --- | --- |
| 🞆 | 🞆 | 🞆 | 🞆 | 🞆 | 🞆 |

**Parte 2**

**Situación 6.**

Un estudiante mayor de tu colegio está fumando fuera del colegio, por ejemplo, en un paradero de bus.

*Indica si crees que un estudiante mayor de tu colegio está fumando fuera del colegio es extremadamente inapropiado, socialmente muy inapropiado, socialmente algo inapropiado, socialmente algo apropiado, socialmente muy apropiado, extremadamente apropiado. Para indicar tu respuesta marca la casilla correspondiente.*

Recuerda que ganarás un premio ($15.000) en la tarjeta de regalo si tu respuesta a una pregunta seleccionada al azar coincide con la respuesta más común brindada hoy por tus compañeros del grado escolar. Es decir, **para que sea más probable ganar el premio, debes responder según lo que tú crees que la mayoría de tus compañeros piensan.**

Un estudiante mayor de tu colegio está fumando fuera del colegio, por ejemplo, en un paradero de bus.

| *Extremadamente inapropiado* | *Socialmente muy inapropiado* | *Socialmente algo inapropiado* | *Socialmente algo apropiado* | *Socialmente muy apropiado* | *Extremadamente apropiado* |
| --- | --- | --- | --- | --- | --- |
| 🞆 | 🞆 | 🞆 | 🞆 | 🞆 | 🞆 |

**Parte 2**

**Situación 7.**

Un estudiante de tu colegio usa un cigarrillo electrónico mientras camina hacia el colegio.

*Indica si crees que el alumno usando un cigarrillo electrónico mientras camina hacia el colegio* *es extremadamente inapropiado, socialmente muy inapropiado, socialmente algo inapropiado, socialmente algo apropiado, socialmente muy apropiado, extremadamente apropiado. Para indicar tu respuesta marca la casilla correspondiente.*

Recuerda que ganarás un premio ($15.000) en la tarjeta de regalo si tu respuesta a una pregunta seleccionada al azar coincide con la respuesta más común brindada hoy por tus compañeros del grado escolar. Es decir, **para que sea más probable ganar el premio, debes responder según lo que tú crees que la mayoría de tus compañeros piensan.**

Un estudiante de tu colegio usa un cigarrillo electrónico mientras camina hacia el colegio.

| *Extremadamente inapropiado* | *Socialmente muy inapropiado* | *Socialmente algo inapropiado* | *Socialmente algo apropiado* | *Socialmente muy apropiado* | *Extremadamente apropiado* |
| --- | --- | --- | --- | --- | --- |
| 🞆 | 🞆 | 🞆 | 🞆 | 🞆 | 🞆 |

**Parte 2**

**Situación 8.**

Un estudiante de tu colegio comparte una fotografía de sí mismo utilizando un cigarrillo electrónico en redes sociales (Ej. Facebook o Instagram).

*Indica si crees que un alumno de tu colegio compartiendo una fotografía de sí mismo utilizando un cigarrillo electrónico en redes sociales (Ej. Facebook o Instagram*) *es extremadamente inapropiado, socialmente muy inapropiado, socialmente algo inapropiado, socialmente algo apropiado, socialmente muy apropiado, extremadamente apropiado.*

Recuerda que ganarás un premio ($15.000) en la tarjeta de regalo si tu respuesta a una pregunta seleccionada al azar coincide con la respuesta más común brindada hoy por tus compañeros del grado escolar. Es decir, **para que sea más probable ganar el premio, debes responder según lo que tú crees que la mayoría de tus compañeros piensan.**

Un estudiante de tu colegio comparte una fotografía de sí mismo utilizando un cigarrillo electrónico en redes sociales (Ej. Facebook o Instagram).

| *Extremadamente inapropiado* | *Socialmente muy inapropiado* | *Socialmente algo inapropiado* | *Socialmente algo apropiado* | *Socialmente muy apropiado* | *Extremadamente apropiado* |
| --- | --- | --- | --- | --- | --- |
| 🞆 | 🞆 | 🞆 | 🞆 | 🞆 | 🞆 |

**Parte 2**

**Situación 9.**

Un estudiante de tu colegio está masticando tabaco.

*Indica si crees que un estudiante de tu colegio masticando tabaco es extremadamente inapropiado, socialmente muy inapropiado, socialmente algo inapropiado, socialmente algo apropiado, socialmente muy apropiado, extremadamente apropiado. Para indicar tu respuesta marca la casilla correspondiente.*

Recuerda que ganarás un premio ($15.000) en la tarjeta de regalo si tu respuesta a una pregunta seleccionada al azar coincide con la respuesta más común brindada hoy por tus compañeros del grado escolar. Es decir, **para que sea más probable ganar el premio, debes responder según lo que tú crees que la mayoría de tus compañeros piensan.**

Un estudiante de tu colegio está masticando tabaco.

| *Extremadamente inapropiado* | *Socialmente muy inapropiado* | *Socialmente algo inapropiado* | *Socialmente algo apropiado* | *Socialmente muy apropiado* | *Extremadamente apropiado* |
| --- | --- | --- | --- | --- | --- |
| 🞆 | 🞆 | 🞆 | 🞆 | 🞆 | 🞆 |

**Este es el final de la Parte 2 del experimento.**

En la Parte 3 del experimento de hoy responderás varias preguntas sobre el comportamiento de tus compañeros, es decir, otros estudiantes en tu mismo grado escolar. Al final del estudio, con un juego de lotería seleccionaremos al azar una de las situaciones. Para esta situación, también seleccionaremos al azar una de las opciones posibles. Por lo tanto, seleccionaremos una situación y una posible elección al azar. Para la opción seleccionada, descubriremos qué respuesta fue seleccionada por la mayoría de tus compañeros en el grado escolar.

Si tú escribes la misma respuesta que la mayoría de tus compañeros de grado, podrás recibir $15.000 por la parte 3 del experimento. Este premio se te entregará en la tarjeta de regalo al final del programa 16 semanas para EntreParceros.

Por favor, da click en el botón cuando estés listo para empezar con la Parte 3.

**Parte 3**

**Pregunta 1.**

¿Cuántos de tus compañeros del grado aceptarían que uno de sus amigos cercanos fumara?

*Indica qué proporción de estudiantes del grado crees que aceptaría que uno de sus amigos cercanos fumara: todos mis compañeros, la mayoría de mis compañeros, muchos de mis compañeros, algunos de mis compañeros, solo unos pocos de mis compañeros, ninguno de mis compañeros. Para indicar tu respuesta marca la casilla correspondiente.*

Recuerda que ganarás un premio ($15.000) en la tarjeta de regalo si tu respuesta a una pregunta seleccionada al azar coincide con la respuesta más común brindada hoy por tus compañeros del grado escolar. Es decir, **para que sea más probable ganar el premio, debes responder según lo que tú crees que la mayoría de tus compañeros piensan.**

¿Cuántos de tus compañeros del grado aceptarían que uno de sus amigos cercanos fumara?

| *Todos mis compañeros* | *La mayoría de mis compañeros* | *Muchos de mis compañeros* | *Algunos de mis compañeros* | *Solo unos pocos de mis compañeros* | *Ninguno de mis compañeros* |
| --- | --- | --- | --- | --- | --- |
| 🞆 | 🞆 | 🞆 | 🞆 | 🞆 | 🞆 |

**Parte 3**

**Pregunta 2.**

¿Cuántos de tus compañeros del grado aceptarían que uno de tus amigos cercanos usara un cigarrillo electrónico?

*Indica qué proporción de estudiantes del grado crees que aceptaría que uno de sus amigos cercanos usara un cigarrillo electrónico: todos mis compañeros, la mayoría de mis compañeros, muchos de mis compañeros, algunos de mis compañeros, solo unos pocos de mis compañeros, ninguno de mis compañeros. Para indicar tu respuesta marca la casilla correspondiente.*

Recuerda que ganarás un premio ($15.000) en la tarjeta de regalo si tu respuesta a una pregunta seleccionada al azar coincide con la respuesta más común brindada hoy por tus compañeros del grado escolar. Es decir, **para que sea más probable ganar el premio, debes responder según lo que tú crees que la mayoría de tus compañeros piensan.**

¿Cuántos de tus compañeros del grado aceptarían que uno de tus amigos cercanos usara un cigarrillo electrónico?

| *Todos mis compañeros* | *La mayoría de mis compañeros* | *Muchos de mis compañeros* | *Algunos de mis compañeros* | *Solo unos pocos de mis compañeros* | *Ninguno de mis compañeros* |
| --- | --- | --- | --- | --- | --- |
| 🞆 | 🞆 | 🞆 | 🞆 | 🞆 | 🞆 |

**Este es el final de la Parte 3 del experimento**

Por favor, espera a que el encargado del salón te indique que puedes seguir con la Parte 4.

**Parte 4.**

Se te darán 10 fichas virtuales equivalentes a $10.000. Es decir, recibirás 10 fichas de $1.000 cada una. A continuación, tendrás la oportunidad de dar cualquier cantidad de tus $10.000 al programa Des-ahógate [reemplazar con el nombre del programa asignado a la institución Des-ahógate – EntreParceros. El programa de prevención Des-ahógate EntreParceros pretende evitar el consumo de tabaco entre adolescentes de tu edad.

Puedes dar cualquier cantidad de los $10.000 que recibiste a Des-ahógate - EntreParceros. Por ejemplo, puedes decidir dar $0 a Des-ahógate EntreParceros y mantener $10.000 para ti. O puedes decidir dar $10.000 a Des-ahógate EntreParceros y mantener $0 para ti. También puedes optar por dar cualquier otra cantidad entre $0 y $10.000 a Des-ahógate EntreParceros.

La cantidad de dinero que no le des a Des-ahógate EntreParceros hará parte de tus ganancias en esta parte. Recuerda que cada ficha equivale a $1.000.

¿Cuántas **fichas** quieres donar a Des-ahógate EntreParceros?

| 🞆 | 0 (tú ganas $10.000) |
| --- | --- |
| 🞆 | 1 (tú ganas $9.000) |
| 🞆 | 2 (tú ganas $8.000) |
| 🞆 | 3 (tú ganas $7.000) |
| 🞆 | 4 (tú ganas $6.000) |
| 🞆 | 5 (tú ganas $5.000) |
| 🞆 | 6 (tú ganas $4.000) |
| 🞆 | 7 (tú ganas $3.000) |
| 🞆 | 8 (tú ganas $2.000) |
| 🞆 | 9 (tú ganas $1.000) |
| 🞆 | 10 (tú ganas $0) |

Gracias por participar de este experimento.

Tus respuestas serán guardas para determinar tus ganancias.

Recuerda que en 16 semanas semanas volveremos para hacer otro estudio igual a este.

**Social network parameter definitions**

**Clustering coefficient:** The proportion of node connections that are connected amongst each other for each node (29). The local clustering coefficient for node *i* was calculated as the ratio of observed edges between node *i*'s nominated friends to the total number of possible edges between node *i*'s nominated friends. Clustering coefficients were calculated based on the 'directed' network.

**Eigenvector centrality:** A measure of each node's influence in the network in terms of the average connectedness (i.e., centrality) of its connections (29). For each node, the number of connected nodes were summed, weighted proportionally by their centrality (29). In this way, eigenvector centrality can be used to conceptualize individuals' "popularity status" since the "popularity" of node *i* will be proportional to the "popularity" of the nodes to which *i* is connected (30). Eigenvector centralities were calculated after symmetrizing the network (i.e., transforming the 'directed' network into an 'undirected' network by making the top right half of the matrix, above the diagonal, the mirror image of the bottom half of the matrix, below the diagonal) (29).

**Closeness centrality:** A normalized measure of the sum of geodesic distances from a node to all other nodes (29,31). The geodesic distance from one node to another is the length of the shortest path connecting them (29). Closeness centralities were calculated after symmetrizing the network for comparability with eigenvector centralities. Closeness centrality for node *i* was calculated as follows:

$$\boldsymbol{CC}_{\boldsymbol{i}}\boldsymbol{=}\frac{\boldsymbol{N-1}}{\sum_{\boldsymbol{j=1}}^{\boldsymbol{j=N}} \boldsymbol{d}_{\boldsymbol{i,j}}}$$

$\boldsymbol{CC}_{\boldsymbol{i}}$=Closeness centrality for node *i*.

$\boldsymbol{d}_{\boldsymbol{i,j}}$=Geodesic distance (i.e., shortest path) between nodes *i* and *j*, where *i* ≠ *j*.

$\boldsymbol{N}$=Total number of nodes in the network.

**Betweenness centrality:** A standardized measure of the number of times a given node falls on the shortest path between two other nodes (29). Betweenness centralities were calculated after symmetrizing the network for comparability with eigenvector centralities. Betweenness centrality for node *i* was calculated as follows:

$$\boldsymbol{BC}_{\boldsymbol{i}}\boldsymbol{=}\frac{\sum_{\boldsymbol{s\neq i\neq t}}^{\boldsymbol{N}} \frac{\boldsymbol{p}_{\boldsymbol{st}}\boldsymbol{(i)}}{\boldsymbol{p}_{\boldsymbol{st}}}}{\boldsymbol{1/2(N-1)(N-2)}}$$

$\boldsymbol{BC}_{\boldsymbol{i}}$=Betweenness centrality for node *i*.

$\boldsymbol{p}_{\boldsymbol{st}}$=Total number of shortest paths from node *s* to node *t*.

$\boldsymbol{p}_{\boldsymbol{st}}\boldsymbol{(i)}$=Total number of shortest paths from node *s* to node *t* passing through node *i*.

$\boldsymbol{N}$=Total number of nodes in the network.

**Gini degree coefficient (degree heterogeneity/sparsity index):** A measure of the shape of the degree distribution (32). The Gini coefficient is a widely used measure of inequality or heterogeneity, which was originally developed by economists to examine inequality in the skewed distributions of income and wealth (33,34). It is formally defined as the normalized expected difference in degree between two randomly selected nodes in the network (33,34), and is preferable to centralization measures and other measures of degree variability since it is robust to network size and makes no assumption about the shape of the degree distribution (32). Gini degree coefficients were calculated after symmetrizing the network. The Gini degree coefficient for each network was calculated as follows:

$$\boldsymbol{GDC}_{\boldsymbol{g}}\boldsymbol{=}\frac{\boldsymbol{1}}{\bar{\boldsymbol{x}}}\frac{\boldsymbol{1}}{\boldsymbol{2}\boldsymbol{N}^{\boldsymbol{2}}}\sum_{\boldsymbol{i=1}}^{\boldsymbol{N}} \sum_{\boldsymbol{j=1}}^{\boldsymbol{N}} \left| \boldsymbol{x}_{\boldsymbol{i}}\boldsymbol{-}\boldsymbol{x}_{\boldsymbol{j}} \right|$$

$\boldsymbol{GDC}_{\boldsymbol{g}}$=Gini degree coefficient for network *g*.

$\boldsymbol{x}_{\boldsymbol{i}}$**=**Degree of node *i*.

$\boldsymbol{x}_{\boldsymbol{j}}$**=**Degree of node *j*.

$\bar{\boldsymbol{x}}$=Mean degree for all nodes in the network.

$\boldsymbol{N}$=Total number of nodes in the network.

**CONSORT 2010 Flow Diagram**

Participated in follow-up (n=585)

(n=281 NI, n=304 Bogotá)

Participated in follow-up (n=720)

(n=389 NI, n=331 Bogotá)

Received **Dead Cool** intervention (n=600)

(n=286 NI, n=314 Bogotá)

Received **ASSIST** intervention (n=745)

(n=405 NI, n=340 Bogotá)

Selected (N=12)

(N=6 NI, N=6 Bogotá)

Entered the school (n=0)

Left the school (n=8)

Non-attendance to school (n=6)

Withdrawn (n=11)

**Excluded (n=51)**

♦  Declined to participate (n=43)

♦  Left the school (n=4)

♦  No response (n=4)

Assessed for eligibility

(n=648 children)

Assessed for eligibility

(n=796 children)

Allocated to **Dead Cool** intervention (N=6)

(N=3 NI, N=3 Bogotá)

Allocated to **ASSIST** intervention (N=6)

(N=3 NI, N=3 Bogotá)

## Allocation

**Excluded (N=45)**

♦  Declined to participate (N=4)

♦  No answer (N=8)

♦  Unsuitable (N=28)

♦  Offered alternative (N=5)

Assessed for eligibility

(N=57 schools)

## Enrollment

Supplementary Figure S1.1. Study Flow Diagram.

## Follow-Up

## Analysis

**Analysed (n=579)**

♦  Not comparable T1 vs T2 (n=6)

♦  Missing data (n=0)

**Analysed (n=704)**

♦  Not comparable T1 vs T2 (n=16)

♦  Missing data (n=0)

Left the school (n=7)

Non-attendance to school (n=8)

Withdrawn (n=2)

Entered the school (n=2)

**Excluded (n=48)**

♦  Declined to participate (n=45)

♦  Left the school (n=2)

♦  No response (n=1)

Table S1.1. Baseline pupil characteristics for MECHANISMS schools. Mean (SD) unless otherwise stated.

|  | **Northern Ireland (N=6)** | **Bogotá (N=6)** | **All schools (N=12)** |
| --- | --- | --- | --- |
| Intervention, N (schools)/n (pupils) |  |  |  |
| *ASSIST schools* | N=3/n=423 | N=3/n=373 | N=6/n=796 |
| *Dead Cool schools* | N=3/n=295 | N=3/n=353 | N=6/n=648 |
| No. of classes, N | 31 | 24 | 55 |
| No. of pupils, n | 718 | 726 | 1444 |
| Participation, n (%) | 691 (96.2%) | 653 (89.9%) | 1344 (93.1%) |
| School MDM (1-890)^a^ | 343.8 (233.0) | - | - |
| School INSE (1-4)^b^ | - | 2.5 (0.5) | - |
| Individual MDM (1-890)^a^ | 356.7 (241.5) | - | - |
| Individual DANE SES (0-6)^c^ | - | 2.4 (0.9) | - |
| Gender, n(%) |  |  |  |
| *Boys* | 298 (43.1%) | 327 (50.1%) | 625 (46.5%) |
| *Girls* | 321 (46.5%) | 312 (47.8%) | 633 (47.1%) |
| *Prefer not to say* | 11 (1.6%) | 5 (0.8%) | 16 (1.2%) |
| Age, n (%) |  |  |  |
| *11 years old* | 1 (0.1%) | 6 (0.9%) | 7 (0.5%) |
| *12 years old* | 243 (35.2%) | 217 (33.2%) | 460 (34.2%) |
| *13 years old* | 380 (55.0%) | 257 (39.4%) | 637 (47.4%) |
| *14 years old* | 6 (0.9%) | 108 (16.5%) | 114 (8.5%) |
| *15 or more years old* | 0 (0.0%) | 66 (10.1%) | 66 (4.9%) |
| Ethnicity, n (%)^d^ |  |  |  |
| *White British* | 165 (23.9%) | - | 165 (12.3%) |
| *White Irish* | 414 (59.9%) | - | 414 (30.8%) |
| *Colombian: No ethnic minority* | - | 555 (85.0%) | 555 (41.3%) |
| *Ethnic minority* | 49 (7.1%) | 89 (13.6%) | 138 (10.3%) |
| Family |  |  |  |
| *Lives with mother, n (%)* | 608 (88.0%) | 548 (83.9%) | 1156 (86.0%) |
| *Lives with father, n (%)* | 151 (21.9%) | 328 (50.2%) | 479 (35.6%) |
| *Number of family members in household* | 2.6 (0.7) | 2.5 (0.9) | 2.6 (0.8) |

^a^Multiple deprivation measure (NI only; 1=most deprived to 890=least deprived). Calculated based on ranking of NI postcodes for seven domains of deprivation including income; employment; health and disability; education, skills and training; access to services; living environment; crime and disorder. Provided by Northern Ireland Statistics and Research Agency (NISRA).

^b^Socio-economic level index (Bogotá only; 1=Lower; 2=Middle-low; 3=Middle-high; 4=Higher). Calculated each year using a sample from each school, based on the characteristics of the home and its infrastructure, some household assets, the relationship of the children with their parents, among other characteristics. Schools are then classified into four levels according to the average of the responses of the pupils enrolled in them. Provided by the Instituto Colombiano para el Fomento de la Educación Superior (ICFES; “Colombian Institute for the Promotion of Higher Education”).

^c^Socio-economic level index of individual pupils (Bogotá only; 0=Informal settlement; 1=Lowest; 2=Low; 3=Middle-Low; 4=Middle; 5=Middle-High; 6=High), according to the Departamento Administrativo Nacional de Estadística (DANE; "National Administrative Department of Statistics").

^d^White Irish, White British (NI only); Colombian: No ethnic minority (Bogotá only). In Northern Ireland, the ethnic minority groups are "African", "Asian", "Chinese", "Any other ethnic group". The non-ethnic minority groups are "White British", "White Irish". In Bogotá, the ethnic minority groups are "Indigenous", "Gypsy/Roma", "Archipelago Raizal", "Palenquero of San Basilio", "Black/Mulatto/Afro-Colombian/Afro-descendant". The non-ethnic minority groups are "None of the above" (i.e., Colombian no ethnic minority).

**Table S1.2.** Outcomes, moderators, and socio-demographic variables.

| **Outcome** | **Scenario/Question/Derivation** | **Responses/Coding** |
| --- | --- | --- |
| **Experiment Part 2: Injunctive norms (α=0.78)^a^** | | |
| Part 2 Situation 2 (P2S2) | Parent smoking in their own home in front of children under age of 5. | -1=Extremely socially inappropriate; -0.6=Very socially inappropriate; -0.2=Somewhat socially inappropriate; +0.2=Somewhat socially appropriate; +0.6=Very socially appropriate; +1=Extremely socially appropriate. |
| Part 2 Situation 3 (P2S3) | An adult smoking in a car with children under the age of 16 in the car. | *As per P2S2.* |
| Part 2 Situation 4 (P2S4) | Someone selling cigarettes to a teenager who looks younger than 16 without requesting proof of age. | *As per P2S2.* |
| Part 2 Situation 5 (P2S5) | In a recent superhero movie the lead actor is seen smoking in the opening scene. | *As per P2S2.* |
| Part 2 Situation 6 (P2S6) | An older student from school is smoking outside school, for example, at a bus stop. | *As per P2S2.* |
| Part 2 Situation 7 (P2S7) | A pupil from school is using an e-cigarette while walking to school. | *As per P2S2.* |
| Part 2 Situation 8 (P2S8) | A pupil from school shares a photograph of him/herself using an e-cigarette on social media. | *As per P2S2.* |
| Part 2 Situation 9 (P2S9) | A pupil from school is chewing tobacco. | *As per P2S2.* |
| Experiment Part 2 | Experimentally measured injunctive norms. | Average of items P2S2 to P2S9. |
| **Experiment Part 3: Descriptive norms (α=0.85)^a^** | | |
| Part 3 Question 1 (P3Q1) | The proportion of my peers who would be accepting of a close friend smoking. | -1=None of my peers; -0.6=Only a few of my peers; -0.2=Some of my peers; +0.2=A lot of my peers; +0.6=Most of my peers; +1=All of my peers. |
| Part 3 Question 2 (P3Q2) | The proportion of my peers who would be accepting of a close friend vaping. | *As per P3Q1.* |
| Experiment Part 3 | Experimentally measured descriptive norms. | Average of items P3Q1 to P3Q2. |
| **Experiment Part 4: Number of tokens donated to ASSIST/Dead Cool** | | |
| Willingness to pay to support anti-smoking norms (Donation to ASSIST/Dead Cool) | You will be given 10 virtual tokens. Each token is worth 50 pence. That means you will receive tokens worth £5. You will then have the opportunity to give any amount of your £5 to the ASSIST/Dead Cool Program… The value of any tokens you do not give to ASSIST/Dead Cool will be your earnings for this Part… How many tokens do you want to give to ASSIST/Dead Cool? | 0 (0 tokens/£0.00 donated to ASSIST/Dead Cool) to 10 (10 tokens/£5.00 donated to ASSIST/Dead Cool). |
| **Survey: Self-report injunctive norms (α=0.75)^b,c^** | | |
| Injunctive Norms 1 (IN1) | Most of the people who are important to me think that I… | -2=Definitely should smoke; -1=Maybe should smoke; 0=Don't know/neutral; +1=Maybe should not smoke; +2=Definitely should not smoke. |
| Injunctive Norms 2 (IN2) | My mother thinks that I… | *As per IN1. Responses of “I don’t have a mother” were also set to 0.* |
| Injunctive Norms 3 (IN3) | My father thinks that I… | *As per IN1. Responses of “I don’t have a father” were also set to 0.* |
| Injunctive Norms 4 (IN4) | My brother(s) think(s) that I… | *As per IN1. Responses of “I don’t have a brother” were also set to 0.* |
| Injunctive Norms 5 (IN5) | My sister(s) think(s) that I… | *As per IN1. Responses of “I don’t have a sister” were also set to 0.* |
| Injunctive Norms 6 (IN6) | My friends think that I… | *As per IN1. Responses of “I don’t have a friend” were also set to 0.* |
| Injunctive Norms 7 (IN7) | My best friend thinks that I… | *As per IN1. Responses of “I don’t have a best friend” were also set to 0.* |
| Injunctive Norms | Self-report injunctive norms. | Average of items IN1 to IN7. |
| **Survey: Self-report descriptive norms 1 (α=0.54)^b,d^** | | |
| Descriptive Norms 1.1 (DN1.1) | Does your best friend smoke? | 1=Very often; 2=Often; 3=Occasionally; 4=Rarely; 5=Never/Don't know. *Responses of “I don’t have a best friend” were also set to 5.* |
| Descriptive Norms 1.2 (DN1.2) | Does your mother smoke? | *As per DN1.1. Responses of “I don’t have a mother” were also set to 5.* |
| Descriptive Norms 1.3 (DN1.3) | Does your father smoke? | *As per DN1.1. Responses of “I don’t have a father” were also set to 5.* |
| Descriptive Norms 1.4 (DN1.4) | Do any of your brothers smoke? | *As per DN1.1. Responses of “I don’t have a brother” were also set to 5.* |
| Descriptive Norms 1.5 (DN1.5) | Do any of your sisters smoke? | *As per DN1.1. Responses of “I don’t have a sister” were also set to 5.* |
| Descriptive Norms 1 | Self-report descriptive norms scale 1. | Average of items DN1.1 to DN1.5. |
| **Survey: Self-report descriptive norms 2 (α=0.53)^b,d^** | | |
| Descriptive Norms 2.1 (DN2.1) | How many of your friends smoke? | 1=Almost all of them; 2=Many of them; 3=Half of them; 4=A few of them; 5=Almost none of them/Don't know. *Responses of “I don’t have a best friend” were also set to 5.* |
| Descriptive Norms 2.2 (DN2.2) | How many of your other family members smoke? | *As per DN2.1. Responses of “I don’t have other family members” were also set to 5.* |
| Descriptive Norms 2.3 (DN2.3) | How many of your classmates smoke? | *As per DN2.1. Responses of “I don’t have classmates” were also set to 5.* |
| Descriptive Norms 2 | Self-report descriptive norms scale 2. | Average of items DN2.1 to DN2.3. |
| **Survey: Self-report smoking behavior (α=0.83)^b^** | | |
| Current smoking behavior | Do you smoke cigarettes at all nowadays? | 1=Does currently smoke; 2=Does not currently smoke. |
| Smoking behavior | Now read the following statements carefully and tick the box next to the one which best describes you. (I have never smoked; I have only ever tried smoking once; I used to smoke sometimes but I never smoke a cigarette now; I sometimes smoke cigarettes now but I don’t smoke as many as one a week). | 1=Sometimes smoke; 2=Previous smoker; 3=Smoked once; 4=Never smoked. |
| Past/current smoking behavior | Just to check, read the statements below carefully and tick the box next to the one which best describes you. (I have never tried smoking a cigarette, not even a puff or two; I did once have a puff or two of a cigarette, but I never smoke now; I do sometimes smoke cigarettes). | 1=Sometimes smoke; 2=Tried smoking; 3=Never smoked. |
| **Survey: Self-report smoking intentions (α=0.77)^b^** | | |
| Intentions (to quit smoking) | If you DO currently smoke, do you intend to quit smoking in the next six months? | 1=Definitely remain a smoker; 2=Probably remain; 3=Don't know; 4=Probably quit; 5=Definitely quit; 6=I don't smoke. |
| Intentions (to try smoking) | Do you think you will try a cigarette soon? | 1=Yes; 2=Don't know; 3=No. |
| Intentions (friends) | If one of your best friends were to offer you a cigarette, would you smoke it? | 1=Definitely yes; 2=Probably yes; 3=Not sure; 4=Probably not; 5=Definitely not. |
| Intentions | If you DON’T currently smoke, do you intend to take up smoking in the next 6 months? | 1=I am a smoker; 2=Definitely start smoking; 3=Probably start smoking; 4=Don't know; 5=Probably remain; 6=Definitely remain a non-smoker. |
| **Survey: Self-report susceptibility towards smoking^b^** | | |
| Smoking susceptibility | Susceptibility to commencing smoking: Calculated from 3 items: 1. Intentions (to try smoking); 2. Intentions (friends); 3. Intentions. | 0=Not susceptible; 1=Susceptible. Coded 0 if participant responses to the 3 items are: 1. No; 2. Definitely not; 3. Definitely remain a non-smoker. Coded 1 otherwise. |
| **Survey: Self-report smoking knowledge and attitudes^b^** | | |
| Knowledge | Knowledge of smoking. | Count number of correct answers to six questions assessing knowledge of the effects of smoking: 0 (0 correct) to 6 (6 correct). |
| Attitudes | Attitudes towards smoking. | Average of 12 items, each coded 1-5: 1 (least anti-smoking attitudes) to 5 (greatest anti-smoking attitudes), α=0.81. |
| **Survey: Psycho-social antecedents^b^** | | |
| Self-efficacy: Emotional | Self-efficacy (Emotional subscale). | Average of 9 items, each coded 1-6: 1 (lowest self-efficacy to resist smoking) to 6 (greatest self-efficacy to resist smoking), α=0.97. |
| Self-efficacy: Friends | Self-efficacy (Friends subscale). | Average of 9 items, each coded 1-6: 1 (lowest self-efficacy to resist smoking) to 6 (greatest self-efficacy to resist smoking), α=0.96. |
| Self-efficacy: Opportunity | Self-efficacy (Opportunity subscale). | Average of 11 items, each coded 1-6: 1 (lowest self-efficacy to resist smoking) to 6 (greatest self-efficacy to resist smoking), α=0.98. |
| Perceived physical risks | Perceived risks of tobacco-use (Physical subscale). | Average of 7 items, each coded 0%-100%: 0% (lowest perceived risk) to 100% (highest perceived risk), α=0.87. |
| Perceived social risks | Perceived risks of tobacco-use (Social subscale). | Average of 3 items, each coded 0%-100%: 0% (lowest perceived risk) to 100% (highest perceived risk), α=0.71. |
| Perceived addiction risks | Perceived risks of tobacco-use (Addiction subscale). | Average of 3 items, each coded 0%-100%: 0% (lowest perceived risk) to 100% (highest perceived risk), α=0.49. |
| Perceived benefits | Perceived benefits of tobacco-use. | Average of 5 items, each coded 0%-100%: 0% (lowest perceived benefit) to 100% (highest perceived benefit), α=0.79. |
| Perceived behavioral control: easy to quit smoking | If I smoked regularly, I'm sure that it would be easy for me to quit. | 1=Strongly disagree; 2=Disagree; 3=Unsure; 4=Agree; 5=Strongly agree. |
| Perceived behavioral control: to avoid smoking | If I decided not to smoke, I am sure I could avoid smoking. | 1=Strongly disagree; 2=Disagree; 3=Unsure; 4=Agree; 5=Strongly agree. |
| **Smokerlyzer readings: Objectively measured smoking behavior** | | |
| Carbon monoxide reading | Objectively measured smoking behavior over the past 24 hours captured using hand-held carbon monoxide monitors (PICOAdvantage Smokerlyzer, Bedfont) to measure expelled air carbon monoxide in parts per million (ppm) in a range of 0–150 ppm with an accuracy of 2ppm/5% (whichever is greater). | Continuous variable (ppm). |
| **Moderators^b^** | | |
| Setting | Participant country/city. | 0=Northern Ireland; 1=Bogotá. |
| Intervention | Intervention program delivered in participant’s school. | 1=ASSIST; 2=Dead Cool. |
| Gender | Participant gender. | 0=Boy; 1=Girl/Prefer not to say. |
| School socio-economic status | School socio-economic status. Combined measure for NI and Bogotá. | NI: 1=NIMDM2017≤222.5; 2=222.5<NIMDM2017≤445; 3=445<NIMDM2017≤667.5; 4=NIMDM2017>667.5.  Bogotá: 1=Lower; 2=Middle-low; 3=Middle-high; 4=Higher. |
| School socio-economic status (NI only) | School socio-economic status. NI only. | 5.7 (NIMDM2017=57) to 80.2 (NIMDM2017=802). |
| Experiment Part 1: Rule-following | Rule-following (individuals’ norm sensitivities): Number of balls allocated to the blue (rule-following) bucket. | 0 (0 balls allocated to blue bucket; least rule-following) to 5 (50 balls allocated to the blue bucket; most rule-following). |
| Pro-sociality | Pro-sociality scale. | Sum of 5 items, each coded 0-2: 0 (least pro-sociality) to 10 (most pro-sociality), α=0.74. |
| Fear of negative evaluation (FNE) | Fear of negative evaluation scale. | Average of 12 items, each coded 1-5: 1 (least FNE) to 5 (most FNE), α=0.89. |
| Need to belong (NTB) | Need to belong scale. | Average of 10 items, each coded 1-5: 1 (least NTB) to 5 (most NTB), α=0.81. |
| 'Big Five': Openness | 'Big Five' Personality Questionnaire (Openness subscale). | Average of 10 items, each coded 0-4: 0 (least openness) to 4 (most openness), α=0.80. |
| 'Big Five': Extraversion | 'Big Five' Personality Questionnaire (Extraversion subscale). | Average of 10 items, each coded 0-4: 0 (least extraverted) to 4 (most extraverted), α=0.78. |
| 'Big Five': Agreeableness | 'Big Five' Personality Questionnaire (Agreeableness subscale). | Average of 10 items, each coded 0-4: 0 (least agreeable) to 4 (most agreeable), α=0.70. |
| 'Big Five': Conscientiousness | 'Big Five' Personality Questionnaire (Conscientiousness subscale). | Average of 10 items, each coded 0-4: 0 (least conscientious) to 4 (most conscientious), α=0.70. |
| 'Big Five': Emotional stability | 'Big Five' Personality Questionnaire (Emotional stability subscale). | Average of 10 items, each coded 0-4: 0 (least stable) to 4 (most stable), α=0.74. |
| Clustering coefficient | The proportion of node connections that are connected amongst each other for each node (29). Calculated based on the original directed networks. | 0 (least interconnections between nominated friends) to 10 (most interconnections between nominated friends). Scaled up from the original calculated clustering coefficients (0-1) by ten (0-10). |
| Eigenvector centrality | A measure of each individual participant's influence in their school's friendship network measured in terms of the average connectedness (i.e., centrality) of their connections (29). Calculated after symmetrizing the networks. | 0.003 (least well-connected friends, i.e., least central) to 3.13 (most well-connected friends, i.e., most central). Scaled up from the original calculated eigenvector centralities by ten. |
| Closeness centrality | A normalized measure of the sum of geodesic distances (i.e., shortest path between two nodes) from a node to all other nodes (29,31). Calculated after symmetrizing the networks. | 2.15 (longest distance to all other nodes, i.e., least central) to 5.10 (shortest distance to all other nodes, i.e. most central). Scaled up from the original calculated closeness centralities by ten. |
| Betweenness centrality | A standardized measure of the number of times a given node falls on the shortest path between two other nodes (29). Calculated after symmetrizing the networks. | 0 (node falls on the least proportion of shortest paths between two other nodes, i.e., least central) to 5.49 (node falls on the greatest proportion of shortest paths between two other nodes, i.e. most central). Scaled up from the original calculated betweenness centralities by ten. |
| Gini degree coefficient | A measure of the shape of the degree distribution (32), formally defined as the normalized expected difference in degree between two randomly selected nodes in the network (33,34). Calculated after symmetrizing the networks. | 1.70 (least expected difference in degree between two randomly selected nodes, i.e., degree distribution is least heterogeneous) to 2.91 (greatest expected difference in degree between two randomly selected nodes, i.e., degree distribution is most heterogeneous). Scaled up from the original calculated Gini degree coefficients by ten. |
| **Survey: Other self-report socio-demographic variables included as covariates** | | |
| Age | Participant age. | 1=12 years or less; 2=13 years; 3=14 years or more. |
| Ethnicity | Participant ethnicity. | 0=No ethnic minority; 1=ethnic minority. |
| Socio-economic status | Participant socio-economic status. | NI: 1=NIMDM2017≤296.6; 2=296.6<NIMDM2017≤593.2; 3=NIMDM2017>593.2.  Bogotá: 1=Informal settlement/Lowest/Low; 2=Middle-Low/Middle; 3=Middle-High/High. |

^a^Responses to experimental items from Parts 2 and 3 were numerically coded to run between -1 and +1 similar to Krupka & Weber (2013) (28).

^b^All items on the survey were coded such that higher numerical values represented greater anti-smoking norms, greater anti-smoking behavior or intentions, greater knowledge of smoking, greater anti-smoking attitudes, higher values of the psycho-social antecedents, or higher values of the personality characteristics.

^c^Responses to survey injunctive norms items were numerically coded to run between -2 and +2 following Cremers et al., (2014) (35).

^d^Responses to survey descriptive norms items were numerically coded to run between +1 and +5 following Cremers et al., (2014) (35).

**References**

1. Murray JM, Kimbrough EO, Krupka EL, Ramalingam A, Kumar R, Power JM, Sanchez-Franco S, Sarmiento OL, Kee F, Hunter RF. Confirmatory factor analysis comparing incentivized experiments with self-report methods to elicit adolescent smoking and vaping social norms. *Sci Rep* (2020) 10:15818. doi: 10.1038/s41598-020-72784-z

2. Murray JM, Sánchez-Franco SC, Sarmiento OL, Kimbrough EO, Tate C, Montgomery SC, Kumar R, Dunne L, Ramalingam A, Krupka EL, et al. Selection homophily and peer influence for adolescents’ smoking and vaping norms and outcomes in high and middle-income settings. *Humanit Soc Sci Commun* (2023) 10:615. doi: 10.1057/s41599-023-02124-9

3. Murray JM, Sanchez-Franco SC, Sarmiento OL, Kimbrough EO, Tate C, Montgomery SC, Kumar R, Dunne L, Ramalingam A, Krupka EL, et al. Moderators of peer influence effects for adolescents’ smoking and vaping norms and outcomes in high and middle-income settings. *SSRN Electronic Journal* (2024) October 16: doi: 10.2139/ssrn.4989587

4. Krupka E, Leider S, Xu C. *Laboratory on the social network : homophily and peer influence for economic preferences*. Ann Arbor, Michigan: University of Michigan. (2016). 1–31 p.

5. Clauset A, Newman MEJ, Moore C. Finding community structure in very large networks. *Phys Rev E* (2004) 70:066111.

6. Department of Education Northern Ireland. Department of Eduation - Institution Search. (2018) http://apps.education-ni.gov.uk/appinstitutes/default.aspx

7. Department of Education Northern Ireland. School enrolments - school level data 2017/18. (2018) https://www.education-ni.gov.uk/publications/school-enrolments-school-level-data-201718

8. Northern Ireland Statistics and Research Agency. Northern Ireland Multiple Deprivation Measure 2017 (NIMDM2017). (2017) https://www.nisra.gov.uk/statistics/deprivation/northern-ireland-multiple-deprivation-measure-2017-nimdm2017 [Accessed February 23, 2023]

9. Barrera M, Castro FG, Strycker LA, Toobert DJ. Cultural adaptations of behavioral health interventions: a progress report. *J Consult Clin Psychol* (2013) 81:196–205. doi: 10.1037/a0027085

10. Sánchez-Franco S, Arias LF, Jaramillo J, Murray JM, Hunter RF, Llorente B, Bauld L, Good S, West J, Kee F, et al. Cultural adaptation of two school-based smoking prevention programs in Bogotá, Colombia. *Transl Behav Med* (2021) 11:1567–1578. doi: 10.1093/tbm/ibab019

11. Campbell R, Starkey F, Holliday J, Audrey S, Bloor M, Parry-Langdon N, Hughes R, Moore L. An informal school-based peer-led intervention for smoking prevention in adolescence (ASSIST): a cluster randomised trial. *Lancet* (2008) 371:1595–1602. doi: 10.1016/S0140-6736(08)60692-3

12. Thurston A, Dunne L, Kee F, Gildea A, Craig N, Stark P, Lazenbatt A. A randomized controlled efficacy trial of a smoking prevention programme with Grade 8 students in high schools. *Int J Educ Res* (2019) 93:23–32. doi: 10.1016/j.ijer.2018.10.003

13. Rogers EM. *Diffusion of innovations*. 5th ed. New York: Free Press. (2003). 551 p.

14. Ajzen I. The theory of planned behavior. *Organ Behav Hum Decis Process* (1991) 50:179–211. doi: 10.1016/0749-5978(91)90020-T

15. Dunne L, Thurston A, Gildea A, Kee F, Lazenbatt A. Protocol: A randomised controlled trial evaluation of Cancer Focus NI’s ‘Dead Cool’ smoking prevention programme in post-primary schools. *Int J Educ Res* (2016) 75:24–30. doi: 10.1016/j.ijer.2015.06.009

16. StataCorp. Stata Statistical Software: Release 13. College Station, TX: StataCorp LP. (2013)

17. Hunter RF, Montes F, Murray JM, Sanchez-Franco SC, Montgomery SC, Jaramillo J, Tate C, Kumar R, Dunne L, Ramalingam A, et al. MECHANISMS Study: using Game Theory to assess the effects of social norms and social networks on adolescent smoking in schools—study protocol. *Front Public Health* (2020) 8:377. doi: 10.3389/fpubh.2020.00377

18. Huber PJ. “The behavior of maximum likelihood estimates under nonstandard conditions.,” In: Le Cam L, Neyman J, editors. *Proceedings of the Fifth Berkeley Symposium on Mathematical Statistics and Probability, volume 1*. Berkeley, California: University of California Press (1967). p. 221–233

19. White H. A heteroskedasticity-consistent covariance matrix estimator and a direct test for heteroskedasticity. *Econometrica* (1980) 48:817. doi: 10.2307/1912934

20. Nagelkerke NJD. A note on a general definition of the coefficient of determination. *Biometrika* (1991) 78:691–692. doi: 10.1093/biomet/78.3.691

21. Hayes AF. *Introduction to Mediation, Moderation, and Conditional Process Analysis: A Regression-Based Approach*. Guilford Press. (2013). 507 p.

22. Johnson PO, Neyman J. Tests of certain linear hypotheses and their application to some educational problems. *Statistical Research Memoirs* (1936) 1:57–93.

23. Holm S. A simple sequentially rejective multiple test procedure. *Scandinavian Journal of Statistics* (1979) 6:65–70.

24. Steegen S, Tuerlinckx F, Gelman A, Vanpaemel W. Increasing transparency through a multiverse analysis. *Perspectives on Psychological Science* (2016) 11:702–712. doi: 10.1177/1745691616658637

25. Giudice M Del, Gangestad SW. A traveler’s guide to the multiverse: promises, pitfalls, and a framework for the evaluation of analytic decisions. *Adv Methods Pract Psychol Sci* (2021) 4:1–15. doi: 10.1177/2515245920954925

26. Kimbrough EO, Vostroknutov A. Norms Make Preferences Social. *J Eur Econ Assoc* (2016) 14:608–638. doi: 10.1111/jeea.12152

27. Kimbrough EO, Vostroknutov A. A portable method of eliciting respect for social norms. *Econ Lett* (2018) 168:147–150. doi: 10.1016/j.econlet.2018.04.030

28. Krupka EL, Weber RA. Identifying social norms using coordination games: why does dictator game sharing vary? *J Eur Econ Assoc* (2013) 11:495–524. doi: 10.1111/jeea.12006

29. Borgatti SP, Everett MG, Johnson JC. *Analyzing Social Networks*. 2nd ed. London: SAGE Publications Ltd. (2018).

30. Robalino JD, Macy M. Peer effects on adolescent smoking: are popular teens more influential? *PLoS One* (2018) 13:e0189360. doi: 10.1371/journal.pone.0189360

31. Freeman LC. Centrality in social networks conceptual clarification. *Soc Networks* (1978) 1:215–239. doi: 10.1016/0378-8733(78)90021-7

32. Badham JM. Commentary: Measuring the shape of degree distributions. *Network Science* (2013) 1:213–225. doi: 10.1017/NWS.2013.10

33. Gini C. *Variabilità e Mutuabilità. Contributo allo Studio delle Distribuzioni e delle Relazioni Statistiche.* Bologna: Tipogr. di P. Cuppini. (1912). 1–158 p.

34. Dalton H. The measurement of the inequality of incomes. *Source: The Economic Journal* (1920) 30:348–361.

35. Cremers HP, Oenema A, Mercken L, Candel M, De Vries H. Explaining socio-economic differences in intention to smoke among primary school children. *BMC Public Health* (2014) 14:191. doi: 10.1186/1471-2458-14-191

36. Bicchieri C, Muldoon R, Sontuoso A. “‘Social norms,.’” In: Zalta EN, editor. *The Stanford Encyclopedia of Philosophy*. (2018) https://plato.stanford.edu/archives/win2018/entries/social-norms/

1. Every participant allocated all 50 balls to a bucket during the baseline and follow-up experiments during the full phase. There were several changes made to the experimental protocol after baseline was completed in the first pilot school in Northern Ireland. The first version of the experiment included a forced waiting time for the RF task of seven minutes, the idea being to make sure that all pupils proceeded to the second part together. Subsequently it was decided to reduce the time allocated for the RF task from seven minutes to five minutes, to remove the forced waiting time and to insert dummy screens informing participants when to wait for further instructions from the experimenter. Data from MECHANISMS pilot schools are not included in the current paper. [↑](#footnote-ref-1)
2. The measurement property of content validity suggests that in order to be considered adequate, a measurement instrument should adequately reflect the underlying theoretical construct being measured. Thus, one of the advantages of the Krupka-Weber method of eliciting social norms is that the structure of the game itself provides incentives for people to report their beliefs about others’ beliefs about social appropriateness. The existence of such shared “second-order” beliefs are a theoretical precondition for the existence of a social norm according to the work of Bicchieri (36). [↑](#footnote-ref-2)
