# Supplementary material for: Moderators of peer influence effects for adolescents’ smoking and vaping norms and outcomes in high and middle-income settings
Source: Front Psychol. 2025 Nov 3;16:1655761. doi: 10.3389/fpsyg.2025.1655761 (PMC12620383; doi:10.3389/fpsyg.2025.1655761)
Supplement: SUPPLEMENTARY File 2 — Regression coefficients for predictors, moderators, and interactions. [file Table_2.docx]

Supplementary File 2

Moderators of peer influence effects for adolescents’ smoking and vaping norms and outcomes in high and middle-income settings.

**Jennifer M. Murray*, Sharon C. Sánchez-Franco, Olga L. Sarmiento, Erik O. Kimbrough, Christopher Tate, Shannon C. Montgomery, Rajnish Kumar, Laura Dunne, Abhijit Ramalingam, Erin L. Krupka, Felipe Montes, Huiyu Zhou, Laurence Moore, Linda Bauld, Blanca Llorente, Frank Kee, Ruth F. Hunter***

*** Correspondence:** Corresponding Authors: [jmurray39@qub.ac.uk](mailto:jmurray39@qub.ac.uk), [ruth.hunter@qub.ac.uk](mailto:ruth.hunter@qub.ac.uk)

**This file includes:**

Supplementary Tables S2.1. to S2.23. (regression coefficients for predictors, moderators, and interactions).

**
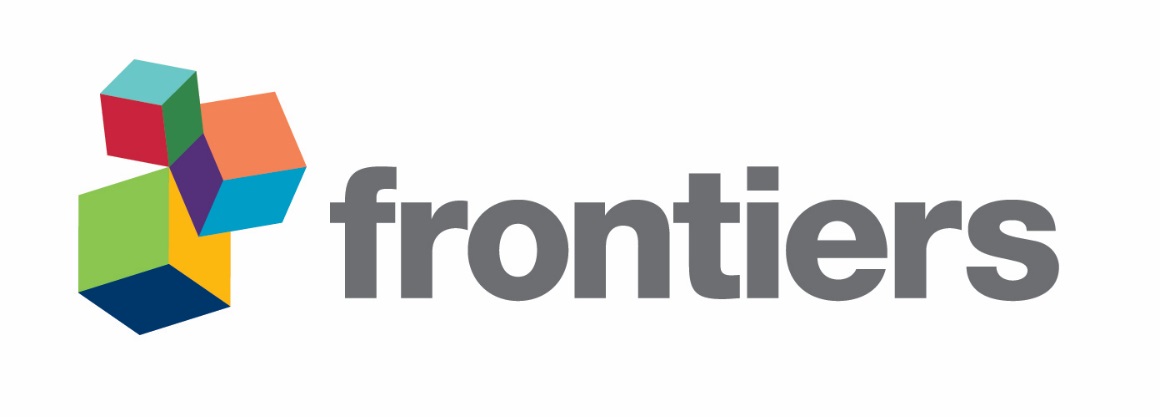
**

**Supplementary file 2: Regression coefficients for predictors, moderators, and interactions.**

**Table S2.1.** Results of ordinary least squares linear regressions including interaction terms examining differences in peer influence effects according to setting (participants in Bogotá compared to participants in Northern Ireland) for outcomes collected at follow-up.

|  | **Dependent variable: Participant responses to the outcome variable at follow-up^a^** | | | | | | | | | | | | | | | | | | | |
| --- | --- | --- | --- | --- | --- | --- | --- | --- | --- | --- | --- | --- | --- | --- | --- | --- | --- | --- | --- | --- |
|  | **(1) -i=Average of nominated friends** | | | | | | | n | **(2) -i=Average of school class** | | | | | | **(3) -i=Average of school year group** | | | | | |
| **IV** | n | **Ave_-i,t_^b^** | | **M^c^** | | **M*Ave_-i,t_^d^** | |  | **Ave_-i,t_^b^** | | **M^c^** | | **M*Ave_-i,t_^d^** | | **Ave_-i,t_^b^** | | **M^c^** | | **M*Ave_-i,t_^d^** | |
| **DV** |  | *b* (SE) | p-value | *b* (SE) | p-value | *b* (SE) | p-value |  | *b* (SE) | p-value | *b* (SE) | p-value | *b* (SE) | p-value | *b* (SE) | p-value | *b* (SE) | p-value | *b* (SE) | p-value |
| **P2S2** | | | | | | | | | | | | | | | | | | | | |
| *t*=base**^i^** | 1073 | 0.22 (0.14) | 0.10 | -0.06 (0.02) | 0.004 | 0.09 (0.17) | 0.59 | 1087 | 0.16 (0.19) | 0.38 | -0.06 (0.02) | 0.02 | 0.21 (0.26) | 0.43 | 0.40 (0.53) | 0.45 | -0.01 (0.04) | 0.72 | 0.57 (0.65) | 0.38 |
| *t*=fu**^n^** | 1018 | -0.004 (0.10) | 0.96 | -0.07 (0.02) | 0.001 | 0.16 (0.13) | 0.23 | **1087** | **-0.48 (0.25)** | **0.05** | **-0.09 (0.03)** | **0.002** | **0.95 (0.28)** | **0.001** | 0.18 (0.47) | 0.70 | -0.09 (0.04) | 0.02 | -0.47 (0.55) | 0.40 |
| **P2S3** | | | | | | | | | | | | | | | | | | | | |
| *t*=base | 1072 | 0.23 (0.11) | 0.03 | 0.02 (0.02) | 0.42 | -0.18 (0.14) | 0.21 | 1086 | 0.11 (0.21) | 0.59 | 0.02 (0.02) | 0.32 | 0.16 (0.34) | 0.65 | 0.11 (0.47) | 0.82 | 0.02 (0.03) | 0.48 | -0.08 (1.10) | 0.94 |
| *t*=fu | 1017 | 0.17 (0.09) | 0.07 | 0.02 (0.02) | 0.29 | -0.09 (0.13) | 0.48 | 1086 | 0.13 (0.18) | 0.48 | 0.02 (0.02) | 0.37 | 0.18 (0.26) | 0.51 | 0.47 (0.30) | 0.11 | 0.01 (0.02) | 0.53 | -1.07 (0.63) | 0.09 |
| **P2S4** | | | | | | | | | | | | | | | | | | | | |
| *t*=base | 1070 | 0.21 (0.13) | 0.11 | 0.07 (0.02) | 0.001 | -0.13 (0.16) | 0.40 | 1084 | 0.17 (0.14) | 0.21 | 0.07 (0.02) | 0.001 | 0.02 (0.26) | 0.93 | **0.21 (0.33)** | **0.51** | **0.07 (0.02)** | **0.001** | **2.02 (0.66)** | **0.002** |
| *t*=fu | 1016 | 0.02 (0.08) | 0.81 | 0.06 (0.02) | 0.006 | 0.11 (0.12) | 0.38 | 1084 | 0.16 (0.15) | 0.29 | 0.06 (0.02) | 0.01 | 0.05 (0.22) | 0.83 | -0.33 (0.41) | 0.42 | 0.08 (0.03) | 0.02 | 0.51 (0.40) | 0.20 |
| **P2S5** | | | | | | | | | | | | | | | | | | | | |
| *t*=base | 1073 | 0.09 (0.10) | 0.33 | -0.008 (0.03) | 0.77 | 0.16 (0.13) | 0.21 | **1087** | **-0.01 (0.16)** | **0.95** | **0.007 (0.03)** | **0.82** | **0.64 (0.23)** | **0.005** | **-0.19 (0.37)** | **0.61** | **0.01 (0.04)** | **0.70** | **1.18 (0.46)** | **0.01** |
| *t*=fu**^i^** | 1018 | -0.01 (0.09) | 0.88 | -0.01 (0.03) | 0.73 | 0.28 (0.12) | 0.02 | **1087** | **-0.16 (0.18)** | **0.38** | **-0.001 (0.03)** | **0.97** | **0.80 (0.21)** | **<0.001** | -0.008 (0.35) | 0.98 | 0.005 (0.03) | 0.87 | 0.64 (0.39) | 0.11 |
| **P2S6** | | | | | | | | | | | | | | | | | | | | |
| *t*=base | 1070 | 0.23 (0.09) | 0.01 | 0.01 (0.03) | 0.71 | -0.11 (0.12) | 0.37 | 1084 | 0.33 (0.14) | 0.02 | 0.005 (0.03) | 0.86 | 0.07 (0.20) | 0.71 | 0.69 (0.24) | 0.004 | -0.002 (0.03) | 0.95 | -0.51 (0.31) | 0.10 |
| *t*=fu | 1015 | 0.29 (0.09) | 0.001 | 0.02 (0.03) | 0.45 | -0.23 (0.12) | 0.06 | 1084 | 0.48 (0.14) | <0.001 | 0.009 (0.03) | 0.74 | -0.07 (0.20) | 0.73 | 0.72 (0.20) | <0.001 | 0.004 (0.03) | 0.89 | -0.56 (0.39) | 0.15 |
| **P2S7** | | | | | | | | | | | | | | | | | | | | |
| *t*=base | 1072 | 0.17 (0.07) | 0.02 | -0.02 (0.03) | 0.46 | -0.02 (0.10) | 0.84 | 1086 | 0.37 (0.11) | 0.001 | -0.01 (0.03) | 0.65 | -0.14 (0.17) | 0.40 | 0.43 (0.18) | 0.02 | -0.02 (0.03) | 0.50 | -0.37 (0.31) | 0.24 |
| *t*=fu | 1018 | 0.26 (0.08) | 0.001 | -0.006 (0.03) | 0.84 | 0.02 (0.11) | 0.86 | 1086 | 0.46 (0.12) | <0.001 | -0.007 (0.03) | 0.79 | -0.10 (0.18) | 0.57 | 0.45 (0.20) | 0.03 | -0.02 (0.03) | 0.52 | -0.39 (0.38) | 0.31 |
| **P2S8** | | | | | | | | | | | | | | | | | | | | |
| *t*=base | 1073 | 0.20 (0.08) | 0.02 | -0.005 (0.03) | 0.84 | 0.18 (0.11) | 0.11 | 1087 | 0.16 (0.13) | 0.20 | -0.01 (0.03) | 0.68 | 0.36 (0.18) | 0.05 | 0.19 (0.22) | 0.40 | -0.04 (0.03) | 0.16 | 0.94 (0.42) | 0.03 |
| *t*=fu | 1018 | 0.14 (0.09) | 0.13 | 0.02 (0.03) | 0.50 | 0.14 (0.12) | 0.23 | 1087 | 0.32 (0.13) | 0.01 | 0.004 (0.02) | 0.86 | 0.25 (0.16) | 0.12 | 0.49 (0.19) | 0.01 | -0.003 (0.03) | 0.92 | -0.006 (0.28) | 0.98 |
| **P2S9** | | | | | | | | | | | | | | | | | | | | |
| *t*=base | 1072 | 0.14 (0.10) | 0.16 | -0.01 (0.02) | 0.67 | 0.02 (0.14) | 0.88 | 1086 | 0.36 (0.21) | 0.10 | 0.005 (0.02) | 0.82 | 0.28 (0.29) | 0.34 | 0.38 (0.38) | 0.31 | 0.004 (0.02) | 0.86 | 0.40 (0.62) | 0.52 |
| *t*=fu | 1017 | 0.18 (0.10) | 0.06 | -0.005 (0.02) | 0.85 | 0.09 (0.13) | 0.49 | 1086 | 0.28 (0.16) | 0.08 | -0.002 (0.02) | 0.94 | 0.21 (0.22) | 0.35 | -0.04 (0.39) | 0.91 | -0.01 (0.02) | 0.69 | 0.37 (0.50) | 0.46 |
| **Experiment Part 2: Injunctive norms (Average P2S2 to P2S9)** | | | | | | | | | | | | | | | | | | | | |
| *t*=base | 1064 | 0.21 (0.08) | 0.005 | 0.008 (0.01) | 0.56 | 0.03 (0.11) | 0.76 | 1078 | 0.20 (0.13) | 0.12 | 0.01 (0.01) | 0.37 | 0.37 (0.21) | 0.09 | 0.27 (0.19) | 0.16 | 0.01 (0.02) | 0.38 | 0.49 (0.44) | 0.27 |
| *t*=fu | 1011 | 0.17 (0.08) | 0.03 | 0.01 (0.01) | 0.42 | 0.15 (0.11) | 0.17 | 1078 | 0.32 (0.12) | 0.008 | 0.01 (0.01) | 0.35 | 0.24 (0.17) | 0.16 | 0.35 (0.20) | 0.08 | 0.008 (0.02) | 0.58 | -0.06 (0.34) | 0.85 |
| **P3Q1** | | | | | | | | | | | | | | | | | | | | |
| *t*=base | 1073 | 0.17 (0.09) | 0.05 | 0.05 (0.03) | 0.17 | -0.13 (0.12) | 0.31 | 1087 | 0.33 (0.13) | 0.009 | 0.06 (0.03) | 0.09 | -0.16 (0.23) | 0.48 | 0.17 (0.19) | 0.37 | 0.06 (0.03) | 0.10 | 0.09 (0.29) | 0.76 |
| *t*=fu | 1018 | 0.11 (0.09) | 0.21 | 0.04 (0.04) | 0.30 | -0.03 (0.12) | 0.79 | 1087 | 0.35 (0.13) | 0.007 | 0.04 (0.03) | 0.21 | -0.13 (0.20) | 0.52 | 0.20 (0.24) | 0.40 | 0.04 (0.03) | 0.21 | -0.04 (0.32) | 0.91 |
| **P3Q2** | | | | | | | | | | | | | | | | | | | | |
| *t*=base | 1073 | 0.16 (0.08) | 0.05 | 0.01 (0.04) | 0.74 | 0.07 (0.11) | 0.53 | 1087 | 0.28 (0.11) | 0.01 | 0.02 (0.04) | 0.54 | 0.10 (0.16) | 0.55 | 0.22 (0.15) | 0.15 | 0.02 (0.04) | 0.55 | 0.25 (0.21) | 0.24 |
| *t*=fu | 1018 | 0.15 (0.09) | 0.08 | -0.02 (0.04) | 0.58 | 0.05 (0.12) | 0.65 | 1087 | 0.20 (0.12) | 0.11 | 0.009 (0.04) | 0.81 | 0.20 (0.17) | 0.24 | 0.15 (0.20) | 0.44 | 0.005 (0.04) | 0.88 | 0.21 (0.25) | 0.41 |
| **Experiment Part 3: Descriptive norms (Average P3Q1 to P3Q2)** | | | | | | | | | | | | | | | | | | | | |
| *t*=base | 1073 | 0.15 (0.08) | 0.06 | 0.03 (0.03) | 0.29 | 0.007 (0.12) | 0.95 | 1087 | 0.29 (0.11) | 0.01 | 0.04 (0.03) | 0.17 | 0.004 (0.18) | 0.98 | 0.15 (0.16) | 0.34 | 0.04 (0.03) | 0.21 | 0.20 (0.22) | 0.36 |
| *t*=fu | 1018 | 0.11 (0.08) | 0.18 | 0.01 (0.03) | 0.77 | 0.02 (0.11) | 0.83 | 1087 | 0.25 (0.12) | 0.04 | 0.03 (0.03) | 0.35 | 0.07 (0.17) | 0.69 | 0.11 (0.21) | 0.61 | 0.03 (0.03) | 0.42 | 0.14 (0.26) | 0.59 |
| **Donation to ASSIST/Dead Cool** | | | | | | | | | | | | | | | | | | | | |
| *t*=base | 1071 | 0.20 (0.08) | 0.02 | 0.46 (0.18) | 0.009 | -0.16 (0.12) | 0.18 | 1085 | 0.12 (0.12) | 0.32 | 0.45 (0.18) | 0.01 | -0.03 (0.19) | 0.89 | **-0.32 (0.24)** | **0.18** | **0.11 (0.21)** | **0.59** | **2.25 (0.58)** | **<0.001^p^** |
| *t*=fu | 1016 | 0.29 (0.09) | 0.001 | 0.31 (0.18) | 0.09 | 0.06 (0.12) | 0.61 | 1085 | 0.32 (0.15) | 0.03 | 0.22 (0.19) | 0.24 | 0.25 (0.18) | 0.17 | 0.53 (0.23) | 0.02 | 0.14 (0.19) | 0.46 | 0.15 (0.29) | 0.62 |
| **IN1** | | | | | | | | | | | | | | | | | | | | |
| *t*=base | 1073 | -0.13 (0.09) | 0.12 | -0.07 (0.05) | 0.12 | 0.18 (0.13) | 0.17 | 1082 | 0.09 (0.19) | 0.64 | -0.07 (0.05) | 0.11 | -0.04 (0.27) | 0.90 | -0.02 (0.34) | 0.96 | -0.08 (0.05) | 0.08 | 0.75 (0.46) | 0.10 |
| *t*=fu | **1070** | **-0.19 (0.09)** | **0.04** | **-0.07 (0.05)** | **0.12** | **0.36 (0.12)** | **0.003** | 1082 | -0.37 (0.15) | 0.01 | -0.08 (0.05) | 0.09 | 0.33 (0.23) | 0.15 | **-0.84 (0.45)** | **0.06** | **-0.08 (0.05)** | **0.08** | **1.28 (0.52)** | **0.01** |
| **IN2** | | | | | | | | | | | | | | | | | | | | |
| *t*=base | 1075 | -0.06 (0.08) | 0.44 | -0.03 (0.03) | 0.31 | 0.07 (0.13) | 0.58 | 1084 | 0.15 (0.20) | 0.47 | -0.02 (0.03) | 0.43 | 0.05 (0.29) | 0.85 | 0.03 (1.00) | 0.98 | -0.03 (0.03) | 0.41 | -0.004 (0.84) | 1.00 |
| *t*=fu | 1072 | 0.08 (0.11) | 0.48 | -0.03 (0.03) | 0.38 | 0.08 (0.15) | 0.60 | 1084 | 0.35 (0.16) | 0.03 | -0.02 (0.03) | 0.50 | -0.47 (0.22) | 0.04 | -1.02 (0.81) | 0.21 | -0.06 (0.04) | 0.12 | 1.11 (0.80) | 0.16 |
| **IN3** | | | | | | | | | | | | | | | | | | | | |
| *t*=base | 1073 | 0.007 (0.09) | 0.93 | -0.01 (0.04) | 0.74 | 0.14 (0.12) | 0.24 | 1082 | 0.17 (0.20) | 0.38 | -0.0009 (0.05) | 0.99 | -0.002 (0.24) | 0.99 | 0.48 (0.38) | 0.20 | 0.02 (0.09) | 0.84 | -0.46 (0.49) | 0.35 |
| *t*=fu | 1070 | 0.08 (0.09) | 0.40 | -0.03 (0.04) | 0.54 | -0.002 (0.11) | 0.99 | 1082 | 0.07 (0.16) | 0.68 | -0.04 (0.04) | 0.43 | -0.18 (0.21) | 0.39 | 0.11 (0.40) | 0.78 | -0.03 (0.06) | 0.66 | -0.13 (0.45) | 0.78 |
| **IN4** | | | | | | | | | | | | | | | | | | | | |
| *t*=base | 1071 | 0.09 (0.08) | 0.26 | 0.15 (0.05) | 0.004 | -0.10 (0.11) | 0.36 | 1081 | 0.14 (0.17) | 0.43 | 0.15 (0.05) | 0.005 | -0.33 (0.26) | 0.21 | 1.20 (0.42) | 0.004 | 0.16 (0.05) | 0.003 | -0.92 (0.55) | 0.09 |
| *t*=fu | 1069 | 0.10 (0.08) | 0.21 | 0.14 (0.05) | 0.01 | -0.11 (0.11) | 0.33 | 1081 | 0.17 (0.14) | 0.24 | 0.14 (0.06) | 0.01 | -0.16 (0.26) | 0.54 | 0.40 (0.31) | 0.20 | 0.13 (0.06) | 0.03 | -0.44 (0.53) | 0.40 |
| **IN5** | | | | | | | | | | | | | | | | | | | | |
| *t*=base | 1073 | -0.003 (0.08) | 0.97 | 0.10 (0.05) | 0.06 | 0.01 (0.11) | 0.90 | 1082 | 0.11 (0.17) | 0.53 | 0.10 (0.06) | 0.07 | -0.17 (0.22) | 0.44 | 0.33 (0.29) | 0.26 | 0.12 (0.06) | 0.05 | -0.24 (0.50) | 0.63 |
| *t*=fu | 1070 | 0.19 (0.10) | 0.05 | 0.10 (0.05) | 0.07 | -0.27 (0.12) | 0.03 | 1082 | 0.23 (0.14) | 0.09 | 0.10 (0.05) | 0.06 | -0.53 (0.25) | 0.03 | 0.05 (0.28) | 0.85 | 0.10 (0.05) | 0.08 | -0.06 (0.45) | 0.89 |
| **IN6** | | | | | | | | | | | | | | | | | | | | |
| *t*=base | 1074 | 0.10 (0.09) | 0.25 | -0.16 (0.06) | 0.007 | 0.15 (0.11) | 0.20 | 1083 | 0.08 (0.13) | 0.54 | -0.17 (0.06) | 0.005 | 0.12 (0.21) | 0.56 | 0.22 (0.20) | 0.28 | -0.14 (0.06) | 0.03 | 0.23 (0.38) | 0.54 |
| *t*=fu | 1071 | 0.08 (0.10) | 0.40 | -0.16 (0.06) | 0.007 | 0.21 (0.12) | 0.09 | 1083 | 0.12 (0.14) | 0.40 | -0.15 (0.06) | 0.01 | 0.12 (0.19) | 0.54 | 0.03 (0.28) | 0.90 | -0.16 (0.07) | 0.03 | 0.24 (0.37) | 0.53 |
| **IN7** | | | | | | | | | | | | | | | | | | | | |
| *t*=base | 1075 | 0.15 (0.09) | 0.11 | -0.08 (0.06) | 0.19 | -0.09 (0.12) | 0.47 | 1084 | -0.02 (0.18) | 0.90 | -0.09 (0.06) | 0.11 | 0.26 (0.24) | 0.28 | 0.20 (0.34) | 0.56 | -0.01 (0.07) | 0.86 | 0.63 (0.44) | 0.15 |
| *t*=fu | 1072 | 0.16 (0.13) | 0.20 | -0.09 (0.06) | 0.13 | 0.01 (0.14) | 0.92 | 1084 | -0.009 (0.17) | 0.96 | -0.10 (0.06) | 0.09 | 0.24 (0.22) | 0.28 | -0.17 (0.35) | 0.62 | -0.11 (0.07) | 0.11 | 0.45 (0.37) | 0.23 |
| **Self-report injunctive norms (Average IN1 to IN7)** | | | | | | | | | | | | | | | | | | | | |
| *t*=base | 1068 | -0.09 (0.09) | 0.29 | -0.02 (0.03) | 0.55 | 0.26 (0.12) | 0.03 | 1078 | 0.009 (0.18) | 0.96 | -0.02 (0.03) | 0.48 | 0.04 (0.23) | 0.87 | 0.24 (0.25) | 0.33 | 0.02 (0.03) | 0.55 | 0.53 (0.37) | 0.15 |
| *t*=fu | 1066 | 0.09 (0.09) | 0.34 | -0.02 (0.03) | 0.57 | 0.15 (0.11) | 0.19 | 1078 | 0.17 (0.13) | 0.20 | -0.01 (0.03) | 0.67 | 0.04 (0.18) | 0.84 | -0.04 (0.26) | 0.88 | -0.02 (0.03) | 0.61 | 0.41 (0.32) | 0.21 |
| **DN1.1** | | | | | | | | | | | | | | | | | | | | |
| *t*=base | 1075 | 0.43 (0.13) | 0.001 | 0.08 (0.05) | 0.08 | -0.32 (0.22) | 0.14 | **1084** | **0.41 (0.17)** | **0.02** | **0.11 (0.04)** | **0.02** | **-0.80 (0.24)** | **0.001** | **1.12 (0.32)** | **0.001** | **0.09 (0.05)** | **0.08** | **-1.55 (0.51)** | **0.003** |
| *t*=fu | 1072 | 0.33 (0.11) | 0.003 | 0.06 (0.05) | 0.19 | -0.09 (0.17) | 0.58 | 1084 | 0.31 (0.15) | 0.04 | 0.11 (0.05) | 0.02 | -0.72 (0.38) | 0.06 | 0.68 (0.20) | 0.001 | 0.07 (0.05) | 0.21 | -0.71 (0.65) | 0.27 |
| **DN1.2** | | | | | | | | | | | | | | | | | | | | |
| *t*=base | 1076 | 0.10 (0.06) | 0.10 | 0.02 (0.05) | 0.73 | 0.07 (0.10) | 0.48 | 1085 | 0.02 (0.08) | 0.78 | 0.02 (0.05) | 0.75 | 0.17 (0.16) | 0.29 | 0.12 (0.11) | 0.24 | -0.02 (0.06) | 0.68 | 0.18 (0.30) | 0.54 |
| *t*=fu | 1073 | -0.05 (0.07) | 0.44 | 0.07 (0.05) | 0.15 | 0.09 (0.08) | 0.30 | 1085 | -0.03 (0.11) | 0.80 | 0.07 (0.05) | 0.14 | 0.02 (0.17) | 0.92 | 0.07 (0.13) | 0.59 | 0.06 (0.05) | 0.30 | -0.09 (0.36) | 0.80 |
| **DN1.3** | | | | | | | | | | | | | | | | | | | | |
| *t*=base | 1075 | 0.13 (0.07) | 0.06 | 0.03 (0.06) | 0.55 | -0.09 (0.09) | 0.29 | 1084 | 0.09 (0.10) | 0.38 | 0.05 (0.06) | 0.42 | -0.16 (0.17) | 0.36 | 0.18 (0.15) | 0.24 | 0.04 (0.07) | 0.61 | -0.24 (0.31) | 0.45 |
| *t*=fu | 1072 | 0.03 (0.08) | 0.66 | 0.05 (0.06) | 0.42 | -0.04 (0.09) | 0.70 | 1084 | -0.005 (0.10) | 1.00 | 0.04 (0.06) | 0.46 | 0.01 (0.18) | 0.94 | **0.25 (0.18)** | **0.18** | **0.14 (0.08)** | **0.09** | **-1.08 (0.44)** | **0.01** |
| **DN1.4** | | | | | | | | | | | | | | | | | | | | |
| *t*=base | 1075 | 0.06 (0.08) | 0.42 | 0.02 (0.04) | 0.62 | -0.07 (0.09) | 0.43 | 1084 | 0.25 (0.19) | 0.19 | 0.02 (0.04) | 0.70 | -0.37 (0.28) | 0.18 | 0.13 (0.21) | 0.52 | 0.01 (0.04) | 0.76 | -0.03 (0.33) | 0.92 |
| *t*=fu | 1072 | -0.07 (0.07) | 0.29 | 0.01 (0.04) | 0.80 | -0.02 (0.09) | 0.79 | 1084 | 0.30 (0.22) | 0.18 | 0.02 (0.04) | 0.69 | -0.49 (0.27) | 0.07 | 0.02 (0.28) | 0.93 | 0.009 (0.04) | 0.83 | -0.21 (0.37) | 0.57 |
| **DN1.5** | | | | | | | | | | | | | | | | | | | | |
| *t*=base | 1075 | 0.13 (0.11) | 0.22 | -0.01 (0.04) | 0.76 | -0.09 (0.12) | 0.46 | 1084 | 0.33 (0.18) | 0.07 | -0.01 (0.04) | 0.69 | -0.39 (0.22) | 0.08 | 0.002 (0.26) | 0.99 | -0.02 (0.04) | 0.62 | 0.67 (0.50) | 0.18 |
| *t*=fu | 1072 | 0.03 (0.07) | 0.73 | -0.02 (0.04) | 0.68 | -0.07 (0.10) | 0.49 | 1084 | 0.31 (0.15) | 0.04 | -0.02 (0.03) | 0.65 | -0.36 (020) | 0.08 | 0.09 (0.22) | 0.66 | -0.02 (0.04) | 0.64 | -0.0003 (0.33) | 1.00 |
| **Self-report descriptive norms 1 (Average DN1.1 to DN1.5)** | | | | | | | | | | | | | | | | | | | | |
| *t*=base | 1073 | 0.12 (0.08) | 0.12 | 0.02 (0.03) | 0.42 | -0.06 (0.10) | 0.54 | 1082 | 0.08 (0.09) | 0.38 | 0.03 (0.03) | 0.30 | -0.15 (0.16) | 0.35 | 0.23 (0.12) | 0.05 | 0.01 (0.03) | 0.66 | -0.20 (0.21) | 0.35 |
| *t*=fu | 1070 | 0.06 (0.06) | 0.35 | 0.03 (0.02) | 0.29 | -0.07 (0.09) | 0.46 | 1082 | 0.03 (0.09) | 0.74 | 0.03 (0.03) | 0.21 | -0.14 (0.18) | 0.44 | 0.23 (0.12) | 0.06 | 0.02 (0.03) | 0.35 | -0.38 (0.27) | 0.15 |
| **DN2.1** | | | | | | | | | | | | | | | | | | | | |
| *t*=base | 1076 | 0.24 (0.10) | 0.01 | 0.07 (0.04) | 0.12 | -0.18 (0.14) | 0.23 | 1085 | 0.12 (0.13) | 0.38 | 0.06 (0.04) | 0.17 | 0.11 (0.23) | 0.64 | 0.25 (0.17) | 0.15 | 0.07 (0.04) | 0.12 | -0.25 (0.34) | 0.47 |
| *t*=fu | 1073 | 0.16 (0.09) | 0.07 | 0.07 (0.04) | 0.09 | 0.03 (0.13) | 0.82 | 1085 | 0.15 (0.14) | 0.28 | 0.07 (0.04) | 0.11 | 0.04 (0.23) | 0.85 | **0.25 (0.17)** | **0.14** | **0.10 (0.04)** | **0.02** | **-1.09 (0.41)** | **0.008** |
| **DN2.2** | | | | | | | | | | | | | | | | | | | | |
| *t*=base | 1076 | 0.09 (0.10) | 0.41 | 0.11 (0.06) | 0.06 | 0.10 (0.13) | 0.46 | 1085 | 0.12 (0.14) | 0.38 | 0.07 (0.07) | 0.32 | 0.31 (0.24) | 0.19 | 0.59 (0.28) | 0.03 | 0.04 (0.07) | 0.59 | -0.35 (0.38) | 0.36 |
| *t*=fu | 1073 | 0.12 (0.10) | 0.25 | 0.12 (0.06) | 0.05 | -0.06 (0.12) | 0.65 | 1085 | -0.23 (0.17) | 0.16 | 0.13 (0.06) | 0.05 | 0.55 (0.24) | 0.02 | 0.31 (0.28) | 0.27 | 0.07 (0.07) | 0.37 | -0.08 (0.43) | 0.85 |
| **DN2.3** | | | | | | | | | | | | | | | | | | | | |
| *t*=base | 1076 | 0.32 (0.10) | 0.001 | 0.07 (0.04) | 0.10 | -0.14 (0.14) | 0.30 | 1085 | 0.59 (0.13) | <0.001 | 0.03 (0.05) | 0.52 | -0.10 (0.19) | 0.60 | 0.67 (0.16) | <0.001 | 0.03 (0.05) | 0.55 | -0.16 (0.28) | 0.58 |
| *t*=fu | 1073 | 0.19 (0.06) | 0.002 | 0.08 (0.05) | 0.07 | -0.07 (0.13) | 0.58 | 1085 | 0.32 (0.12) | 0.006 | 0.05 (0.05) | 0.32 | 0.11 (0.17) | 0.53 | 0.52 (0.14) | <0.001 | 0.03 (0.05) | 0.47 | -0.05 (0.25) | 0.83 |
| **Self-report descriptive norms 2 (Average DN2.1 to DN2.3)** | | | | | | | | | | | | | | | | | | | | |
| *t*=base | 1076 | 0.18 (0.10) | 0.09 | 0.06 (0.03) | 0.07 | 0.03 (0.14) | 0.80 | 1085 | 0.34 (0.13) | 0.009 | 0.04 (0.04) | 0.32 | -0.02 (0.23) | 0.93 | 0.53 (0.17) | 0.001 | 0.08 (0.04) | 0.07 | -0.79 (0.40) | 0.05 |
| *t*=fu | 1073 | 0.09 (0.09) | 0.33 | 0.08 (0.03) | 0.02 | -0.01 (0.12) | 0.91 | 1085 | 0.18 (0.13) | 0.15 | 0.06 (0.03) | 0.10 | 0.06 (0.18) | 0.76 | **0.46 (0.15)** | **0.002** | **0.16 (0.05)** | **0.001** | **-1.68 (0.53)** | **0.002** |
| **Self-report smoking behavior** | | | | | | | | | | | | | | | | | | | | |
| *t*=base | 1083 | 0.23 (0.12) | 0.05 | -0.01 (0.04) | 0.69 | -0.14 (0.14) | 0.33 | 1093 | 0.17 (0.16) | 0.31 | 0.005 (0.04) | 0.89 | 0.11 (0.20) | 0.59 | 0.73 (0.27) | 0.007 | 0.05 (0.04) | 0.19 | -0.15 (0.31) | 0.62 |
| *t*=fu | 1081 | 0.33 (0.12) | 0.007 | 0.01 (0.04) | 0.80 | -0.14 (0.14) | 0.32 | 1093 | 0.24 (0.14) | 0.08 | 0.01 (0.04) | 0.77 | -0.04 (0.18) | 0.84 | 0.53 (0.19) | 0.006 | 0.04 (0.04) | 0.33 | -0.18 (0.23) | 0.44 |
| **Intentions** | | | | | | | | | | | | | | | | | | | | |
| *t*=base | 1078 | 0.25 (0.11) | 0.03 | -0.12 (0.07) | 0.12 | -0.15 (0.16) | 0.36 | 1087 | 0.50 (0.22) | 0.02 | -0.07 (0.08) | 0.35 | -0.10 (0.30) | 0.74 | 1.02 (0.41) | 0.01 | 0.01 (0.10) | 0.89 | -0.07 (0.71) | 0.92 |
| *t*=fu | 1075 | 0.41 (0.16) | 0.01 | -0.08 (0.08) | 0.31 | -0.30 (0.18) | 0.10 | 1087 | 0.21 (0.17) | 0.23 | -0.13 (0.08) | 0.10 | -0.33 (0.24) | 0.16 | 0.56 (0.27) | 0.04 | -0.06 (0.10) | 0.57 | -0.52 (0.38) | 0.17 |
| **Knowledge** | | | | | | | | | | | | | | | | | | | | |
| *t*=base**^i^** | 1080 | 0.15 (0.09) | 0.10 | -0.29 (0.11) | 0.006 | -0.05 (0.12) | 0.66 | 1089 | 0.43 (0.12) | 0.001 | -0.12 (0.12) | 0.33 | -0.18 (0.20) | 0.38 | 0.15 (0.27) | 0.57 | -0.07 (0.17) | 0.69 | 0.50 (0.40) | 0.21 |
| *t*=fu**^k^** | 1077 | 0.37 (0.09) | <0.001 | -0.18 (0.10) | 0.09 | -0.25 (0.11) | 0.03 | 1089 | 0.47 (0.11) | <0.001 | -0.14 (0.12) | 0.22 | -0.35 (0.18) | 0.05 | -0.81 (0.48) | 0.09 | -0.65 (0.25) | 0.009 | 1.00 (0.44) | 0.02 |
| **Attitudes** | | | | | | | | | | | | | | | | | | | | |
| *t*=base | 1067 | 0.18 (0.09) | 0.04 | -0.007 (0.04) | 0.87 | -0.02 (0.11) | 0.85 | 1076 | 0.11 (0.15) | 0.46 | -0.003 (0.04) | 0.94 | -0.30 (0.20) | 0.14 | 0.48 (0.24) | 0.04 | 0.006 (0.04) | 0.89 | -0.59 (0.46) | 0.20 |
| *t*=fu | 1064 | 0.20 (0.11) | 0.06 | 0.007 (0.04) | 0.87 | -0.04 (0.14) | 0.75 | 1076 | 0.08 (0.15) | 0.58 | -0.01 (0.04) | 0.79 | -0.53 (0.24) | 0.03 | 0.39 (0.19) | 0.04 | -0.006 (0.05) | 0.90 | -0.98 (0.46) | 0.03 |
| **Self-efficacy (Emotional)** | | | | | | | | | | | | | | | | | | | | |
| *t*=base | 1072 | 0.12 (0.10) | 0.22 | -0.19 (0.05) | 0.001 | -0.04 (0.14) | 0.76 | 1081 | 0.40 (0.15) | 0.007 | -0.17 (0.06) | 0.002 | -0.33 (0.23) | 0.16 | 0.64 (0.28) | 0.03 | -0.16 (0.06) | 0.01 | -0.59 (0.35) | 0.10 |
| *t*=fu | 1068 | 0.38 (0.13) | 0.004 | -0.13 (0.06) | 0.03 | -0.19 (0.15) | 0.21 | 1081 | 0.10 (0.12) | 0.43 | -0.18 (0.06) | 0.003 | -0.07 (0.19) | 0.70 | 0.34 (0.21) | 0.11 | -0.17 (0.08) | 0.02 | -0.55 (0.35) | 0.12 |
| **Self-efficacy (Friends)** | | | | | | | | | | | | | | | | | | | | |
| *t*=base | 1078 | 0.02 (0.08) | 0.79 | -0.13 (0.05) | 0.01 | 0.07 (0.12) | 0.59 | 1087 | 0.11 (0.12) | 0.37 | -0.12 (0.05) | 0.01 | 0.11 (0.22) | 0.60 | 0.08 (0.20) | 0.68 | -0.13 (0.05) | 0.01 | -0.02 (0.30) | 0.94 |
| *t*=fu | 1075 | 0.16 (0.12) | 0.19 | -0.11 (0.05) | 0.03 | 0.04 (0.14) | 0.79 | 1087 | -0.07 (0.12) | 0.57 | -0.13 (0.05) | 0.009 | 0.14 (0.19) | 0.45 | -0.03 (0.25) | 0.89 | -0.14 (0.06) | 0.02 | 0.02 (0.36) | 0.96 |
| **Self-efficacy (Opportunity)** | | | | | | | | | | | | | | | | | | | | |
| *t*=base | 1079 | 0.11 (0.09) | 0.25 | -0.11 (0.05) | 0.02 | 0.01 (0.14) | 0.94 | 1088 | 0.20 (0.15) | 0.17 | -0.10 (0.05) | 0.03 | 0.23 (0.27) | 0.38 | 0.11 (0.27) | 0.68 | -0.11 (0.05) | 0.04 | 0.18 (0.40) | 0.65 |
| *t*=fu | 1076 | 0.06 (0.10) | 0.56 | -0.11 (0.05) | 0.02 | 0.13 (0.13) | 0.31 | 1088 | 0.09 (0.13) | 0.47 | -0.11 (0.05) | 0.03 | 0.02 (0.18) | 0.93 | 0.006 (0.26) | 0.98 | -0.17 (0.06) | 0.008 | -0.48 (0.33) | 0.14 |
| **Perceived physical risks** | | | | | | | | | | | | | | | | | | | | |
| *t*=base | 1074 | 0.07 (0.09) | 0.43 | -2.12 (1.34) | 0.11 | -0.007 (0.11) | 0.95 | 1082 | 0.06 (0.12) | 0.65 | -1.87 (1.33) | 0.16 | 0.12 (0.17) | 0.47 | 0.06 (0.16) | 0.73 | -2.15 (1.38) | 0.12 | -0.15 (0.32) | 0.64 |
| *t*=fu | 1070 | 0.08 (0.08) | 0.30 | -1.92 (1.33) | 0.15 | 0.04 (0.11) | 0.70 | 1082 | -0.005 (0.15) | 0.97 | -2.01 (1.33) | 0.13 | 0.13 (0.19) | 0.50 | **-0.31 (0.24)** | **0.20** | **-4.55 (1.60)** | **0.005** | **-0.97 (0.38)** | **0.01** |
| **Perceived social risks** | | | | | | | | | | | | | | | | | | | | |
| *t*=base | 1076 | -0.02 (0.09) | 0.86 | -6.49 (1.78) | <0.001 | 0.13 (0.11) | 0.27 | **1085** | **-0.15 (0.13)** | **0.22** | **-6.49 (1.86)** | **0.001** | **0.43 (0.17)** | **0.01** | -0.03 (0.22) | 0.89 | -4.10 (3.10) | 0.19 | 0.49 (0.41) | 0.23 |
| *t*=fu**^i^** | 1073 | 0.13 (0.10) | 0.16 | -6.02 (1.77) | 0.001 | -0.11 (0.12) | 0.38 | 1085 | -0.14 (0.17) | 0.41 | -7.55 (2.05) | <0.001 | 0.19 (0.21) | 0.35 | -0.40 (0.34) | 0.24 | -10.82 (4.02) | 0.007 | 0.19 (0.37) | 0.60 |
| **Perceived addiction risks** | | | | | | | | | | | | | | | | | | | | |
| *t*=base**^j^** | 1016 | 0.15 (0.10) | 0.15 | -9.99 (2.10) | <0.001 | -0.16 (0.13) | 0.24 | 1025 | 0.23 (0.18) | 0.22 | -7.87 (2.69) | 0.004 | -0.04 (0.27) | 0.88 | 0.58 (0.42) | 0.16 | 0.30 (4.68) | 0.95 | 0.30 (0.56) | 0.59 |
| *t*=fu**^i^** | 1009 | -0.02 (0.08) | 0.82 | -12.08 (2.10) | <0.001 | -0.06 (0.12) | 0.58 | 1025 | 0.08 (0.16) | 0.63 | -11.83 (2.75) | <0.001 | -0.23 (0.24) | 0.33 | 0.26 (0.31) | 0.41 | -6.45 (4.10) | 0.12 | 0.02 (0.41) | 0.95 |
| **Perceived benefits** | | | | | | | | | | | | | | | | | | | | |
| *t*=base | 1029 | -0.02 (0.09) | 0.85 | -0.36 (1.36) | 0.79 | -0.05 (0.12) | 0.67 | 1038 | 0.02 (0.16) | 0.91 | -0.55 (1.36) | 0.69 | 0.14 (0.24) | 0.55 | 0.07 (0.23) | 0.77 | -0.69 (1.36) | 0.61 | 0.78 (0.45) | 0.08 |
| *t*=fu**^o^** | 1025 | -0.0008 (0.09) | 0.99 | -0.51 (1.36) | 0.71 | 0.05 (0.12) | 0.69 | 1038 | -0.17 (0.17) | 0.33 | -0.51 (1.35) | 0.71 | 0.15 (0.25) | 0.54 | -1.30 (0.52) | 0.01 | -0.56 (1.35) | 0.68 | 1.45 (0.62) | 0.02 |
| **Perceived behavioral control (easy to quit)** | | | | | | | | | | | | | | | | | | | | |
| *t*=base**^l^** | 1076 | 0.09 (0.11) | 0.44 | 0.66 (0.13) | <0.001 | -0.09 (0.15) | 0.55 | 1085 | 0.03 (0.17) | 0.87 | 0.63 (0.18) | <0.001 | 0.12 (0.29) | 0.68 | -0.33 (0.58) | 0.56 | 0.61 (0.45) | 0.18 | 0.92 (1.05) | 0.38 |
| *t*=fu**^m^** | 1073 | 0.001 (0.10) | 0.99 | 0.81 (0.12) | <0.001 | -0.18 (0.14) | 0.20 | **1085** | **0.40 (0.17)** | **0.02** | **0.93 (0.19)** | **<0.001** | **-1.27 (0.31)** | **<0.001^p^** | **-3.73 (0.89)** | **<0.001** | **3.33 (0.60)** | **<0.001** | **2.56 (0.95)** | **0.007** |
| **Perceived behavioral control (to avoid smoking)** | | | | | | | | | | | | | | | | | | | | |
| *t*=base | **1080** | **0.27 (0.12)** | **0.02** | **-0.29 (0.08)** | **<0.001** | **-0.37 (0.15)** | **0.01** | 1089 | 0.46 (0.22) | 0.04 | -0.23 (0.09) | 0.007 | -0.42 (0.29) | 0.15 | 0.64 (0.44) | 0.15 | -0.17 (0.11) | 0.12 | -0.25 (0.54) | 0.63 |
| *t*=fu | 1077 | 0.18 (0.11) | 0.10 | -0.28 (0.08) | 0.001 | -0.24 (0.14) | 0.10 | 1089 | -0.21 (0.21) | 0.31 | -0.37 (0.09) | <0.001 | 0.03 (0.29) | 0.92 | -0.03 (0.42) | 0.94 | -0.30 (0.13) | 0.02 | 0.08 (0.49) | 0.88 |
| **Objectively measured smoking behavior** | | | | | | | | | | | | | | | | | | | | |
| *t*=base**^j^** | 1041 | 0.13 (0.07) | 0.05 | 1.07 (0.14) | <0.001 | -0.12 (0.11) | 0.28 | 1048 | 0.06 (0.07) | 0.45 | 1.24 (0.14) | <0.001 | -0.20 (0.12) | 0.08 | **0.86 (0.13)** | **<0.001** | **0.44 (0.21)** | **0.03** | **-0.94 (0.18)** | **<0.001^p^** |
| *t*=fu**^i^** | **1022** | **0.74 (0.10)** | **<0.001** | **0.43 (0.13)** | **0.001** | **-0.43 (0.13)** | **0.001** | 1048 | 0.91 (0.07) | <0.001 | -0.07 (0.12) | 0.55 | -0.08 (0.12) | 0.50 | 0.89 (0.11) | <0.001 | -0.15 (0.15) | 0.31 | 0.04 (0.16) | 0.78 |
| **Smoking susceptibility^e^** | | | | | | | | | | | | | | | | | | | | |
|  | n | **Percent_-i,t_^f^** | | **M^g^** | | **M*Percent_-i,t_^h^** | | n | **Percent_-i,t_^f^** | | **M^g^** | | **M*Percent_-i,t_^h^** | | **Percent_-i,t_^f^** | | **M^g^** | | **M*Percent_-i,t_^h^** | |
|  |  | OR (SE) | p-value | OR (SE) | p-value | OR (SE) | p-value |  | OR (SE) | p-value | OR (SE) | p-value | OR (SE) | p-value | OR (SE) | p-value | OR (SE) | p-value | OR (SE) | p-value |
| *t*=base | 1078 | 1.04 (0.05) | 0.39 | 1.76 (0.30) | 0.001 | 1.12 (0.07) | 0.10 | 1087 | 0.95 (0.10) | 0.63 | 1.91 (0.33) | <0.001 | 1.11 (0.14) | 0.39 | 0.77 (0.19) | 0.29 | 2.10 (0.44) | <0.001 | 1.39 (0.37) | 0.21 |
| *t*=fu | 1075 | 1.14 (0.06) | 0.01 | 1.63 (0.28) | 0.005 | 0.96 (0.06) | 0.53 | 1087 | 1.12 (0.10) | 0.20 | 1.71 (0.33) | 0.005 | 0.91 (0.11) | 0.41 | 1.19 (0.24) | 0.39 | 1.50 (0.39) | 0.12 | 0.94 (0.21) | 0.78 |

IV: Independent variable; DV: Dependent variable; Ave: average of peer group (*i*); M: moderator (Setting: 0=Northern Ireland; 1=Bogotá); Percent: percentage of peer group (*-i*) classified as susceptible to commencing smoking; OR: odds ratio; SE: standard error.

^a^In each model the outcome variable is the focal participant’s (*i*) response to the relevant item at follow-up. The predictor variable is the average of the relevant group’s (*-i*) responses to the equivalent item at baseline (*t*=base) or follow-up (*t*=fu), where *–i*=(1) focal participant's nominated friends; (2) focal participant’s school class; (3) focal participant’s school year group. The moderator, and interaction of the moderator with the predictor variable, were also included as independent variables in all models. All models include robust (Huber White) standard errors specified using Stata’s ‘vce(robust)’ option. The following baseline variables are included as covariates in all models: gender (0=boy; 1=girl/prefer not to say), age (1=12 years or less; 2=13 years; 3=14 years or more), intervention (1=ASSIST; 2=Dead Cool), ethnicity (0=no ethnic minority; 1=ethnic minority), individuals' socio-economic status (NI: 1=NIMDM2017≤296.6; 2=296.6<NIMDM2017≤593.2; 3=NIMDM2017>593.2; Bogotá: 1=Informal settlement/Lowest/Low; 2=Middle-Low/Middle; 3=Middle-High/High), and baseline values of the outcome variable. The predictor variable and baseline values of the outcome variable were mean-centered.

^b^Unstandardized regression coefficients representing the average change in the outcome variable for a one-unit increase in the predictor variable among participants in Northern Ireland schools.

^c^Unstandardized regression coefficients representing the average change in the outcome variable for participants in Bogotá schools compared to participants in Northern Ireland schools among participants who are average on the predictor variable.

^d^Unstandardized regression coefficients representing the average change in the association between the outcome variable and the predictor variable for participants in Bogotá schools compared to participants in Northern Ireland schools.

^e^Logistic regressions were run for models including focal participants' smoking susceptibility as the outcome variable, with robust (Huber White) standard errors specified using Stata’s ‘vce(robust)’ option. The predictor variable is the percentage of the relevant group (*-i*) classified as susceptible to commencing smoking at baseline (*t*=base) or follow-up (*t*=fu), where *–i*=(1) focal participant's nominated friends; (2) focal participant’s school class; (3) focal participant’s school year group. The moderator, and interaction of the moderator with the predictor variable, were also included as independent variables in all models. The following baseline variables are included as covariates in all models: gender (0=boy; 1=girl/prefer not to say), age (1=12 years or less; 2=13 years; 3=14 years or more), intervention (1=ASSIST; 2=Dead Cool), ethnicity (0=no ethnic minority; 1=ethnic minority), individuals' socio-economic status (NI: 1=NIMDM2017≤296.6; 2=296.6<NIMDM2017≤593.2; 3=NIMDM2017>593.2; Bogotá: 1=Informal settlement/Lowest/Low; 2=Middle-Low/Middle; 3=Middle-High/High), and baseline values of the outcome variable. Results are odds ratios, standard errors, and p-values. The predictor variable was mean-centered.

^f^Odds ratios representing the multiplicative change in odds of being susceptible to commencing smoking for a 10% increase in the number of nominated friends/pupils in the same school class/pupils in the same school year group classified as being susceptible to commencing smoking (1 out of 10 nominated friends/pupils in the same school class/pupils in the same school year group; predictor variable) among participants in Northern Ireland schools.

^g^Odds ratios representing the multiplicative change in odds of being susceptible to commencing smoking for participants in Bogotá schools compared to participants in Northern Ireland schools among participants who are average on the predictor variable.

^h^Ratio of ratios representing the ratio of: (1) the odds ratio representing the multiplicative change in odds of being susceptible to commencing smoking for a 10% increase in the number of nominated friends/pupils in the same school class/pupils in the same school year group classified as being susceptible to commencing smoking among participants in Bogotá schools; to (2) the odds ratio representing the multiplicative change in odds of being susceptible to commencing smoking for a 10% increase in the number of nominated friends/pupils in the same school class/pupils in the same school year group classified as being susceptible to commencing smoking among participants in Northern Ireland schools.

^i^At least one IV has variance inflation factor (vif)>10 for the model examining school year group average.

^j^At least one IV has vif>20 for the model examining school year group average.

^k^At least one IV has vif>30 for the model examining school year group average.

^l^At least one IV has vif>50 for the model examining school year group average.

^m^At least one IV has vif>100 for the model examining school year group average. Potentially problematic levels of multi-collinearity.

^n^For the model examining school class average, vif=4.32 for predictor, vif=2.10 for setting, vif=3.23 for interaction. Predictor coefficient is positive and significant without including setting or interaction.

^o^For the model examining school class average, vif=5.82 for predictor, vif=1.33 for setting, vif=6.11 for interaction. Predictor coefficient is non-significant without including setting or interaction.

^p^Retained statistical significance at the 5% level after using the Holm-Bonferroni procedure to correct the p-values for multiple testing (p≤0.05; based on 276 tests of interaction effects reported in Table S2.1).

**Table S2.2.** Results of ordinary least squares linear regressions including interaction terms examining differences in peer influence effects according to intervention (Dead Cool schools compared to ASSIST schools) for outcomes collected at follow-up.

|  | **Dependent variable: Participant responses to the outcome variable at follow-up^a^** | | | | | | | | | | | | | | | | | | | |
| --- | --- | --- | --- | --- | --- | --- | --- | --- | --- | --- | --- | --- | --- | --- | --- | --- | --- | --- | --- | --- |
|  | **(1) -i=Average of nominated friends** | | | | | | | n | **(2) -i=Average of school class** | | | | | | **(3) -i=Average of school year group** | | | | | |
| **IV** | n | **Ave_-i,t_^b^** | | **M^c^** | | **M*Ave_-i,t_^d^** | |  | **Ave_-i,t_^b^** | | **M^c^** | | **M*Ave_-i,t_^d^** | | **Ave_-i,t_^b^** | | **M^c^** | | **M*Ave_-i,t_^d^** | |
| **DV** |  | *b* (SE) | p-value | *b* (SE) | p-value | *b* (SE) | p-value |  | *b* (SE) | p-value | *b* (SE) | p-value | *b* (SE) | p-value | *b* (SE) | p-value | *b* (SE) | p-value | *b* (SE) | p-value |
| **P2S2** | | | | | | | | | | | | | | | | | | | | |
| *t*=base | 1073 | 0.27 (0.12) | 0.02 | -0.03 (0.02) | 0.05 | 0.19 (0.15) | 0.21 | 1087 | 0.48 (0.15) | 0.001 | -0.04 (0.02) | 0.03 | -0.13 (0.21) | 0.52 | 0.91 (0.25) | <0.001 | -0.05 (0.02) | 0.01 | -0.31 (0.30) | 0.30 |
| *t*=fu | 1018 | 0.13 (0.08) | 0.12 | -0.02 (0.02) | 0.25 | 0.05 (0.11) | 0.64 | 1087 | 0.28 (0.15) | 0.06 | -0.03 (0.02) | 0.14 | 0.07 (0.19) | 0.72 | 0.64 (0.22) | 0.005 | -0.03 (0.02) | 0.15 | -0.20 (0.28) | 0.49 |
| **P2S3** | | | | | | | | | | | | | | | | | | | | |
| *t*=base | 1072 | 0.09 (0.09) | 0.32 | 0.002 (0.02) | 0.92 | 0.05 (0.14) | 0.72 | 1086 | -0.13 (0.19) | 0.49 | 0.009 (0.02) | 0.69 | 0.65 (0.32) | 0.04 | -0.21 (0.34) | 0.53 | 0.008 (0.02) | 0.73 | 0.73 (0.62) | 0.24 |
| *t*=fu | 1017 | 0.07 (0.09) | 0.40 | 0.006 (0.02) | 0.78 | 0.10 (0.13) | 0.46 | 1086 | 0.11 (0.19) | 0.56 | 0.003 (0.02) | 0.88 | 0.20 (0.27) | 0.45 | 0.31 (0.29) | 0.29 | -0.001 (0.02) | 0.95 | -0.55 (0.60) | 0.36 |
| **P2S4** | | | | | | | | | | | | | | | | | | | | |
| *t*=base | 1070 | 0.10 (0.10) | 0.30 | -0.04 (0.02) | 0.06 | 0.11 (0.16) | 0.50 | 1084 | 0.14 (0.14) | 0.32 | -0.04 (0.02) | 0.10 | 0.16 (0.29) | 0.58 | **-0.43 (0.45)** | **0.33** | **-0.01 (0.03)** | **0.63** | **1.95 (0.66)** | **0.003** |
| *t*=fu | 1016 | 0.05 (0.09) | 0.61 | -0.04 (0.02) | 0.05 | 0.16 (0.13) | 0.20 | 1084 | 0.22 (0.17) | 0.19 | -0.03 (0.02) | 0.12 | 0.14 (0.21) | 0.51 | -0.43 (0.53) | 0.42 | -0.05 (0.03) | 0.09 | 1.03 (0.56) | 0.06 |
| **P2S5** | | | | | | | | | | | | | | | | | | | | |
| *t*=base | 1073 | 0.16 (0.07) | 0.03 | 0.009 (0.02) | 0.69 | 0.09 (0.13) | 0.50 | 1087 | 0.29 (0.12) | 0.02 | 0.007 (0.02) | 0.76 | 0.10 (0.20) | 0.61 | 0.42 (0.17) | 0.01 | 0.003 (0.02) | 0.90 | 0.26 (0.31) | 0.41 |
| *t*=fu | 1018 | 0.09 (0.08) | 0.27 | 0.005 (0.02) | 0.82 | 0.19 (0.11) | 0.09 | 1087 | 0.27 (0.13) | 0.04 | 0.01 (0.02) | 0.64 | 0.33 (0.17) | 0.05 | 0.34 (0.18) | 0.05 | 0.008 (0.02) | 0.74 | 0.24 (0.24) | 0.33 |
| **P2S6** | | | | | | | | | | | | | | | | | | | | |
| *t*=base | 1070 | 0.09 (0.08) | 0.30 | -0.01 (0.03) | 0.71 | 0.23 (0.14) | 0.10 | 1084 | 0.32 (0.14) | 0.02 | 0.007 (0.03) | 0.80 | 0.16 (0.25) | 0.54 | 0.26 (0.21) | 0.21 | 0.03 (0.03) | 0.37 | 0.78 (0.43) | 0.07 |
| *t*=fu | 1015 | 0.13 (0.08) | 0.13 | -0.01 (0.03) | 0.61 | 0.06 (0.11) | 0.60 | 1084 | 0.44 (0.13) | 0.001 | -0.004 (0.03) | 0.87 | 0.009 (0.20) | 0.96 | 0.44 (0.21) | 0.03 | -0.0003 (0.03) | 0.99 | 0.17 (0.30) | 0.57 |
| **P2S7** | | | | | | | | | | | | | | | | | | | | |
| *t*=base | 1072 | 0.15 (0.07) | 0.05 | -0.05 (0.03) | 0.06 | 0.04 (0.11) | 0.72 | 1086 | 0.32 (0.10) | 0.002 | -0.04 (0.03) | 0.11 | -0.08 (0.19) | 0.67 | 0.36 (0.20) | 0.07 | -0.04 (0.03) | 0.13 | -0.15 (0.29) | 0.60 |
| *t*=fu | 1018 | 0.31 (0.07) | <0.001 | -0.04 (0.03) | 0.12 | -0.11 (0.11) | 0.31 | 1086 | 0.46 (0.11) | <0.001 | -0.03 (0.03) | 0.20 | -0.14 (0.20) | 0.46 | 0.42 (0.22) | 0.06 | -0.05 (0.03) | 0.12 | -0.31 (0.35) | 0.38 |
| **P2S8** | | | | | | | | | | | | | | | | | | | | |
| *t*=base | 1073 | 0.33 (0.08) | <0.001 | -0.007 (0.02) | 0.77 | -0.07 (0.10) | 0.51 | 1087 | 0.32 (0.12) | 0.01 | -0.01 (0.02) | 0.56 | -0.02 (0.17) | 0.91 | 0.69 (0.30) | 0.02 | -0.001 (0.03) | 0.96 | -0.32 (0.33) | 0.33 |
| *t*=fu | 1018 | 0.15 (0.07) | 0.05 | -0.02 (0.02) | 0.50 | 0.18 (0.11) | 0.09 | 1087 | 0.37 (0.11) | 0.001 | -0.002 (0.02) | 0.94 | 0.21 (0.16) | 0.19 | 0.34 (0.22) | 0.12 | -0.005 (0.02) | 0.85 | 0.23 (0.27) | 0.40 |
| **P2S9** | | | | | | | | | | | | | | | | | | | | |
| *t*=base | 1072 | 0.13 (0.08) | 0.12 | -0.009 (0.02) | 0.64 | 0.06 (0.14) | 0.67 | 1086 | 0.45 (0.14) | 0.001 | -0.01 (0.02) | 0.48 | 0.18 (0.30) | 0.56 | 0.52 (0.24) | 0.03 | -0.01 (0.02) | 0.49 | 0.16 (0.53) | 0.76 |
| *t*=fu | 1017 | 0.22 (0.08) | 0.006 | -0.002 (0.02) | 0.92 | 0.04 (0.13) | 0.77 | 1086 | 0.37 (0.13) | 0.005 | -0.006 (0.02) | 0.77 | 0.08 (0.22) | 0.72 | 0.20 (0.27) | 0.46 | -0.01 (0.02) | 0.62 | 0.03 (0.40) | 0.94 |
| **Experiment Part 2: Injunctive norms (Average P2S2 to P2S9)** | | | | | | | | | | | | | | | | | | | | |
| *t*=base | 1064 | 0.15 (0.07) | 0.02 | -0.008 (0.01) | 0.56 | 0.21 (0.12) | 0.08 | 1078 | 0.31 (0.11) | 0.005 | -0.008 (0.01) | 0.60 | 0.15 (0.25) | 0.54 | 0.30 (0.16) | 0.07 | -0.002 (0.02) | 0.89 | 0.46 (0.38) | 0.23 |
| *t*=fu | 1011 | 0.19 (0.07) | 0.005 | -0.01 (0.01) | 0.50 | 0.13 (0.11) | 0.22 | 1078 | 0.35 (0.10) | 0.001 | -0.003 (0.01) | 0.84 | 0.25 (0.16) | 0.12 | 0.17 (0.18) | 0.33 | -0.008 (0.02) | 0.61 | 0.30 (0.29) | 0.29 |
| **P3Q1** | | | | | | | | | | | | | | | | | | | | |
| *t*=base | 1073 | 0.12 (0.08) | 0.12 | -0.02 (0.03) | 0.56 | -0.09 (0.13) | 0.50 | 1087 | 0.23 (0.13) | 0.08 | -0.01 (0.03) | 0.76 | 0.04 (0.24) | 0.85 | 0.07 (0.19) | 0.72 | -0.01 (0.03) | 0.70 | 0.23 (0.31) | 0.45 |
| *t*=fu | 1018 | 0.13 (0.08) | 0.12 | -0.03 (0.03) | 0.30 | -0.10 (0.12) | 0.41 | 1087 | 0.27 (0.14) | 0.05 | -0.02 (0.03) | 0.59 | 0.06 (0.20) | 0.77 | 0.13 (0.25) | 0.62 | -0.02 (0.03) | 0.47 | 0.13 (0.34) | 0.70 |
| **P3Q2** | | | | | | | | | | | | | | | | | | | | |
| *t*=base | 1073 | 0.20 (0.07) | 0.003 | 0.04 (0.03) | 0.26 | -0.01 (0.11) | 0.92 | 1087 | 0.28 (0.10) | 0.005 | 0.05 (0.03) | 0.10 | 0.10 (0.16) | 0.54 | 0.23 (0.14) | 0.09 | 0.06 (0.03) | 0.09 | 0.22 (0.20) | 0.29 |
| *t*=fu | 1018 | 0.22 (0.07) | 0.003 | 0.02 (0.03) | 0.60 | -0.11 (0.11) | 0.33 | 1087 | 0.34 (0.10) | 0.001 | 0.02 (0.03) | 0.49 | -0.10 (0.16) | 0.51 | 0.24 (0.16) | 0.15 | 0.02 (0.03) | 0.44 | 0.08 (0.22) | 0.70 |
| **Experiment Part 3: Descriptive norms (Average P3Q1 to P3Q2)** | | | | | | | | | | | | | | | | | | | | |
| *t*=base | 1073 | 0.14 (0.07) | 0.04 | 0.02 (0.03) | 0.56 | 0.01 (0.12) | 0.93 | 1087 | 0.21 (0.11) | 0.04 | 0.03 (0.03) | 0.34 | 0.16 (0.18) | 0.39 | 0.09 (0.15) | 0.54 | 0.03 (0.03) | 0.40 | 0.31 (0.22) | 0.16 |
| *t*=fu | 1018 | 0.17 (0.08) | 0.03 | -0.003 (0.03) | 0.92 | -0.12 (0.11) | 0.28 | 1087 | 0.27 (0.12) | 0.02 | 0.008 (0.03) | 0.79 | 0.008 (0.17) | 0.96 | 0.08 (0.20) | 0.67 | 0.005 (0.03) | 0.87 | 0.17 (0.25) | 0.49 |
| **Donation to ASSIST/Dead Cool** | | | | | | | | | | | | | | | | | | | | |
| *t*=base | **1071** | **0.30 (0.07)** | **<0.001** | **-0.29 (0.16)** | **0.06** | **-0.38 (0.12)** | **0.001** | 1085 | 0.26 (0.12) | 0.03 | -0.29 (0.16) | 0.07 | -0.26 (0.19) | 0.17 | **0.83 (0.27)** | **0.002** | **-0.23 (0.17)** | **0.18** | **-1.24 (0.43)** | **0.004** |
| *t*=fu | 1016 | 0.29 (0.07) | <0.001 | -0.09 (0.15) | 0.55 | 0.09 (0.11) | 0.41 | 1085 | 0.59 (0.11) | <0.001 | -0.15 (0.15) | 0.30 | -0.30 (0.17) | 0.09 | 0.67 (0.18) | <0.001 | -0.10 (0.16) | 0.53 | -0.02 (0.27) | 0.95 |
| **IN1** | | | | | | | | | | | | | | | | | | | | |
| *t*=base | 1073 | -0.09 (0.11) | 0.40 | 0.04 (0.04) | 0.42 | 0.09 (0.13) | 0.51 | 1082 | -0.08 (0.19) | 0.67 | 0.04 (0.04) | 0.35 | 0.21 (0.26) | 0.42 | 0.28 (0.29) | 0.33 | 0.05 (0.04) | 0.29 | 0.17 (0.41) | 0.68 |
| *t*=fu | 1070 | 0.09 (0.08) | 0.27 | 0.03 (0.04) | 0.43 | -0.06 (0.12) | 0.64 | 1082 | -0.09 (0.17) | 0.60 | 0.05 (0.04) | 0.27 | -0.15 (0.24) | 0.53 | 0.42 (0.29) | 0.15 | 0.03 (0.04) | 0.45 | -0.52 (0.44) | 0.24 |
| **IN2** | | | | | | | | | | | | | | | | | | | | |
| *t*=base | 1075 | -0.15 (0.08) | 0.07 | 0.04 (0.03) | 0.14 | 0.37 (0.17) | 0.03 | 1084 | -0.14 (0.23) | 0.54 | 0.04 (0.03) | 0.18 | 0.55 (0.33) | 0.10 | -0.65 (0.77) | 0.39 | 0.04 (0.04) | 0.32 | 1.84 (0.96) | 0.06 |
| *t*=fu | 1072 | 0.17 (0.11) | 0.12 | 0.04 (0.03) | 0.16 | -0.07 (0.14) | 0.63 | 1084 | 0.17 (0.13) | 0.18 | 0.04 (0.03) | 0.17 | -0.12 (0.21) | 0.57 | -0.26 (0.44) | 0.56 | 0.04 (0.03) | 0.18 | 0.64 (0.55) | 0.25 |
| **IN3** | | | | | | | | | | | | | | | | | | | | |
| *t*=base | 1073 | 0.08 (0.08) | 0.34 | 0.03 (0.04) | 0.51 | 0.07 (0.13) | 0.58 | 1082 | 0.20 (0.17) | 0.23 | 0.01 (0.04) | 0.75 | -0.06 (0.21) | 0.78 | 0.11 (0.27) | 0.69 | 0.002 (0.05) | 0.97 | 0.34 (0.34) | 0.32 |
| *t*=fu | 1070 | 0.15 (0.07) | 0.05 | 0.04 (0.04) | 0.34 | -0.17 (0.11) | 0.12 | 1082 | -0.02 (0.13) | 0.91 | 0.04 (0.04) | 0.32 | 0.04 (0.19) | 0.83 | -0.28 (0.32) | 0.38 | 0.04 (0.04) | 0.35 | 0.66 (0.38) | 0.09 |
| **IN4** | | | | | | | | | | | | | | | | | | | | |
| *t*=base | 1071 | 0.11 (0.07) | 0.14 | -0.003 (0.05) | 0.96 | -0.19 (0.12) | 0.10 | 1081 | 0.08 (0.19) | 0.67 | -0.001 (0.05) | 0.98 | -0.23 (0.26) | 0.36 | 0.64 (0.39) | 0.10 | -0.07 (0.06) | 0.24 | 0.13 (0.61) | 0.83 |
| *t*=fu | 1069 | 0.13 (0.07) | 0.06 | 0.005 (0.05) | 0.92 | -0.17 (0.11) | 0.10 | 1081 | 0.21 (0.16) | 0.21 | -0.005 (0.05) | 0.92 | -0.07 (0.23) | 0.77 | 0.59 (0.28) | 0.04 | 0.004 (0.05) | 0.94 | -0.41 (0.42) | 0.33 |
| **IN5** | | | | | | | | | | | | | | | | | | | | |
| *t*=base | 1073 | -0.04 (0.08) | 0.62 | -0.02 (0.05) | 0.73 | 0.06 (0.11) | 0.59 | 1082 | 0.12 (0.17) | 0.49 | -0.02 (0.05) | 0.67 | -0.32 (0.22) | 0.15 | 0.14 (0.37) | 0.70 | -0.03 (0.06) | 0.65 | -0.27 (0.45) | 0.54 |
| *t*=fu | **1070** | **0.18 (0.08)** | **0.02** | **-0.01 (0.05)** | **0.82** | **-0.39 (0.12)** | **0.001** | **1082** | **0.23 (0.14)** | **0.10** | **-0.02 (0.05)** | **0.73** | **-0.59 (0.23)** | **0.01** | 0.31 (0.25) | 0.21 | -0.02 (0.05) | 0.75 | -0.73 (0.41) | 0.08 |
| **IN6** | | | | | | | | | | | | | | | | | | | | |
| *t*=base | 1074 | 0.19 (0.08) | 0.02 | 0.07 (0.05) | 0.16 | 0.04 (0.11) | 0.72 | 1083 | 0.02 (0.15) | 0.88 | 0.08 (0.05) | 0.13 | 0.36 (0.19) | 0.06 | 0.12 (0.23) | 0.59 | 0.07 (0.05) | 0.21 | 0.66 (0.31) | 0.03 |
| *t*=fu | 1071 | 0.29 (0.08) | <0.001 | 0.06 (0.05) | 0.23 | -0.11 (0.11) | 0.34 | 1083 | 0.24 (0.14) | 0.08 | 0.06 (0.05) | 0.24 | 0.06 (0.18) | 0.75 | -0.06 (0.41) | 0.89 | 0.08 (0.06) | 0.23 | 0.57 (0.44) | 0.19 |
| **IN7** | | | | | | | | | | | | | | | | | | | | |
| *t*=base | 1075 | 0.13 (0.08) | 0.10 | 0.12 (0.05) | 0.02 | -0.04 (0.11) | 0.69 | 1084 | 0.15 (0.18) | 0.41 | 0.12 (0.05) | 0.02 | 0.11 (0.23) | 0.62 | 0.45 (0.28) | 0.10 | 0.09 (0.05) | 0.08 | 0.21 (0.34) | 0.54 |
| *t*=fu | 1072 | 0.16 (0.08) | 0.05 | 0.11 (0.05) | 0.03 | 0.07 (0.12) | 0.54 | 1084 | 0.18 (0.15) | 0.22 | 0.11 (0.05) | 0.04 | 0.06 (0.21) | 0.79 | 0.14 (0.33) | 0.67 | 0.10 (0.06) | 0.10 | 0.32 (0.37) | 0.40 |
| **Self-report injunctive norms (Average IN1 to IN7)** | | | | | | | | | | | | | | | | | | | | |
| *t*=base | 1068 | 0.02 (0.09) | 0.78 | 0.03 (0.03) | 0.30 | 0.11 (0.12) | 0.35 | 1078 | -0.10 (0.21) | 0.63 | 0.04 (0.03) | 0.24 | 0.29 (0.24) | 0.23 | 0.07 (0.36) | 0.84 | 0.02 (0.03) | 0.65 | 0.47 (0.41) | 0.25 |
| *t*=fu | 1066 | 0.24 (0.07) | 0.001 | 0.03 (0.03) | 0.33 | -0.12 (0.11) | 0.27 | 1078 | 0.21 (0.14) | 0.12 | 0.02 (0.03) | 0.40 | -0.03 (0.18) | 0.86 | -0.03 (0.34) | 0.94 | 0.03 (0.03) | 0.37 | 0.38 (0.38) | 0.32 |
| **DN1.1** | | | | | | | | | | | | | | | | | | | | |
| *t*=base | **1075** | **0.37 (0.13)** | **0.003** | **0.04 (0.04)** | **0.29** | **-0.41 (0.16)** | **0.01** | **1084** | **0.46 (0.17)** | **0.008** | **0.06 (0.03)** | **0.06** | **-0.86 (0.24)** | **<0.001** | 1.13 (0.31) | <0.001 | 0.09 (0.09) | 0.33 | -2.22 (1.09) | 0.04 |
| *t*=fu | 1072 | 0.33 (0.10) | 0.002 | 0.02 (0.04) | 0.55 | 0.0008 (0.17) | 1.00 | 1084 | 0.30 (0.15) | 0.05 | 0.05 (0.04) | 0.24 | -0.37 (0.31) | 0.24 | 0.67 (0.20) | 0.001 | 0.02 (0.05) | 0.74 | -0.46 (0.49) | 0.35 |
| **DN1.2** | | | | | | | | | | | | | | | | | | | | |
| *t*=base | 1076 | 0.13 (0.05) | 0.02 | -0.03 (0.04) | 0.53 | -0.003 (0.11) | 0.98 | 1085 | 0.06 (0.08) | 0.43 | -0.03 (0.04) | 0.43 | 0.09 (0.14) | 0.52 | 0.16 (0.09) | 0.07 | -0.04 (0.04) | 0.41 | 0.01 (0.21) | 0.95 |
| *t*=fu | 1073 | -0.01 (0.05) | 0.86 | -0.006 (0.04) | 0.88 | 0.02 (0.09) | 0.86 | 1085 | 0.01 (0.09) | 0.89 | -0.01 (0.04) | 0.76 | 0.04 (0.15) | 0.80 | 0.12 (0.09) | 0.19 | -0.007 (0.04) | 0.88 | -0.12 (0.27) | 0.66 |
| **DN1.3** | | | | | | | | | | | | | | | | | | | | |
| *t*=base | 1075 | 0.11 (0.06) | 0.06 | -0.0004 (0.05) | 0.99 | -0.08 (0.09) | 0.38 | 1084 | 0.08 (0.11) | 0.49 | -0.0006 (0.05) | 0.99 | -0.05 (0.15) | 0.75 | 0.09 (0.15) | 0.53 | -0.03 (0.06) | 0.60 | 0.18 (0.26) | 0.49 |
| *t*=fu | 1072 | 0.02 (0.07) | 0.82 | 0.005 (0.05) | 0.93 | 0.02 (0.09) | 0.81 | 1084 | 0.03 (0.11) | 0.78 | 0.004 (0.05) | 0.94 | -0.007 (0.15) | 0.96 | 0.06 (0.16) | 0.71 | -0.008 (0.05) | 0.89 | 0.08 (0.27) | 0.77 |
| **DN1.4** | | | | | | | | | | | | | | | | | | | | |
| *t*=base | 1075 | 0.05 (0.06) | 0.45 | 0.03 (0.04) | 0.42 | -0.07 (0.08) | 0.38 | 1084 | 0.31 (0.23) | 0.18 | 0.03 (0.04) | 0.44 | -0.41 (0.27) | 0.14 | 0.19 (0.24) | 0.43 | 0.03 (0.04) | 0.49 | -0.11 (0.30) | 0.70 |
| *t*=fu | 1072 | -0.04 (0.05) | 0.46 | 0.04 (0.04) | 0.37 | -0.12 (0.09) | 0.16 | 1084 | 0.25 (0.23) | 0.28 | 0.03 (0.04) | 0.43 | -0.36 (0.27) | 0.19 | -0.29 (0.38) | 0.44 | 0.04 (0.04) | 0.32 | 0.26 (0.42) | 0.54 |
| **DN1.5** | | | | | | | | | | | | | | | | | | | | |
| *t*=base | 1075 | 0.12 (0.08) | 0.15 | 0.03 (0.03) | 0.28 | -0.09 (0.10) | 0.40 | 1084 | 0.27 (0.18) | 0.12 | 0.03 (0.03) | 0.31 | -0.27 (0.21) | 0.18 | -0.15 (0.33) | 0.65 | 0.02 (0.03) | 0.48 | 0.76 (0.44) | 0.09 |
| *t*=fu | 1072 | 0.03 (0.06) | 0.56 | 0.04 (0.03) | 0.24 | -0.11 (0.10) | 0.27 | 1084 | 0.25 (0.15) | 0.10 | 0.03 (0.03) | 0.36 | -0.20 (0.19) | 0.30 | -0.22 (0.30) | 0.46 | 0.03 (0.03) | 0.37 | 0.50 (0.34) | 0.14 |
| **Self-report descriptive norms 1 (Average DN1.1 to DN1.5)** | | | | | | | | | | | | | | | | | | | | |
| *t*=base | 1073 | 0.14 (0.07) | 0.03 | 0.001 (0.02) | 0.96 | -0.13 (0.09) | 0.16 | 1082 | 0.08 (0.09) | 0.37 | 0.004 (0.02) | 0.85 | -0.10 (0.14) | 0.46 | 0.18 (0.12) | 0.13 | -0.01 (0.03) | 0.62 | -0.03 (0.18) | 0.88 |
| *t*=fu | 1070 | 0.08 (0.06) | 0.18 | 0.009 (0.02) | 0.68 | -0.15 (0.09) | 0.09 | 1082 | 0.04 (0.09) | 0.68 | 0.007 (0.02) | 0.75 | -0.08 (0.14) | 0.57 | 0.16 (0.11) | 0.14 | -0.002 (0.03) | 0.95 | -0.11 (0.20) | 0.57 |
| **DN2.1** | | | | | | | | | | | | | | | | | | | | |
| *t*=base | 1076 | 0.18 (0.08) | 0.03 | 0.07 (0.04) | 0.12 | -0.05 (0.15) | 0.72 | 1085 | 0.16 (0.13) | 0.23 | 0.06 (0.04) | 0.17 | 0.07 (0.22) | 0.75 | 0.23 (0.16) | 0.16 | 0.06 (0.05) | 0.20 | -0.08 (0.39) | 0.83 |
| *t*=fu | 1073 | 0.20 (0.08) | 0.01 | 0.07 (0.04) | 0.07 | -0.04 (0.13) | 0.74 | 1085 | 0.24 (0.14) | 0.10 | 0.07 (0.04) | 0.12 | -0.21 (0.22) | 0.36 | 0.16 (0.19) | 0.41 | 0.08 (0.05) | 0.13 | -0.20 (0.34) | 0.56 |
| **DN2.2** | | | | | | | | | | | | | | | | | | | | |
| *t*=base | 1076 | 0.14 (0.07) | 0.07 | 0.02 (0.05) | 0.66 | 0.10 (0.13) | 0.45 | 1085 | 0.37 (0.11) | 0.001 | 0.02 (0.05) | 0.75 | -0.20 (0.20) | 0.33 | 0.54 (0.19) | 0.004 | 0.01 (0.06) | 0.80 | -0.30 (0.31) | 0.34 |
| *t*=fu | 1073 | 0.13 (0.08) | 0.10 | 0.03 (0.05) | 0.50 | -0.05 (0.11) | 0.65 | 1085 | 0.20 (0.11) | 0.08 | 0.03 (0.05) | 0.57 | -0.10 (0.18) | 0.56 | 0.45 (0.17) | 0.007 | 0.04 (0.06) | 0.53 | -0.43 (0.31) | 0.16 |
| **DN2.3** | | | | | | | | | | | | | | | | | | | | |
| *t*=base | 1076 | 0.24 (0.09) | 0.005 | 0.05 (0.04) | 0.21 | 0.05 (0.16) | 0.73 | 1085 | 0.50 (0.11) | <0.001 | -0.00005 (0.04) | 1.00 | 0.26 (0.20) | 0.20 | 0.59 (0.14) | <0.001 | -0.01 (0.04) | 0.78 | 0.34 (0.32) | 0.29 |
| *t*=fu | 1073 | 0.18 (0.06) | 0.004 | 0.05 (0.04) | 0.16 | 0.03 (0.12) | 0.83 | 1085 | 0.32 (0.11) | 0.004 | 0.01 (0.04) | 0.71 | 0.18 (0.17) | 0.27 | 0.53 (0.14) | <0.001 | 0.004 (0.04) | 0.92 | 0.01 (0.24) | 0.96 |
| **Self-report descriptive norms 2 (Average DN2.1 to DN2.3)** | | | | | | | | | | | | | | | | | | | | |
| *t*=base | 1076 | 0.20 (0.08) | 0.01 | 0.03 (0.03) | 0.33 | 0.05 (0.13) | 0.72 | 1085 | 0.40 (0.11) | <0.001 | 0.02 (0.03) | 0.55 | -0.13 (0.22) | 0.57 | 0.43 (0.13) | 0.001 | -0.04 (0.06) | 0.48 | 0.65 (0.63) | 0.31 |
| *t*=fu | 1073 | 0.14 (0.07) | 0.05 | 0.05 (0.03) | 0.06 | -0.13 (0.11) | 0.27 | 1085 | 0.29 (0.10) | 0.005 | 0.03 (0.03) | 0.31 | -0.15 (0.16) | 0.34 | 0.39 (0.13) | 0.003 | 0.04 (0.04) | 0.30 | -0.41 (0.33) | 0.22 |
| **Self-report smoking behavior** | | | | | | | | | | | | | | | | | | | | |
| *t*=base | 1083 | 0.10 (0.09) | 0.24 | -0.05 (0.03) | 0.11 | 0.14 (0.13) | 0.27 | 1093 | -0.002 (0.15) | 0.99 | -0.05 (0.04) | 0.19 | 0.44 (0.21) | 0.04 | 0.56 (0.39) | 0.16 | -0.08 (0.04) | 0.05 | -0.009 (0.43) | 0.98 |
| *t*=fu | 1081 | 0.19 (0.09) | 0.05 | -0.03 (0.03) | 0.28 | 0.12 (0.12) | 0.31 | 1093 | -0.02 (0.16) | 0.90 | -0.04 (0.03) | 0.29 | 0.34 (0.18) | 0.06 | 0.31 (0.30) | 0.30 | -0.05 (0.03) | 0.17 | 0.09 (0.31) | 0.77 |
| **Intentions** | | | | | | | | | | | | | | | | | | | | |
| *t*=base | 1078 | 0.13 (0.11) | 0.23 | 0.22 (0.07) | 0.002 | 0.09 (0.16) | 0.59 | 1087 | 0.69 (0.20) | 0.001 | 0.18 (0.07) | 0.01 | -0.47 (0.27) | 0.08 | 1.19 (0.30) | <0.001 | 0.13 (0.07) | 0.08 | -0.65 (0.49) | 0.18 |
| *t*=fu | 1075 | 0.29 (0.10) | 0.004 | 0.19 (0.07) | 0.007 | -0.25 (0.13) | 0.06 | 1087 | 0.20 (0.14) | 0.16 | 0.21 (0.08) | 0.006 | -0.31 (0.21) | 0.15 | 0.47 (0.16) | 0.002 | 0.14 (0.08) | 0.08 | -0.33 (0.33) | 0.32 |
| **Knowledge** | | | | | | | | | | | | | | | | | | | | |
| *t*=base | 1080 | 0.25 (0.08) | 0.001 | 0.36 (0.08) | <0.001 | -0.10 (0.10) | 0.34 | 1089 | 0.52 (0.10) | <0.001 | 0.28 (0.08) | 0.001 | -0.26 (0.14) | 0.08 | 0.55 (0.13) | <0.001 | 0.27 (0.09) | 0.002 | -0.28 (0.20) | 0.16 |
| *t*=fu | 1077 | 0.33 (0.07) | <0.001 | 0.27 (0.09) | 0.002 | -0.13 (0.11) | 0.21 | 1089 | 0.48 (0.09) | <0.001 | 0.21 (0.09) | 0.02 | -0.26 (0.15) | 0.08 | 0.46 (0.12) | <0.001 | 0.19 (0.10) | 0.06 | -0.19 (0.23) | 0.43 |
| **Attitudes** | | | | | | | | | | | | | | | | | | | | |
| *t*=base | 1067 | 0.23 (0.09) | 0.008 | 0.10 (0.04) | 0.006 | -0.14 (0.11) | 0.20 | 1076 | 0.07 (0.15) | 0.64 | 0.12 (0.04) | 0.003 | -0.22 (0.21) | 0.29 | 0.32 (0.25) | 0.20 | 0.08 (0.04) | 0.05 | 0.07 (0.45) | 0.87 |
| *t*=fu | 1064 | 0.20 (0.10) | 0.04 | 0.10 (0.04) | 0.01 | -0.07 (0.12) | 0.56 | **1076** | **-0.44 (0.19)** | **0.02** | **0.13 (0.04)** | **0.001** | **0.62 (0.23)** | **0.008** | 0.02 (0.25) | 0.94 | 0.09 (0.04) | 0.04 | 0.42 (0.33) | 0.20 |
| **Self-efficacy (Emotional)** | | | | | | | | | | | | | | | | | | | | |
| *t*=base | 1072 | 0.14 (0.10) | 0.17 | 0.09 (0.05) | 0.07 | -0.08 (0.14) | 0.57 | 1081 | 0.27 (0.16) | 0.10 | 0.07 (0.05) | 0.18 | 0.08 (0.24) | 0.75 | 0.70 (0.34) | 0.04 | 0.04 (0.05) | 0.48 | -0.35 (0.38) | 0.36 |
| *t*=fu | 1068 | 0.38 (0.09) | <0.001 | 0.07 (0.05) | 0.15 | -0.25 (0.12) | 0.05 | 1081 | 0.03 (0.15) | 0.85 | 0.08 (0.05) | 0.10 | 0.32 (0.18) | 0.08 | 0.28 (0.26) | 0.28 | 0.06 (0.06) | 0.33 | 0.20 (0.28) | 0.49 |
| **Self-efficacy (Friends)** | | | | | | | | | | | | | | | | | | | | |
| *t*=base | 1078 | 0.10 (0.08) | 0.22 | 0.04 (0.05) | 0.42 | -0.11 (0.12) | 0.36 | 1087 | 0.31 (0.14) | 0.03 | 0.03 (0.05) | 0.55 | -0.28 (0.22) | 0.20 | 0.27 (0.25) | 0.29 | 0.03 (0.05) | 0.62 | -0.16 (0.33) | 0.63 |
| *t*=fu | 1075 | 0.29 (0.09) | 0.001 | 0.03 (0.05) | 0.48 | -0.28 (0.12) | 0.02 | 1087 | 0.14 (0.13) | 0.29 | 0.04 (0.05) | 0.44 | -0.07 (0.18) | 0.70 | 0.38 (0.26) | 0.14 | 0.02 (0.05) | 0.69 | -0.25 (0.32) | 0.44 |
| **Self-efficacy (Opportunity)** | | | | | | | | | | | | | | | | | | | | |
| *t*=base | 1079 | 0.16 (0.09) | 0.08 | 0.07 (0.04) | 0.09 | -0.07 (0.15) | 0.61 | 1088 | 0.40 (0.18) | 0.03 | 0.04 (0.05) | 0.33 | -0.04 (0.26) | 0.87 | 0.59 (0.38) | 0.13 | 0.03 (0.05) | 0.49 | -0.25 (0.44) | 0.57 |
| *t*=fu | 1076 | 0.20 (0.10) | 0.03 | 0.07 (0.04) | 0.11 | -0.13 (0.13) | 0.34 | 1088 | 0.19 (0.14) | 0.16 | 0.06 (0.04) | 0.17 | 0.03 (0.18) | 0.85 | 0.26 (0.28) | 0.36 | 0.05 (0.05) | 0.30 | 0.05 (0.31) | 0.86 |
| **Perceived physical risks** | | | | | | | | | | | | | | | | | | | | |
| *t*=base | 1074 | 0.08 (0.08) | 0.33 | 5.10 (1.28) | <0.001 | -0.008 (0.10) | 0.94 | 1082 | 0.15 (0.15) | 0.31 | 4.88 (1.29) | <0.001 | -0.008 (0.18) | 0.96 | -0.09 (0.23) | 0.70 | 5.15 (1.32) | <0.001 | 0.25 (0.28) | 0.38 |
| *t*=fu | 1070 | 0.15 (0.08) | 0.05 | 4.50 (1.30) | 0.001 | -0.08 (0.11) | 0.47 | 1082 | 0.11 (0.16) | 0.51 | 4.55 (1.42) | 0.001 | 0.005 (0.21) | 0.98 | -0.97 (0.45) | 0.03 | 7.42 (1.88) | <0.001 | 1.11 (0.50) | 0.03 |
| **Perceived social risks** | | | | | | | | | | | | | | | | | | | | |
| *t*=base | 1076 | 0.09 (0.08) | 0.29 | 5.05 (1.42) | <0.001 | 0.13 (0.10) | 0.19 | 1085 | 0.42 (0.15) | 0.005 | 4.39 (1.41) | 0.002 | -0.20 (0.16) | 0.23 | 0.58 (0.21) | 0.006 | 4.03 (1.44) | 0.005 | -0.23 (0.22) | 0.31 |
| *t*=fu | 1073 | 0.18 (0.08) | 0.02 | 4.24 (1.44) | 0.003 | -0.11 (0.11) | 0.33 | 1085 | 0.33 (0.12) | 0.007 | 3.66 (1.47) | 0.01 | -0.18 (0.16) | 0.26 | 0.42 (0.21) | 0.04 | 2.87 (1.62) | 0.08 | -0.03 (0.23) | 0.90 |
| **Perceived addiction risks** | | | | | | | | | | | | | | | | | | | | |
| *t*=base | 1016 | 0.16 (0.08) | 0.05 | 2.69 (1.48) | 0.07 | 0.14 (0.11) | 0.20 | 1025 | 0.47 (0.12) | <0.001 | 2.04 (1.45) | 0.16 | 0.06 (0.15) | 0.68 | 0.69 (0.14) | <0.001 | 1.78 (1.45) | 0.22 | 0.03 (0.17) | 0.84 |
| *t*=fu | 1009 | 0.13 (0.06) | 0.03 | 2.27 (1.49) | 0.13 | 0.02 (0.10) | 0.80 | 1025 | 0.37 (0.10) | <0.001 | 1.40 (1.49) | 0.35 | 0.05 (0.14) | 0.70 | 0.53 (0.12) | <0.001 | 0.92 (1.48) | 0.54 | 0.08 (0.16) | 0.61 |
| **Perceived benefits** | | | | | | | | | | | | | | | | | | | | |
| *t*=base | 1029 | -0.04 (0.08) | 0.60 | 1.98 (1.27) | 0.12 | -0.004 (0.13) | 0.98 | 1038 | 0.23 (0.18) | 0.19 | 2.40 (1.26) | 0.06 | -0.30 (0.25) | 0.22 | 0.61 (0.36) | 0.09 | 2.92 (1.29) | 0.02 | -0.47 (0.44) | 0.29 |
| *t*=fu | 1025 | 0.08 (0.08) | 0.31 | 2.06 (1.26) | 0.10 | -0.12 (0.11) | 0.28 | 1038 | -0.09 (0.19) | 0.64 | 2.34 (1.26) | 0.06 | 0.01 (0.25) | 0.97 | 0.14 (0.31) | 0.65 | 2.55 (1.27) | 0.05 | -1.00 (0.58) | 0.08 |
| **Perceived behavioral control (easy to quit)** | | | | | | | | | | | | | | | | | | | | |
| *t*=base | 1076 | 0.27 (0.08) | 0.002 | -0.16 (0.09) | 0.06 | 0.002 (0.11) | 0.99 | 1085 | 0.55 (0.13) | <0.001 | -0.15 (0.09) | 0.08 | -0.09 (0.15) | 0.57 | 0.83 (0.15) | <0.001 | -0.13 (0.09) | 0.13 | -0.24 (0.17) | 0.16 |
| *t*=fu | 1073 | 0.22 (0.08) | 0.003 | -0.13 (0.09) | 0.13 | -0.11 (0.11) | 0.30 | 1085 | 0.58 (0.10) | <0.001 | -0.08 (0.09) | 0.34 | -0.20 (0.14) | 0.15 | 0.62 (0.12) | <0.001 | -0.07 (0.09) | 0.40 | -0.04 (0.16) | 0.79 |
| **Perceived behavioral control (to avoid smoking)** | | | | | | | | | | | | | | | | | | | | |
| *t*=base | 1080 | 0.10 (0.10) | 0.32 | 0.14 (0.07) | 0.07 | -0.08 (0.14) | 0.57 | 1089 | 0.27 (0.18) | 0.12 | 0.11 (0.07) | 0.13 | 0.18 (0.26) | 0.48 | 0.61 (0.28) | 0.03 | 0.06 (0.08) | 0.42 | 0.34 (0.41) | 0.40 |
| *t*=fu | 1077 | -0.07 (0.10) | 0.49 | 0.16 (0.07) | 0.03 | 0.29 (0.14) | 0.03 | 1089 | -0.11 (0.19) | 0.57 | 0.15 (0.08) | 0.05 | 0.40 (0.25) | 0.12 | 0.12 (0.30) | 0.68 | 0.11 (0.08) | 0.18 | 0.56 (0.35) | 0.11 |
| **Objectively measured smoking behavior** | | | | | | | | | | | | | | | | | | | | |
| *t*=base | **1041** | **0.29 (0.07)** | **<0.001** | **0.26 (0.10)** | **0.008** | **0.19 (0.07)** | **0.01** | **1048** | **0.26 (0.07)** | **<0.001** | **0.22 (0.09)** | **0.02** | **0.27 (0.07)** | **<0.001** | 0.51 (0.07) | <0.001 | 0.34 (0.09) | <0.001 | 0.16 (0.09) | 0.06 |
| *t*=fu | 1022 | 0.39 (0.11) | <0.001 | 0.02 (0.09) | 0.79 | 0.21 (0.12) | 0.08 | 1048 | 0.85 (0.06) | <0.001 | 0.04 (0.08) | 0.61 | -0.02 (0.09) | 0.84 | 0.86 (0.09) | <0.001 | 0.02 (0.08) | 0.76 | 0.008 (0.10) | 0.94 |
| **Smoking susceptibility^e^** | | | | | | | | | | | | | | | | | | | | |
|  | n | **Percent_-i,t_^f^** | | **M^g^** | | **M*Percent_-i,t_^h^** | | n | **Percent_-i,t_^f^** | | **M^g^** | | **M*Percent_-i,t_^h^** | | **Percent_-i,t_^f^** | | **M^g^** | | **M*Percent_-i,t_^h^** | |
|  |  | OR (SE) | p-value | OR (SE) | p-value | OR (SE) | p-value |  | OR (SE) | p-value | OR (SE) | p-value | OR (SE) | p-value | OR (SE) | p-value | OR (SE) | p-value | OR (SE) | p-value |
| *t*=base | 1078 | 1.14 (0.05) | 0.003 | 0.70 (0.11) | 0.02 | 0.99 (0.07) | 0.92 | 1087 | 1.07 (0.11) | 0.52 | 0.69 (0.11) | 0.02 | 1.04 (0.13) | 0.78 | **2.14 (0.53)** | **0.002** | **0.83 (0.14)** | **0.26** | **0.52 (0.14)** | **0.01** |
| *t*=fu | 1075 | 1.11 (0.04) | 0.008 | 0.72 (0.11) | 0.03 | 1.05 (0.06) | 0.38 | 1087 | 1.19 (0.09) | 0.03 | 0.78 (0.12) | 0.11 | 0.97 (0.10) | 0.75 | 1.42 (0.15) | 0.001 | 0.86 (0.14) | 0.37 | 0.84 (0.12) | 0.23 |

IV: Independent variable; DV: Dependent variable; Ave: average of peer group (*i*); M: moderator (Intervention: 1=ASSIST; 2=Dead Cool); Percent: percentage of peer group (*-i*) classified as susceptible to commencing smoking; OR: odds ratio; SE: standard error.

^a^In each model the outcome variable is the focal participant’s (*i*) response to the relevant item at follow-up. The predictor variable is the average of the relevant group’s (*-i*) responses to the equivalent item at baseline (*t*=base) or follow-up (*t*=fu), where *–i*=(1) focal participant's nominated friends; (2) focal participant’s school class; (3) focal participant’s school year group. The moderator, and interaction of the moderator with the predictor variable, were also included as independent variables in all models. All models include robust (Huber White) standard errors specified using Stata’s ‘vce(robust)’ option. The following baseline variables are included as covariates in all models: gender (0=boy; 1=girl/prefer not to say), age (1=12 years or less; 2=13 years; 3=14 years or more), intervention (1=ASSIST; 2=Dead Cool), ethnicity (0=no ethnic minority; 1=ethnic minority), individuals' socio-economic status (NI: 1=NIMDM2017≤296.6; 2=296.6<NIMDM2017≤593.2; 3=NIMDM2017>593.2; Bogotá: 1=Informal settlement/Lowest/Low; 2=Middle-Low/Middle; 3=Middle-High/High), and baseline values of the outcome variable. The predictor variable and baseline values of the outcome variable were mean-centered.

^b^Unstandardized regression coefficients representing the average change in the outcome variable for a one-unit increase in the predictor variable among participants in ASSIST schools.

^c^Unstandardized regression coefficients representing the average change in the outcome variable for participants in Dead Cool schools compared to participants in ASSIST schools among participants who are average on the predictor variable.

^d^Unstandardized regression coefficients representing the average change in the association between the outcome variable and the predictor variable for participants in Dead Cool schools compared to participants in ASSIST schools.

^e^Logistic regressions were run for models including focal participants' smoking susceptibility as the outcome variable, with robust (Huber White) standard errors specified using Stata’s ‘vce(robust)’ option. The predictor variable is the percentage of the relevant group (*-i*) classified as susceptible to commencing smoking at baseline (*t*=base) or follow-up (*t*=fu), where *–i*=(1) focal participant's nominated friends; (2) focal participant’s school class; (3) focal participant’s school year group. The moderator, and interaction of the moderator with the predictor variable, were also included as independent variables in all models. The following baseline variables are included as covariates in all models: gender (0=boy; 1=girl/prefer not to say), age (1=12 years or less; 2=13 years; 3=14 years or more), intervention (1=ASSIST; 2=Dead Cool), ethnicity (0=no ethnic minority; 1=ethnic minority), individuals' socio-economic status (NI: 1=NIMDM2017≤296.6; 2=296.6<NIMDM2017≤593.2; 3=NIMDM2017>593.2; Bogotá: 1=Informal settlement/Lowest/Low; 2=Middle-Low/Middle; 3=Middle-High/High), and baseline values of the outcome variable. Results are odds ratios, standard errors, and p-values. The predictor variable was mean-centered.

^f^Odds ratios representing the multiplicative change in odds of being susceptible to commencing smoking for a 10% increase in the number of nominated friends/pupils in the same school class/pupils in the same school year group classified as being susceptible to commencing smoking (1 out of 10 nominated friends/pupils in the same school class/pupils in the same school year group; predictor variable) among participants in ASSIST schools.

^g^Odds ratios representing the multiplicative change in odds of being susceptible to commencing smoking for participants in Dead Cool schools compared to participants in ASSIST schools among participants who are average on the predictor variable.

^h^Ratio of ratios representing the ratio of: (1) the odds ratio representing the multiplicative change in odds of being susceptible to commencing smoking for a 10% increase in the number of nominated friends/pupils in the same school class/pupils in the same school year group classified as being susceptible to commencing smoking among participants in Dead Cool schools; to (2) the odds ratio representing the multiplicative change in odds of being susceptible to commencing smoking for a 10% increase in the number of nominated friends/pupils in the same school class/pupils in the same school year group classified as being susceptible to commencing smoking among participants in ASSIST schools.

**Table S2.3.** Results of ordinary least squares linear regressions including interaction terms examining differences in peer influence effects according to gender (boys compared to girls/prefer not to say) for outcomes collected at follow-up.

|  | **Dependent variable: Participant responses to the outcome variable at follow-up^a^** | | | | | | | | | | | | | | | | | | | |
| --- | --- | --- | --- | --- | --- | --- | --- | --- | --- | --- | --- | --- | --- | --- | --- | --- | --- | --- | --- | --- |
|  | **(1) -i=Average of nominated friends** | | | | | | | n | **(2) -i=Average of school class** | | | | | | **(3) -i=Average of school year group** | | | | | |
| **IV** | n | **Ave_-i,t_^b^** | | **M^c^** | | **M*Ave_-i,t_^d^** | |  | **Ave_-i,t_^b^** | | **M^c^** | | **M*Ave_-i,t_^d^** | | **Ave_-i,t_^b^** | | **M^c^** | | **M*Ave_-i,t_^d^** | |
| **DV** |  | *b* (SE) | p-value | *b* (SE) | p-value | *b* (SE) | p-value |  | *b* (SE) | p-value | *b* (SE) | p-value | *b* (SE) | p-value | *b* (SE) | p-value | *b* (SE) | p-value | *b* (SE) | p-value |
| **P2S2** | | | | | | | | | | | | | | | | | | | | |
| *t*=base | 1073 | 0.34 (0.11) | 0.002 | 0.02 (0.02) | 0.30 | 0.005 (0.16) | 0.98 | 1087 | 0.36 (0.15) | 0.02 | 0.02 (0.02) | 0.18 | 0.15 (0.21) | 0.49 | 0.84 (0.22) | <0.001 | 0.02 (0.02) | 0.17 | -0.10 (0.30) | 0.73 |
| *t*=fu | 1018 | 0.11 (0.07) | 0.11 | 0.02 (0.02) | 0.26 | 0.10 (0.12) | 0.42 | 1087 | 0.33 (0.14) | 0.02 | 0.02 (0.02) | 0.18 | -0.05 (0.19) | 0.80 | 0.53 (0.22) | 0.02 | 0.02 (0.02) | 0.22 | 0.07 (0.30) | 0.83 |
| **P2S3** | | | | | | | | | | | | | | | | | | | | |
| *t*=base | 1072 | 0.12 (0.11) | 0.27 | 0.008 (0.02) | 0.71 | -0.008 (0.14) | 0.95 | 1086 | -0.07 (0.23) | 0.75 | 0.004 (0.02) | 0.83 | 0.52 (0.29) | 0.08 | -0.19 (0.39) | 0.62 | 0.005 (0.02) | 0.81 | 0.42 (0.49) | 0.40 |
| *t*=fu | 1017 | 0.03 (0.10) | 0.77 | 0.009 (0.02) | 0.65 | 0.18 (0.13) | 0.16 | 1086 | -0.02 (0.22) | 0.94 | 0.006 (0.02) | 0.78 | 0.47 (0.27) | 0.08 | -0.07 (0.36) | 0.84 | 0.005 (0.02) | 0.80 | 0.53 (0.50) | 0.29 |
| **P2S4** | | | | | | | | | | | | | | | | | | | | |
| *t*=base | 1070 | 0.03 (0.11) | 0.80 | 0.01 (0.02) | 0.56 | 0.20 (0.15) | 0.18 | 1084 | -0.09 (0.17) | 0.62 | 0.007 (0.02) | 0.71 | 0.48 (0.23) | 0.03 | 0.13 (0.46) | 0.77 | 0.007 (0.02) | 0.72 | 0.53 (0.47) | 0.26 |
| *t*=fu | 1016 | 0.15 (0.10) | 0.12 | 0.007 (0.02) | 0.74 | -0.07 (0.13) | 0.59 | 1084 | 0.24 (0.17) | 0.15 | 0.01 (0.02) | 0.62 | 0.09 (0.20 | 0.65 | 0.30 (0.26) | 0.25 | 0.008 (0.02) | 0.69 | 0.25 (0.30) | 0.42 |
| **P2S5** | | | | | | | | | | | | | | | | | | | | |
| *t*=base | 1073 | 0.19 (0.09) | 0.03 | -0.04 (0.02) | 0.13 | 0.01 (0.12) | 0.91 | 1087 | 0.21 (0.15) | 0.15 | -0.05 (0.02) | 0.03 | 0.20 (0.18) | 0.27 | 0.36 (0.21) | 0.09 | -0.05 (0.03) | 0.04 | 0.24 (0.26) | 0.35 |
| *t*=fu | 1018 | 0.13 (0.09) | 0.14 | -0.03 (0.02) | 0.19 | 0.07 (0.11) | 0.53 | 1087 | 0.41 (0.14) | 0.003 | -0.04 (0.02) | 0.06 | 0.09 (0.17) | 0.59 | 0.37 (0.19) | 0.05 | -0.05 (0.02) | 0.05 | 0.16 (0.23) | 0.48 |
| **P2S6** | | | | | | | | | | | | | | | | | | | | |
| *t*=base | 1070 | 0.13 (0.10) | 0.17 | 0.06 (0.02) | 0.02 | 0.06 (0.13) | 0.66 | 1084 | 0.44 (0.16) | 0.006 | 0.07 (0.02) | 0.008 | -0.13 (0.20) | 0.53 | 0.34 (0.25) | 0.18 | 0.06 (0.03) | 0.02 | 0.12 (0.30) | 0.69 |
| *t*=fu | 1015 | 0.12 (0.08) | 0.12 | 0.06 (0.03) | 0.02 | 0.07 (0.11) | 0.52 | 1084 | 0.36 (0.14) | 0.01 | 0.06 (0.02) | 0.01 | 0.16 (0.19) | 0.38 | 0.29 (0.22) | 0.19 | 0.06 (0.03) | 0.02 | 0.38 (0.27) | 0.17 |
| **P2S7** | | | | | | | | | | | | | | | | | | | | |
| *t*=base | 1072 | 0.09 (0.09) | 0.32 | 0.03 (0.02) | 0.21 | 0.13 (0.11) | 0.22 | 1086 | 0.27 (0.13) | 0.04 | 0.03 (0.02) | 0.26 | 0.03 (0.16) | 0.86 | 0.11 (0.20) | 0.60 | 0.02 (0.02) | 0.40 | 0.31 (0.24) | 0.19 |
| *t*=fu | 1018 | 0.29 (0.08) | <0.001 | 0.02 (0.02) | 0.31 | -0.04 (0.11) | 0.73 | 1086 | 0.41 (0.14) | 0.003 | 0.03 (0.02) | 0.17 | -0.01 (0.17) | 0.95 | 0.02 (0.24) | 0.94 | 0.02 (0.02) | 0.43 | 0.53 (0.27) | 0.05 |
| **P2S8** | | | | | | | | | | | | | | | | | | | | |
| *t*=base | 1073 | 0.31 (0.08) | <0.001 | 0.05 (0.02) | 0.03 | -0.02 (0.10) | 0.85 | 1087 | 0.38 (0.12) | 0.002 | 0.06 (0.02) | 0.007 | -0.14 (0.16) | 0.38 | 0.59 (0.20) | 0.003 | 0.06 (0.02) | 0.005 | -0.24 (0.25) | 0.34 |
| *t*=fu | 1018 | 0.15 (0.07) | 0.04 | 0.05 (0.02) | 0.02 | 0.16 (0.11) | 0.13 | 1087 | 0.42 (0.12) | <0.001 | 0.05 (0.02) | 0.01 | 0.09 (0.15) | 0.54 | 0.41 (0.20) | 0.04 | 0.05 (0.02) | 0.02 | 0.13 (0.24) | 0.58 |
| **P2S9** | | | | | | | | | | | | | | | | | | | | |
| *t*=base | 1072 | 0.04 (0.09) | 0.66 | 0.009 (0.02) | 0.66 | 0.23 (0.14) | 0.09 | 1086 | 0.24 (0.19) | 0.22 | 0.006 (0.02) | 0.75 | 0.51 (0.25) | 0.05 | 0.15 (0.32) | 0.64 | 0.004 (0.02) | 0.83 | 0.79 (0.44) | 0.07 |
| *t*=fu | 1017 | 0.17 (0.09) | 0.07 | 0.02 (0.02) | 0.45 | 0.15 (0.13) | 0.27 | **1086** | **0.08 (0.17)** | **0.64** | **-0.003 (0.02)** | **0.87** | **0.64 (0.21)** | **0.003** | -0.16 (0.29) | 0.59 | -0.003 (0.02) | 0.89 | 0.72 (0.40) | 0.07 |
| **Experiment Part 2: Injunctive norms (Average P2S2 to P2S9)** | | | | | | | | | | | | | | | | | | | | |
| *t*=base | 1064 | 0.18 (0.09) | 0.05 | 0.02 (0.01) | 0.13 | 0.08 (0.11) | 0.47 | 1078 | 0.25 (0.15) | 0.11 | 0.02 (0.01) | 0.14 | 0.19 (0.19) | 0.34 | 0.17 (0.21) | 0.43 | 0.02 (0.01) | 0.12 | 0.43 (0.27) | 0.11 |
| *t*=fu | 1011 | 0.21 (0.08) | 0.008 | 0.02 (0.01) | 0.16 | 0.08 (0.11) | 0.45 | 1078 | 0.37 (0.13) | 0.004 | 0.02 (0.01) | 0.10 | 0.19 (0.16) | 0.23 | -0.006 (0.21) | 0.98 | 0.02 (0.01) | 0.09 | 0.55 (0.26) | 0.04 |
| **P3Q1** | | | | | | | | | | | | | | | | | | | | |
| *t*=base | 1073 | -0.02 (0.09) | 0.79 | 0.07 (0.03) | 0.03 | 0.23 (0.12) | 0.06 | 1087 | 0.12 (0.16) | 0.45 | 0.06 (0.03) | 0.04 | 0.21 (0.20) | 0.29 | 0.26 (0.22) | 0.23 | 0.08 (0.03) | 0.01 | -0.21 (0.26) | 0.44 |
| *t*=fu | **1018** | **-0.11 (0.09)** | **0.19** | **0.06 (0.03)** | **0.04** | **0.43 (0.12)** | **<0.001** | 1087 | 0.12 (0.15) | 0.43 | 0.05 (0.03) | 0.13 | 0.34 (0.20) | 0.09 | 0.21 (0.25) | 0.40 | 0.07 (0.03) | 0.03 | -0.03 (0.32) | 0.92 |
| **P3Q2** | | | | | | | | | | | | | | | | | | | | |
| *t*=base | 1073 | 0.09 (0.08) | 0.26 | 0.07 (0.03) | 0.02 | 0.20 (0.10) | 0.05 | 1087 | 0.17 (0.12) | 0.16 | 0.05 (0.03) | 0.08 | 0.25 (0.15) | 0.09 | 0.28 (0.15) | 0.07 | 0.06 (0.03) | 0.04 | 0.07 (0.19) | 0.71 |
| *t*=fu | **1018** | **-0.009 (0.09)** | **0.92** | **0.07 (0.03)** | **0.02** | **0.34 (0.11)** | **0.003** | 1087 | 0.13 (0.13) | 0.30 | 0.04 (0.03) | 0.17 | 0.32 (0.16) | 0.04 | 0.22 (0.18) | 0.22 | 0.06 (0.03) | 0.06 | 0.11 (0.22) | 0.61 |
| **Experiment Part 3: Descriptive norms (Average P3Q1 to P3Q2)** | | | | | | | | | | | | | | | | | | | | |
| *t*=base | 1073 | 0.05 (0.08) | 0.55 | 0.07 (0.03) | 0.02 | 0.19 (0.11) | 0.07 | 1087 | 0.15 (0.14) | 0.28 | 0.06 (0.03) | 0.02 | 0.20 (0.17) | 0.23 | 0.23 (0.17) | 0.18 | 0.07 (0.03) | 0.02 | -0.05 (0.20) | 0.81 |
| *t*=fu | **1018** | **-0.08 (0.08)** | **0.35** | **0.07 (0.03)** | **0.01** | **0.39 (0.11)** | **<0.001** | 1087 | 0.11 (0.13) | 0.42 | 0.06 (0.03) | 0.03 | 0.32 (0.17) | 0.06 | 0.16 (0.20) | 0.43 | 0.07 (0.03) | 0.02 | 0.03 (0.25) | 0.91 |
| **Donation to ASSIST/Dead Cool** | | | | | | | | | | | | | | | | | | | | |
| *t*=base | 1071 | 0.12 (0.08) | 0.12 | 0.11 (0.15) | 0.48 | 0.04 (0.11) | 0.70 | 1085 | 0.27 (0.13) | 0.04 | 0.13 (0.15) | 0.37 | -0.24 (0.18) | 0.18 | 0.53 (0.30) | 0.08 | 0.13 (0.15) | 0.41 | -0.51 (0.37) | 0.17 |
| *t*=fu | 1016 | 0.28 (0.09) | 0.001 | 0.04 (0.15) | 0.81 | 0.11 (0.11) | 0.32 | 1085 | 0.49 (0.12) | <0.001 | 0.14 (0.15) | 0.36 | -0.01 (0.17) | 0.94 | 0.86 (0.21) | <0.001 | 0.10 (0.15) | 0.52 | -0.38 (0.26) | 0.15 |
| **IN1** | | | | | | | | | | | | | | | | | | | | |
| *t*=base | 1073 | -0.02 (0.08) | 0.78 | -0.05 (0.04) | 0.26 | -0.04 (0.13) | 0.77 | 1082 | -0.002 (0.15) | 0.99 | -0.04 (0.04) | 0.30 | 0.10 (0.26) | 0.71 | **0.94 (0.28)** | **0.001** | **-0.04 (0.04)** | **0.29** | **-1.12 (0.44)** | **0.01** |
| *t*=fu | 1070 | 0.14 (0.08) | 0.09 | -0.05 (0.04) | 0.26 | -0.14 (0.12) | 0.24 | 1082 | 0.03 (0.16) | 0.87 | -0.05 (0.04) | 0.27 | -0.41 (0.24) | 0.09 | 0.55 (0.27) | 0.04 | -0.05 (0.04) | 0.25 | -0.68 (0.46) | 0.14 |
| **IN2** | | | | | | | | | | | | | | | | | | | | |
| *t*=base | 1075 | -0.05 (0.08) | 0.57 | -0.006 (0.03) | 0.81 | 0.06 (0.14) | 0.66 | 1084 | 0.16 (0.22) | 0.47 | -0.007 (0.03) | 0.80 | 0.09 (0.33) | 0.78 | -0.16 (0.62) | 0.80 | -0.009 (0.03) | 0.75 | 0.81 (0.68) | 0.23 |
| *t*=fu | 1072 | 0.10 (0.09) | 0.28 | -0.007 (0.03) | 0.81 | 0.09 (0.15) | 0.55 | 1084 | 0.20 (0.14) | 0.17 | -0.006 (0.03) | 0.81 | -0.16 (0.20) | 0.44 | 0.08 (0.34) | 0.81 | -0.006 (0.03) | 0.81 | -0.0004 (0.45) | 1.00 |
| **IN3** | | | | | | | | | | | | | | | | | | | | |
| *t*=base | **1073** | **0.31 (0.11)** | **0.006** | **-0.02 (0.04)** | **0.56** | **-0.32 (0.13)** | **0.01** | 1082 | 0.18 (0.15) | 0.22 | -0.03 (0.04) | 0.33 | -0.01 (0.20) | 0.96 | 0.28 (0.22) | 0.19 | -0.03 (0.04) | 0.34 | -0.04 (0.27) | 0.89 |
| *t*=fu | 1070 | 0.15 (0.08) | 0.05 | -0.03 (0.04) | 0.34 | -0.13 (0.11) | 0.22 | 1082 | 0.05 (0.12) | 0.71 | -0.03 (0.04) | 0.36 | -0.09 (0.18) | 0.63 | 0.29 (0.24) | 0.23 | -0.03 (0.04) | 0.37 | -0.32 (0.32) | 0.32 |
| **IN4** | | | | | | | | | | | | | | | | | | | | |
| *t*=base | 1071 | 0.11 (0.08) | 0.18 | -0.03 (0.05) | 0.51 | -0.16 (0.11) | 0.16 | 1081 | 0.07 (0.18) | 0.69 | -0.04 (0.05) | 0.41 | -0.18 (0.25) | 0.46 | 1.05 (0.40) | 0.009 | -0.06 (0.05) | 0.23 | -0.70 (0.49) | 0.15 |
| *t*=fu | 1069 | 0.14 (0.08) | 0.08 | -0.04 (0.05) | 0.43 | -0.15 (0.11) | 0.15 | 1081 | 0.14 (0.16) | 0.38 | -0.04 (0.05) | 0.45 | 0.06 (0.22) | 0.78 | 0.34 (0.30) | 0.26 | -0.04 (0.05) | 0.38 | 0.15 (0.40) | 0.71 |
| **IN5** | | | | | | | | | | | | | | | | | | | | |
| *t*=base | 1073 | -0.004 (0.08) | 0.96 | -0.03 (0.05) | 0.57 | -0.02 (0.11) | 0.87 | 1082 | 0.12 (0.15) | 0.44 | -0.03 (0.05) | 0.45 | -0.34 (0.21) | 0.10 | 0.40 (0.30) | 0.18 | -0.04 (0.04) | 0.36 | -0.79 (0.37) | 0.03 |
| *t*=fu | 1070 | 0.10 (0.09) | 0.26 | -0.03 (0.05) | 0.50 | -0.13 (0.12) | 0.27 | 1082 | 0.20 (0.17) | 0.23 | -0.03 (0.05) | 0.57 | -0.31 (0.22) | 0.16 | **0.61 (0.31)** | **0.05** | **-0.03 (0.05)** | **0.55** | **-0.94 (0.38)** | **0.01** |
| **IN6** | | | | | | | | | | | | | | | | | | | | |
| *t*=base | 1074 | 0.27 (0.08) | 0.001 | 0.03 (0.05) | 0.51 | -0.12 (0.12) | 0.29 | 1083 | 0.21 (0.15) | 0.16 | 0.04 (0.05) | 0.41 | -0.02 (0.19) | 0.93 | 0.79 (0.23) | 0.001 | 0.04 (0.05) | 0.39 | -0.66 (0.29) | 0.02 |
| *t*=fu | 1071 | 0.27 (0.08) | <0.001 | 0.03 (0.05) | 0.61 | -0.06 (0.11) | 0.59 | 1083 | 0.34 (0.13) | 0.008 | 0.04 (0.05) | 0.40 | -0.13 (0.17) | 0.43 | 0.74 (0.22) | 0.001 | 0.04 (0.05) | 0.41 | -0.62 (0.27) | 0.02 |
| **IN7** | | | | | | | | | | | | | | | | | | | | |
| *t*=base | 1075 | 0.21 (0.08) | 0.02 | 0.03 (0.05) | 0.46 | -0.18 (0.11) | 0.10 | 1084 | 0.26 (0.17) | 0.13 | 0.04 (0.05) | 0.36 | -0.10 (0.22) | 0.64 | 0.74 (0.24) | 0.002 | 0.05 (0.05) | 0.32 | -0.35 (0.30) | 0.24 |
| *t*=fu | 1072 | 0.14 (0.08) | 0.10 | 0.02 (0.05) | 0.63 | 0.12 (0.12) | 0.33 | 1084 | 0.31 (0.16) | 0.05 | 0.04 (0.05) | 0.39 | -0.19 (0.20) | 0.33 | 0.48 (0.23) | 0.04 | 0.04 (0.05) | 0.39 | -0.27 (0.28) | 0.33 |
| **Self-report injunctive norms (Average IN1 to IN7)** | | | | | | | | | | | | | | | | | | | | |
| *t*=base | 1068 | 0.19 (0.08) | 0.02 | -0.02 (0.03) | 0.53 | -0.24 (0.11) | 0.04 | 1078 | 0.13 (0.16) | 0.42 | -0.02 (0.03) | 0.51 | -0.14 (0.21) | 0.51 | **0.78 (0.24)** | **0.001** | **-0.02 (0.03)** | **0.48** | **-0.76 (0.30)** | **0.01** |
| *t*=fu | 1066 | 0.23 (0.08) | 0.002 | -0.02 (0.03) | 0.40 | -0.09 (0.11) | 0.41 | 1078 | 0.35 (0.13) | 0.007 | -0.02 (0.03) | 0.53 | -0.33 (0.17) | 0.05 | **0.65 (0.22)** | **0.003** | **-0.02 (0.03)** | **0.47** | **-0.77 (0.27)** | **0.005** |
| **DN1.1** | | | | | | | | | | | | | | | | | | | | |
| *t*=base | 1075 | 0.22 (0.15) | 0.14 | -0.03 (0.04) | 0.46 | 0.17 (0.20) | 0.41 | 1084 | 0.22 (0.17) | 0.20 | -0.03 (0.04) | 0.42 | 0.15 (0.27) | 0.58 | 0.91 (0.40) | 0.02 | -0.03 (0.04) | 0.53 | 0.26 (0.45) | 0.56 |
| *t*=fu | 1072 | 0.23 (0.11) | 0.03 | -0.02 (0.04) | 0.55 | 0.23 (0.17) | 0.18 | 1084 | 0.04 (0.18) | 0.84 | -0.03 (0.04) | 0.42 | 0.38 (0.26) | 0.13 | 0.36 (0.26) | 0.17 | -0.02 (0.04) | 0.52 | 0.46 (0.34) | 0.18 |
| **DN1.2** | | | | | | | | | | | | | | | | | | | | |
| *t*=base | 1076 | 0.16 (0.07) | 0.03 | -0.04 (0.04) | 0.31 | -0.07 (0.09) | 0.42 | 1085 | 0.07 (0.10) | 0.50 | -0.05 (0.04) | 0.16 | 0.01 (0.12) | 0.90 | 0.19 (0.12) | 0.10 | -0.05 (0.04) | 0.15 | -0.06 (0.13) | 0.66 |
| *t*=fu | 1073 | -0.05 (0.08) | 0.51 | -0.05 (0.04) | 0.18 | 0.09 (0.09) | 0.32 | 1085 | 0.03 (0.12) | 0.77 | -0.05 (0.04) | 0.17 | -0.02 (0.14) | 0.88 | 0.13 (0.12) | 0.30 | -0.05 (0.04) | 0.15 | -0.05 (0.15) | 0.75 |
| **DN1.3** | | | | | | | | | | | | | | | | | | | | |
| *t*=base | 1075 | 0.03 (0.06) | 0.68 | -0.06 (0.05) | 0.24 | 0.12 (0.09) | 0.17 | 1084 | 0.008 (0.12) | 0.95 | -0.05 (0.05) | 0.26 | 0.10 (0.15) | 0.49 | 0.02 (0.18) | 0.90 | -0.05 (0.05) | 0.26 | 0.20 (0.20) | 0.34 |
| *t*=fu | 1072 | 0.07 (0.08) | 0.34 | -0.05 (0.05) | 0.32 | -0.10 (0.10) | 0.32 | 1084 | -0.02 (0.12) | 0.84 | -0.05 (0.05) | 0.26 | 0.10 (0.15) | 0.50 | -0.03 (0.21) | 0.89 | -0.05 (0.05) | 0.26 | 0.20 (0.24) | 0.41 |
| **DN1.4** | | | | | | | | | | | | | | | | | | | | |
| *t*=base | 1075 | -0.02 (0.07) | 0.81 | 0.05 (0.04) | 0.21 | 0.06 (0.09) | 0.52 | 1084 | 0.17 (0.22) | 0.43 | 0.04 (0.04) | 0.28 | -0.18 (0.27) | 0.51 | 0.13 (0.18) | 0.47 | 0.04 (0.04) | 0.31 | -0.05 (0.27) | 0.86 |
| *t*=fu | 1072 | -0.11 (0.06) | 0.06 | 0.04 (0.04) | 0.25 | 0.06 (0.08) | 0.51 | 1084 | 0.26 (0.26) | 0.32 | 0.04 (0.04) | 0.27 | -0.32 (0.29) | 0.27 | -0.04 (0.25) | 0.88 | 0.04 (0.04) | 0.28 | -0.12 (0.31) | 0.71 |
| **DN1.5** | | | | | | | | | | | | | | | | | | | | |
| *t*=base | 1075 | 0.14 (0.08) | 0.09 | -0.04 (0.03) | 0.16 | -0.14 (0.09) | 0.13 | 1084 | 0.34 (0.20) | 0.09 | -0.03 (0.03) | 0.23 | -0.34 (0.24) | 0.15 | 0.29 (0.24) | 0.23 | -0.04 (0.03) | 0.21 | -0.01 (0.39) | 0.97 |
| *t*=fu | 1072 | 0.003 (0.06) | 0.96 | -0.03 (0.03) | 0.24 | -0.03 (0.10) | 0.75 | 1084 | 0.30 (0.18) | 0.09 | -0.04 (0.03) | 0.15 | -0.24 (0.22) | 0.29 | 0.15 (0.19) | 0.43 | -0.03 (0.03) | 0.19 | -0.12 (0.27) | 0.66 |
| **Self-report descriptive norms 1 (Average DN1.1 to DN1.5)** | | | | | | | | | | | | | | | | | | | | |
| *t*=base | 1073 | 0.10 (0.07) | 0.16 | -0.02 (0.02) | 0.35 | -0.02 (0.10) | 0.85 | 1082 | 0.05 (0.12) | 0.67 | -0.02 (0.02) | 0.26 | 0.005 (0.13) | 0.97 | 0.19 (0.14) | 0.19 | -0.02 (0.02) | 0.25 | -0.02 (0.15) | 0.89 |
| *t*=fu | 1070 | 0.05 (0.06) | 0.46 | -0.02 (0.02) | 0.37 | -0.02 (0.09) | 0.81 | 1082 | 0.006 (0.12) | 0.96 | -0.02 (0.02) | 0.26 | 0.02 (0.14) | 0.86 | 0.11 (0.15) | 0.45 | -0.02 (0.02) | 0.25 | 0.05 (0.16) | 0.75 |
| **DN2.1** | | | | | | | | | | | | | | | | | | | | |
| *t*=base | 1076 | 0.22 (0.11) | 0.05 | -0.06 (0.04) | 0.15 | -0.09 (0.14) | 0.53 | 1085 | 0.08 (0.13) | 0.57 | -0.07 (0.04) | 0.07 | 0.17 (0.20) | 0.38 | 0.21 (0.20) | 0.29 | -0.07 (0.04) | 0.08 | 0.002 (0.26) | 0.99 |
| *t*=fu | 1073 | 0.24 (0.10) | 0.02 | -0.06 (0.04) | 0.12 | -0.10 (0.13) | 0.45 | 1085 | 0.12 (0.15) | 0.43 | -0.07 (0.04) | 0.06 | 0.12 (0.21) | 0.56 | 0.12 (0.22) | 0.58 | -0.07 (0.04) | 0.07 | -0.004 (0.26) | 0.99 |
| **DN2.2** | | | | | | | | | | | | | | | | | | | | |
| *t*=base | 1076 | 0.06 (0.08) | 0.43 | -0.04 (0.05) | 0.39 | 0.23 (0.12) | 0.05 | **1085** | **0.07 (0.12)** | **0.54** | **-0.05 (0.05)** | **0.32** | **0.45 (0.17)** | **0.008** | 0.31 (0.20) | 0.13 | -0.06 (0.05) | 0.25 | 0.33 (0.25) | 0.19 |
| *t*=fu | 1073 | 0.09 (0.09) | 0.33 | -0.04 (0.05) | 0.47 | 0.04 (0.12) | 0.72 | 1085 | -0.01 (0.13) | 0.92 | -0.05 (0.05) | 0.32 | 0.35 (0.17) | 0.04 | 0.21 (0.20) | 0.29 | -0.05 (0.05) | 0.30 | 0.32 (0.24) | 0.18 |
| **DN2.3** | | | | | | | | | | | | | | | | | | | | |
| *t*=base | 1076 | 0.30 (0.14) | 0.04 | -0.04 (0.04) | 0.31 | -0.05 (0.16) | 0.76 | **1085** | **0.36 (0.14)** | **0.008** | **-0.05 (0.03)** | **0.17** | **0.40 (0.16)** | **0.01** | **0.33 (0.16)** | **0.05** | **-0.05 (0.03)** | **0.13** | **0.57 (0.21)** | **0.007** |
| *t*=fu | 1073 | 0.13 (0.10) | 0.19 | -0.06 (0.04) | 0.11 | 0.10 (0.11) | 0.38 | **1085** | **0.20 (0.12)** | **0.10** | **-0.05 (0.03)** | **0.14** | **0.36 (0.14)** | **0.01** | **0.23 (0.15)** | **0.12** | **-0.05 (0.03)** | **0.14** | **0.55 (0.19)** | **0.004** |
| **Self-report descriptive norms 2 (Average DN2.1 to DN2.3)** | | | | | | | | | | | | | | | | | | | | |
| *t*=base | 1076 | 0.11 (0.10) | 0.24 | -0.04 (0.03) | 0.23 | 0.20 (0.12) | 0.09 | **1085** | **0.12 (0.13)** | **0.35** | **-0.05 (0.03)** | **0.08** | **0.45 (0.17)** | **0.008** | 0.23 (0.19) | 0.21 | -0.05 (0.03) | 0.08 | 0.36 (0.22) | 0.11 |
| *t*=fu | 1073 | 0.03 (0.09) | 0.73 | -0.05 (0.03) | 0.14 | 0.16 (0.11) | 0.15 | 1085 | 0.07 (0.12) | 0.56 | -0.05 (0.03) | 0.08 | 0.33 (0.15) | 0.03 | 0.21 (0.20) | 0.29 | -0.06 (0.05) | 0.26 | 0.32 (0.24) | 0.18 |
| **Self-report smoking behavior** | | | | | | | | | | | | | | | | | | | | |
| *t*=base | 1083 | 0.11 (0.07) | 0.14 | 0.03 (0.03) | 0.28 | 0.10 (0.13) | 0.44 | 1093 | 0.13 (0.15) | 0.36 | 0.04 (0.03) | 0.26 | 0.16 (0.19) | 0.42 | 0.42 (0.24) | 0.08 | 0.04 (0.03) | 0.25 | 0.25 (0.26) | 0.34 |
| *t*=fu | 1081 | 0.18 (0.07) | 0.01 | 0.02 (0.03) | 0.45 | 0.16 (0.13) | 0.21 | 1093 | 0.13 (0.12) | 0.27 | 0.03 (0.03) | 0.27 | 0.17 (0.16) | 0.29 | 0.31 (0.15) | 0.05 | 0.03 (0.03) | 0.25 | 0.14 (0.19) | 0.45 |
| **Intentions** | | | | | | | | | | | | | | | | | | | | |
| *t*=base | 1078 | 0.07 (0.12) | 0.54 | -0.03 (0.07) | 0.62 | 0.18 (0.16) | 0.24 | 1087 | 0.53 (0.21) | 0.01 | -0.04 (0.07) | 0.56 | -0.09 (0.28) | 0.76 | 1.03 (0.35) | 0.004 | -0.03 (0.08) | 0.69 | -0.14 (0.47) | 0.77 |
| *t*=fu | **1075** | **0.06 (0.08)** | **0.43** | **-0.04 (0.07)** | **0.58** | **0.39 (0.15)** | **0.007** | 1087 | 0.08 (0.15) | 0.61 | -0.05 (0.07) | 0.48 | 0.06 (0.21) | 0.77 | 0.37 (0.18) | 0.05 | -0.04 (0.07) | 0.56 | 0.02 (0.26) | 0.92 |
| **Knowledge** | | | | | | | | | | | | | | | | | | | | |
| *t*=base | 1080 | 0.24 (0.07) | 0.002 | 0.15 (0.08) | 0.07 | -0.08 (0.10) | 0.44 | 1089 | 0.41 (0.10) | <0.001 | 0.13 (0.08) | 0.10 | 0.002 (0.13) | 0.99 | 0.48 (0.14) | 0.001 | 0.14 (0.08) | 0.08 | -0.06 (0.18) | 0.73 |
| *t*=fu | 1077 | 0.26 (0.07) | 0.001 | 0.11 (0.08) | 0.17 | 0.06 (0.10) | 0.56 | 1089 | 0.42 (0.10) | <0.001 | 0.14 (0.08) | 0.08 | -0.02 (0.12) | 0.88 | 0.46 (0.13) | 0.001 | 0.16 (0.08) | 0.05 | -0.08 (0.16) | 0.63 |
| **Attitudes** | | | | | | | | | | | | | | | | | | | | |
| *t*=base | 1067 | 0.13 (0.09) | 0.16 | -0.04 (0.04) | 0.30 | 0.05 (0.11) | 0.66 | 1076 | -0.02 (0.16) | 0.92 | -0.04 (0.04) | 0.29 | -0.04 (0.20) | 0.84 | 0.44 (0.30) | 0.14 | -0.03 (0.04) | 0.41 | -0.19 (0.40) | 0.63 |
| *t*=fu | 1064 | 0.13 (0.10) | 0.21 | -0.03 (0.04) | 0.37 | 0.09 (0.12) | 0.47 | 1076 | -0.06 (0.18) | 0.73 | -0.04 (0.04) | 0.27 | -0.07 (0.22) | 0.75 | 0.21 (0.25) | 0.40 | -0.04 (0.04) | 0.32 | 0.03 (0.30) | 0.93 |
| **Self-efficacy (Emotional)** | | | | | | | | | | | | | | | | | | | | |
| *t*=base | 1072 | 0.18 (0.11) | 0.09 | -0.08 (0.05) | 0.08 | -0.13 (0.14) | 0.37 | 1081 | 0.26 (0.17) | 0.12 | -0.09 (0.05) | 0.06 | 0.09 (0.22) | 0.69 | 0.41 (0.21) | 0.06 | -0.09 (0.05) | 0.06 | 0.11 (0.29) | 0.70 |
| *t*=fu | 1068 | 0.25 (0.09) | 0.006 | -0.08 (0.05) | 0.11 | 0.06 (0.13) | 0.62 | 1081 | 0.25 (0.13) | 0.05 | -0.09 (0.05) | 0.05 | -0.07 (0.16) | 0.66 | 0.58 (0.18) | 0.001 | -0.09 (0.05) | 0.05 | -0.32 (0.21) | 0.13 |
| **Self-efficacy (Friends)** | | | | | | | | | | | | | | | | | | | | |
| *t*=base | 1078 | 0.11 (0.10) | 0.24 | -0.13 (0.04) | 0.003 | -0.09 (0.12) | 0.45 | 1087 | 0.18 (0.15) | 0.23 | -0.13 (0.04) | 0.003 | 0.03 (0.21) | 0.88 | 0.24 (0.23) | 0.30 | -0.14 (0.04) | 0.002 | -0.11 (0.29) | 0.69 |
| *t*=fu | 1075 | 0.17 (0.09) | 0.07 | -0.11 (0.04) | 0.009 | 0.05 (0.13) | 0.67 | 1087 | 0.15 (0.14) | 0.27 | -0.14 (0.04) | 0.002 | -0.09 (0.19) | 0.63 | 0.44 (0.23) | 0.06 | -0.14 (0.04) | 0.002 | -0.37 (0.30) | 0.21 |
| **Self-efficacy (Opportunity)** | | | | | | | | | | | | | | | | | | | | |
| *t*=base | 1079 | 0.18 (0.11) | 0.11 | -0.03 (0.04) | 0.42 | -0.09 (0.14) | 0.54 | 1088 | 0.39 (0.18) | 0.03 | -0.04 (0.04) | 0.35 | -0.03 (0.25) | 0.92 | 0.54 (0.26) | 0.04 | -0.04 (0.04) | 0.35 | -0.21 (0.32) | 0.51 |
| *t*=fu | 1076 | 0.13 (0.10) | 0.20 | -0.03 (0.04) | 0.41 | 0.09 (0.13) | 0.50 | 1088 | 0.29 (0.14) | 0.04 | -0.04 (0.04) | 0.34 | -0.16 (0.17) | 0.35 | 0.40 (0.22) | 0.07 | -0.04 (0.04) | 0.36 | -0.21 (0.25) | 0.41 |
| **Perceived physical risks** | | | | | | | | | | | | | | | | | | | | |
| *t*=base | 1074 | 0.02 (0.08) | 0.78 | -2.96 (1.21) | 0.01 | 0.10 (0.10) | 0.33 | 1082 | 0.30 (0.11) | 0.009 | -2.98 (1.20) | 0.01 | -0.30 (0.16) | 0.06 | **0.50 (0.20)** | **0.01** | **-2.85 (1.21)** | **0.02** | **-0.82 (0.26)** | **0.002** |
| *t*=fu | 1070 | 0.09 (0.07) | 0.24 | -2.86 (1.20) | 0.02 | 0.05 (0.10) | 0.61 | 1082 | 0.14 (0.14) | 0.29 | -3.07 (1.21) | 0.01 | -0.08 (0.19) | 0.69 | -0.05 (0.27) | 0.86 | -3.04 (1.22) | 0.01 | -0.15 (0.30) | 0.61 |
| **Perceived social risks** | | | | | | | | | | | | | | | | | | | | |
| *t*=base | 1076 | 0.06 (0.07) | 0.41 | -2.71 (1.38) | 0.05 | 0.20 (0.09) | 0.03 | 1085 | 0.28 (0.09) | 0.003 | -2.56 (1.39) | 0.07 | -0.01 (0.13) | 0.93 | 0.56 (0.14) | <0.001 | -2.31 (1.39) | 0.10 | -0.27 (0.16) | 0.10 |
| *t*=fu | 1073 | 0.13 (0.08) | 0.11 | -2.57 (1.38) | 0.06 | 0.007 (0.11) | 0.95 | 1085 | 0.21 (0.11) | 0.04 | -2.52 (1.38) | 0.07 | 0.05 (0.15) | 0.76 | 0.48 (0.16) | 0.002 | -2.51 (1.38) | 0.07 | -0.14 (0.19) | 0.47 |
| **Perceived addiction risks** | | | | | | | | | | | | | | | | | | | | |
| *t*=base | 1016 | 0.10 (0.08) | 0.21 | 1.23 (1.44) | 0.39 | 0.25 (0.10) | 0.02 | 1025 | 0.35 (0.13) | 0.005 | 1.19 (1.42) | 0.40 | 0.27 (0.15) | 0.08 | 0.53 (0.15) | 0.001 | 1.24 (1.41) | 0.38 | 0.31 (0.17) | 0.07 |
| *t*=fu | 1009 | 0.06 (0.07) | 0.44 | 0.93 (1.45) | 0.52 | 0.15 (0.09) | 0.10 | **1025** | **0.17 (0.12)** | **0.15** | **0.78 (1.42)** | **0.58** | **0.39 (0.14)** | **0.005** | 0.40 (0.14) | 0.004 | 0.94 (1.41) | 0.51 | 0.29 (0.16) | 0.07 |
| **Perceived benefits** | | | | | | | | | | | | | | | | | | | | |
| *t*=base | 1029 | -0.15 (0.09) | 0.11 | -1.90 (1.22) | 0.12 | 0.20 (0.12) | 0.10 | 1038 | 0.02 (0.18) | 0.91 | -2.03 (1.22) | 0.10 | 0.13 (0.23) | 0.56 | 0.37 (0.33) | 0.26 | -1.96 (1.22) | 0.11 | -0.06 (0.39) | 0.87 |
| *t*=fu | 1025 | 0.08 (0.09) | 0.36 | -1.88 (1.23) | 0.13 | -0.10 (0.12) | 0.37 | 1038 | -0.006 (0.18) | 0.98 | -1.93 (1.22) | 0.12 | -0.16 (0.24) | 0.52 | -0.31 (0.39) | 0.42 | -2.01 (1.22) | 0.10 | 0.32 (0.49) | 0.52 |
| **Perceived behavioral control (easy to quit)** | | | | | | | | | | | | | | | | | | | | |
| *t*=base | 1076 | 0.19 (0.08) | 0.03 | -0.20 (0.08) | 0.01 | 0.16 (0.11) | 0.14 | 1085 | 0.42 (0.12) | <0.001 | -0.19 (0.08) | 0.02 | 0.15 (0.14) | 0.28 | 0.70 (0.14) | <0.001 | -0.19 (0.08) | 0.02 | -0.03 (0.16) | 0.84 |
| *t*=fu | 1073 | 0.17 (0.08) | 0.03 | -0.16 (0.08) | 0.06 | 0.002 (0.10) | 0.98 | 1085 | 0.51 (0.11) | <0.001 | -0.20 (0.08) | 0.01 | -0.03 (0.13) | 0.82 | 0.62 (0.13) | <0.001 | -0.19 (0.08) | 0.02 | -0.02 (0.16) | 0.87 |
| **Perceived behavioral control (to avoid smoking)** | | | | | | | | | | | | | | | | | | | | |
| *t*=base | 1080 | 0.06 (0.10) | 0.58 | -0.11 (0.07) | 0.11 | 0.01 (0.14) | 0.92 | 1089 | 0.14 (0.19) | 0.47 | -0.11 (0.07) | 0.13 | 0.44 (0.25) | 0.08 | 0.61 (0.29) | 0.04 | -0.10 (0.07) | 0.15 | 0.34 (0.37) | 0.35 |
| *t*=fu | 1077 | 0.06 (0.12) | 0.60 | -0.10 (0.07) | 0.17 | 0.05 (0.14) | 0.73 | 1089 | -0.12 (0.18) | 0.48 | -0.12 (0.07) | 0.10 | 0.49 (0.24) | 0.04 | 0.28 (0.25) | 0.26 | -0.12 (0.07) | 0.09 | 0.48 (0.30) | 0.11 |
| **Objectively measured smoking behavior** | | | | | | | | | | | | | | | | | | | | |
| *t*=base | 1041 | 0.38 (0.06) | <0.001 | -0.06 (0.08) | 0.51 | -0.03 (0.07) | 0.70 | 1048 | 0.43 (0.07) | <0.001 | -0.05 (0.08) | 0.53 | -0.09 (0.07) | 0.17 | 0.62 (0.07) | <0.001 | -0.03 (0.08) | 0.71 | -0.06 (0.07) | 0.41 |
| *t*=fu | 1022 | 0.42 (0.11) | <0.001 | -0.05 (0.08) | 0.49 | 0.14 (0.13) | 0.28 | 1048 | 0.88 (0.07) | <0.001 | -0.03 (0.07) | 0.67 | -0.07 (0.08) | 0.40 | 0.91 (0.08) | <0.001 | -0.05 (0.07) | 0.54 | -0.09 (0.09) | 0.35 |
| **Smoking susceptibility^e^** | | | | | | | | | | | | | | | | | | | | |
|  | n | **Percent_-i,t_^f^** | | **M^g^** | | **M*Percent_-i,t_^h^** | | n | **Percent_-i,t_^f^** | | **M^g^** | | **M*Percent_-i,t_^h^** | | **Percent_-i,t_^f^** | | **M^g^** | | **M*Percent_-i,t_^h^** | |
|  |  | OR (SE) | p-value | OR (SE) | p-value | OR (SE) | p-value |  | OR (SE) | p-value | OR (SE) | p-value | OR (SE) | p-value | OR (SE) | p-value | OR (SE) | p-value | OR (SE) | p-value |
| *t*=base | 1078 | 1.15 (0.06) | 0.009 | 1.41 (0.21) | 0.02 | 0.98 (0.07) | 0.82 | 1087 | 1.06 (0.09) | 0.53 | 1.52 (0.22) | 0.005 | 1.08 (0.13) | 0.53 | 1.23 (0.17) | 0.12 | 1.51 (0.22) | 0.005 | 0.96 (0.17) | 0.84 |
| *t*=fu | 1075 | 1.16 (0.05) | <0.001 | 1.45 (0.22) | 0.01 | 0.95 (0.05) | 0.36 | 1087 | 1.22 (0.08) | 0.005 | 1.52 (0.22) | 0.004 | 0.92 (0.09) | 0.38 | 1.42 (0.14) | <0.001 | 1.54 (0.23) | 0.003 | 0.85 (0.11) | 0.22 |

IV: Independent variable; DV: Dependent variable; Ave: average of peer group (*i*); M: moderator (Gender: 0=boy; 1=girl/prefer not to say); Percent: percentage of peer group (*-i*) classified as susceptible to commencing smoking; OR: odds ratio; SE: standard error.

^a^In each model the outcome variable is the focal participant’s (*i*) response to the relevant item at follow-up. The predictor variable is the average of the relevant group’s (*-i*) responses to the equivalent item at baseline (*t*=base) or follow-up (*t*=fu), where *–i*=(1) focal participant's nominated friends; (2) focal participant’s school class; (3) focal participant’s school year group. The moderator, and interaction of the moderator with the predictor variable, were also included as independent variables in all models. All models include robust (Huber White) standard errors specified using Stata’s ‘vce(robust)’ option. The following baseline variables are included as covariates in all models: gender (0=boy; 1=girl/prefer not to say), age (1=12 years or less; 2=13 years; 3=14 years or more), intervention (1=ASSIST; 2=Dead Cool), ethnicity (0=no ethnic minority; 1=ethnic minority), individuals' socio-economic status (NI: 1=NIMDM2017≤296.6; 2=296.6<NIMDM2017≤593.2; 3=NIMDM2017>593.2; Bogotá: 1=Informal settlement/Lowest/Low; 2=Middle-Low/Middle; 3=Middle-High/High), and baseline values of the outcome variable. The predictor variable and baseline values of the outcome variable were mean-centered.

^b^Unstandardized regression coefficients representing the average change in the outcome variable for a one-unit increase in the predictor variable among boys.

^c^Unstandardized regression coefficients representing the average change in the outcome variable for girls/prefer not to say compared to boys among participants who are average on the predictor variable.

^d^Unstandardized regression coefficients representing the average change in the association between the outcome variable and the predictor variable for girls/prefer not to say compared to boys.

^e^Logistic regressions were run for models including focal participants' smoking susceptibility as the outcome variable, with robust (Huber White) standard errors specified using Stata’s ‘vce(robust)’ option. The predictor variable is the percentage of the relevant group (*-i*) classified as susceptible to commencing smoking at baseline (*t*=base) or follow-up (*t*=fu), where *–i*=(1) focal participant's nominated friends; (2) focal participant’s school class; (3) focal participant’s school year group. The moderator, and interaction of the moderator with the predictor variable, were also included as independent variables in all models. The following baseline variables are included as covariates in all models: gender (0=boy; 1=girl/prefer not to say), age (1=12 years or less; 2=13 years; 3=14 years or more), intervention (1=ASSIST; 2=Dead Cool), ethnicity (0=no ethnic minority; 1=ethnic minority), individuals' socio-economic status (NI: 1=NIMDM2017≤296.6; 2=296.6<NIMDM2017≤593.2; 3=NIMDM2017>593.2; Bogotá: 1=Informal settlement/Lowest/Low; 2=Middle-Low/Middle; 3=Middle-High/High), and baseline values of the outcome variable. Results are odds ratios, standard errors, and p-values. The predictor variable was mean-centered.

^f^Odds ratios representing the multiplicative change in odds of being susceptible to commencing smoking for a 10% increase in the number of nominated friends/pupils in the same school class/pupils in the same school year group classified as being susceptible to commencing smoking (1 out of 10 nominated friends/pupils in the same school class/pupils in the same school year group; predictor variable) among boys.

^g^Odds ratios representing the multiplicative change in odds of being susceptible to commencing smoking for girls/prefer not to say compared to boys among participants who are average on the predictor variable.

^h^Ratio of ratios representing the ratio of: (1) the odds ratio representing the multiplicative change in odds of being susceptible to commencing smoking for a 10% increase in the number of nominated friends/pupils in the same school class/pupils in the same school year group classified as being susceptible to commencing smoking among girls/prefer not to say; to (2) the odds ratio representing the multiplicative change in odds of being susceptible to commencing smoking for a 10% increase in the number of nominated friends/pupils in the same school class/pupils in the same school year group classified as being susceptible to commencing smoking among boys.

**Table S2.4.** Results of ordinary least squares linear regressions including interaction terms examining differences in peer influence effects according to school socio-economic status for outcomes collected at follow-up (all schools).

|  | **Dependent variable: Participant responses to the outcome variable at follow-up^a^** | | | | | | | | | | | | | | | | | | | |
| --- | --- | --- | --- | --- | --- | --- | --- | --- | --- | --- | --- | --- | --- | --- | --- | --- | --- | --- | --- | --- |
|  | **(1) -i=Average of nominated friends** | | | | | | | n | **(2) -i=Average of school class** | | | | | | **(3) -i=Average of school year group** | | | | | |
| **IV** | n | **Ave_-i,t_^b^** | | **M^c^** | | **M*Ave_-i,t_^d^** | |  | **Ave_-i,t_^b^** | | **M^c^** | | **M*Ave_-i,t_^d^** | | **Ave_-i,t_^b^** | | **M^c^** | | **M*Ave_-i,t_^d^** | |
| **DV** |  | *b* (SE) | p-value | *b* (SE) | p-value | *b* (SE) | p-value |  | *b* (SE) | p-value | *b* (SE) | p-value | *b* (SE) | p-value | *b* (SE) | p-value | *b* (SE) | p-value | *b* (SE) | p-value |
| **P2S2** | | | | | | | | | | | | | | | | | | | | |
| *t=base* | 1132 | 0.31 (0.08) | 0.0001 | -0.03 (0.01) | 0.02 | -0.02 (0.09) | 0.81 | 1147 | 0.36 (0.10) | 0.0003 | -0.02 (0.01) | 0.05 | -0.14 (0.12) | 0.24 | 0.66 (0.16) | <0.0001 | -0.02 (0.01) | 0.17 | -0.16 (0.24) | 0.52 |
| *t=fu* | 1072 | 0.15 (0.06) | 0.01 | -0.03 (0.01) | 0.02 | 0.01 (0.08) | 0.92 | 1147 | 0.22 (0.10) | 0.02 | -0.03 (0.01) | 0.04 | 0.07 (0.11) | 0.56 | 0.39 (0.16) | 0.01 | -0.01 (0.01) | 0.31 | -0.19 (0.20) | 0.34 |
| **P2S3** | | | | | | | | | | | | | | | | | | | | |
| *t=base* | 1133 | 0.13 (0.07) | 0.06 | -0.02 (0.01) | 0.17 | -0.06 (0.08) | 0.44 | 1148 | 0.23 (0.15) | 0.12 | -0.03 (0.01) | 0.05 | 0.15 (0.15) | 0.33 | 0.03 (0.27) | 0.90 | -0.01 (0.02) | 0.71 | -0.53 (0.39) | 0.17 |
| *t=fu* | 1072 | 0.13 (0.07) | 0.04 | -0.01 (0.01) | 0.28 | 0.03 (0.08) | 0.73 | 1148 | 0.18 (0.14) | 0.19 | -0.02 (0.01) | 0.20 | 0.06 (0.15) | 0.69 | 0.01 (0.28) | 0.98 | -0.02 (0.01) | 0.20 | 0.42 (0.28) | 0.14 |
| **P2S4** | | | | | | | | | | | | | | | | | | | | |
| *t=base* | 1131 | 0.15 (0.07) | 0.04 | 0.02 (0.01) | 0.17 | 0.02 (0.09) | 0.82 | 1146 | 0.33 (0.13) | 0.01 | 0.02 (0.01) | 0.15 | 0.24 (0.13) | 0.06 | **1.42 (0.40)** | **0.0004** | **0.02 (0.01)** | **0.17** | **1.10 (0.33)** | **0.001** |
| *t=fu* | 1071 | 0.17 (0.06) | 0.01 | 0.01 (0.01) | 0.26 | 0.11 (0.08) | 0.18 | 1146 | 0.32 (0.11) | 0.003 | 0.01 (0.01) | 0.19 | 0.20 (0.14) | 0.15 | 0.38 (0.21) | 0.07 | 0.02 (0.01) | 0.17 | 0.43 (0.32) | 0.19 |
| **P2S5** | | | | | | | | | | | | | | | | | | | | |
| *t=base* | 1134 | 0.21 (0.06) | 0.0002 | 0.02 (0.01) | 0.11 | 0.03 (0.07) | 0.61 | 1149 | 0.36 (0.09) | 0.0001 | 0.03 (0.02) | 0.07 | -0.11 (0.14) | 0.43 | 0.76 (0.19) | <0.0001 | 0.004 (0.02) | 0.86 | 0.47 (0.33) | 0.15 |
| *t=fu* | 1073 | 0.19 (0.05) | 0.0003 | 0.03 (0.02) | 0.07 | 0.05 (0.07) | 0.51 | 1149 | 0.45 (0.08) | <0.0001 | 0.01 (0.02) | 0.56 | 0.13 (0.15) | 0.39 | 0.48 (0.14) | 0.0004 | 0.02 (0.02) | 0.38 | -0.17 (0.38) | 0.66 |
| **P2S6** | | | | | | | | | | | | | | | | | | | | |
| *t=base* | 1130 | 0.18 (0.07) | 0.01 | 0.03 (0.01) | 0.03 | -0.05 (0.07) | 0.41 | 1145 | 0.36 (0.11) | 0.001 | 0.03 (0.01) | 0.01 | -0.07 (0.10) | 0.49 | **0.38 (0.18)** | **0.04** | **0.04 (0.01)** | **0.01** | **-0.58 (0.20)** | **0.003** |
| *t=fu* | 1070 | 0.12 (0.05) | 0.03 | 0.03 (0.01) | 0.03 | 0.04 (0.06) | 0.53 | 1145 | 0.40 (0.10) | <0.0001 | 0.02 (0.01) | 0.20 | 0.11 (0.10) | 0.23 | **0.09 (0.20)** | **0.66** | **0.06 (0.02)** | **0.004** | **-0.81 (0.31)** | **0.01** |
| **P2S7** | | | | | | | | | | | | | | | | | | | | |
| *t=base* | 1132 | 0.19 (0.05) | 0.001 | 0.03 (0.01) | 0.06 | 0.01 (0.06) | 0.84 | 1147 | 0.33 (0.09) | 0.0001 | 0.03 (0.01) | 0.05 | -0.02 (0.09) | 0.85 | 0.28 (0.15) | 0.06 | 0.03 (0.01) | 0.02 | -0.31 (0.20) | 0.13 |
| *t=fu* | 1072 | 0.26 (0.05) | <0.0001 | 0.02 (0.01) | 0.08 | 0.03 (0.07) | 0.66 | 1147 | 0.40 (0.09) | <0.0001 | 0.01 (0.01) | 0.33 | 0.11 (0.08) | 0.19 | 0.22 (0.20) | 0.26 | 0.03 (0.02) | 0.07 | -0.34 (0.24) | 0.17 |
| **P2S8** | | | | | | | | | | | | | | | | | | | | |
| *t=base* | 1133 | 0.28 (0.05) | <0.0001 | 0.02 (0.01) | 0.13 | 0.01 (0.07) | 0.87 | 1148 | 0.29 (0.08) | 0.0005 | 0.02 (0.01) | 0.18 | -0.001 (0.11) | 0.99 | 0.45 (0.14) | 0.001 | 0.01 (0.01) | 0.30 | -0.12 (0.21) | 0.56 |
| *t=fu* | 1073 | 0.23 (0.05) | <0.0001 | 0.02 (0.01) | 0.12 | -0.02 (0.06) | 0.74 | 1148 | 0.44 (0.08) | <0.0001 | 0.01 (0.01) | 0.31 | 0.03 (0.10) | 0.77 | 0.46 (0.13) | 0.001 | 0.02 (0.01) | 0.27 | -0.23 (0.21) | 0.27 |
| **P2S9** | | | | | | | | | | | | | | | | | | | | |
| *t=base* | 1133 | 0.18 (0.07) | 0.01 | -0.0001 (0.01) | 0.99 | 0.01 (0.08) | 0.94 | 1148 | 0.56 (0.13) | <0.0001 | 0.0002 (0.01) | 0.98 | 0.12 (0.18) | 0.49 | 0.72 (0.26) | 0.01 | -0.0004 (0.01) | 0.97 | 0.26 (0.40) | 0.51 |
| *t=fu* | 1072 | 0.28 (0.07) | <0.0001 | -0.003 (0.01) | 0.81 | -0.01 (0.08) | 0.90 | 1148 | 0.41 (0.11) | 0.0002 | -0.004 (0.01) | 0.76 | -0.11 (0.14) | 0.40 | 0.35 (0.20) | 0.08 | -0.005 (0.01) | 0.68 | 0.001 (0.26) | 1.00 |
| **Experiment Part 2: Injunctive norms (Average P2S2 to P2S9)** | | | | | | | | | | | | | | | | | | | | |
| *t=base* | 1122 | 0.27 (0.06) | <0.0001 | 0.01 (0.01) | 0.14 | 0.06 (0.06) | 0.29 | 1137 | 0.46 (0.11) | <0.0001 | 0.01 (0.01) | 0.22 | 0.22 (0.12) | 0.07 | 0.65 (0.23) | 0.005 | 0.01 (0.01) | 0.30 | 0.32 (0.28) | 0.25 |
| *t=fu* | 1065 | 0.28 (0.05) | <0.0001 | 0.01 (0.01) | 0.23 | 0.12 (0.06) | 0.05 | 1137 | 0.45 (0.08) | <0.0001 | 0.01 (0.01) | 0.49 | 0.15 (0.09) | 0.09 | 0.30 (0.16) | 0.06 | 0.01 (0.01) | 0.30 | -0.05 (0.23) | 0.83 |
| **P3Q1** | | | | | | | | | | | | | | | | | | | | |
| *t=base* | 1134 | 0.08 (0.06) | 0.20 | 0.02 (0.02) | 0.19 | -0.09 (0.07) | 0.23 | 1149 | 0.17 (0.12) | 0.15 | 0.03 (0.02) | 0.13 | -0.17 (0.11) | 0.13 | 0.14 (0.17) | 0.43 | 0.02 (0.02) | 0.23 | -0.03 (0.16) | 0.85 |
| *t=fu* | 1073 | 0.11 (0.06) | 0.09 | 0.02 (0.02) | 0.28 | -0.01 (0.08) | 0.91 | 1149 | 0.28 (0.10) | 0.01 | 0.02 (0.02) | 0.25 | -0.10 (0.11) | 0.36 | 0.27 (0.16) | 0.10 | 0.02 (0.02) | 0.27 | 0.11 (0.19) | 0.54 |
| **P3Q2** | | | | | | | | | | | | | | | | | | | | |
| *t=base* | 1133 | 0.21 (0.05) | 0.0001 | 0.03 (0.02) | 0.06 | -0.08 (0.06) | 0.20 | 1148 | 0.35 (0.08) | <0.0001 | 0.04 (0.02) | 0.03 | -0.06 (0.08) | 0.45 | 0.38 (0.11) | 0.0004 | 0.04 (0.02) | 0.04 | 0.01 (0.11) | 0.91 |
| *t=fu* | 1073 | 0.19 (0.06) | 0.001 | 0.03 (0.02) | 0.14 | -0.05 (0.06) | 0.43 | 1148 | 0.31 (0.08) | 0.0001 | 0.03 (0.02) | 0.11 | -0.09 (0.09) | 0.33 | 0.32 (0.11) | 0.005 | 0.03 (0.02) | 0.13 | -0.01 (0.13) | 0.96 |
| **Experiment Part 3: Descriptive norms (Average P3Q1 to P3Q2)** | | | | | | | | | | | | | | | | | | | | |
| *t=base* | 1133 | 0.16 (0.06) | 0.01 | 0.03 (0.02) | 0.05 | -0.07 (0.06) | 0.27 | 1148 | 0.28 (0.09) | 0.003 | 0.04 (0.02) | 0.03 | -0.08 (0.09) | 0.41 | 0.26 (0.12) | 0.03 | 0.03 (0.02) | 0.06 | 0.02 (0.12) | 0.90 |
| *t=fu* | 1073 | 0.14 (0.06) | 0.01 | 0.03 (0.02) | 0.13 | -0.03 (0.07) | 0.68 | 1148 | 0.27 (0.09) | 0.002 | 0.03 (0.02) | 0.11 | -0.10 (0.10) | 0.31 | 0.24 (0.13) | 0.06 | 0.03 (0.02) | 0.14 | 0.05 (0.14) | 0.74 |
| **Donation to ASSIST/Dead Cool** | | | | | | | | | | | | | | | | | | | | |
| *t=base* | 1132 | 0.13 (0.06) | 0.02 | -0.03 (0.10) | 0.73 | 0.09 (0.07) | 0.18 | 1147 | 0.11 (0.10) | 0.27 | -0.01 (0.09) | 0.90 | 0.20 (0.13) | 0.13 | 0.11 (0.19) | 0.57 | -0.05 (0.11) | 0.63 | 0.05 (0.26) | 0.85 |
| *t=fu* | 1071 | 0.36 (0.06) | <0.0001 | -0.04 (0.10) | 0.71 | 0.07 (0.08) | 0.32 | 1147 | 0.47 (0.09) | <0.0001 | -0.03 (0.09) | 0.73 | -0.09 (0.15) | 0.56 | 0.68 (0.14) | <0.0001 | -0.01 (0.10) | 0.91 | -0.09 (0.37) | 0.80 |
| **IN1** | | | | | | | | | | | | | | | | | | | | |
| *t=base* | **1138** | **0.05 (0.07)** | **0.51** | **-0.03 (0.02)** | **0.21** | **0.29 (0.08)** | **0.001** | 1148 | 0.19 (0.14) | 0.17 | -0.03 (0.02) | 0.17 | 0.04 (0.14) | 0.79 | 0.49 (0.24) | 0.04 | -0.04 (0.03) | 0.10 | 0.45 (0.24) | 0.06 |
| *t=fu* | **1135** | **0.005 (0.06)** | **0.94** | **-0.04 (0.02)** | **0.12** | **0.33 (0.08)** | **<0.0001^i^** | **1148** | **-0.19 (0.12)** | **0.11** | **-0.03 (0.02)** | **0.14** | **0.34 (0.13)** | **0.01** | 0.06 (0.24) | 0.79 | -0.04 (0.02) | 0.11 | 0.53 (0.29) | 0.07 |
| **IN2** | | | | | | | | | | | | | | | | | | | | |
| *t=base* | 1140 | -0.01 (0.06) | 0.91 | -0.01 (0.02) | 0.37 | 0.07 (0.07) | 0.37 | 1150 | 0.22 (0.18) | 0.23 | -0.02 (0.02) | 0.31 | -0.03 (0.17) | 0.85 | 0.36 (0.46) | 0.43 | -0.03 (0.02) | 0.26 | 0.34 (0.84) | 0.68 |
| *t=fu* | 1137 | 0.11 (0.07) | 0.12 | -0.01 (0.02) | 0.35 | 0.08 (0.07) | 0.27 | 1150 | 0.13 (0.11) | 0.22 | -0.01 (0.02) | 0.41 | -0.14 (0.10) | 0.14 | -0.05 (0.29) | 0.86 | -0.01 (0.02) | 0.43 | -0.90 (0.49) | 0.06 |
| **IN3** | | | | | | | | | | | | | | | | | | | | |
| *t=base* | 1137 | 0.18 (0.09) | 0.04 | 0.01 (0.02) | 0.80 | -0.10 (0.10) | 0.31 | 1146 | 0.24 (0.12) | 0.05 | 0.01 (0.03) | 0.59 | -0.24 (0.19) | 0.22 | 0.28 (0.17) | 0.11 | -0.002 (0.03) | 0.95 | 0.01 (0.27) | 0.98 |
| *t=fu* | 1133 | 0.10 (0.06) | 0.11 | -0.01 (0.02) | 0.68 | 0.03 (0.07) | 0.69 | 1146 | 0.03 (0.09) | 0.72 | -0.01 (0.02) | 0.71 | 0.02 (0.11) | 0.83 | 0.02 (0.22) | 0.92 | -0.02 (0.03) | 0.49 | 0.23 (0.34) | 0.49 |
| **IN4** | | | | | | | | | | | | | | | | | | | | |
| *t=base* | 1135 | 0.03 (0.06) | 0.58 | -0.04 (0.03) | 0.20 | 0.01 (0.06) | 0.89 | **1146** | **-0.05 (0.13)** | **0.68** | **-0.05 (0.03)** | **0.13** | **-0.31 (0.12)** | **0.01** | 0.91 (0.35) | 0.01 | 0.02 (0.03) | 0.51 | 0.19 (0.26) | 0.47 |
| *t=fu* | 1133 | 0.06 (0.05) | 0.21 | -0.03 (0.03) | 0.22 | 0.07 (0.06) | 0.25 | 1146 | 0.17 (0.11) | 0.12 | -0.02 (0.03) | 0.39 | 0.03 (0.11) | 0.81 | 0.39 (0.20) | 0.05 | -0.01 (0.03) | 0.67 | 0.10 (0.25) | 0.70 |
| **IN5** | | | | | | | | | | | | | | | | | | | | |
| *t=base* | **1137** | **0.06 (0.07)** | **0.36** | **0.01 (0.03)** | **0.62** | **-0.15 (0.06)** | **0.01** | 1147 | -0.04 (0.11) | 0.71 | 0.01 (0.03) | 0.77 | -0.05 (0.12) | 0.66 | 0.09 (0.22) | 0.68 | 0.02 (0.03) | 0.51 | -0.29 (0.20) | 0.16 |
| *t=fu* | 1134 | 0.03 (0.06) | 0.59 | 0.005 (0.03) | 0.86 | -0.14 (0.07) | 0.03 | 1147 | 0.04 (0.11) | 0.73 | 0.003 (0.03) | 0.91 | -0.22 (0.11) | 0.06 | -0.01 (0.24) | 0.95 | 0.0001 (0.03) | 1.00 | -0.45 (0.25) | 0.08 |
| **IN6** | | | | | | | | | | | | | | | | | | | | |
| *t=base* | 1138 | 0.21 (0.05) | 0.0001 | -0.04 (0.03) | 0.12 | 0.06 (0.06) | 0.33 | 1148 | 0.23 (0.10) | 0.02 | -0.04 (0.03) | 0.20 | 0.01 (0.11) | 0.96 | 0.51 (0.16) | 0.001 | -0.06 (0.03) | 0.04 | 0.23 (0.15) | 0.14 |
| *t=fu* | 1135 | 0.25 (0.06) | <0.0001 | -0.04 (0.03) | 0.20 | 0.08 (0.07) | 0.23 | 1148 | 0.29 (0.09) | 0.001 | -0.03 (0.03) | 0.31 | 0.08 (0.09) | 0.39 | 0.43 (0.15) | 0.005 | -0.03 (0.03) | 0.37 | 0.06 (0.18) | 0.76 |
| **IN7** | | | | | | | | | | | | | | | | | | | | |
| *t=base* | 1139 | 0.13 (0.06) | 0.03 | -0.04 (0.03) | 0.19 | 0.04 (0.06) | 0.55 | 1149 | 0.24 (0.11) | 0.03 | -0.04 (0.03) | 0.15 | 0.21 (0.13) | 0.10 | 0.57 (0.16) | 0.001 | -0.04 (0.03) | 0.18 | 0.27 (0.19) | 0.16 |
| *t=fu* | 1136 | 0.18 (0.06) | 0.004 | -0.03 (0.03) | 0.24 | 0.09 (0.08) | 0.28 | 1149 | 0.20 (0.11) | 0.05 | -0.03 (0.03) | 0.26 | 0.16 (0.11) | 0.12 | 0.34 (0.16) | 0.04 | -0.03 (0.03) | 0.31 | 0.20 (0.16) | 0.23 |
| **Self-report injunctive norms (Average IN1 to IN7)** | | | | | | | | | | | | | | | | | | | | |
| *t=base* | 1132 | 0.15 (0.08) | 0.06 | -0.02 (0.02) | 0.13 | 0.07 (0.06) | 0.24 | 1142 | 0.13 (0.11) | 0.25 | -0.02 (0.02) | 0.33 | -0.14 (0.13) | 0.25 | 0.41 (0.17) | 0.01 | -0.02 (0.02) | 0.18 | 0.12 (0.16) | 0.46 |
| *t=fu* | **1129** | **0.17 (0.06)** | **0.003** | **-0.03 (0.02)** | **0.09** | **0.17 (0.06)** | **0.01** | 1142 | 0.21 (0.09) | 0.02 | -0.02 (0.02) | 0.22 | 0.13 (0.09) | 0.15 | 0.27 (0.15) | 0.07 | -0.02 (0.02) | 0.27 | 0.06 (0.16) | 0.72 |
| **DN1.1** | | | | | | | | | | | | | | | | | | | | |
| *t=base* | **1141** | **0.07 (0.11)** | **0.49** | **0.04 (0.02)** | **0.10** | **-0.34 (0.09)** | **0.0002** | **1151** | **0.07 (0.13)** | **0.62** | **0.04 (0.02)** | **0.13** | **-0.33 (0.11)** | **0.004** | 0.35 (0.40) | 0.38 | 0.005 (0.03) | 0.86 | -0.73 (0.31) | 0.02 |
| *t=fu* | 1138 | 0.20 (0.07) | 0.01 | 0.03 (0.02) | 0.16 | -0.11 (0.07) | 0.10 | **1151** | **-0.11 (0.13)** | **0.40** | **0.04 (0.02)** | **0.09** | **-0.41 (0.13)** | **0.002** | 0.36 (0.27) | 0.19 | 0.01 (0.02) | 0.57 | -0.33 (0.31) | 0.29 |
| **DN1.2** | | | | | | | | | | | | | | | | | | | | |
| *t=base* | 1142 | 0.11 (0.05) | 0.02 | -0.01 (0.03) | 0.79 | 0.05 (0.05) | 0.28 | 1152 | 0.03 (0.06) | 0.56 | -0.02 (0.03) | 0.55 | -0.04 (0.06) | 0.54 | 0.15 (0.07) | 0.04 | -0.03 (0.03) | 0.28 | -0.06 (0.08) | 0.45 |
| *t=fu* | 1139 | -0.05 (0.05) | 0.28 | 0.01 (0.03) | 0.69 | 0.07 (0.05) | 0.16 | 1152 | 0.001 (0.07) | 0.99 | -0.01 (0.03) | 0.86 | -0.0003 (0.08) | 1.00 | 0.10 (0.08) | 0.17 | -0.03 (0.03) | 0.36 | -0.08 (0.09) | 0.40 |
| **DN1.3** | | | | | | | | | | | | | | | | | | | | |
| *t=base* | 1141 | 0.09 (0.04) | 0.04 | 0.02 (0.03) | 0.43 | 0.04 (0.05) | 0.42 | 1151 | 0.05 (0.07) | 0.49 | 0.01 (0.03) | 0.64 | -0.02 (0.08) | 0.84 | 0.15 (0.12) | 0.21 | 0.01 (0.03) | 0.74 | 0.03 (0.11) | 0.82 |
| *t=fu* | 1138 | 0.02 (0.05) | 0.59 | 0.02 (0.03) | 0.51 | 0.02 (0.05) | 0.63 | 1151 | 0.03 (0.07) | 0.63 | 0.02 (0.03) | 0.55 | 0.02 (0.08) | 0.82 | 0.08 (0.15) | 0.57 | 0.01 (0.03) | 0.69 | 0.01 (0.16) | 0.95 |
| **DN1.4** | | | | | | | | | | | | | | | | | | | | |
| *t=base* | 1141 | 0.02 (0.04) | 0.62 | 0.01 (0.02) | 0.68 | -0.03 (0.05) | 0.51 | 1151 | 0.07 (0.14) | 0.60 | 0.01 (0.02) | 0.70 | -0.08 (0.13) | 0.54 | 0.09 (0.14) | 0.51 | 0.01 (0.03) | 0.68 | -0.05 (0.13) | 0.67 |
| *t=fu* | 1138 | -0.08 (0.04) | 0.05 | 0.01 (0.02) | 0.69 | -0.03 (0.06) | 0.64 | 1151 | 0.04 (0.12) | 0.76 | 0.01 (0.02) | 0.81 | -0.16 (0.12) | 0.19 | -0.15 (0.16) | 0.35 | 0.01 (0.02) | 0.80 | -0.15 (0.18) | 0.41 |
| **DN1.5** | | | | | | | | | | | | | | | | | | | | |
| *t=base* | 1141 | 0.07 (0.05) | 0.14 | 0.002 (0.02) | 0.93 | -0.02 (0.06) | 0.68 | **1151** | **0.21 (0.11)** | **0.06** | **-0.01 (0.02)** | **0.56** | **-0.31 (0.11)** | **0.005** | 0.31 (0.20) | 0.12 | 0.003 (0.02) | 0.87 | -0.11 (0.17) | 0.52 |
| *t=fu* | 1138 | -0.03 (0.04) | 0.51 | 0.002 (0.02) | 0.91 | -0.09 (0.06) | 0.11 | **1151** | **0.03 (0.10)** | **0.80** | **-0.01 (0.02)** | **0.62** | **-0.37 (0.11)** | **0.001** | 0.16 (0.14) | 0.26 | -0.001 (0.02) | 0.93 | -0.15 (0.15) | 0.33 |
| **Self-report descriptive norms 1 (Average DN1.1 to DN1.5)** | | | | | | | | | | | | | | | | | | | | |
| *t=base* | 1139 | 0.08 (0.05) | 0.11 | 0.002 (0.01) | 0.87 | -0.04 (0.05) | 0.41 | 1149 | 0.02 (0.07) | 0.74 | -0.004 (0.01) | 0.75 | -0.12 (0.07) | 0.06 | 0.16 (0.09) | 0.06 | -0.01 (0.01) | 0.50 | -0.13 (0.08) | 0.10 |
| *t=fu* | 1136 | -0.03 (0.05) | 0.59 | 0.01 (0.01) | 0.48 | -0.02 (0.04) | 0.64 | 1149 | -0.03 (0.07) | 0.69 | 0.0004 (0.01) | 0.98 | -0.13 (0.07) | 0.07 | 0.12 (0.09) | 0.22 | -0.005 (0.01) | 0.73 | -0.11 (0.09) | 0.24 |
| **DN2.1** | | | | | | | | | | | | | | | | | | | | |
| *t=base* | 1142 | 0.13 (0.07) | 0.09 | -0.01 (0.02) | 0.63 | -0.17 (0.08) | 0.05 | 1152 | 0.05 (0.11) | 0.68 | 0.001 (0.02) | 0.95 | -0.17 (0.12) | 0.14 | 0.09 (0.21) | 0.66 | -0.004 (0.02) | 0.87 | -0.21 (0.21) | 0.32 |
| *t=fu* | 1139 | 0.13 (0.07) | 0.05 | -0.001 (0.02) | 0.96 | -0.11 (0.07) | 0.13 | 1152 | 0.07 (0.13) | 0.59 | -0.002 (0.02) | 0.94 | -0.19 (0.12) | 0.12 | -0.27 (0.23) | 0.25 | 0.01 (0.02) | 0.79 | -0.42 (0.21) | 0.04 |
| **DN2.2** | | | | | | | | | | | | | | | | | | | | |
| *t=base* | 1141 | 0.18 (0.06) | 0.002 | 0.02 (0.03) | 0.44 | -0.04 (0.07) | 0.61 | 1151 | 0.28 (0.09) | 0.003 | 0.02 (0.03) | 0.56 | -0.01 (0.10) | 0.88 | 0.44 (0.15) | 0.003 | 0.002 (0.03) | 0.95 | -0.08 (0.14) | 0.60 |
| *t=fu* | 1138 | 0.08 (0.06) | 0.14 | 0.02 (0.03) | 0.43 | -0.05 (0.07) | 0.45 | 1151 | 0.15 (0.08) | 0.08 | 0.01 (0.03) | 0.67 | -0.10 (0.10) | 0.34 | 0.36 (0.13) | 0.01 | -0.0002 (0.03) | 1.00 | -0.06 (0.13) | 0.62 |
| **DN2.3** | | | | | | | | | | | | | | | | | | | | |
| *t=base* | 1142 | 0.26 (0.08) | 0.002 | 0.01 (0.02) | 0.64 | -0.02 (0.08) | 0.82 | 1152 | 0.59 (0.11) | <0.0001 | -0.02 (0.02) | 0.41 | -0.10 (0.10) | 0.32 | 0.76 (0.17) | <0.0001 | -0.02 (0.02) | 0.27 | 0.01 (0.17) | 0.96 |
| *t=fu* | 1139 | 0.17 (0.06) | 0.01 | 0.02 (0.02) | 0.39 | -0.09 (0.06) | 0.16 | 1152 | 0.39 (0.09) | <0.0001 | 0.01 (0.02) | 0.79 | -0.10 (0.09) | 0.25 | 0.55 (0.15) | 0.0001 | -0.005 (0.02) | 0.82 | -0.09 (0.15) | 0.56 |
| **Self-report descriptive norms 2 (Average DN2.1 to DN2.3)** | | | | | | | | | | | | | | | | | | | | |
| *t=base* | 1141 | 0.18 (0.06) | 0.003 | -0.001 (0.02) | 0.98 | -0.09 (0.07) | 0.21 | 1151 | 0.30 (0.10) | 0.004 | -0.01 (0.02) | 0.76 | -0.05 (0.10) | 0.64 | 0.46 (0.16) | 0.003 | -0.01 (0.02) | 0.47 | -0.004 (0.15) | 0.98 |
| *t=fu* | 1138 | 0.04 (0.06) | 0.48 | 0.01 (0.02) | 0.46 | -0.08 (0.06) | 0.16 | 1151 | 0.17 (0.08) | 0.03 | -0.001 (0.02) | 0.96 | -0.14 (0.09) | 0.10 | 0.32 (0.14) | 0.02 | -0.01 (0.02) | 0.66 | -0.08 (0.13) | 0.54 |
| **Self-report smoking behavior** | | | | | | | | | | | | | | | | | | | | |
| *t=base* | 1151 | 0.13 (0.07) | 0.08 | -0.002 (0.02) | 0.92 | -0.07 (0.09) | 0.43 | 1162 | 0.21 (0.10) | 0.03 | -0.01 (0.02) | 0.69 | -0.07 (0.12) | 0.55 | 0.50 (0.16) | 0.001 | -0.01 (0.02) | 0.72 | -0.35 (0.18) | 0.05 |
| *t=fu* | 1148 | 0.20 (0.06) | 0.002 | -0.005 (0.02) | 0.82 | -0.14 (0.07) | 0.07 | 1162 | 0.21 (0.08) | 0.01 | -0.005 (0.02) | 0.81 | -0.04 (0.10) | 0.70 | 0.36 (0.10) | 0.001 | -0.003 (0.02) | 0.88 | -0.17 (0.13) | 0.17 |
| **Intentions** | | | | | | | | | | | | | | | | | | | | |
| *t=base* | 1145 | 0.18 (0.08) | 0.02 | 0.02 (0.03) | 0.64 | 0.03 (0.09) | 0.70 | 1155 | 0.48 (0.13) | 0.0003 | 0.01 (0.03) | 0.80 | 0.06 (0.17) | 0.73 | 0.94 (0.24) | 0.0001 | -0.002 (0.04) | 0.95 | 0.42 (0.29) | 0.15 |
| *t=fu* | 1141 | 0.20 (0.07) | 0.004 | 0.02 (0.04) | 0.61 | -0.07 (0.09) | 0.40 | 1155 | 0.14 (0.11) | 0.17 | 0.002 (0.04) | 0.95 | 0.13 (0.13) | 0.29 | 0.37 (0.15) | 0.01 | 0.001 (0.04) | 0.99 | 0.15 (0.17) | 0.36 |
| **Knowledge** | | | | | | | | | | | | | | | | | | | | |
| *t=base* | 1147 | 0.21 (0.05) | 0.0001 | 0.04 (0.05) | 0.45 | 0.02 (0.07) | 0.80 | 1157 | 0.46 (0.07) | <0.0001 | 0.04 (0.05) | 0.45 | 0.09 (0.09) | 0.35 | 0.57 (0.10) | <0.0001 | 0.01 (0.06) | 0.84 | 0.29 (0.16) | 0.06 |
| *t=fu* | 1144 | 0.29 (0.05) | <0.0001 | 0.04 (0.05) | 0.40 | -0.01 (0.06) | 0.89 | 1157 | 0.44 (0.07) | <0.0001 | 0.02 (0.05) | 0.64 | 0.04 (0.08) | 0.58 | 0.47 (0.10) | <0.0001 | -0.005 (0.06) | 0.93 | 0.20 (0.14) | 0.16 |
| **Attitudes** | | | | | | | | | | | | | | | | | | | | |
| *t=base* | 1127 | 0.18 (0.06) | 0.002 | -0.02 (0.02) | 0.28 | 0.002 (0.06) | 0.97 | 1136 | 0.01 (0.11) | 0.96 | -0.01 (0.02) | 0.50 | 0.05 (0.12) | 0.71 | 0.62 (0.25) | 0.01 | -0.04 (0.03) | 0.09 | -0.13 (0.20) | 0.49 |
| *t=fu* | 1122 | 0.19 (0.06) | 0.003 | -0.02 (0.02) | 0.46 | -0.02 (0.08) | 0.81 | 1136 | -0.02 (0.12) | 0.87 | -0.01 (0.02) | 0.63 | -0.13 (0.11) | 0.25 | 0.26 (0.18) | 0.16 | -0.01 (0.02) | 0.59 | -0.21 (0.16) | 0.19 |
| **Self-efficacy (Emotional)** | | | | | | | | | | | | | | | | | | | | |
| *t=base* | 1138 | 0.14 (0.07) | 0.04 | -0.06 (0.03) | 0.03 | 0.03 (0.08) | 0.67 | 1147 | 0.38 (0.12) | 0.002 | -0.07 (0.03) | 0.02 | 0.13 (0.12) | 0.28 | 0.59 (0.17) | 0.001 | -0.07 (0.03) | 0.01 | 0.08 (0.19) | 0.70 |
| *t=fu* | 1132 | 0.27 (0.06) | <0.0001 | -0.04 (0.03) | 0.21 | -0.07 (0.08) | 0.37 | 1147 | 0.24 (0.08) | 0.003 | -0.06 (0.03) | 0.07 | 0.10 (0.10) | 0.31 | 0.44 (0.11) | 0.0001 | -0.05 (0.03) | 0.12 | 0.08 (0.13) | 0.53 |
| **Self-efficacy (Friends)** | | | | | | | | | | | | | | | | | | | | |
| *t=base* | 1142 | 0.08 (0.06) | 0.17 | -0.05 (0.02) | 0.03 | 0.08 (0.07) | 0.25 | 1151 | 0.30 (0.12) | 0.01 | -0.07 (0.03) | 0.01 | 0.21 (0.11) | 0.06 | **0.55 (0.20)** | **0.01** | **-0.09 (0.03)** | **0.002** | **0.44 (0.18)** | **0.01** |
| *t=fu* | 1138 | 0.19 (0.06) | 0.002 | -0.04 (0.03) | 0.10 | -0.02 (0.08) | 0.84 | 1151 | 0.10 (0.09) | 0.25 | -0.06 (0.03) | 0.03 | 0.19 (0.09) | 0.04 | 0.18 (0.15) | 0.23 | -0.07 (0.03) | 0.01 | 0.33 (0.16) | 0.04 |
| **Self-efficacy (Opportunity)** | | | | | | | | | | | | | | | | | | | | |
| *t=base* | 1143 | 0.14 (0.07) | 0.04 | -0.03 (0.02) | 0.25 | -0.003 (0.08) | 0.97 | 1152 | 0.43 (0.14) | 0.002 | -0.03 (0.02) | 0.16 | 0.03 (0.12) | 0.78 | 0.65 (0.23) | 0.01 | -0.04 (0.02) | 0.08 | 0.38 (0.24) | 0.11 |
| *t=fu* | 1139 | 0.15 (0.06) | 0.02 | -0.02 (0.02) | 0.39 | -0.0002 (0.08) | 1.00 | 1152 | 0.24 (0.09) | 0.01 | -0.02 (0.02) | 0.39 | 0.04 (0.11) | 0.69 | 0.31 (0.15) | 0.03 | -0.02 (0.03) | 0.36 | 0.10 (0.17) | 0.58 |
| **Perceived physical risks** | | | | | | | | | | | | | | | | | | | | |
| *t=base* | 1137 | 0.11 (0.05) | 0.04 | -0.90 (0.73) | 0.22 | -0.09 (0.06) | 0.17 | 1145 | 0.18 (0.09) | 0.04 | -1.39 (0.78) | 0.07 | 0.02 (0.11) | 0.83 | 0.23 (0.15) | 0.13 | -1.95 (0.95) | 0.04 | 0.21 (0.19) | 0.28 |
| *t=fu* | 1131 | 0.10 (0.05) | 0.07 | -0.93 (0.71) | 0.19 | 0.04 (0.06) | 0.57 | 1145 | 0.11 (0.10) | 0.28 | -0.95 (0.69) | 0.17 | -0.01 (0.12) | 0.95 | -0.12 (0.22) | 0.59 | -0.77 (0.74) | 0.29 | -0.12 (0.30) | 0.68 |
| **Perceived social risks** | | | | | | | | | | | | | | | | | | | | |
| *t=base* | 1143 | 0.14 (0.05) | 0.01 | -1.47 (0.83) | 0.08 | 0.02 (0.07) | 0.74 | 1153 | 0.26 (0.07) | 0.0003 | -1.32 (0.86) | 0.13 | 0.03 (0.11) | 0.78 | 0.42 (0.09) | <0.0001 | -1.74 (0.92) | 0.06 | 0.31 (0.16) | 0.06 |
| *t=fu* | 1139 | 0.11 (0.06) | 0.05 | -1.35 (0.83) | 0.11 | -0.01 (0.07) | 0.85 | 1153 | 0.21 (0.08) | 0.01 | -1.15 (0.83) | 0.17 | -0.01 (0.12) | 0.96 | 0.38 (0.12) | 0.002 | -1.02 (0.89) | 0.25 | 0.16 (0.18) | 0.36 |
| **Perceived addiction risks** | | | | | | | | | | | | | | | | | | | | |
| *t=base* | 1073 | 0.20 (0.06) | 0.0005 | -1.93 (0.93) | 0.04 | 0.10 (0.07) | 0.16 | 1083 | 0.47 (0.09) | <0.0001 | -1.20 (0.95) | 0.21 | 0.03 (0.10) | 0.76 | 0.69 (0.10) | <0.0001 | -0.53 (1.04) | 0.61 | 0.003 (0.15) | 0.98 |
| *t=fu* | 1066 | 0.13 (0.05) | 0.01 | -1.59 (0.90) | 0.08 | -0.06 (0.06) | 0.31 | 1083 | 0.40 (0.08) | <0.0001 | -0.90 (0.92) | 0.33 | -0.05 (0.10) | 0.60 | 0.57 (0.09) | <0.0001 | -0.43 (0.98) | 0.66 | -0.04 (0.14) | 0.77 |
| **Perceived benefits** | | | | | | | | | | | | | | | | | | | | |
| *t=base* | 1079 | -0.03 (0.06) | 0.60 | -0.49 (0.74) | 0.51 | -0.06 (0.07) | 0.39 | 1089 | 0.17 (0.11) | 0.12 | -0.35 (0.76) | 0.65 | -0.21 (0.13) | 0.10 | 0.45 (0.22) | 0.04 | -1.59 (1.30) | 0.22 | 0.42 (0.57) | 0.47 |
| *t=fu* | 1075 | 0.05 (0.06) | 0.40 | -0.68 (0.72) | 0.35 | 0.03 (0.08) | 0.69 | 1089 | -0.06 (0.12) | 0.61 | -0.26 (0.76) | 0.73 | -0.23 (0.16) | 0.15 | -0.16 (0.27) | 0.54 | 1.01 (1.48) | 0.49 | -1.37 (1.03) | 0.18 |
| **Perceived behavioral control (easy to quit)** | | | | | | | | | | | | | | | | | | | | |
| *t=base* | 1138 | 0.30 (0.06) | <0.0001 | 0.08 (0.05) | 0.14 | -0.08 (0.07) | 0.25 | 1147 | 0.50 (0.08) | <0.0001 | 0.04 (0.06) | 0.51 | -0.11 (0.10) | 0.28 | 0.68 (0.10) | <0.0001 | 0.04 (0.06) | 0.52 | 0.05 (0.13) | 0.68 |
| *t=fu* | 1133 | 0.20 (0.06) | 0.0004 | 0.11 (0.05) | 0.04 | 0.02 (0.07) | 0.79 | 1147 | 0.51 (0.08) | <0.0001 | 0.05 (0.06) | 0.39 | 0.06 (0.09) | 0.54 | 0.63 (0.10) | <0.0001 | 0.01 (0.06) | 0.85 | -0.01 (0.13) | 0.91 |
| **Perceived behavioral control (to avoid smoking)** | | | | | | | | | | | | | | | | | | | | |
| *t=base* | 1143 | 0.08 (0.07) | 0.22 | -0.02 (0.04) | 0.55 | -0.04 (0.08) | 0.59 | 1152 | 0.37 (0.13) | 0.004 | -0.02 (0.04) | 0.58 | -0.12 (0.16) | 0.44 | 0.84 (0.20) | <0.0001 | -0.03 (0.05) | 0.48 | -0.08 (0.26) | 0.75 |
| *t=fu* | 1138 | 0.09 (0.07) | 0.21 | -0.03 (0.04) | 0.49 | -0.02 (0.08) | 0.85 | 1152 | 0.15 (0.13) | 0.23 | -0.03 (0.04) | 0.43 | 0.04 (0.16) | 0.80 | 0.55 (0.17) | 0.001 | -0.01 (0.05) | 0.84 | -0.04 (0.21) | 0.85 |
| **Objectively measured smoking behavior** | | | | | | | | | | | | | | | | | | | | |
| *t=base* | 1104 | 0.38 (0.05) | <0.0001 | -0.02 (0.05) | 0.65 | -0.01 (0.05) | 0.90 | 1112 | 0.44 (0.05) | <0.0001 | -0.08 (0.06) | 0.14 | -0.09 (0.05) | 0.10 | 0.66 (0.06) | <0.0001 | -0.16 (0.06) | 0.01 | -0.09 (0.08) | 0.24 |
| *t=fu* | 1083 | 0.48 (0.09) | <0.0001 | 0.07 (0.06) | 0.22 | 0.13 (0.08) | 0.08 | 1112 | 0.83 (0.05) | <0.0001 | -0.01 (0.05) | 0.91 | 0.02 (0.06) | 0.68 | 0.85 (0.07) | <0.0001 | 0.002 (0.06) | 0.97 | 0.05 (0.08) | 0.55 |
| **Smoking susceptibility^e^** | | | | | | | | | | | | | | | | | | | | |
|  | n | **Percent_-i,t_^f^** | | **M^g^** | | **M*Percent_-i,t_^h^** | | n | **Percent_-i,t_^f^** | | **M^g^** | | **M*Percent_-i,t_^h^** | | **Percent_-i,t_^f^** | | **M^g^** | | **M*Percent_-i,t_^h^** | |
|  |  | OR (SE) | p-value | OR (SE) | p-value | OR (SE) | p-value |  | OR (SE) | p-value | OR (SE) | p-value | OR (SE) | p-value | OR (SE) | p-value | OR (SE) | p-value | OR (SE) | p-value |
| *t=base* | 1145 | 1.12 (0.03) | 0.0002 | 1.21 (0.10) | 0.03 | 1.07 (0.04) | 0.08 | 1155 | 1.12 (0.07) | 0.05 | 1.19 (0.10) | 0.03 | 1.08 (0.09) | 0.37 | 1.21 (0.12) | 0.07 | 1.20 (0.10) | 0.03 | 1.07 (0.17) | 0.65 |
| *t=fu* | 1141 | 1.13 (0.03) | <0.0001 | 1.14 (0.10) | 0.13 | 1.02 (0.03) | 0.56 | 1155 | 1.15 (0.06) | 0.003 | 1.17 (0.10) | 0.07 | 1.09 (0.06) | 0.14 | 1.29 (0.09) | 0.0003 | 1.11 (0.11) | 0.31 | 1.01 (0.09) | 0.90 |

IV: Independent variable; DV: Dependent variable; Ave: average of peer group (*i*); M: moderator (School socio-economic status: 1=lowest SES to 4=highest SES. NI: 1=NIMDM2017≤222.5; 2=222.5<NIMDM2017≤445; 3=445<NIMDM2017≤667.5; 4=NIMDM2017>667.5. Bogotá: 1=Lower; 2=Middle-low; 3=Middle-high; 4=Higher); Percent: percentage of peer group (*-i*) classified as susceptible to commencing smoking; OR: odds ratio; SE: standard error.

^a^In each model the outcome variable is the focal participant’s (*i*) response to the relevant item at follow-up. The predictor variable is the average of the relevant group’s (*-i*) responses to the equivalent item at baseline (*t*=base) or follow-up (*t*=fu), where *–i*=(1) focal participant's nominated friends; (2) focal participant’s school class; (3) focal participant’s school year group. The moderator, and interaction of the moderator with the predictor variable, were also included as independent variables in all models. All models include robust (Huber White) standard errors specified using Stata’s ‘vce(robust)’ option. The following baseline variables are included as covariates in all models: gender (0=boy; 1=girl/prefer not to say), age (1=12 years or less; 2=13 years; 3=14 years or more), intervention (1=ASSIST; 2=Dead Cool), ethnicity (0=no ethnic minority; 1=ethnic minority), and baseline values of the outcome variable. The predictor variable, moderator variable, and baseline values of the outcome variable were mean-centered.

^b^Unstandardized regression coefficients representing the average change in the outcome variable for a one-unit increase in the predictor variable among participants who are average on school socio-economic status.

^c^Unstandardized regression coefficients representing the average change in the outcome variable for a one-unit increase in school socio-economic status among participants who are average on the predictor variable.

^d^Unstandardized regression coefficients representing the average change in the association between the outcome variable and the predictor variable for a one-unit increase in school socio-economic status.

^e^Logistic regressions were run for models including focal participants' smoking susceptibility as the outcome variable, with robust (Huber White) standard errors specified using Stata’s ‘vce(robust)’ option. The predictor variable is the percentage of the relevant group (*-i*) classified as susceptible to commencing smoking at baseline (*t*=base) or follow-up (*t*=fu), where *–i*=(1) focal participant's nominated friends; (2) focal participant’s school class; (3) focal participant’s school year group. The moderator, and interaction of the moderator with the predictor variable, were also included as independent variables in all models. The following baseline variables are included as covariates in all models: gender (0=boy; 1=girl/prefer not to say), age (1=12 years or less; 2=13 years; 3=14 years or more), intervention (1=ASSIST; 2=Dead Cool), ethnicity (0=no ethnic minority; 1=ethnic minority), and baseline values of the outcome variable. Results are odds ratios, standard errors, and p-values. The predictor variable, and moderator variable were mean-centered.

^f^Odds ratios representing the multiplicative change in odds of being susceptible to commencing smoking for a 10% increase in the number of nominated friends/pupils in the same school class/pupils in the same school year group classified as being susceptible to commencing smoking (1 out of 10 nominated friends/pupils in the same school class/pupils in the same school year group; predictor variable) among participants who are average on school socio-economic status.

^g^Odds ratios representing the multiplicative change in odds of being susceptible to commencing smoking for a one-unit increase in school socio-economic status among participants who are average on the predictor variable.

^h^Ratio of ratios representing the ratio of: (1) the odds ratio representing the multiplicative change in odds of being susceptible to commencing smoking for a 10% increase in the number of nominated friends/pupils in the same school class/pupils in the same school year group classified as being susceptible to commencing smoking among participants who are one unit above average on school socio-economic status; to (2) the odds ratio representing the multiplicative change in odds of being susceptible to commencing smoking for a 10% increase in the number of nominated friends/pupils in the same school class/pupils in the same school year group classified as being susceptible to commencing smoking among participants who are average on school socio-economic status.

^i^Retained statistical significance at the 5% level after using the Holm-Bonferroni procedure to correct the p-values for multiple testing (p≤0.05; based on 276 tests of interaction effects reported in Table S2.4).

**Table S2.5.** Results of ordinary least squares linear regressions including interaction terms examining differences in peer influence effects according to school socio-economic status for outcomes collected at follow-up in NI schools (Northern Ireland Multiple Deprivation Measure NIMDM2017).

|  | **Dependent variable: Participant responses to the outcome variable at follow-upa** | | | | | | |
| --- | --- | --- | --- | --- | --- | --- | --- |
|  | **(1) -i=Average of nominated friends** | | | | | | |
| **IV** | n | **Ave-i,tb** | | **Mc** | | **M*Ave-i,td** | |
| **DV** |  | *b (SE)* | p-value | *b (SE)* | p-value | *b (SE)* | p-value |
| **P2S2** | | | | | | | |
| *t=base* | 521 | 0.18 (0.13) | 0.16 | -0.001 (0.001) | 0.05 | -0.005 (0.01) | 0.27 |
| *t=fu* | 471 | -0.03 (0.09) | 0.79 | -0.001 (0.001) | 0.07 | -0.008 (0.004) | 0.07 |
| **P2S3** | | | | | | | |
| *t=base* | 522 | 0.25 (0.11) | 0.03 | -0.002 (0.001) | 0.01 | -0.002 (0.004) | 0.63 |
| *t=fu* | 471 | 0.15 (0.10) | 0.13 | -0.002 (0.001) | 0.03 | -0.003 (0.005) | 0.58 |
| **P2S4** | | | | | | | |
| *t=base* | 520 | 0.23 (0.12) | 0.06 | 0.0002 (0.001) | 0.75 | 0.002 (0.005) | 0.73 |
| *t=fu* | 470 | 0.01 (0.10) | 0.93 | -0.0002 (0.001) | 0.75 | -0.003 (0.005) | 0.51 |
| **P2S5** | | | | | | | |
| *t=base* | 523 | 0.09 (0.09) | 0.33 | 0.0001 (0.001) | 0.94 | 0.003 (0.004) | 0.51 |
| *t=fu* | 472 | -0.01 (0.09) | 0.91 | 0.0002 (0.001) | 0.78 | 0.007 (0.01) | 0.16 |
| **P2S6** | | | | | | | |
| *t=base* | 519 | 0.19 (0.10) | 0.06 | 0.001 (0.001) | 0.27 | 0.003 (0.005) | 0.56 |
| *t=fu* | 469 | 0.06 (0.09) | 0.51 | 0.0004 (0.001) | 0.64 | 0.007 (0.004) | 0.05 |
| **P2S7** | | | | | | | |
| *t=base* | 521 | 0.12 (0.09) | 0.21 | 0.001 (0.001) | 0.36 | 0.002 (0.004) | 0.72 |
| *t=fu* | 471 | 0.11 (0.09) | 0.22 | 0.001 (0.001) | 0.41 | 0.006 (0.005) | 0.23 |
| **P2S8** | | | | | | | |
| *t=base* | 522 | 0.12 (0.10) | 0.25 | 0.0004 (0.001) | 0.67 | -0.001 (0.004) | 0.82 |
| *t=fu* | 472 | 0.06 (0.09) | 0.49 | 0.0004 (0.001) | 0.68 | -0.005 (0.004) | 0.24 |
| **P2S9** | | | | | | | |
| *t=base* | 522 | 0.16 (0.11) | 0.12 | -0.0001 (0.001) | 0.84 | 0.009 (0.01) | 0.10 |
| *t=fu* | 471 | 0.21 (0.10) | 0.04 | -0.0006 (0.001) | 0.40 | -0.004 (0.005) | 0.43 |
| **Experiment Part 2: Injunctive norms (Average P2S2 to P2S9)** | | | | | | | |
| *t=base* | 511 | 0.24 (0.09) | 0.01 | 0.0002 (0.0004) | 0.60 | 0.003 (0.004) | 0.44 |
| *t=fu* | 464 | 0.18 (0.09) | 0.05 | 0.0002 (0.0005) | 0.72 | 0.005 (0.004) | 0.24 |
| **P3Q1** | | | | | | | |
| *t=base* | 523 | 0.17 (0.09) | 0.06 | 0.001 (0.001) | 0.66 | -0.003 (0.004) | 0.43 |
| *t=fu* | 472 | 0.07 (0.09) | 0.42 | 0.0000 (0.001) | 0.97 | -0.003 (0.01) | 0.49 |
| **P3Q2** | | | | | | | |
| *t=base* | 522 | 0.18 (0.09) | 0.05 | 0.001 (0.001) | 0.49 | -0.004 (0.004) | 0.31 |
| *t=fu* | 472 | 0.15 (0.09) | 0.09 | 0.001 (0.001) | 0.61 | -0.003 (0.005) | 0.48 |
| **Experiment Part 3: Descriptive norms (Average P3Q1 to P3Q2)** | | | | | | | |
| *t=base* | 522 | 0.20 (0.09) | 0.04 | 0.001 (0.001) | 0.44 | -0.004 (0.004) | 0.36 |
| *t=fu* | 472 | 0.11 (0.09) | 0.21 | 0.001 (0.001) | 0.64 | -0.003 (0.005) | 0.59 |
| **Donation to ASSIST/Dead Cool** | | | | | | | |
| *t=base* | 521 | 0.16 (0.08) | 0.06 | -0.003 (0.006) | 0.60 | 0.004 (0.004) | 0.27 |
| *t=fu* | 470 | 0.34 (0.09) | 0.0002 | -0.002 (0.006) | 0.69 | 0.007 (0.004) | 0.12 |
| **IN1** | | | | | | | |
| *t=base* | **533** | **0.02 (0.11)** | **0.84** | **-0.001 (0.001)** | **0.58** | **0.01 (0.005)** | **0.003** |
| *t=fu* | 531 | -0.19 (0.10) | 0.06 | -0.0001 (0.001) | 0.97 | 0.01 (0.005) | 0.04 |
| **IN2** | | | | | | | |
| *t=base* | 535 | -0.03 (0.07) | 0.67 | -0.001 (0.001) | 0.50 | 0.002 (0.003) | 0.56 |
| *t=fu* | 533 | 0.02 (0.08) | 0.84 | -0.001 (0.001) | 0.50 | 0.002 (0.004) | 0.60 |
| **IN3** | | | | | | | |
| *t=base* | 532 | 0.20 (0.18) | 0.29 | 0.0002 (0.001) | 0.82 | -0.002 (0.005) | 0.60 |
| *t=fu* | 529 | 0.10 (0.14) | 0.46 | 0.0001 (0.001) | 0.90 | 0.002 (0.004) | 0.63 |
| **IN4** | | | | | | | |
| *t=base* | 530 | 0.08 (0.08) | 0.33 | -0.003 (0.002) | 0.11 | 0.002 (0.003) | 0.40 |
| *t=fu* | 529 | 0.08 (0.08) | 0.28 | -0.002 (0.002) | 0.17 | 0.004 (0.003) | 0.26 |
| **IN5** | | | | | | | |
| *t=base* | **532** | **0.13 (0.11)** | **0.27** | **0.0001 (0.002)** | **0.96** | **-0.008 (0.003)** | **0.01** |
| *t=fu* | 530 | 0.14 (0.09) | 0.13 | 0.0003 (0.002) | 0.84 | -0.006 (0.004) | 0.15 |
| **IN6** | | | | | | | |
| *t=base* | 533 | 0.12 (0.09) | 0.17 | -0.001 (0.002) | 0.66 | 0.002 (0.004) | 0.50 |
| *t=fu* | 531 | 0.07 (0.10) | 0.46 | -0.0001 (0.002) | 0.95 | 0.004 (0.004) | 0.34 |
| **IN7** | | | | | | | |
| *t=base* | 534 | 0.19 (0.09) | 0.04 | -0.001 (0.001) | 0.57 | 0.003 (0.003) | 0.41 |
| *t=fu* | 532 | 0.11 (0.11) | 0.31 | 0.0000 (0.002) | 0.99 | 0.001 (0.01) | 0.89 |
| **Self-report injunctive norms (Average IN1 to IN7)** | | | | | | | |
| *t=base* | 527 | 0.15 (0.16) | 0.34 | -0.001 (0.001) | 0.28 | 0.002 (0.003) | 0.50 |
| *t=fu* | 525 | 0.07 (0.10) | 0.50 | -0.001 (0.001) | 0.38 | 0.008 (0.004) | 0.04 |
| **DN1.1** | | | | | | | |
| *t=base* | **536** | **-0.01 (0.15)** | **0.96** | **0.005 (0.001)** | **0.0004** | **-0.013 (0.004)** | **0.001** |
| *t=fu* | 534 | 0.12 (0.10) | 0.24 | 0.004 (0.001) | 0.002 | -0.006 (0.003) | 0.05 |
| **DN1.2** | | | | | | | |
| *t=base* | 537 | 0.06 (0.06) | 0.32 | -0.0007 (0.001) | 0.66 | 0.002 (0.003) | 0.45 |
| *t=fu* | 535 | -0.17 (0.07) | 0.02 | 0.0005 (0.002) | 0.75 | 0.002 (0.003) | 0.44 |
| **DN1.3** | | | | | | | |
| *t=base* | 536 | 0.13 (0.07) | 0.08 | 0.001 (0.002) | 0.46 | 0.0002 (0.003) | 0.95 |
| *t=fu* | 534 | 0.02 (0.07) | 0.82 | 0.001 (0.002) | 0.48 | 0.0003 (0.003) | 0.91 |
| **DN1.4** | | | | | | | |
| *t=base* | 536 | 0.09 (0.10) | 0.34 | 0.001 (0.001) | 0.29 | -0.002 (0.002) | 0.36 |
| *t=fu* | 534 | -0.09 (0.06) | 0.13 | 0.002 (0.001) | 0.19 | 0.0001 (0.004) | 0.97 |
| **DN1.5** | | | | | | | |
| *t=base* | 536 | 0.09 (0.10) | 0.41 | 0.0003 (0.001) | 0.73 | 0.001 (0.003) | 0.86 |
| *t=fu* | 534 | -0.03 (0.07) | 0.63 | 0.0005 (0.001) | 0.66 | -0.002 (0.003) | 0.38 |
| **Self-report descriptive norms 1 (Average DN1.1 to DN1.5)** | | | | | | | |
| *t=base* | 534 | 0.04 (0.08) | 0.62 | 0.001 (0.001) | 0.30 | -0.003 (0.003) | 0.31 |
| *t=fu* | 532 | -0.11 (0.09) | 0.21 | 0.001 (0.001) | 0.10 | 0.0004 (0.002) | 0.88 |
| **DN2.1** | | | | | | | |
| *t=base* | 537 | 0.18 (0.10) | 0.06 | -0.001 (0.001) | 0.39 | -0.001 (0.01) | 0.78 |
| *t=fu* | 535 | 0.01 (0.09) | 0.88 | 0.0006 (0.001) | 0.69 | -0.007 (0.004) | 0.10 |
| **DN2.2** | | | | | | | |
| *t=base* | 536 | 0.07 (0.10) | 0.49 | -0.0001 (0.002) | 0.95 | -0.005 (0.005) | 0.27 |
| *t=fu* | 534 | 0.01 (0.10) | 0.95 | 0.0007 (0.002) | 0.68 | -0.004 (0.004) | 0.37 |
| **DN2.3** | | | | | | | |
| *t=base* | 537 | 0.07 (0.12) | 0.54 | 0.003 (0.001) | 0.03 | -0.004 (0.01) | 0.47 |
| *t=fu* | 535 | -0.003 (0.07) | 0.96 | 0.004 (0.001) | 0.002 | -0.005 (0.003) | 0.12 |
| **Self-report descriptive norms 2 (Average DN2.1 to DN2.3)** | | | | | | | |
| *t=base* | 536 | -0.04 (0.11) | 0.71 | 0.001 (0.001) | 0.29 | -0.007 (0.004) | 0.08 |
| *t=fu* | 534 | -0.13 (0.10) | 0.19 | 0.002 (0.001) | 0.09 | -0.004 (0.003) | 0.19 |
| **Self-report smoking behavior** | | | | | | | |
| *t=base* | 546 | 0.06 (0.12) | 0.60 | 0.001 (0.001) | 0.25 | -0.006 (0.01) | 0.24 |
| *t=fu* | **544** | **0.18 (0.12)** | **0.13** | **0.001 (0.001)** | **0.49** | **-0.01 (0.004)** | **0.01** |
| **Intentions** | | | | | | | |
| *t=base* | 540 | 0.22 (0.10) | 0.03 | 0.002 (0.001) | 0.12 | -0.002 (0.004) | 0.62 |
| *t=fu* | 537 | 0.24 (0.13) | 0.06 | 0.003 (0.002) | 0.14 | -0.007 (0.01) | 0.27 |
| **Knowledge** | | | | | | | |
| *t=base* | 542 | 0.12 (0.08) | 0.17 | 0.005 (0.003) | 0.08 | 0.0002 (0.004) | 0.96 |
| *t=fu* | 540 | 0.31 (0.09) | 0.0005 | 0.004 (0.003) | 0.13 | 0.0005 (0.004) | 0.91 |
| **Attitudes** | | | | | | | |
| *t=base* | 522 | 0.14 (0.10) | 0.15 | 0.0003 (0.001) | 0.80 | -0.002 (0.004) | 0.54 |
| *t=fu* | 518 | 0.18 (0.11) | 0.10 | 0.001 (0.001) | 0.62 | -0.005 (0.005) | 0.28 |
| **Self-efficacy (Emotional)** | | | | | | | |
| *t=base* | 533 | 0.11 (0.09) | 0.22 | -0.0001 (0.001) | 0.94 | -0.002 (0.005) | 0.72 |
| *t=fu* | **528** | **0.16 (0.10)** | **0.12** | **0.002 (0.001)** | **0.15** | **-0.02 (0.01)** | **0.004** |
| **Self-efficacy (Friends)** | | | | | | | |
| *t=base* | 537 | -0.01 (0.08) | 0.91 | 0.0001 (0.001) | 0.95 | -0.004 (0.004) | 0.33 |
| *t=fu* | **534** | **-0.01 (0.10)** | **0.91** | **0.002 (0.001)** | **0.09** | **-0.02 (0.01)** | **0.002** |
| **Self-efficacy (Opportunity)** | | | | | | | |
| *t=base* | 538 | 0.14 (0.10) | 0.16 | -0.001 (0.001) | 0.55 | -0.004 (0.01) | 0.46 |
| *t=fu* | **535** | **-0.10 (0.10)** | **0.27** | **0.001 (0.001)** | **0.40** | **-0.02 (0.01)** | **0.01** |
| **Perceived physical risks** | | | | | | | |
| *t=base* | **532** | **0.19 (0.08)** | **0.02** | **0.005 (0.04)** | **0.90** | **-0.007 (0.003)** | **0.01** |
| *t=fu* | 527 | 0.03 (0.08) | 0.67 | -0.005 (0.04) | 0.89 | -0.0004 (0.003) | 0.92 |
| **Perceived social risks** | | | | | | | |
| *t=base* | 538 | -0.01 (0.10) | 0.94 | -0.007 (0.04) | 0.87 | -0.0001 (0.003) | 0.98 |
| *t=fu* | 535 | 0.04 (0.10) | 0.66 | -0.01 (0.04) | 0.76 | -0.006 (0.004) | 0.13 |
| **Perceived addiction risks** | | | | | | | |
| *t=base* | 511 | 0.11 (0.10) | 0.23 | 0.03 (0.04) | 0.44 | 0.005 (0.01) | 0.36 |
| *t=fu* | 509 | -0.08 (0.08) | 0.33 | 0.01 (0.05) | 0.83 | -0.007 (0.003) | 0.02 |
| **Perceived benefits** | | | | | | | |
| *t=base* | 474 | -0.01 (0.08) | 0.94 | -0.05 (0.04) | 0.22 | -0.001 (0.003) | 0.81 |
| *t=fu* | 471 | 0.002 (0.08) | 0.98 | -0.05 (0.04) | 0.18 | -0.002 (0.004) | 0.70 |
| **Perceived behavioral control (easy to quit)** | | | | | | | |
| *t=base* | 533 | 0.21 (0.11) | 0.06 | 0.003 (0.003) | 0.30 | -0.005 (0.004) | 0.19 |
| *t=fu* | 529 | 0.04 (0.11) | 0.70 | 0.002 (0.003) | 0.46 | 0.003 (0.005) | 0.58 |
| **Perceived behavioral control (to avoid smoking)** | | | | | | | |
| *t=base* | 538 | 0.27 (0.11) | 0.02 | 0.001 (0.002) | 0.54 | 0.003 (0.005) | 0.52 |
| *t=fu* | 534 | 0.10 (0.10) | 0.32 | 0.0004 (0.002) | 0.86 | 0.003 (0.005) | 0.47 |
| **Objectively measured smoking behavior** | | | | | | | |
| *t=base* | 497 | 0.05 (0.07) | 0.44 | -0.01 (0.002) | 0.0005 | -0.005 (0.003) | 0.05 |
| *t=fu* | 493 | 0.70 (0.09) | <0.0001 | -0.002 (0.002) | 0.16 | -0.007 (0.004) | 0.12 |
| **Smoking susceptibilitye** | | | | | | | |
|  | n | **Percent-i,tf** | | **Mg** | | **M*Percent-i,th** | |
|  |  | OR (SE) | p-value | OR (SE) | p-value | OR (SE) | p-value |
| *t=base* | 540 | 1.04 (0.05) | 0.47 | 1.01 (0.005) | 0.32 | 1.0003 (0.002) | 0.87 |
| *t=fu* | 537 | 1.11 (0.05) | 0.03 | 1.002 (0.005) | 0.65 | 0.998 (0.002) | 0.33 |

IV: Independent variable; DV: Dependent variable; Ave: average of peer group (*i*); M: moderator (School socio-economic status: 5.7=lowest SES [NIMDM2017=57] to 80.2=highest SES [NIMDM2017=802]); Percent: percentage of peer group (*-i*) classified as susceptible to commencing smoking; OR: odds ratio; SE: standard error.

^a^In each model the outcome variable is the focal participant’s (*i*) response to the relevant item at follow-up. The predictor variable is the average of the relevant group’s (*-i*) responses to the equivalent item at baseline (*t*=base) or follow-up (*t*=fu), where *–i*=(1) focal participant's nominated friends. The moderator, and interaction of the moderator with the predictor variable, were also included as independent variables in all models. All models include robust (Huber White) standard errors specified using Stata’s ‘vce(robust)’ option. The following baseline variables are included as covariates in all models: gender (0=boy; 1=girl/prefer not to say), age (1=12 years or less; 2=13 years; 3=14 years or more), intervention (1=ASSIST; 2=Dead Cool), ethnicity (0=no ethnic minority; 1=ethnic minority), and baseline values of the outcome variable. The predictor variable, moderator variable, and baseline values of the outcome variable were mean-centered.

^b^Unstandardized regression coefficients representing the average change in the outcome variable for a one-unit increase in the predictor variable among participants who are average on school socio-economic status.

^c^Unstandardized regression coefficients representing the average change in the outcome variable for a one-unit increase in school socio-economic status among participants who are average on the predictor variable.

^d^Unstandardized regression coefficients representing the average change in the association between the outcome variable and the predictor variable for a one-unit increase in school socio-economic status.

^e^Logistic regressions were run for models including focal participants' smoking susceptibility as the outcome variable, with robust (Huber White) standard errors specified using Stata’s ‘vce(robust)’ option. The predictor variable is the percentage of the relevant group (*-i*) classified as susceptible to commencing smoking at baseline (*t*=base) or follow-up (*t*=fu), where *–i*=(1) focal participant's nominated friends. The moderator, and interaction of the moderator with the predictor variable, were also included as independent variables in all models. The following baseline variables are included as covariates in all models: gender (0=boy; 1=girl/prefer not to say), age (1=12 years or less; 2=13 years; 3=14 years or more), intervention (1=ASSIST; 2=Dead Cool), ethnicity (0=no ethnic minority; 1=ethnic minority), and baseline values of the outcome variable. Results are odds ratios, standard errors, and p-values. The predictor variable, and moderator variable were mean-centered.

^f^Odds ratios representing the multiplicative change in odds of being susceptible to commencing smoking for a 10% increase in the number of nominated friends classified as being susceptible to commencing smoking (1 out of 10 nominated friends; predictor variable) among participants who are average on school socio-economic status.

^g^Odds ratios representing the multiplicative change in odds of being susceptible to commencing smoking for a one-unit increase in school socio-economic status among participants who are average on the predictor variable.

^h^Ratio of ratios representing the ratio of: (1) the odds ratio representing the multiplicative change in odds of being susceptible to commencing smoking for a 10% increase in the number of nominated friends classified as being susceptible to commencing smoking among participants who are one unit above average on school socio-economic status; to (2) the odds ratio representing the multiplicative change in odds of being susceptible to commencing smoking for a 10% increase in the number of nominated friends classified as being susceptible to commencing smoking among participants who are average on school socio-economic status.

**Table S2.6.** Results of ordinary least squares linear regressions including interaction terms examining differences in peer influence effects according to school socio-economic status for outcomes collected at follow-up in Bogotá schools (socio-economic level index for educational institutions).

|  | **Dependent variable: Participant responses to the outcome variable at follow-upa** | | | | | | |
| --- | --- | --- | --- | --- | --- | --- | --- |
|  | **(1) -i=Average of nominated friends** | | | | | | |
| **IV** | n | **Ave-i,tb** | | **Mc** | | **M*Ave-i,td** | |
| **DV** |  | *b (SE)* | p-value | *b (SE)* | p-value | *b (SE)* | p-value |
| **P2S2** | | | | | | | |
| *t=base* | 611 | 0.32 (0.10) | 0.002 | -0.01 (0.02) | 0.64 | 0.10 (0.21) | 0.64 |
| *t=fu* | 601 | 0.22 (0.09) | 0.01 | -0.005 (0.02) | 0.82 | 0.34 (0.17) | 0.04 |
| **P2S3** | | | | | | | |
| *t=base* | 611 | 0.05 (0.09) | 0.57 | 0.03 (0.03) | 0.38 | 0.03 (0.18) | 0.88 |
| *t=fu* | 601 | 0.07 (0.09) | 0.44 | 0.02 (0.03) | 0.45 | 0.31 (0.18) | 0.09 |
| **P2S4** | | | | | | | |
| *t=base* | 611 | 0.09 (0.10) | 0.37 | -0.01 (0.03) | 0.80 | 0.09 (0.19) | 0.65 |
| *t=fu* | 601 | 0.18 (0.09) | 0.04 | -0.01 (0.03) | 0.86 | 0.35 (0.18) | 0.05 |
| **P2S5** | | | | | | | |
| *t=base* | 611 | 0.18 (0.08) | 0.04 | 0.12 (0.03) | 0.0002 | 0.11 (0.17) | 0.53 |
| *t=fu* | 601 | 0.19 (0.08) | 0.01 | 0.12 (0.03) | 0.0004 | 0.05 (0.14) | 0.73 |
| **P2S6** | | | | | | | |
| *t=base* | 611 | 0.11 (0.09) | 0.21 | 0.09 (0.04) | 0.02 | 0.18 (0.18) | 0.34 |
| *t=fu* | 601 | 0.02 (0.08) | 0.79 | 0.10 (0.04) | 0.01 | 0.07 (0.16) | 0.66 |
| **P2S7** | | | | | | | |
| *t=base* | 611 | 0.15 (0.08) | 0.06 | 0.07 (0.03) | 0.03 | 0.16 (0.16) | 0.32 |
| *t=fu* | 601 | 0.27 (0.07) | 0.0003 | 0.06 (0.03) | 0.08 | -0.13 (0.15) | 0.40 |
| **P2S8** | | | | | | | |
| *t=base* | 611 | 0.36 (0.07) | <0.0001 | 0.08 (0.03) | 0.02 | -0.11 (0.14) | 0.47 |
| *t=fu* | 601 | 0.27 (0.07) | 0.0002 | 0.07 (0.03) | 0.03 | -0.01 (0.14) | 0.96 |
| **P2S9** | | | | | | | |
| *t=base* | 611 | 0.14 (0.10) | 0.13 | 0.05 (0.03) | 0.10 | -0.13 (0.19) | 0.48 |
| *t=fu* | 601 | 0.27 (0.09) | 0.003 | 0.04 (0.03) | 0.20 | 0.02 (0.17) | 0.91 |
| **Experiment Part 2: Injunctive norms (Average P2S2 to P2S9)** | | | | | | | |
| *t=base* | 611 | 0.21 (0.08) | 0.01 | 0.04 (0.02) | 0.04 | 0.28 (0.18) | 0.11 |
| *t=fu* | 601 | 0.30 (0.07) | <0.0001 | 0.03 (0.02) | 0.08 | 0.24 (0.14) | 0.09 |
| **P3Q1** | | | | | | | |
| *t=base* | 611 | 0.07 (0.09) | 0.47 | 0.12 (0.04) | 0.005 | 0.001 (0.17) | 0.99 |
| *t=fu* | 601 | 0.10 (0.09) | 0.27 | 0.11 (0.04) | 0.01 | 0.18 (0.17) | 0.29 |
| **P3Q2** | | | | | | | |
| *t=base* | 611 | 0.23 (0.08) | 0.003 | 0.09 (0.05) | 0.04 | -0.05 (0.15) | 0.76 |
| *t=fu* | 601 | 0.20 (0.08) | 0.01 | 0.11 (0.05) | 0.02 | 0.08 (0.15) | 0.62 |
| **Experiment Part 3: Descriptive norms (Average P3Q1 to P3Q2)** | | | | | | | |
| *t=base* | 611 | 0.17 (0.08) | 0.04 | 0.11 (0.04) | 0.01 | 0.01 (0.16) | 0.95 |
| *t=fu* | 601 | 0.14 (0.08) | 0.07 | 0.11 (0.04) | 0.01 | 0.11 (0.15) | 0.49 |
| **Donation to ASSIST/Dead Cool** | | | | | | | |
| *t=base* | 611 | 0.01 (0.08) | 0.88 | -0.59 (0.19) | 0.002 | 0.08 (0.15) | 0.59 |
| *t=fu* | 601 | 0.30 (0.07) | 0.0001 | -0.41 (0.20) | 0.04 | -0.08 (0.16) | 0.61 |
| **IN1** | | | | | | | |
| *t=base* | 605 | 0.08 (0.11) | 0.44 | -0.11 (0.07) | 0.11 | 0.36 (0.20) | 0.07 |
| *t=fu* | **604** | **0.11 (0.07)** | **0.13** | **-0.09 (0.07)** | **0.19** | **0.44 (0.17)** | **0.01** |
| **IN2** | | | | | | | |
| *t=base* | 605 | 0.06 (0.11) | 0.57 | 0.003 (0.04) | 0.95 | 0.25 (0.21) | 0.24 |
| *t=fu* | 604 | 0.16 (0.10) | 0.12 | 0.002 (0.04) | 0.96 | -0.001 (0.21) | 0.99 |
| **IN3** | | | | | | | |
| *t=base* | 605 | 0.14 (0.08) | 0.08 | 0.01 (0.06) | 0.87 | -0.26 (0.16) | 0.10 |
| *t=fu* | 604 | 0.08 (0.07) | 0.23 | 0.002 (0.06) | 0.97 | -0.05 (0.14) | 0.75 |
| **IN4** | | | | | | | |
| *t=base* | 605 | -0.03 (0.07) | 0.68 | -0.07 (0.07) | 0.31 | 0.09 (0.14) | 0.55 |
| *t=fu* | 604 | -0.04 (0.07) | 0.60 | -0.07 (0.07) | 0.30 | 0.29 (0.14) | 0.04 |
| **IN5** | | | | | | | |
| *t=base* | 605 | 0.01 (0.07) | 0.90 | -0.07 (0.07) | 0.32 | -0.05 (0.15) | 0.76 |
| *t=fu* | 604 | -0.11 (0.07) | 0.13 | -0.09 (0.07) | 0.17 | 0.09 (0.15) | 0.53 |
| **IN6** | | | | | | | |
| *t=base* | 605 | 0.22 (0.07) | 0.003 | 0.03 (0.07) | 0.64 | -0.05 (0.15) | 0.72 |
| *t=fu* | 604 | 0.30 (0.07) | <0.0001 | 0.01 (0.07) | 0.86 | 0.01 (0.15) | 0.96 |
| **IN7** | | | | | | | |
| *t=base* | 605 | 0.05 (0.07) | 0.46 | -0.07 (0.07) | 0.32 | 0.004 (0.15) | 0.98 |
| *t=fu* | 604 | 0.18 (0.07) | 0.01 | -0.04 (0.07) | 0.53 | 0.16 (0.15) | 0.29 |
| **Self-report injunctive norms (Average IN1 to IN7)** | | | | | | | |
| *t=base* | 605 | 0.16 (0.08) | 0.04 | -0.03 (0.04) | 0.47 | 0.13 (0.15) | 0.36 |
| *t=fu* | 604 | 0.23 (0.06) | 0.0004 | -0.03 (0.04) | 0.43 | 0.22 (0.14) | 0.11 |
| **DN1.1** | | | | | | | |
| *t=base* | 605 | 0.17 (0.13) | 0.20 | -0.09 (0.04) | 0.04 | -0.07 (0.28) | 0.79 |
| *t=fu* | 604 | 0.14 (0.11) | 0.21 | -0.06 (0.04) | 0.14 | 0.42 (0.21) | 0.04 |
| **DN1.2** | | | | | | | |
| *t=base* | 605 | 0.18 (0.09) | 0.04 | -0.04 (0.05) | 0.34 | 0.22 (0.18) | 0.22 |
| *t=fu* | 604 | 0.04 (0.06) | 0.52 | -0.03 (0.05) | 0.47 | 0.11 (0.13) | 0.42 |
| **DN1.3** | | | | | | | |
| *t=base* | 605 | 0.05 (0.06) | 0.38 | 0.01 (0.06) | 0.86 | 0.20 (0.12) | 0.09 |
| *t=fu* | 604 | 0.01 (0.05) | 0.87 | 0.01 (0.07) | 0.88 | -0.01 (0.10) | 0.89 |
| **DN1.4** | | | | | | | |
| *t=base* | 605 | 0.02 (0.06) | 0.78 | -0.004 (0.06) | 0.95 | 0.14 (0.12) | 0.25 |
| *t=fu* | 604 | -0.10 (0.05) | 0.06 | -0.004 (0.06) | 0.95 | -0.04 (0.10) | 0.64 |
| **DN1.5** | | | | | | | |
| *t=base* | 605 | 0.03 (0.05) | 0.52 | 0.04 (0.04) | 0.27 | -0.01 (0.10) | 0.93 |
| *t=fu* | 604 | -0.06 (0.06) | 0.28 | 0.05 (0.04) | 0.18 | -0.08 (0.12) | 0.54 |
| **Self-report descriptive norms 1 (Average DN1.1 to DN1.5)** | | | | | | | |
| *t=base* | **605** | **0.11 (0.06)** | **0.07** | **-0.02 (0.03)** | **0.53** | **0.32 (0.12)** | **0.01** |
| *t=fu* | 604 | 0.007 (0.07) | 0.92 | -0.01 (0.03) | 0.63 | 0.02 (0.13) | 0.90 |
| **DN2.1** | | | | | | | |
| *t=base* | 605 | 0.10 (0.11) | 0.37 | 0.02 (0.06) | 0.72 | -0.10 (0.21) | 0.64 |
| *t=fu* | 604 | 0.20 (0.09) | 0.02 | 0.002 (0.05) | 0.96 | -0.02 (0.17) | 0.91 |
| **DN2.2** | | | | | | | |
| *t=base* | 605 | 0.19 (0.08) | 0.02 | 0.11 (0.07) | 0.11 | -0.07 (0.16) | 0.65 |
| *t=fu* | 604 | 0.01 (0.07) | 0.85 | 0.10 (0.07) | 0.16 | -0.21 (0.14) | 0.13 |
| **DN2.3** | | | | | | | |
| *t=base* | 605 | 0.20 (0.11) | 0.08 | -0.12 (0.04) | 0.01 | 0.31 (0.22) | 0.15 |
| *t=fu* | 604 | 0.07 (0.11) | 0.56 | -0.13 (0.04) | 0.003 | 0.25 (0.22) | 0.26 |
| **Self-report descriptive norms 2 (Average DN2.1 to DN2.3)** | | | | | | | |
| *t=base* | 605 | 0.21 (0.08) | 0.01 | 0.004 (0.04) | 0.92 | -0.06 (0.17) | 0.71 |
| *t=fu* | 604 | 0.07 (0.07) | 0.31 | -0.01 (0.04) | 0.78 | -0.13 (0.14) | 0.35 |
| **Self-report smoking behavior** | | | | | | | |
| *t=base* | 605 | 0.08 (0.08) | 0.32 | -0.01 (0.04) | 0.80 | 0.18 (0.16) | 0.24 |
| *t=fu* | 604 | 0.18 (0.07) | 0.01 | -0.01 (0.04) | 0.72 | 0.28 (0.14) | 0.04 |
| **Intentions** | | | | | | | |
| *t=base* | 605 | 0.13 (0.11) | 0.25 | -0.12 (0.10) | 0.26 | 0.12 (0.22) | 0.59 |
| *t=fu* | 604 | 0.10 (0.09) | 0.25 | -0.11 (0.11) | 0.29 | -0.08 (0.18) | 0.65 |
| **Knowledge** | | | | | | | |
| *t=base* | 605 | 0.02 (0.08) | 0.77 | 0.01 (0.11) | 0.94 | -0.06 (0.16) | 0.72 |
| *t=fu* | 604 | 0.05 (0.08) | 0.53 | 0.01 (0.11) | 0.91 | 0.10 (0.14) | 0.49 |
| **Attitudes** | | | | | | | |
| *t=base* | 605 | 0.16 (0.08) | 0.04 | -0.07 (0.05) | 0.15 | 0.14 (0.15) | 0.35 |
| *t=fu* | 604 | 0.13 (0.08) | 0.11 | -0.07 (0.05) | 0.18 | 0.28 (0.17) | 0.09 |
| **Self-efficacy (Emotional)** | | | | | | | |
| *t=base* | 605 | 0.08 (0.10) | 0.41 | -0.11 (0.07) | 0.11 | 0.16 (0.20) | 0.43 |
| *t=fu* | 604 | 0.19 (0.08) | 0.02 | -0.10 (0.07) | 0.15 | 0.01 (0.16) | 0.96 |
| **Self-efficacy (Friends)** | | | | | | | |
| *t=base* | 605 | 0.11 (0.09) | 0.22 | -0.14 (0.07) | 0.05 | 0.13 (0.18) | 0.46 |
| *t=fu* | 604 | 0.19 (0.08) | 0.01 | -0.12 (0.07) | 0.08 | 0.01 (0.15) | 0.93 |
| **Self-efficacy (Opportunity)** | | | | | | | |
| *t=base* | 605 | 0.13 (0.10) | 0.19 | -0.04 (0.06) | 0.48 | -0.02 (0.19) | 0.91 |
| *t=fu* | 604 | 0.20 (0.08) | 0.01 | -0.04 (0.06) | 0.50 | -0.17 (0.16) | 0.28 |
| **Perceived physical risks** | | | | | | | |
| *t=base* | 605 | 0.05 (0.07) | 0.48 | -1.11 (1.98) | 0.57 | 0.002 (0.13) | 0.99 |
| *t=fu* | 604 | 0.12 (0.07) | 0.08 | -0.74 (1.94) | 0.70 | 0.12 (0.14) | 0.37 |
| **Perceived social risks** | | | | | | | |
| *t=base* | 605 | 0.10 (0.07) | 0.12 | -1.68 (2.14) | 0.43 | 0.02 (0.13) | 0.90 |
| *t=fu* | 604 | 0.03 (0.08) | 0.72 | -0.87 (2.15) | 0.69 | 0.21 (0.16) | 0.20 |
| **Perceived addiction risks** | | | | | | | |
| *t=base* | 562 | -0.01 (0.08) | 0.89 | -0.51 (2.00) | 0.80 | 0.29 (0.17) | 0.09 |
| *t=fu* | 557 | -0.05 (0.08) | 0.54 | -0.04 (2.01) | 0.99 | 0.19 (0.16) | 0.25 |
| **Perceived benefits** | | | | | | | |
| *t=base* | 605 | -0.08 (0.08) | 0.34 | 1.08 (1.80) | 0.55 | -0.002 (0.17) | 0.99 |
| *t=fu* | 604 | 0.05 (0.07) | 0.52 | 1.16 (1.82) | 0.52 | 0.14 (0.15) | 0.35 |
| **Perceived behavioral control (easy to quit)** | | | | | | | |
| *t=base* | 605 | 0.002 (0.09) | 0.98 | 0.04 (0.11) | 0.70 | -0.005 (0.18) | 0.98 |
| *t=fu* | 604 | -0.15 (0.09) | 0.09 | 0.06 (0.11) | 0.61 | 0.01 (0.18) | 0.94 |
| **Perceived behavioral control (to avoid smoking)** | | | | | | | |
| *t=base* | 605 | -0.11 (0.08) | 0.21 | 0.13 (0.11) | 0.25 | 0.13 (0.17) | 0.46 |
| *t=fu* | 604 | -0.07 (0.09) | 0.47 | 0.12 (0.11) | 0.27 | -0.03 (0.19) | 0.88 |
| **Objectively measured smoking behavior** | | | | | | | |
| *t=base* | **607** | **-0.12 (0.12)** | **0.30** | **0.19 (0.15)** | **0.21** | **-0.64 (0.20)** | **0.001** |
| *t=fu* | **590** | **0.36 (0.06)** | **<0.0001** | **0.15 (0.13)** | **0.26** | **0.43 (0.13)** | **0.001** |
| **Smoking susceptibilitye** | | | | | | | |
|  | n | **Percent-i,tf** | | **Mg** | | **M*Percent-i,th** | |
|  |  | OR (SE) | p-value | OR (SE) | p-value | OR (SE) | p-value |
| *t=base* | 605 | 1.18 (0.05) | 0.0001 | 1.25 (0.25) | 0.26 | 1.11 (0.09) | 0.23 |
| *t=fu* | 604 | 1.09 (0.04) | 0.01 | 1.15 (0.23) | 0.47 | 1.14 (0.08) | 0.07 |

IV: Independent variable; DV: Dependent variable; Ave: average of peer group (*i*); M: moderator (School socio-economic status: 1=Lower; 2=Middle-low; 3=Middle-high; 4=Higher); Percent: percentage of peer group (*-i*) classified as susceptible to commencing smoking; OR: odds ratio; SE: standard error.

^a^In each model the outcome variable is the focal participant’s (*i*) response to the relevant item at follow-up. The predictor variable is the average of the relevant group’s (*-i*) responses to the equivalent item at baseline (*t*=base) or follow-up (*t*=fu), where *–i*=(1) focal participant's nominated friends. The moderator, and interaction of the moderator with the predictor variable, were also included as independent variables in all models. All models include robust (Huber White) standard errors specified using Stata’s ‘vce(robust)’ option. The following baseline variables are included as covariates in all models: gender (0=boy; 1=girl/prefer not to say), age (1=12 years or less; 2=13 years; 3=14 years or more), intervention (1=ASSIST; 2=Dead Cool), ethnicity (0=no ethnic minority; 1=ethnic minority), and baseline values of the outcome variable. The predictor variable, moderator variable, and baseline values of the outcome variable were mean-centered.

^b^Unstandardized regression coefficients representing the average change in the outcome variable for a one-unit increase in the predictor variable among participants who are average on school socio-economic status.

^c^Unstandardized regression coefficients representing the average change in the outcome variable for a one-unit increase in school socio-economic status among participants who are average on the predictor variable.

^d^Unstandardized regression coefficients representing the average change in the association between the outcome variable and the predictor variable for a one-unit increase in school socio-economic status.

^e^Logistic regressions were run for models including focal participants' smoking susceptibility as the outcome variable, with robust (Huber White) standard errors specified using Stata’s ‘vce(robust)’ option. The predictor variable is the percentage of the relevant group (*-i*) classified as susceptible to commencing smoking at baseline (*t*=base) or follow-up (*t*=fu), where *–i*=(1) focal participant's nominated friends. The moderator, and interaction of the moderator with the predictor variable, were also included as independent variables in all models. The following baseline variables are included as covariates in all models: gender (0=boy; 1=girl/prefer not to say), age (1=12 years or less; 2=13 years; 3=14 years or more), intervention (1=ASSIST; 2=Dead Cool), ethnicity (0=no ethnic minority; 1=ethnic minority), and baseline values of the outcome variable. Results are odds ratios, standard errors, and p-values. The predictor variable, and moderator variable were mean-centered.

^f^Odds ratios representing the multiplicative change in odds of being susceptible to commencing smoking for a 10% increase in the number of nominated friends classified as being susceptible to commencing smoking (1 out of 10 nominated friends; predictor variable) among participants who are average on school socio-economic status.

^g^Odds ratios representing the multiplicative change in odds of being susceptible to commencing smoking for a one-unit increase in school socio-economic status among participants who are average on the predictor variable.

^h^Ratio of ratios representing the ratio of: (1) the odds ratio representing the multiplicative change in odds of being susceptible to commencing smoking for a 10% increase in the number of nominated friends classified as being susceptible to commencing smoking among participants who are one unit above average on school socio-economic status; to (2) the odds ratio representing the multiplicative change in odds of being susceptible to commencing smoking for a 10% increase in the number of nominated friends classified as being susceptible to commencing smoking among participants who are average on school socio-economic status.

**Table S2.7.** Results of ordinary least squares linear regressions including interaction terms examining differences in peer influence effects according to norm sensitivities (rule-following) for outcomes collected at follow-up.

|  | **Dependent variable: Participant responses to the outcome variable at follow-up^a^** | | | | | | | | | | | | | | | | | | | |
| --- | --- | --- | --- | --- | --- | --- | --- | --- | --- | --- | --- | --- | --- | --- | --- | --- | --- | --- | --- | --- |
|  | **(1) -i=Average of nominated friends** | | | | | | | n | **(2) -i=Average of school class** | | | | | | **(3) -i=Average of school year group** | | | | | |
| **IV** | n | **Ave_-i,t_^b^** | | **M^c^** | | **M*Ave_-i,t_^d^** | |  | **Ave_-i,t_^b^** | | **M^c^** | | **M*Ave_-i,t_^d^** | | **Ave_-i,t_^b^** | | **M^c^** | | **M*Ave_-i,t_^d^** | |
| **DV** |  | *b* (SE) | p-value | *b* (SE) | p-value | *b* (SE) | p-value |  | *b* (SE) | p-value | *b* (SE) | p-value | *b* (SE) | p-value | *b* (SE) | p-value | *b* (SE) | p-value | *b* (SE) | p-value |
| **P2S2** | | | | | | | | | | | | | | | | | | | | |
| *t*=base | 1073 | 0.34 (0.08) | <0.001 | 0.005 (0.005) | 0.34 | 0.03 (0.05) | 0.50 | 1087 | 0.42 (0.12) | <0.001 | 0.006 (0.005) | 0.24 | -0.08 (0.06) | 0.19 | 0.82 (0.18) | <0.001 | 0.007 (0.005) | 0.18 | -0.15 (0.09) | 0.10 |
| *t*=fu | 1018 | 0.15 (0.06) | 0.02 | 0.005 (0.005) | 0.37 | -0.02 (0.04) | 0.51 | 1087 | 0.31 (0.10) | 0.003 | 0.005 (0.005) | 0.33 | -0.04 (0.06) | 0.52 | 0.59 (0.16) | <0.001 | 0.006 (0.005) | 0.23 | -0.14 (0.09) | 0.14 |
| **P2S3** | | | | | | | | | | | | | | | | | | | | |
| *t*=base | 1072 | 0.11 (0.07) | 0.11 | -0.002 (0.006) | 0.71 | -0.009 (0.04) | 0.81 | 1086 | 0.17 (0.16) | 0.29 | -0.002 (0.006) | 0.68 | 0.01 (0.08) | 0.89 | -0.005 (0.28) | 0.99 | -0.002 (0.006) | 0.68 | -0.10 (0.13) | 0.45 |
| *t*=fu | 1017 | 0.11 (0.07) | 0.10 | -0.004 (0.006) | 0.52 | 0.04 (0.04) | 0.33 | 1086 | 0.21 (0.14) | 0.14 | -0.002 (0.006) | 0.67 | 0.07 (0.08) | 0.40 | 0.17 (0.26) | 0.50 | -0.003 (0.006) | 0.66 | -0.006 (0.13) | 0.96 |
| **P2S4** | | | | | | | | | | | | | | | | | | | | |
| *t*=base | 1070 | 0.13 (0.08) | 0.08 | -0.004 (0.006) | 0.50 | -0.05 (0.04) | 0.26 | 1084 | 0.16 (0.12) | 0.19 | -0.004 (0.006) | 0.45 | -0.07 (0.07) | 0.32 | 0.46 (0.32) | 0.15 | -0.005 (0.006) | 0.41 | 0.01 (0.14) | 0.94 |
| *t*=fu | 1016 | 0.12 (0.07) | 0.07 | -0.008 (0.006) | 0.18 | 0.02 (0.04) | 0.53 | 1084 | 0.27 (0.12) | 0.02 | -0.005 (0.006) | 0.39 | 0.11 (0.07) | 0.09 | 0.42 (0.20) | 0.03 | -0.005 (0.006) | 0.37 | 0.10 (0.09) | 0.29 |
| **P2S5** | | | | | | | | | | | | | | | | | | | | |
| *t*=base | 1073 | 0.19 (0.06) | 0.002 | 0.0007 (0.007) | 0.92 | 0.05 (0.04) | 0.21 | 1087 | 0.32 (0.10) | 0.001 | -0.0004 (0.007) | 0.96 | 0.01 (0.05) | 0.82 | 0.48 (0.15) | 0.002 | 0.0002 (0.007) | 0.97 | 0.06 (0.08) | 0.41 |
| *t*=fu | 1018 | 0.17 (0.06) | 0.003 | -0.001 (0.007) | 0.88 | 0.06 (0.03) | 0.07 | 1087 | 0.45 (0.09) | <0.001 | -0.0006 (0.007) | 0.93 | 0.02 (0.05) | 0.73 | 0.44 (0.14) | 0.002 | 0.0009 (0.007) | 0.90 | 0.11 (0.07) | 0.11 |
| **P2S6** | | | | | | | | | | | | | | | | | | | | |
| *t*=base | 1070 | 0.16 (0.07) | 0.02 | 0.003 (0.007) | 0.63 | 0.03 (0.04) | 0.44 | 1084 | 0.36 (0.11) | 0.001 | 0.002 (0.007) | 0.79 | 0.02 (0.06) | 0.75 | 0.40 (0.19) | 0.03 | 0.002 (0.007) | 0.80 | 0.14 (0.09) | 0.13 |
| *t*=fu | 1015 | 0.15 (0.06) | 0.007 | -0.0004 (0.007) | 0.96 | -0.008 (0.03) | 0.81 | 1084 | 0.45 (0.10) | <0.001 | 0.002 (0.007) | 0.75 | 0.006 (0.05) | 0.91 | 0.50 (0.15) | 0.001 | 0.002 (0.007) | 0.76 | 0.06 (0.08) | 0.42 |
| **P2S7** | | | | | | | | | | | | | | | | | | | | |
| *t*=base | 1072 | 0.16 (0.05) | 0.002 | 0.007 (0.007) | 0.28 | 0.05 (0.03) | 0.05 | 1086 | 0.30 (0.09) | 0.001 | 0.006 (0.006) | 0.34 | 0.07 (0.04) | 0.08 | 0.29 (0.14) | 0.04 | 0.006 (0.006) | 0.33 | 0.09 (0.06) | 0.16 |
| *t*=fu | 1018 | 0.26 (0.05) | <0.001 | 0.006 (0.007) | 0.41 | 0.06 (0.03) | 0.07 | 1086 | 0.41 (0.09) | <0.001 | 0.007 (0.006) | 0.30 | 0.05 (0.04) | 0.27 | 0.33 (0.17) | 0.05 | 0.007 (0.006) | 0.28 | 0.12 (0.07) | 0.08 |
| **P2S8** | | | | | | | | | | | | | | | | | | | | |
| *t*=base | 1073 | 0.28 (0.05) | <0.001 | 0.01 (0.006) | 0.10 | 0.06 (0.03) | 0.02 | **1087** | **0.32 (0.08)** | **<0.001** | **0.01 (0.006)** | **0.11** | **0.11 (0.04)** | **0.01** | 0.44 (0.14) | 0.002 | 0.01 (0.006) | 0.12 | 0.09 (0.07) | 0.18 |
| *t*=fu | 1018 | 0.22 (0.05) | <0.001 | 0.008 (0.006) | 0.20 | 0.06 (0.03) | 0.03 | 1087 | 0.46 (0.08) | <0.001 | 0.01 (0.006) | 0.10 | 0.09 (0.05) | 0.05 | 0.48 (0.13) | <0.001 | 0.01 (0.006) | 0.08 | 0.12 (0.07) | 0.08 |
| **P2S9** | | | | | | | | | | | | | | | | | | | | |
| *t*=base | 1072 | 0.15 (0.07) | 0.02 | 0.0003 (0.006) | 0.95 | 0.03 (0.04) | 0.38 | 1086 | 0.51 (0.13) | <0.001 | 0.001 (0.006) | 0.84 | 0.06 (0.07) | 0.37 | 0.57 (0.22) | 0.008 | 0.001 (0.006) | 0.80 | 0.13 (0.11) | 0.26 |
| *t*=fu | 1017 | 0.23 (0.06) | <0.001 | -0.003 (0.006) | 0.56 | 0.08 (0.04) | 0.03 | 1086 | 0.40 (0.11) | <0.001 | 0.0009 (0.006) | 0.88 | 0.12 (0.06) | 0.04 | 0.22 (0.20) | 0.28 | 0.001 (0.006) | 0.86 | 0.07 (0.11) | 0.49 |
| **Experiment Part 2: Injunctive norms (Average P2S2 to P2S9)** | | | | | | | | | | | | | | | | | | | | |
| *t*=base | 1064 | 0.22 (0.06) | <0.001 | 0.002 (0.004) | 0.64 | 0.05 (0.03) | 0.16 | 1078 | 0.36 (0.10) | <0.001 | 0.001 (0.004) | 0.71 | 0.08 (0.05) | 0.14 | 0.41 (0.15) | 0.006 | 0.001 (0.004) | 0.71 | 0.12 (0.08) | 0.15 |
| *t*=fu | 1011 | 0.24 (0.05) | <0.001 | 0.00009 (0.004) | 0.98 | 0.04 (0.03) | 0.26 | 1078 | 0.45 (0.08) | <0.001 | 0.001 (0.004) | 0.74 | 0.07 (0.05) | 0.13 | 0.30 (0.14) | 0.03 | 0.002 (0.004) | 0.67 | 0.12 (0.07) | 0.11 |
| **P3Q1** | | | | | | | | | | | | | | | | | | | | |
| *t*=base | 1073 | 0.09 (0.06) | 0.18 | 0.007 (0.009) | 0.38 | 0.06 (0.04) | 0.07 | 1087 | 0.24 (0.11) | 0.03 | 0.009 (0.009) | 0.30 | 0.03 (0.05) | 0.60 | 0.15 (0.15) | 0.32 | 0.01 (0.009) | 0.26 | 0.02 (0.08) | 0.84 |
| *t*=fu | 1018 | 0.10 (0.06) | 0.11 | 0.006 (0.009) | 0.47 | 0.03 (0.03) | 0.31 | 1087 | 0.30 (0.10) | 0.003 | 0.01 (0.009) | 0.26 | 0.009 (0.05) | 0.87 | 0.20 (0.17) | 0.24 | 0.01 (0.009) | 0.25 | -0.01 (0.09) | 0.88 |
| **P3Q2** | | | | | | | | | | | | | | | | | | | | |
| *t*=base | 1073 | 0.20 (0.05) | <0.001 | 0.01 (0.009) | 0.14 | 0.04 (0.03) | 0.19 | 1087 | 0.34 (0.08) | <0.001 | 0.02 (0.009) | 0.06 | 0.06 (0.04) | 0.15 | 0.33 (0.10) | 0.002 | 0.02 (0.009) | 0.08 | 0.09 (0.05) | 0.12 |
| *t*=fu | 1018 | 0.19 (0.06) | 0.001 | 0.01 (0.009) | 0.15 | 0.03 (0.03) | 0.45 | 1087 | 0.32 (0.08) | <0.001 | 0.02 (0.009) | 0.05 | 0.06 (0.04) | 0.19 | 0.29 (0.12) | 0.02 | 0.02 (0.009) | 0.07 | 0.06 (0.07) | 0.37 |
| **Experiment Part 3: Descriptive norms (Average P3Q1 to P3Q2)** | | | | | | | | | | | | | | | | | | | | |
| *t*=base | 1073 | 0.14 (0.06) | 0.01 | 0.01 (0.008) | 0.21 | 0.05 (0.03) | 0.10 | 1087 | 0.28 (0.09) | 0.002 | 0.01 (0.008) | 0.12 | 0.05 (0.04) | 0.23 | 0.21 (0.11) | 0.07 | 0.01 (0.008) | 0.12 | 0.06 (0.06) | 0.34 |
| *t*=fu | 1018 | 0.13 (0.06) | 0.02 | 0.01 (0.008) | 0.23 | 0.04 (0.03) | 0.24 | 1087 | 0.29 (0.09) | 0.001 | 0.01 (0.008) | 0.10 | 0.05 (0.05) | 0.30 | 0.18 (0.13) | 0.17 | 0.01 (0.008) | 0.11 | 0.05 (0.08) | 0.55 |
| **Donation to ASSIST/Dead Cool** | | | | | | | | | | | | | | | | | | | | |
| *t*=base | 1071 | 0.13 (0.06) | 0.03 | 0.18 (0.04) | <0.001 | 0.04 (0.03) | 0.20 | 1085 | 0.11 (0.09) | 0.23 | 0.18 (0.04) | <0.001 | -0.05 (0.05) | 0.31 | 0.23 (0.20) | 0.26 | 0.18 (0.04) | <0.001 | 0.01 (0.09) | 0.87 |
| *t*=fu | 1016 | 0.33 (0.06) | <0.001 | 0.16 (0.04) | <0.001 | 0.02 (0.03) | 0.41 | 1085 | 0.45 (0.09) | <0.001 | 0.17 (0.04) | <0.001 | -0.05 (0.04) | 0.24 | 0.62 (0.14) | <0.001 | 0.17 (0.04) | <0.001 | -0.10 (0.07) | 0.14 |
| **IN1** | | | | | | | | | | | | | | | | | | | | |
| *t*=base | 1040 | -0.05 (0.07) | 0.44 | 0.03 (0.01) | 0.04 | 0.04 (0.04) | 0.34 | 1049 | 0.03 (0.14) | 0.85 | 0.03 (0.01) | 0.04 | 0.12 (0.08) | 0.14 | 0.38 (0.22) | 0.09 | 0.03 (0.01) | 0.05 | 0.12 (0.12) | 0.30 |
| *t*=fu | 1037 | 0.07 (0.06) | 0.24 | 0.03 (0.01) | 0.04 | 0.005 (0.04) | 0.89 | 1049 | -0.17 (0.12) | 0.17 | 0.03 (0.01) | 0.03 | -0.002 (0.08) | 0.98 | 0.24 (0.23) | 0.29 | 0.02 (0.01) | 0.05 | 0.09 (0.14) | 0.50 |
| **IN2** | | | | | | | | | | | | | | | | | | | | |
| *t*=base | 1042 | -0.009 (0.08) | 0.90 | 0.01 (0.008) | 0.18 | 0.02 (0.04) | 0.66 | 1051 | 0.18 (0.19) | 0.32 | 0.01 (0.008) | 0.15 | 0.13 (0.09) | 0.17 | 0.50 (0.53) | 0.34 | 0.01 (0.008) | 0.14 | -0.07 (0.21) | 0.73 |
| *t*=fu | 1039 | 0.12 (0.07) | 0.11 | 0.01 (0.008) | 0.19 | -0.06 (0.05) | 0.17 | 1051 | 0.08 (0.11) | 0.46 | 0.01 (0.008) | 0.19 | -0.02 (0.06) | 0.78 | 0.10 (0.28) | 0.72 | 0.01 (0.008) | 0.17 | 0.09 (0.16) | 0.58 |
| **IN3** | | | | | | | | | | | | | | | | | | | | |
| *t*=base | 1040 | 0.11 (0.07) | 0.11 | 0.0005 (0.01) | 0.96 | 0.05 (0.04) | 0.19 | 1049 | 0.19 (0.11) | 0.08 | 0.0007 (0.01) | 0.95 | 0.04 (0.05) | 0.45 | 0.34 (0.19) | 0.07 | 0.001 (0.01) | 0.92 | -0.002 (0.07) | 0.98 |
| *t*=fu | 1037 | 0.08 (0.05) | 0.15 | 0.0002 (0.01) | 0.99 | 0.008 (0.03) | 0.78 | 1049 | 0.006 (0.10) | 0.95 | 0.0002 (0.01) | 0.98 | 0.04 (0.05) | 0.47 | 0.22 (0.19) | 0.27 | 0.0006 (0.01) | 0.95 | 0.10 (0.09) | 0.26 |
| **IN4** | | | | | | | | | | | | | | | | | | | | |
| *t*=base | 1039 | 0.02 (0.06) | 0.72 | 0.004 (0.01) | 0.77 | -0.02 (0.03) | 0.44 | 1049 | -0.03 (0.13) | 0.82 | 0.006 (0.01) | 0.68 | -0.02 (0.08) | 0.78 | 0.61 (0.32) | 0.06 | 0.003 (0.01) | 0.82 | -0.06 (0.14) | 0.68 |
| *t*=fu | 1037 | 0.04 (0.05) | 0.41 | 0.005 (0.01) | 0.70 | -0.03 (0.03) | 0.31 | 1049 | 0.16 (0.12) | 0.18 | 0.004 (0.01) | 0.77 | 0.005 (0.07) | 0.94 | 0.39 (0.23) | 0.09 | 0.003 (0.01) | 0.83 | 0.03 (0.11) | 0.75 |
| **IN5** | | | | | | | | | | | | | | | | | | | | |
| *t*=base | 1040 | -0.02 (0.06) | 0.75 | 0.007 (0.01) | 0.61 | 0.03 (0.03) | 0.31 | 1049 | -0.04 (0.11) | 0.70 | 0.007 (0.01) | 0.60 | -0.01 (0.06) | 0.84 | 0.02 (0.23) | 0.92 | 0.007 (0.01) | 0.61 | 0.02 (0.11) | 0.83 |
| *t*=fu | 1037 | 0.04 (0.06) | 0.48 | 0.007 (0.01) | 0.60 | -0.02 (0.03) | 0.51 | 1049 | 0.02 (0.12) | 0.86 | 0.006 (0.01) | 0.64 | 0.05 (0.06) | 0.38 | 0.09 (0.21) | 0.65 | 0.006 (0.01) | 0.64 | 0.08 (0.10) | 0.44 |
| **IN6** | | | | | | | | | | | | | | | | | | | | |
| *t*=base | 1041 | 0.21 (0.06) | <0.001 | 0.02 (0.01) | 0.27 | 0.02 (0.03) | 0.52 | 1050 | 0.24 (0.10) | 0.02 | 0.01 (0.01) | 0.30 | 0.05 (0.05) | 0.38 | 0.52 (0.16) | 0.001 | 0.02 (0.01) | 0.18 | 0.08 (0.08) | 0.29 |
| *t*=fu | 1038 | 0.25 (0.06) | <0.001 | 0.02 (0.01) | 0.29 | -0.02 (0.03) | 0.46 | 1050 | 0.31 (0.09) | 0.001 | 0.01 (0.01) | 0.32 | -0.08 (0.05) | 0.12 | 0.47 (0.16) | 0.003 | 0.02 (0.01) | 0.20 | 0.02 (0.08) | 0.75 |
| **IN7** | | | | | | | | | | | | | | | | | | | | |
| *t*=base | 1042 | 0.12 (0.06) | 0.06 | 0.01 (0.01) | 0.41 | -0.01 (0.03) | 0.75 | 1051 | 0.25 (0.12) | 0.03 | 0.01 (0.01) | 0.37 | 0.09 (0.06) | 0.13 | 0.65 (0.18) | <0.001 | 0.01 (0.01) | 0.28 | 0.11 (0.09) | 0.20 |
| *t*=fu | 1039 | 0.19 (0.06) | 0.003 | 0.01 (0.01) | 0.36 | 0.007 (0.03) | 0.82 | 1051 | 0.22 (0.11) | 0.05 | 0.01 (0.01) | 0.43 | -0.05 (0.05) | 0.34 | 0.39 (0.18) | 0.03 | 0.01 (0.01) | 0.37 | 0.08 (0.08) | 0.36 |
| **Self-report injunctive norms (Average IN1 to IN7)** | | | | | | | | | | | | | | | | | | | | |
| *t*=base | 1036 | 0.08 (0.06) | 0.18 | 0.009 (0.008) | 0.21 | 0.04 (0.03) | 0.29 | 1046 | 0.10 (0.11) | 0.36 | 0.01 (0.008) | 0.17 | 0.12 (0.06) | 0.06 | 0.46 (0.17) | 0.009 | 0.01 (0.008) | 0.16 | 0.10 (0.08) | 0.23 |
| *t*=fu | 1034 | 0.20 (0.05) | <0.001 | 0.01 (0.008) | 0.20 | -0.03 (0.03) | 0.35 | 1046 | 0.20 (0.09) | 0.03 | 0.009 (0.007) | 0.23 | -0.01 (0.05) | 0.82 | 0.28 (0.16) | 0.07 | 0.01 (0.008) | 0.19 | 0.10 (0.08) | 0.20 |
| **DN1.1** | | | | | | | | | | | | | | | | | | | | |
| *t*=base | 1042 | 0.27 (0.10) | 0.005 | 0.03 (0.01) | 0.009 | -0.08 (0.06) | 0.15 | 1051 | 0.19 (0.13) | 0.14 | 0.03 (0.01) | 0.01 | -0.14 (0.08) | 0.08 | 1.02 (0.31) | 0.001 | 0.03 (0.01) | 0.007 | -0.16 (0.14) | 0.23 |
| *t*=fu | 1039 | 0.31 (0.09) | <0.001 | 0.03 (0.01) | 0.01 | -0.001 (0.05) | 0.98 | 1051 | 0.19 (0.13) | 0.14 | 0.03 (0.01) | 0.01 | -0.07 (0.07) | 0.35 | 0.59 (0.18) | 0.001 | 0.03 (0.01) | 0.01 | -0.12 (0.10) | 0.26 |
| **DN1.2** | | | | | | | | | | | | | | | | | | | | |
| *t*=base | 1043 | 0.12 (0.05) | 0.02 | 0.01 (0.01) | 0.19 | -0.02 (0.03) | 0.43 | 1052 | 0.08 (0.07) | 0.25 | 0.01 (0.01) | 0.20 | -0.03 (0.04) | 0.47 | 0.13 (0.09) | 0.14 | 0.01 (0.01) | 0.24 | -0.04 (0.04) | 0.24 |
| *t*=fu | 1040 | -0.001 (0.04) | 0.97 | 0.02 (0.01) | 0.16 | -0.04 (0.03) | 0.09 | 1052 | 0.02 (0.08) | 0.77 | 0.01 (0.01) | 0.22 | -0.04 (0.04) | 0.33 | 0.08 (0.09) | 0.41 | 0.01 (0.01) | 0.24 | -0.05 (0.04) | 0.22 |
| **DN1.3** | | | | | | | | | | | | | | | | | | | | |
| *t*=base | 1042 | 0.08 (0.04) | 0.09 | -0.005 (0.01) | 0.76 | 0.02 (0.02) | 0.39 | 1051 | 0.06 (0.08) | 0.47 | -0.005 (0.01) | 0.73 | 0.01 (0.04) | 0.81 | 0.12 (0.13) | 0.36 | -0.006 (0.01) | 0.70 | -0.02 (0.06) | 0.72 |
| *t*=fu | 1039 | 0.02 (0.05) | 0.68 | -0.004 (0.02) | 0.77 | -0.02 (0.03) | 0.58 | 1051 | 0.03 (0.08) | 0.74 | -0.006 (0.01) | 0.71 | -0.008 (0.04) | 0.84 | 0.06 (0.15) | 0.71 | -0.006 (0.01) | 0.68 | -0.05 (0.07) | 0.46 |
| **DN1.4** | | | | | | | | | | | | | | | | | | | | |
| *t*=base | 1042 | 0.003 (0.04) | 0.94 | 0.01 (0.01) | 0.35 | 0.0008 (0.02) | 0.97 | 1051 | 0.04 (0.13) | 0.77 | 0.01 (0.01) | 0.36 | -0.08 (0.07) | 0.28 | 0.12 (0.15) | 0.42 | 0.01 (0.01) | 0.31 | -0.11 (0.07) | 0.15 |
| *t*=fu | 1039 | -0.08 (0.04) | 0.03 | 0.01 (0.01) | 0.25 | 0.008 (0.03) | 0.80 | 1051 | 0.05 (0.13) | 0.68 | 0.01 (0.01) | 0.33 | -0.10 (0.09) | 0.25 | -0.07 (0.18) | 0.71 | 0.01 (0.01) | 0.32 | -0.13 (0.09) | 0.15 |
| **DN1.5** | | | | | | | | | | | | | | | | | | | | |
| *t*=base | 1042 | 0.09 (0.05) | 0.10 | 0.006 (0.008) | 0.47 | -0.04 (0.04) | 0.26 | 1051 | 0.08 (0.08) | 0.35 | 0.006 (0.008) | 0.49 | -0.04 (0.05) | 0.48 | 0.33 (0.18) | 0.08 | 0.007 (0.009) | 0.43 | 0.01 (0.09) | 0.87 |
| *t*=fu | 1039 | -0.02 (0.04) | 0.56 | 0.007 (0.009) | 0.41 | 0.02 (0.02) | 0.50 | 1051 | 0.09 (0.08) | 0.27 | 0.006 (0.008) | 0.48 | -0.02 (0.05) | 0.71 | 0.11 (0.16) | 0.51 | 0.007 (0.009) | 0.43 | 0.04 (0.07) | 0.60 |
| **Self-report descriptive norms 1 (Average DN1.1 to DN1.5)** | | | | | | | | | | | | | | | | | | | | |
| *t*=base | 1040 | 0.10 (0.05) | 0.05 | 0.009 (0.007) | 0.19 | -0.008 (0.03) | 0.78 | 1049 | 0.05 (0.07) | 0.45 | 0.009 (0.007) | 0.18 | -0.02 (0.04) | 0.58 | 0.16 (0.11) | 0.12 | 0.009 (0.007) | 0.19 | -0.05 (0.05) | 0.29 |
| *t*=fu | 1037 | 0.04 (0.05) | 0.43 | 0.01 (0.007) | 0.16 | -0.03 (0.03) | 0.30 | 1049 | 0.01 (0.07) | 0.86 | 0.009 (0.007) | 0.17 | -0.01 (0.04) | 0.73 | 0.11 (0.10) | 0.26 | 0.009 (0.007) | 0.20 | -0.05 (0.05) | 0.24 |
| **DN2.1** | | | | | | | | | | | | | | | | | | | | |
| *t*=base | 1043 | 0.17 (0.07) | 0.01 | 0.03 (0.01) | 0.02 | -0.08 (0.05) | 0.10 | 1052 | 0.14 (0.11) | 0.17 | 0.03 (0.01) | 0.02 | -0.02 (0.07) | 0.76 | 0.23 (0.15) | 0.12 | 0.03 (0.01) | 0.02 | -0.05 (0.09) | 0.53 |
| *t*=fu | 1040 | 0.19 (0.06) | 0.004 | 0.02 (0.01) | 0.04 | -0.02 (0.04) | 0.50 | 1052 | 0.19 (0.11) | 0.10 | 0.03 (0.01) | 0.02 | -0.03 (0.07) | 0.64 | 0.14 (0.17) | 0.40 | 0.03 (0.01) | 0.02 | -0.02 (0.08) | 0.79 |
| **DN2.2** | | | | | | | | | | | | | | | | | | | | |
| *t*=base | 1043 | 0.20 (0.06) | 0.002 | 0.03 (0.01) | 0.07 | -0.006 (0.03) | 0.86 | 1052 | 0.31 (0.10) | 0.002 | 0.03 (0.01) | 0.05 | 0.009 (0.05) | 0.86 | 0.50 (0.16) | 0.002 | 0.03 (0.01) | 0.08 | -0.05 (0.07) | 0.49 |
| *t*=fu | 1040 | 0.12 (0.06) | 0.05 | 0.03 (0.02) | 0.07 | -0.02 (0.03) | 0.54 | 1052 | 0.16 (0.09) | 0.09 | 0.03 (0.01) | 0.06 | 0.03 (0.05) | 0.56 | 0.39 (0.15) | 0.01 | 0.03 (0.01) | 0.07 | -0.05 (0.07) | 0.43 |
| **DN2.3** | | | | | | | | | | | | | | | | | | | | |
| *t*=base | 1043 | 0.27 (0.07) | <0.001 | 0.01 (0.01) | 0.32 | 0.03 (0.04) | 0.48 | 1052 | 0.62 (0.10) | <0.001 | 0.01 (0.01) | 0.36 | 0.02 (0.05) | 0.66 | 0.70 (0.13) | <0.001 | 0.01 (0.01) | 0.39 | 0.06 (0.07) | 0.33 |
| *t*=fu | 1040 | 0.18 (0.06) | 0.002 | 0.006 (0.01) | 0.57 | 0.04 (0.04) | 0.23 | 1052 | 0.42 (0.09) | <0.001 | 0.008 (0.01) | 0.48 | 0.10 (0.05) | 0.04 | 0.58 (0.12) | <0.001 | 0.008 (0.01) | 0.46 | 0.11 (0.06) | 0.08 |
| **Self-report descriptive norms 2 (Average DN2.1 to DN2.3)** | | | | | | | | | | | | | | | | | | | | |
| *t*=base | 1043 | 0.24 (0.07) | <0.001 | 0.02 (0.009) | 0.02 | -0.03 (0.04) | 0.45 | 1052 | 0.38 (0.10) | <0.001 | 0.02 (0.009) | 0.02 | -0.01 (0.05) | 0.85 | 0.48 (0.14) | <0.001 | 0.02 (0.009) | 0.02 | -0.04 (0.07) | 0.53 |
| *t*=fu | 1040 | 0.11 (0.06) | 0.06 | 0.02 (0.009) | 0.04 | 0.004 (0.03) | 0.91 | 1052 | 0.26 (0.09) | 0.003 | 0.02 (0.009) | 0.03 | 0.009 (0.05) | 0.85 | 0.39 (0.13) | 0.002 | 0.02 (0.009) | 0.02 | -0.02 (0.06) | 0.78 |
| **Self-report smoking behavior** | | | | | | | | | | | | | | | | | | | | |
| *t*=base | 1048 | 0.14 (0.07) | 0.05 | 0.01 (0.01) | 0.21 | -0.04 (0.04) | 0.32 | 1058 | 0.23 (0.11) | 0.04 | 0.01 (0.01) | 0.24 | 0.02 (0.06) | 0.78 | 0.59 (0.17) | 0.001 | 0.01 (0.01) | 0.16 | -0.08 (0.09) | 0.40 |
| *t*=fu | 1046 | 0.24 (0.06) | <0.001 | 0.01 (0.01) | 0.18 | -0.007 (0.05) | 0.87 | 1058 | 0.22 (0.09) | 0.01 | 0.01 (0.01) | 0.26 | 0.005 (0.05) | 0.92 | 0.40 (0.11) | <0.001 | 0.01 (0.01) | 0.18 | -0.08 (0.06) | 0.22 |
| **Intentions** | | | | | | | | | | | | | | | | | | | | |
| *t*=base | 1044 | 0.17 (0.08) | 0.04 | 0.008 (0.02) | 0.68 | -0.09 (0.05) | 0.06 | 1053 | 0.50 (0.14) | <0.001 | 0.01 (0.02) | 0.60 | -0.13 (0.08) | 0.10 | 0.98 (0.26) | <0.001 | 0.02 (0.02) | 0.41 | -0.08 (0.13) | 0.55 |
| *t*=fu | 1041 | 0.21 (0.08) | 0.008 | 0.01 (0.02) | 0.49 | -0.03 (0.04) | 0.51 | 1053 | 0.11 (0.11) | 0.31 | 0.01 (0.02) | 0.56 | -0.006 (0.06) | 0.92 | 0.37 (0.15) | 0.01 | 0.01 (0.02) | 0.48 | -0.008 (0.07) | 0.91 |
| **Knowledge** | | | | | | | | | | | | | | | | | | | | |
| *t*=base | 1047 | 0.20 (0.06) | <0.001 | 0.04 (0.02) | 0.12 | 0.05 (0.03) | 0.10 | 1056 | 0.41 (0.08) | <0.001 | 0.04 (0.02) | 0.08 | 0.01 (0.04) | 0.76 | 0.46 (0.10) | <0.001 | 0.05 (0.02) | 0.05 | 0.04 (0.05) | 0.42 |
| *t*=fu | 1044 | 0.29 (0.06) | <0.001 | 0.03 (0.02) | 0.22 | 0.002 (0.03) | 0.95 | 1056 | 0.41 (0.08) | <0.001 | 0.03 (0.02) | 0.15 | 0.004 (0.04) | 0.92 | 0.44 (0.11) | <0.001 | 0.05 (0.02) | 0.05 | 0.06 (0.05) | 0.19 |
| **Attitudes** | | | | | | | | | | | | | | | | | | | | |
| *t*=base | 1032 | 0.15 (0.06) | 0.009 | 0.02 (0.01) | 0.06 | -0.04 (0.03) | 0.26 | 1041 | -0.04 (0.11) | 0.74 | 0.02 (0.01) | 0.05 | -0.04 (0.06) | 0.49 | 0.36 (0.20) | 0.08 | 0.02 (0.01) | 0.03 | -0.07 (0.11) | 0.49 |
| *t*=fu | 1029 | 0.13 (0.06) | 0.04 | 0.02 (0.01) | 0.05 | -0.002 (0.04) | 0.95 | 1041 | -0.14 (0.12) | 0.22 | 0.02 (0.01) | 0.05 | 0.005 (0.06) | 0.94 | 0.21 (0.17) | 0.21 | 0.02 (0.01) | 0.05 | -0.03 (0.09) | 0.77 |
| **Self-efficacy (Emotional)** | | | | | | | | | | | | | | | | | | | | |
| *t*=base | 1040 | 0.13 (0.08) | 0.11 | 0.02 (0.01) | 0.22 | 0.04 (0.04) | 0.33 | 1049 | 0.32 (0.12) | 0.009 | 0.02 (0.01) | 0.17 | -0.01 (0.07) | 0.87 | 0.51 (0.17) | 0.002 | 0.02 (0.01) | 0.11 | -0.07 (0.09) | 0.41 |
| *t*=fu | 1036 | 0.27 (0.07) | <0.001 | 0.02 (0.01) | 0.26 | -0.04 (0.04) | 0.34 | 1049 | 0.24 (0.09) | 0.008 | 0.02 (0.01) | 0.20 | 0.005 (0.05) | 0.91 | 0.46 (0.13) | 0.001 | 0.02 (0.01) | 0.11 | -0.08 (0.07) | 0.23 |
| **Self-efficacy (Friends)** | | | | | | | | | | | | | | | | | | | | |
| *t*=base | 1045 | 0.08 (0.07) | 0.27 | 0.01 (0.01) | 0.46 | -0.04 (0.04) | 0.34 | 1054 | 0.20 (0.12) | 0.08 | 0.009 (0.01) | 0.47 | -0.01 (0.06) | 0.81 | 0.20 (0.17) | 0.23 | 0.01 (0.01) | 0.39 | -0.13 (0.08) | 0.10 |
| *t*=fu | 1042 | 0.20 (0.07) | 0.005 | 0.006 (0.01) | 0.63 | 0.04 (0.04) | 0.27 | 1054 | 0.13 (0.10) | 0.19 | 0.009 (0.01) | 0.46 | 0.04 (0.05) | 0.43 | 0.27 (0.18) | 0.14 | 0.01 (0.01) | 0.36 | -0.11 (0.08) | 0.19 |
| **Self-efficacy (Opportunity)** | | | | | | | | | | | | | | | | | | | | |
| *t*=base | 1046 | 0.14 (0.08) | 0.08 | 0.008 (0.01) | 0.50 | -0.02 (0.04) | 0.61 | 1055 | 0.37 (0.15) | 0.01 | 0.008 (0.01) | 0.49 | -0.02 (0.07) | 0.79 | 0.44 (0.21) | 0.04 | 0.01 (0.01) | 0.35 | -0.13 (0.10) | 0.19 |
| *t*=fu | 1043 | 0.15 (0.07) | 0.04 | 0.009 (0.01) | 0.48 | 0.006 (0.04) | 0.88 | 1055 | 0.22 (0.10) | 0.04 | 0.008 (0.01) | 0.49 | 0.008 (0.05) | 0.86 | 0.32 (0.17) | 0.06 | 0.01 (0.01) | 0.36 | -0.03 (0.08) | 0.67 |
| **Perceived physical risks** | | | | | | | | | | | | | | | | | | | | |
| *t*=base | 1039 | 0.08 (0.06) | 0.14 | 0.49 (0.35) | 0.16 | -0.03 (0.03) | 0.31 | 1047 | 0.15 (0.09) | 0.08 | 0.49 (0.35) | 0.16 | -0.05 (0.05) | 0.25 | **0.10 (0.14)** | **0.47** | **0.49 (0.35)** | **0.16** | **-0.22 (0.07)** | **0.003** |
| *t*=fu | 1035 | 0.11 (0.06) | 0.04 | 0.47 (0.35) | 0.18 | 0.01 (0.03) | 0.68 | 1047 | 0.12 (0.11) | 0.29 | 0.46 (0.35) | 0.19 | -0.04 (0.05) | 0.48 | **-0.03 (0.21)** | **0.89** | **0.40 (0.35)** | **0.25** | **-0.25 (0.08)** | **0.003** |
| **Perceived social risks** | | | | | | | | | | | | | | | | | | | | |
| *t*=base | 1042 | 0.15 (0.05) | 0.005 | 0.58 (0.40) | 0.15 | 0.008 (0.03) | 0.79 | 1051 | 0.27 (0.08) | <0.001 | 0.61 (0.40) | 0.13 | 0.02 (0.04) | 0.58 | 0.42 (0.10) | <0.001 | 0.67 (0.40) | 0.09 | -0.01 (0.05) | 0.82 |
| *t*=fu | 1039 | 0.13 (0.06) | 0.03 | 0.47 (0.40) | 0.24 | 0.02 (0.03) | 0.46 | 1051 | 0.25 (0.09) | 0.004 | 0.51 (0.40) | 0.20 | -0.02 (0.04) | 0.67 | 0.42 (0.12) | 0.001 | 0.62 (0.40) | 0.12 | -0.006 (0.06) | 0.91 |
| **Perceived addiction risks** | | | | | | | | | | | | | | | | | | | | |
| *t*=base | 984 | 0.22 (0.06) | <0.001 | 0.10 (0.43) | 0.81 | 0.04 (0.03) | 0.14 | 993 | 0.50 (0.09) | <0.001 | 0.24 (0.42) | 0.57 | 0.04 (0.04) | 0.31 | 0.72 (0.11) | <0.001 | 0.33 (0.42) | 0.43 | 0.03 (0.05) | 0.51 |
| *t*=fu | 977 | 0.15 (0.05) | 0.004 | 0.11 (0.43) | 0.81 | 0.02 (0.03) | 0.47 | 993 | 0.40 (0.08) | <0.001 | 0.20 (0.43) | 0.64 | 0.05 (0.04) | 0.19 | 0.58 (0.10) | <0.001 | 0.30 (0.42) | 0.47 | 0.02 (0.05) | 0.65 |
| **Perceived benefits** | | | | | | | | | | | | | | | | | | | | |
| *t*=base | 1000 | -0.02 (0.06) | 0.72 | 0.41 (0.36) | 0.26 | 0.04 (0.03) | 0.26 | 1009 | 0.12 (0.12) | 0.33 | 0.44 (0.36) | 0.22 | -0.02 (0.07) | 0.80 | 0.38 (0.21) | 0.08 | 0.41 (0.36) | 0.25 | -0.07 (0.11) | 0.55 |
| *t*=fu | 996 | 0.04 (0.06) | 0.50 | 0.50 (0.36) | 0.17 | 0.02 (0.03) | 0.61 | 1009 | -0.08 (0.13) | 0.50 | 0.42 (0.36) | 0.25 | 0.03 (0.07) | 0.65 | -0.17 (0.26) | 0.51 | 0.42 (0.36) | 0.24 | 0.09 (0.14) | 0.55 |
| **Perceived behavioral control (easy to quit)** | | | | | | | | | | | | | | | | | | | | |
| *t*=base | 1041 | 0.26 (0.06) | <0.001 | 0.006 (0.03) | 0.81 | 0.05 (0.03) | 0.11 | 1050 | 0.49 (0.08) | <0.001 | -0.0007 (0.02) | 0.98 | 0.06 (0.04) | 0.15 | 0.67 (0.10) | <0.001 | -0.002 (0.02) | 0.95 | 0.11 (0.05) | 0.03 |
| *t*=fu | 1038 | 0.17 (0.06) | 0.005 | 0.006 (0.03) | 0.82 | 0.06 (0.03) | 0.06 | 1050 | 0.50 (0.08) | <0.001 | -0.001 (0.02) | 0.96 | 0.08 (0.04) | 0.06 | **0.61 (0.10)** | **<0.001** | **-0.001 (0.02)** | **0.96** | **0.12 (0.05)** | **0.01** |
| **Perceived behavioral control (to avoid smoking)** | | | | | | | | | | | | | | | | | | | | |
| *t*=base | 1045 | 0.08 (0.07) | 0.30 | 0.05 (0.02) | 0.03 | -0.08 (0.04) | 0.04 | **1054** | **0.40 (0.14)** | **0.004** | **0.04 (0.02)** | **0.03** | **-0.20 (0.07)** | **0.005** | 0.84 (0.22) | <0.001 | 0.06 (0.02) | 0.006 | -0.14 (0.11) | 0.20 |
| *t*=fu | 1042 | 0.08 (0.07) | 0.26 | 0.05 (0.02) | 0.02 | -0.005 (0.04) | 0.90 | 1054 | 0.13 (0.14) | 0.35 | 0.05 (0.02) | 0.02 | -0.006 (0.07) | 0.93 | 0.60 (0.18) | 0.001 | 0.06 (0.02) | 0.007 | -0.09 (0.09) | 0.29 |
| **Objectively measured smoking behavior** | | | | | | | | | | | | | | | | | | | | |
| *t*=base | 1012 | 0.36 (0.05) | <0.001 | 0.04 (0.03) | 0.10 | -0.007 (0.02) | 0.68 | 1019 | 0.37 (0.06) | <0.001 | 0.05 (0.03) | 0.07 | -0.009 (0.02) | 0.64 | 0.58 (0.06) | <0.001 | 0.04 (0.03) | 0.13 | 0.004 (0.02) | 0.84 |
| *t*=fu | 993 | 0.48 (0.07) | <0.001 | 0.03 (0.03) | 0.21 | 0.04 (0.04) | 0.31 | 1019 | 0.84 (0.06) | <0.001 | 0.007 (0.02) | 0.75 | -0.002 (0.03) | 0.93 | 0.86 (0.06) | <0.001 | 0.02 (0.02) | 0.34 | 0.001 (0.03) | 0.97 |
| **Smoking susceptibility^e^** | | | | | | | | | | | | | | | | | | | | |
|  | n | **Percent_-i,t_^f^** | | **M^g^** | | **M*Percent_-i,t_^h^** | | n | **Percent_-i,t_^f^** | | **M^g^** | | **M*Percent_-i,t_^h^** | | **Percent_-i,t_^f^** | | **M^g^** | | **M*Percent_-i,t_^h^** | |
|  |  | OR (SE) | p-value | OR (SE) | p-value | OR (SE) | p-value |  | OR (SE) | p-value | OR (SE) | p-value | OR (SE) | p-value | OR (SE) | p-value | OR (SE) | p-value | OR (SE) | p-value |
| *t*=base | 1044 | 1.15 (0.04) | <0.001 | 0.97 (0.04) | 0.51 | 0.99 (0.02) | 0.60 | 1053 | 1.11 (0.07) | 0.09 | 0.97 (0.04) | 0.49 | 0.98 (0.03) | 0.49 | 1.26 (0.12) | 0.02 | 0.97 (0.04) | 0.45 | 0.93 (0.05) | 0.14 |
| *t*=fu | 1041 | 1.13 (0.03) | <0.001 | 0.97 (0.04) | 0.53 | 0.99 (0.02) | 0.51 | 1053 | 1.21 (0.07) | <0.001 | 0.97 (0.04) | 0.48 | 0.95 (0.03) | 0.10 | 1.35 (0.10) | <0.001 | 0.96 (0.04) | 0.34 | 0.96 (0.04) | 0.24 |

IV: Independent variable; DV: Dependent variable; Ave: average of peer group (*i*); M: moderator (Rule-following: 0 [0 balls allocated to blue bucket] to 5 [50 balls allocated to the blue bucket]); Percent: percentage of peer group (*-i*) classified as susceptible to commencing smoking; OR: odds ratio; SE: standard error.

^a^In each model the outcome variable is the focal participant’s (*i*) response to the relevant item at follow-up. The predictor variable is the average of the relevant group’s (*-i*) responses to the equivalent item at baseline (*t*=base) or follow-up (*t*=fu), where *–i*=(1) focal participant's nominated friends; (2) focal participant’s school class; (3) focal participant’s school year group. The moderator, and interaction of the moderator with the predictor variable, were also included as independent variables in all models. All models include robust (Huber White) standard errors specified using Stata’s ‘vce(robust)’ option. The following baseline variables are included as covariates in all models: gender (0=boy; 1=girl/prefer not to say), age (1=12 years or less; 2=13 years; 3=14 years or more), intervention (1=ASSIST; 2=Dead Cool), ethnicity (0=no ethnic minority; 1=ethnic minority), individuals' socio-economic status (NI: 1=NIMDM2017≤296.6; 2=296.6<NIMDM2017≤593.2; 3=NIMDM2017>593.2; Bogotá: 1=Informal settlement/Lowest/Low; 2=Middle-Low/Middle; 3=Middle-High/High), and baseline values of the outcome variable. The predictor variable, moderator variable, and baseline values of the outcome variable were mean-centered.

^b^Unstandardized regression coefficients representing the average change in the outcome variable for a one-unit increase in the predictor variable among participants who are average on rule-following.

^c^Unstandardized regression coefficients representing the average change in the outcome variable for a one-unit increase in rule-following (an increase of 10 balls allocated to the blue bucket) among participants who are average on the predictor variable.

^d^Unstandardized regression coefficients representing the average change in the association between the outcome variable and the predictor variable for a one-unit increase in rule-following (an increase of 10 balls allocated to the blue bucket).

^e^Logistic regressions were run for models including focal participants' smoking susceptibility as the outcome variable, with robust (Huber White) standard errors specified using Stata’s ‘vce(robust)’ option. The predictor variable is the percentage of the relevant group (*-i*) classified as susceptible to commencing smoking at baseline (*t*=base) or follow-up (*t*=fu), where *–i*=(1) focal participant's nominated friends; (2) focal participant’s school class; (3) focal participant’s school year group. The moderator, and interaction of the moderator with the predictor variable, were also included as independent variables in all models. The following baseline variables are included as covariates in all models: gender (0=boy; 1=girl/prefer not to say), age (1=12 years or less; 2=13 years; 3=14 years or more), intervention (1=ASSIST; 2=Dead Cool), ethnicity (0=no ethnic minority; 1=ethnic minority), individuals' socio-economic status (NI: 1=NIMDM2017≤296.6; 2=296.6<NIMDM2017≤593.2; 3=NIMDM2017>593.2; Bogotá: 1=Informal settlement/Lowest/Low; 2=Middle-Low/Middle; 3=Middle-High/High), and baseline values of the outcome variable. Results are odds ratios, standard errors, and p-values. The predictor variable, and moderator variable were mean-centered.

^f^Odds ratios representing the multiplicative change in odds of being susceptible to commencing smoking for a 10% increase in the number of nominated friends/pupils in the same school class/pupils in the same school year group classified as being susceptible to commencing smoking (1 out of 10 nominated friends/pupils in the same school class/pupils in the same school year group; predictor variable) among participants who are average on rule-following.

^g^Odds ratios representing the multiplicative change in odds of being susceptible to commencing smoking for a one-unit increase in rule-following (an increase of 10 balls allocated to the blue bucket) among participants who are average on the predictor variable.

^h^Ratio of ratios representing the ratio of: (1) the odds ratio representing the multiplicative change in odds of being susceptible to commencing smoking for a 10% increase in the number of nominated friends/pupils in the same school class/pupils in the same school year group classified as being susceptible to commencing smoking among participants who are one unit above average on rule-following; to (2) the odds ratio representing the multiplicative change in odds of being susceptible to commencing smoking for a 10% increase in the number of nominated friends/pupils in the same school class/pupils in the same school year group classified as being susceptible to commencing smoking among participants who are average on rule-following.

**Table S2.8.** Results of ordinary least squares linear regressions including interaction terms examining differences in peer influence effects according to pro-sociality for outcomes collected at follow-up.

|  | **Dependent variable: Participant responses to the outcome variable at follow-up^a^** | | | | | | | | | | | | | | | | | | | |
| --- | --- | --- | --- | --- | --- | --- | --- | --- | --- | --- | --- | --- | --- | --- | --- | --- | --- | --- | --- | --- |
|  | **(1) -i=Average of nominated friends** | | | | | | | n | **(2) -i=Average of school class** | | | | | | **(3) -i=Average of school year group** | | | | | |
| **IV** | n | **Ave_-i,t_^b^** | | **M^c^** | | **M*Ave_-i,t_^d^** | |  | **Ave_-i,t_^b^** | | **M^c^** | | **M*Ave_-i,t_^d^** | | **Ave_-i,t_^b^** | | **M^c^** | | **M*Ave_-i,t_^d^** | |
| **DV** |  | *b* (SE) | p-value | *b* (SE) | p-value | *b* (SE) | p-value |  | *b* (SE) | p-value | *b* (SE) | p-value | *b* (SE) | p-value | *b* (SE) | p-value | *b* (SE) | p-value | *b* (SE) | p-value |
| **P2S2** | | | | | | | | | | | | | | | | | | | | |
| *t*=base | 1058 | 0.34 (0.09) | <0.001 | -0.005 (0.005) | 0.33 | -0.02 (0.04) | 0.68 | 1071 | 0.49 (0.12) | <0.001 | -0.006 (0.005) | 0.24 | -0.01 (0.06) | 0.79 | 0.86 (0.19) | <0.001 | -0.009 (0.005) | 0.10 | -0.04 (0.09) | 0.63 |
| *t*=fu | 1005 | 0.18 (0.07) | 0.008 | -0.008 (0.005) | 0.12 | -0.07 (0.04) | 0.12 | 1071 | 0.32 (0.10) | 0.002 | -0.006 (0.005) | 0.18 | -0.07 (0.05) | 0.12 | 0.63 (0.17) | <0.001 | -0.008 (0.005) | 0.15 | -0.05 (0.09) | 0.54 |
| **P2S3** | | | | | | | | | | | | | | | | | | | | |
| *t*=base | 1057 | 0.10 (0.07) | 0.14 | -0.01 (0.006) | 0.05 | 0.03 (0.03) | 0.38 | 1070 | 0.22 (0.16) | 0.16 | -0.009 (0.005) | 0.08 | 0.07 (0.07) | 0.31 | 0.12 (0.28) | 0.66 | -0.009 (0.005) | 0.09 | 0.10 (0.13) | 0.43 |
| *t*=fu | 1004 | 0.13 (0.07) | 0.05 | -0.009 (0.005) | 0.10 | 0.03 (0.03) | 0.37 | 1070 | 0.25 (0.14) | 0.08 | -0.009 (0.005) | 0.09 | 0.05 (0.06) | 0.43 | 0.29 (0.27) | 0.29 | -0.01 (0.005) | 0.08 | -0.16 (0.14) | 0.27 |
| **P2S4** | | | | | | | | | | | | | | | | | | | | |
| *t*=base | 1055 | 0.13 (0.07) | 0.08 | -0.02 (0.006) | 0.002 | 0.03 (0.04) | 0.38 | 1068 | 0.16 (0.13) | 0.22 | -0.02 (0.006) | 0.003 | 0.09 (0.07) | 0.23 | 0.40 (0.35) | 0.25 | -0.02 (0.006) | 0.004 | 0.15 (0.18) | 0.40 |
| *t*=fu | 1003 | 0.11 (0.07) | 0.10 | -0.02 (0.006) | 0.002 | -0.02 (0.03) | 0.53 | 1068 | 0.25 (0.12) | 0.03 | -0.02 (0.006) | 0.007 | 0.05 (0.07) | 0.46 | 0.33 (0.22) | 0.13 | -0.02 (0.007) | 0.02 | 0.03 (0.11) | 0.82 |
| **P2S5** | | | | | | | | | | | | | | | | | | | | |
| *t*=base | 1058 | 0.23 (0.06) | <0.001 | -0.009 (0.006) | 0.15 | 0.06 (0.03) | 0.02 | 1071 | 0.36 (0.10) | <0.001 | -0.01 (0.006) | 0.04 | 0.03 (0.05) | 0.57 | 0.53 (0.15) | <0.001 | -0.01 (0.006) | 0.03 | 0.01 (0.07) | 0.85 |
| *t*=fu | 1005 | 0.18 (0.06) | 0.001 | -0.02 (0.006) | 0.01 | 0.02 (0.03) | 0.43 | 1071 | 0.48 (0.09) | <0.001 | -0.01 (0.006) | 0.02 | 0.02 (0.04) | 0.55 | 0.48 (0.14) | 0.001 | -0.01 (0.006) | 0.03 | -0.02 (0.06) | 0.80 |
| **P2S6** | | | | | | | | | | | | | | | | | | | | |
| *t*=base | 1056 | 0.16 (0.07) | 0.02 | -0.01 (0.007) | 0.04 | 0.01 (0.03) | 0.77 | 1069 | 0.36 (0.11) | 0.001 | -0.01 (0.007) | 0.05 | 0.01 (0.05) | 0.84 | 0.41 (0.19) | 0.03 | -0.01 (0.007) | 0.05 | 0.15 (0.08) | 0.04 |
| *t*=fu | 1003 | 0.15 (0.06) | 0.009 | -0.01 (0.007) | 0.07 | 0.006 (0.02) | 0.77 | 1069 | 0.43 (0.10) | <0.001 | -0.01 (0.007) | 0.05 | 0.009 (0.05) | 0.86 | **0.45 (0.15)** | **0.003** | **-0.01 (0.007)** | **0.06** | **0.18 (0.07)** | **0.01** |
| **P2S7** | | | | | | | | | | | | | | | | | | | | |
| *t*=base | 1058 | 0.17 (0.05) | 0.002 | -0.004 (0.006) | 0.54 | 0.04 (0.03) | 0.20 | 1071 | 0.30 (0.09) | 0.001 | -0.004 (0.006) | 0.53 | 0.02 (0.03) | 0.53 | 0.29 (0.14) | 0.03 | -0.003 (0.006) | 0.55 | 0.13 (0.06) | 0.03 |
| *t*=fu | 1005 | 0.28 (0.06) | <0.001 | -0.006 (0.006) | 0.32 | -0.03 (0.02) | 0.18 | 1071 | 0.39 (0.09) | <0.001 | -0.004 (0.006) | 0.54 | 0.06 (0.04) | 0.10 | **0.28 (0.17)** | **0.10** | **-0.002 (0.006)** | **0.72** | **0.23 (0.07)** | **0.001** |
| **P2S8** | | | | | | | | | | | | | | | | | | | | |
| *t*=base | 1058 | 0.30 (0.05) | <0.001 | -0.01 (0.006) | 0.07 | 0.03 (0.03) | 0.25 | 1071 | 0.31 (0.09) | <0.001 | -0.01 (0.006) | 0.08 | 0.02 (0.04) | 0.56 | 0.43 (0.14) | 0.003 | -0.01 (0.006) | 0.12 | 0.03 (0.07) | 0.63 |
| *t*=fu | 1005 | 0.24 (0.05) | <0.001 | -0.01 (0.006) | 0.04 | 0.03 (0.02) | 0.14 | 1071 | 0.47 (0.08) | <0.001 | -0.01 (0.006) | 0.06 | 0.02 (0.03) | 0.65 | 0.47 (0.14) | 0.001 | -0.01 (0.006) | 0.10 | 0.10 (0.06) | 0.12 |
| **P2S9** | | | | | | | | | | | | | | | | | | | | |
| *t*=base | 1057 | 0.16 (0.07) | 0.02 | -0.008 (0.005) | 0.15 | 0.01 (0.03) | 0.72 | 1070 | 0.51 (0.13) | <0.001 | -0.009 (0.005) | 0.10 | -0.09 (0.06) | 0.15 | 0.58 (0.22) | 0.008 | -0.009 (0.006) | 0.11 | -0.009 (0.11) | 0.93 |
| *t*=fu | **1004** | **0.24 (0.06)** | **<0.001** | **-0.009 (0.005)** | **0.10** | **-0.08 (0.03)** | **0.009** | 1070 | 0.40 (0.11) | <0.001 | -0.008 (0.005) | 0.12 | -0.04 (0.06) | 0.53 | 0.22 (0.20) | 0.28 | -0.008 (0.005) | 0.14 | 0.03 (0.11) | 0.81 |
| **Experiment Part 2: Injunctive norms (Average P2S2 to P2S9)** | | | | | | | | | | | | | | | | | | | | |
| *t*=base | 1051 | 0.23 (0.06) | <0.001 | -0.007 (0.004) | 0.04 | 0.05 (0.03) | 0.09 | 1064 | 0.37 (0.10) | <0.001 | -0.007 (0.003) | 0.04 | 0.05 (0.05) | 0.29 | **0.41 (0.15)** | **0.005** | **-0.007 (0.003)** | **0.05** | **0.19 (0.07)** | **0.007** |
| *t*=fu | 1064 | 0.37 (0.10) | <0.001 | -0.007 (0.003) | 0.04 | 0.05 (0.05) | 0.29 | 1064 | 0.47 (0.08) | <0.001 | -0.007 (0.003) | 0.03 | 0.05 (0.04) | 0.27 | **0.30 (0.14)** | **0.03** | **-0.007 (0.003)** | **0.06** | **0.18 (0.07)** | **0.008** |
| **P3Q1** | | | | | | | | | | | | | | | | | | | | |
| *t*=base | 1058 | 0.10 (0.06) | 0.11 | -0.02 (0.007) | 0.002 | 0.03 (0.03) | 0.30 | 1071 | 0.26 (0.11) | 0.02 | -0.02 (0.008) | 0.003 | 0.03 (0.05) | 0.49 | 0.17 (0.15) | 0.25 | -0.02 (0.008) | 0.003 | 0.04 (0.07) | 0.56 |
| *t*=fu | 1005 | 0.09 (0.06) | 0.16 | -0.03 (0.008) | 0.001 | 0.02 (0.03) | 0.63 | 1071 | 0.29 (0.10) | 0.005 | -0.02 (0.008) | 0.003 | 0.06 (0.05) | 0.23 | 0.18 (0.17) | 0.31 | -0.02 (0.008) | 0.004 | 0.03 (0.08) | 0.71 |
| **P3Q2** | | | | | | | | | | | | | | | | | | | | |
| *t*=base | 1058 | 0.20 (0.05) | <0.001 | -0.02 (0.008) | 0.02 | 0.03 (0.03) | 0.34 | 1071 | 0.32 (0.08) | <0.001 | -0.02 (0.008) | 0.02 | 0.02 (0.04) | 0.61 | 0.34 (0.11) | 0.002 | -0.02 (0.008) | 0.02 | 0.01 (0.05) | 0.81 |
| *t*=fu | 1005 | 0.19 (0.06) | 0.001 | -0.02 (0.008) | 0.009 | -0.02 (0.03) | 0.41 | 1071 | 0.30 (0.08) | <0.001 | -0.02 (0.008) | 0.02 | 0.01 (0.04) | 0.82 | 0.28 (0.12) | 0.02 | -0.02 (0.008) | 0.02 | -0.003 (0.06) | 0.96 |
| **Experiment Part 3: Descriptive norms (Average P3Q1 to P3Q2)** | | | | | | | | | | | | | | | | | | | | |
| *t*=base | 1058 | 0.16 (0.06) | 0.005 | -0.02 (0.007) | 0.005 | 0.03 (0.03) | 0.30 | 1071 | 0.27 (0.09) | 0.002 | -0.02 (0.007) | 0.006 | 0.03 (0.05) | 0.58 | 0.23 (0.12) | 0.05 | -0.02 (0.007) | 0.005 | 0.03 (0.06) | 0.65 |
| *t*=fu | 1071 | 0.27 (0.09) | 0.002 | -0.02 (0.007) | 0.006 | 0.03 (0.05) | 0.58 | 1071 | 0.27 (0.09) | 0.002 | -0.02 (0.007) | 0.006 | 0.03 (0.05) | 0.52 | 0.18 (0.13) | 0.19 | -0.02 (0.007) | 0.006 | 0.01 (0.06) | 0.87 |
| **Donation to ASSIST/Dead Cool** | | | | | | | | | | | | | | | | | | | | |
| *t*=base | 1057 | 0.13 (0.06) | 0.02 | 0.005 (0.04) | 0.91 | 0.02 (0.02) | 0.37 | 1070 | 0.16 (0.09) | 0.08 | 0.02 (0.04) | 0.67 | -0.09 (0.04) | 0.04 | 0.33 (0.22) | 0.15 | 0.02 (0.04) | 0.63 | -0.07 (0.12) | 0.55 |
| *t*=fu | 1004 | 0.34 (0.06) | <0.001 | 0.04 (0.04) | 0.36 | -0.008 (0.03) | 0.81 | 1070 | 0.50 (0.09) | <0.001 | 0.03 (0.04) | 0.43 | -0.04 (0.04) | 0.31 | 0.70 (0.14) | <0.001 | 0.03 (0.04) | 0.49 | 0.05 (0.07) | 0.50 |
| **IN1** | | | | | | | | | | | | | | | | | | | | |
| *t*=base | 1061 | -0.04 (0.07) | 0.54 | 0.01 (0.01) | 0.18 | 0.04 (0.03) | 0.21 | 1070 | 0.04 (0.13) | 0.74 | 0.01 (0.01) | 0.24 | 0.01 (0.06) | 0.86 | 0.38 (0.22) | 0.08 | 0.01 (0.01) | 0.25 | -0.04 (0.11) | 0.72 |
| *t*=fu | 1059 | 0.07 (0.06) | 0.25 | 0.02 (0.01) | 0.14 | 0.03 (0.02) | 0.22 | 1070 | -0.19 (0.12) | 0.12 | 0.01 (0.01) | 0.19 | 0.10 (0.06) | 0.09 | 0.26 (0.23) | 0.26 | 0.01 (0.01) | 0.28 | 0.09 (0.10) | 0.37 |
| **IN2** | | | | | | | | | | | | | | | | | | | | |
| *t*=base | 1063 | -0.01 (0.08) | 0.86 | 0.004 (0.008) | 0.59 | 0.01 (0.02) | 0.54 | 1072 | 0.20 (0.19) | 0.29 | 0.003 (0.008) | 0.68 | 0.02 (0.08) | 0.81 | 0.17 (0.58) | 0.77 | 0.004 (0.008) | 0.61 | 0.24 (0.17) | 0.16 |
| *t*=fu | 1061 | 0.14 (0.08) | 0.08 | 0.003 (0.008) | 0.72 | -0.03 (0.03) | 0.43 | 1072 | 0.13 (0.11) | 0.24 | 0.003 (0.007) | 0.68 | -0.06 (0.06) | 0.32 | 0.06 (0.28) | 0.84 | 0.004 (0.008) | 0.63 | 0.10 (0.12) | 0.38 |
| **IN3** | | | | | | | | | | | | | | | | | | | | |
| *t*=base | 1061 | 0.11 (0.07) | 0.12 | 0.005 (0.01) | 0.59 | -0.01 (0.03) | 0.72 | 1070 | 0.20 (0.12) | 0.08 | 0.005 (0.009) | 0.60 | -0.06 (0.05) | 0.22 | 0.28 (0.19) | 0.13 | 0.004 (0.009) | 0.69 | -0.06 (0.07) | 0.38 |
| *t*=fu | 1059 | 0.08 (0.05) | 0.14 | 0.006 (0.01) | 0.53 | -0.05 (0.03) | 0.09 | 1070 | -0.01 (0.10) | 0.89 | 0.007 (0.01) | 0.48 | 0.01 (0.04) | 0.79 | 0.12 (0.20) | 0.56 | 0.006 (0.01) | 0.52 | 0.01 (0.07) | 0.87 |
| **IN4** | | | | | | | | | | | | | | | | | | | | |
| *t*=base | 1059 | 0.03 (0.06) | 0.65 | 0.003 (0.01) | 0.81 | -0.02 (0.03) | 0.57 | 1069 | -0.003 (0.13) | 0.98 | -0.00001 (0.01) | 1.00 | -0.10 (0.06) | 0.11 | 0.61 (0.32) | 0.06 | -0.0007 (0.01) | 0.96 | -0.15 (0.12) | 0.18 |
| *t*=fu | 1058 | 0.06 (0.05) | 0.30 | 0.001 (0.01) | 0.93 | -0.02 (0.03) | 0.44 | **1069** | **0.19 (0.11)** | **0.10** | **0.006 (0.01)** | **0.65** | **-0.16 (0.05)** | **0.002** | 0.40 (0.22) | 0.07 | 0.003 (0.01) | 0.82 | -0.11 (0.10) | 0.26 |
| **IN5** | | | | | | | | | | | | | | | | | | | | |
| *t*=base | 1061 | -0.03 (0.06) | 0.65 | 0.01 (0.01) | 0.28 | -0.04 (0.03) | 0.15 | 1070 | -0.06 (0.11) | 0.58 | 0.01 (0.01) | 0.37 | -0.05 (0.06) | 0.41 | -0.05 (0.23) | 0.85 | 0.01 (0.01) | 0.43 | -0.11 (0.12) | 0.36 |
| *t*=fu | 1059 | 0.03 (0.06) | 0.64 | 0.01 (0.01) | 0.37 | -0.02 (0.03) | 0.58 | 1070 | 0.03 (0.11) | 0.80 | 0.01 (0.01) | 0.29 | -0.08 (0.05) | 0.12 | 0.07 (0.20) | 0.71 | 0.01 (0.01) | 0.32 | -0.15 (0.10) | 0.13 |
| **IN6** | | | | | | | | | | | | | | | | | | | | |
| *t*=base | 1062 | 0.20 (0.06) | 0.001 | 0.02 (0.01) | 0.06 | -0.02 (0.03) | 0.42 | 1071 | 0.17 (0.10) | 0.09 | 0.02 (0.01) | 0.06 | 0.09 (0.05) | 0.07 | 0.40 (0.17) | 0.02 | 0.02 (0.01) | 0.09 | -0.02 (0.07) | 0.79 |
| *t*=fu | 1060 | 0.24 (0.06) | <0.001 | 0.02 (0.01) | 0.08 | -0.02 (0.02) | 0.45 | 1071 | 0.26 (0.09) | 0.005 | 0.02 (0.01) | 0.09 | -0.008 (0.04) | 0.84 | 0.37 (0.16) | 0.02 | 0.02 (0.01) | 0.10 | -0.01 (0.07) | 0.84 |
| **IN7** | | | | | | | | | | | | | | | | | | | | |
| *t*=base | 1063 | 0.10 (0.06) | 0.10 | 0.04 (0.01) | <0.001 | 0.009 (0.03) | 0.74 | 1072 | 0.16 (0.12) | 0.18 | 0.05 (0.01) | <0.001 | 0.12 (0.06) | 0.04 | 0.45 (0.18) | 0.02 | 0.04 (0.01) | <0.001 | 0.03 (0.08) | 0.71 |
| *t*=fu | 1061 | 0.18 (0.06) | 0.006 | 0.05 (0.01) | <0.001 | 0.03 (0.03) | 0.35 | 1072 | 0.18 (0.11) | 0.09 | 0.05 (0.01) | <0.001 | 0.02 (0.05) | 0.63 | 0.24 (0.18) | 0.19 | 0.05 (0.01) | <0.001 | 0.04 (0.07) | 0.53 |
| **Self-report injunctive norms (Average IN1 to IN7)** | | | | | | | | | | | | | | | | | | | | |
| *t*=base | 1056 | 0.07 (0.06) | 0.25 | 0.01 (0.007) | 0.13 | -0.01 (0.03) | 0.59 | 1066 | 0.06 (0.12) | 0.60 | 0.009 (0.007) | 0.18 | 0.001 (0.05) | 0.98 | 0.37 (0.18) | 0.04 | 0.007 (0.007) | 0.29 | -0.08 (0.07) | 0.26 |
| *t*=fu | 1066 | 0.06 (0.12) | 0.60 | 0.009 (0.007) | 0.18 | 0.001 (0.05) | 0.98 | 1066 | 0.18 (0.09) | 0.05 | 0.009 (0.007) | 0.20 | -0.04 (0.04) | 0.27 | 0.22 (0.16) | 0.15 | 0.008 (0.007) | 0.24 | -0.07 (0.06) | 0.27 |
| **DN1.1** | | | | | | | | | | | | | | | | | | | | |
| *t*=base | 1063 | 0.29 (0.10) | 0.004 | 0.02 (0.01) | 0.14 | 0.006 (0.05) | 0.89 | 1072 | 0.30 (0.15) | 0.04 | 0.01 (0.01) | 0.17 | 0.11 (0.07) | 0.09 | 1.03 (0.30) | 0.001 | 0.02 (0.01) | 0.12 | 0.16 (0.11) | 0.16 |
| *t*=fu | 1061 | 0.32 (0.09) | <0.001 | 0.02 (0.01) | 0.17 | 0.02 (0.05) | 0.64 | 1072 | 0.24 (0.13) | 0.07 | 0.01 (0.01) | 0.19 | 0.10 (0.07) | 0.14 | 0.59 (0.18) | 0.001 | 0.01 (0.01) | 0.16 | 0.10 (0.09) | 0.25 |
| **DN1.2** | | | | | | | | | | | | | | | | | | | | |
| *t*=base | 1064 | 0.12 (0.05) | 0.02 | -0.01 (0.01) | 0.16 | 0.03 (0.02) | 0.18 | 1073 | 0.04 (0.07) | 0.63 | -0.02 (0.01) | 0.10 | 0.07 (0.03) | 0.03 | 0.15 (0.09) | 0.11 | -0.01 (0.01) | 0.16 | 0.06 (0.03) | 0.10 |
| *t*=fu | 1062 | -0.01 (0.05) | 0.82 | -0.01 (0.009) | 0.11 | 0.01 (0.02) | 0.36 | 1073 | 0.002 (0.08) | 0.98 | -0.02 (0.01) | 0.10 | 0.08 (0.04) | 0.02 | 0.09 (0.09) | 0.33 | -0.01 (0.01) | 0.15 | 0.06 (0.04) | 0.11 |
| **DN1.3** | | | | | | | | | | | | | | | | | | | | |
| *t*=base | 1063 | 0.09 (0.04) | 0.04 | -0.002 (0.02) | 0.90 | 0.001 (0.02) | 0.94 | 1072 | 0.05 (0.08) | 0.55 | -0.002 (0.01) | 0.90 | 0.02 (0.04) | 0.58 | 0.11 (0.13) | 0.40 | -0.003 (0.02) | 0.82 | 0.07 (0.06) | 0.24 |
| *t*=fu | 1061 | 0.03 (0.05) | 0.62 | -0.003 (0.02) | 0.86 | 0.005 (0.02) | 0.79 | 1072 | 0.03 (0.08) | 0.67 | -0.002 (0.01) | 0.89 | 0.03 (0.03) | 0.44 | 0.01 (0.14) | 0.94 | -0.006 (0.01) | 0.69 | 0.12 (0.06) | 0.07 |
| **DN1.4** | | | | | | | | | | | | | | | | | | | | |
| *t*=base | 1063 | 0.01 (0.04) | 0.82 | 0.00008 (0.008) | 0.99 | -0.01 (0.02) | 0.44 | 1072 | 0.08 (0.14) | 0.57 | 0.003 (0.008) | 0.72 | -0.06 (0.04) | 0.15 | 0.14 (0.14) | 0.33 | 0.002 (0.008) | 0.83 | -0.08 (0.06) | 0.18 |
| *t*=fu | 1061 | -0.09 (0.04) | 0.03 | 0.001 (0.008) | 0.90 | -0.02 (0.01) | 0.11 | 1072 | 0.12 (0.16) | 0.47 | 0.002 (0.008) | 0.77 | -0.03 (0.04) | 0.48 | -0.08 (0.17) | 0.64 | 0.002 (0.008) | 0.80 | -0.07 (0.07) | 0.32 |
| **DN1.5** | | | | | | | | | | | | | | | | | | | | |
| *t*=base | 1063 | 0.08 (0.05) | 0.13 | 0.003 (0.006) | 0.60 | -0.0009 (0.02) | 0.96 | 1072 | 0.18 (0.12) | 0.13 | 0.003 (0.006) | 0.62 | -0.03 (0.05) | 0.52 | 0.34 (0.19) | 0.07 | 0.003 (0.006) | 0.67 | -0.08 (0.11) | 0.49 |
| *t*=fu | 1061 | -0.01 (0.05) | 0.84 | 0.003 (0.006) | 0.57 | -0.01 (0.02) | 0.60 | 1072 | 0.20 (0.11) | 0.08 | 0.004 (0.006) | 0.55 | -0.02 (0.06) | 0.69 | 0.11 (0.17) | 0.52 | 0.003 (0.006) | 0.59 | -0.07 (0.08) | 0.38 |
| **Self-report descriptive norms 1 (Average DN1.1 to DN1.5)** | | | | | | | | | | | | | | | | | | | | |
| *t*=base | 1061 | 0.10 (0.05) | 0.05 | -0.0003 (0.006) | 0.95 | -0.0001 (0.02) | 1.00 | 1070 | 0.04 (0.07) | 0.57 | -0.001 (0.006) | 0.86 | 0.05 (0.04) | 0.16 | 0.17 (0.11) | 0.10 | 0.0001 (0.006) | 0.98 | 0.03 (0.04) | 0.48 |
| *t*=fu | 1070 | 0.04 (0.07) | 0.57 | -0.001 (0.006) | 0.86 | 0.05 (0.04) | 0.16 | 1070 | 0.02 (0.08) | 0.81 | -0.0009 (0.006) | 0.87 | 0.06 (0.04) | 0.12 | 0.12 (0.10) | 0.25 | -0.0003 (0.006) | 0.95 | 0.05 (0.04) | 0.31 |
| **DN2.1** | | | | | | | | | | | | | | | | | | | | |
| *t*=base | 1064 | 0.17 (0.07) | 0.02 | 0.0008 (0.01) | 0.93 | 0.05 (0.03) | 0.13 | **1073** | **0.17 (0.10)** | **0.09** | **0.0009 (0.009)** | **0.92** | **0.12 (0.05)** | **0.009** | 0.23 (0.15) | 0.11 | 0.0002 (0.009) | 0.98 | 0.08 (0.06) | 0.16 |
| *t*=fu | 1062 | 0.19 (0.07) | 0.004 | 0.0007 (0.009) | 0.94 | 0.03 (0.03) | 0.25 | 1073 | 0.18 (0.11) | 0.11 | 0.002 (0.009) | 0.86 | 0.08 (0.04) | 0.05 | 0.12 (0.16) | 0.46 | 0.0007 (0.009) | 0.94 | 0.13 (0.06) | 0.03 |
| **DN2.2** | | | | | | | | | | | | | | | | | | | | |
| *t*=base | 1064 | 0.19 (0.06) | 0.004 | -0.004 (0.01) | 0.79 | 0.05 (0.02) | 0.04 | 1073 | 0.29 (0.10) | 0.003 | -0.001 (0.01) | 0.94 | 0.08 (0.04) | 0.07 | 0.49 (0.16) | 0.003 | -0.001 (0.01) | 0.93 | 0.11 (0.07) | 0.09 |
| *t*=fu | 1062 | 0.11 (0.06) | 0.07 | 0.0003 (0.01) | 0.98 | 0.02 (0.03) | 0.46 | 1073 | 0.17 (0.09) | 0.07 | -0.001 (0.01) | 0.92 | 0.06 (0.04) | 0.18 | 0.39 (0.15) | 0.009 | -0.001 (0.01) | 0.93 | 0.11 (0.06) | 0.08 |
| **DN2.3** | | | | | | | | | | | | | | | | | | | | |
| *t*=base | 1064 | 0.27 (0.08) | <0.001 | 0.01 (0.01) | 0.37 | -0.03 (0.05) | 0.49 | 1073 | 0.58 (0.10) | <0.001 | 0.008 (0.01) | 0.47 | -0.003 (0.05) | 0.95 | 0.67 (0.13) | <0.001 | 0.009 (0.01) | 0.39 | -0.005 (0.07) | 0.94 |
| *t*=fu | 1062 | 0.18 (0.05) | 0.001 | 0.008 (0.01) | 0.43 | -0.007 (0.04) | 0.85 | 1073 | 0.40 (0.09) | <0.001 | 0.007 (0.01) | 0.49 | -0.03 (0.05) | 0.52 | 0.55 (0.12) | <0.001 | 0.009 (0.01) | 0.42 | -0.02 (0.06) | 0.77 |
| **Self-report descriptive norms 2 (Average DN2.1 to DN2.3)** | | | | | | | | | | | | | | | | | | | | |
| *t*=base | 1064 | 0.23 (0.07) | 0.001 | 0.0007 (0.007) | 0.92 | 0.05 (0.03) | 0.07 | **1073** | **0.37 (0.10)** | **<0.001** | **0.0003 (0.007)** | **0.97** | **0.11 (0.04)** | **0.01** | 0.47 (0.13) | <0.001 | 0.0008 (0.007) | 0.92 | 0.09 (0.06) | 0.10 |
| *t*=fu | **1073** | **0.37 (0.10)** | **<0.001** | **0.0003 (0.007)** | **0.97** | **0.11 (0.04)** | **0.01** | 1073 | 0.26 (0.08) | 0.002 | 0.002 (0.007) | 0.83 | 0.05 (0.04) | 0.13 | 0.38 (0.12) | 0.002 | 0.001 (0.007) | 0.87 | 0.08 (0.05) | 0.12 |
| **Self-report smoking behavior** | | | | | | | | | | | | | | | | | | | | |
| *t*=base | 1067 | 0.17 (0.07) | 0.02 | 0.002 (0.008) | 0.79 | 0.02 (0.03) | 0.49 | 1076 | 0.22 (0.11) | 0.05 | 0.002 (0.008) | 0.85 | 0.004 (0.04) | 0.92 | 0.56 (0.17) | 0.001 | -0.0000 (0.008) | 1.00 | 0.05 (0.08) | 0.52 |
| *t*=fu | 1065 | 0.28 (0.06) | <0.001 | -0.0007 (0.008) | 0.93 | 0.04 (0.03) | 0.15 | 1076 | 0.20 (0.08) | 0.02 | 0.002 (0.007) | 0.74 | 0.08 (0.04) | 0.04 | 0.39 (0.10) | <0.001 | -0.0002 (0.008) | 0.98 | 0.04 (0.05) | 0.42 |
| **Intentions** | | | | | | | | | | | | | | | | | | | | |
| *t*=base | **1062** | **0.18 (0.08)** | **0.03** | **0.04 (0.02)** | **0.02** | **0.08 (0.03)** | **0.01** | 1071 | 0.45 (0.14) | 0.001 | 0.04 (0.02) | 0.02 | 0.08 (0.07) | 0.25 | 0.84 (0.25) | 0.001 | 0.03 (0.02) | 0.03 | 0.08 (0.11) | 0.45 |
| *t*=fu | 1060 | 0.21 (0.07) | 0.004 | 0.04 (0.02) | 0.01 | 0.05 (0.04) | 0.15 | 1071 | 0.07 (0.11) | 0.54 | 0.04 (0.02) | 0.009 | 0.05 (0.05) | 0.31 | 0.29 (0.15) | 0.05 | 0.04 (0.02) | 0.02 | 0.05 (0.06) | 0.41 |
| **Knowledge** | | | | | | | | | | | | | | | | | | | | |
| *t*=base | 1066 | 0.18 (0.05) | 0.001 | 0.05 (0.02) | 0.02 | -0.008 (0.02) | 0.72 | 1075 | 0.39 (0.08) | <0.001 | 0.04 (0.02) | 0.09 | -0.002 (0.03) | 0.95 | 0.41 (0.10) | <0.001 | 0.04 (0.02) | 0.06 | 0.005 (0.04) | 0.90 |
| *t*=fu | 1064 | 0.28 (0.06) | <0.001 | 0.05 (0.02) | 0.02 | 0.01 (0.02) | 0.55 | 1075 | 0.39 (0.08) | <0.001 | 0.04 (0.02) | 0.05 | 0.02 (0.03) | 0.43 | 0.37 (0.11) | 0.001 | 0.04 (0.02) | 0.06 | 0.002 (0.04) | 0.95 |
| **Attitudes** | | | | | | | | | | | | | | | | | | | | |
| *t*=base | 1052 | 0.17 (0.06) | 0.005 | 0.004 (0.009) | 0.65 | 0.03 (0.03) | 0.24 | 1061 | -0.03 (0.11) | 0.76 | 0.003 (0.009) | 0.73 | 0.003 (0.05) | 0.95 | 0.30 (0.21) | 0.17 | 0.002 (0.009) | 0.80 | -0.02 (0.09) | 0.84 |
| *t*=fu | 1050 | 0.17 (0.06) | 0.009 | -0.001 (0.009) | 0.87 | -0.001 (0.03) | 0.97 | 1061 | -0.11 (0.12) | 0.36 | 0.004 (0.009) | 0.68 | 0.03 (0.05) | 0.51 | 0.20 (0.18) | 0.24 | 0.002 (0.009) | 0.82 | -0.02 (0.07) | 0.72 |
| **Self-efficacy (Emotional)** | | | | | | | | | | | | | | | | | | | | |
| *t*=base | 1056 | 0.10 (0.08) | 0.21 | 0.02 (0.01) | 0.17 | -0.01 (0.03) | 0.69 | 1065 | 0.28 (0.12) | 0.02 | 0.02 (0.01) | 0.17 | -0.02 (0.07) | 0.82 | 0.42 (0.17) | 0.02 | 0.02 (0.01) | 0.20 | -0.11 (0.09) | 0.25 |
| *t*=fu | 1053 | 0.29 (0.07) | <0.001 | 0.02 (0.01) | 0.13 | 0.02 (0.02) | 0.34 | 1065 | 0.19 (0.09) | 0.03 | 0.02 (0.01) | 0.18 | -0.03 (0.04) | 0.53 | 0.37 (0.13) | 0.005 | 0.01 (0.01) | 0.32 | -0.10 (0.06) | 0.09 |
| **Self-efficacy (Friends)** | | | | | | | | | | | | | | | | | | | | |
| *t*=base | 1062 | 0.06 (0.06) | 0.39 | 0.01 (0.01) | 0.23 | -0.005 (0.03) | 0.85 | 1071 | 0.18 (0.11) | 0.11 | 0.02 (0.01) | 0.20 | -0.05 (0.06) | 0.33 | 0.21 (0.18) | 0.24 | 0.02 (0.01) | 0.19 | -0.20 (0.09) | 0.02 |
| *t*=fu | 1060 | 0.20 (0.07) | 0.003 | 0.02 (0.01) | 0.19 | 0.02 (0.03) | 0.55 | 1071 | 0.08 (0.10) | 0.43 | 0.02 (0.01) | 0.20 | -0.04 (0.04) | 0.38 | 0.20 (0.18) | 0.26 | 0.01 (0.01) | 0.31 | -0.19 (0.08) | 0.02 |
| **Self-efficacy (Opportunity)** | | | | | | | | | | | | | | | | | | | | |
| *t*=base | 1063 | 0.12 (0.08) | 0.10 | 0.02 (0.01) | 0.14 | -0.004 (0.03) | 0.90 | 1072 | 0.38 (0.15) | 0.01 | 0.02 (0.01) | 0.13 | -0.05 (0.08) | 0.50 | 0.44 (0.21) | 0.04 | 0.01 (0.01) | 0.17 | -0.21 (0.10) | 0.03 |
| *t*=fu | 1061 | 0.16 (0.07) | 0.03 | 0.02 (0.01) | 0.10 | -0.005 (0.03) | 0.88 | 1072 | 0.18 (0.10) | 0.07 | 0.02 (0.01) | 0.14 | -0.08 (0.04) | 0.05 | **0.24 (0.17)** | **0.16** | **0.01 (0.01)** | **0.22** | **-0.19 (0.07)** | **0.008** |
| **Perceived physical risks** | | | | | | | | | | | | | | | | | | | | |
| *t*=base | **1058** | **0.09 (0.05)** | **0.10** | **0.32 (0.32)** | **0.31** | **0.06 (0.02)** | **0.001** | 1066 | 0.14 (0.09) | 0.11 | 0.25 (0.32) | 0.43 | 0.06 (0.04) | 0.11 | 0.08 (0.15) | 0.60 | 0.25 (0.32) | 0.44 | -0.03 (0.07) | 0.64 |
| *t*=fu | 1055 | 0.12 (0.05) | 0.03 | 0.28 (0.32) | 0.38 | 0.02 (0.02) | 0.33 | **1066** | **0.13 (0.11)** | **0.26** | **0.35 (0.33)** | **0.29** | **0.10 (0.04)** | **0.01** | -0.21 (0.22) | 0.34 | 0.30 (0.32) | 0.35 | 0.05 (0.08) | 0.56 |
| **Perceived social risks** | | | | | | | | | | | | | | | | | | | | |
| *t*=base | 1060 | 0.16 (0.05) | 0.002 | 0.48 (0.40) | 0.22 | 0.04 (0.02) | 0.03 | 1069 | 0.25 (0.07) | 0.001 | 0.36 (0.40) | 0.37 | 0.05 (0.03) | 0.12 | 0.37 (0.10) | <0.001 | 0.27 (0.40) | 0.50 | 0.02 (0.05) | 0.76 |
| *t*=fu | 1058 | 0.13 (0.06) | 0.02 | 0.47 (0.39) | 0.23 | 0.009 (0.03) | 0.74 | 1069 | 0.22 (0.09) | 0.009 | 0.44 (0.40) | 0.28 | 0.08 (0.04) | 0.04 | 0.36 (0.12) | 0.003 | 0.31 (0.40) | 0.44 | 0.04 (0.06) | 0.49 |
| **Perceived addiction risks** | | | | | | | | | | | | | | | | | | | | |
| *t*=base | **1000** | **0.23 (0.06)** | **<0.001** | **0.54 (0.36)** | **0.14** | **0.06 (0.02)** | **0.008** | 1009 | 0.49 (0.09) | <0.001 | 0.34 (0.34) | 0.32 | 0.02 (0.04) | 0.49 | 0.71 (0.10) | <0.001 | 0.14 (0.34) | 0.67 | -0.01 (0.04) | 0.75 |
| *t*=fu | 994 | 0.14 (0.05) | 0.006 | 0.55 (0.36) | 0.13 | 0.004 (0.02) | 0.84 | 1009 | 0.38 (0.08) | <0.001 | 0.44 (0.35) | 0.21 | 0.04 (0.03) | 0.19 | 0.57 (0.09) | <0.001 | 0.23 (0.34) | 0.51 | 0.004 (0.04) | 0.92 |
| **Perceived benefits** | | | | | | | | | | | | | | | | | | | | |
| *t*=base | 1015 | -0.04 (0.06) | 0.51 | -0.41 (0.31) | 0.19 | 0.001 (0.03) | 0.97 | 1024 | 0.08 (0.12) | 0.51 | -0.38 (0.30) | 0.21 | 0.02 (0.06) | 0.69 | 0.32 (0.21) | 0.12 | -0.35 (0.30) | 0.24 | -0.03 (0.10) | 0.80 |
| *t*=fu | 1012 | 0.02 (0.06) | 0.75 | -0.42 (0.31) | 0.18 | 0.01 (0.03) | 0.64 | 1024 | -0.07 (0.12) | 0.56 | -0.40 (0.30) | 0.19 | 0.01 (0.06) | 0.83 | -0.13 (0.25) | 0.61 | -0.40 (0.31) | 0.20 | 0.02 (0.11) | 0.89 |
| **Perceived behavioral control (easy to quit)** | | | | | | | | | | | | | | | | | | | | |
| *t*=base | 1061 | 0.27 (0.06) | <0.001 | -0.02 (0.02) | 0.43 | 0.01 (0.03) | 0.59 | 1070 | 0.51 (0.08) | <0.001 | -0.006 (0.02) | 0.76 | -0.007 (0.04) | 0.85 | 0.70 (0.10) | <0.001 | 0.003 (0.02) | 0.88 | -0.05 (0.04) | 0.22 |
| *t*=fu | 1059 | 0.17 (0.06) | 0.004 | -0.02 (0.02) | 0.33 | 0.001 (0.03) | 0.97 | 1070 | 0.50 (0.08) | <0.001 | -0.007 (0.02) | 0.74 | -0.06 (0.03) | 0.05 | 0.62 (0.10) | <0.001 | 0.0008 (0.02) | 0.97 | -0.04 (0.04) | 0.29 |
| **Perceived behavioral control (to avoid smoking)** | | | | | | | | | | | | | | | | | | | | |
| *t*=base | 1064 | 0.08 (0.07) | 0.28 | 0.03 (0.02) | 0.05 | 0.06 (0.03) | 0.05 | 1073 | 0.35 (0.14) | 0.009 | 0.03 (0.02) | 0.07 | 0.09 (0.06) | 0.14 | 0.77 (0.22) | <0.001 | 0.03 (0.02) | 0.09 | 0.06 (0.09) | 0.50 |
| *t*=fu | 1062 | 0.07 (0.07) | 0.28 | 0.03 (0.02) | 0.07 | 0.04 (0.03) | 0.31 | 1073 | 0.08 (0.14) | 0.56 | 0.04 (0.02) | 0.04 | 0.14 (0.06) | 0.02 | 0.51 (0.18) | 0.004 | 0.03 (0.02) | 0.07 | 0.09 (0.08) | 0.24 |
| **Objectively measured smoking behavior** | | | | | | | | | | | | | | | | | | | | |
| *t*=base | 1025 | 0.35 (0.05) | <0.001 | -0.03 (0.03) | 0.24 | 0.003 (0.02) | 0.87 | 1032 | 0.36 (0.05) | <0.001 | -0.03 (0.02) | 0.26 | -0.01 (0.02) | 0.44 | 0.57 (0.06) | <0.001 | -0.02 (0.02) | 0.40 | -0.004 (0.02) | 0.84 |
| *t*=fu | 1007 | 0.48 (0.08) | <0.001 | -0.03 (0.02) | 0.14 | 0.03 (0.03) | 0.41 | 1032 | 0.84 (0.06) | <0.001 | -0.03 (0.02) | 0.18 | -0.02 (0.02) | 0.45 | 0.86 (0.06) | <0.001 | -0.02 (0.02) | 0.39 | -0.04 (0.03) | 0.11 |
| **Smoking susceptibility^e^** | | | | | | | | | | | | | | | | | | | | |
|  | n | **Percent_-i,t_^f^** | | **M^g^** | | **M*Percent_-i,t_^h^** | | n | **Percent_-i,t_^f^** | | **M^g^** | | **M*Percent_-i,t_^h^** | | **Percent_-i,t_^f^** | | **M^g^** | | **M*Percent_-i,t_^h^** | |
|  |  | OR (SE) | p-value | OR (SE) | p-value | OR (SE) | p-value |  | OR (SE) | p-value | OR (SE) | p-value | OR (SE) | p-value | OR (SE) | p-value | OR (SE) | p-value | OR (SE) | p-value |
| *t*=base | 1062 | 1.13 (0.04) | <0.001 | 0.87 (0.03) | 0.001 | 0.98 (0.02) | 0.32 | 1071 | 1.05 (0.06) | 0.45 | 0.86 (0.03) | <0.001 | 1.06 (0.03) | 0.04 | 1.12 (0.11) | 0.24 | 0.86 (0.03) | <0.001 | 1.04 (0.05) | 0.35 |
| *t*=fu | 1060 | 1.12 (0.03) | <0.001 | 0.87 (0.03) | <0.001 | 1.00 (0.01) | 0.99 | 1071 | 1.14 (0.06) | 0.01 | 0.87 (0.03) | <0.001 | 1.02 (0.02) | 0.38 | 1.23 (0.09) | 0.006 | 0.87 (0.03) | <0.001 | 1.02 (0.03) | 0.55 |

IV: Independent variable; DV: Dependent variable; Ave: average of peer group (*i*); M: moderator (Pro-sociality: 0 [least pro-social] to 10 [most pro-social]); Percent: percentage of peer group (*-i*) classified as susceptible to commencing smoking; OR: odds ratio; SE: standard error.

^a^In each model the outcome variable is the focal participant’s (*i*) response to the relevant item at follow-up. The predictor variable is the average of the relevant group’s (*-i*) responses to the equivalent item at baseline (*t*=base) or follow-up (*t*=fu), where *–i*=(1) focal participant's nominated friends; (2) focal participant’s school class; (3) focal participant’s school year group. The moderator, and interaction of the moderator with the predictor variable, were also included as independent variables in all models. All models include robust (Huber White) standard errors specified using Stata’s ‘vce(robust)’ option. The following baseline variables are included as covariates in all models: gender (0=boy; 1=girl/prefer not to say), age (1=12 years or less; 2=13 years; 3=14 years or more), intervention (1=ASSIST; 2=Dead Cool), ethnicity (0=no ethnic minority; 1=ethnic minority), individuals' socio-economic status (NI: 1=NIMDM2017≤296.6; 2=296.6<NIMDM2017≤593.2; 3=NIMDM2017>593.2; Bogotá: 1=Informal settlement/Lowest/Low; 2=Middle-Low/Middle; 3=Middle-High/High), and baseline values of the outcome variable. The predictor variable, moderator variable, and baseline values of the outcome variable were mean-centered.

^b^Unstandardized regression coefficients representing the average change in the outcome variable for a one-unit increase in the predictor variable among participants who are average on pro-sociality.

^c^Unstandardized regression coefficients representing the average change in the outcome variable for a one-unit increase in pro-sociality among participants who are average on the predictor variable.

^d^Unstandardized regression coefficients representing the average change in the association between the outcome variable and the predictor variable for a one-unit increase in pro-sociality.

^e^Logistic regressions were run for models including focal participants' smoking susceptibility as the outcome variable, with robust (Huber White) standard errors specified using Stata’s ‘vce(robust)’ option. The predictor variable is the percentage of the relevant group (*-i*) classified as susceptible to commencing smoking at baseline (*t*=base) or follow-up (*t*=fu), where *–i*=(1) focal participant's nominated friends; (2) focal participant’s school class; (3) focal participant’s school year group. The moderator, and interaction of the moderator with the predictor variable, were also included as independent variables in all models. The following baseline variables are included as covariates in all models: gender (0=boy; 1=girl/prefer not to say), age (1=12 years or less; 2=13 years; 3=14 years or more), intervention (1=ASSIST; 2=Dead Cool), ethnicity (0=no ethnic minority; 1=ethnic minority), individuals' socio-economic status (NI: 1=NIMDM2017≤296.6; 2=296.6<NIMDM2017≤593.2; 3=NIMDM2017>593.2; Bogotá: 1=Informal settlement/Lowest/Low; 2=Middle-Low/Middle; 3=Middle-High/High), and baseline values of the outcome variable. Results are odds ratios, standard errors, and p-values. The predictor variable, and moderator variable were mean-centered.

^f^Odds ratios representing the multiplicative change in odds of being susceptible to commencing smoking for a 10% increase in the number of nominated friends/pupils in the same school class/pupils in the same school year group classified as being susceptible to commencing smoking (1 out of 10 nominated friends/pupils in the same school class/pupils in the same school year group; predictor variable) among participants who are average on pro-sociality.

^g^Odds ratios representing the multiplicative change in odds of being susceptible to commencing smoking for a one-unit increase in pro-sociality among participants who are average on the predictor variable.

^h^Ratio of ratios representing the ratio of: (1) the odds ratio representing the multiplicative change in odds of being susceptible to commencing smoking for a 10% increase in the number of nominated friends/pupils in the same school class/pupils in the same school year group classified as being susceptible to commencing smoking among participants who are one unit above average on pro-sociality; to (2) the odds ratio representing the multiplicative change in odds of being susceptible to commencing smoking for a 10% increase in the number of nominated friends/pupils in the same school class/pupils in the same school year group classified as being susceptible to commencing smoking among participants who are average on pro-sociality.

**Table S2.9.** Results of ordinary least squares linear regressions including interaction terms examining differences in peer influence effects according to fear of negative evaluation for outcomes collected at follow-up.

|  | **Dependent variable: Participant responses to the outcome variable at follow-up^a^** | | | | | | | | | | | | | | | | | | | |
| --- | --- | --- | --- | --- | --- | --- | --- | --- | --- | --- | --- | --- | --- | --- | --- | --- | --- | --- | --- | --- |
|  | **(1) -i=Average of nominated friends** | | | | | | | n | **(2) -i=Average of school class** | | | | | | **(3) -i=Average of school year group** | | | | | |
| **IV** | n | **Ave_-i,t_^b^** | | **M^c^** | | **M*Ave_-i,t_^d^** | |  | **Ave_-i,t_^b^** | | **M^c^** | | **M*Ave_-i,t_^d^** | | **Ave_-i,t_^b^** | | **M^c^** | | **M*Ave_-i,t_^d^** | |
| **DV** |  | *b* (SE) | p-value | *b* (SE) | p-value | *b* (SE) | p-value |  | *b* (SE) | p-value | *b* (SE) | p-value | *b* (SE) | p-value | *b* (SE) | p-value | *b* (SE) | p-value | *b* (SE) | p-value |
| **P2S2** | | | | | | | | | | | | | | | | | | | | |
| *t*=base | 1042 | 0.34 (0.08) | <0.001 | 0.006 (0.01) | 0.63 | 0.22 (0.13) | 0.10 | 1055 | 0.44 (0.12) | <0.001 | 0.003 (0.01) | 0.80 | 0.01 (0.17) | 0.95 | 0.78 (0.18) | <0.001 | -0.003 (0.01) | 0.79 | 0.02 (0.22) | 0.92 |
| *t*=fu | 992 | 0.17 (0.06) | 0.005 | -0.001 (0.01) | 0.91 | -0.002 (0.09) | 0.99 | 1055 | 0.30 (0.10) | 0.003 | 0.004 (0.01) | 0.78 | -0.24 (0.14) | 0.09 | 0.57 (0.17) | 0.001 | -0.001 (0.01) | 0.94 | 0.02 (0.21) | 0.92 |
| **P2S3** | | | | | | | | | | | | | | | | | | | | |
| *t*=base | 1041 | 0.10 (0.07) | 0.18 | 0.003 (0.01) | 0.86 | 0.07 (0.10) | 0.46 | 1054 | 0.21 (0.16) | 0.18 | 0.002 (0.01) | 0.90 | 0.12 (0.24) | 0.63 | 0.08 (0.28) | 0.78 | 0.002 (0.01) | 0.87 | 0.45 (0.34) | 0.18 |
| *t*=fu | 991 | 0.12 (0.07) | 0.08 | -0.00004 (0.01) | 1.00 | -0.005 (0.09) | 0.96 | 1054 | 0.24 (0.14) | 0.08 | 0.0003 (0.01) | 0.99 | -0.15 (0.19) | 0.46 | 0.25 (0.26) | 0.35 | 0.001 (0.01) | 0.95 | 0.24 (0.35) | 0.48 |
| **P2S4** | | | | | | | | | | | | | | | | | | | | |
| *t*=base | 1039 | 0.12 (0.08) | 0.14 | 0.001 (0.01) | 0.94 | 0.16 (0.10) | 0.13 | 1052 | 0.14 (0.12) | 0.26 | -0.004 (0.01) | 0.76 | -0.04 (0.16) | 0.80 | 0.47 (0.32) | 0.14 | -0.005 (0.01) | 0.74 | -0.24 (0.25) | 0.36 |
| *t*=fu | 990 | 0.12 (0.07) | 0.07 | -0.004 (0.01) | 0.81 | -0.10 (0.10) | 0.32 | 1052 | 0.27 (0.12) | 0.02 | -0.006 (0.01) | 0.67 | -0.31 (0.13) | 0.02 | 0.42 (0.20) | 0.03 | -0.004 (0.01) | 0.80 | -0.34 (0.20) | 0.09 |
| **P2S5** | | | | | | | | | | | | | | | | | | | | |
| *t*=base | 1042 | 0.20 (0.06) | 0.001 | -0.02 (0.02) | 0.19 | 0.06 (0.09) | 0.54 | 1055 | 0.35 (0.10) | <0.001 | -0.03 (0.02) | 0.07 | 0.12 (0.15) | 0.43 | 0.51 (0.15) | 0.001 | -0.03 (0.02) | 0.08 | 0.09 (0.21) | 0.66 |
| *t*=fu | 992 | 0.18 (0.06) | 0.002 | -0.03 (0.02) | 0.11 | 0.06 (0.09) | 0.51 | 1055 | 0.48 (0.09) | <0.001 | -0.03 (0.02) | 0.06 | -0.03 (0.13) | 0.81 | 0.49 (0.14) | 0.001 | -0.03 (0.02) | 0.08 | 0.23 (0.19) | 0.22 |
| **P2S6** | | | | | | | | | | | | | | | | | | | | |
| *t*=base | **1041** | **0.18 (0.07)** | **0.006** | **-0.003 (0.02)** | **0.89** | **0.26 (0.10)** | **0.009** | 1054 | 0.37 (0.11) | 0.001 | -0.004 (0.02) | 0.86 | 0.32 (0.15) | 0.03 | **0.42 (0.19)** | **0.03** | **0.001 (0.02)** | **0.94** | **0.62 (0.22)** | **0.005** |
| *t*=fu | 991 | 0.17 (0.06) | 0.005 | -0.002 (0.02) | 0.90 | 0.16 (0.08) | 0.05 | 1054 | 0.43 (0.10) | <0.001 | -0.005 (0.02) | 0.79 | 0.09 (0.15) | 0.56 | 0.48 (0.15) | 0.002 | 0.001 (0.02) | 0.95 | 0.41 (0.18) | 0.02 |
| **P2S7** | | | | | | | | | | | | | | | | | | | | |
| *t*=base | 1042 | 0.18 (0.05) | 0.001 | 0.03 (0.02) | 0.08 | 0.07 (0.08) | 0.42 | 1055 | 0.30 (0.09) | 0.001 | 0.03 (0.02) | 0.09 | 0.15 (0.12) | 0.23 | 0.30 (0.14) | 0.03 | 0.03 (0.02) | 0.08 | 0.36 (0.16) | 0.03 |
| *t*=fu | 992 | 0.28 (0.06) | <0.001 | 0.03 (0.02) | 0.11 | -0.02 (0.09) | 0.84 | 1055 | 0.38 (0.09) | <0.001 | 0.03 (0.02) | 0.14 | 0.04 (0.13) | 0.77 | 0.30 (0.17) | 0.08 | 0.03 (0.02) | 0.06 | 0.41 (0.19) | 0.03 |
| **P2S8** | | | | | | | | | | | | | | | | | | | | |
| *t*=base | 1042 | 0.31 (0.05) | <0.001 | 0.007 (0.02) | 0.70 | 0.14 (0.08) | 0.09 | 1055 | 0.32 (0.09) | <0.001 | 0.005 (0.02) | 0.77 | 0.12 (0.12) | 0.30 | 0.47 (0.14) | 0.001 | 0.009 (0.02) | 0.64 | 0.04 (0.19) | 0.83 |
| *t*=fu | 992 | 0.24 (0.05) | <0.001 | 0.002 (0.02) | 0.90 | 0.18 (0.08) | 0.03 | 1055 | 0.47 (0.08) | <0.001 | 0.001 (0.02) | 0.95 | 0.04 (0.12) | 0.74 | 0.50 (0.14) | <0.001 | 0.006 (0.02) | 0.75 | 0.15 (0.18) | 0.40 |
| **P2S9** | | | | | | | | | | | | | | | | | | | | |
| *t*=base | 1041 | 0.16 (0.07) | 0.02 | 0.01 (0.02) | 0.47 | -0.10 (0.11) | 0.38 | 1054 | 0.50 (0.13) | <0.001 | 0.009 (0.02) | 0.58 | -0.39 (0.20) | 0.05 | 0.53 (0.22) | 0.02 | 0.01 (0.02) | 0.55 | -0.44 (0.36) | 0.23 |
| *t*=fu | 991 | 0.24 (0.07) | <0.001 | 0.01 (0.02) | 0.41 | -0.15 (0.10) | 0.16 | 1054 | 0.38 (0.11) | 0.001 | 0.01 (0.02) | 0.52 | -0.27 (0.17) | 0.11 | 0.16 (0.20) | 0.45 | 0.01 (0.02) | 0.54 | -0.37 (0.35) | 0.28 |
| **Experiment Part 2: Injunctive norms (Average P2S2 to P2S9)** | | | | | | | | | | | | | | | | | | | | |
| *t*=base | 1036 | 0.23 (0.06) | <0.001 | 0.004 (0.01) | 0.71 | 0.15 (0.08) | 0.07 | 1049 | 0.35 (0.10) | 0.001 | 0.0008 (0.01) | 0.94 | 0.16 (0.13) | 0.22 | 0.39 (0.15) | 0.009 | 0.001 (0.01) | 0.91 | 0.15 (0.19) | 0.44 |
| *t*=fu | 987 | 0.26 (0.05) | <0.001 | 0.0008 (0.01) | 0.94 | 0.02 (0.08) | 0.76 | 1049 | 0.46 (0.08) | <0.001 | -0.001 (0.01) | 0.90 | -0.04 (0.12) | 0.74 | 0.31 (0.14) | 0.03 | 0.002 (0.01) | 0.84 | 0.21 (0.18) | 0.24 |
| **P3Q1** | | | | | | | | | | | | | | | | | | | | |
| *t*=base | 1042 | 0.09 (0.06) | 0.14 | -0.04 (0.02) | 0.05 | 0.06 (0.09) | 0.49 | 1055 | 0.24 (0.11) | 0.03 | -0.04 (0.02) | 0.05 | -0.08 (0.15) | 0.59 | 0.17 (0.15) | 0.26 | -0.04 (0.02) | 0.05 | -0.03 (0.19) | 0.88 |
| *t*=fu | 992 | 0.10 (0.06) | 0.13 | -0.03 (0.02) | 0.18 | -0.12 (0.08) | 0.12 | 1055 | 0.28 (0.11) | 0.007 | -0.04 (0.02) | 0.07 | -0.09 (0.14) | 0.53 | 0.20 (0.17) | 0.26 | -0.04 (0.02) | 0.07 | -0.22 (0.24) | 0.35 |
| **P3Q2** | | | | | | | | | | | | | | | | | | | | |
| *t*=base | 1042 | 0.20 (0.05) | <0.001 | -0.05 (0.02) | 0.05 | 0.003 (0.08) | 0.97 | 1055 | 0.30 (0.08) | <0.001 | -0.05 (0.02) | 0.04 | -0.009 (0.11) | 0.94 | 0.33 (0.11) | 0.002 | -0.05 (0.02) | 0.04 | -0.07 (0.14) | 0.61 |
| *t*=fu | 992 | 0.18 (0.06) | 0.002 | -0.04 (0.02) | 0.13 | -0.05 (0.09) | 0.56 | 1055 | 0.30 (0.08) | <0.001 | -0.05 (0.02) | 0.04 | 0.01 (0.12) | 0.93 | 0.28 (0.12) | 0.02 | -0.05 (0.02) | 0.05 | -0.07 (0.17) | 0.70 |
| **Experiment Part 3: Descriptive norms (Average P3Q1 to P3Q2)** | | | | | | | | | | | | | | | | | | | | |
| *t*=base | 1042 | 0.15 (0.06) | 0.008 | -0.05 (0.02) | 0.04 | 0.001 (0.08) | 0.99 | 1055 | 0.26 (0.09) | 0.004 | -0.05 (0.02) | 0.03 | -0.08 (0.12) | 0.54 | 0.22 (0.12) | 0.06 | -0.05 (0.02) | 0.03 | -0.07 (0.15) | 0.63 |
| *t*=fu | 992 | 0.13 (0.06) | 0.03 | -0.04 (0.02) | 0.12 | -0.11 (0.08) | 0.16 | 1055 | 0.27 (0.09) | 0.003 | -0.04 (0.02) | 0.03 | -0.05 (0.13) | 0.69 | 0.18 (0.13) | 0.18 | -0.04 (0.02) | 0.04 | -0.17 (0.19) | 0.37 |
| **Donation to ASSIST/Dead Cool** | | | | | | | | | | | | | | | | | | | | |
| *t*=base | 1041 | 0.12 (0.06) | 0.03 | -0.09 (0.11) | 0.42 | 0.18 (0.08) | 0.02 | 1054 | 0.16 (0.09) | 0.09 | 0.02 (0.12) | 0.89 | -0.18 (0.12) | 0.14 | 0.39 (0.21) | 0.06 | 0.02 (0.12) | 0.86 | -0.54 (0.28) | 0.05 |
| *t*=fu | 991 | 0.35 (0.06) | <0.001 | -0.03 (0.12) | 0.80 | -0.06 (0.08) | 0.43 | 1054 | 0.49 (0.08) | <0.001 | 0.003 (0.12) | 0.98 | -0.02 (0.16) | 0.92 | 0.70 (0.14) | <0.001 | 0.03 (0.12) | 0.79 | 0.06 (0.21) | 0.78 |
| **IN1** | | | | | | | | | | | | | | | | | | | | |
| *t*=base | 1044 | -0.04 (0.07) | 0.54 | -0.008 (0.03) | 0.81 | -0.13 (0.09) | 0.14 | 1053 | 0.05 (0.13) | 0.70 | -0.008 (0.03) | 0.82 | 0.006 (0.18) | 0.97 | 0.39 (0.22) | 0.08 | -0.007 (0.03) | 0.84 | -0.05 (0.31) | 0.87 |
| *t*=fu | 1042 | 0.06 (0.06) | 0.29 | -0.01 (0.03) | 0.76 | -0.03 (0.09) | 0.73 | 1053 | -0.18 (0.12) | 0.15 | -0.01 (0.03) | 0.68 | 0.29 (0.21) | 0.16 | 0.30 (0.24) | 0.22 | -0.01 (0.03) | 0.77 | 0.70 (0.46) | 0.13 |
| **IN2** | | | | | | | | | | | | | | | | | | | | |
| *t*=base | 1046 | -0.01 (0.09) | 0.89 | -0.03 (0.02) | 0.11 | -0.03 (0.11) | 0.75 | 1055 | 0.20 (0.19) | 0.29 | -0.04 (0.02) | 0.09 | 0.27 (0.33) | 0.40 | 0.25 (0.61) | 0.68 | -0.03 (0.02) | 0.13 | 0.23 (0.60) | 0.70 |
| *t*=fu | 1044 | 0.16 (0.08) | 0.05 | -0.04 (0.02) | 0.06 | 0.12 (0.12) | 0.32 | 1055 | 0.12 (0.11) | 0.26 | -0.03 (0.02) | 0.11 | 0.14 (0.18) | 0.44 | 0.13 (0.29) | 0.66 | -0.03 (0.02) | 0.13 | -0.03 (0.36) | 0.94 |
| **IN3** | | | | | | | | | | | | | | | | | | | | |
| *t*=base | 1045 | 0.11 (0.07) | 0.09 | 0.001 (0.03) | 0.97 | 0.02 (0.09) | 0.83 | 1054 | 0.20 (0.12) | 0.10 | 0.002 (0.03) | 0.94 | 0.006 (0.16) | 0.97 | 0.26 (0.20) | 0.18 | -0.005 (0.03) | 0.86 | 0.15 (0.21) | 0.46 |
| *t*=fu | 1043 | 0.08 (0.06) | 0.15 | 0.006 (0.03) | 0.83 | 0.02 (0.07) | 0.74 | 1054 | -0.02 (0.10) | 0.88 | 0.006 (0.03) | 0.81 | -0.01 (0.17) | 0.93 | 0.14 (0.21) | 0.51 | 0.00009 (0.03) | 1.00 | 0.15 (0.25) | 0.55 |
| **IN4** | | | | | | | | | | | | | | | | | | | | |
| *t*=base | 1042 | 0.02 (0.06) | 0.72 | -0.03 (0.04) | 0.38 | -0.02 (0.09) | 0.82 | 1052 | -0.03 (0.13) | 0.82 | -0.04 (0.04) | 0.31 | 0.16 (0.21) | 0.44 | 0.70 (0.32) | 0.03 | -0.04 (0.04) | 0.28 | -0.05 (0.40) | 0.91 |
| *t*=fu | 1041 | 0.06 (0.05) | 0.31 | -0.03 (0.04) | 0.34 | -0.07 (0.08) | 0.38 | 1052 | 0.17 (0.12) | 0.15 | -0.04 (0.04) | 0.31 | -0.06 (0.17) | 0.71 | 0.42 (0.22) | 0.06 | -0.03 (0.04) | 0.37 | 0.25 (0.29) | 0.39 |
| **IN5** | | | | | | | | | | | | | | | | | | | | |
| *t*=base | 1044 | -0.02 (0.06) | 0.68 | -0.003 (0.03) | 0.94 | 0.004 (0.08) | 0.96 | 1053 | -0.06 (0.11) | 0.60 | -0.0007 (0.03) | 0.98 | 0.11 (0.16) | 0.49 | 0.02 (0.24) | 0.94 | -0.001 (0.03) | 0.98 | -0.44 (0.29) | 0.13 |
| *t*=fu | 1042 | 0.03 (0.06) | 0.64 | -0.006 (0.03) | 0.87 | 0.09 (0.09) | 0.33 | 1053 | 0.04 (0.12) | 0.75 | -0.002 (0.03) | 0.96 | 0.08 (0.17) | 0.66 | 0.14 (0.21) | 0.50 | -0.006 (0.03) | 0.86 | -0.40 (0.29) | 0.16 |
| **IN6** | | | | | | | | | | | | | | | | | | | | |
| *t*=base | 1045 | 0.21 (0.06) | 0.001 | 0.008 (0.04) | 0.83 | -0.03 (0.08) | 0.68 | 1054 | 0.19 (0.10) | 0.06 | 0.003 (0.04) | 0.93 | 0.06 (0.14) | 0.67 | 0.45 (0.17) | 0.007 | -0.0002 (0.04) | 1.00 | 0.17 (0.22) | 0.43 |
| *t*=fu | 1043 | 0.25 (0.06) | <0.001 | -0.001 (0.04) | 0.98 | -0.08 (0.09) | 0.39 | 1054 | 0.28 (0.09) | 0.002 | 0.002 (0.04) | 0.97 | -0.01 (0.14) | 0.92 | 0.43 (0.16) | 0.009 | -0.01 (0.04) | 0.81 | 0.20 (0.22) | 0.36 |
| **IN7** | | | | | | | | | | | | | | | | | | | | |
| *t*=base | 1046 | 0.10 (0.06) | 0.09 | 0.009 (0.03) | 0.79 | 0.09 (0.08) | 0.25 | 1055 | 0.20 (0.12) | 0.11 | 0.01 (0.04) | 0.75 | -0.009 (0.18) | 0.96 | 0.55 (0.19) | 0.003 | -0.002 (0.04) | 0.96 | -0.07 (0.27) | 0.78 |
| *t*=fu | 1044 | 0.19 (0.06) | 0.003 | 0.008 (0.04) | 0.83 | 0.04 (0.10) | 0.72 | 1055 | 0.22 (0.11) | 0.05 | 0.008 (0.04) | 0.82 | 0.10 (0.15) | 0.50 | 0.34 (0.18) | 0.07 | 0.002 (0.04) | 0.97 | 0.22 (0.23) | 0.33 |
| **Self-report injunctive norms (Average IN1 to IN7)** | | | | | | | | | | | | | | | | | | | | |
| *t*=base | 1040 | 0.07 (0.06) | 0.25 | -0.008 (0.02) | 0.67 | -0.05 (0.09) | 0.60 | 1050 | 0.06 (0.12) | 0.60 | -0.009 (0.02) | 0.63 | 0.09 (0.16) | 0.58 | 0.42 (0.19) | 0.03 | -0.01 (0.02) | 0.47 | 0.07 (0.23) | 0.76 |
| *t*=fu | 1039 | 0.19 (0.06) | <0.001 | -0.008 (0.02) | 0.68 | -0.08 (0.08) | 0.32 | 1050 | 0.20 (0.09) | 0.03 | -0.01 (0.02) | 0.62 | 0.06 (0.14) | 0.68 | 0.27 (0.16) | 0.09 | -0.01 (0.02) | 0.54 | 0.17 (0.20) | 0.41 |
| **DN1.1** | | | | | | | | | | | | | | | | | | | | |
| *t*=base | 1047 | 0.31 (0.11) | 0.006 | -0.02 (0.03) | 0.60 | 0.23 (0.15) | 0.12 | 1056 | 0.28 (0.14) | 0.05 | -0.02 (0.03) | 0.47 | 0.34 (0.21) | 0.11 | 0.95 (0.29) | 0.001 | -0.004 (0.03) | 0.87 | 0.44 (0.29) | 0.13 |
| *t*=fu | 1045 | 0.31 (0.08) | <0.001 | -0.01 (0.03) | 0.61 | 0.13 (0.12) | 0.30 | 1056 | 0.21 (0.13) | 0.12 | -0.02 (0.03) | 0.53 | 0.30 (0.20) | 0.13 | 0.55 (0.18) | 0.002 | -0.004 (0.03) | 0.87 | 0.37 (0.22) | 0.10 |
| **DN1.2** | | | | | | | | | | | | | | | | | | | | |
| *t*=base | 1047 | 0.12 (0.05) | 0.01 | -0.06 (0.03) | 0.05 | 0.11 (0.07) | 0.11 | 1056 | 0.04 (0.07) | 0.57 | -0.07 (0.03) | 0.02 | 0.24 (0.10) | 0.02 | 0.12 (0.09) | 0.16 | -0.06 (0.03) | 0.04 | 0.22 (0.10) | 0.04 |
| *t*=fu | 1045 | -0.01 (0.05) | 0.80 | -0.07 (0.03) | 0.03 | 0.09 (0.07) | 0.19 | 1056 | -0.01 (0.08) | 0.86 | -0.07 (0.03) | 0.02 | 0.25 (0.11) | 0.02 | 0.06 (0.09) | 0.53 | -0.06 (0.03) | 0.04 | 0.25 (0.11) | 0.03 |
| **DN1.3** | | | | | | | | | | | | | | | | | | | | |
| *t*=base | 1047 | 0.09 (0.04) | 0.06 | -0.10 (0.03) | 0.003 | 0.07 (0.06) | 0.26 | 1056 | -0.006 (0.08) | 0.95 | -0.10 (0.03) | 0.002 | 0.21 (0.10) | 0.04 | 0.06 (0.13) | 0.66 | -0.10 (0.03) | 0.002 | 0.28 (0.13) | 0.03 |
| *t*=fu | 1045 | 0.0003 (0.05) | 1.00 | -0.10 (0.03) | 0.002 | 0.02 (0.06) | 0.68 | 1056 | -0.03 (0.08) | 0.71 | -0.10 (0.03) | 0.002 | 0.19 (0.10) | 0.04 | **-0.04 (0.14)** | **0.80** | **-0.10 (0.03)** | **0.003** | **0.41 (0.16)** | **0.009** |
| **DN1.4** | | | | | | | | | | | | | | | | | | | | |
| *t*=base | 1046 | 0.01 (0.05) | 0.74 | -0.03 (0.03) | 0.23 | -0.06 (0.06) | 0.34 | 1055 | 0.09 (0.14) | 0.53 | -0.04 (0.03) | 0.21 | 0.08 (0.17) | 0.64 | 0.14 (0.14) | 0.33 | -0.03 (0.03) | 0.25 | -0.08 (0.20) | 0.70 |
| *t*=fu | 1044 | -0.08 (0.04) | 0.04 | -0.03 (0.03) | 0.24 | -0.01 (0.07) | 0.84 | 1055 | 0.11 (0.16) | 0.50 | -0.04 (0.03) | 0.20 | 0.14 (0.17) | 0.41 | -0.08 (0.17) | 0.66 | -0.03 (0.03) | 0.29 | -0.15 (0.20) | 0.46 |
| **DN1.5** | | | | | | | | | | | | | | | | | | | | |
| *t*=base | 1046 | 0.06 (0.05) | 0.21 | -0.005 (0.01) | 0.72 | 0.003 (0.05) | 0.95 | 1055 | 0.16 (0.12) | 0.19 | -0.006 (0.01) | 0.71 | 0.06 (0.12) | 0.62 | 0.24 (0.18) | 0.18 | -0.003 (0.01) | 0.82 | -0.14 (0.21) | 0.50 |
| *t*=fu | 1044 | -0.01 (0.05) | 0.83 | -0.007 (0.01) | 0.64 | 0.08 (0.05) | 0.13 | 1055 | 0.18 (0.11) | 0.11 | -0.008 (0.02) | 0.62 | 0.12 (0.12) | 0.35 | 0.10 (0.16) | 0.53 | -0.003 (0.01) | 0.83 | -0.11 (0.16) | 0.47 |
| **Self-report descriptive norms 1 (Average DN1.1 to DN1.5)** | | | | | | | | | | | | | | | | | | | | |
| *t*=base | 1046 | 0.10 (0.05) | 0.05 | -0.04 (0.02) | 0.01 | 0.11 (0.07) | 0.13 | **1055** | **0.02 (0.07)** | **0.78** | **-0.04 (0.02)** | **0.006** | **0.26 (0.09)** | **0.006** | 0.15 (0.10) | 0.15 | -0.04 (0.01) | 0.02 | 0.19 (0.09) | 0.04 |
| *t*=fu | 1044 | 0.02 (0.05) | 0.66 | -0.04 (0.02) | 0.007 | 0.15 (0.07) | 0.02 | **1055** | **-0.02 (0.08)** | **0.80** | **-0.04 (0.02)** | **0.006** | **0.29 (0.09)** | **0.002** | 0.10 (0.10) | 0.30 | -0.03 (0.01) | 0.02 | 0.19 (0.09) | 0.04 |
| **DN2.1** | | | | | | | | | | | | | | | | | | | | |
| *t*=base | 1047 | 0.19 (0.07) | 0.01 | -0.06 (0.03) | 0.02 | 0.24 (0.10) | 0.02 | 1056 | 0.19 (0.10) | 0.08 | -0.06 (0.03) | 0.02 | 0.26 (0.15) | 0.07 | 0.21 (0.15) | 0.15 | -0.05 (0.03) | 0.04 | 0.31 (0.18) | 0.08 |
| *t*=fu | 1045 | 0.19 (0.07) | 0.006 | -0.05 (0.03) | 0.04 | 0.01 (0.08) | 0.89 | 1056 | 0.21 (0.12) | 0.07 | -0.05 (0.03) | 0.04 | 0.04 (0.15) | 0.81 | 0.09 (0.16) | 0.58 | -0.05 (0.03) | 0.03 | 0.35 (0.17) | 0.04 |
| **DN2.2** | | | | | | | | | | | | | | | | | | | | |
| *t*=base | 1047 | 0.18 (0.06) | 0.004 | -0.08 (0.04) | 0.03 | 0.22 (0.09) | 0.02 | 1056 | 0.27 (0.10) | 0.005 | -0.06 (0.04) | 0.07 | 0.25 (0.13) | 0.05 | 0.46 (0.17) | 0.006 | -0.06 (0.04) | 0.11 | 0.33 (0.18) | 0.06 |
| *t*=fu | 1045 | 0.11 (0.06) | 0.07 | -0.08 (0.03) | 0.03 | 0.02 (0.08) | 0.82 | 1056 | 0.16 (0.09) | 0.08 | -0.07 (0.04) | 0.05 | 0.19 (0.12) | 0.12 | 0.36 (0.16) | 0.02 | -0.06 (0.04) | 0.12 | 0.30 (0.16) | 0.06 |
| **DN2.3** | | | | | | | | | | | | | | | | | | | | |
| *t*=base | 1047 | 0.28 (0.08) | <0.001 | -0.003 (0.03) | 0.92 | -0.01 (0.12) | 0.90 | 1056 | 0.60 (0.10) | <0.001 | 0.01 (0.03) | 0.73 | 0.04 (0.12) | 0.72 | 0.67 (0.13) | <0.001 | 0.007 (0.03) | 0.81 | 0.14 (0.14) | 0.33 |
| *t*=fu | 1045 | 0.19 (0.06) | 0.001 | -0.004 (0.03) | 0.90 | -0.002 (0.07) | 0.97 | 1056 | 0.41 (0.09) | <0.001 | 0.003 (0.03) | 0.90 | -0.02 (0.11) | 0.89 | 0.55 (0.12) | <0.001 | 0.006 (0.03) | 0.84 | 0.06 (0.13) | 0.63 |
| **Self-report descriptive norms 2 (Average DN2.1 to DN2.3)** | | | | | | | | | | | | | | | | | | | | |
| *t*=base | 1047 | 0.23 (0.07) | <0.001 | -0.04 (0.02) | 0.04 | 0.20 (0.09) | 0.02 | 1056 | 0.36 (0.10) | <0.001 | -0.03 (0.02) | 0.09 | 0.27 (0.12) | 0.02 | 0.44 (0.14) | 0.001 | -0.03 (0.02) | 0.11 | 0.24 (0.14) | 0.08 |
| *t*=fu | 1045 | 0.11 (0.06) | 0.06 | -0.04 (0.02) | 0.05 | 0.07 (0.07) | 0.28 | 1056 | 0.26 (0.09) | 0.002 | -0.03 (0.02) | 0.08 | 0.18 (0.10) | 0.06 | 0.35 (0.13) | 0.006 | -0.03 (0.02) | 0.11 | 0.24 (0.12) | 0.05 |
| **Self-report smoking behavior** | | | | | | | | | | | | | | | | | | | | |
| *t*=base | 1050 | 0.15 (0.08) | 0.05 | -0.05 (0.02) | 0.04 | 0.03 (0.11) | 0.76 | 1059 | 0.26 (0.11) | 0.02 | -0.05 (0.02) | 0.04 | 0.01 (0.14) | 0.92 | 0.59 (0.17) | <0.001 | -0.06 (0.02) | 0.01 | 0.18 (0.18) | 0.31 |
| *t*=fu | **1048** | **0.27 (0.06)** | **<0.001** | **-0.06 (0.02)** | **0.009** | **0.28 (0.09)** | **0.003** | 1059 | 0.23 (0.08) | 0.007 | -0.05 (0.02) | 0.02 | 0.20 (0.10) | 0.04 | 0.43 (0.10) | <0.001 | -0.06 (0.02) | 0.01 | 0.27 (0.12) | 0.02 |
| **Intentions** | | | | | | | | | | | | | | | | | | | | |
| *t*=base | 1047 | 0.17 (0.09) | 0.05 | -0.03 (0.05) | 0.54 | 0.008 (0.11) | 0.94 | 1056 | 0.48 (0.14) | 0.001 | -0.05 (0.05) | 0.32 | 0.38 (0.23) | 0.10 | 0.94 (0.26) | <0.001 | -0.05 (0.05) | 0.27 | 0.35 (0.39) | 0.37 |
| *t*=fu | 1045 | 0.22 (0.08) | 0.005 | -0.05 (0.05) | 0.35 | 0.20 (0.12) | 0.09 | 1056 | 0.11 (0.12) | 0.36 | -0.05 (0.05) | 0.36 | 0.38 (0.19) | 0.05 | 0.36 (0.15) | 0.02 | -0.05 (0.05) | 0.33 | 0.43 (0.26) | 0.10 |
| **Knowledge** | | | | | | | | | | | | | | | | | | | | |
| *t*=base | 1049 | 0.19 (0.05) | 0.001 | 0.08 (0.06) | 0.18 | 0.04 (0.07) | 0.62 | 1058 | 0.40 (0.08) | <0.001 | 0.07 (0.06) | 0.26 | -0.04 (0.10) | 0.68 | 0.42 (0.11) | <0.001 | 0.06 (0.06) | 0.33 | -0.10 (0.13) | 0.42 |
| *t*=fu | 1047 | 0.28 (0.06) | <0.001 | 0.10 (0.06) | 0.13 | -0.02 (0.08) | 0.83 | 1058 | 0.41 (0.08) | <0.001 | 0.06 (0.06) | 0.33 | -0.005 (0.09) | 0.95 | 0.38 (0.11) | 0.001 | 0.07 (0.06) | 0.31 | -0.10 (0.13) | 0.46 |
| **Attitudes** | | | | | | | | | | | | | | | | | | | | |
| *t*=base | 1037 | 0.16 (0.06) | 0.007 | 0.02 (0.03) | 0.35 | -0.007 (0.08) | 0.93 | 1046 | -0.03 (0.11) | 0.75 | 0.02 (0.03) | 0.49 | 0.05 (0.15) | 0.72 | 0.33 (0.22) | 0.13 | 0.02 (0.03) | 0.50 | 0.18 (0.25) | 0.49 |
| *t*=fu | 1035 | 0.17 (0.07) | 0.01 | 0.02 (0.03) | 0.48 | 0.06 (0.09) | 0.51 | 1046 | -0.11 (0.12) | 0.37 | 0.02 (0.03) | 0.48 | 0.26 (0.15) | 0.07 | 0.15 (0.17) | 0.39 | 0.02 (0.03) | 0.52 | 0.47 (0.20) | 0.02 |
| **Self-efficacy (Emotional)** | | | | | | | | | | | | | | | | | | | | |
| *t*=base | 1040 | 0.10 (0.08) | 0.20 | 0.006 (0.04) | 0.89 | -0.11 (0.13) | 0.37 | 1049 | 0.27 (0.12) | 0.03 | -0.004 (0.04) | 0.93 | 0.03 (0.23) | 0.90 | 0.40 (0.17) | 0.02 | -0.007 (0.04) | 0.87 | 0.27 (0.28) | 0.33 |
| *t*=fu | **1037** | **0.30 (0.07)** | **<0.001** | **-0.02 (0.04)** | **0.59** | **0.27 (0.11)** | **0.01** | 1049 | 0.20 (0.09) | 0.03 | -0.007 (0.04) | 0.87 | 0.05 (0.17) | 0.76 | 0.37 (0.14) | 0.006 | -0.01 (0.04) | 0.72 | 0.07 (0.20) | 0.75 |
| **Self-efficacy (Friends)** | | | | | | | | | | | | | | | | | | | | |
| *t*=base | 1045 | 0.05 (0.06) | 0.41 | -0.009 (0.04) | 0.81 | -0.10 (0.11) | 0.36 | 1054 | 0.19 (0.11) | 0.09 | -0.01 (0.04) | 0.70 | 0.001 (0.18) | 0.99 | 0.15 (0.17) | 0.40 | -0.01 (0.04) | 0.74 | 0.11 (0.22) | 0.60 |
| *t*=fu | 1043 | 0.23 (0.07) | 0.001 | -0.03 (0.04) | 0.44 | 0.22 (0.09) | 0.02 | 1054 | 0.10 (0.10) | 0.31 | -0.01 (0.04) | 0.71 | 0.02 (0.18) | 0.92 | 0.25 (0.19) | 0.18 | -0.02 (0.04) | 0.63 | 0.08 (0.28) | 0.78 |
| **Self-efficacy (Opportunity)** | | | | | | | | | | | | | | | | | | | | |
| *t*=base | 1046 | 0.13 (0.07) | 0.09 | -0.02 (0.03) | 0.57 | -0.12 (0.14) | 0.42 | 1055 | 0.39 (0.15) | 0.008 | -0.03 (0.03) | 0.45 | -0.007 (0.24) | 0.98 | 0.37 (0.21) | 0.08 | -0.03 (0.03) | 0.37 | 0.26 (0.25) | 0.30 |
| *t*=fu | 1044 | 0.21 (0.08) | 0.006 | -0.04 (0.03) | 0.22 | 0.21 (0.10) | 0.04 | 1055 | 0.21 (0.10) | 0.04 | -0.03 (0.03) | 0.42 | 0.008 (0.19) | 0.97 | 0.26 (0.18) | 0.14 | -0.03 (0.03) | 0.35 | 0.21 (0.23) | 0.37 |
| **Perceived physical risks** | | | | | | | | | | | | | | | | | | | | |
| *t*=base | 1041 | 0.05 (0.05) | 0.31 | 3.17 (0.87) | <0.001 | -0.09 (0.09) | 0.28 | 1049 | 0.16 (0.09) | 0.08 | 3.11 (0.87) | <0.001 | -0.23 (0.12) | 0.05 | 0.06 (0.14) | 0.70 | 3.15 (0.87) | <0.001 | -0.30 (0.18) | 0.10 |
| *t*=fu | 1038 | 0.10 (0.05) | 0.06 | 3.07 (0.87) | <0.001 | -0.11 (0.07) | 0.13 | 1049 | 0.08 (0.11) | 0.50 | 3.16 (0.87) | <0.001 | -0.18 (0.13) | 0.18 | -0.25 (0.21) | 0.24 | 3.37 (0.90) | <0.001 | -0.25 (0.19) | 0.18 |
| **Perceived social risks** | | | | | | | | | | | | | | | | | | | | |
| *t*=base | 1043 | 0.13 (0.05) | 0.02 | 3.37 (0.98) | 0.001 | -0.04 (0.08) | 0.62 | 1052 | 0.22 (0.07) | 0.003 | 3.24 (1.01) | 0.001 | -0.07 (0.09) | 0.48 | 0.31 (0.10) | 0.001 | 2.88 (1.03) | 0.005 | -0.006 (0.12) | 0.96 |
| *t*=fu | 1041 | 0.10 (0.06) | 0.08 | 3.37 (0.98) | 0.001 | -0.05 (0.08) | 0.53 | 1052 | 0.18 (0.09) | 0.04 | 3.50 (1.02) | 0.001 | -0.15 (0.11) | 0.17 | 0.28 (0.12) | 0.02 | 3.04 (1.07) | 0.004 | -0.04 (0.14) | 0.79 |
| **Perceived addiction risks** | | | | | | | | | | | | | | | | | | | | |
| *t*=base | 985 | 0.21 (0.06) | <0.001 | 1.91 (1.16) | 0.10 | 0.03 (0.07) | 0.64 | 994 | 0.49 (0.09) | <0.001 | 1.14 (1.15) | 0.32 | 0.07 (0.11) | 0.56 | 0.69 (0.11) | <0.001 | 0.85 (1.15) | 0.46 | 0.08 (0.13) | 0.55 |
| *t*=fu | 979 | 0.14 (0.05) | 0.01 | 1.66 (1.20) | 0.16 | 0.05 (0.07) | 0.48 | 994 | 0.38 (0.08) | <0.001 | 1.33 (1.17) | 0.26 | 0.05 (0.10) | 0.60 | 0.55 (0.09) | <0.001 | 1.08 (1.15) | 0.35 | 0.05 (0.12) | 0.69 |
| **Perceived benefits** | | | | | | | | | | | | | | | | | | | | |
| *t*=base | 1004 | -0.03 (0.06) | 0.60 | 1.51 (0.90) | 0.09 | 0.04 (0.09) | 0.66 | 1013 | 0.09 (0.12) | 0.46 | 1.59 (0.89) | 0.07 | 0.15 (0.15) | 0.32 | 0.32 (0.22) | 0.14 | 1.76 (0.91) | 0.05 | 0.24 (0.29) | 0.41 |
| *t*=fu | 1001 | 0.02 (0.06) | 0.76 | 1.41 (0.90) | 0.12 | -0.05 (0.09) | 0.58 | 1013 | -0.08 (0.13) | 0.54 | 1.61 (0.88) | 0.07 | -0.18 (0.18) | 0.31 | -0.07 (0.25) | 0.80 | 1.60 (0.89) | 0.07 | 0.39 (0.41) | 0.34 |
| **Perceived behavioral control (easy to quit)** | | | | | | | | | | | | | | | | | | | | |
| *t*=base | **1044** | **0.26 (0.06)** | **<0.001** | **-0.05 (0.06)** | **0.38** | **0.18 (0.07)** | **0.009** | 1053 | 0.48 (0.08) | <0.001 | -0.04 (0.06) | 0.50 | 0.12 (0.10) | 0.25 | 0.67 (0.10) | <0.001 | -0.03 (0.06) | 0.63 | 0.07 (0.12) | 0.58 |
| *t*=fu | 1042 | 0.16 (0.06) | 0.007 | -0.09 (0.06) | 0.15 | 0.12 (0.07) | 0.08 | 1053 | 0.48 (0.08) | <0.001 | -0.05 (0.06) | 0.46 | 0.05 (0.10) | 0.60 | 0.60 (0.10) | <0.001 | -0.03 (0.06) | 0.63 | 0.11 (0.12) | 0.36 |
| **Perceived behavioral control (to avoid smoking)** | | | | | | | | | | | | | | | | | | | | |
| *t*=base | 1047 | 0.05 (0.07) | 0.45 | -0.02 (0.05) | 0.62 | 0.19 (0.10) | 0.05 | 1056 | 0.36 (0.14) | 0.009 | -0.05 (0.05) | 0.38 | 0.18 (0.19) | 0.35 | 0.80 (0.23) | <0.001 | -0.07 (0.05) | 0.21 | 0.38 (0.32) | 0.23 |
| *t*=fu | 1045 | 0.08 (0.07) | 0.23 | -0.04 (0.05) | 0.47 | 0.03 (0.10) | 0.77 | 1056 | 0.11 (0.14) | 0.43 | -0.04 (0.05) | 0.43 | 0.15 (0.18) | 0.43 | 0.55 (0.19) | 0.003 | -0.07 (0.05) | 0.18 | 0.37 (0.25) | 0.14 |
| **Objectively measured smoking behavior** | | | | | | | | | | | | | | | | | | | | |
| *t*=base | 1010 | 0.35 (0.06) | <0.001 | -0.05 (0.07) | 0.45 | -0.003 (0.05) | 0.95 | 1017 | 0.36 (0.05) | <0.001 | -0.06 (0.07) | 0.34 | -0.03 (0.05) | 0.55 | 0.57 (0.06) | <0.001 | 0.0007 (0.07) | 0.99 | -0.03 (0.06) | 0.63 |
| *t*=fu | 992 | 0.47 (0.08) | <0.001 | -0.09 (0.06) | 0.15 | -0.12 (0.08) | 0.12 | 1017 | 0.83 (0.06) | <0.001 | -0.006 (0.05) | 0.92 | -0.06 (0.06) | 0.33 | 0.85 (0.06) | <0.001 | 0.02 (0.06) | 0.72 | -0.13 (0.08) | 0.09 |
| **Smoking susceptibility^e^** | | | | | | | | | | | | | | | | | | | | |
|  | n | **Percent_-i,t_^f^** | | **M^g^** | | **M*Percent_-i,t_^h^** | | n | **Percent_-i,t_^f^** | | **M^g^** | | **M*Percent_-i,t_^h^** | | **Percent_-i,t_^f^** | | **M^g^** | | **M*Percent_-i,t_^h^** | |
|  |  | OR (SE) | p-value | OR (SE) | p-value | OR (SE) | p-value |  | OR (SE) | p-value | OR (SE) | p-value | OR (SE) | p-value | OR (SE) | p-value | OR (SE) | p-value | OR (SE) | p-value |
| *t*=base | 1047 | 1.13 (0.04) | <0.001 | 1.00 (0.11) | 1.00 | 0.91 (0.04) | 0.03 | 1056 | 1.07 (0.06) | 0.26 | 1.01 (0.11) | 0.91 | 1.06 (0.09) | 0.49 | 1.20 (0.12) | 0.06 | 1.05 (0.12) | 0.68 | 1.30 (0.18) | 0.05 |
| *t*=fu | 1045 | 1.13 (0.03) | <0.001 | 1.00 (0.11) | 0.98 | 0.93 (0.04) | 0.12 | 1056 | 1.15 (0.06) | 0.01 | 1.03 (0.12) | 0.76 | 1.08 (0.08) | 0.29 | 1.26 (0.10) | 0.002 | 1.07 (0.12) | 0.54 | 1.14 (0.12) | 0.21 |

IV: Independent variable; DV: Dependent variable; Ave: average of peer group (*i*); M: moderator (Fear of negative evaluation: 1 [least fear of negative evaluation] to 5 [most fear of negative evaluation]); Percent: percentage of peer group (*-i*) classified as susceptible to commencing smoking; OR: odds ratio; SE: standard error.

^a^In each model the outcome variable is the focal participant’s (*i*) response to the relevant item at follow-up. The predictor variable is the average of the relevant group’s (*-i*) responses to the equivalent item at baseline (*t*=base) or follow-up (*t*=fu), where *–i*=(1) focal participant's nominated friends; (2) focal participant’s school class; (3) focal participant’s school year group. The moderator, and interaction of the moderator with the predictor variable, were also included as independent variables in all models. All models include robust (Huber White) standard errors specified using Stata’s ‘vce(robust)’ option. The following baseline variables are included as covariates in all models: gender (0=boy; 1=girl/prefer not to say), age (1=12 years or less; 2=13 years; 3=14 years or more), intervention (1=ASSIST; 2=Dead Cool), ethnicity (0=no ethnic minority; 1=ethnic minority), individuals' socio-economic status (NI: 1=NIMDM2017≤296.6; 2=296.6<NIMDM2017≤593.2; 3=NIMDM2017>593.2; Bogotá: 1=Informal settlement/Lowest/Low; 2=Middle-Low/Middle; 3=Middle-High/High), and baseline values of the outcome variable. The predictor variable, moderator variable, and baseline values of the outcome variable were mean-centered.

^b^Unstandardized regression coefficients representing the average change in the outcome variable for a one-unit increase in the predictor variable among participants who are average on fear of negative evaluation.

^c^Unstandardized regression coefficients representing the average change in the outcome variable for a one-unit increase in fear of negative evaluation among participants who are average on the predictor variable.

^d^Unstandardized regression coefficients representing the average change in the association between the outcome variable and the predictor variable for a one-unit increase in fear of negative evaluation.

^e^Logistic regressions were run for models including focal participants' smoking susceptibility as the outcome variable, with robust (Huber White) standard errors specified using Stata’s ‘vce(robust)’ option. The predictor variable is the percentage of the relevant group (*-i*) classified as susceptible to commencing smoking at baseline (*t*=base) or follow-up (*t*=fu), where *–i*=(1) focal participant's nominated friends; (2) focal participant’s school class; (3) focal participant’s school year group. The moderator, and interaction of the moderator with the predictor variable, were also included as independent variables in all models. The following baseline variables are included as covariates in all models: gender (0=boy; 1=girl/prefer not to say), age (1=12 years or less; 2=13 years; 3=14 years or more), intervention (1=ASSIST; 2=Dead Cool), ethnicity (0=no ethnic minority; 1=ethnic minority), individuals' socio-economic status (NI: 1=NIMDM2017≤296.6; 2=296.6<NIMDM2017≤593.2; 3=NIMDM2017>593.2; Bogotá: 1=Informal settlement/Lowest/Low; 2=Middle-Low/Middle; 3=Middle-High/High), and baseline values of the outcome variable. Results are odds ratios, standard errors, and p-values. The predictor variable, and moderator variable were mean-centered.

^f^Odds ratios representing the multiplicative change in odds of being susceptible to commencing smoking for a 10% increase in the number of nominated friends/pupils in the same school class/pupils in the same school year group classified as being susceptible to commencing smoking (1 out of 10 nominated friends/pupils in the same school class/pupils in the same school year group; predictor variable) among participants who are average on fear of negative evaluation.

^g^Odds ratios representing the multiplicative change in odds of being susceptible to commencing smoking for a one-unit increase in fear of negative evaluation among participants who are average on the predictor variable.

^h^Ratio of ratios representing the ratio of: (1) the odds ratio representing the multiplicative change in odds of being susceptible to commencing smoking for a 10% increase in the number of nominated friends/pupils in the same school class/pupils in the same school year group classified as being susceptible to commencing smoking among participants who are one unit above average on fear of negative evaluation; to (2) the odds ratio representing the multiplicative change in odds of being susceptible to commencing smoking for a 10% increase in the number of nominated friends/pupils in the same school class/pupils in the same school year group classified as being susceptible to commencing smoking among participants who are average on fear of negative evaluation.

**Table S2.10.** Results of ordinary least squares linear regressions including interaction terms examining differences in peer influence effects according to need to belong for outcomes collected at follow-up.

|  | **Dependent variable: Participant responses to the outcome variable at follow-up^a^** | | | | | | | | | | | | | | | | | | | |
| --- | --- | --- | --- | --- | --- | --- | --- | --- | --- | --- | --- | --- | --- | --- | --- | --- | --- | --- | --- | --- |
|  | **(1) -i=Average of nominated friends** | | | | | | | n | **(2) -i=Average of school class** | | | | | | **(3) -i=Average of school year group** | | | | | |
| **IV** | n | **Ave_-i,t_^b^** | | **M^c^** | | **M*Ave_-i,t_^d^** | |  | **Ave_-i,t_^b^** | | **M^c^** | | **M*Ave_-i,t_^d^** | | **Ave_-i,t_^b^** | | **M^c^** | | **M*Ave_-i,t_^d^** | |
| **DV** |  | *b* (SE) | p-value | *b* (SE) | p-value | *b* (SE) | p-value |  | *b* (SE) | p-value | *b* (SE) | p-value | *b* (SE) | p-value | *b* (SE) | p-value | *b* (SE) | p-value | *b* (SE) | p-value |
| **P2S2** | | | | | | | | | | | | | | | | | | | | |
| *t*=base | 1051 | 0.34 (0.09) | <0.001 | -0.006 (0.01) | 0.68 | -0.005 (0.12) | 0.97 | 1064 | 0.46 (0.12) | <0.001 | -0.009 (0.01) | 0.53 | 0.0002 (0.20) | 1.00 | 0.83 (0.19) | <0.001 | -0.01 (0.02) | 0.39 | 0.14 (0.29) | 0.62 |
| *t*=fu | 995 | 0.17 (0.07) | 0.01 | -0.01 (0.01) | 0.42 | -0.12 (0.11) | 0.25 | 1064 | 0.33 (0.10) | 0.002 | -0.01 (0.01) | 0.42 | -0.25 (0.16) | 0.11 | 0.61 (0.17) | <0.001 | -0.01 (0.02) | 0.50 | 0.10 (0.30) | 0.73 |
| **P2S3** | | | | | | | | | | | | | | | | | | | | |
| *t*=base | 1050 | 0.11 (0.07) | 0.11 | -0.02 (0.02) | 0.19 | 0.02 (0.10) | 0.84 | 1063 | 0.20 (0.16) | 0.20 | -0.02 (0.02) | 0.19 | -0.18 (0.25) | 0.48 | 0.09 (0.28) | 0.74 | -0.02 (0.02) | 0.27 | 0.52 (0.40) | 0.20 |
| *t*=fu | 994 | 0.11 (0.07) | 0.10 | -0.02 (0.02) | 0.19 | -0.05 (0.10) | 0.64 | 1063 | 0.22 (0.14) | 0.11 | -0.02 (0.02) | 0.19 | -0.26 (0.21) | 0.21 | 0.23 (0.26) | 0.39 | -0.02 (0.02) | 0.22 | 0.10 (0.41) | 0.80 |
| **P2S4** | | | | | | | | | | | | | | | | | | | | |
| *t*=base | 1049 | 0.12 (0.08) | 0.11 | -0.03 (0.02) | 0.09 | 0.14 (0.10) | 0.17 | 1062 | 0.15 (0.12) | 0.24 | -0.03 (0.02) | 0.06 | 0.25 (0.19) | 0.19 | 0.42 (0.33) | 0.20 | -0.03 (0.02) | 0.07 | 0.25 (0.35) | 0.48 |
| *t*=fu | 994 | 0.11 (0.07) | 0.12 | -0.04 (0.02) | 0.02 | -0.14 (0.09) | 0.13 | 1062 | 0.27 (0.12) | 0.02 | -0.03 (0.02) | 0.09 | -0.10 (0.16) | 0.50 | 0.39 (0.20) | 0.05 | -0.03 (0.01) | 0.10 | -0.18 (0.23) | 0.43 |
| **P2S5** | | | | | | | | | | | | | | | | | | | | |
| *t*=base | 1051 | 0.20 (0.06) | 0.001 | -0.04 (0.02) | 0.05 | 0.05 (0.11) | 0.65 | 1064 | 0.36 (0.10) | <0.001 | -0.05 (0.02) | 0.02 | 0.19 (0.17) | 0.25 | 0.53 (0.15) | <0.001 | -0.05 (0.02) | 0.02 | 0.09 (0.24) | 0.70 |
| *t*=fu | 995 | 0.18 (0.06) | 0.001 | -0.05 (0.02) | 0.01 | 0.06 (0.09) | 0.54 | 1064 | 0.48 (0.09) | <0.001 | -0.05 (0.02) | 0.02 | 0.09 (0.13) | 0.48 | 0.51 (0.14) | <0.001 | -0.05 (0.02) | 0.02 | 0.22 (0.20) | 0.26 |
| **P2S6** | | | | | | | | | | | | | | | | | | | | |
| *t*=base | 1049 | 0.16 (0.07) | 0.02 | -0.04 (0.02) | 0.07 | 0.03 (0.11) | 0.78 | 1062 | 0.37 (0.11) | 0.001 | -0.04 (0.02) | 0.09 | -0.01 (0.17) | 0.94 | 0.37 (0.19) | 0.05 | -0.03 (0.02) | 0.11 | 0.18 (0.25) | 0.47 |
| *t*=fu | 993 | 0.14 (0.06) | 0.01 | -0.04 (0.02) | 0.09 | 0.13 (0.10) | 0.19 | 1062 | 0.43 (0.10) | <0.001 | -0.04 (0.02) | 0.10 | 0.05 (0.17) | 0.78 | 0.47 (0.15) | 0.002 | -0.03 (0.02) | 0.11 | 0.17 (0.22) | 0.44 |
| **P2S7** | | | | | | | | | | | | | | | | | | | | |
| *t*=base | 1050 | 0.17 (0.05) | 0.002 | -0.007 (0.02) | 0.71 | -0.04 (0.09) | 0.64 | 1063 | 0.29 (0.09) | 0.001 | -0.006 (0.02) | 0.77 | -0.05 (0.13) | 0.72 | 0.28 (0.14) | 0.05 | -0.005 (0.02) | 0.80 | 0.17 (0.20) | 0.41 |
| *t*=fu | 995 | 0.26 (0.06) | <0.001 | -0.01 (0.02) | 0.62 | -0.04 (0.09) | 0.68 | 1063 | 0.39 (0.09) | <0.001 | -0.007 (0.02) | 0.73 | -0.02 (0.14) | 0.91 | 0.31 (0.17) | 0.07 | -0.003 (0.02) | 0.89 | 0.31 (0.22) | 0.16 |
| **P2S8** | | | | | | | | | | | | | | | | | | | | |
| *t*=base | 1051 | 0.30 (0.05) | <0.001 | -0.03 (0.02) | 0.17 | 0.11 (0.08) | 0.18 | 1064 | 0.30 (0.09) | 0.001 | -0.03 (0.02) | 0.18 | 0.02 (0.13) | 0.85 | 0.44 (0.14) | 0.002 | -0.02 (0.02) | 0.23 | -0.13 (0.21) | 0.54 |
| *t*=fu | 995 | 0.22 (0.06) | <0.001 | -0.03 (0.02) | 0.08 | 0.03 (0.08) | 0.69 | 1064 | 0.46 (0.08) | <0.001 | -0.03 (0.02) | 0.17 | -0.05 (0.12) | 0.67 | 0.48 (0.14) | <0.001 | -0.03 (0.02) | 0.16 | -0.05 (0.19) | 0.78 |
| **P2S9** | | | | | | | | | | | | | | | | | | | | |
| *t*=base | 1051 | 0.17 (0.07) | 0.01 | -0.02 (0.02) | 0.18 | -0.13 (0.12) | 0.26 | **1064** | **0.52 (0.12)** | **<0.001** | **-0.02 (0.02)** | **0.17** | **-0.54 (0.21)** | **0.01** | 0.54 (0.21) | 0.01 | -0.03 (0.02) | 0.16 | -0.16 (0.39) | 0.68 |
| *t*=fu | 995 | 0.23 (0.06) | <0.001 | -0.02 (0.02) | 0.17 | -0.08 (0.11) | 0.49 | 1064 | 0.38 (0.11) | <0.001 | -0.02 (0.02) | 0.20 | -0.10 (0.18) | 0.57 | 0.19 (0.20) | 0.36 | -0.02 (0.02) | 0.19 | -0.09 (0.35) | 0.79 |
| **Experiment Part 2: Injunctive norms (Average P2S2 to P2S9)** | | | | | | | | | | | | | | | | | | | | |
| *t*=base | 1045 | 0.23 (0.06) | <0.001 | -0.02 (0.01) | 0.09 | 0.05 (0.10) | 0.64 | 1058 | 0.37 (0.10) | <0.001 | -0.02 (0.01) | 0.07 | 0.002 (0.16) | 0.99 | 0.43 (0.15) | 0.004 | -0.02 (0.01) | 0.09 | 0.13 (0.24) | 0.59 |
| *t*=fu | 991 | 0.25 (0.05) | <0.001 | -0.02 (0.01) | 0.05 | -0.04 (0.09) | 0.68 | 1058 | 0.46 (0.08) | <0.001 | -0.02 (0.01) | 0.07 | -0.08 (0.12) | 0.54 | 0.34 (0.14) | 0.02 | -0.02 (0.01) | 0.10 | 0.10 (0.21) | 0.63 |
| **P3Q1** | | | | | | | | | | | | | | | | | | | | |
| *t*=base | 1051 | 0.09 (0.06) | 0.17 | -0.04 (0.02) | 0.15 | 0.14 (0.10) | 0.16 | 1064 | 0.23 (0.11) | 0.04 | -0.04 (0.03) | 0.14 | -0.08 (0.17) | 0.66 | 0.14 (0.15) | 0.37 | -0.03 (0.03) | 0.21 | 0.33 (0.22) | 0.14 |
| *t*=fu | 995 | 0.11 (0.06) | 0.10 | -0.03 (0.03) | 0.23 | -0.17 (0.09) | 0.05 | 1064 | 0.28 (0.10) | 0.008 | -0.03 (0.03) | 0.19 | -0.16 (0.15) | 0.29 | 0.17 (0.17) | 0.33 | -0.03 (0.03) | 0.19 | 0.07 (0.25) | 0.79 |
| **P3Q2** | | | | | | | | | | | | | | | | | | | | |
| *t*=base | 1051 | 0.19 (0.05) | <0.001 | -0.03 (0.03) | 0.22 | 0.12 (0.09) | 0.16 | 1064 | 0.32 (0.08) | <0.001 | -0.04 (0.03) | 0.17 | -0.002 (0.12) | 0.99 | 0.32 (0.11) | 0.003 | -0.04 (0.03) | 0.18 | 0.01 (0.17) | 0.95 |
| *t*=fu | 995 | 0.18 (0.06) | 0.002 | -0.03 (0.03) | 0.29 | -0.10 (0.09) | 0.26 | 1064 | 0.31 (0.08) | <0.001 | -0.03 (0.03) | 0.19 | -0.11 (0.13) | 0.40 | 0.27 (0.12) | 0.03 | -0.03 (0.03) | 0.20 | -0.06 (0.19) | 0.73 |
| **Experiment Part 3: Descriptive norms (Average P3Q1 to P3Q2)** | | | | | | | | | | | | | | | | | | | | |
| *t*=base | 1051 | 0.14 (0.06) | 0.01 | -0.03 (0.02) | 0.17 | 0.13 (0.09) | 0.17 | 1064 | 0.27 (0.09) | 0.004 | -0.03 (0.02) | 0.14 | -0.05 (0.14) | 0.74 | 0.20 (0.12) | 0.08 | -0.03 (0.02) | 0.18 | 0.14 (0.18) | 0.44 |
| *t*=fu | 995 | 0.13 (0.06) | 0.02 | -0.03 (0.02) | 0.25 | -0.15 (0.09) | 0.07 | 1064 | 0.27 (0.09) | 0.003 | -0.03 (0.02) | 0.18 | -0.14 (0.14) | 0.31 | 0.16 (0.13) | 0.22 | -0.03 (0.02) | 0.18 | -0.003 (0.20) | 0.99 |
| **Donation to ASSIST/Dead Cool** | | | | | | | | | | | | | | | | | | | | |
| *t*=base | 1050 | 0.13 (0.06) | 0.03 | -0.07 (0.12) | 0.59 | 0.17 (0.09) | 0.06 | 1063 | 0.14 (0.10) | 0.14 | -0.02 (0.13) | 0.89 | -0.02 (0.14) | 0.89 | 0.27 (0.22) | 0.21 | -0.01 (0.13) | 0.91 | 0.06 (0.31) | 0.85 |
| *t*=fu | 994 | 0.34 (0.06) | <0.001 | -0.007 (0.13) | 0.96 | -0.03 (0.08) | 0.72 | 1063 | 0.46 (0.09) | <0.001 | 0.006 (0.12) | 0.96 | -0.07 (0.14) | 0.62 | 0.67 (0.14) | <0.001 | 0.02 (0.12) | 0.85 | 0.15 (0.22) | 0.48 |
| **IN1** | | | | | | | | | | | | | | | | | | | | |
| *t*=base | 1050 | -0.05 (0.07) | 0.47 | 0.04 (0.03) | 0.26 | -0.07 (0.09) | 0.44 | 1059 | 0.06 (0.13) | 0.65 | 0.04 (0.03) | 0.28 | 0.19 (0.19) | 0.34 | 0.37 (0.22) | 0.09 | 0.04 (0.03) | 0.27 | -0.03 (0.34) | 0.92 |
| *t*=fu | 1047 | 0.07 (0.06) | 0.25 | 0.04 (0.03) | 0.28 | 0.006 (0.10) | 0.95 | 1059 | -0.16 (0.12) | 0.20 | 0.04 (0.03) | 0.29 | 0.31 (0.22) | 0.17 | 0.25 (0.23) | 0.28 | 0.04 (0.03) | 0.31 | 0.26 (0.40) | 0.53 |
| **IN2** | | | | | | | | | | | | | | | | | | | | |
| *t*=base | 1052 | -0.01 (0.08) | 0.89 | -0.01 (0.02) | 0.55 | -0.001 (0.09) | 0.99 | 1061 | 0.20 (0.19) | 0.29 | -0.01 (0.02) | 0.70 | -0.51 (0.33) | 0.12 | 0.21 (0.58) | 0.72 | -0.02 (0.02) | 0.52 | -0.28 (0.64) | 0.67 |
| *t*=fu | 1049 | 0.15 (0.08) | 0.06 | -0.02 (0.02) | 0.48 | -0.03 (0.12) | 0.83 | 1061 | 0.14 (0.11) | 0.19 | -0.01 (0.02) | 0.56 | -0.27 (0.19) | 0.16 | 0.07 (0.28) | 0.80 | -0.02 (0.02) | 0.52 | -0.28 (0.38) | 0.47 |
| **IN3** | | | | | | | | | | | | | | | | | | | | |
| *t*=base | 1050 | 0.11 (0.07) | 0.11 | 0.03 (0.03) | 0.32 | -0.001 (0.12) | 0.99 | 1059 | 0.19 (0.12) | 0.10 | 0.03 (0.03) | 0.28 | -0.17 (0.16) | 0.29 | 0.25 (0.19) | 0.19 | 0.03 (0.03) | 0.34 | -0.18 (0.22) | 0.42 |
| *t*=fu | 1047 | 0.08 (0.06) | 0.13 | 0.04 (0.03) | 0.23 | 0.04 (0.07) | 0.60 | 1059 | -0.02 (0.10) | 0.87 | 0.04 (0.03) | 0.24 | -0.05 (0.14) | 0.73 | 0.10 (0.20) | 0.62 | 0.03 (0.03) | 0.27 | -0.04 (0.25) | 0.86 |
| **IN4** | | | | | | | | | | | | | | | | | | | | |
| *t*=base | 1048 | 0.03 (0.06) | 0.65 | -0.008 (0.04) | 0.85 | 0.03 (0.10) | 0.73 | 1058 | -0.06 (0.13) | 0.64 | -0.01 (0.04) | 0.76 | -0.11 (0.22) | 0.60 | 0.70 (0.32) | 0.03 | -0.02 (0.04) | 0.63 | 0.48 (0.43) | 0.26 |
| *t*=fu | 1046 | 0.06 (0.05) | 0.27 | -0.01 (0.04) | 0.76 | 0.03 (0.09) | 0.73 | 1058 | 0.19 (0.12) | 0.12 | -0.006 (0.04) | 0.88 | -0.20 (0.18) | 0.27 | 0.44 (0.22) | 0.05 | -0.01 (0.04) | 0.79 | 0.27 (0.32) | 0.40 |
| **IN5** | | | | | | | | | | | | | | | | | | | | |
| *t*=base | 1050 | -0.02 (0.06) | 0.78 | 0.06 (0.04) | 0.11 | -0.01 (0.09) | 0.89 | 1059 | -0.06 (0.11) | 0.56 | 0.06 (0.04) | 0.11 | 0.09 (0.16) | 0.56 | -0.05 (0.24) | 0.83 | 0.06 (0.04) | 0.12 | -0.27 (0.32) | 0.41 |
| *t*=fu | 1047 | 0.03 (0.06) | 0.64 | 0.06 (0.04) | 0.12 | 0.11 (0.10) | 0.24 | 1059 | 0.05 (0.11) | 0.69 | 0.06 (0.04) | 0.11 | 0.03 (0.17) | 0.88 | 0.12 (0.20) | 0.57 | 0.06 (0.04) | 0.10 | -0.47 (0.32) | 0.14 |
| **IN6** | | | | | | | | | | | | | | | | | | | | |
| *t*=base | 1051 | 0.20 (0.06) | 0.001 | 0.04 (0.04) | 0.30 | -0.01 (0.10) | 0.90 | 1060 | 0.17 (0.10) | 0.08 | 0.05 (0.04) | 0.26 | 0.11 (0.17) | 0.52 | 0.39 (0.16) | 0.02 | 0.04 (0.04) | 0.28 | 0.17 (0.24) | 0.49 |
| *t*=fu | 1048 | 0.25 (0.06) | <0.001 | 0.04 (0.04) | 0.32 | 0.06 (0.09) | 0.49 | 1060 | 0.27 (0.09) | 0.004 | 0.04 (0.04) | 0.29 | 0.13 (0.14) | 0.35 | 0.38 (0.16) | 0.02 | 0.04 (0.04) | 0.32 | 0.16 (0.21) | 0.43 |
| **IN7** | | | | | | | | | | | | | | | | | | | | |
| *t*=base | **1052** | **0.12 (0.06)** | **0.04** | **0.02 (0.04)** | **0.56** | **0.22 (0.09)** | **0.01** | 1061 | 0.19 (0.12) | 0.12 | 0.03 (0.04) | 0.49 | 0.14 (0.19) | 0.45 | 0.54 (0.18) | 0.004 | 0.02 (0.04) | 0.67 | 0.19 (0.25) | 0.47 |
| *t*=fu | 1049 | 0.20 (0.07) | 0.002 | 0.02 (0.04) | 0.58 | 0.15 (0.09) | 0.12 | 1061 | 0.22 (0.11) | 0.05 | 0.02 (0.04) | 0.53 | 0.24 (0.15) | 0.11 | 0.32 (0.18) | 0.08 | 0.02 (0.04) | 0.56 | 0.22 (0.22) | 0.32 |
| **Self-report injunctive norms (Average IN1 to IN7)** | | | | | | | | | | | | | | | | | | | | |
| *t*=base | 1045 | 0.06 (0.06) | 0.32 | 0.03 (0.02) | 0.24 | -0.04 (0.09) | 0.63 | 1055 | 0.05 (0.12) | 0.69 | 0.03 (0.02) | 0.22 | -0.06 (0.19) | 0.74 | 0.37 (0.18) | 0.04 | 0.02 (0.02) | 0.33 | -0.11 (0.24) | 0.64 |
| *t*=fu | 1043 | 0.20 (0.06) | <0.001 | 0.03 (0.02) | 0.24 | -0.03 (0.09) | 0.77 | 1055 | 0.20 (0.09) | 0.03 | 0.03 (0.02) | 0.25 | 0.01 (0.13) | 0.91 | 0.23 (0.16) | 0.14 | 0.02 (0.02) | 0.27 | -0.11 (0.22) | 0.61 |
| **DN1.1** | | | | | | | | | | | | | | | | | | | | |
| *t*=base | 1052 | 0.33 (0.11) | 0.003 | -0.02 (0.03) | 0.46 | 0.18 (0.16) | 0.24 | 1061 | 0.29 (0.14) | 0.05 | -0.03 (0.03) | 0.41 | 0.21 (0.21) | 0.33 | 1.01 (0.30) | 0.001 | -0.01 (0.03) | 0.65 | 0.21 (0.33) | 0.54 |
| *t*=fu | 1049 | 0.32 (0.08) | <0.001 | -0.02 (0.03) | 0.45 | 0.19 (0.12) | 0.12 | 1061 | 0.22 (0.14) | 0.12 | -0.03 (0.03) | 0.41 | 0.19 (0.21) | 0.37 | 0.56 (0.18) | 0.002 | -0.02 (0.03) | 0.47 | 0.50 (0.25) | 0.05 |
| **DN1.2** | | | | | | | | | | | | | | | | | | | | |
| *t*=base | **1053** | **0.12 (0.05)** | **0.01** | **-0.06 (0.03)** | **0.10** | **0.21 (0.08)** | **0.006** | 1062 | 0.06 (0.07) | 0.37 | -0.07 (0.04) | 0.06 | 0.26 (0.13) | 0.04 | 0.14 (0.09) | 0.12 | -0.06 (0.03) | 0.07 | 0.23 (0.13) | 0.07 |
| *t*=fu | 1050 | -0.006 (0.04) | 0.89 | -0.06 (0.03) | 0.07 | 0.13 (0.08) | 0.10 | 1062 | 0.02 (0.08) | 0.78 | -0.07 (0.04) | 0.05 | 0.33 (0.14) | 0.02 | 0.08 (0.09) | 0.37 | -0.06 (0.03) | 0.07 | 0.27 (0.14) | 0.04 |
| **DN1.3** | | | | | | | | | | | | | | | | | | | | |
| *t*=base | 1052 | 0.09 (0.05) | 0.04 | -0.06 (0.04) | 0.11 | 0.01 (0.07) | 0.87 | 1061 | 0.06 (0.09) | 0.51 | -0.06 (0.04) | 0.12 | 0.10 (0.14) | 0.46 | 0.10 (0.13) | 0.47 | -0.07 (0.04) | 0.09 | 0.32 (0.19) | 0.10 |
| *t*=fu | 1049 | 0.03 (0.05) | 0.57 | -0.06 (0.04) | 0.10 | -0.01 (0.07) | 0.85 | 1061 | 0.02 (0.09) | 0.80 | -0.06 (0.04) | 0.11 | 0.11 (0.13) | 0.38 | 0.03 (0.15) | 0.84 | -0.08 (0.04) | 0.07 | 0.47 (0.22) | 0.03 |
| **DN1.4** | | | | | | | | | | | | | | | | | | | | |
| *t*=base | 1052 | 0.01 (0.05) | 0.76 | -0.04 (0.03) | 0.21 | -0.007 (0.06) | 0.89 | 1061 | 0.07 (0.14) | 0.59 | -0.04 (0.03) | 0.22 | 0.09 (0.17) | 0.62 | 0.13 (0.14) | 0.36 | -0.04 (0.03) | 0.25 | -0.31 (0.22) | 0.15 |
| *t*=fu | 1049 | -0.09 (0.04) | 0.05 | -0.03 (0.03) | 0.31 | -0.05 (0.07) | 0.54 | 1061 | 0.10 (0.15) | 0.52 | -0.04 (0.03) | 0.20 | 0.15 (0.18) | 0.40 | -0.09 (0.17) | 0.61 | -0.04 (0.03) | 0.26 | -0.37 (0.24) | 0.13 |
| **DN1.5** | | | | | | | | | | | | | | | | | | | | |
| *t*=base | 1052 | 0.06 (0.05) | 0.20 | 0.007 (0.02) | 0.74 | 0.02 (0.06) | 0.71 | 1061 | 0.15 (0.12) | 0.20 | 0.008 (0.02) | 0.70 | 0.08 (0.19) | 0.69 | 0.23 (0.18) | 0.22 | 0.009 (0.02) | 0.68 | -0.06 (0.31) | 0.86 |
| *t*=fu | 1049 | -0.005 (0.05) | 0.92 | 0.009 (0.02) | 0.67 | 0.04 (0.09) | 0.68 | 1061 | 0.18 (0.11) | 0.10 | 0.009 (0.02) | 0.69 | 0.04 (0.18) | 0.85 | 0.08 (0.16) | 0.63 | 0.01 (0.02) | 0.65 | -0.06 (0.22) | 0.80 |
| **Self-report descriptive norms 1 (Average DN1.1 to DN1.5)** | | | | | | | | | | | | | | | | | | | | |
| *t*=base | 1050 | 0.12 (0.05) | 0.02 | -0.03 (0.02) | 0.11 | 0.13 (0.09) | 0.13 | 1059 | 0.06 (0.08) | 0.46 | -0.03 (0.02) | 0.11 | 0.22 (0.13) | 0.10 | 0.17 (0.11) | 0.11 | -0.03 (0.02) | 0.14 | 0.18 (0.14) | 0.19 |
| *t*=fu | 1047 | 0.04 (0.05) | 0.35 | -0.03 (0.02) | 0.10 | 0.10 (0.08) | 0.25 | 1059 | 0.02 (0.08) | 0.82 | -0.03 (0.02) | 0.09 | 0.28 (0.13) | 0.04 | 0.13 (0.10) | 0.22 | -0.03 (0.02) | 0.12 | 0.26 (0.14) | 0.06 |
| **DN2.1** | | | | | | | | | | | | | | | | | | | | |
| *t*=base | 1053 | 0.18 (0.07) | 0.01 | -0.06 (0.03) | 0.06 | 0.15 (0.11) | 0.15 | 1062 | 0.18 (0.11) | 0.10 | -0.07 (0.03) | 0.05 | 0.18 (0.16) | 0.26 | 0.23 (0.15) | 0.12 | -0.07 (0.03) | 0.05 | 0.33 (0.22) | 0.13 |
| *t*=fu | 1050 | 0.20 (0.07) | 0.003 | -0.06 (0.03) | 0.07 | 0.11 (0.09) | 0.21 | 1062 | 0.21 (0.12) | 0.07 | -0.07 (0.03) | 0.06 | 0.04 (0.17) | 0.82 | 0.12 (0.16) | 0.45 | -0.07 (0.03) | 0.04 | 0.38 (0.21) | 0.07 |
| **DN2.2** | | | | | | | | | | | | | | | | | | | | |
| *t*=base | 1053 | 0.19 (0.06) | 0.004 | -0.002 (0.04) | 0.96 | 0.08 (0.10) | 0.43 | 1062 | 0.32 (0.10) | 0.001 | 0.009 (0.04) | 0.82 | 0.01 (0.14) | 0.93 | 0.53 (0.17) | 0.002 | 0.02 (0.04) | 0.68 | -0.09 (0.20) | 0.64 |
| *t*=fu | 1050 | 0.10 (0.06) | 0.08 | -0.002 (0.04) | 0.95 | 0.02 (0.08) | 0.82 | 1062 | 0.19 (0.09) | 0.04 | 0.0006 (0.04) | 0.99 | 0.04 (0.14) | 0.77 | 0.41 (0.16) | 0.008 | 0.01 (0.04) | 0.82 | 0.05 (0.20) | 0.80 |
| **DN2.3** | | | | | | | | | | | | | | | | | | | | |
| *t*=base | 1053 | 0.28 (0.08) | <0.001 | 0.02 (0.03) | 0.46 | 0.001 (0.12) | 0.99 | 1062 | 0.60 (0.10) | <0.001 | 0.03 (0.03) | 0.38 | 0.06 (0.15) | 0.71 | 0.67 (0.13) | <0.001 | 0.03 (0.03) | 0.43 | 0.23 (0.20) | 0.23 |
| *t*=fu | 1050 | 0.19 (0.06) | 0.001 | 0.03 (0.03) | 0.32 | -0.04 (0.09) | 0.70 | 1062 | 0.42 (0.09) | <0.001 | 0.03 (0.03) | 0.43 | -0.08 (0.14) | 0.57 | 0.56 (0.12) | <0.001 | 0.03 (0.03) | 0.40 | 0.04 (0.18) | 0.81 |
| **Self-report descriptive norms 2 (Average DN2.1 to DN2.3)** | | | | | | | | | | | | | | | | | | | | |
| *t*=base | 1053 | 0.23 (0.07) | <0.001 | -0.01 (0.03) | 0.68 | 0.11 (0.11) | 0.31 | 1062 | 0.39 (0.10) | <0.001 | -0.006 (0.03) | 0.81 | 0.10 (0.14) | 0.50 | 0.46 (0.14) | 0.001 | -0.01 (0.03) | 0.70 | 0.26 (0.19) | 0.16 |
| *t*=fu | 1050 | 0.12 (0.06) | 0.05 | -0.006 (0.02) | 0.79 | 0.07 (0.09) | 0.41 | 1062 | 0.28 (0.09) | 0.001 | -0.01 (0.03) | 0.70 | 0.07 (0.13) | 0.61 | 0.37 (0.13) | 0.004 | -0.01 (0.03) | 0.67 | 0.27 (0.17) | 0.10 |
| **Self-report smoking behavior** | | | | | | | | | | | | | | | | | | | | |
| *t*=base | 1055 | 0.17 (0.08) | 0.02 | -0.03 (0.02) | 0.21 | 0.02 (0.08) | 0.82 | 1064 | 0.23 (0.11) | 0.04 | -0.03 (0.02) | 0.20 | -0.04 (0.14) | 0.79 | 0.58 (0.17) | 0.001 | -0.04 (0.02) | 0.14 | 0.10 (0.18) | 0.58 |
| *t*=fu | **1052** | **0.30 (0.07)** | **<0.001** | **-0.04 (0.03)** | **0.09** | **0.25 (0.10)** | **0.01** | 1064 | 0.24 (0.09) | 0.006 | -0.03 (0.02) | 0.17 | 0.14 (0.12) | 0.24 | 0.41 (0.10) | <0.001 | -0.03 (0.02) | 0.16 | 0.23 (0.13) | 0.08 |
| **Intentions** | | | | | | | | | | | | | | | | | | | | |
| *t*=base | 1051 | 0.19 (0.09) | 0.03 | -0.04 (0.05) | 0.51 | 0.09 (0.11) | 0.40 | 1060 | 0.48 (0.14) | 0.001 | -0.04 (0.05) | 0.46 | 0.31 (0.22) | 0.15 | 0.95 (0.26) | <0.001 | -0.05 (0.05) | 0.32 | 0.33 (0.40) | 0.40 |
| *t*=fu | 1048 | 0.23 (0.08) | 0.003 | -0.04 (0.06) | 0.42 | 0.17 (0.11) | 0.14 | 1060 | 0.11 (0.11) | 0.32 | -0.04 (0.05) | 0.48 | 0.14 (0.18) | 0.45 | 0.38 (0.15) | 0.01 | -0.05 (0.05) | 0.38 | 0.31 (0.24) | 0.19 |
| **Knowledge** | | | | | | | | | | | | | | | | | | | | |
| *t*=base | 1054 | 0.19 (0.06) | <0.001 | 0.12 (0.07) | 0.08 | 0.07 (0.08) | 0.40 | 1063 | 0.42 (0.08) | <0.001 | 0.09 (0.07) | 0.18 | 0.03 (0.12) | 0.83 | 0.42 (0.10) | <0.001 | 0.08 (0.07) | 0.23 | -0.15 (0.15) | 0.33 |
| *t*=fu | 1051 | 0.29 (0.06) | <0.001 | 0.12 (0.07) | 0.08 | 0.04 (0.09) | 0.65 | 1063 | 0.40 (0.08) | <0.001 | 0.09 (0.07) | 0.17 | 0.05 (0.11) | 0.68 | 0.38 (0.11) | 0.001 | 0.09 (0.07) | 0.19 | -0.11 (0.14) | 0.45 |
| **Attitudes** | | | | | | | | | | | | | | | | | | | | |
| *t*=base | 1042 | 0.16 (0.06) | 0.007 | 0.02 (0.03) | 0.45 | -0.16 (0.09) | 0.08 | 1051 | -0.03 (0.11) | 0.81 | 0.02 (0.03) | 0.43 | -0.14 (0.17) | 0.39 | 0.32 (0.22) | 0.14 | 0.02 (0.03) | 0.50 | -0.05 (0.31) | 0.87 |
| *t*=fu | 1039 | 0.18 (0.07) | 0.006 | 0.02 (0.03) | 0.52 | -0.01 (0.09) | 0.88 | 1051 | -0.09 (0.12) | 0.46 | 0.02 (0.03) | 0.44 | 0.04 (0.17) | 0.81 | 0.18 (0.18) | 0.31 | 0.02 (0.03) | 0.52 | 0.37 (0.25) | 0.14 |
| **Self-efficacy (Emotional)** | | | | | | | | | | | | | | | | | | | | |
| *t*=base | 1045 | 0.11 (0.08) | 0.18 | 0.05 (0.04) | 0.26 | -0.04 (0.13) | 0.77 | 1054 | 0.28 (0.12) | 0.02 | 0.05 (0.04) | 0.30 | -0.13 (0.22) | 0.56 | 0.41 (0.17) | 0.02 | 0.05 (0.04) | 0.30 | 0.06 (0.28) | 0.83 |
| *t*=fu | **1041** | **0.31 (0.07)** | **<0.001** | **0.04 (0.04)** | **0.38** | **0.31 (0.10)** | **0.002** | 1054 | 0.20 (0.09) | 0.03 | 0.04 (0.04) | 0.31 | 0.03 (0.15) | 0.82 | 0.36 (0.14) | 0.008 | 0.04 (0.04) | 0.40 | -0.01 (0.21) | 0.94 |
| **Self-efficacy (Friends)** | | | | | | | | | | | | | | | | | | | | |
| *t*=base | 1050 | 0.06 (0.07) | 0.37 | 0.02 (0.04) | 0.64 | -0.02 (0.11) | 0.88 | 1059 | 0.19 (0.11) | 0.09 | 0.02 (0.04) | 0.65 | -0.18 (0.19) | 0.35 | 0.13 (0.17) | 0.46 | 0.02 (0.04) | 0.63 | -0.09 (0.24) | 0.70 |
| *t*=fu | 1047 | 0.23 (0.07) | 0.001 | 0.01 (0.04) | 0.73 | 0.22 (0.10) | 0.02 | 1059 | 0.09 (0.10) | 0.35 | 0.02 (0.04) | 0.68 | -0.09 (0.17) | 0.60 | 0.20 (0.18) | 0.28 | 0.01 (0.04) | 0.72 | -0.11 (0.28) | 0.71 |
| **Self-efficacy (Opportunity)** | | | | | | | | | | | | | | | | | | | | |
| *t*=base | 1051 | 0.14 (0.08) | 0.08 | 0.002 (0.04) | 0.96 | 0.15 (0.13) | 0.25 | 1060 | 0.38 (0.14) | 0.009 | -0.00004 (0.04) | 1.00 | -0.02 (0.25) | 0.95 | 0.35 (0.21) | 0.10 | 0.002 (0.04) | 0.97 | 0.23 (0.30) | 0.44 |
| *t*=fu | **1048** | **0.24 (0.08)** | **0.002** | **-0.002 (0.04)** | **0.96** | **0.30 (0.11)** | **0.005** | 1060 | 0.21 (0.10) | 0.04 | -0.0007 (0.04) | 0.99 | -0.006 (0.16) | 0.97 | 0.23 (0.17) | 0.19 | 0.002 (0.04) | 0.97 | 0.14 (0.25) | 0.59 |
| **Perceived physical risks** | | | | | | | | | | | | | | | | | | | | |
| *t*=base | 1046 | 0.06 (0.05) | 0.29 | 2.85 (1.06) | 0.008 | -0.02 (0.09) | 0.80 | 1054 | 0.14 (0.09) | 0.12 | 2.56 (1.06) | 0.02 | -0.30 (0.13) | 0.03 | 0.05 (0.14) | 0.73 | 2.71 (1.07) | 0.01 | -0.23 (0.22) | 0.29 |
| *t*=fu | 1042 | 0.11 (0.06) | 0.05 | 2.54 (1.05) | 0.02 | -0.07 (0.10) | 0.46 | 1054 | 0.06 (0.11) | 0.60 | 2.64 (1.07) | 0.01 | -0.11 (0.16) | 0.50 | -0.23 (0.21) | 0.28 | 2.80 (1.08) | 0.01 | 0.02 (0.26) | 0.95 |
| **Perceived social risks** | | | | | | | | | | | | | | | | | | | | |
| *t*=base | 1049 | 0.14 (0.05) | 0.007 | 3.89 (1.15) | 0.001 | 0.04 (0.08) | 0.64 | 1058 | 0.24 (0.07) | 0.001 | 3.56 (1.17) | 0.002 | 0.005 (0.10) | 0.96 | 0.36 (0.10) | <0.001 | 3.42 (1.17) | 0.003 | 0.04 (0.14) | 0.79 |
| *t*=fu | 1046 | 0.12 (0.06) | 0.05 | 3.89 (1.15) | 0.001 | -0.09 (0.09) | 0.36 | 1058 | 0.20 (0.09) | 0.02 | 3.66 (1.17) | 0.002 | -0.09 (0.13) | 0.47 | 0.34 (0.12) | 0.004 | 3.47 (1.17) | 0.003 | -0.01 (0.16) | 0.95 |
| **Perceived addiction risks** | | | | | | | | | | | | | | | | | | | | |
| *t*=base | 990 | 0.20 (0.06) | <0.001 | 2.18 (1.24) | 0.08 | 0.05 (0.08) | 0.53 | 999 | 0.47 (0.09) | <0.001 | 1.32 (1.25) | 0.29 | 0.12 (0.13) | 0.34 | 0.68 (0.10) | <0.001 | 1.14 (1.23) | 0.35 | 0.13 (0.14) | 0.37 |
| *t*=fu | 983 | 0.12 (0.05) | 0.02 | 2.23 (1.27) | 0.08 | 0.02 (0.07) | 0.80 | 999 | 0.35 (0.08) | <0.001 | 1.72 (1.25) | 0.17 | 0.14 (0.11) | 0.21 | 0.54 (0.09) | <0.001 | 1.38 (1.24) | 0.26 | 0.12 (0.13) | 0.35 |
| **Perceived benefits** | | | | | | | | | | | | | | | | | | | | |
| *t*=base | 1008 | -0.03 (0.06) | 0.67 | 0.89 (1.03) | 0.39 | 0.03 (0.10) | 0.77 | 1017 | 0.07 (0.12) | 0.57 | 0.90 (1.02) | 0.38 | 0.22 (0.19) | 0.27 | 0.34 (0.21) | 0.10 | 1.01 (1.02) | 0.33 | 0.21 (0.37) | 0.57 |
| *t*=fu | 1004 | 0.02 (0.06) | 0.76 | 0.65 (1.03) | 0.53 | -0.16 (0.10) | 0.12 | 1017 | -0.09 (0.13) | 0.46 | 0.94 (1.02) | 0.36 | -0.16 (0.21) | 0.45 | -0.10 (0.25) | 0.71 | 0.91 (1.02) | 0.38 | 0.18 (0.41) | 0.66 |
| **Perceived behavioral control (easy to quit)** | | | | | | | | | | | | | | | | | | | | |
| *t*=base | 1049 | 0.27 (0.06) | <0.001 | -0.06 (0.07) | 0.39 | 0.19 (0.08) | 0.02 | 1058 | 0.50 (0.08) | <0.001 | -0.02 (0.07) | 0.73 | 0.11 (0.11) | 0.36 | 0.70 (0.10) | <0.001 | -0.004 (0.07) | 0.95 | 0.10 (0.14) | 0.47 |
| *t*=fu | 1046 | 0.17 (0.06) | 0.003 | -0.08 (0.07) | 0.28 | 0.08 (0.08) | 0.30 | 1058 | 0.49 (0.08) | <0.001 | -0.02 (0.07) | 0.73 | 0.11 (0.11) | 0.30 | 0.63 (0.10) | <0.001 | -0.01 (0.07) | 0.86 | 0.13 (0.13) | 0.31 |
| **Perceived behavioral control (to avoid smoking)** | | | | | | | | | | | | | | | | | | | | |
| *t*=base | 1052 | 0.08 (0.07) | 0.28 | 0.01 (0.06) | 0.83 | 0.25 (0.11) | 0.02 | 1061 | 0.35 (0.14) | 0.01 | 0.006 (0.06) | 0.92 | 0.23 (0.22) | 0.28 | 0.76 (0.23) | 0.001 | -0.02 (0.06) | 0.79 | 0.18 (0.35) | 0.61 |
| *t*=fu | 1049 | 0.09 (0.07) | 0.19 | 0.005 (0.06) | 0.93 | 0.07 (0.11) | 0.49 | 1061 | 0.11 (0.14) | 0.44 | 0.01 (0.06) | 0.87 | 0.17 (0.21) | 0.42 | 0.51 (0.18) | 0.005 | -0.01 (0.06) | 0.81 | 0.11 (0.27) | 0.68 |
| **Objectively measured smoking behavior** | | | | | | | | | | | | | | | | | | | | |
| *t*=base | 1017 | 0.35 (0.05) | <0.001 | -0.04 (0.07) | 0.51 | 0.03 (0.05) | 0.54 | 1024 | 0.36 (0.05) | <0.001 | -0.05 (0.07) | 0.42 | 0.05 (0.06) | 0.38 | 0.57 (0.06) | <0.001 | 0.007 (0.06) | 0.91 | 0.02 (0.06) | 0.71 |
| *t*=fu | 998 | 0.49 (0.08) | <0.001 | -0.04 (0.06) | 0.52 | -0.18 (0.08) | 0.04 | 1024 | 0.84 (0.06) | <0.001 | 0.009 (0.05) | 0.87 | -0.06 (0.07) | 0.41 | 0.86 (0.06) | <0.001 | 0.02 (0.06) | 0.75 | -0.05 (0.08) | 0.49 |
| **Smoking susceptibility^e^** | | | | | | | | | | | | | | | | | | | | |
|  | n | **Percent_-i,t_^f^** | | **M^g^** | | **M*Percent_-i,t_^h^** | | n | **Percent_-i,t_^f^** | | **M^g^** | | **M*Percent_-i,t_^h^** | | **Percent_-i,t_^f^** | | **M^g^** | | **M*Percent_-i,t_^h^** | |
|  |  | OR (SE) | p-value | OR (SE) | p-value | OR (SE) | p-value |  | OR (SE) | p-value | OR (SE) | p-value | OR (SE) | p-value | OR (SE) | p-value | OR (SE) | p-value | OR (SE) | p-value |
| *t*=base | 1051 | 1.14 (0.04) | <0.001 | 0.83 (0.10) | 0.11 | 0.97 (0.05) | 0.55 | 1060 | 0.09 (0.06) | 0.15 | -0.21 (0.12) | 0.08 | 0.13 (0.09) | 0.16 | 1.19 (0.11) | 0.07 | 0.81 (0.10) | 0.07 | 1.36 (0.19) | 0.02 |
| *t*=fu | 1048 | 1.13 (0.03) | <0.001 | 0.81 (0.10) | 0.07 | 0.96 (0.04) | 0.34 | 1060 | 1.16 (0.06) | 0.006 | 0.84 (0.10) | 0.14 | 1.01 (0.08) | 0.86 | 1.25 (0.09) | 0.003 | 0.85 (0.10) | 0.17 | 1.09 (0.12) | 0.40 |

IV: Independent variable; DV: Dependent variable; Ave: average of peer group (*i*); M: moderator (Need to belong: 1 [least need to belong] to 5 [most need to belong]); Percent: percentage of peer group (*-i*) classified as susceptible to commencing smoking; OR: odds ratio; SE: standard error.

^a^In each model the outcome variable is the focal participant’s (*i*) response to the relevant item at follow-up. The predictor variable is the average of the relevant group’s (*-i*) responses to the equivalent item at baseline (*t*=base) or follow-up (*t*=fu), where *–i*=(1) focal participant's nominated friends; (2) focal participant’s school class; (3) focal participant’s school year group. The moderator, and interaction of the moderator with the predictor variable, were also included as independent variables in all models. All models include robust (Huber White) standard errors specified using Stata’s ‘vce(robust)’ option. The following baseline variables are included as covariates in all models: gender (0=boy; 1=girl/prefer not to say), age (1=12 years or less; 2=13 years; 3=14 years or more), intervention (1=ASSIST; 2=Dead Cool), ethnicity (0=no ethnic minority; 1=ethnic minority), individuals' socio-economic status (NI: 1=NIMDM2017≤296.6; 2=296.6<NIMDM2017≤593.2; 3=NIMDM2017>593.2; Bogotá: 1=Informal settlement/Lowest/Low; 2=Middle-Low/Middle; 3=Middle-High/High), and baseline values of the outcome variable. The predictor variable, moderator variable, and baseline values of the outcome variable were mean-centered.

^b^Unstandardized regression coefficients representing the average change in the outcome variable for a one-unit increase in the predictor variable among participants who are average on need to belong.

^c^Unstandardized regression coefficients representing the average change in the outcome variable for a one-unit increase in need to belong among participants who are average on the predictor variable.

^d^Unstandardized regression coefficients representing the average change in the association between the outcome variable and the predictor variable for a one-unit increase in need to belong.

^e^Logistic regressions were run for models including focal participants' smoking susceptibility as the outcome variable, with robust (Huber White) standard errors specified using Stata’s ‘vce(robust)’ option. The predictor variable is the percentage of the relevant group (*-i*) classified as susceptible to commencing smoking at baseline (*t*=base) or follow-up (*t*=fu), where *–i*=(1) focal participant's nominated friends; (2) focal participant’s school class; (3) focal participant’s school year group. The moderator, and interaction of the moderator with the predictor variable, were also included as independent variables in all models. The following baseline variables are included as covariates in all models: gender (0=boy; 1=girl/prefer not to say), age (1=12 years or less; 2=13 years; 3=14 years or more), intervention (1=ASSIST; 2=Dead Cool), ethnicity (0=no ethnic minority; 1=ethnic minority), individuals' socio-economic status (NI: 1=NIMDM2017≤296.6; 2=296.6<NIMDM2017≤593.2; 3=NIMDM2017>593.2; Bogotá: 1=Informal settlement/Lowest/Low; 2=Middle-Low/Middle; 3=Middle-High/High), and baseline values of the outcome variable. Results are odds ratios, standard errors, and p-values. The predictor variable, and moderator variable were mean-centered.

^f^Odds ratios representing the multiplicative change in odds of being susceptible to commencing smoking for a 10% increase in the number of nominated friends/pupils in the same school class/pupils in the same school year group classified as being susceptible to commencing smoking (1 out of 10 nominated friends/pupils in the same school class/pupils in the same school year group; predictor variable) among participants who are average on need to belong.

^g^Odds ratios representing the multiplicative change in odds of being susceptible to commencing smoking for a one-unit increase in need to belong among participants who are average on the predictor variable.

^h^Ratio of ratios representing the ratio of: (1) the odds ratio representing the multiplicative change in odds of being susceptible to commencing smoking for a 10% increase in the number of nominated friends/pupils in the same school class/pupils in the same school year group classified as being susceptible to commencing smoking among participants who are one unit above average on need to belong; to (2) the odds ratio representing the multiplicative change in odds of being susceptible to commencing smoking for a 10% increase in the number of nominated friends/pupils in the same school class/pupils in the same school year group classified as being susceptible to commencing smoking among participants who are average on need to belong.

**Table S2.11.** Results of ordinary least squares linear regressions including interaction terms examining differences in peer influence effects according to openness for outcomes collected at follow-up.

|  | **Dependent variable: Participant responses to the outcome variable at follow-up^a^** | | | | | | | | | | | | | | | | | | | |
| --- | --- | --- | --- | --- | --- | --- | --- | --- | --- | --- | --- | --- | --- | --- | --- | --- | --- | --- | --- | --- |
|  | **(1) -i=Average of nominated friends** | | | | | | | n | **(2) -i=Average of school class** | | | | | | **(3) -i=Average of school year group** | | | | | |
| **IV** | n | **Ave_-i,t_^b^** | | **M^c^** | | **M*Ave_-i,t_^d^** | |  | **Ave_-i,t_^b^** | | **M^c^** | | **M*Ave_-i,t_^d^** | | **Ave_-i,t_^b^** | | **M^c^** | | **M*Ave_-i,t_^d^** | |
| **DV** |  | *b* (SE) | p-value | *b* (SE) | p-value | *b* (SE) | p-value |  | *b* (SE) | p-value | *b* (SE) | p-value | *b* (SE) | p-value | *b* (SE) | p-value | *b* (SE) | p-value | *b* (SE) | p-value |
| **P2S2** | | | | | | | | | | | | | | | | | | | | |
| *t*=base | 1030 | 0.36 (0.09) | <0.001 | -0.02 (0.02) | 0.12 | -0.04 (0.14) | 0.74 | 1043 | 0.45 (0.12) | <0.001 | -0.02 (0.02) | 0.17 | -0.05 (0.22) | 0.83 | 0.84 (0.18) | <0.001 | -0.02 (0.02) | 0.34 | 0.18 (0.31) | 0.56 |
| *t*=fu | 976 | 0.16 (0.06) | 0.01 | -0.02 (0.02) | 0.13 | -0.08 (0.11) | 0.45 | 1043 | 0.32 (0.10) | 0.002 | -0.02 (0.02) | 0.15 | -0.05 (0.17) | 0.75 | 0.61 (0.16) | <0.001 | -0.02 (0.02) | 0.24 | -0.01 (0.31) | 0.97 |
| **P2S3** | | | | | | | | | | | | | | | | | | | | |
| *t*=base | 1029 | 0.12 (0.07) | 0.10 | -0.05 (0.02) | 0.001 | -0.07 (0.11) | 0.53 | 1042 | 0.20 (0.16) | 0.21 | -0.05 (0.02) | 0.001 | 0.11 (0.22) | 0.61 | 0.14 (0.28) | 0.63 | -0.05 (0.02) | <0.001 | -0.57 (0.39) | 0.15 |
| *t*=fu | 975 | 0.11 (0.07) | 0.11 | -0.05 (0.02) | 0.003 | -0.10 (0.10) | 0.35 | 1042 | 0.23 (0.14) | 0.12 | -0.05 (0.02) | 0.001 | 0.08 (0.20) | 0.69 | 0.16 (0.27) | 0.56 | -0.05 (0.02) | 0.001 | -0.73 (0.39) | 0.06 |
| **P2S4** | | | | | | | | | | | | | | | | | | | | |
| *t*=base | 1027 | 0.11 (0.08) | 0.18 | -0.03 (0.02) | 0.06 | 0.09 (0.13) | 0.48 | 1040 | 0.14 (0.12) | 0.24 | -0.03 (0.02) | 0.05 | 0.03 (0.19) | 0.86 | 0.56 (0.32) | 0.09 | -0.03 (0.02) | 0.04 | 0.66 (0.47) | 0.16 |
| *t*=fu | 974 | 0.11 (0.07) | 0.11 | -0.04 (0.02) | 0.03 | -0.06 (0.11) | 0.56 | 1040 | 0.28 (0.12) | 0.02 | -0.03 (0.02) | 0.05 | 0.009 (0.18) | 0.96 | 0.49 (0.21) | 0.02 | -0.04 (0.02) | 0.03 | -0.04 (0.29) | 0.90 |
| **P2S5** | | | | | | | | | | | | | | | | | | | | |
| *t*=base | 1030 | 0.19 (0.06) | 0.001 | -0.02 (0.02) | 0.26 | 0.11 (0.11) | 0.33 | 1043 | 0.33 (0.10) | 0.001 | -0.03 (0.02) | 0.16 | -0.07 (0.16) | 0.66 | 0.45 (0.15) | 0.003 | -0.03 (0.02) | 0.17 | 0.02 (0.21) | 0.94 |
| *t*=fu | 976 | 0.17 (0.06) | 0.004 | -0.03 (0.02) | 0.14 | 0.17 (0.09) | 0.05 | 1043 | 0.45 (0.09) | <0.001 | -0.02 (0.02) | 0.28 | 0.10 (0.14) | 0.47 | 0.45 (0.14) | 0.002 | -0.03 (0.02) | 0.15 | -0.05 (0.18) | 0.79 |
| **P2S6** | | | | | | | | | | | | | | | | | | | | |
| *t*=base | 1027 | 0.16 (0.07) | 0.02 | -0.03 (0.02) | 0.08 | 0.20 (0.10) | 0.04 | 1040 | 0.38 (0.11) | 0.001 | -0.03 (0.02) | 0.08 | 0.24 (0.15) | 0.11 | **0.45 (0.19)** | **0.02** | **-0.04 (0.02)** | **0.06** | **0.60 (0.24)** | **0.01** |
| *t*=fu | 973 | 0.14 (0.06) | 0.01 | -0.03 (0.02) | 0.14 | 0.10 (0.09) | 0.27 | 1040 | 0.40 (0.10) | <0.001 | -0.03 (0.02) | 0.13 | 0.27 (0.15) | 0.07 | **0.50 (0.15)** | **0.001** | **-0.03 (0.02)** | **0.12** | **0.65 (0.22)** | **0.003** |
| **P2S7** | | | | | | | | | | | | | | | | | | | | |
| *t*=base | 1029 | 0.16 (0.05) | 0.003 | -0.06 (0.02) | 0.001 | 0.13 (0.09) | 0.13 | 1042 | 0.27 (0.09) | 0.003 | -0.07 (0.02) | <0.001 | 0.06 (0.13) | 0.62 | 0.30 (0.14) | 0.03 | -0.07 (0.02) | <0.001 | 0.28 (0.19) | 0.13 |
| *t*=fu | 976 | 0.25 (0.06) | <0.001 | -0.06 (0.02) | 0.002 | 0.0005 (0.08) | 1.00 | 1042 | 0.33 (0.09) | <0.001 | -0.06 (0.02) | 0.001 | 0.29 (0.13) | 0.03 | **0.28 (0.17)** | **0.10** | **-0.06 (0.02)** | **0.001** | **0.60 (0.22)** | **0.007** |
| **P2S8** | | | | | | | | | | | | | | | | | | | | |
| *t*=base | **1030** | **0.31 (0.05)** | **<0.001** | **-0.03 (0.02)** | **0.10** | **0.21 (0.08)** | **0.008** | 1043 | 0.32 (0.09) | <0.001 | -0.03 (0.02) | 0.11 | 0.10 (0.12) | 0.40 | 0.51 (0.14) | <0.001 | -0.04 (0.02) | 0.06 | 0.24 (0.20) | 0.21 |
| *t*=fu | 976 | 0.22 (0.06) | <0.001 | -0.02 (0.02) | 0.19 | 0.08 (0.09) | 0.39 | 1043 | 0.45 (0.08) | <0.001 | -0.03 (0.02) | 0.16 | 0.14 (0.12) | 0.21 | 0.51 (0.14) | <0.001 | -0.03 (0.02) | 0.13 | 0.19 (0.19) | 0.30 |
| **P2S9** | | | | | | | | | | | | | | | | | | | | |
| *t*=base | 1030 | 0.15 (0.07) | 0.03 | -0.04 (0.02) | 0.01 | 0.21 (0.11) | 0.04 | 1043 | 0.47 (0.13) | <0.001 | -0.04 (0.02) | 0.02 | 0.20 (0.18) | 0.27 | 0.54 (0.22) | 0.02 | -0.04 (0.02) | 0.02 | 0.37 (0.33) | 0.27 |
| *t*=fu | 976 | 0.24 (0.07) | <0.001 | -0.04 (0.02) | 0.03 | -0.02 (0.11) | 0.89 | 1043 | 0.36 (0.11) | 0.001 | -0.04 (0.02) | 0.02 | 0.31 (0.17) | 0.07 | 0.17 (0.21) | 0.43 | -0.04 (0.02) | 0.02 | 0.34 (0.29) | 0.25 |
| **Experiment Part 2: Injunctive norms (Average P2S2 to P2S9)** | | | | | | | | | | | | | | | | | | | | |
| *t*=base | **1022** | **0.23 (0.06)** | **<0.001** | **-0.03 (0.01)** | **0.006** | **0.25 (0.09)** | **0.007** | 1035 | 0.35 (0.10) | 0.001 | -0.03 (0.01) | 0.004 | 0.20 (0.15) | 0.17 | **0.47 (0.15)** | **0.002** | **-0.03 (0.01)** | **0.004** | **0.56 (0.21)** | **0.01** |
| *t*=fu | 970 | 0.25 (0.05) | <0.001 | -0.03 (0.01) | 0.005 | 0.05 (0.09) | 0.57 | 1035 | 0.44 (0.08) | <0.001 | -0.03 (0.01) | 0.01 | 0.26 (0.12) | 0.03 | 0.31 (0.14) | 0.03 | -0.03 (0.01) | 0.006 | 0.44 (0.20) | 0.03 |
| **P3Q1** | | | | | | | | | | | | | | | | | | | | |
| *t*=base | 1030 | 0.09 (0.07) | 0.16 | 0.03 (0.02) | 0.16 | -0.03 (0.10) | 0.74 | 1043 | 0.23 (0.11) | 0.04 | 0.03 (0.02) | 0.20 | 0.11 (0.15) | 0.45 | 0.15 (0.15) | 0.33 | 0.03 (0.02) | 0.22 | 0.07 (0.20) | 0.72 |
| *t*=fu | 976 | 0.11 (0.06) | 0.10 | 0.03 (0.02) | 0.19 | 0.13 (0.09) | 0.16 | 1043 | 0.27 (0.11) | 0.01 | 0.02 (0.02) | 0.30 | 0.06 (0.14) | 0.68 | 0.14 (0.18) | 0.41 | 0.03 (0.02) | 0.25 | -0.11 (0.23) | 0.62 |
| **P3Q2** | | | | | | | | | | | | | | | | | | | | |
| *t*=base | 1030 | 0.20 (0.06) | <0.001 | 0.02 (0.02) | 0.33 | 0.14 (0.09) | 0.11 | 1043 | 0.32 (0.08) | <0.001 | 0.02 (0.03) | 0.37 | 0.08 (0.13) | 0.54 | 0.33 (0.11) | 0.003 | 0.02 (0.03) | 0.42 | 0.04 (0.16) | 0.79 |
| *t*=fu | 976 | 0.20 (0.06) | 0.001 | 0.02 (0.03) | 0.43 | 0.08 (0.09) | 0.39 | 1043 | 0.32 (0.08) | <0.001 | 0.02 (0.03) | 0.40 | 0.09 (0.13) | 0.50 | 0.28 (0.12) | 0.02 | 0.02 (0.03) | 0.45 | -0.01 (0.18) | 0.96 |
| **Experiment Part 3: Descriptive norms (Average P3Q1 to P3Q2)** | | | | | | | | | | | | | | | | | | | | |
| *t*=base | 1030 | 0.16 (0.06) | 0.008 | 0.03 (0.02) | 0.17 | 0.09 (0.09) | 0.33 | 1043 | 0.27 (0.09) | 0.003 | 0.03 (0.02) | 0.21 | 0.12 (0.14) | 0.36 | 0.21 (0.12) | 0.07 | 0.03 (0.02) | 0.24 | 0.09 (0.17) | 0.61 |
| *t*=fu | 976 | 0.14 (0.06) | 0.02 | 0.03 (0.02) | 0.23 | 0.12 (0.09) | 0.18 | 1043 | 0.27 (0.09) | 0.002 | 0.02 (0.02) | 0.28 | 0.10 (0.13) | 0.43 | 0.16 (0.14) | 0.23 | 0.02 (0.02) | 0.27 | 0.02 (0.20) | 0.91 |
| **Donation to ASSIST/Dead Cool** | | | | | | | | | | | | | | | | | | | | |
| *t*=base | 1029 | 0.14 (0.06) | 0.02 | 0.13 (0.11) | 0.27 | 0.007 (0.08) | 0.93 | 1042 | 0.12 (0.10) | 0.20 | 0.15 (0.11) | 0.19 | -0.11 (0.14) | 0.46 | 0.26 (0.21) | 0.22 | 0.16 (0.11) | 0.16 | -0.51 (0.27) | 0.06 |
| *t*=fu | 975 | 0.35 (0.06) | <0.001 | 0.09 (0.11) | 0.40 | 0.03 (0.08) | 0.69 | 1042 | 0.51 (0.09) | <0.001 | 0.09 (0.11) | 0.39 | 0.05 (0.12) | 0.69 | 0.73 (0.14) | <0.001 | 0.11 (0.11) | 0.32 | -0.18 (0.18) | 0.32 |
| **IN1** | | | | | | | | | | | | | | | | | | | | |
| *t*=base | 1028 | -0.05 (0.07) | 0.49 | 0.02 (0.03) | 0.55 | 0.06 (0.11) | 0.56 | 1037 | 0.05 (0.13) | 0.70 | 0.03 (0.04) | 0.44 | -0.004 (0.18) | 0.98 | 0.32 (0.22) | 0.16 | 0.03 (0.04) | 0.47 | 0.16 (0.30) | 0.59 |
| *t*=fu | 1025 | 0.07 (0.06) | 0.24 | 0.02 (0.04) | 0.61 | 0.13 (0.08) | 0.12 | 1037 | -0.18 (0.13) | 0.14 | 0.03 (0.04) | 0.45 | 0.30 (0.20) | 0.13 | 0.24 (0.23) | 0.30 | 0.03 (0.04) | 0.45 | 0.16 (0.39) | 0.67 |
| **IN2** | | | | | | | | | | | | | | | | | | | | |
| *t*=base | 1030 | -0.0007 (0.08) | 0.99 | 0.01 (0.02) | 0.48 | 0.07 (0.08) | 0.37 | 1039 | 0.22 (0.19) | 0.26 | 0.01 (0.02) | 0.51 | 0.17 (0.22) | 0.43 | 0.40 (0.55) | 0.47 | 0.01 (0.02) | 0.45 | 0.23 (0.49) | 0.63 |
| *t*=fu | 1027 | 0.16 (0.08) | 0.04 | 0.01 (0.02) | 0.56 | -0.19 (0.10) | 0.06 | 1039 | 0.12 (0.11) | 0.28 | 0.01 (0.02) | 0.52 | -0.11 (0.14) | 0.43 | 0.12 (0.28) | 0.68 | 0.01 (0.02) | 0.52 | -0.06 (0.30) | 0.84 |
| **IN3** | | | | | | | | | | | | | | | | | | | | |
| *t*=base | 1028 | 0.12 (0.07) | 0.08 | -0.01 (0.03) | 0.68 | -0.05 (0.09) | 0.56 | 1037 | 0.21 (0.11) | 0.05 | -0.007 (0.03) | 0.79 | 0.15 (0.12) | 0.24 | 0.36 (0.19) | 0.07 | -0.004 (0.03) | 0.89 | 0.26 (0.20) | 0.19 |
| *t*=fu | 1025 | 0.09 (0.06) | 0.12 | -0.01 (0.03) | 0.68 | -0.09 (0.09) | 0.33 | 1037 | 0.02 (0.10) | 0.88 | -0.01 (0.03) | 0.57 | 0.08 (0.14) | 0.55 | 0.20 (0.20) | 0.30 | -0.01 (0.03) | 0.64 | 0.05 (0.22) | 0.82 |
| **IN4** | | | | | | | | | | | | | | | | | | | | |
| *t*=base | 1027 | 0.03 (0.06) | 0.56 | 0.06 (0.04) | 0.13 | -0.14 (0.08) | 0.09 | 1037 | -0.02 (0.13) | 0.89 | 0.05 (0.04) | 0.17 | -0.13 (0.19) | 0.49 | 0.61 (0.32) | 0.06 | 0.05 (0.04) | 0.16 | -0.24 (0.39) | 0.54 |
| *t*=fu | 1025 | 0.07 (0.06) | 0.23 | 0.05 (0.04) | 0.17 | -0.03 (0.08) | 0.71 | 1037 | 0.17 (0.12) | 0.15 | 0.05 (0.04) | 0.15 | -0.24 (0.18) | 0.19 | 0.39 (0.23) | 0.09 | 0.05 (0.04) | 0.17 | -0.24 (0.30) | 0.43 |
| **IN5** | | | | | | | | | | | | | | | | | | | | |
| *t*=base | 1029 | -0.01 (0.06) | 0.86 | -0.05 (0.04) | 0.17 | -0.03 (0.09) | 0.71 | 1038 | -0.09 (0.11) | 0.43 | -0.05 (0.03) | 0.13 | 0.07 (0.16) | 0.67 | -0.07 (0.24) | 0.77 | -0.05 (0.03) | 0.13 | -0.06 (0.31) | 0.84 |
| *t*=fu | 1026 | 0.04 (0.06) | 0.50 | -0.05 (0.03) | 0.15 | -0.10 (0.09) | 0.28 | 1038 | 0.05 (0.12) | 0.66 | -0.05 (0.03) | 0.16 | -0.11 (0.17) | 0.53 | 0.12 (0.20) | 0.55 | -0.05 (0.03) | 0.12 | 0.16 (0.30) | 0.60 |
| **IN6** | | | | | | | | | | | | | | | | | | | | |
| *t*=base | 1029 | 0.20 (0.06) | 0.001 | 0.01 (0.04) | 0.78 | -0.06 (0.09) | 0.47 | 1038 | 0.20 (0.10) | 0.05 | 0.02 (0.04) | 0.60 | 0.17 (0.15) | 0.24 | 0.43 (0.17) | 0.009 | 0.03 (0.04) | 0.46 | 0.19 (0.21) | 0.37 |
| *t*=fu | 1026 | 0.24 (0.06) | <0.001 | 0.01 (0.04) | 0.79 | -0.08 (0.08) | 0.31 | 1038 | 0.28 (0.09) | 0.003 | 0.02 (0.04) | 0.59 | 0.02 (0.13) | 0.88 | 0.44 (0.16) | 0.008 | 0.03 (0.04) | 0.51 | -0.01 (0.20) | 0.95 |
| **IN7** | | | | | | | | | | | | | | | | | | | | |
| *t*=base | 1030 | 0.10 (0.06) | 0.10 | 0.07 (0.03) | 0.03 | -0.05 (0.08) | 0.56 | 1039 | 0.22 (0.12) | 0.07 | 0.08 (0.03) | 0.01 | -0.007 (0.14) | 0.96 | 0.64 (0.19) | 0.001 | 0.09 (0.03) | 0.004 | -0.07 (0.21) | 0.72 |
| *t*=fu | 1027 | 0.18 (0.06) | 0.004 | 0.07 (0.03) | 0.03 | 0.03 (0.08) | 0.67 | 1039 | 0.23 (0.11) | 0.04 | 0.08 (0.03) | 0.01 | -0.02 (0.13) | 0.85 | 0.41 (0.18) | 0.02 | 0.09 (0.03) | 0.008 | -0.01 (0.18) | 0.95 |
| **Self-report injunctive norms (Average IN1 to IN7)** | | | | | | | | | | | | | | | | | | | | |
| *t*=base | 1024 | 0.08 (0.06) | 0.20 | 0.006 (0.02) | 0.78 | -0.12 (0.08) | 0.15 | 1034 | 0.08 (0.11) | 0.50 | 0.008 (0.02) | 0.69 | 0.03 (0.13) | 0.82 | 0.45 (0.18) | 0.01 | 0.01 (0.02) | 0.55 | -0.05 (0.22) | 0.82 |
| *t*=fu | 1022 | 0.20 (0.06) | <0.001 | 0.004 (0.02) | 0.84 | -0.03 (0.08) | 0.69 | 1034 | 0.21 (0.09) | 0.03 | 0.008 (0.02) | 0.68 | -0.05 (0.13) | 0.70 | 0.29 (0.16) | 0.06 | 0.008 (0.02) | 0.68 | -0.08 (0.20) | 0.69 |
| **DN1.1** | | | | | | | | | | | | | | | | | | | | |
| *t*=base | 1030 | 0.30 (0.11) | 0.004 | 0.02 (0.03) | 0.52 | -0.01 (0.13) | 0.91 | 1039 | 0.33 (0.15) | 0.03 | 0.02 (0.03) | 0.56 | 0.32 (0.23) | 0.16 | 0.91 (0.33) | 0.005 | 0.01 (0.04) | 0.71 | 0.28 (0.40) | 0.49 |
| *t*=fu | 1027 | 0.34 (0.09) | <0.001 | 0.01 (0.03) | 0.78 | 0.20 (0.11) | 0.07 | 1039 | 0.23 (0.14) | 0.10 | 0.02 (0.03) | 0.52 | 0.13 (0.23) | 0.57 | 0.58 (0.19) | 0.003 | 0.02 (0.03) | 0.63 | 0.26 (0.31) | 0.41 |
| **DN1.2** | | | | | | | | | | | | | | | | | | | | |
| *t*=base | 1031 | 0.13 (0.05) | 0.02 | 0.0006 (0.03) | 0.98 | 0.07 (0.07) | 0.29 | 1040 | 0.09 (0.07) | 0.20 | -0.0006 (0.03) | 0.98 | 0.13 (0.11) | 0.24 | 0.14 (0.09) | 0.12 | -0.003 (0.03) | 0.93 | 0.09 (0.11) | 0.37 |
| *t*=fu | 1028 | -0.005 (0.04) | 0.92 | 0.005 (0.03) | 0.87 | 0.08 (0.08) | 0.29 | 1040 | 0.02 (0.08) | 0.77 | 0.007 (0.03) | 0.82 | 0.06 (0.13) | 0.65 | 0.07 (0.09) | 0.43 | 0.004 (0.03) | 0.90 | 0.07 (0.11) | 0.54 |
| **DN1.3** | | | | | | | | | | | | | | | | | | | | |
| *t*=base | 1030 | 0.08 (0.05) | 0.07 | 0.01 (0.04) | 0.77 | -0.01 (0.06) | 0.81 | 1039 | 0.06 (0.08) | 0.47 | 0.008 (0.04) | 0.85 | 0.11 (0.14) | 0.43 | 0.10 (0.13) | 0.45 | 0.008 (0.04) | 0.86 | 0.06 (0.21) | 0.79 |
| *t*=fu | 1027 | 0.02 (0.05) | 0.66 | 0.02 (0.04) | 0.63 | -0.10 (0.07) | 0.17 | 1039 | 0.02 (0.08) | 0.84 | 0.01 (0.04) | 0.74 | 0.02 (0.14) | 0.89 | 0.06 (0.14) | 0.67 | 0.01 (0.04) | 0.81 | 0.04 (0.21) | 0.86 |
| **DN1.4** | | | | | | | | | | | | | | | | | | | | |
| *t*=base | 1031 | 0.006 (0.05) | 0.90 | 0.01 (0.03) | 0.67 | -0.005 (0.05) | 0.92 | 1040 | 0.05 (0.15) | 0.75 | 0.02 (0.03) | 0.48 | 0.001 (0.13) | 0.99 | 0.07 (0.14) | 0.64 | 0.02 (0.03) | 0.50 | 0.17 (0.19) | 0.35 |
| *t*=fu | 1028 | -0.08 (0.04) | 0.05 | 0.01 (0.03) | 0.66 | -0.03 (0.06) | 0.58 | 1040 | 0.10 (0.17) | 0.55 | 0.02 (0.03) | 0.47 | -0.006 (0.16) | 0.97 | -0.12 (0.17) | 0.49 | 0.02 (0.03) | 0.51 | 0.26 (0.24) | 0.28 |
| **DN1.5** | | | | | | | | | | | | | | | | | | | | |
| *t*=base | 1031 | 0.06 (0.05) | 0.23 | 0.004 (0.02) | 0.84 | -0.03 (0.07) | 0.70 | 1040 | 0.18 (0.12) | 0.13 | 0.004 (0.02) | 0.81 | -0.21 (0.15) | 0.17 | 0.28 (0.17) | 0.11 | 0.002 (0.02) | 0.90 | 0.35 (0.35) | 0.31 |
| *t*=fu | 1028 | -0.009 (0.05) | 0.85 | 0.004 (0.02) | 0.85 | 0.02 (0.07) | 0.76 | 1040 | 0.22 (0.11) | 0.06 | 0.003 (0.02) | 0.90 | -0.08 (0.17) | 0.62 | 0.14 (0.16) | 0.39 | 0.003 (0.02) | 0.89 | 0.11 (0.22) | 0.64 |
| **Self-report descriptive norms 1 (Average DN1.1 to DN1.5)** | | | | | | | | | | | | | | | | | | | | |
| *t*=base | 1029 | 0.11 (0.05) | 0.03 | 0.009 (0.02) | 0.59 | -0.002 (0.07) | 0.98 | 1038 | 0.08 (0.08) | 0.27 | 0.009 (0.02) | 0.59 | 0.07 (0.12) | 0.58 | 0.16 (0.11) | 0.13 | 0.008 (0.02) | 0.64 | 0.05 (0.14) | 0.73 |
| *t*=fu | 1026 | 0.04 (0.05) | 0.37 | 0.01 (0.02) | 0.56 | 0.04 (0.08) | 0.61 | 1038 | 0.04 (0.08) | 0.56 | 0.01 (0.02) | 0.58 | 0.07 (0.12) | 0.55 | 0.15 (0.10) | 0.14 | 0.008 (0.02) | 0.64 | 0.08 (0.13) | 0.55 |
| **DN2.1** | | | | | | | | | | | | | | | | | | | | |
| *t*=base | 1031 | 0.18 (0.08) | 0.02 | 0.003 (0.03) | 0.94 | 0.14 (0.10) | 0.18 | 1040 | 0.18 (0.11) | 0.09 | 0.007 (0.03) | 0.82 | 0.08 (0.15) | 0.58 | 0.19 (0.15) | 0.20 | 0.01 (0.03) | 0.74 | -0.13 (0.20) | 0.53 |
| *t*=fu | 1028 | 0.17 (0.07) | 0.01 | 0.02 (0.03) | 0.62 | -0.03 (0.08) | 0.72 | 1040 | 0.18 (0.12) | 0.11 | 0.01 (0.03) | 0.70 | -0.12 (0.16) | 0.46 | 0.11 (0.17) | 0.52 | 0.008 (0.03) | 0.80 | 0.04 (0.18) | 0.83 |
| **DN2.2** | | | | | | | | | | | | | | | | | | | | |
| *t*=base | 1031 | 0.18 (0.06) | 0.004 | 0.06 (0.04) | 0.11 | 0.20 (0.09) | 0.02 | 1040 | 0.31 (0.10) | 0.002 | 0.05 (0.04) | 0.18 | 0.21 (0.13) | 0.12 | 0.45 (0.17) | 0.009 | 0.05 (0.04) | 0.24 | 0.07 (0.19) | 0.71 |
| *t*=fu | 1028 | 0.09 (0.06) | 0.14 | 0.06 (0.04) | 0.09 | 0.11 (0.09) | 0.24 | 1040 | 0.16 (0.09) | 0.09 | 0.06 (0.04) | 0.14 | 0.18 (0.13) | 0.16 | 0.34 (0.16) | 0.03 | 0.05 (0.04) | 0.19 | 0.11 (0.19) | 0.58 |
| **DN2.3** | | | | | | | | | | | | | | | | | | | | |
| *t*=base | 1031 | 0.25 (0.08) | 0.001 | -0.03 (0.03) | 0.29 | 0.07 (0.11) | 0.52 | 1040 | 0.59 (0.10) | <0.001 | -0.05 (0.03) | 0.13 | 0.23 (0.14) | 0.10 | 0.64 (0.13) | <0.001 | -0.04 (0.03) | 0.19 | 0.13 (0.17) | 0.47 |
| *t*=fu | 1028 | 0.17 (0.06) | 0.003 | -0.02 (0.03) | 0.42 | 0.05 (0.10) | 0.66 | 1040 | 0.40 (0.09) | <0.001 | -0.04 (0.03) | 0.16 | 0.17 (0.13) | 0.19 | 0.53 (0.12) | <0.001 | -0.04 (0.03) | 0.19 | 0.08 (0.17) | 0.65 |
| **Self-report descriptive norms 2 (Average DN2.1 to DN2.3)** | | | | | | | | | | | | | | | | | | | | |
| *t*=base | 1031 | 0.22 (0.06) | 0.001 | 0.007 (0.02) | 0.77 | 0.19 (0.10) | 0.06 | 1040 | 0.36 (0.10) | <0.001 | 0.004 (0.02) | 0.88 | 0.14 (0.14) | 0.34 | 0.40 (0.14) | 0.004 | 0.006 (0.02) | 0.81 | 0.001 (0.18) | 0.99 |
| *t*=fu | 1028 | 0.10 (0.06) | 0.08 | 0.02 (0.02) | 0.44 | 0.07 (0.08) | 0.36 | 1040 | 0.23 (0.08) | 0.005 | 0.01 (0.02) | 0.63 | -0.005 (0.11) | 0.97 | 0.33 (0.13) | 0.01 | 0.007 (0.02) | 0.76 | 0.02 (0.16) | 0.92 |
| **Self-report smoking behavior** | | | | | | | | | | | | | | | | | | | | |
| *t*=base | 1035 | 0.13 (0.07) | 0.06 | 0.02 (0.02) | 0.28 | -0.08 (0.08) | 0.30 | 1044 | 0.23 (0.11) | 0.04 | 0.03 (0.02) | 0.14 | -0.13 (0.13) | 0.34 | 0.52 (0.17) | 0.002 | 0.04 (0.02) | 0.09 | 0.001 (0.18) | 1.00 |
| *t*=fu | 1032 | 0.24 (0.06) | <0.001 | 0.03 (0.02) | 0.24 | -0.05 (0.09) | 0.58 | 1044 | 0.22 (0.09) | 0.02 | 0.03 (0.02) | 0.13 | -0.04 (0.13) | 0.78 | 0.38 (0.11) | <0.001 | 0.04 (0.02) | 0.10 | -0.02 (0.13) | 0.87 |
| **Intentions** | | | | | | | | | | | | | | | | | | | | |
| *t*=base | 1031 | 0.15 (0.09) | 0.08 | 0.12 (0.05) | 0.01 | 0.13 (0.10) | 0.21 | 1040 | 0.49 (0.14) | 0.001 | 0.13 (0.05) | 0.01 | -0.30 (0.19) | 0.12 | 0.93 (0.25) | <0.001 | 0.13 (0.05) | 0.004 | -0.18 (0.34) | 0.58 |
| *t*=fu | 1028 | 0.19 (0.08) | 0.02 | 0.12 (0.05) | 0.01 | -0.03 (0.11) | 0.80 | 1040 | 0.09 (0.12) | 0.42 | 0.12 (0.05) | 0.02 | -0.25 (0.16) | 0.12 | 0.34 (0.15) | 0.02 | 0.12 (0.05) | 0.009 | -0.25 (0.19) | 0.21 |
| **Knowledge** | | | | | | | | | | | | | | | | | | | | |
| *t*=base | 1034 | 0.20 (0.06) | <0.001 | 0.03 (0.06) | 0.68 | -0.10 (0.07) | 0.15 | 1043 | 0.45 (0.08) | <0.001 | 0.05 (0.06) | 0.40 | -0.20 (0.11) | 0.07 | 0.47 (0.11) | <0.001 | 0.06 (0.06) | 0.36 | -0.12 (0.14) | 0.37 |
| *t*=fu | 1031 | 0.29 (0.06) | <0.001 | 0.06 (0.06) | 0.36 | -0.12 (0.08) | 0.13 | 1043 | 0.44 (0.08) | <0.001 | 0.06 (0.06) | 0.31 | -0.10 (0.10) | 0.34 | 0.44 (0.11) | <0.001 | 0.06 (0.06) | 0.31 | -0.07 (0.12) | 0.58 |
| **Attitudes** | | | | | | | | | | | | | | | | | | | | |
| *t*=base | 1023 | 0.15 (0.06) | 0.01 | 0.07 (0.03) | 0.02 | -0.004 (0.09) | 0.97 | 1032 | -0.05 (0.11) | 0.62 | 0.07 (0.03) | 0.02 | -0.08 (0.15) | 0.59 | 0.36 (0.22) | 0.10 | 0.07 (0.03) | 0.01 | -0.19 (0.35) | 0.59 |
| *t*=fu | 1020 | 0.16 (0.06) | 0.02 | 0.07 (0.03) | 0.02 | -0.19 (0.09) | 0.03 | 1032 | -0.11 (0.12) | 0.36 | 0.07 (0.03) | 0.02 | -0.18 (0.18) | 0.32 | 0.23 (0.17) | 0.19 | 0.07 (0.03) | 0.01 | -0.27 (0.27) | 0.31 |
| **Self-efficacy (Emotional)** | | | | | | | | | | | | | | | | | | | | |
| *t*=base | 1026 | 0.09 (0.07) | 0.24 | -0.01 (0.04) | 0.69 | -0.07 (0.11) | 0.52 | 1035 | 0.26 (0.12) | 0.03 | -0.006 (0.04) | 0.86 | -0.14 (0.17) | 0.41 | 0.37 (0.17) | 0.03 | -0.007 (0.04) | 0.85 | -0.40 (0.21) | 0.06 |
| *t*=fu | 1022 | 0.26 (0.07) | <0.001 | -0.006 (0.04) | 0.87 | 0.04 (0.10) | 0.68 | 1035 | 0.22 (0.09) | 0.02 | -0.002 (0.04) | 0.95 | -0.07 (0.12) | 0.53 | 0.37 (0.13) | 0.007 | -0.001 (0.04) | 0.98 | -0.25 (0.16) | 0.12 |
| **Self-efficacy (Friends)** | | | | | | | | | | | | | | | | | | | | |
| *t*=base | 1031 | 0.05 (0.06) | 0.41 | 0.007 (0.04) | 0.84 | 0.02 (0.09) | 0.85 | 1040 | 0.18 (0.11) | 0.10 | 0.02 (0.03) | 0.64 | -0.10 (0.16) | 0.52 | 0.13 (0.17) | 0.44 | 0.01 (0.03) | 0.68 | -0.38 (0.25) | 0.13 |
| *t*=fu | 1028 | 0.19 (0.07) | 0.004 | 0.007 (0.03) | 0.85 | 0.09 (0.09) | 0.33 | 1040 | 0.09 (0.10) | 0.39 | 0.02 (0.03) | 0.65 | -0.06 (0.13) | 0.63 | 0.23 (0.19) | 0.21 | 0.01 (0.03) | 0.67 | -0.31 (0.22) | 0.16 |
| **Self-efficacy (Opportunity)** | | | | | | | | | | | | | | | | | | | | |
| *t*=base | 1032 | 0.11 (0.07) | 0.13 | 0.0004 (0.03) | 0.99 | -0.01 (0.10) | 0.92 | 1041 | 0.38 (0.14) | 0.009 | 0.009 (0.03) | 0.76 | -0.17 (0.21) | 0.42 | 0.37 (0.20) | 0.07 | 0.008 (0.03) | 0.79 | -0.48 (0.25) | 0.05 |
| *t*=fu | 1029 | 0.17 (0.07) | 0.02 | 0.004 (0.03) | 0.90 | 0.09 (0.08) | 0.24 | 1041 | 0.22 (0.10) | 0.03 | 0.01 (0.03) | 0.70 | -0.14 (0.12) | 0.27 | 0.25 (0.17) | 0.15 | 0.007 (0.03) | 0.82 | -0.42 (0.18) | 0.02 |
| **Perceived physical risks** | | | | | | | | | | | | | | | | | | | | |
| *t*=base | 1028 | 0.07 (0.05) | 0.16 | 1.94 (0.97) | 0.05 | -0.09 (0.08) | 0.27 | 1036 | 0.18 (0.09) | 0.05 | 1.73 (0.97) | 0.07 | -0.09 (0.13) | 0.50 | 0.12 (0.15) | 0.41 | 1.77 (0.96) | 0.07 | -0.11 (0.22) | 0.62 |
| *t*=fu | 1024 | 0.12 (0.06) | 0.04 | 1.74 (0.97) | 0.07 | 0.01 (0.08) | 0.86 | 1036 | 0.12 (0.11) | 0.29 | 1.77 (0.98) | 0.07 | 0.04 (0.14) | 0.75 | -0.04 (0.21) | 0.86 | 1.64 (0.96) | 0.09 | -0.36 (0.23) | 0.12 |
| **Perceived social risks** | | | | | | | | | | | | | | | | | | | | |
| *t*=base | 1029 | 0.16 (0.05) | 0.002 | 1.50 (1.08) | 0.17 | 0.006 (0.07) | 0.93 | 1038 | 0.29 (0.07) | <0.001 | 1.69 (1.07) | 0.12 | 0.04 (0.10) | 0.73 | 0.44 (0.10) | <0.001 | 1.94 (1.07) | 0.07 | -0.08 (0.13) | 0.53 |
| *t*=fu | 1026 | 0.14 (0.06) | 0.02 | 1.16 (1.07) | 0.28 | -0.07 (0.08) | 0.36 | 1038 | 0.25 (0.08) | 0.003 | 1.63 (1.08) | 0.13 | 0.11 (0.12) | 0.37 | 0.45 (0.12) | <0.001 | 1.87 (1.08) | 0.08 | -0.07 (0.14) | 0.63 |
| **Perceived addiction risks** | | | | | | | | | | | | | | | | | | | | |
| *t*=base | 974 | 0.20 (0.06) | 0.001 | 0.51 (1.12) | 0.64 | 0.13 (0.08) | 0.08 | 983 | 0.48 (0.09) | <0.001 | 0.50 (1.10) | 0.65 | 0.09 (0.11) | 0.41 | 0.70 (0.11) | <0.001 | 0.87 (1.09) | 0.43 | 0.12 (0.13) | 0.33 |
| *t*=fu | 967 | 0.14 (0.05) | 0.007 | 0.10 (1.12) | 0.93 | 0.003 (0.07) | 0.96 | 983 | 0.38 (0.08) | <0.001 | 0.71 (1.12) | 0.53 | 0.18 (0.10) | 0.08 | 0.57 (0.09) | <0.001 | 0.84 (1.11) | 0.45 | 0.12 (0.12) | 0.29 |
| **Perceived benefits** | | | | | | | | | | | | | | | | | | | | |
| *t*=base | 990 | -0.03 (0.06) | 0.62 | 0.51 (0.98) | 0.61 | 0.14 (0.09) | 0.14 | 999 | 0.08 (0.12) | 0.53 | 0.58 (0.98) | 0.56 | 0.19 (0.19) | 0.31 | 0.34 (0.20) | 0.09 | 0.58 (0.98) | 0.56 | 0.13 (0.31) | 0.68 |
| *t*=fu | 986 | 0.02 (0.06) | 0.70 | 0.51 (0.99) | 0.61 | 0.15 (0.08) | 0.07 | 999 | -0.09 (0.13) | 0.49 | 0.62 (0.98) | 0.53 | 0.05 (0.19) | 0.79 | -0.11 (0.25) | 0.66 | 0.58 (0.98) | 0.55 | -0.17 (0.35) | 0.63 |
| **Perceived behavioral control (easy to quit)** | | | | | | | | | | | | | | | | | | | | |
| *t*=base | 1030 | 0.27 (0.06) | <0.001 | 0.16 (0.07) | 0.01 | -0.06 (0.08) | 0.47 | 1039 | 0.49 (0.08) | <0.001 | 0.14 (0.06) | 0.04 | -0.06 (0.11) | 0.59 | 0.65 (0.10) | <0.001 | 0.13 (0.06) | 0.04 | -0.14 (0.13) | 0.28 |
| *t*=fu | 1027 | 0.14 (0.06) | 0.02 | 0.15 (0.07) | 0.02 | -0.07 (0.08) | 0.39 | 1039 | 0.45 (0.08) | <0.001 | 0.13 (0.06) | 0.04 | -0.12 (0.11) | 0.26 | 0.57 (0.10) | <0.001 | 0.12 (0.06) | 0.06 | -0.08 (0.12) | 0.51 |
| **Perceived behavioral control (to avoid smoking)** | | | | | | | | | | | | | | | | | | | | |
| *t*=base | 1033 | 0.05 (0.07) | 0.44 | 0.08 (0.06) | 0.17 | -0.03 (0.10) | 0.75 | 1042 | 0.38 (0.14) | 0.008 | 0.10 (0.06) | 0.10 | -0.18 (0.19) | 0.32 | 0.82 (0.23) | <0.001 | 0.12 (0.06) | 0.04 | 0.09 (0.30) | 0.76 |
| *t*=fu | 1030 | 0.08 (0.07) | 0.24 | 0.07 (0.06) | 0.22 | -0.09 (0.10) | 0.37 | 1042 | 0.11 (0.14) | 0.43 | 0.08 (0.06) | 0.16 | -0.13 (0.20) | 0.53 | 0.55 (0.18) | 0.003 | 0.11 (0.06) | 0.06 | 0.13 (0.25) | 0.60 |
| **Objectively measured smoking behavior** | | | | | | | | | | | | | | | | | | | | |
| *t*=base | 997 | 0.38 (0.05) | <0.001 | 0.06 (0.06) | 0.31 | -0.07 (0.06) | 0.23 | 1004 | 0.37 (0.05) | <0.001 | 0.05 (0.06) | 0.40 | -0.06 (0.05) | 0.27 | 0.58 (0.06) | <0.001 | 0.01 (0.06) | 0.82 | -0.05 (0.06) | 0.41 |
| *t*=fu | 978 | 0.48 (0.08) | <0.001 | 0.05 (0.06) | 0.43 | -0.14 (0.08) | 0.07 | 1004 | 0.82 (0.06) | <0.001 | -0.005 (0.05) | 0.93 | 0.01 (0.06) | 0.87 | 0.85 (0.06) | <0.001 | -0.02 (0.06) | 0.73 | 0.02 (0.07) | 0.71 |
| **Smoking susceptibility^e^** | | | | | | | | | | | | | | | | | | | | |
|  | n | **Percent_-i,t_^f^** | | **M^g^** | | **M*Percent_-i,t_^h^** | | n | **Percent_-i,t_^f^** | | **M^g^** | | **M*Percent_-i,t_^h^** | | **Percent_-i,t_^f^** | | **M^g^** | | **M*Percent_-i,t_^h^** | |
|  |  | OR (SE) | p-value | OR (SE) | p-value | OR (SE) | p-value |  | OR (SE) | p-value | OR (SE) | p-value | OR (SE) | p-value | OR (SE) | p-value | OR (SE) | p-value | OR (SE) | p-value |
| *t*=base | 1031 | 1.15 (0.04) | <0.001 | 0.73 (0.09) | 0.007 | 1.01 (0.05) | 0.90 | 1040 | 1.09 (0.07) | 0.15 | 0.71 (0.08) | 0.004 | 0.98 (0.08) | 0.80 | 1.24 (0.12) | 0.03 | 0.72 (0.08) | 0.005 | 0.79 (0.11) | 0.08 |
| *t*=fu | 1028 | 1.13 (0.03) | <0.001 | 0.73 (0.08) | 0.007 | 0.94 (0.04) | 0.14 | 1040 | 1.17 (0.06) | 0.004 | 0.69 (0.08) | 0.002 | 0.99 (0.07) | 0.93 | 1.29 (0.10) | 0.001 | 0.70 (0.08) | 0.003 | 0.88 (0.09) | 0.22 |

IV: Independent variable; DV: Dependent variable; Ave: average of peer group (*i*); M: moderator ('Big Five' openness: 0 [least openness] to 4 [most openness]); Percent: percentage of peer group (*-i*) classified as susceptible to commencing smoking; OR: odds ratio; SE: standard error.

^a^In each model the outcome variable is the focal participant’s (*i*) response to the relevant item at follow-up. The predictor variable is the average of the relevant group’s (*-i*) responses to the equivalent item at baseline (*t*=base) or follow-up (*t*=fu), where *–i*=(1) focal participant's nominated friends; (2) focal participant’s school class; (3) focal participant’s school year group. The moderator, and interaction of the moderator with the predictor variable, were also included as independent variables in all models. All models include robust (Huber White) standard errors specified using Stata’s ‘vce(robust)’ option. The following baseline variables are included as covariates in all models: gender (0=boy; 1=girl/prefer not to say), age (1=12 years or less; 2=13 years; 3=14 years or more), intervention (1=ASSIST; 2=Dead Cool), ethnicity (0=no ethnic minority; 1=ethnic minority), individuals' socio-economic status (NI: 1=NIMDM2017≤296.6; 2=296.6<NIMDM2017≤593.2; 3=NIMDM2017>593.2; Bogotá: 1=Informal settlement/Lowest/Low; 2=Middle-Low/Middle; 3=Middle-High/High), and baseline values of the outcome variable. The predictor variable, moderator variable, and baseline values of the outcome variable were mean-centered.

^b^Unstandardized regression coefficients representing the average change in the outcome variable for a one-unit increase in the predictor variable among participants who are average on openness.

^c^Unstandardized regression coefficients representing the average change in the outcome variable for a one-unit increase in openness among participants who are average on the predictor variable.

^d^Unstandardized regression coefficients representing the average change in the association between the outcome variable and the predictor variable for a one-unit increase in openness.

^e^Logistic regressions were run for models including focal participants' smoking susceptibility as the outcome variable, with robust (Huber White) standard errors specified using Stata’s ‘vce(robust)’ option. The predictor variable is the percentage of the relevant group (*-i*) classified as susceptible to commencing smoking at baseline (*t*=base) or follow-up (*t*=fu), where *–i*=(1) focal participant's nominated friends; (2) focal participant’s school class; (3) focal participant’s school year group. The moderator, and interaction of the moderator with the predictor variable, were also included as independent variables in all models. The following baseline variables are included as covariates in all models: gender (0=boy; 1=girl/prefer not to say), age (1=12 years or less; 2=13 years; 3=14 years or more), intervention (1=ASSIST; 2=Dead Cool), ethnicity (0=no ethnic minority; 1=ethnic minority), individuals' socio-economic status (NI: 1=NIMDM2017≤296.6; 2=296.6<NIMDM2017≤593.2; 3=NIMDM2017>593.2; Bogotá: 1=Informal settlement/Lowest/Low; 2=Middle-Low/Middle; 3=Middle-High/High), and baseline values of the outcome variable. Results are odds ratios, standard errors, and p-values. The predictor variable, and moderator variable were mean-centered.

^f^Odds ratios representing the multiplicative change in odds of being susceptible to commencing smoking for a 10% increase in the number of nominated friends/pupils in the same school class/pupils in the same school year group classified as being susceptible to commencing smoking (1 out of 10 nominated friends/pupils in the same school class/pupils in the same school year group; predictor variable) among participants who are average on openness.

^g^Odds ratios representing the multiplicative change in odds of being susceptible to commencing smoking for a one-unit increase in openness among participants who are average on the predictor variable.

^h^Ratio of ratios representing the ratio of: (1) the odds ratio representing the multiplicative change in odds of being susceptible to commencing smoking for a 10% increase in the number of nominated friends/pupils in the same school class/pupils in the same school year group classified as being susceptible to commencing smoking among participants who are one unit above average on openness; to (2) the odds ratio representing the multiplicative change in odds of being susceptible to commencing smoking for a 10% increase in the number of nominated friends/pupils in the same school class/pupils in the same school year group classified as being susceptible to commencing smoking among participants who are average on openness.

**Table S2.12.** Results of ordinary least squares linear regressions including interaction terms examining differences in peer influence effects according to extraversion for outcomes collected at follow-up.

|  | **Dependent variable: Participant responses to the outcome variable at follow-up^a^** | | | | | | | | | | | | | | | | | | | |
| --- | --- | --- | --- | --- | --- | --- | --- | --- | --- | --- | --- | --- | --- | --- | --- | --- | --- | --- | --- | --- |
|  | **(1) -i=Average of nominated friends** | | | | | | | n | **(2) -i=Average of school class** | | | | | | **(3) -i=Average of school year group** | | | | | |
| **IV** | n | **Ave_-i,t_^b^** | | **M^c^** | | **M*Ave_-i,t_^d^** | |  | **Ave_-i,t_^b^** | | **M^c^** | | **M*Ave_-i,t_^d^** | | **Ave_-i,t_^b^** | | **M^c^** | | **M*Ave_-i,t_^d^** | |
| **DV** |  | *b* (SE) | p-value | *b* (SE) | p-value | *b* (SE) | p-value |  | *b* (SE) | p-value | *b* (SE) | p-value | *b* (SE) | p-value | *b* (SE) | p-value | *b* (SE) | p-value | *b* (SE) | p-value |
| **P2S2** | | | | | | | | | | | | | | | | | | | | |
| *t*=base | 1029 | 0.35 (0.09) | <0.001 | -0.008 (0.01) | 0.56 | -0.09 (0.08) | 0.28 | 1042 | 0.46 (0.12) | <0.001 | -0.009 (0.01) | 0.50 | 0.03 (0.15) | 0.82 | 0.84 (0.18) | <0.001 | -0.008 (0.01) | 0.56 | 0.17 (0.22) | 0.43 |
| *t*=fu | 978 | 0.17 (0.06) | 0.006 | -0.003 (0.01) | 0.80 | 0.14 (0.08) | 0.07 | 1042 | 0.33 (0.10) | 0.002 | -0.009 (0.01) | 0.52 | 0.03 (0.14) | 0.81 | 0.62 (0.17) | <0.001 | -0.008 (0.01) | 0.53 | -0.05 (0.22) | 0.83 |
| **P2S3** | | | | | | | | | | | | | | | | | | | | |
| *t*=base | 1029 | 0.11 (0.07) | 0.12 | -0.02 (0.01) | 0.14 | 0.04 (0.08) | 0.63 | 1042 | 0.22 (0.16) | 0.18 | -0.02 (0.01) | 0.20 | -0.05 (0.25) | 0.85 | 0.23 (0.29) | 0.42 | -0.02 (0.01) | 0.20 | -0.19 (0.34) | 0.58 |
| *t*=fu | 978 | 0.12 (0.07) | 0.08 | -0.01 (0.01) | 0.39 | 0.13 (0.09) | 0.14 | 1042 | 0.22 (0.14) | 0.13 | -0.02 (0.01) | 0.25 | 0.01 (0.22) | 0.95 | 0.18 (0.27) | 0.50 | -0.02 (0.01) | 0.22 | -0.40 (0.35) | 0.26 |
| **P2S4** | | | | | | | | | | | | | | | | | | | | |
| *t*=base | 1026 | 0.12 (0.08) | 0.12 | -0.04 (0.01) | 0.009 | 0.02 (0.08) | 0.80 | 1039 | 0.16 (0.12) | 0.21 | -0.03 (0.01) | 0.01 | 0.10 (0.16) | 0.54 | 0.57 (0.33) | 0.08 | -0.03 (0.01) | 0.01 | 0.27 (0.29) | 0.35 |
| *t*=fu | 976 | 0.14 (0.07) | 0.05 | -0.04 (0.01) | 0.007 | -0.04 (0.09) | 0.66 | 1039 | 0.27 (0.12) | 0.02 | -0.03 (0.01) | 0.01 | 0.10 (0.13) | 0.46 | 0.50 (0.20) | 0.02 | -0.03 (0.01) | 0.01 | -0.22 (0.21) | 0.30 |
| **P2S5** | | | | | | | | | | | | | | | | | | | | |
| *t*=base | 1029 | 0.22 (0.06) | <0.001 | -0.008 (0.02) | 0.64 | 0.09 (0.09) | 0.28 | **1042** | **0.34 (0.10)** | **0.001** | **-0.01 (0.02)** | **0.37** | **0.33 (0.13)** | **0.01** | 0.48 (0.15) | 0.001 | -0.01 (0.02) | 0.39 | 0.40 (0.19) | 0.04 |
| *t*=fu | **978** | **0.19 (0.06)** | **0.001** | **-0.02 (0.02)** | **0.30** | **0.20 (0.08)** | **0.01** | **1042** | **0.47 (0.09)** | **<0.001** | **-0.007 (0.02)** | **0.66** | **0.37 (0.13)** | **0.003** | 0.45 (0.14) | 0.001 | -0.01 (0.02) | 0.52 | 0.36 (0.17) | 0.04 |
| **P2S6** | | | | | | | | | | | | | | | | | | | | |
| *t*=base | 1026 | 0.17 (0.07) | 0.01 | -0.02 (0.02) | 0.24 | 0.08 (0.09) | 0.40 | 1039 | 0.38 (0.11) | 0.001 | -0.02 (0.02) | 0.32 | 0.12 (0.12) | 0.35 | 0.45 (0.19) | 0.02 | -0.02 (0.02) | 0.31 | 0.22 (0.22) | 0.30 |
| *t*=fu | 975 | 0.15 (0.06) | 0.009 | -0.01 (0.02) | 0.41 | 0.11 (0.08) | 0.17 | 1039 | 0.41 (0.10) | <0.001 | -0.01 (0.02) | 0.47 | 0.18 (0.12) | 0.15 | 0.51 (0.15) | 0.001 | -0.01 (0.02) | 0.41 | 0.20 (0.18) | 0.27 |
| **P2S7** | | | | | | | | | | | | | | | | | | | | |
| *t*=base | 1029 | 0.17 (0.06) | 0.002 | -0.04 (0.02) | 0.05 | 0.09 (0.09) | 0.30 | 1042 | 0.27 (0.09) | 0.002 | -0.04 (0.02) | 0.04 | -0.06 (0.12) | 0.58 | 0.28 (0.14) | 0.04 | -0.04 (0.02) | 0.04 | -0.11 (0.17) | 0.50 |
| *t*=fu | 978 | 0.26 (0.06) | <0.001 | -0.03 (0.02) | 0.07 | 0.04 (0.08) | 0.61 | 1042 | 0.37 (0.10) | <0.001 | -0.03 (0.02) | 0.07 | 0.09 (0.12) | 0.48 | 0.28 (0.17) | 0.10 | -0.04 (0.02) | 0.04 | -0.04 (0.19) | 0.82 |
| **P2S8** | | | | | | | | | | | | | | | | | | | | |
| *t*=base | 1029 | 0.29 (0.05) | <0.001 | -0.02 (0.02) | 0.26 | 0.19 (0.08) | 0.02 | 1042 | 0.30 (0.09) | 0.001 | -0.02 (0.02) | 0.30 | 0.22 (0.12) | 0.06 | 0.49 (0.14) | 0.001 | -0.02 (0.02) | 0.32 | 0.07 (0.18) | 0.72 |
| *t*=fu | 978 | 0.22 (0.06) | <0.001 | -0.009 (0.02) | 0.59 | 0.02 (0.08) | 0.80 | 1042 | 0.46 (0.08) | <0.001 | -0.01 (0.02) | 0.51 | 0.23 (0.11) | 0.05 | 0.50 (0.14) | <0.001 | -0.01 (0.02) | 0.42 | 0.08 (0.17) | 0.64 |
| **P2S9** | | | | | | | | | | | | | | | | | | | | |
| *t*=base | **1029** | **0.15 (0.07)** | **0.03** | **-0.03 (0.02)** | **0.04** | **0.25 (0.10)** | **0.01** | **1042** | **0.50 (0.13)** | **<0.001** | **-0.03 (0.01)** | **0.04** | **0.49 (0.18)** | **0.008** | **0.57 (0.23)** | **0.01** | **-0.03 (0.01)** | **0.03** | **0.85 (0.31)** | **0.006** |
| *t*=fu | 978 | 0.24 (0.07) | 0.001 | -0.02 (0.02) | 0.11 | 0.09 (0.09) | 0.32 | 1042 | 0.37 (0.11) | 0.001 | -0.03 (0.02) | 0.07 | 0.38 (0.17) | 0.02 | **0.16 (0.21)** | **0.45** | **-0.03 (0.01)** | **0.05** | **0.78 (0.29)** | **0.007** |
| **Experiment Part 2: Injunctive norms (Average P2S2 to P2S9)** | | | | | | | | | | | | | | | | | | | | |
| *t*=base | 1023 | 0.23 (0.06) | <0.001 | -0.02 (0.01) | 0.07 | 0.14 (0.08) | 0.08 | 1036 | 0.36 (0.10) | <0.001 | -0.02 (0.01) | 0.08 | 0.18 (0.13) | 0.19 | 0.46 (0.15) | 0.002 | -0.02 (0.01) | 0.08 | 0.28 (0.19) | 0.14 |
| *t*=fu | 973 | 0.25 (0.05) | <0.001 | -0.02 (0.01) | 0.10 | 0.11 (0.08) | 0.16 | 1036 | 0.46 (0.08) | <0.001 | -0.01 (0.01) | 0.18 | 0.25 (0.12) | 0.04 | 0.31 (0.14) | 0.03 | -0.02 (0.01) | 0.10 | 0.17 (0.18) | 0.33 |
| **P3Q1** | | | | | | | | | | | | | | | | | | | | |
| *t*=base | 1029 | 0.10 (0.07) | 0.13 | 0.03 (0.02) | 0.18 | -0.05 (0.09) | 0.56 | 1042 | 0.25 (0.11) | 0.03 | 0.03 (0.02) | 0.17 | -0.05 (0.15) | 0.74 | 0.16 (0.16) | 0.30 | 0.03 (0.02) | 0.17 | 0.01 (0.19) | 0.94 |
| *t*=fu | 978 | 0.10 (0.06) | 0.13 | 0.03 (0.02) | 0.25 | -0.05 (0.09) | 0.59 | 1042 | 0.29 (0.11) | 0.006 | 0.03 (0.02) | 0.22 | 0.06 (0.15) | 0.67 | 0.15 (0.18) | 0.41 | 0.03 (0.02) | 0.20 | -0.11 (0.23) | 0.63 |
| **P3Q2** | | | | | | | | | | | | | | | | | | | | |
| *t*=base | 1029 | 0.20 (0.05) | <0.001 | 0.04 (0.02) | 0.08 | 0.17 (0.08) | 0.04 | 1042 | 0.33 (0.08) | <0.001 | 0.04 (0.02) | 0.10 | 0.004 (0.11) | 0.97 | 0.33 (0.11) | 0.003 | 0.04 (0.02) | 0.10 | 0.08 (0.14) | 0.59 |
| *t*=fu | 978 | 0.19 (0.06) | 0.001 | 0.03 (0.02) | 0.23 | 0.10 (0.08) | 0.21 | 1042 | 0.31 (0.08) | <0.001 | 0.04 (0.02) | 0.13 | 0.13 (0.12) | 0.30 | 0.28 (0.12) | 0.02 | 0.04 (0.02) | 0.12 | 0.05 (0.17) | 0.74 |
| **Experiment Part 3: Descriptive norms (Average P3Q1 to P3Q2)** | | | | | | | | | | | | | | | | | | | | |
| *t*=base | 1029 | 0.16 (0.06) | 0.007 | 0.04 (0.02) | 0.09 | 0.09 (0.08) | 0.30 | 1042 | 0.28 (0.09) | 0.002 | 0.03 (0.02) | 0.09 | -0.009 (0.13) | 0.95 | 0.22 (0.12) | 0.07 | 0.03 (0.02) | 0.10 | 0.07 (0.15) | 0.65 |
| *t*=fu | 978 | 0.13 (0.06) | 0.02 | 0.03 (0.02) | 0.20 | 0.04 (0.08) | 0.67 | 1042 | 0.28 (0.09) | 0.002 | 0.03 (0.02) | 0.13 | 0.11 (0.13) | 0.37 | 0.16 (0.14) | 0.24 | 0.03 (0.02) | 0.11 | 0.03 (0.18) | 0.86 |
| **Donation to ASSIST/Dead Cool** | | | | | | | | | | | | | | | | | | | | |
| *t*=base | 1028 | 0.14 (0.06) | 0.02 | -0.22 (0.10) | 0.03 | -0.07 (0.07) | 0.33 | 1041 | 0.16 (0.10) | 0.11 | -0.20 (0.10) | 0.05 | -0.16 (0.11) | 0.14 | 0.34 (0.22) | 0.11 | -0.21 (0.10) | 0.05 | -0.09 (0.24) | 0.71 |
| *t*=fu | 977 | 0.36 (0.06) | <0.001 | -0.20 (0.10) | 0.05 | -0.05 (0.07) | 0.49 | 1041 | 0.51 (0.09) | <0.001 | -0.21 (0.10) | 0.04 | 0.04 (0.11) | 0.73 | 0.69 (0.15) | <0.001 | -0.19 (0.10) | 0.06 | 0.15 (0.17) | 0.37 |
| **IN1** | | | | | | | | | | | | | | | | | | | | |
| *t*=base | 1026 | -0.04 (0.07) | 0.53 | 0.009 (0.03) | 0.78 | 0.22 (0.10) | 0.02 | 1035 | 0.05 (0.13) | 0.70 | 0.01 (0.03) | 0.69 | 0.08 (0.20) | 0.70 | 0.37 (0.22) | 0.09 | 0.01 (0.03) | 0.72 | -0.09 (0.34) | 0.78 |
| *t*=fu | 1025 | 0.07 (0.06) | 0.26 | 0.008 (0.03) | 0.81 | 0.11 (0.09) | 0.23 | 1035 | -0.18 (0.12) | 0.16 | 0.01 (0.03) | 0.72 | 0.10 (0.20) | 0.62 | 0.28 (0.23) | 0.23 | 0.01 (0.03) | 0.74 | -0.54 (0.42) | 0.20 |
| **IN2** | | | | | | | | | | | | | | | | | | | | |
| *t*=base | 1028 | -0.003 (0.08) | 0.97 | 0.03 (0.02) | 0.08 | 0.07 (0.08) | 0.34 | 1037 | 0.23 (0.19) | 0.23 | 0.03 (0.02) | 0.09 | 0.03 (0.21) | 0.89 | 0.44 (0.56) | 0.44 | 0.03 (0.02) | 0.08 | -0.43 (0.48) | 0.37 |
| *t*=fu | 1027 | 0.18 (0.09) | 0.03 | 0.03 (0.02) | 0.05 | -0.21 (0.10) | 0.03 | 1037 | 0.12 (0.12) | 0.30 | 0.03 (0.02) | 0.06 | -0.28 (0.16) | 0.07 | 0.15 (0.28) | 0.60 | 0.03 (0.02) | 0.08 | -0.41 (0.31) | 0.18 |
| **IN3** | | | | | | | | | | | | | | | | | | | | |
| *t*=base | 1026 | 0.12 (0.06) | 0.07 | -0.02 (0.02) | 0.39 | -0.09 (0.09) | 0.35 | 1035 | 0.22 (0.11) | 0.05 | -0.02 (0.02) | 0.41 | 0.03 (0.13) | 0.80 | 0.33 (0.19) | 0.08 | -0.02 (0.02) | 0.45 | -0.26 (0.17) | 0.14 |
| *t*=fu | 1025 | 0.08 (0.06) | 0.14 | -0.02 (0.02) | 0.44 | -0.04 (0.07) | 0.58 | 1035 | 0.006 (0.10) | 0.96 | -0.02 (0.02) | 0.38 | -0.008 (0.13) | 0.95 | 0.22 (0.20) | 0.26 | -0.02 (0.02) | 0.39 | -0.36 (0.20) | 0.07 |
| **IN4** | | | | | | | | | | | | | | | | | | | | |
| *t*=base | 1025 | 0.02 (0.06) | 0.76 | 0.05 (0.03) | 0.14 | -0.11 (0.07) | 0.15 | 1035 | -0.007 (0.13) | 0.96 | 0.04 (0.03) | 0.19 | -0.22 (0.16) | 0.19 | 0.57 (0.32) | 0.08 | 0.04 (0.03) | 0.18 | -0.39 (0.34) | 0.25 |
| *t*=fu | 1025 | 0.06 (0.06) | 0.29 | 0.04 (0.03) | 0.21 | 0.002 (0.07) | 0.98 | 1035 | 0.15 (0.12) | 0.21 | 0.04 (0.03) | 0.18 | -0.24 (0.14) | 0.10 | 0.31 (0.23) | 0.16 | 0.04 (0.03) | 0.22 | -0.17 (0.27) | 0.51 |
| **IN5** | | | | | | | | | | | | | | | | | | | | |
| *t*=base | 1027 | -0.02 (0.06) | 0.76 | 0.007 (0.03) | 0.83 | -0.05 (0.07) | 0.53 | 1036 | -0.05 (0.11) | 0.64 | 0.003 (0.03) | 0.93 | 0.02 (0.14) | 0.87 | -0.006 (0.23) | 0.98 | 0.003 (0.03) | 0.93 | 0.08 (0.26) | 0.74 |
| *t*=fu | 1026 | 0.02 (0.06) | 0.70 | 0.004 (0.03) | 0.89 | 0.002 (0.08) | 0.98 | 1036 | 0.06 (0.12) | 0.61 | 0.003 (0.03) | 0.92 | 0.07 (0.13) | 0.58 | 0.10 (0.21) | 0.64 | 0.003 (0.03) | 0.93 | 0.10 (0.25) | 0.68 |
| **IN6** | | | | | | | | | | | | | | | | | | | | |
| *t*=base | 1027 | 0.19 (0.06) | 0.001 | 0.04 (0.03) | 0.26 | -0.12 (0.08) | 0.16 | 1036 | 0.21 (0.10) | 0.04 | 0.04 (0.03) | 0.24 | -0.05 (0.13) | 0.69 | 0.44 (0.17) | 0.008 | 0.04 (0.03) | 0.22 | -0.01 (0.20) | 0.96 |
| *t*=fu | 1026 | 0.23 (0.06) | <0.001 | 0.04 (0.03) | 0.24 | -0.09 (0.07) | 0.23 | 1036 | 0.26 (0.09) | 0.005 | 0.05 (0.04) | 0.17 | -0.15 (0.12) | 0.21 | 0.43 (0.16) | 0.007 | 0.04 (0.03) | 0.22 | -0.29 (0.19) | 0.13 |
| **IN7** | | | | | | | | | | | | | | | | | | | | |
| *t*=base | 1028 | 0.09 (0.06) | 0.11 | 0.09 (0.03) | 0.004 | -0.004 (0.07) | 0.96 | 1037 | 0.22 (0.12) | 0.07 | 0.10 (0.03) | 0.002 | -0.20 (0.15) | 0.18 | 0.59 (0.18) | 0.001 | 0.10 (0.03) | 0.001 | -0.39 (0.21) | 0.07 |
| *t*=fu | 1027 | 0.19 (0.06) | 0.004 | 0.09 (0.03) | 0.005 | 0.02 (0.09) | 0.85 | 1037 | 0.21 (0.11) | 0.06 | 0.10 (0.03) | 0.001 | -0.27 (0.14) | 0.05 | 0.40 (0.18) | 0.03 | 0.10 (0.03) | 0.001 | -0.42 (0.20) | 0.03 |
| **Self-report injunctive norms (Average IN1 to IN7)** | | | | | | | | | | | | | | | | | | | | |
| *t*=base | 1022 | 0.07 (0.06) | 0.21 | 0.03 (0.02) | 0.15 | -0.10 (0.08) | 0.23 | 1032 | 0.09 (0.12) | 0.42 | 0.03 (0.02) | 0.16 | -0.24 (0.13) | 0.07 | 0.46 (0.18) | 0.01 | 0.03 (0.02) | 0.14 | -0.37 (0.20) | 0.06 |
| *t*=fu | 1022 | 0.19 (0.06) | <0.001 | 0.02 (0.02) | 0.19 | -0.009 (0.08) | 0.91 | 1032 | 0.18 (0.09) | 0.05 | 0.03 (0.02) | 0.12 | -0.21 (0.12) | 0.07 | 0.31 (0.16) | 0.05 | 0.03 (0.02) | 0.16 | -0.46 (0.19) | 0.02 |
| **DN1.1** | | | | | | | | | | | | | | | | | | | | |
| *t*=base | 1029 | 0.28 (0.10) | 0.005 | 0.004 (0.03) | 0.88 | -0.21 (0.14) | 0.14 | 1038 | 0.27 (0.15) | 0.08 | -0.001 (0.03) | 0.97 | -0.04 (0.17) | 0.82 | 0.97 (0.32) | 0.002 | -0.008 (0.03) | 0.77 | 0.10 (0.25) | 0.69 |
| *t*=fu | 1028 | 0.31 (0.09) | 0.001 | -0.006 (0.03) | 0.82 | 0.15 (0.13) | 0.27 | 1038 | 0.19 (0.13) | 0.15 | -0.0004 (0.03) | 0.99 | -0.12 (0.17) | 0.49 | 0.59 (0.19) | 0.002 | -0.006 (0.03) | 0.81 | 0.12 (0.18) | 0.51 |
| **DN1.2** | | | | | | | | | | | | | | | | | | | | |
| *t*=base | 1029 | 0.13 (0.05) | 0.01 | 0.02 (0.02) | 0.40 | 0.07 (0.05) | 0.20 | 1038 | 0.08 (0.07) | 0.27 | 0.02 (0.02) | 0.48 | 0.10 (0.07) | 0.15 | 0.15 (0.09) | 0.11 | 0.02 (0.02) | 0.49 | -0.003 (0.08) | 0.97 |
| *t*=fu | 1028 | 0.002 (0.04) | 0.96 | 0.02 (0.02) | 0.39 | 0.06 (0.04) | 0.16 | 1038 | 0.04 (0.08) | 0.65 | 0.02 (0.02) | 0.44 | 0.10 (0.08) | 0.22 | 0.09 (0.10) | 0.34 | 0.02 (0.02) | 0.43 | -0.006 (0.08) | 0.95 |
| **DN1.3** | | | | | | | | | | | | | | | | | | | | |
| *t*=base | 1028 | 0.08 (0.05) | 0.09 | -0.002 (0.04) | 0.96 | 0.004 (0.06) | 0.95 | 1037 | 0.04 (0.08) | 0.61 | 0.0006 (0.04) | 0.99 | -0.03 (0.12) | 0.83 | 0.08 (0.13) | 0.54 | -0.001 (0.04) | 0.98 | 0.01 (0.15) | 0.94 |
| *t*=fu | 1027 | 0.03 (0.05) | 0.60 | 0.002 (0.04) | 0.96 | 0.02 (0.07) | 0.80 | 1037 | 0.02 (0.08) | 0.85 | 0.0008 (0.04) | 0.98 | -0.06 (0.11) | 0.58 | 0.01 (0.15) | 0.94 | 0.0009 (0.04) | 0.98 | 0.02 (0.17) | 0.90 |
| **DN1.4** | | | | | | | | | | | | | | | | | | | | |
| *t*=base | 1029 | 0.004 (0.05) | 0.93 | -0.006 (0.02) | 0.78 | -0.04 (0.04) | 0.36 | 1038 | 0.06 (0.15) | 0.67 | 0.0003 (0.02) | 0.99 | -0.11 (0.14) | 0.43 | 0.10 (0.15) | 0.50 | -0.002 (0.02) | 0.92 | 0.04 (0.14) | 0.74 |
| *t*=fu | 1028 | -0.09 (0.04) | 0.03 | -0.002 (0.02) | 0.94 | -0.08 (0.05) | 0.08 | 1038 | 0.12 (0.16) | 0.48 | -0.0007 (0.02) | 0.98 | -0.10 (0.22) | 0.66 | -0.10 (0.17) | 0.56 | -0.001 (0.02) | 0.96 | 0.06 (0.15) | 0.68 |
| **DN1.5** | | | | | | | | | | | | | | | | | | | | |
| *t*=base | 1029 | 0.08 (0.05) | 0.14 | 0.002 (0.02) | 0.91 | -0.03 (0.06) | 0.63 | 1038 | 0.22 (0.12) | 0.07 | 0.0006 (0.02) | 0.97 | -0.05 (0.16) | 0.73 | 0.40 (0.19) | 0.04 | -0.0002 (0.02) | 0.99 | -0.09 (0.19) | 0.65 |
| *t*=fu | 1028 | -0.007 (0.05) | 0.88 | 0.0008 (0.02) | 0.96 | 0.05 (0.06) | 0.36 | 1038 | 0.24 (0.12) | 0.04 | -0.002 (0.02) | 0.90 | -0.03 (0.14) | 0.81 | 0.15 (0.17) | 0.35 | 0.00004 (0.01) | 1.00 | -0.01 (0.14) | 0.94 |
| **Self-report descriptive norms 1 (Average DN1.1 to DN1.5)** | | | | | | | | | | | | | | | | | | | | |
| *t*=base | 1028 | 0.10 (0.05) | 0.05 | 0.005 (0.01) | 0.72 | -0.01 (0.07) | 0.82 | 1037 | 0.08 (0.08) | 0.30 | 0.006 (0.01) | 0.69 | 0.009 (0.09) | 0.92 | 0.17 (0.11) | 0.12 | 0.004 (0.01) | 0.76 | -0.01 (0.09) | 0.91 |
| *t*=fu | 1027 | 0.04 (0.05) | 0.37 | 0.006 (0.01) | 0.67 | 0.03 (0.06) | 0.67 | 1037 | 0.05 (0.08) | 0.55 | 0.006 (0.01) | 0.70 | 0.02 (0.09) | 0.80 | 0.13 (0.10) | 0.22 | 0.005 (0.01) | 0.72 | 0.03 (0.10) | 0.73 |
| **DN2.1** | | | | | | | | | | | | | | | | | | | | |
| *t*=base | 1029 | 0.16 (0.07) | 0.03 | -0.02 (0.03) | 0.49 | -0.06 (0.11) | 0.59 | 1038 | 0.16 (0.11) | 0.14 | -0.02 (0.03) | 0.37 | -0.17 (0.14) | 0.23 | 0.22 (0.15) | 0.15 | -0.03 (0.03) | 0.30 | -0.17 (0.15) | 0.27 |
| *t*=fu | 1028 | 0.18 (0.07) | 0.008 | -0.02 (0.03) | 0.55 | -0.08 (0.09) | 0.35 | 1038 | 0.21 (0.12) | 0.09 | -0.02 (0.03) | 0.40 | -0.17 (0.17) | 0.32 | 0.14 (0.17) | 0.41 | -0.03 (0.03) | 0.29 | -0.18 (0.13) | 0.18 |
| **DN2.2** | | | | | | | | | | | | | | | | | | | | |
| *t*=base | 1029 | 0.18 (0.06) | 0.005 | 0.07 (0.03) | 0.03 | -0.004 (0.07) | 0.95 | 1038 | 0.33 (0.10) | 0.001 | 0.06 (0.03) | 0.04 | -0.10 (0.09) | 0.26 | 0.51 (0.17) | 0.003 | 0.06 (0.03) | 0.07 | -0.08 (0.17) | 0.63 |
| *t*=fu | 1028 | 0.10 (0.06) | 0.11 | 0.07 (0.03) | 0.03 | 0.06 (0.07) | 0.42 | 1038 | 0.18 (0.09) | 0.05 | 0.06 (0.03) | 0.04 | -0.03 (0.10) | 0.76 | 0.38 (0.16) | 0.01 | 0.06 (0.03) | 0.06 | -0.06 (0.15) | 0.68 |
| **DN2.3** | | | | | | | | | | | | | | | | | | | | |
| *t*=base | 1029 | 0.26 (0.08) | 0.001 | -0.02 (0.03) | 0.45 | -0.02 (0.10) | 0.87 | 1038 | 0.59 (0.10) | <0.001 | -0.03 (0.03) | 0.24 | 0.05 (0.13) | 0.68 | 0.65 (0.13) | <0.001 | -0.03 (0.03) | 0.28 | -0.009 (0.15) | 0.95 |
| *t*=fu | 1028 | 0.20 (0.08) | 0.007 | -0.03 (0.03) | 0.36 | 0.05 (0.06) | 0.43 | 1038 | 0.40 (0.09) | <0.001 | -0.03 (0.03) | 0.30 | 0.05 (0.11) | 0.65 | 0.53 (0.12) | <0.001 | -0.03 (0.03) | 0.27 | -0.04 (0.14) | 0.78 |
| **Self-report descriptive norms 2 (Average DN2.1 to DN2.3)** | | | | | | | | | | | | | | | | | | | | |
| *t*=base | 1029 | 0.20 (0.07) | 0.002 | 0.01 (0.02) | 0.58 | -0.04 (0.07) | 0.63 | 1038 | 0.37 (0.10) | <0.001 | 0.006 (0.02) | 0.75 | -0.16 (0.10) | 0.12 | 0.43 (0.13) | 0.001 | 0.003 (0.02) | 0.89 | -0.09 (0.13) | 0.50 |
| *t*=fu | 1028 | 0.10 (0.06) | 0.10 | 0.01 (0.02) | 0.57 | 0.01 (0.06) | 0.86 | 1038 | 0.26 (0.08) | 0.002 | 0.007 (0.02) | 0.74 | -0.10 (0.09) | 0.27 | 0.35 (0.13) | 0.005 | 0.002 (0.02) | 0.90 | -0.08 (0.12) | 0.49 |
| **Self-report smoking behavior** | | | | | | | | | | | | | | | | | | | | |
| *t*=base | 1032 | 0.14 (0.07) | 0.05 | 0.004 (0.02) | 0.81 | -0.08 (0.07) | 0.27 | 1041 | 0.21 (0.11) | 0.06 | 0.005 (0.02) | 0.80 | 0.006 (0.09) | 0.95 | 0.56 (0.16) | 0.001 | 0.004 (0.02) | 0.83 | 0.04 (0.13) | 0.76 |
| *t*=fu | 1031 | 0.25 (0.07) | <0.001 | -0.001 (0.02) | 0.94 | -0.05 (0.08) | 0.57 | 1041 | 0.18 (0.08) | 0.03 | 0.001 (0.02) | 0.94 | 0.09 (0.08) | 0.25 | 0.39 (0.10) | <0.001 | 0.001 (0.02) | 0.94 | 0.06 (0.09) | 0.47 |
| **Intentions** | | | | | | | | | | | | | | | | | | | | |
| *t*=base | 1028 | 0.15 (0.09) | 0.07 | 0.09 (0.04) | 0.03 | 0.12 (0.09) | 0.15 | **1037** | **0.47 (0.14)** | **0.001** | **0.11 (0.04)** | **0.01** | **-0.57 (0.18)** | **0.002** | 0.92 (0.26) | <0.001 | 0.10 (0.04) | 0.02 | -0.49 (0.30) | 0.10 |
| *t*=fu | 1027 | 0.21 (0.08) | 0.007 | 0.10 (0.04) | 0.02 | -0.13 (0.09) | 0.13 | 1037 | 0.05 (0.12) | 0.67 | 0.10 (0.04) | 0.02 | -0.37 (0.15) | 0.02 | 0.33 (0.15) | 0.03 | 0.09 (0.04) | 0.02 | -0.33 (0.20) | 0.09 |
| **Knowledge** | | | | | | | | | | | | | | | | | | | | |
| *t*=base | 1031 | 0.20 (0.06) | <0.001 | 0.13 (0.06) | 0.02 | -0.01 (0.07) | 0.83 | 1040 | 0.43 (0.08) | <0.001 | 0.13 (0.05) | 0.01 | -0.14 (0.09) | 0.13 | 0.46 (0.11) | <0.001 | 0.14 (0.06) | 0.02 | -0.09 (0.12) | 0.47 |
| *t*=fu | 1030 | 0.29 (0.06) | <0.001 | 0.15 (0.06) | 0.008 | -0.05 (0.07) | 0.46 | 1040 | 0.42 (0.08) | <0.001 | 0.13 (0.06) | 0.02 | -0.04 (0.08) | 0.62 | 0.43 (0.11) | <0.001 | 0.14 (0.06) | 0.01 | -0.03 (0.12) | 0.83 |
| **Attitudes** | | | | | | | | | | | | | | | | | | | | |
| *t*=base | 1021 | 0.16 (0.06) | 0.007 | 0.01 (0.02) | 0.55 | 0.02 (0.08) | 0.77 | 1030 | -0.05 (0.11) | 0.65 | 0.02 (0.02) | 0.47 | -0.19 (0.12) | 0.12 | 0.32 (0.22) | 0.14 | 0.02 (0.02) | 0.51 | -0.33 (0.23) | 0.16 |
| *t*=fu | 1020 | 0.16 (0.07) | 0.02 | 0.02 (0.02) | 0.41 | -0.08 (0.08) | 0.31 | 1030 | -0.13 (0.12) | 0.28 | 0.02 (0.02) | 0.47 | -0.15 (0.13) | 0.23 | 0.20 (0.17) | 0.23 | 0.02 (0.02) | 0.49 | -0.30 (0.17) | 0.08 |
| **Self-efficacy (Emotional)** | | | | | | | | | | | | | | | | | | | | |
| *t*=base | 1024 | 0.09 (0.07) | 0.21 | 0.02 (0.03) | 0.57 | -0.005 (0.10) | 0.96 | 1033 | 0.27 (0.12) | 0.03 | 0.02 (0.03) | 0.48 | -0.08 (0.12) | 0.49 | 0.40 (0.17) | 0.02 | 0.02 (0.03) | 0.52 | -0.21 (0.16) | 0.20 |
| *t*=fu | 1022 | 0.27 (0.07) | <0.001 | 0.02 (0.03) | 0.52 | 0.008 (0.08) | 0.92 | 1033 | 0.21 (0.09) | 0.02 | 0.02 (0.03) | 0.42 | -0.12 (0.09) | 0.18 | 0.38 (0.13) | 0.005 | 0.02 (0.03) | 0.44 | -0.18 (0.12) | 0.16 |
| **Self-efficacy (Friends)** | | | | | | | | | | | | | | | | | | | | |
| *t*=base | 1028 | 0.05 (0.06) | 0.39 | -0.01 (0.03) | 0.65 | 0.008 (0.08) | 0.93 | 1037 | 0.18 (0.11) | 0.12 | -0.009 (0.03) | 0.74 | -0.11 (0.13) | 0.43 | 0.13 (0.17) | 0.46 | -0.01 (0.03) | 0.71 | -0.12 (0.18) | 0.49 |
| *t*=fu | 1027 | 0.19 (0.07) | 0.005 | -0.01 (0.03) | 0.64 | 0.06 (0.09) | 0.48 | 1037 | 0.08 (0.10) | 0.44 | -0.008 (0.03) | 0.78 | -0.17 (0.12) | 0.16 | 0.21 (0.18) | 0.25 | -0.009 (0.03) | 0.74 | -0.28 (0.18) | 0.12 |
| **Self-efficacy (Opportunity)** | | | | | | | | | | | | | | | | | | | | |
| *t*=base | 1029 | 0.12 (0.07) | 0.12 | 0.02 (0.03) | 0.50 | -0.02 (0.11) | 0.84 | 1038 | 0.37 (0.15) | 0.01 | 0.02 (0.03) | 0.47 | -0.02 (0.18) | 0.90 | 0.36 (0.20) | 0.08 | 0.02 (0.02) | 0.44 | -0.14 (0.19) | 0.46 |
| *t*=fu | 1028 | 0.15 (0.07) | 0.03 | 0.02 (0.03) | 0.46 | 0.04 (0.10) | 0.72 | 1038 | 0.21 (0.10) | 0.05 | 0.03 (0.03) | 0.34 | -0.13 (0.12) | 0.29 | 0.25 (0.17) | 0.14 | 0.02 (0.02) | 0.38 | -0.17 (0.17) | 0.31 |
| **Perceived physical risks** | | | | | | | | | | | | | | | | | | | | |
| *t*=base | 1025 | 0.07 (0.06) | 0.19 | 1.24 (0.81) | 0.13 | -0.03 (0.06) | 0.66 | 1033 | 0.17 (0.09) | 0.06 | 1.04 (0.80) | 0.19 | 0.05 (0.10) | 0.60 | 0.08 (0.15) | 0.59 | 1.12 (0.80) | 0.16 | 0.04 (0.17) | 0.82 |
| *t*=fu | 1023 | 0.12 (0.06) | 0.03 | 1.16 (0.81) | 0.16 | 0.02 (0.07) | 0.73 | 1033 | 0.11 (0.11) | 0.34 | 1.10 (0.80) | 0.17 | 0.14 (0.12) | 0.22 | -0.16 (0.22) | 0.48 | 1.13 (0.81) | 0.16 | 0.12 (0.17) | 0.49 |
| **Perceived social risks** | | | | | | | | | | | | | | | | | | | | |
| *t*=base | 1026 | 0.15 (0.05) | 0.003 | -0.12 (0.91) | 0.89 | 0.05 (0.06) | 0.41 | 1035 | 0.28 (0.07) | <0.001 | -0.10 (0.91) | 0.91 | 0.002 (0.08) | 0.98 | 0.41 (0.10) | <0.001 | 0.06 (0.90) | 0.94 | -0.13 (0.11) | 0.22 |
| *t*=fu | 1025 | 0.14 (0.06) | 0.02 | -0.07 (0.91) | 0.94 | -0.01 (0.08) | 0.89 | 1035 | 0.22 (0.09) | 0.009 | -0.12 (0.91) | 0.90 | 0.04 (0.10) | 0.68 | 0.41 (0.12) | 0.001 | 0.09 (0.91) | 0.93 | -0.11 (0.12) | 0.37 |
| **Perceived addiction risks** | | | | | | | | | | | | | | | | | | | | |
| *t*=base | 972 | 0.21 (0.06) | <0.001 | -0.58 (0.96) | 0.55 | 0.09 (0.07) | 0.17 | 981 | 0.50 (0.09) | <0.001 | -0.60 (0.95) | 0.53 | -0.002 (0.09) | 0.98 | 0.71 (0.10) | <0.001 | -0.51 (0.94) | 0.59 | 0.003 (0.11) | 0.98 |
| *t*=fu | 967 | 0.14 (0.05) | 0.006 | -0.34 (0.96) | 0.72 | -0.008 (0.06) | 0.89 | 981 | 0.40 (0.08) | <0.001 | -0.56 (0.95) | 0.55 | 0.06 (0.08) | 0.47 | 0.58 (0.09) | <0.001 | -0.54 (0.93) | 0.57 | 0.02 (0.10) | 0.83 |
| **Perceived benefits** | | | | | | | | | | | | | | | | | | | | |
| *t*=base | 987 | -0.03 (0.06) | 0.60 | -0.44 (0.82) | 0.60 | 0.09 (0.08) | 0.27 | 996 | 0.07 (0.12) | 0.56 | -0.51 (0.82) | 0.54 | 0.14 (0.16) | 0.40 | 0.35 (0.21) | 0.09 | -0.50 (0.83) | 0.55 | 0.02 (0.25) | 0.95 |
| *t*=fu | 985 | 0.02 (0.06) | 0.72 | -0.53 (0.83) | 0.52 | 0.09 (0.07) | 0.17 | 996 | -0.09 (0.12) | 0.45 | -0.52 (0.82) | 0.52 | 0.22 (0.15) | 0.14 | -0.11 (0.26) | 0.67 | -0.44 (0.82) | 0.59 | 0.09 (0.36) | 0.81 |
| **Perceived behavioral control (easy to quit)** | | | | | | | | | | | | | | | | | | | | |
| *t*=base | 1028 | 0.29 (0.06) | <0.001 | 0.04 (0.06) | 0.54 | 0.04 (0.07) | 0.58 | 1037 | 0.52 (0.08) | <0.001 | 0.04 (0.06) | 0.46 | 0.02 (0.09) | 0.86 | 0.71 (0.10) | <0.001 | 0.03 (0.06) | 0.59 | -0.02 (0.11) | 0.82 |
| *t*=fu | 1027 | 0.17 (0.06) | 0.005 | 0.03 (0.06) | 0.58 | 0.001 (0.07) | 0.99 | 1037 | 0.50 (0.08) | <0.001 | 0.02 (0.06) | 0.77 | -0.03 (0.09) | 0.73 | 0.64 (0.10) | <0.001 | 0.02 (0.06) | 0.70 | -0.009 (0.11) | 0.93 |
| **Perceived behavioral control (to avoid smoking)** | | | | | | | | | | | | | | | | | | | | |
| *t*=base | 1030 | 0.05 (0.07) | 0.46 | 0.03 (0.05) | 0.49 | 0.06 (0.10) | 0.54 | 1039 | 0.35 (0.14) | 0.01 | 0.05 (0.05) | 0.28 | -0.19 (0.17) | 0.27 | 0.80 (0.22) | <0.001 | 0.05 (0.05) | 0.26 | -0.001 (0.27) | 1.00 |
| *t*=fu | 1029 | 0.08 (0.07) | 0.24 | 0.04 (0.05) | 0.45 | -0.09 (0.10) | 0.34 | 1039 | 0.13 (0.14) | 0.37 | 0.04 (0.05) | 0.37 | -0.03 (0.17) | 0.88 | 0.55 (0.18) | 0.002 | 0.05 (0.05) | 0.30 | 0.06 (0.22) | 0.78 |
| **Objectively measured smoking behavior** | | | | | | | | | | | | | | | | | | | | |
| *t*=base | 993 | 0.35 (0.06) | <0.001 | 0.05 (0.05) | 0.34 | 0.01 (0.04) | 0.73 | 1000 | 0.36 (0.06) | <0.001 | 0.04 (0.05) | 0.48 | -0.03 (0.04) | 0.54 | 0.57 (0.06) | <0.001 | 0.01 (0.05) | 0.84 | -0.003 (0.05) | 0.95 |
| *t*=fu | 976 | 0.47 (0.09) | <0.001 | 0.02 (0.05) | 0.63 | -0.04 (0.06) | 0.53 | 1000 | 0.83 (0.06) | <0.001 | 0.02 (0.04) | 0.69 | -0.009 (0.05) | 0.86 | 0.85 (0.06) | <0.001 | -0.02 (0.04) | 0.68 | 0.02 (0.06) | 0.71 |
| **Smoking susceptibility^e^** | | | | | | | | | | | | | | | | | | | | |
|  | n | **Percent_-i,t_^f^** | | **M^g^** | | **M*Percent_-i,t_^h^** | | n | **Percent_-i,t_^f^** | | **M^g^** | | **M*Percent_-i,t_^h^** | | **Percent_-i,t_^f^** | | **M^g^** | | **M*Percent_-i,t_^h^** | |
|  |  | OR (SE) | p-value | OR (SE) | p-value | OR (SE) | p-value |  | OR (SE) | p-value | OR (SE) | p-value | OR (SE) | p-value | OR (SE) | p-value | OR (SE) | p-value | OR (SE) | p-value |
| *t*=base | 1028 | 1.14 (0.04) | <0.001 | 0.80 (0.08) | 0.02 | 1.00 (0.05) | 0.94 | 1037 | 1.08 (0.07) | 0.19 | 0.79 (0.08) | 0.01 | 0.91 (0.07) | 0.24 | **1.22 (0.12)** | **0.05** | **0.79 (0.08)** | **0.02** | **0.73 (0.09)** | **0.01** |
| *t*=fu | 1027 | 1.13 (0.03) | <0.001 | 0.79 (0.08) | 0.02 | 0.97 (0.04) | 0.45 | 1037 | 1.15 (0.06) | 0.01 | 0.76 (0.08) | 0.006 | 0.85 (0.06) | 0.02 | **1.27 (0.10)** | **0.002** | **0.78 (0.08)** | **0.01** | **0.78 (0.07)** | **0.006** |

IV: Independent variable; DV: Dependent variable; Ave: average of peer group (*i*); M: moderator ('Big Five' extraversion: 0 [least extraverted] to 4 [most extraverted]); Percent: percentage of peer group (*-i*) classified as susceptible to commencing smoking; OR: odds ratio; SE: standard error.

^a^In each model the outcome variable is the focal participant’s (*i*) response to the relevant item at follow-up. The predictor variable is the average of the relevant group’s (*-i*) responses to the equivalent item at baseline (*t*=base) or follow-up (*t*=fu), where *–i*=(1) focal participant's nominated friends; (2) focal participant’s school class; (3) focal participant’s school year group. The moderator, and interaction of the moderator with the predictor variable, were also included as independent variables in all models. All models include robust (Huber White) standard errors specified using Stata’s ‘vce(robust)’ option. The following baseline variables are included as covariates in all models: gender (0=boy; 1=girl/prefer not to say), age (1=12 years or less; 2=13 years; 3=14 years or more), intervention (1=ASSIST; 2=Dead Cool), ethnicity (0=no ethnic minority; 1=ethnic minority), individuals' socio-economic status (NI: 1=NIMDM2017≤296.6; 2=296.6<NIMDM2017≤593.2; 3=NIMDM2017>593.2; Bogotá: 1=Informal settlement/Lowest/Low; 2=Middle-Low/Middle; 3=Middle-High/High), and baseline values of the outcome variable. The predictor variable, moderator variable, and baseline values of the outcome variable were mean-centered.

^b^Unstandardized regression coefficients representing the average change in the outcome variable for a one-unit increase in the predictor variable among participants who are average on extraversion.

^c^Unstandardized regression coefficients representing the average change in the outcome variable for a one-unit increase in extraversion among participants who are average on the predictor variable.

^d^Unstandardized regression coefficients representing the average change in the association between the outcome variable and the predictor variable for a one-unit increase in extraversion.

^e^Logistic regressions were run for models including focal participants' smoking susceptibility as the outcome variable, with robust (Huber White) standard errors specified using Stata’s ‘vce(robust)’ option. The predictor variable is the percentage of the relevant group (*-i*) classified as susceptible to commencing smoking at baseline (*t*=base) or follow-up (*t*=fu), where *–i*=(1) focal participant's nominated friends; (2) focal participant’s school class; (3) focal participant’s school year group. The moderator, and interaction of the moderator with the predictor variable, were also included as independent variables in all models. The following baseline variables are included as covariates in all models: gender (0=boy; 1=girl/prefer not to say), age (1=12 years or less; 2=13 years; 3=14 years or more), intervention (1=ASSIST; 2=Dead Cool), ethnicity (0=no ethnic minority; 1=ethnic minority), individuals' socio-economic status (NI: 1=NIMDM2017≤296.6; 2=296.6<NIMDM2017≤593.2; 3=NIMDM2017>593.2; Bogotá: 1=Informal settlement/Lowest/Low; 2=Middle-Low/Middle; 3=Middle-High/High), and baseline values of the outcome variable. Results are odds ratios, standard errors, and p-values. The predictor variable, and moderator variable were mean-centered.

^f^Odds ratios representing the multiplicative change in odds of being susceptible to commencing smoking for a 10% increase in the number of nominated friends/pupils in the same school class/pupils in the same school year group classified as being susceptible to commencing smoking (1 out of 10 nominated friends/pupils in the same school class/pupils in the same school year group; predictor variable) among participants who are average on extraversion.

^g^Odds ratios representing the multiplicative change in odds of being susceptible to commencing smoking for a one-unit increase in extraversion among participants who are average on the predictor variable.

^h^Ratio of ratios representing the ratio of: (1) the odds ratio representing the multiplicative change in odds of being susceptible to commencing smoking for a 10% increase in the number of nominated friends/pupils in the same school class/pupils in the same school year group classified as being susceptible to commencing smoking among participants who are one unit above average on extraversion; to (2) the odds ratio representing the multiplicative change in odds of being susceptible to commencing smoking for a 10% increase in the number of nominated friends/pupils in the same school class/pupils in the same school year group classified as being susceptible to commencing smoking among participants who are average on extraversion.

**Table S2.13.** Results of ordinary least squares linear regressions including interaction terms examining differences in peer influence effects according to agreeableness for outcomes collected at follow-up.

|  | **Dependent variable: Participant responses to the outcome variable at follow-up^a^** | | | | | | | | | | | | | | | | | | | |
| --- | --- | --- | --- | --- | --- | --- | --- | --- | --- | --- | --- | --- | --- | --- | --- | --- | --- | --- | --- | --- |
|  | **(1) -i=Average of nominated friends** | | | | | | | n | **(2) -i=Average of school class** | | | | | | **(3) -i=Average of school year group** | | | | | |
| **IV** | n | **Ave_-i,t_^b^** | | **M^c^** | | **M*Ave_-i,t_^d^** | |  | **Ave_-i,t_^b^** | | **M^c^** | | **M*Ave_-i,t_^d^** | | **Ave_-i,t_^b^** | | **M^c^** | | **M*Ave_-i,t_^d^** | |
| **DV** |  | *b* (SE) | p-value | *b* (SE) | p-value | *b* (SE) | p-value |  | *b* (SE) | p-value | *b* (SE) | p-value | *b* (SE) | p-value | *b* (SE) | p-value | *b* (SE) | p-value | *b* (SE) | p-value |
| **P2S2** | | | | | | | | | | | | | | | | | | | | |
| *t*=base | 1013 | 0.37 (0.09) | <0.001 | -0.05 (0.01) | <0.001 | -0.15 (0.10) | 0.15 | 1025 | 0.50 (0.12) | <0.001 | -0.05 (0.01) | <0.001 | -0.14 (0.18) | 0.44 | 0.89 (0.19) | <0.001 | -0.05 (0.01) | 0.001 | -0.12 (0.25) | 0.64 |
| *t*=fu | 964 | 0.14 (0.06) | 0.02 | -0.05 (0.01) | 0.001 | -0.20 (0.13) | 0.13 | 1025 | 0.34 (0.11) | 0.001 | -0.05 (0.01) | 0.001 | -0.18 (0.16) | 0.25 | 0.66 (0.17) | <0.001 | -0.05 (0.02) | 0.001 | -0.30 (0.27) | 0.26 |
| **P2S3** | | | | | | | | | | | | | | | | | | | | |
| *t*=base | 1013 | 0.11 (0.07) | 0.12 | -0.06 (0.02) | <0.001 | -0.03 (0.10) | 0.79 | 1025 | 0.19 (0.16) | 0.25 | -0.06 (0.02) | <0.001 | -0.12 (0.23) | 0.59 | 0.18 (0.28) | 0.54 | -0.06 (0.02) | <0.001 | -0.06 (0.39) | 0.87 |
| *t*=fu | 964 | 0.10 (0.07) | 0.16 | -0.06 (0.02) | <0.001 | -0.13 (0.09) | 0.15 | 1025 | 0.18 (0.14) | 0.19 | -0.06 (0.02) | <0.001 | -0.33 (0.20) | 0.09 | 0.13 (0.27) | 0.62 | -0.06 (0.02) | <0.001 | -0.49 (0.39) | 0.21 |
| **P2S4** | | | | | | | | | | | | | | | | | | | | |
| *t*=base | 1010 | 0.10 (0.08) | 0.20 | -0.05 (0.02) | 0.003 | 0.01 (0.12) | 0.92 | 1022 | 0.12 (0.12) | 0.32 | -0.05 (0.02) | 0.002 | 0.02 (0.16) | 0.91 | 0.50 (0.33) | 0.13 | -0.05 (0.02) | 0.003 | 0.09 (0.31) | 0.77 |
| *t*=fu | 962 | 0.11 (0.07) | 0.12 | -0.05 (0.02) | 0.002 | -0.004 (0.09) | 0.96 | 1022 | 0.27 (0.12) | 0.02 | -0.05 (0.01) | 0.002 | 0.08 (0.14) | 0.57 | 0.46 (0.20) | 0.03 | -0.05 (0.01) | 0.001 | -0.04 (0.21) | 0.86 |
| **P2S5** | | | | | | | | | | | | | | | | | | | | |
| *t*=base | 1013 | 0.19 (0.06) | 0.002 | -0.06 (0.02) | 0.001 | -0.05 (0.09) | 0.62 | 1025 | 0.32 (0.10) | 0.001 | -0.07 (0.02) | <0.001 | -0.01 (0.15) | 0.92 | 0.48 (0.15) | 0.001 | -0.07 (0.02) | <0.001 | 0.26 (0.20) | 0.21 |
| *t*=fu | 964 | 0.17 (0.06) | 0.002 | -0.08 (0.02) | <0.001 | 0.03 (0.08) | 0.76 | 1025 | 0.47 (0.09) | <0.001 | -0.06 (0.02) | 0.001 | 0.06 (0.14) | 0.69 | 0.46 (0.14) | 0.001 | -0.07 (0.02) | <0.001 | 0.10 (0.19) | 0.60 |
| **P2S6** | | | | | | | | | | | | | | | | | | | | |
| *t*=base | 1010 | 0.16 (0.07) | 0.02 | -0.03 (0.02) | 0.10 | -0.02 (0.12) | 0.87 | 1022 | 0.39 (0.12) | 0.001 | -0.03 (0.02) | 0.15 | 0.10 (0.15) | 0.49 | 0.40 (0.19) | 0.04 | -0.03 (0.02) | 0.17 | 0.49 (0.23) | 0.04 |
| *t*=fu | 961 | 0.15 (0.06) | 0.01 | -0.03 (0.02) | 0.18 | -0.08 (0.08) | 0.31 | 1022 | 0.43 (0.10) | <0.001 | -0.03 (0.02) | 0.21 | 0.03 (0.17) | 0.84 | 0.48 (0.16) | 0.002 | -0.03 (0.02) | 0.21 | 0.30 (0.22) | 0.17 |
| **P2S7** | | | | | | | | | | | | | | | | | | | | |
| *t*=base | 1013 | 0.15 (0.06) | 0.007 | -0.05 (0.02) | 0.01 | -0.02 (0.09) | 0.84 | 1025 | 0.26 (0.09) | 0.004 | -0.05 (0.02) | 0.01 | -0.14 (0.13) | 0.30 | 0.30 (0.14) | 0.04 | -0.05 (0.02) | 0.01 | 0.01 (0.20) | 0.95 |
| *t*=fu | 964 | 0.26 (0.06) | <0.001 | -0.05 (0.02) | 0.01 | -0.03 (0.08) | 0.74 | 1025 | 0.39 (0.09) | <0.001 | -0.04 (0.02) | 0.04 | 0.21 (0.14) | 0.13 | 0.31 (0.17) | 0.08 | -0.04 (0.02) | 0.03 | 0.36 (0.23) | 0.13 |
| **P2S8** | | | | | | | | | | | | | | | | | | | | |
| *t*=base | 1013 | 0.29 (0.05) | <0.001 | -0.06 (0.02) | 0.001 | -0.04 (0.08) | 0.61 | 1025 | 0.30 (0.09) | 0.001 | -0.06 (0.02) | 0.001 | -0.01 (0.13) | 0.94 | 0.48 (0.14) | 0.001 | -0.06 (0.02) | <0.001 | 0.04 (0.20) | 0.82 |
| *t*=fu | 964 | 0.22 (0.06) | <0.001 | -0.06 (0.02) | 0.001 | -0.02 (0.08) | 0.83 | 1025 | 0.45 (0.08) | <0.001 | -0.06 (0.02) | 0.001 | 0.08 (0.12) | 0.49 | 0.50 (0.14) | <0.001 | -0.06 (0.02) | <0.001 | 0.12 (0.18) | 0.50 |
| **P2S9** | | | | | | | | | | | | | | | | | | | | |
| *t*=base | 1012 | 0.15 (0.07) | 0.03 | -0.04 (0.02) | 0.03 | -0.12 (0.11) | 0.26 | 1024 | 0.49 (0.13) | <0.001 | -0.04 (0.02) | 0.03 | -0.37 (0.20) | 0.06 | 0.49 (0.22) | 0.03 | -0.04 (0.02) | 0.03 | -0.50 (0.35) | 0.15 |
| *t*=fu | **963** | **0.25 (0.07)** | **<0.001** | **-0.04 (0.02)** | **0.02** | **-0.25 (0.10)** | **0.01** | 1024 | 0.35 (0.11) | 0.001 | -0.04 (0.02) | 0.03 | -0.22 (0.19) | 0.25 | 0.19 (0.21) | 0.35 | -0.04 (0.02) | 0.02 | -0.27 (0.33) | 0.40 |
| **Experiment Part 2: Injunctive norms (Average P2S2 to P2S9)** | | | | | | | | | | | | | | | | | | | | |
| *t*=base | 1006 | 0.23 (0.06) | <0.001 | -0.03 (0.01) | 0.001 | -0.05 (0.09) | 0.57 | 1018 | 0.37 (0.10) | <0.001 | -0.03 (0.01) | 0.001 | -0.04 (0.15) | 0.80 | 0.46 (0.15) | 0.002 | -0.03 (0.01) | 0.002 | 0.28 (0.23) | 0.22 |
| *t*=fu | 958 | 0.25 (0.05) | <0.001 | -0.04 (0.01) | <0.001 | -0.13 (0.08) | 0.11 | 1018 | 0.47 (0.08) | <0.001 | -0.03 (0.01) | 0.004 | 0.10 (0.13) | 0.45 | 0.34 (0.14) | 0.02 | -0.03 (0.01) | 0.002 | 0.27 (0.20) | 0.18 |
| **P3Q1** | | | | | | | | | | | | | | | | | | | | |
| *t*=base | 1013 | 0.09 (0.07) | 0.16 | -0.07 (0.02) | 0.005 | -0.04 (0.10) | 0.67 | 1025 | 0.27 (0.11) | 0.02 | -0.07 (0.02) | 0.005 | -0.04 (0.15) | 0.77 | 0.20 (0.16) | 0.20 | -0.07 (0.02) | 0.005 | 0.02 (0.20) | 0.92 |
| *t*=fu | 964 | 0.10 (0.06) | 0.12 | -0.06 (0.02) | 0.008 | 0.04 (0.09) | 0.63 | 1025 | 0.35 (0.11) | 0.001 | -0.07 (0.02) | 0.004 | 0.0009 (0.15) | 1.00 | 0.23 (0.18) | 0.19 | -0.07 (0.02) | 0.005 | -0.24 (0.26) | 0.36 |
| **P3Q2** | | | | | | | | | | | | | | | | | | | | |
| *t*=base | 1013 | 0.20 (0.06) | <0.001 | -0.05 (0.03) | 0.07 | 0.11 (0.08) | 0.19 | 1025 | 0.33 (0.08) | <0.001 | -0.05 (0.02) | 0.03 | -0.01 (0.11) | 0.92 | 0.33 (0.11) | 0.003 | -0.06 (0.03) | 0.03 | 0.06 (0.15) | 0.70 |
| *t*=fu | 964 | 0.18 (0.06) | 0.002 | -0.05 (0.03) | 0.06 | 0.11 (0.08) | 0.19 | 1025 | 0.34 (0.08) | <0.001 | -0.06 (0.02) | 0.03 | 0.10 (0.11) | 0.37 | 0.29 (0.12) | 0.02 | -0.06 (0.03) | 0.03 | 0.08 (0.16) | 0.63 |
| **Experiment Part 3: Descriptive norms (Average P3Q1 to P3Q2)** | | | | | | | | | | | | | | | | | | | | |
| *t*=base | 1013 | 0.16 (0.06) | 0.007 | -0.05 (0.02) | 0.02 | 0.04 (0.09) | 0.68 | 1025 | 0.29 (0.09) | 0.001 | -0.05 (0.02) | 0.01 | -0.02 (0.12) | 0.89 | 0.24 (0.12) | 0.05 | -0.06 (0.02) | 0.01 | 0.04 (0.15) | 0.78 |
| *t*=fu | 964 | 0.13 (0.06) | 0.02 | -0.05 (0.02) | 0.02 | 0.08 (0.08) | 0.36 | 1025 | 0.33 (0.09) | <0.001 | -0.06 (0.02) | 0.009 | 0.07 (0.12) | 0.55 | 0.21 (0.14) | 0.13 | -0.06 (0.02) | 0.01 | -0.03 (0.18) | 0.89 |
| **Donation to ASSIST/Dead Cool** | | | | | | | | | | | | | | | | | | | | |
| *t*=base | 1012 | 0.11 (0.06) | 0.06 | 0.17 (0.13) | 0.18 | -0.009 (0.10) | 0.93 | 1024 | 0.13 (0.10) | 0.22 | 0.22 (0.13) | 0.08 | -0.27 (0.15) | 0.07 | 0.32 (0.22) | 0.14 | 0.24 (0.13) | 0.07 | -0.52 (0.32) | 0.10 |
| *t*=fu | 963 | 0.35 (0.06) | <0.001 | 0.14 (0.12) | 0.26 | 0.01 (0.09) | 0.91 | 1024 | 0.52 (0.09) | <0.001 | 0.14 (0.12) | 0.26 | -0.02 (0.14) | 0.87 | 0.69 (0.15) | <0.001 | 0.16 (0.12) | 0.21 | 0.09 (0.20) | 0.65 |
| **IN1** | | | | | | | | | | | | | | | | | | | | |
| *t*=base | 1015 | -0.05 (0.06) | 0.46 | 0.09 (0.03) | 0.01 | 0.12 (0.12) | 0.32 | 1023 | 0.05 (0.14) | 0.73 | 0.09 (0.03) | 0.005 | 0.06 (0.20) | 0.75 | 0.40 (0.22) | 0.07 | 0.09 (0.03) | 0.004 | -0.51 (0.31) | 0.10 |
| *t*=fu | 1013 | 0.07 (0.06) | 0.27 | 0.08 (0.03) | 0.01 | 0.17 (0.09) | 0.07 | **1023** | **-0.21 (0.13)** | **0.11** | **0.09 (0.03)** | **0.007** | **0.58 (0.21)** | **0.006** | 0.22 (0.23) | 0.35 | 0.09 (0.03) | 0.007 | 0.43 (0.33) | 0.20 |
| **IN2** | | | | | | | | | | | | | | | | | | | | |
| *t*=base | 1017 | 0.008 (0.08) | 0.93 | 0.03 (0.02) | 0.13 | 0.20 (0.14) | 0.13 | 1025 | 0.22 (0.19) | 0.25 | 0.03 (0.02) | 0.12 | 0.18 (0.26) | 0.49 | 0.39 (0.56) | 0.49 | 0.03 (0.02) | 0.09 | 0.52 (0.54) | 0.34 |
| *t*=fu | 1015 | 0.15 (0.08) | 0.05 | 0.03 (0.02) | 0.13 | -0.12 (0.11) | 0.25 | 1025 | 0.13 (0.12) | 0.27 | 0.03 (0.02) | 0.11 | -0.16 (0.17) | 0.33 | 0.11 (0.28) | 0.69 | 0.03 (0.02) | 0.10 | -0.08 (0.35) | 0.82 |
| **IN3** | | | | | | | | | | | | | | | | | | | | |
| *t*=base | 1015 | 0.14 (0.07) | 0.03 | 0.08 (0.03) | 0.007 | -0.20 (0.12) | 0.09 | 1023 | 0.22 (0.11) | 0.05 | 0.08 (0.03) | 0.007 | 0.18 (0.16) | 0.25 | 0.29 (0.19) | 0.14 | 0.08 (0.03) | 0.007 | 0.08 (0.21) | 0.71 |
| *t*=fu | 1013 | 0.07 (0.05) | 0.20 | 0.07 (0.03) | 0.01 | -0.08 (0.08) | 0.29 | 1023 | -0.004 (0.10) | 0.97 | 0.07 (0.03) | 0.01 | 0.006 (0.15) | 0.97 | 0.16 (0.20) | 0.43 | 0.07 (0.03) | 0.01 | -0.16 (0.24) | 0.50 |
| **IN4** | | | | | | | | | | | | | | | | | | | | |
| *t*=base | 1014 | 0.03 (0.06) | 0.56 | 0.13 (0.04) | 0.001 | -0.09 (0.09) | 0.29 | 1023 | -0.04 (0.13) | 0.77 | 0.11 (0.04) | 0.003 | -0.19 (0.20) | 0.34 | 0.51 (0.32) | 0.11 | 0.12 (0.04) | 0.002 | -0.88 (0.41) | 0.03 |
| *t*=fu | 1013 | 0.05 (0.05) | 0.35 | 0.11 (0.04) | 0.003 | -0.06 (0.08) | 0.47 | 1023 | 0.09 (0.12) | 0.45 | 0.12 (0.04) | 0.002 | -0.31 (0.17) | 0.07 | 0.33 (0.23) | 0.15 | 0.11 (0.04) | 0.004 | -0.03 (0.32) | 0.93 |
| **IN5** | | | | | | | | | | | | | | | | | | | | |
| *t*=base | 1016 | -0.005 (0.06) | 0.93 | 0.03 (0.04) | 0.35 | 0.01 (0.08) | 0.91 | 1024 | -0.10 (0.11) | 0.36 | 0.03 (0.04) | 0.37 | 0.12 (0.16) | 0.44 | -0.06 (0.24) | 0.81 | 0.03 (0.04) | 0.41 | -0.29 (0.28) | 0.31 |
| *t*=fu | 1014 | 0.02 (0.06) | 0.78 | 0.04 (0.04) | 0.32 | -0.16 (0.09) | 0.07 | 1024 | -0.001 (0.12) | 0.99 | 0.04 (0.04) | 0.34 | -0.19 (0.16) | 0.23 | 0.12 (0.20) | 0.55 | 0.04 (0.04) | 0.33 | -0.53 (0.30) | 0.08 |
| **IN6** | | | | | | | | | | | | | | | | | | | | |
| *t*=base | 1016 | 0.18 (0.06) | 0.002 | 0.11 (0.04) | 0.005 | -0.15 (0.09) | 0.10 | 1024 | 0.24 (0.10) | 0.02 | 0.12 (0.04) | 0.002 | -0.16 (0.16) | 0.32 | 0.48 (0.17) | 0.004 | 0.13 (0.04) | 0.001 | -0.30 (0.23) | 0.19 |
| *t*=fu | 1014 | 0.22 (0.06) | <0.001 | 0.12 (0.04) | 0.003 | -0.12 (0.08) | 0.14 | 1024 | 0.27 (0.09) | 0.004 | 0.12 (0.04) | 0.002 | -0.06 (0.14) | 0.67 | 0.42 (0.16) | 0.01 | 0.13 (0.04) | 0.001 | -0.07 (0.22) | 0.74 |
| **IN7** | | | | | | | | | | | | | | | | | | | | |
| *t*=base | 1017 | 0.09 (0.06) | 0.12 | 0.12 (0.04) | 0.001 | -0.06 (0.08) | 0.45 | 1025 | 0.21 (0.12) | 0.10 | 0.13 (0.04) | <0.001 | 0.03 (0.18) | 0.87 | 0.59 (0.19) | 0.002 | 0.14 (0.03) | <0.001 | -0.20 (0.25) | 0.41 |
| *t*=fu | 1015 | 0.18 (0.06) | 0.005 | 0.13 (0.04) | 0.001 | -0.02 (0.10) | 0.81 | 1025 | 0.24 (0.11) | 0.03 | 0.14 (0.04) | <0.001 | -0.009 (0.15) | 0.96 | 0.37 (0.18) | 0.05 | 0.14 (0.04) | <0.001 | -0.04 (0.22) | 0.85 |
| **Self-report injunctive norms (Average IN1 to IN7)** | | | | | | | | | | | | | | | | | | | | |
| *t*=base | 1011 | 0.07 (0.06) | 0.21 | 0.08 (0.02) | <0.001 | -0.07 (0.09) | 0.44 | 1020 | 0.06 (0.12) | 0.59 | 0.07 (0.02) | <0.001 | -0.01 (0.14) | 0.92 | 0.46 (0.18) | 0.01 | 0.08 (0.02) | <0.001 | -0.47 (0.21) | 0.02 |
| *t*=fu | 1010 | 0.18 (0.05) | 0.001 | 0.07 (0.02) | <0.001 | -0.14 (0.08) | 0.06 | 1020 | 0.16 (0.09) | 0.08 | 0.07 (0.02) | <0.001 | -0.19 (0.11) | 0.09 | 0.30 (0.16) | 0.06 | 0.08 (0.02) | <0.001 | -0.46 (0.19) | 0.02 |
| **DN1.1** | | | | | | | | | | | | | | | | | | | | |
| *t*=base | 1017 | 0.23 (0.11) | 0.04 | 0.04 (0.04) | 0.28 | -0.23 (0.18) | 0.20 | 1025 | 0.37 (0.17) | 0.03 | 0.03 (0.03) | 0.32 | 0.09 (0.26) | 0.74 | 0.97 (0.33) | 0.004 | 0.03 (0.04) | 0.41 | 0.12 (0.48) | 0.80 |
| *t*=fu | 1015 | 0.37 (0.10) | <0.001 | 0.03 (0.04) | 0.35 | 0.08 (0.18) | 0.65 | 1025 | 0.31 (0.15) | 0.04 | 0.03 (0.04) | 0.37 | 0.20 (0.27) | 0.46 | 0.59 (0.20) | 0.003 | 0.03 (0.04) | 0.37 | 0.12 (0.37) | 0.74 |
| **DN1.2** | | | | | | | | | | | | | | | | | | | | |
| *t*=base | 1018 | 0.14 (0.05) | 0.008 | 0.01 (0.03) | 0.66 | 0.05 (0.07) | 0.50 | 1026 | 0.10 (0.07) | 0.16 | 0.02 (0.03) | 0.61 | 0.12 (0.10) | 0.21 | 0.18 (0.09) | 0.06 | 0.02 (0.03) | 0.56 | 0.06 (0.11) | 0.63 |
| *t*=fu | 1016 | 0.01 (0.04) | 0.81 | 0.02 (0.03) | 0.47 | 0.10 (0.07) | 0.18 | 1026 | 0.05 (0.08) | 0.53 | 0.02 (0.03) | 0.62 | 0.16 (0.12) | 0.19 | 0.11 (0.10) | 0.25 | 0.03 (0.03) | 0.43 | 0.02 (0.13) | 0.85 |
| **DN1.3** | | | | | | | | | | | | | | | | | | | | |
| *t*=base | 1017 | 0.08 (0.05) | 0.08 | 0.04 (0.04) | 0.36 | -0.07 (0.07) | 0.34 | 1025 | 0.06 (0.08) | 0.48 | 0.03 (0.04) | 0.46 | 0.08 (0.13) | 0.57 | 0.12 (0.13) | 0.38 | 0.03 (0.04) | 0.51 | 0.09 (0.15) | 0.57 |
| *t*=fu | 1015 | 0.03 (0.05) | 0.61 | 0.04 (0.04) | 0.36 | -0.02 (0.06) | 0.79 | 1025 | 0.03 (0.08) | 0.69 | 0.03 (0.04) | 0.40 | 0.06 (0.12) | 0.64 | 0.07 (0.15) | 0.66 | 0.03 (0.04) | 0.43 | 0.05 (0.17) | 0.77 |
| **DN1.4** | | | | | | | | | | | | | | | | | | | | |
| *t*=base | 1018 | 0.01 (0.05) | 0.82 | 0.003 (0.03) | 0.90 | -0.02 (0.07) | 0.70 | 1026 | 0.07 (0.14) | 0.62 | 0.01 (0.03) | 0.70 | -0.11 (0.14) | 0.40 | 0.12 (0.15) | 0.40 | 0.005 (0.03) | 0.85 | 0.12 (0.20) | 0.55 |
| *t*=fu | 1016 | -0.06 (0.04) | 0.13 | 0.005 (0.03) | 0.86 | 0.03 (0.05) | 0.55 | 1026 | 0.09 (0.16) | 0.60 | 0.008 (0.03) | 0.76 | -0.08 (0.13) | 0.56 | -0.12 (0.18) | 0.49 | 0.004 (0.03) | 0.87 | 0.22 (0.22) | 0.30 |
| **DN1.5** | | | | | | | | | | | | | | | | | | | | |
| *t*=base | 1018 | 0.07 (0.05) | 0.16 | 0.02 (0.02) | 0.14 | -0.13 (0.07) | 0.09 | 1026 | 0.19 (0.11) | 0.10 | 0.02 (0.02) | 0.17 | -0.31 (0.18) | 0.09 | 0.37 (0.20) | 0.06 | 0.02 (0.02) | 0.22 | -0.14 (0.25) | 0.58 |
| *t*=fu | 1016 | -0.02 (0.05) | 0.73 | 0.02 (0.01) | 0.18 | -0.04 (0.06) | 0.53 | 1026 | 0.22 (0.11) | 0.05 | 0.02 (0.02) | 0.21 | -0.24 (0.17) | 0.16 | 0.14 (0.17) | 0.39 | 0.02 (0.02) | 0.23 | -0.04 (0.17) | 0.81 |
| **Self-report descriptive norms 1 (Average DN1.1 to DN1.5)** | | | | | | | | | | | | | | | | | | | | |
| *t*=base | 1016 | 0.09 (0.05) | 0.08 | 0.01 (0.02) | 0.47 | -0.03 (0.08) | 0.74 | 1024 | 0.09 (0.08) | 0.22 | 0.01 (0.02) | 0.52 | 0.09 (0.11) | 0.39 | 0.19 (0.11) | 0.08 | 0.01 (0.02) | 0.48 | 0.03 (0.12) | 0.78 |
| *t*=fu | 1014 | 0.04 (0.05) | 0.44 | 0.01 (0.02) | 0.41 | 0.02 (0.07) | 0.74 | 1024 | 0.06 (0.08) | 0.42 | 0.009 (0.02) | 0.59 | 0.14 (0.11) | 0.20 | 0.15 (0.11) | 0.15 | 0.01 (0.02) | 0.50 | 0.07 (0.12) | 0.55 |
| **DN2.1** | | | | | | | | | | | | | | | | | | | | |
| *t*=base | 1018 | 0.15 (0.07) | 0.03 | 0.01 (0.03) | 0.75 | 0.05 (0.12) | 0.65 | 1026 | 0.21 (0.11) | 0.07 | 0.01 (0.03) | 0.61 | 0.06 (0.18) | 0.72 | 0.22 (0.15) | 0.15 | 0.02 (0.03) | 0.56 | -0.04 (0.22) | 0.86 |
| *t*=fu | 1016 | 0.20 (0.07) | 0.003 | 0.02 (0.03) | 0.51 | 0.02 (0.09) | 0.87 | 1026 | 0.20 (0.12) | 0.11 | 0.02 (0.03) | 0.55 | 0.02 (0.16) | 0.90 | 0.12 (0.17) | 0.47 | 0.02 (0.03) | 0.57 | 0.02 (0.22) | 0.91 |
| **DN2.2** | | | | | | | | | | | | | | | | | | | | |
| *t*=base | 1018 | 0.17 (0.07) | 0.008 | 0.03 (0.04) | 0.50 | 0.10 (0.10) | 0.30 | 1026 | 0.32 (0.10) | 0.001 | 0.04 (0.04) | 0.38 | -0.02 (0.13) | 0.88 | 0.55 (0.17) | 0.001 | 0.01 (0.04) | 0.75 | 0.24 (0.22) | 0.28 |
| *t*=fu | 1016 | 0.09 (0.06) | 0.12 | 0.04 (0.04) | 0.27 | -0.07 (0.08) | 0.38 | 1026 | 0.19 (0.10) | 0.04 | 0.04 (0.04) | 0.29 | -0.02 (0.12) | 0.89 | 0.44 (0.16) | 0.006 | 0.02 (0.04) | 0.56 | 0.20 (0.21) | 0.34 |
| **DN2.3** | | | | | | | | | | | | | | | | | | | | |
| *t*=base | 1018 | 0.25 (0.07) | 0.001 | 0.03 (0.03) | 0.25 | -0.21 (0.11) | 0.06 | 1026 | 0.56 (0.10) | <0.001 | 0.02 (0.03) | 0.39 | -0.15 (0.15) | 0.31 | 0.63 (0.13) | <0.001 | 0.03 (0.03) | 0.28 | -0.16 (0.20) | 0.43 |
| *t*=fu | 1016 | 0.17 (0.06) | 0.002 | 0.03 (0.03) | 0.25 | -0.04 (0.11) | 0.71 | 1026 | 0.38 (0.09) | <0.001 | 0.03 (0.03) | 0.34 | -0.13 (0.13) | 0.30 | 0.52 (0.12) | <0.001 | 0.03 (0.03) | 0.31 | -0.12 (0.17) | 0.47 |
| **Self-report descriptive norms 2 (Average DN2.1 to DN2.3)** | | | | | | | | | | | | | | | | | | | | |
| *t*=base | 1018 | 0.20 (0.06) | 0.002 | 0.01 (0.02) | 0.63 | 0.008 (0.10) | 0.94 | 1026 | 0.38 (0.10) | <0.001 | 0.01 (0.02) | 0.56 | -0.07 (0.14) | 0.62 | 0.44 (0.14) | 0.001 | 0.01 (0.02) | 0.65 | 0.05 (0.21) | 0.83 |
| *t*=fu | 1016 | 0.11 (0.06) | 0.05 | 0.01 (0.02) | 0.50 | 0.06 (0.08) | 0.41 | 1026 | 0.27 (0.09) | 0.002 | 0.02 (0.02) | 0.48 | -0.007 (0.12) | 0.96 | 0.37 (0.13) | 0.004 | 0.01 (0.02) | 0.60 | 0.06 (0.19) | 0.75 |
| **Self-report smoking behavior** | | | | | | | | | | | | | | | | | | | | |
| *t*=base | 1021 | 0.11 (0.07) | 0.09 | 0.09 (0.02) | <0.001 | -0.11 (0.10) | 0.24 | 1029 | 0.27 (0.11) | 0.01 | 0.09 (0.02) | <0.001 | -0.26 (0.14) | 0.05 | **0.63 (0.16)** | **<0.001** | **0.09 (0.02)** | **<0.001** | **-0.50 (0.18)** | **0.006** |
| *t*=fu | 1019 | 0.22 (0.06) | <0.001 | 0.09 (0.02) | <0.001 | -0.22 (0.09) | 0.02 | 1029 | 0.23 (0.09) | 0.006 | 0.09 (0.02) | <0.001 | -0.12 (0.10) | 0.25 | **0.44 (0.10)** | **<0.001** | **0.09 (0.02)** | **<0.001** | **-0.34 (0.13)** | **0.01** |
| **Intentions** | | | | | | | | | | | | | | | | | | | | |
| *t*=base | 1017 | 0.13 (0.09) | 0.14 | 0.20 (0.05) | <0.001 | 0.04 (0.11) | 0.74 | 1025 | 0.49 (0.14) | 0.001 | 0.21 (0.05) | <0.001 | -0.19 (0.20) | 0.34 | 0.94 (0.26) | <0.001 | 0.21 (0.05) | <0.001 | -0.24 (0.36) | 0.51 |
| *t*=fu | 1015 | 0.21 (0.08) | 0.008 | 0.21 (0.05) | <0.001 | -0.10 (0.10) | 0.28 | 1025 | 0.09 (0.12) | 0.43 | 0.21 (0.05) | <0.001 | 0.02 (0.16) | 0.90 | 0.38 (0.15) | 0.01 | 0.22 (0.05) | <0.001 | 0.02 (0.20) | 0.94 |
| **Knowledge** | | | | | | | | | | | | | | | | | | | | |
| *t*=base | 1020 | 0.20 (0.06) | 0.001 | 0.06 (0.06) | 0.38 | -0.06 (0.08) | 0.44 | 1028 | 0.41 (0.08) | <0.001 | 0.06 (0.06) | 0.35 | -0.19 (0.11) | 0.10 | 0.44 (0.11) | <0.001 | 0.07 (0.06) | 0.27 | -0.09 (0.14) | 0.52 |
| *t*=fu | 1018 | 0.28 (0.06) | <0.001 | 0.08 (0.06) | 0.21 | -0.08 (0.08) | 0.31 | 1028 | 0.41 (0.08) | <0.001 | 0.06 (0.06) | 0.33 | -0.12 (0.10) | 0.23 | 0.41 (0.12) | <0.001 | 0.07 (0.06) | 0.28 | -0.10 (0.13) | 0.44 |
| **Attitudes** | | | | | | | | | | | | | | | | | | | | |
| *t*=base | 1010 | 0.15 (0.06) | 0.01 | 0.08 (0.03) | 0.01 | 0.01 (0.09) | 0.90 | 1018 | -0.05 (0.11) | 0.66 | 0.08 (0.03) | 0.004 | -0.27 (0.16) | 0.08 | 0.37 (0.22) | 0.09 | 0.08 (0.03) | 0.004 | -0.43 (0.30) | 0.15 |
| *t*=fu | 1008 | 0.15 (0.07) | 0.02 | 0.08 (0.03) | 0.009 | -0.15 (0.09) | 0.12 | 1018 | -0.11 (0.12) | 0.36 | 0.08 (0.03) | 0.006 | -0.10 (0.16) | 0.56 | 0.24 (0.17) | 0.16 | 0.08 (0.03) | 0.005 | -0.45 (0.23) | 0.05 |
| **Self-efficacy (Emotional)** | | | | | | | | | | | | | | | | | | | | |
| *t*=base | 1012 | 0.08 (0.08) | 0.31 | 0.13 (0.04) | 0.001 | 0.06 (0.11) | 0.57 | 1020 | 0.29 (0.12) | 0.02 | 0.13 (0.04) | <0.001 | -0.04 (0.16) | 0.82 | 0.42 (0.17) | 0.01 | 0.14 (0.04) | <0.001 | -0.25 (0.22) | 0.25 |
| *t*=fu | 1009 | 0.26 (0.07) | <0.001 | 0.13 (0.04) | <0.001 | -0.07 (0.09) | 0.46 | 1020 | 0.24 (0.09) | 0.01 | 0.14 (0.04) | <0.001 | -0.08 (0.11) | 0.49 | 0.39 (0.14) | 0.005 | 0.14 (0.04) | <0.001 | -0.20 (0.15) | 0.18 |
| **Self-efficacy (Friends)** | | | | | | | | | | | | | | | | | | | | |
| *t*=base | 1017 | 0.03 (0.06) | 0.63 | 0.14 (0.04) | <0.001 | -0.02 (0.09) | 0.86 | 1025 | 0.22 (0.12) | 0.06 | 0.14 (0.04) | <0.001 | -0.09 (0.17) | 0.58 | 0.19 (0.18) | 0.28 | 0.14 (0.04) | <0.001 | -0.31 (0.23) | 0.17 |
| *t*=fu | 1015 | 0.18 (0.07) | 0.008 | 0.14 (0.04) | <0.001 | -0.07 (0.10) | 0.53 | 1025 | 0.10 (0.10) | 0.31 | 0.14 (0.04) | <0.001 | -0.09 (0.14) | 0.52 | 0.27 (0.19) | 0.16 | 0.14 (0.04) | <0.001 | -0.29 (0.23) | 0.20 |
| **Self-efficacy (Opportunity)** | | | | | | | | | | | | | | | | | | | | |
| *t*=base | 1018 | 0.10 (0.07) | 0.17 | 0.11 (0.03) | 0.001 | 0.01 (0.10) | 0.92 | 1026 | 0.41 (0.15) | 0.007 | 0.11 (0.03) | <0.001 | 0.02 (0.21) | 0.94 | 0.41 (0.21) | 0.05 | 0.12 (0.03) | <0.001 | -0.18 (0.25) | 0.48 |
| *t*=fu | 1016 | 0.14 (0.07) | 0.06 | 0.11 (0.03) | <0.001 | -0.04 (0.10) | 0.67 | 1026 | 0.23 (0.10) | 0.03 | 0.12 (0.03) | <0.001 | -0.18 (0.13) | 0.16 | 0.31 (0.18) | 0.08 | 0.12 (0.03) | <0.001 | -0.18 (0.20) | 0.36 |
| **Perceived physical risks** | | | | | | | | | | | | | | | | | | | | |
| *t*=base | 1015 | 0.08 (0.05) | 0.15 | 1.72 (1.00) | 0.09 | 0.03 (0.07) | 0.64 | 1023 | 0.19 (0.09) | 0.04 | 1.48 (1.01) | 0.14 | 0.04 (0.12) | 0.77 | 0.13 (0.15) | 0.40 | 1.67 (1.00) | 0.10 | 0.003 (0.19) | 0.99 |
| *t*=fu | 1013 | 0.12 (0.06) | 0.03 | 1.57 (1.01) | 0.12 | -0.005 (0.07) | 0.94 | 1023 | 0.13 (0.11) | 0.25 | 1.56 (1.01) | 0.12 | 0.09 (0.13) | 0.49 | -0.05 (0.22) | 0.81 | 1.63 (1.01) | 0.11 | -0.09 (0.21) | 0.67 |
| **Perceived social risks** | | | | | | | | | | | | | | | | | | | | |
| *t*=base | 1015 | 0.15 (0.05) | 0.004 | 1.26 (1.09) | 0.25 | 0.009 (0.07) | 0.89 | 1023 | 0.27 (0.07) | <0.001 | 1.20 (1.09) | 0.27 | 0.04 (0.09) | 0.64 | 0.40 (0.10) | <0.001 | 1.63 (1.07) | 0.13 | -0.05 (0.12) | 0.70 |
| *t*=fu | 1013 | 0.13 (0.06) | 0.02 | 1.17 (1.10) | 0.29 | -0.009 (0.08) | 0.91 | 1023 | 0.22 (0.08) | 0.01 | 1.27 (1.09) | 0.24 | 0.10 (0.11) | 0.37 | 0.40 (0.12) | 0.001 | 1.54 (1.08) | 0.15 | -0.07 (0.15) | 0.65 |
| **Perceived addiction risks** | | | | | | | | | | | | | | | | | | | | |
| *t*=base | 963 | 0.22 (0.06) | <0.001 | 0.01 (1.19) | 0.99 | 0.13 (0.08) | 0.11 | 971 | 0.51 (0.09) | <0.001 | 0.06 (1.18) | 0.96 | 0.08 (0.12) | 0.50 | 0.73 (0.11) | <0.001 | 0.04 (1.17) | 0.97 | 0.06 (0.14) | 0.65 |
| *t*=fu | 957 | 0.15 (0.05) | 0.003 | -0.23 (1.20) | 0.85 | -0.03 (0.07) | 0.67 | 971 | 0.41 (0.08) | <0.001 | -0.05 (1.18) | 0.96 | 0.13 (0.11) | 0.21 | 0.60 (0.09) | <0.001 | -0.04 (1.17) | 0.97 | 0.06 (0.12) | 0.63 |
| **Perceived benefits** | | | | | | | | | | | | | | | | | | | | |
| *t*=base | 980 | -0.04 (0.06) | 0.51 | -0.41 (0.98) | 0.68 | 0.09 (0.10) | 0.38 | 988 | 0.06 (0.13) | 0.62 | -0.33 (0.97) | 0.74 | -0.007 (0.18) | 0.97 | 0.32 (0.21) | 0.14 | -0.29 (0.98) | 0.77 | 0.09 (0.31) | 0.78 |
| *t*=fu | 977 | 0.01 (0.06) | 0.84 | -0.42 (0.98) | 0.67 | 0.06 (0.08) | 0.44 | 988 | -0.09 (0.13) | 0.50 | -0.37 (0.97) | 0.70 | 0.03 (0.19) | 0.87 | -0.13 (0.26) | 0.61 | -0.40 (0.98) | 0.69 | -0.14 (0.40) | 0.72 |
| **Perceived behavioral control (easy to quit)** | | | | | | | | | | | | | | | | | | | | |
| *t*=base | 1017 | 0.29 (0.06) | <0.001 | 0.05 (0.06) | 0.42 | 0.06 (0.08) | 0.43 | 1025 | 0.57 (0.08) | <0.001 | 0.03 (0.06) | 0.60 | 0.16 (0.10) | 0.13 | 0.74 (0.10) | <0.001 | 0.03 (0.06) | 0.61 | 0.09 (0.12) | 0.45 |
| *t*=fu | 1015 | 0.18 (0.06) | 0.003 | 0.06 (0.07) | 0.37 | 0.02 (0.08) | 0.85 | 1025 | 0.52 (0.08) | <0.001 | 0.03 (0.06) | 0.59 | 0.04 (0.10) | 0.67 | 0.68 (0.10) | <0.001 | 0.02 (0.06) | 0.73 | 0.12 (0.12) | 0.33 |
| **Perceived behavioral control (to avoid smoking)** | | | | | | | | | | | | | | | | | | | | |
| *t*=base | 1019 | 0.06 (0.07) | 0.43 | 0.13 (0.05) | 0.02 | -0.04 (0.10) | 0.68 | 1027 | 0.42 (0.14) | 0.002 | 0.15 (0.05) | 0.006 | -0.04 (0.18) | 0.85 | 0.87 (0.22) | <0.001 | 0.16 (0.05) | 0.002 | 0.18 (0.30) | 0.56 |
| *t*=fu | 1017 | 0.09 (0.07) | 0.21 | 0.12 (0.05) | 0.02 | -0.15 (0.11) | 0.17 | 1027 | 0.17 (0.14) | 0.21 | 0.13 (0.05) | 0.01 | 0.21 (0.19) | 0.26 | 0.58 (0.18) | 0.001 | 0.15 (0.05) | 0.005 | 0.29 (0.23) | 0.20 |
| **Objectively measured smoking behavior** | | | | | | | | | | | | | | | | | | | | |
| *t*=base | 983 | 0.35 (0.06) | <0.001 | 0.02 (0.08) | 0.78 | -0.02 (0.06) | 0.72 | 989 | 0.37 (0.06) | <0.001 | 0.007 (0.08) | 0.93 | -0.04 (0.06) | 0.45 | 0.56 (0.06) | <0.001 | -0.02 (0.07) | 0.80 | -0.05 (0.06) | 0.44 |
| *t*=fu | 965 | 0.47 (0.08) | <0.001 | 0.002 (0.07) | 0.98 | -0.12 (0.09) | 0.17 | 989 | 0.83 (0.06) | <0.001 | -0.03 (0.06) | 0.64 | -0.02 (0.07) | 0.76 | 0.85 (0.06) | <0.001 | -0.006 (0.06) | 0.93 | -0.04 (0.08) | 0.65 |
| **Smoking susceptibility^e^** | | | | | | | | | | | | | | | | | | | | |
|  | n | **Percent_-i,t_^f^** | | **M^g^** | | **M*Percent_-i,t_^h^** | | n | **Percent_-i,t_^f^** | | **M^g^** | | **M*Percent_-i,t_^h^** | | **Percent_-i,t_^f^** | | **M^g^** | | **M*Percent_-i,t_^h^** | |
|  |  | OR (SE) | p-value | OR (SE) | p-value | OR (SE) | p-value |  | OR (SE) | p-value | OR (SE) | p-value | OR (SE) | p-value | OR (SE) | p-value | OR (SE) | p-value | OR (SE) | p-value |
| *t*=base | 1017 | 1.12 (0.04) | 0.001 | 0.64 (0.08) | <0.001 | 0.99 (0.05) | 0.81 | 1025 | 1.06 (0.07) | 0.31 | 0.61 (0.07) | <0.001 | 1.08 (0.11) | 0.47 | 1.19 (0.12) | 0.08 | 0.60 (0.07) | <0.001 | 1.03 (0.14) | 0.84 |
| *t*=fu | 1015 | 1.12 (0.03) | <0.001 | 0.61 (0.07) | <0.001 | 0.92 (0.04) | 0.07 | 1025 | 1.18 (0.07) | 0.002 | 0.59 (0.07) | <0.001 | 1.02 (0.08) | 0.77 | 1.31 (0.10) | <0.001 | 0.58 (0.07) | <0.001 | 0.94 (0.10) | 0.56 |

IV: Independent variable; DV: Dependent variable; Ave: average of peer group (*i*); M: moderator ('Big Five' agreeableness: 0 [least agreeable] to 4 [most agreeable]); Percent: percentage of peer group (*-i*) classified as susceptible to commencing smoking; OR: odds ratio; SE: standard error.

^a^In each model the outcome variable is the focal participant’s (*i*) response to the relevant item at follow-up. The predictor variable is the average of the relevant group’s (*-i*) responses to the equivalent item at baseline (*t*=base) or follow-up (*t*=fu), where *–i*=(1) focal participant's nominated friends; (2) focal participant’s school class; (3) focal participant’s school year group. The moderator, and interaction of the moderator with the predictor variable, were also included as independent variables in all models. All models include robust (Huber White) standard errors specified using Stata’s ‘vce(robust)’ option. The following baseline variables are included as covariates in all models: gender (0=boy; 1=girl/prefer not to say), age (1=12 years or less; 2=13 years; 3=14 years or more), intervention (1=ASSIST; 2=Dead Cool), ethnicity (0=no ethnic minority; 1=ethnic minority), individuals' socio-economic status (NI: 1=NIMDM2017≤296.6; 2=296.6<NIMDM2017≤593.2; 3=NIMDM2017>593.2; Bogotá: 1=Informal settlement/Lowest/Low; 2=Middle-Low/Middle; 3=Middle-High/High), and baseline values of the outcome variable. The predictor variable, moderator variable, and baseline values of the outcome variable were mean-centered.

^b^Unstandardized regression coefficients representing the average change in the outcome variable for a one-unit increase in the predictor variable among participants who are average on agreeableness.

^c^Unstandardized regression coefficients representing the average change in the outcome variable for a one-unit increase in agreeableness among participants who are average on the predictor variable.

^d^Unstandardized regression coefficients representing the average change in the association between the outcome variable and the predictor variable for a one-unit increase in agreeableness.

^e^Logistic regressions were run for models including focal participants' smoking susceptibility as the outcome variable, with robust (Huber White) standard errors specified using Stata’s ‘vce(robust)’ option. The predictor variable is the percentage of the relevant group (*-i*) classified as susceptible to commencing smoking at baseline (*t*=base) or follow-up (*t*=fu), where *–i*=(1) focal participant's nominated friends; (2) focal participant’s school class; (3) focal participant’s school year group. The moderator, and interaction of the moderator with the predictor variable, were also included as independent variables in all models. The following baseline variables are included as covariates in all models: gender (0=boy; 1=girl/prefer not to say), age (1=12 years or less; 2=13 years; 3=14 years or more), intervention (1=ASSIST; 2=Dead Cool), ethnicity (0=no ethnic minority; 1=ethnic minority), individuals' socio-economic status (NI: 1=NIMDM2017≤296.6; 2=296.6<NIMDM2017≤593.2; 3=NIMDM2017>593.2; Bogotá: 1=Informal settlement/Lowest/Low; 2=Middle-Low/Middle; 3=Middle-High/High), and baseline values of the outcome variable. Results are odds ratios, standard errors, and p-values. The predictor variable, and moderator variable were mean-centered.

^f^Odds ratios representing the multiplicative change in odds of being susceptible to commencing smoking for a 10% increase in the number of nominated friends/pupils in the same school class/pupils in the same school year group classified as being susceptible to commencing smoking (1 out of 10 nominated friends/pupils in the same school class/pupils in the same school year group; predictor variable) among participants who are average on agreeableness.

^g^Odds ratios representing the multiplicative change in odds of being susceptible to commencing smoking for a one-unit increase in agreeableness among participants who are average on the predictor variable.

^h^Ratio of ratios representing the ratio of: (1) the odds ratio representing the multiplicative change in odds of being susceptible to commencing smoking for a 10% increase in the number of nominated friends/pupils in the same school class/pupils in the same school year group classified as being susceptible to commencing smoking among participants who are one unit above average on agreeableness; to (2) the odds ratio representing the multiplicative change in odds of being susceptible to commencing smoking for a 10% increase in the number of nominated friends/pupils in the same school class/pupils in the same school year group classified as being susceptible to commencing smoking among participants who are average on agreeableness.

**Table S2.14.** Results of ordinary least squares linear regressions including interaction terms examining differences in peer influence effects according to conscientiousness for outcomes collected at follow-up.

|  | **Dependent variable: Participant responses to the outcome variable at follow-up^a^** | | | | | | | | | | | | | | | | | | | |
| --- | --- | --- | --- | --- | --- | --- | --- | --- | --- | --- | --- | --- | --- | --- | --- | --- | --- | --- | --- | --- |
|  | **(1) -i=Average of nominated friends** | | | | | | | n | **(2) -i=Average of school class** | | | | | | **(3) -i=Average of school year group** | | | | | |
| **IV** | n | **Ave_-i,t_^b^** | | **M^c^** | | **M*Ave_-i,t_^d^** | |  | **Ave_-i,t_^b^** | | **M^c^** | | **M*Ave_-i,t_^d^** | | **Ave_-i,t_^b^** | | **M^c^** | | **M*Ave_-i,t_^d^** | |
| **DV** |  | *b* (SE) | p-value | *b* (SE) | p-value | *b* (SE) | p-value |  | *b* (SE) | p-value | *b* (SE) | p-value | *b* (SE) | p-value | *b* (SE) | p-value | *b* (SE) | p-value | *b* (SE) | p-value |
| **P2S2** | | | | | | | | | | | | | | | | | | | | |
| *t*=base | 1009 | 0.35 (0.09) | <0.001 | -0.04 (0.01) | 0.002 | -0.04 (0.12) | 0.77 | 1021 | 0.44 (0.12) | <0.001 | -0.04 (0.01) | 0.002 | -0.09 (0.17) | 0.60 | 0.83 (0.19) | <0.001 | -0.04 (0.01) | 0.003 | -0.22 (0.25) | 0.38 |
| *t*=fu | 961 | 0.12 (0.06) | 0.05 | -0.04 (0.01) | 0.001 | -0.17 (0.10) | 0.10 | 1021 | 0.30 (0.10) | 0.004 | -0.04 (0.01) | 0.002 | -0.17 (0.14) | 0.25 | 0.62 (0.17) | <0.001 | -0.04 (0.01) | 0.002 | -0.35 (0.24) | 0.15 |
| **P2S3** | | | | | | | | | | | | | | | | | | | | |
| *t*=base | 1009 | 0.09 (0.07) | 0.21 | -0.07 (0.01) | <0.001 | -0.13 (0.09) | 0.18 | 1021 | 0.15 (0.16) | 0.33 | -0.07 (0.01) | <0.001 | -0.31 (0.22) | 0.16 | 0.09 (0.28) | 0.75 | -0.08 (0.01) | <0.001 | -0.76 (0.37) | 0.04 |
| *t*=fu | 961 | 0.09 (0.07) | 0.17 | -0.07 (0.01) | <0.001 | -0.15 (0.09) | 0.08 | 1021 | 0.18 (0.14) | 0.20 | -0.07 (0.01) | <0.001 | -0.30 (0.19) | 0.13 | 0.05 (0.28) | 0.87 | -0.07 (0.01) | <0.001 | -0.66 (0.36) | 0.07 |
| **P2S4** | | | | | | | | | | | | | | | | | | | | |
| *t*=base | 1006 | 0.10 (0.08) | 0.22 | -0.03 (0.02) | 0.04 | -0.01 (0.12) | 0.90 | 1018 | 0.13 (0.12) | 0.29 | -0.03 (0.01) | 0.03 | 0.07 (0.15) | 0.65 | 0.55 (0.33) | 0.09 | -0.03 (0.01) | 0.04 | 0.22 (0.30) | 0.47 |
| *t*=fu | 959 | 0.12 (0.07) | 0.09 | -0.03 (0.02) | 0.05 | -0.08 (0.08) | 0.36 | 1018 | 0.26 (0.12) | 0.03 | -0.03 (0.01) | 0.03 | 0.02 (0.14) | 0.90 | 0.47 (0.21) | 0.03 | -0.03 (0.01) | 0.02 | -0.10 (0.22) | 0.64 |
| **P2S5** | | | | | | | | | | | | | | | | | | | | |
| *t*=base | 1009 | 0.20 (0.06) | 0.002 | -0.04 (0.02) | 0.04 | -0.13 (0.10) | 0.19 | 1021 | 0.36 (0.10) | 0.001 | -0.04 (0.02) | 0.03 | -0.11 (0.15) | 0.48 | 0.53 (0.15) | 0.001 | -0.04 (0.02) | 0.03 | 0.10 (0.22) | 0.65 |
| *t*=fu | 961 | 0.19 (0.06) | 0.002 | -0.05 (0.02) | 0.009 | 0.03 (0.09) | 0.71 | 1021 | 0.49 (0.09) | <0.001 | -0.04 (0.02) | 0.05 | 0.04 (0.13) | 0.76 | 0.50 (0.15) | 0.001 | -0.04 (0.02) | 0.02 | -0.06 (0.19) | 0.74 |
| **P2S6** | | | | | | | | | | | | | | | | | | | | |
| *t*=base | 1006 | 0.14 (0.07) | 0.03 | -0.05 (0.02) | 0.02 | -0.06 (0.11) | 0.60 | 1018 | 0.38 (0.11) | 0.001 | -0.04 (0.02) | 0.03 | 0.02 (0.16) | 0.89 | 0.48 (0.19) | 0.01 | -0.04 (0.02) | 0.03 | 0.49 (0.25) | 0.05 |
| *t*=fu | 958 | 0.14 (0.06) | 0.01 | -0.05 (0.02) | 0.01 | 0.04 (0.09) | 0.67 | 1018 | 0.41 (0.10) | <0.001 | -0.04 (0.02) | 0.04 | 0.03 (0.15) | 0.82 | 0.50 (0.15) | 0.001 | -0.04 (0.02) | 0.04 | 0.26 (0.21) | 0.21 |
| **P2S7** | | | | | | | | | | | | | | | | | | | | |
| *t*=base | 1009 | 0.15 (0.06) | 0.008 | -0.06 (0.02) | 0.002 | -0.07 (0.09) | 0.41 | 1021 | 0.25 (0.09) | 0.005 | -0.06 (0.02) | 0.002 | -0.17 (0.13) | 0.20 | 0.31 (0.14) | 0.03 | -0.06 (0.02) | 0.001 | -0.11 (0.19) | 0.56 |
| *t*=fu | 961 | 0.27 (0.06) | <0.001 | -0.06 (0.02) | 0.001 | -0.15 (0.10) | 0.12 | 1021 | 0.36 (0.09) | <0.001 | -0.06 (0.02) | 0.002 | -0.10 (0.14) | 0.48 | 0.32 (0.17) | 0.06 | -0.06 (0.02) | 0.003 | 0.12 (0.22) | 0.57 |
| **P2S8** | | | | | | | | | | | | | | | | | | | | |
| *t*=base | 1009 | 0.30 (0.05) | <0.001 | -0.07 (0.02) | <0.001 | -0.04 (0.08) | 0.59 | 1021 | 0.29 (0.09) | 0.001 | -0.07 (0.02) | <0.001 | -0.20 (0.13) | 0.14 | 0.53 (0.14) | <0.001 | -0.08 (0.02) | <0.001 | -0.19 (0.21) | 0.36 |
| *t*=fu | 961 | 0.24 (0.06) | <0.001 | -0.08 (0.02) | <0.001 | -0.13 (0.08) | 0.13 | 1021 | 0.46 (0.08) | <0.001 | -0.07 (0.02) | <0.001 | -0.12 (0.12) | 0.33 | 0.53 (0.14) | <0.001 | -0.08 (0.02) | <0.001 | -0.06 (0.19) | 0.73 |
| **P2S9** | | | | | | | | | | | | | | | | | | | | |
| *t*=base | 1009 | 0.14 (0.07) | 0.03 | -0.09 (0.02) | <0.001 | -0.13 (0.11) | 0.22 | 1021 | 0.45 (0.12) | <0.001 | -0.09 (0.02) | <0.001 | -0.39 (0.22) | 0.07 | 0.47 (0.22) | 0.03 | -0.09 (0.02) | <0.001 | -0.21 (0.35) | 0.55 |
| *t*=fu | 961 | 0.22 (0.06) | 0.001 | -0.09 (0.02) | <0.001 | -0.19 (0.10) | 0.05 | 1021 | 0.33 (0.11) | 0.002 | -0.09 (0.02) | <0.001 | -0.26 (0.17) | 0.13 | 0.19 (0.21) | 0.36 | -0.09 (0.02) | <0.001 | -0.16 (0.31) | 0.61 |
| **Experiment Part 2: Injunctive norms (Average P2S2 to P2S9)** | | | | | | | | | | | | | | | | | | | | |
| *t*=base | 1003 | 0.20 (0.06) | <0.001 | -0.05 (0.01) | <0.001 | -0.12 (0.09) | 0.18 | 1015 | 0.32 (0.10) | 0.001 | -0.04 (0.01) | <0.001 | -0.13 (0.16) | 0.40 | 0.46 (0.15) | 0.002 | -0.05 (0.01) | <0.001 | 0.18 (0.22) | 0.42 |
| *t*=fu | 956 | 0.24 (0.05) | <0.001 | -0.05 (0.01) | <0.001 | -0.09 (0.08) | 0.28 | 1015 | 0.45 (0.08) | <0.001 | -0.04 (0.01) | <0.001 | -0.05 (0.12) | 0.69 | 0.34 (0.14) | 0.02 | -0.05 (0.01) | <0.001 | 0.13 (0.20) | 0.51 |
| **P3Q1** | | | | | | | | | | | | | | | | | | | | |
| *t*=base | 1009 | 0.10 (0.07) | 0.15 | -0.11 (0.02) | <0.001 | -0.06 (0.10) | 0.54 | 1021 | 0.27 (0.11) | 0.01 | -0.11 (0.02) | <0.001 | -0.11 (0.15) | 0.44 | 0.22 (0.15) | 0.16 | -0.11 (0.02) | <0.001 | -0.24 (0.19) | 0.19 |
| *t*=fu | 961 | 0.12 (0.06) | 0.07 | -0.11 (0.02) | <0.001 | 0.09 (0.09) | 0.35 | 1021 | 0.36 (0.11) | 0.001 | -0.11 (0.02) | <0.001 | -0.11 (0.15) | 0.49 | 0.28 (0.18) | 0.13 | -0.11 (0.02) | <0.001 | -0.23 (0.24) | 0.34 |
| **P3Q2** | | | | | | | | | | | | | | | | | | | | |
| *t*=base | 1009 | 0.21 (0.05) | <0.001 | -0.08 (0.03) | 0.001 | 0.04 (0.09) | 0.64 | 1021 | 0.34 (0.08) | <0.001 | -0.09 (0.03) | 0.001 | -0.04 (0.12) | 0.76 | 0.37 (0.11) | 0.001 | -0.09 (0.03) | <0.001 | -0.07 (0.14) | 0.62 |
| *t*=fu | 961 | 0.20 (0.06) | 0.001 | -0.08 (0.03) | 0.001 | 0.08 (0.09) | 0.38 | 1021 | 0.34 (0.08) | <0.001 | -0.09 (0.03) | 0.001 | 0.03 (0.11) | 0.79 | 0.33 (0.12) | 0.007 | -0.09 (0.03) | 0.001 | -0.01 (0.17) | 0.93 |
| **Experiment Part 3: Descriptive norms (Average P3Q1 to P3Q2)** | | | | | | | | | | | | | | | | | | | | |
| *t*=base | 1009 | 0.17 (0.06) | 0.005 | -0.09 (0.02) | <0.001 | 0.01 (0.09) | 0.91 | 1021 | 0.30 (0.09) | 0.001 | -0.09 (0.02) | <0.001 | -0.05 (0.13) | 0.70 | 0.27 (0.12) | 0.02 | -0.09 (0.02) | <0.001 | -0.12 (0.15) | 0.41 |
| *t*=fu | 961 | 0.15 (0.06) | 0.01 | -0.09 (0.02) | <0.001 | 0.09 (0.09) | 0.28 | 1021 | 0.33 (0.09) | <0.001 | -0.09 (0.02) | <0.001 | -0.02 (0.13) | 0.90 | 0.25 (0.14) | 0.07 | -0.09 (0.02) | <0.001 | -0.08 (0.19) | 0.65 |
| **Donation to ASSIST/Dead Cool** | | | | | | | | | | | | | | | | | | | | |
| *t*=base | 1008 | 0.12 (0.06) | 0.03 | 0.06 (0.12) | 0.59 | -0.07 (0.09) | 0.42 | 1020 | 0.14 (0.10) | 0.15 | 0.12 (0.12) | 0.30 | -0.24 (0.13) | 0.06 | 0.39 (0.22) | 0.07 | 0.10 (0.12) | 0.38 | -0.26 (0.26) | 0.33 |
| *t*=fu | 960 | 0.34 (0.06) | <0.001 | 0.02 (0.12) | 0.85 | 0.01 (0.09) | 0.87 | 1020 | 0.49 (0.09) | <0.001 | 0.06 (0.12) | 0.60 | -0.09 (0.12) | 0.47 | 0.69 (0.15) | <0.001 | 0.05 (0.12) | 0.65 | 0.01 (0.19) | 0.95 |
| **IN1** | | | | | | | | | | | | | | | | | | | | |
| *t*=base | 1013 | -0.04 (0.06) | 0.56 | 0.09 (0.04) | 0.01 | 0.23 (0.12) | 0.05 | 1021 | 0.04 (0.14) | 0.78 | 0.10 (0.04) | 0.008 | 0.12 (0.21) | 0.56 | 0.36 (0.23) | 0.11 | 0.10 (0.04) | 0.008 | 0.21 (0.37) | 0.57 |
| *t*=fu | 1011 | 0.07 (0.06) | 0.31 | 0.09 (0.04) | 0.01 | 0.09 (0.10) | 0.36 | 1021 | -0.22 (0.13) | 0.08 | 0.10 (0.04) | 0.006 | 0.37 (0.22) | 0.10 | 0.20 (0.23) | 0.39 | 0.10 (0.04) | 0.008 | 0.19 (0.45) | 0.68 |
| **IN2** | | | | | | | | | | | | | | | | | | | | |
| *t*=base | 1015 | 0.01 (0.09) | 0.91 | -0.01 (0.02) | 0.50 | 0.13 (0.13) | 0.34 | 1023 | 0.24 (0.20) | 0.21 | -0.01 (0.02) | 0.50 | 0.40 (0.26) | 0.12 | 0.30 (0.59) | 0.61 | -0.01 (0.02) | 0.57 | 0.36 (0.49) | 0.46 |
| *t*=fu | 1013 | 0.17 (0.08) | 0.04 | -0.01 (0.02) | 0.57 | -0.25 (0.11) | 0.02 | 1023 | 0.15 (0.12) | 0.22 | -0.01 (0.02) | 0.55 | -0.02 (0.15) | 0.88 | 0.09 (0.28) | 0.75 | -0.01 (0.02) | 0.56 | 0.14 (0.29) | 0.65 |
| **IN3** | | | | | | | | | | | | | | | | | | | | |
| *t*=base | 1013 | 0.13 (0.07) | 0.07 | 0.03 (0.03) | 0.21 | -0.09 (0.10) | 0.34 | 1021 | 0.22 (0.11) | 0.05 | 0.03 (0.03) | 0.21 | 0.01 (0.16) | 0.95 | 0.30 (0.20) | 0.13 | 0.04 (0.03) | 0.18 | 0.12 (0.21) | 0.55 |
| *t*=fu | 1011 | 0.08 (0.06) | 0.16 | 0.03 (0.03) | 0.27 | -0.11 (0.07) | 0.09 | 1021 | -0.01 (0.10) | 0.89 | 0.03 (0.03) | 0.24 | -0.006 (0.15) | 0.97 | 0.15 (0.20) | 0.45 | 0.03 (0.03) | 0.22 | 0.04 (0.24) | 0.87 |
| **IN4** | | | | | | | | | | | | | | | | | | | | |
| *t*=base | 1012 | 0.02 (0.06) | 0.71 | 0.07 (0.04) | 0.06 | -0.17 (0.09) | 0.05 | 1021 | -0.03 (0.13) | 0.82 | 0.06 (0.04) | 0.09 | -0.29 (0.18) | 0.11 | 0.44 (0.32) | 0.17 | 0.07 (0.04) | 0.06 | -0.64 (0.42) | 0.13 |
| *t*=fu | 1011 | 0.06 (0.06) | 0.27 | 0.06 (0.04) | 0.10 | 0.04 (0.09) | 0.65 | 1021 | 0.11 (0.12) | 0.34 | 0.06 (0.04) | 0.09 | -0.22 (0.16) | 0.17 | 0.25 (0.23) | 0.27 | 0.06 (0.04) | 0.09 | -0.24 (0.30) | 0.43 |
| **IN5** | | | | | | | | | | | | | | | | | | | | |
| *t*=base | 1014 | -0.008 (0.06) | 0.89 | -0.02 (0.04) | 0.55 | -0.002 (0.08) | 0.98 | 1022 | -0.08 (0.11) | 0.47 | -0.02 (0.04) | 0.50 | 0.15 (0.17) | 0.38 | -0.12 (0.24) | 0.62 | -0.02 (0.04) | 0.49 | 0.21 (0.29) | 0.46 |
| *t*=fu | 1012 | 0.02 (0.06) | 0.76 | -0.02 (0.04) | 0.60 | -0.15 (0.10) | 0.13 | 1022 | 0.008 (0.12) | 0.95 | -0.02 (0.04) | 0.51 | -0.06 (0.17) | 0.73 | 0.06 (0.21) | 0.77 | -0.02 (0.04) | 0.53 | -0.21 (0.29) | 0.48 |
| **IN6** | | | | | | | | | | | | | | | | | | | | |
| *t*=base | 1014 | 0.20 (0.06) | 0.001 | 0.09 (0.04) | 0.03 | -0.02 (0.09) | 0.81 | 1022 | 0.19 (0.10) | 0.06 | 0.09 (0.04) | 0.01 | 0.08 (0.14) | 0.55 | 0.48 (0.17) | 0.004 | 0.11 (0.04) | 0.006 | -0.04 (0.23) | 0.85 |
| *t*=fu | 1012 | 0.23 (0.06) | <0.001 | 0.09 (0.04) | 0.02 | -0.19 (0.09) | 0.03 | 1022 | 0.25 (0.09) | 0.008 | 0.10 (0.04) | 0.01 | 0.05 (0.13) | 0.70 | 0.47 (0.16) | 0.004 | 0.11 (0.04) | 0.006 | -0.04 (0.21) | 0.84 |
| **IN7** | | | | | | | | | | | | | | | | | | | | |
| *t*=base | 1015 | 0.09 (0.06) | 0.11 | 0.12 (0.03) | <0.001 | -0.004 (0.08) | 0.96 | 1023 | 0.20 (0.12) | 0.10 | 0.13 (0.03) | <0.001 | -0.007 (0.17) | 0.97 | 0.63 (0.19) | 0.001 | 0.14 (0.03) | <0.001 | -0.26 (0.22) | 0.25 |
| *t*=fu | 1013 | 0.18 (0.06) | 0.006 | 0.12 (0.03) | <0.001 | -0.13 (0.10) | 0.21 | 1023 | 0.21 (0.11) | 0.06 | 0.13 (0.03) | <0.001 | -0.07 (0.16) | 0.63 | 0.39 (0.18) | 0.03 | 0.13 (0.03) | <0.001 | -0.10 (0.21) | 0.64 |
| **Self-report injunctive norms (Average IN1 to IN7)** | | | | | | | | | | | | | | | | | | | | |
| *t*=base | 1009 | 0.08 (0.06) | 0.17 | 0.04 (0.02) | 0.05 | 0.004 (0.09) | 0.96 | 1018 | 0.07 (0.12) | 0.52 | 0.04 (0.02) | 0.04 | 0.03 (0.15) | 0.83 | 0.44 (0.18) | 0.02 | 0.04 (0.02) | 0.03 | -0.16 (0.22) | 0.48 |
| *t*=fu | 1008 | 0.18 (0.05) | 0.001 | 0.04 (0.02) | 0.05 | -0.19 (0.08) | 0.02 | 1018 | 0.15 (0.09) | 0.11 | 0.04 (0.02) | 0.04 | -0.0005 (0.13) | 1.00 | 0.28 (0.16) | 0.08 | 0.04 (0.02) | 0.04 | -0.13 (0.20) | 0.50 |
| **DN1.1** | | | | | | | | | | | | | | | | | | | | |
| *t*=base | 1015 | 0.33 (0.11) | 0.004 | -0.01 (0.04) | 0.72 | 0.15 (0.15) | 0.32 | 1023 | 0.33 (0.17) | 0.05 | -0.007 (0.04) | 0.85 | 0.31 (0.36) | 0.39 | 1.12 (0.35) | 0.001 | -0.02 (0.04) | 0.70 | 0.53 (0.55) | 0.33 |
| *t*=fu | 1013 | 0.37 (0.11) | 0.001 | -0.01 (0.04) | 0.71 | 0.10 (0.15) | 0.50 | 1023 | 0.27 (0.16) | 0.10 | -0.008 (0.04) | 0.83 | 0.32 (0.34) | 0.36 | 0.67 (0.21) | 0.001 | -0.01 (0.04) | 0.79 | 0.28 (0.42) | 0.50 |
| **DN1.2** | | | | | | | | | | | | | | | | | | | | |
| *t*=base | 1016 | 0.12 (0.05) | 0.02 | 0.009 (0.03) | 0.78 | 0.05 (0.07) | 0.51 | 1024 | 0.09 (0.07) | 0.22 | 0.02 (0.03) | 0.57 | 0.02 (0.11) | 0.84 | 0.16 (0.09) | 0.07 | 0.02 (0.03) | 0.58 | -0.03 (0.12) | 0.81 |
| *t*=fu | 1014 | -0.002 (0.04) | 0.96 | 0.01 (0.03) | 0.65 | 0.14 (0.09) | 0.13 | 1024 | 0.03 (0.08) | 0.68 | 0.02 (0.03) | 0.54 | 0.02 (0.13) | 0.87 | 0.11 (0.09) | 0.26 | 0.02 (0.03) | 0.51 | -0.04 (0.13) | 0.77 |
| **DN1.3** | | | | | | | | | | | | | | | | | | | | |
| *t*=base | 1015 | 0.08 (0.05) | 0.08 | 0.03 (0.04) | 0.42 | 0.02 (0.05) | 0.69 | 1023 | 0.06 (0.08) | 0.48 | 0.03 (0.04) | 0.42 | 0.16 (0.12) | 0.20 | 0.13 (0.13) | 0.34 | 0.02 (0.04) | 0.53 | 0.17 (0.17) | 0.31 |
| *t*=fu | 1013 | 0.03 (0.06) | 0.65 | 0.04 (0.03) | 0.27 | -0.03 (0.05) | 0.59 | 1023 | 0.03 (0.08) | 0.68 | 0.03 (0.04) | 0.38 | 0.15 (0.13) | 0.25 | 0.07 (0.15) | 0.64 | 0.03 (0.04) | 0.44 | 0.17 (0.19) | 0.36 |
| **DN1.4** | | | | | | | | | | | | | | | | | | | | |
| *t*=base | 1016 | 0.01 (0.05) | 0.76 | -0.007 (0.03) | 0.84 | -0.05 (0.06) | 0.47 | 1024 | 0.09 (0.15) | 0.53 | -0.001 (0.03) | 0.96 | -0.02 (0.17) | 0.93 | 0.13 (0.15) | 0.40 | -0.003 (0.03) | 0.92 | 0.08 (0.21) | 0.70 |
| *t*=fu | 1014 | -0.07 (0.04) | 0.08 | -0.007 (0.03) | 0.83 | 0.04 (0.07) | 0.63 | 1024 | 0.14 (0.16) | 0.40 | -0.005 (0.03) | 0.88 | 0.12 (0.20) | 0.56 | -0.10 (0.18) | 0.59 | -0.006 (0.03) | 0.86 | 0.15 (0.24) | 0.54 |
| **DN1.5** | | | | | | | | | | | | | | | | | | | | |
| *t*=base | 1016 | 0.07 (0.05) | 0.16 | 0.008 (0.02) | 0.68 | -0.13 (0.08) | 0.10 | 1024 | 0.22 (0.13) | 0.09 | 0.007 (0.02) | 0.71 | -0.10 (0.17) | 0.55 | 0.39 (0.20) | 0.05 | 0.006 (0.02) | 0.78 | 0.14 (0.27) | 0.60 |
| *t*=fu | 1014 | -0.01 (0.05) | 0.79 | 0.005 (0.02) | 0.81 | 0.02 (0.06) | 0.74 | 1024 | 0.24 (0.12) | 0.04 | 0.007 (0.02) | 0.74 | -0.11 (0.18) | 0.54 | 0.16 (0.17) | 0.34 | 0.006 (0.02) | 0.78 | -0.04 (0.18) | 0.82 |
| **Self-report descriptive norms 1 (Average DN1.1 to DN1.5)** | | | | | | | | | | | | | | | | | | | | |
| *t*=base | 1014 | 0.11 (0.05) | 0.03 | -0.005 (0.02) | 0.79 | 0.01 (0.08) | 0.89 | 1022 | 0.10 (0.08) | 0.21 | -0.004 (0.02) | 0.84 | 0.10 (0.12) | 0.39 | 0.21 (0.11) | 0.05 | -0.004 (0.02) | 0.84 | 0.06 (0.14) | 0.67 |
| *t*=fu | 1012 | 0.05 (0.05) | 0.38 | -0.004 (0.02) | 0.81 | 0.06 (0.09) | 0.50 | 1022 | 0.06 (0.08) | 0.41 | -0.005 (0.02) | 0.79 | 0.14 (0.14) | 0.31 | 0.17 (0.10) | 0.10 | -0.004 (0.02) | 0.82 | 0.09 (0.14) | 0.53 |
| **DN2.1** | | | | | | | | | | | | | | | | | | | | |
| *t*=base | 1016 | 0.22 (0.08) | 0.005 | 0.006 (0.03) | 0.85 | 0.22 (0.10) | 0.03 | 1024 | 0.22 (0.11) | 0.05 | 0.02 (0.03) | 0.56 | 0.22 (0.19) | 0.26 | 0.27 (0.15) | 0.07 | 0.02 (0.03) | 0.57 | 0.21 (0.25) | 0.40 |
| *t*=fu | 1014 | 0.22 (0.07) | 0.002 | 0.02 (0.03) | 0.42 | 0.03 (0.09) | 0.71 | 1024 | 0.22 (0.12) | 0.07 | 0.02 (0.03) | 0.48 | 0.10 (0.19) | 0.60 | 0.20 (0.17) | 0.24 | 0.02 (0.03) | 0.58 | 0.34 (0.24) | 0.15 |
| **DN2.2** | | | | | | | | | | | | | | | | | | | | |
| *t*=base | 1016 | 0.18 (0.07) | 0.006 | 0.03 (0.04) | 0.44 | 0.11 (0.10) | 0.28 | 1024 | 0.33 (0.10) | 0.001 | 0.04 (0.04) | 0.39 | 0.09 (0.15) | 0.54 | 0.51 (0.17) | 0.003 | 0.03 (0.04) | 0.57 | 0.22 (0.23) | 0.34 |
| *t*=fu | 1014 | 0.11 (0.06) | 0.07 | 0.04 (0.04) | 0.30 | 0.06 (0.09) | 0.49 | 1024 | 0.18 (0.09) | 0.05 | 0.04 (0.04) | 0.31 | 0.07 (0.14) | 0.63 | 0.41 (0.16) | 0.01 | 0.03 (0.04) | 0.43 | 0.19 (0.21) | 0.36 |
| **DN2.3** | | | | | | | | | | | | | | | | | | | | |
| *t*=base | 1016 | 0.26 (0.08) | 0.001 | 0.03 (0.03) | 0.31 | -0.07 (0.09) | 0.44 | 1024 | 0.59 (0.09) | <0.001 | 0.02 (0.03) | 0.48 | -0.09 (0.12) | 0.45 | 0.62 (0.13) | <0.001 | 0.03 (0.03) | 0.31 | -0.17 (0.18) | 0.34 |
| *t*=fu | 1014 | 0.19 (0.06) | 0.002 | 0.03 (0.03) | 0.19 | -0.09 (0.07) | 0.23 | 1024 | 0.41 (0.09) | <0.001 | 0.02 (0.03) | 0.44 | -0.13 (0.10) | 0.20 | 0.53 (0.12) | <0.001 | 0.03 (0.03) | 0.34 | -0.22 (0.15) | 0.16 |
| **Self-report descriptive norms 2 (Average DN2.1 to DN2.3)** | | | | | | | | | | | | | | | | | | | | |
| *t*=base | 1016 | 0.23 (0.06) | <0.001 | 0.01 (0.02) | 0.69 | 0.16 (0.10) | 0.11 | 1024 | 0.40 (0.10) | <0.001 | 0.01 (0.02) | 0.58 | 0.15 (0.16) | 0.35 | 0.45 (0.14) | 0.001 | 0.02 (0.03) | 0.55 | 0.09 (0.22) | 0.66 |
| *t*=fu | 1014 | 0.13 (0.06) | 0.02 | 0.02 (0.02) | 0.36 | 0.09 (0.09) | 0.30 | 1024 | 0.29 (0.08) | 0.001 | 0.02 (0.02) | 0.44 | 0.08 (0.13) | 0.54 | 0.39 (0.13) | 0.003 | 0.02 (0.02) | 0.49 | 0.11 (0.20) | 0.56 |
| **Self-report smoking behavior** | | | | | | | | | | | | | | | | | | | | |
| *t*=base | 1019 | 0.15 (0.07) | 0.04 | 0.07 (0.02) | 0.001 | -0.02 (0.09) | 0.82 | 1027 | 0.29 (0.11) | 0.01 | 0.08 (0.02) | <0.001 | -0.24 (0.14) | 0.09 | 0.66 (0.17) | <0.001 | 0.08 (0.02) | <0.001 | -0.39 (0.18) | 0.03 |
| *t*=fu | 1017 | 0.22 (0.06) | <0.001 | 0.07 (0.02) | 0.001 | -0.21 (0.09) | 0.03 | 1027 | 0.24 (0.09) | 0.004 | 0.08 (0.02) | <0.001 | -0.12 (0.11) | 0.27 | **0.46 (0.11)** | **<0.001** | **0.09 (0.02)** | **<0.001** | **-0.33 (0.13)** | **0.009** |
| **Intentions** | | | | | | | | | | | | | | | | | | | | |
| *t*=base | 1015 | 0.14 (0.09) | 0.12 | 0.20 (0.05) | <0.001 | 0.01 (0.12) | 0.93 | 1023 | 0.44 (0.14) | 0.002 | 0.21 (0.05) | <0.001 | -0.26 (0.20) | 0.20 | 0.95 (0.26) | <0.001 | 0.22 (0.05) | <0.001 | -0.57 (0.37) | 0.13 |
| *t*=fu | 1013 | 0.19 (0.07) | 0.009 | 0.21 (0.05) | <0.001 | -0.10 (0.12) | 0.37 | 1023 | 0.10 (0.11) | 0.38 | 0.21 (0.05) | <0.001 | -0.14 (0.16) | 0.39 | 0.37 (0.15) | 0.02 | 0.22 (0.05) | <0.001 | -0.32 (0.24) | 0.18 |
| **Knowledge** | | | | | | | | | | | | | | | | | | | | |
| *t*=base | 1018 | 0.21 (0.06) | <0.001 | 0.09 (0.06) | 0.17 | -0.09 (0.08) | 0.26 | 1026 | 0.45 (0.08) | <0.001 | 0.11 (0.06) | 0.07 | -0.15 (0.12) | 0.21 | 0.47 (0.11) | <0.001 | 0.12 (0.06) | 0.06 | 0.05 (0.14) | 0.72 |
| *t*=fu | 1016 | 0.29 (0.06) | <0.001 | 0.11 (0.06) | 0.08 | -0.07 (0.08) | 0.39 | 1026 | 0.43 (0.08) | <0.001 | 0.11 (0.06) | 0.09 | -0.10 (0.11) | 0.34 | 0.45 (0.12) | <0.001 | 0.11 (0.06) | 0.07 | 0.02 (0.13) | 0.87 |
| **Attitudes** | | | | | | | | | | | | | | | | | | | | |
| *t*=base | 1007 | 0.15 (0.06) | 0.01 | 0.11 (0.03) | <0.001 | -0.07 (0.08) | 0.40 | 1015 | -0.06 (0.11) | 0.62 | 0.11 (0.03) | <0.001 | -0.19 (0.14) | 0.18 | 0.38 (0.22) | 0.09 | 0.11 (0.03) | <0.001 | -0.63 (0.29) | 0.03 |
| *t*=fu | 1005 | 0.16 (0.07) | 0.01 | 0.10 (0.03) | <0.001 | -0.16 (0.09) | 0.07 | 1015 | -0.11 (0.12) | 0.36 | 0.11 (0.03) | <0.001 | -0.35 (0.15) | 0.02 | **0.23 (0.18)** | **0.19** | **0.12 (0.03)** | **<0.001** | **-0.84 (0.23)** | **<0.001** |
| **Self-efficacy (Emotional)** | | | | | | | | | | | | | | | | | | | | |
| *t*=base | 1010 | 0.07 (0.07) | 0.31 | 0.13 (0.04) | <0.001 | -0.11 (0.11) | 0.31 | 1018 | 0.28 (0.12) | 0.02 | 0.13 (0.03) | <0.001 | -0.36 (0.15) | 0.02 | 0.43 (0.17) | 0.01 | 0.13 (0.04) | <0.001 | -0.39 (0.20) | 0.05 |
| *t*=fu | 1007 | 0.26 (0.06) | <0.001 | 0.13 (0.03) | <0.001 | -0.22 (0.10) | 0.04 | **1018** | **0.24 (0.09)** | **0.01** | **0.13 (0.03)** | **<0.001** | **-0.31 (0.11)** | **0.005** | 0.41 (0.14) | 0.003 | 0.14 (0.03) | <0.001 | -0.32 (0.16) | 0.05 |
| **Self-efficacy (Friends)** | | | | | | | | | | | | | | | | | | | | |
| *t*=base | 1015 | 0.04 (0.06) | 0.51 | 0.14 (0.03) | <0.001 | 0.02 (0.10) | 0.87 | 1023 | 0.20 (0.12) | 0.09 | 0.15 (0.03) | <0.001 | -0.23 (0.14) | 0.10 | 0.19 (0.18) | 0.29 | 0.15 (0.03) | <0.001 | -0.44 (0.22) | 0.04 |
| *t*=fu | 1013 | 0.18 (0.06) | 0.005 | 0.14 (0.03) | <0.001 | -0.04 (0.11) | 0.74 | 1023 | 0.07 (0.10) | 0.47 | 0.15 (0.03) | <0.001 | -0.17 (0.13) | 0.21 | 0.28 (0.20) | 0.16 | 0.15 (0.03) | <0.001 | -0.35 (0.23) | 0.12 |
| **Self-efficacy (Opportunity)** | | | | | | | | | | | | | | | | | | | | |
| *t*=base | 1016 | 0.11 (0.07) | 0.11 | 0.09 (0.03) | 0.001 | 0.05 (0.09) | 0.55 | 1024 | 0.41 (0.15) | 0.008 | 0.10 (0.03) | <0.001 | -0.13 (0.16) | 0.44 | 0.42 (0.21) | 0.05 | 0.10 (0.03) | <0.001 | -0.28 (0.24) | 0.25 |
| *t*=fu | 1014 | 0.16 (0.07) | 0.01 | 0.09 (0.03) | 0.001 | 0.08 (0.08) | 0.33 | **1024** | **0.23 (0.11)** | **0.03** | **0.10 (0.03)** | **<0.001** | **-0.34 (0.12)** | **0.003** | 0.31 (0.18) | 0.09 | 0.10 (0.03) | <0.001 | -0.34 (0.18) | 0.06 |
| **Perceived physical risks** | | | | | | | | | | | | | | | | | | | | |
| *t*=base | 1012 | 0.07 (0.05) | 0.18 | 0.61 (0.91) | 0.50 | -0.08 (0.09) | 0.37 | 1020 | 0.17 (0.09) | 0.06 | 0.59 (0.91) | 0.52 | -0.16 (0.13) | 0.20 | 0.10 (0.15) | 0.51 | 0.67 (0.91) | 0.46 | -0.21 (0.18) | 0.26 |
| *t*=fu | 1010 | 0.12 (0.06) | 0.03 | 0.56 (0.92) | 0.55 | 0.02 (0.07) | 0.79 | 1020 | 0.11 (0.11) | 0.35 | 0.65 (0.92) | 0.48 | -0.10 (0.13) | 0.44 | -0.10 (0.22) | 0.64 | 0.68 (0.94) | 0.47 | -0.22 (0.20) | 0.28 |
| **Perceived social risks** | | | | | | | | | | | | | | | | | | | | |
| *t*=base | 1014 | 0.14 (0.05) | 0.007 | 0.48 (1.02) | 0.64 | -0.08 (0.07) | 0.26 | 1022 | 0.25 (0.07) | 0.001 | 0.50 (1.01) | 0.62 | -0.03 (0.09) | 0.73 | 0.39 (0.10) | <0.001 | 0.99 (1.01) | 0.33 | -0.06 (0.11) | 0.59 |
| *t*=fu | 1012 | 0.13 (0.06) | 0.02 | 0.35 (1.01) | 0.73 | -0.12 (0.08) | 0.12 | 1022 | 0.21 (0.09) | 0.02 | 0.53 (1.02) | 0.60 | -0.05 (0.11) | 0.65 | 0.39 (0.12) | 0.001 | 0.88 (1.03) | 0.39 | -0.10 (0.13) | 0.44 |
| **Perceived addiction risks** | | | | | | | | | | | | | | | | | | | | |
| *t*=base | 957 | 0.21 (0.06) | <0.001 | -0.84 (1.16) | 0.47 | 0.12 (0.07) | 0.10 | 965 | 0.49 (0.09) | <0.001 | -0.43 (1.16) | 0.72 | 0.14 (0.12) | 0.24 | 0.72 (0.11) | <0.001 | -0.14 (1.14) | 0.90 | 0.15 (0.13) | 0.27 |
| *t*=fu | 951 | 0.15 (0.05) | 0.003 | -0.65 (1.18) | 0.58 | -0.04 (0.06) | 0.51 | 965 | 0.41 (0.08) | <0.001 | -0.43 (1.16) | 0.71 | 0.16 (0.10) | 0.12 | 0.58 (0.09) | <0.001 | -0.28 (1.14) | 0.81 | 0.14 (0.12) | 0.23 |
| **Perceived benefits** | | | | | | | | | | | | | | | | | | | | |
| *t*=base | 974 | -0.05 (0.06) | 0.48 | -2.14 (0.96) | 0.03 | -0.03 (0.11) | 0.75 | 982 | 0.07 (0.13) | 0.56 | -1.90 (0.97) | 0.05 | 0.05 (0.19) | 0.78 | 0.34 (0.21) | 0.11 | -1.84 (0.98) | 0.06 | 0.04 (0.31) | 0.89 |
| *t*=fu | 971 | 0.003 (0.06) | 0.95 | -1.91 (0.99) | 0.06 | 0.10 (0.09) | 0.26 | 982 | -0.10 (0.13) | 0.45 | -1.94 (0.97) | 0.05 | 0.12 (0.20) | 0.52 | -0.11 (0.26) | 0.67 | -1.93 (0.98) | 0.05 | 0.15 (0.44) | 0.73 |
| **Perceived behavioral control (easy to quit)** | | | | | | | | | | | | | | | | | | | | |
| *t*=base | 1014 | 0.29 (0.06) | <0.001 | 0.02 (0.07) | 0.73 | -0.04 (0.09) | 0.62 | 1022 | 0.55 (0.08) | <0.001 | -0.008 (0.07) | 0.90 | -0.05 (0.11) | 0.64 | 0.72 (0.10) | <0.001 | -0.03 (0.06) | 0.68 | 0.004 (0.13) | 0.98 |
| *t*=fu | 1012 | 0.16 (0.06) | 0.007 | 0.02 (0.07) | 0.79 | 0.003 (0.09) | 0.97 | 1022 | 0.49 (0.08) | <0.001 | -0.02 (0.06) | 0.80 | -0.07 (0.11) | 0.54 | 0.66 (0.10) | <0.001 | -0.03 (0.06) | 0.66 | -0.004 (0.12) | 0.97 |
| **Perceived behavioral control (to avoid smoking)** | | | | | | | | | | | | | | | | | | | | |
| *t*=base | 1017 | 0.07 (0.07) | 0.33 | 0.10 (0.05) | 0.07 | -0.02 (0.10) | 0.84 | 1025 | 0.40 (0.14) | 0.004 | 0.11 (0.05) | 0.05 | 0.02 (0.19) | 0.93 | 0.83 (0.23) | <0.001 | 0.13 (0.05) | 0.02 | 0.35 (0.31) | 0.26 |
| *t*=fu | 1015 | 0.08 (0.07) | 0.25 | 0.10 (0.05) | 0.07 | 0.12 (0.11) | 0.28 | 1025 | 0.15 (0.14) | 0.29 | 0.10 (0.05) | 0.06 | 0.34 (0.18) | 0.06 | 0.57 (0.18) | 0.002 | 0.12 (0.05) | 0.02 | 0.36 (0.23) | 0.12 |
| **Objectively measured smoking behavior** | | | | | | | | | | | | | | | | | | | | |
| *t*=base | 981 | 0.36 (0.05) | <0.001 | 0.05 (0.07) | 0.43 | 0.001 (0.06) | 0.98 | 987 | 0.37 (0.06) | <0.001 | 0.02 (0.07) | 0.71 | -0.006 (0.05) | 0.91 | 0.56 (0.06) | <0.001 | 0.002 (0.07) | 0.97 | 0.006 (0.06) | 0.92 |
| *t*=fu | 963 | 0.47 (0.08) | <0.001 | 0.01 (0.06) | 0.81 | -0.07 (0.10) | 0.47 | 987 | 0.82 (0.06) | <0.001 | -0.002 (0.05) | 0.98 | 0.03 (0.07) | 0.68 | 0.85 (0.06) | <0.001 | -0.01 (0.06) | 0.84 | 0.02 (0.07) | 0.78 |
| **Smoking susceptibility^e^** | | | | | | | | | | | | | | | | | | | | |
|  | n | **Percent_-i,t_^f^** | | **M^g^** | | **M*Percent_-i,t_^h^** | | n | **Percent_-i,t_^f^** | | **M^g^** | | **M*Percent_-i,t_^h^** | | **Percent_-i,t_^f^** | | **M^g^** | | **M*Percent_-i,t_^h^** | |
|  |  | OR (SE) | p-value | OR (SE) | p-value | OR (SE) | p-value |  | OR (SE) | p-value | OR (SE) | p-value | OR (SE) | p-value | OR (SE) | p-value | OR (SE) | p-value | OR (SE) | p-value |
| *t*=base | 1015 | 1.13 (0.04) | <0.001 | 0.68 (0.08) | 0.001 | 1.00 (0.05) | 0.97 | 1023 | 1.06 (0.06) | 0.33 | 0.65 (0.08) | <0.001 | 1.01 (0.10) | 0.96 | 1.20 (0.12) | 0.06 | 0.64 (0.07) | <0.001 | 0.95 (0.13) | 0.73 |
| *t*=fu | 1013 | 1.12 (0.03) | <0.001 | 0.66 (0.08) | <0.001 | 1.01 (0.05) | 0.86 | 1023 | 1.16 (0.06) | 0.006 | 0.63 (0.07) | <0.001 | 1.03 (0.09) | 0.73 | 1.30 (0.10) | 0.001 | 0.62 (0.07) | <0.001 | 0.94 (0.10) | 0.56 |

IV: Independent variable; DV: Dependent variable; Ave: average of peer group (*i*); M: moderator ('Big Five' conscientiousness: 0 [least conscientious] to 4 [most conscientious]); Percent: percentage of peer group (*-i*) classified as susceptible to commencing smoking; OR: odds ratio; SE: standard error.

^a^In each model the outcome variable is the focal participant’s (*i*) response to the relevant item at follow-up. The predictor variable is the average of the relevant group’s (*-i*) responses to the equivalent item at baseline (*t*=base) or follow-up (*t*=fu), where *–i*=(1) focal participant's nominated friends; (2) focal participant’s school class; (3) focal participant’s school year group. The moderator, and interaction of the moderator with the predictor variable, were also included as independent variables in all models. All models include robust (Huber White) standard errors specified using Stata’s ‘vce(robust)’ option. The following baseline variables are included as covariates in all models: gender (0=boy; 1=girl/prefer not to say), age (1=12 years or less; 2=13 years; 3=14 years or more), intervention (1=ASSIST; 2=Dead Cool), ethnicity (0=no ethnic minority; 1=ethnic minority), individuals' socio-economic status (NI: 1=NIMDM2017≤296.6; 2=296.6<NIMDM2017≤593.2; 3=NIMDM2017>593.2; Bogotá: 1=Informal settlement/Lowest/Low; 2=Middle-Low/Middle; 3=Middle-High/High), and baseline values of the outcome variable. The predictor variable, moderator variable, and baseline values of the outcome variable were mean-centered.

^b^Unstandardized regression coefficients representing the average change in the outcome variable for a one-unit increase in the predictor variable among participants who are average on conscientiousness.

^c^Unstandardized regression coefficients representing the average change in the outcome variable for a one-unit increase in conscientiousness among participants who are average on the predictor variable.

^d^Unstandardized regression coefficients representing the average change in the association between the outcome variable and the predictor variable for a one-unit increase in conscientiousness.

^e^Logistic regressions were run for models including focal participants' smoking susceptibility as the outcome variable, with robust (Huber White) standard errors specified using Stata’s ‘vce(robust)’ option. The predictor variable is the percentage of the relevant group (*-i*) classified as susceptible to commencing smoking at baseline (*t*=base) or follow-up (*t*=fu), where *–i*=(1) focal participant's nominated friends; (2) focal participant’s school class; (3) focal participant’s school year group. The moderator, and interaction of the moderator with the predictor variable, were also included as independent variables in all models. The following baseline variables are included as covariates in all models: gender (0=boy; 1=girl/prefer not to say), age (1=12 years or less; 2=13 years; 3=14 years or more), intervention (1=ASSIST; 2=Dead Cool), ethnicity (0=no ethnic minority; 1=ethnic minority), individuals' socio-economic status (NI: 1=NIMDM2017≤296.6; 2=296.6<NIMDM2017≤593.2; 3=NIMDM2017>593.2; Bogotá: 1=Informal settlement/Lowest/Low; 2=Middle-Low/Middle; 3=Middle-High/High), and baseline values of the outcome variable. Results are odds ratios, standard errors, and p-values. The predictor variable, and moderator variable were mean-centered.

^f^Odds ratios representing the multiplicative change in odds of being susceptible to commencing smoking for a 10% increase in the number of nominated friends/pupils in the same school class/pupils in the same school year group classified as being susceptible to commencing smoking (1 out of 10 nominated friends/pupils in the same school class/pupils in the same school year group; predictor variable) among participants who are average on conscientiousness.

^g^Odds ratios representing the multiplicative change in odds of being susceptible to commencing smoking for a one-unit increase in conscientiousness among participants who are average on the predictor variable.

^h^Ratio of ratios representing the ratio of: (1) the odds ratio representing the multiplicative change in odds of being susceptible to commencing smoking for a 10% increase in the number of nominated friends/pupils in the same school class/pupils in the same school year group classified as being susceptible to commencing smoking among participants who are one unit above average on conscientiousness; to (2) the odds ratio representing the multiplicative change in odds of being susceptible to commencing smoking for a 10% increase in the number of nominated friends/pupils in the same school class/pupils in the same school year group classified as being susceptible to commencing smoking among participants who are average on conscientiousness.

**Table S2.15.** Results of ordinary least squares linear regressions including interaction terms examining differences in peer influence effects according to emotional stability for outcomes collected at follow-up.

|  | **Dependent variable: Participant responses to the outcome variable at follow-up^a^** | | | | | | | | | | | | | | | | | | | |
| --- | --- | --- | --- | --- | --- | --- | --- | --- | --- | --- | --- | --- | --- | --- | --- | --- | --- | --- | --- | --- |
| **(1) -i=Average of nominated friends** | | | | | | | n | **(2) -i=Average of school class** | | | | | | **(3) -i=Average of school year group** | | | | | |
| **IV** | n | **Ave_-i,t_^b^** | | **M^c^** | | **M*Ave_-i,t_^d^** | | **Ave_-i,t_^b^** | | **M^c^** | | **M*Ave_-i,t_^d^** | | **Ave_-i,t_^b^** | | **M^c^** | | **M*Ave_-i,t_^d^** | |
| **DV** | *b* (SE) | p-value | *b* (SE) | p-value | *b* (SE) | p-value | *b* (SE) | p-value | *b* (SE) | p-value | *b* (SE) | p-value | *b* (SE) | p-value | *b* (SE) | p-value | *b* (SE) | p-value |
| **P2S2** | | | | | | | | | | | | | | | | | | | | |
| *t*=base | 1012 | 0.35 (0.08) | <0.001 | -0.02 (0.01) | 0.23 | -0.25 (0.13) | 0.05 | 1024 | 0.43 (0.12) | <0.001 | -0.02 (0.01) | 0.21 | -0.06 (0.14) | 0.67 | 0.81 (0.19) | <0.001 | -0.01 (0.01) | 0.30 | -0.0007 (0.23) | 1.00 |
| *t*=fu | 962 | 0.16 (0.07) | 0.02 | -0.009 (0.01) | 0.50 | 0.13 (0.07) | 0.09 | 1024 | 0.33 (0.10) | 0.001 | -0.02 (0.01) | 0.24 | 0.18 (0.14) | 0.20 | 0.56 (0.17) | 0.001 | -0.02 (0.01) | 0.27 | -0.05 (0.20) | 0.79 |
| **P2S3** | | | | | | | | | | | | | | | | | | | | |
| *t*=base | 1012 | 0.11 (0.07) | 0.13 | -0.01 (0.01) | 0.37 | 0.00002 (0.09) | 1.00 | 1024 | 0.17 (0.17) | 0.30 | -0.01 (0.01) | 0.36 | 0.02 (0.25) | 0.94 | 0.20 (0.30) | 0.50 | -0.02 (0.01) | 0.29 | -0.39 (0.35) | 0.26 |
| *t*=fu | 962 | 0.12 (0.07) | 0.09 | -0.01 (0.01) | 0.37 | -0.02 (0.09) | 0.85 | 1024 | 0.23 (0.15) | 0.11 | -0.01 (0.01) | 0.36 | -0.01 (0.23) | 0.95 | 0.15 (0.28) | 0.60 | -0.02 (0.01) | 0.25 | -0.50 (0.35) | 0.15 |
| **P2S4** | | | | | | | | | | | | | | | | | | | | |
| *t*=base | 1009 | 0.10 (0.08) | 0.22 | -0.01 (0.01) | 0.39 | -0.12 (0.11) | 0.27 | 1021 | 0.15 (0.13) | 0.24 | -0.01 (0.01) | 0.46 | -0.08 (0.13) | 0.52 | 0.47 (0.33) | 0.15 | -0.01 (0.01) | 0.47 | -0.24 (0.29) | 0.40 |
| *t*=fu | 960 | 0.12 (0.07) | 0.10 | -0.01 (0.01) | 0.40 | 0.15 (0.10) | 0.11 | 1021 | 0.28 (0.12) | 0.02 | -0.01 (0.01) | 0.42 | 0.05 (0.15) | 0.75 | 0.44 (0.21) | 0.04 | -0.01 (0.01) | 0.33 | -0.08 (0.20) | 0.69 |
| **P2S5** | | | | | | | | | | | | | | | | | | | | |
| *t*=base | 1012 | 0.20 (0.06) | 0.001 | -0.006 (0.02) | 0.73 | -0.06 (0.08) | 0.45 | 1024 | 0.31 (0.10) | 0.003 | -0.007 (0.02) | 0.69 | -0.11 (0.12) | 0.37 | 0.50 (0.16) | 0.002 | -0.006 (0.02) | 0.73 | -0.05 (0.19) | 0.78 |
| *t*=fu | 962 | 0.19 (0.06) | 0.002 | -0.002 (0.02) | 0.89 | 0.07 (0.08) | 0.38 | 1024 | 0.48 (0.09) | <0.001 | -0.003 (0.02) | 0.85 | 0.01 (0.12) | 0.92 | 0.48 (0.14) | 0.001 | -0.008 (0.02) | 0.61 | -0.14 (0.17) | 0.42 |
| **P2S6** | | | | | | | | | | | | | | | | | | | | |
| *t*=base | 1009 | 0.16 (0.07) | 0.02 | -0.02 (0.02) | 0.13 | 0.03 (0.08) | 0.68 | 1021 | 0.40 (0.12) | <0.001 | -0.02 (0.02) | 0.13 | 0.12 (0.11) | 0.30 | 0.44 (0.19) | 0.02 | -0.02 (0.02) | 0.13 | 0.02 (0.18) | 0.93 |
| *t*=fu | 959 | 0.15 (0.06) | 0.01 | -0.02 (0.02) | 0.15 | -0.05 (0.07) | 0.40 | 1021 | 0.41 (0.10) | <0.001 | -0.02 (0.02) | 0.17 | 0.08 (0.11) | 0.45 | 0.51 (0.15) | 0.001 | -0.02 (0.02) | 0.12 | -0.11 (0.15) | 0.48 |
| **P2S7** | | | | | | | | | | | | | | | | | | | | |
| *t*=base | 1011 | 0.15 (0.06) | 0.005 | -0.02 (0.02) | 0.29 | -0.08 (0.08) | 0.33 | 1023 | 0.28 (0.09) | 0.003 | -0.02 (0.02) | 0.25 | -0.06 (0.13) | 0.63 | 0.28 (0.14) | 0.05 | -0.02 (0.02) | 0.23 | -0.08 (0.16) | 0.61 |
| *t*=fu | 962 | 0.26 (0.06) | <0.001 | -0.02 (0.02) | 0.29 | 0.06 (0.08) | 0.46 | 1023 | 0.39 (0.10) | <0.001 | -0.02 (0.02) | 0.37 | 0.06 (0.12) | 0.63 | 0.32 (0.17) | 0.07 | -0.02 (0.02) | 0.19 | -0.21 (0.17) | 0.22 |
| **P2S8** | | | | | | | | | | | | | | | | | | | | |
| *t*=base | 1012 | 0.29 (0.05) | <0.001 | -0.05 (0.02) | 0.002 | -0.15 (0.07) | 0.02 | 1024 | 0.32 (0.09) | <0.001 | -0.05 (0.02) | 0.001 | -0.10 (0.11) | 0.38 | 0.50 (0.14) | <0.001 | -0.06 (0.02) | 0.001 | -0.22 (0.18) | 0.23 |
| *t*=fu | 962 | 0.24 (0.06) | <0.001 | -0.05 (0.02) | 0.004 | -0.05 (0.08) | 0.53 | 1024 | 0.46 (0.08) | <0.001 | -0.05 (0.02) | 0.001 | -0.09 (0.12) | 0.44 | 0.53 (0.14) | <0.001 | -0.06 (0.02) | 0.001 | -0.21 (0.16) | 0.19 |
| **P2S9** | | | | | | | | | | | | | | | | | | | | |
| *t*=base | 1012 | 0.16 (0.07) | 0.02 | -0.03 (0.01) | 0.05 | -0.04 (0.09) | 0.65 | 1024 | 0.51 (0.13) | <0.001 | -0.03 (0.01) | 0.06 | -0.12 (0.17) | 0.48 | 0.43 (0.22) | 0.06 | -0.03 (0.01) | 0.05 | -0.31 (0.30) | 0.30 |
| *t*=fu | 962 | 0.25 (0.07) | <0.001 | -0.03 (0.01) | 0.05 | -0.03 (0.09) | 0.75 | 1024 | 0.37 (0.12) | 0.001 | -0.03 (0.01) | 0.04 | -0.12 (0.17) | 0.50 | 0.12 (0.21) | 0.58 | -0.03 (0.01) | 0.03 | -0.22 (0.28) | 0.44 |
| **Experiment Part 2: Injunctive norms (Average P2S2 to P2S9)** | | | | | | | | | | | | | | | | | | | | |
| *t*=base | 1005 | 0.22 (0.06) | <0.001 | -0.01 (0.01) | 0.17 | -0.13 (0.07) | 0.06 | 1017 | 0.36 (0.11) | 0.001 | -0.01 (0.01) | 0.16 | -0.10 (0.13) | 0.45 | 0.42 (0.16) | 0.007 | -0.01 (0.01) | 0.13 | -0.23 (0.18) | 0.21 |
| *t*=fu | 957 | 0.26 (0.05) | <0.001 | -0.01 (0.01) | 0.24 | 0.05 (0.07) | 0.51 | 1017 | 0.48 (0.09) | <0.001 | -0.01 (0.01) | 0.20 | 0.01 (0.13) | 0.93 | 0.33 (0.14) | 0.02 | -0.02 (0.01) | 0.08 | -0.26 (0.16) | 0.11 |
| **P3Q1** | | | | | | | | | | | | | | | | | | | | |
| *t*=base | 1012 | 0.10 (0.07) | 0.14 | -0.04 (0.02) | 0.08 | -0.02 (0.09) | 0.82 | 1024 | 0.24 (0.11) | 0.03 | -0.04 (0.02) | 0.07 | 0.01 (0.14) | 0.92 | 0.17 (0.16) | 0.28 | -0.04 (0.02) | 0.08 | -0.04 (0.18) | 0.83 |
| *t*=fu | 962 | 0.11 (0.06) | 0.08 | -0.04 (0.02) | 0.05 | 0.12 (0.09) | 0.16 | 1024 | 0.31 (0.11) | 0.003 | -0.04 (0.02) | 0.05 | -0.04 (0.14) | 0.80 | 0.24 (0.18) | 0.18 | -0.04 (0.02) | 0.06 | -0.09 (0.22) | 0.69 |
| **P3Q2** | | | | | | | | | | | | | | | | | | | | |
| *t*=base | 1012 | 0.21 (0.06) | <0.001 | -0.04 (0.02) | 0.11 | 0.005 (0.07) | 0.95 | 1024 | 0.31 (0.08) | <0.001 | -0.04 (0.02) | 0.10 | 0.03 (0.10) | 0.80 | 0.32 (0.11) | 0.004 | -0.04 (0.02) | 0.11 | -0.002 (0.14) | 0.99 |
| *t*=fu | 962 | 0.18 (0.06) | 0.002 | -0.03 (0.02) | 0.15 | 0.11 (0.08) | 0.16 | 1024 | 0.30 (0.08) | <0.001 | -0.04 (0.02) | 0.08 | -0.03 (0.11) | 0.80 | 0.29 (0.12) | 0.02 | -0.04 (0.02) | 0.08 | -0.01 (0.16) | 0.94 |
| **Experiment Part 3: Descriptive norms (Average P3Q1 to P3Q2)** | | | | | | | | | | | | | | | | | | | | |
| *t*=base | 1012 | 0.16 (0.06) | 0.006 | -0.03 (0.02) | 0.10 | 0.009 (0.08) | 0.91 | 1024 | 0.27 (0.09) | 0.003 | -0.04 (0.02) | 0.09 | 0.04 (0.12) | 0.71 | 0.22 (0.12) | 0.07 | -0.03 (0.02) | 0.09 | -0.01 (0.14) | 0.92 |
| *t*=fu | 962 | 0.14 (0.06) | 0.02 | -0.04 (0.02) | 0.09 | 0.13 (0.08) | 0.10 | 1024 | 0.28 (0.09) | 0.002 | -0.04 (0.02) | 0.07 | -0.02 (0.12) | 0.86 | 0.21 (0.14) | 0.13 | -0.04 (0.02) | 0.07 | -0.04 (0.18) | 0.84 |
| **Donation to ASSIST/Dead Cool** | | | | | | | | | | | | | | | | | | | | |
| *t*=base | 1011 | 0.14 (0.06) | 0.02 | 0.07 (0.10) | 0.49 | -0.16 (0.08) | 0.04 | 1023 | 0.15 (0.10) | 0.13 | 0.008 (0.10) | 0.94 | 0.05 (0.13) | 0.70 | 0.35 (0.22) | 0.12 | -0.02 (0.10) | 0.85 | 0.31 (0.24) | 0.20 |
| *t*=fu | 961 | 0.35 (0.06) | <0.001 | -0.05 (0.10) | 0.66 | -0.06 (0.08) | 0.43 | 1023 | 0.50 (0.09) | <0.001 | -0.02 (0.10) | 0.88 | -0.001 (0.12) | 0.99 | 0.70 (0.15) | <0.001 | -0.03 (0.10) | 0.80 | 0.02 (0.17) | 0.89 |
| **IN1** | | | | | | | | | | | | | | | | | | | | |
| *t*=base | 1013 | -0.03 (0.07) | 0.63 | 0.03 (0.02) | 0.24 | 0.11 (0.08) | 0.16 | 1021 | 0.04 (0.14) | 0.77 | 0.03 (0.02) | 0.24 | -0.19 (0.15) | 0.20 | 0.30 (0.22) | 0.18 | 0.03 (0.02) | 0.27 | 0.15 (0.28) | 0.59 |
| *t*=fu | 1010 | 0.07 (0.06) | 0.31 | 0.03 (0.02) | 0.23 | 0.06 (0.08) | 0.47 | 1021 | -0.20 (0.13) | 0.11 | 0.03 (0.02) | 0.23 | 0.28 (0.18) | 0.12 | 0.21 (0.24) | 0.38 | 0.03 (0.02) | 0.24 | 0.12 (0.31) | 0.70 |
| **IN2** | | | | | | | | | | | | | | | | | | | | |
| *t*=base | 1015 | -0.004 (0.08) | 0.96 | 0.004 (0.01) | 0.78 | 0.02 (0.07) | 0.82 | 1023 | 0.20 (0.19) | 0.29 | 0.003 (0.01) | 0.86 | 0.26 (0.18) | 0.15 | 0.39 (0.57) | 0.49 | 0.003 (0.01) | 0.84 | 0.59 (0.37) | 0.11 |
| *t*=fu | 1012 | 0.16 (0.08) | 0.05 | 0.004 (0.02) | 0.77 | 0.03 (0.08) | 0.73 | 1023 | 0.14 (0.12) | 0.24 | 0.002 (0.01) | 0.91 | 0.12 (0.13) | 0.34 | 0.11 (0.27) | 0.67 | 0.002 (0.01) | 0.88 | 0.39 (0.21) | 0.06 |
| **IN3** | | | | | | | | | | | | | | | | | | | | |
| *t*=base | 1013 | 0.14 (0.07) | 0.03 | 0.02 (0.02) | 0.36 | -0.08 (0.09) | 0.37 | 1021 | 0.23 (0.11) | 0.05 | 0.02 (0.02) | 0.44 | -0.02 (0.12) | 0.89 | 0.36 (0.20) | 0.07 | 0.02 (0.02) | 0.35 | -0.10 (0.18) | 0.58 |
| *t*=fu | 1010 | 0.08 (0.05) | 0.14 | 0.02 (0.02) | 0.52 | -0.03 (0.06) | 0.62 | 1021 | -0.02 (0.10) | 0.85 | 0.02 (0.02) | 0.50 | -0.03 (0.14) | 0.82 | 0.21 (0.20) | 0.30 | 0.02 (0.02) | 0.44 | -0.14 (0.20) | 0.48 |
| **IN4** | | | | | | | | | | | | | | | | | | | | |
| *t*=base | 1012 | 0.04 (0.06) | 0.55 | 0.07 (0.03) | 0.03 | 0.08 (0.07) | 0.28 | 1021 | -0.03 (0.13) | 0.83 | 0.07 (0.03) | 0.03 | -0.07 (0.16) | 0.66 | 0.56 (0.32) | 0.08 | 0.07 (0.03) | 0.03 | -0.20 (0.35) | 0.57 |
| *t*=fu | 1010 | 0.07 (0.06) | 0.23 | 0.06 (0.03) | 0.04 | 0.15 (0.07) | 0.04 | 1021 | 0.14 (0.12) | 0.24 | 0.07 (0.03) | 0.03 | -0.07 (0.15) | 0.62 | 0.29 (0.23) | 0.22 | 0.06 (0.03) | 0.04 | 0.05 (0.25) | 0.84 |
| **IN5** | | | | | | | | | | | | | | | | | | | | |
| *t*=base | 1014 | 0.005 (0.06) | 0.93 | -0.04 (0.03) | 0.20 | -0.06 (0.08) | 0.42 | 1022 | -0.09 (0.11) | 0.43 | -0.04 (0.03) | 0.18 | -0.13 (0.15) | 0.38 | -0.13 (0.23) | 0.59 | -0.04 (0.03) | 0.20 | -0.08 (0.28) | 0.79 |
| *t*=fu | 1011 | 0.01 (0.06) | 0.81 | -0.04 (0.03) | 0.22 | -0.11 (0.08) | 0.15 | 1022 | 0.003 (0.12) | 0.98 | -0.04 (0.03) | 0.18 | -0.18 (0.15) | 0.23 | 0.10 (0.20) | 0.62 | -0.04 (0.03) | 0.19 | -0.16 (0.28) | 0.57 |
| **IN6** | | | | | | | | | | | | | | | | | | | | |
| *t*=base | 1014 | 0.22 (0.06) | <0.001 | -0.02 (0.03) | 0.53 | 0.07 (0.07) | 0.28 | 1022 | 0.19 (0.10) | 0.06 | -0.02 (0.03) | 0.62 | -0.04 (0.12) | 0.71 | 0.41 (0.17) | 0.01 | -0.01 (0.03) | 0.67 | 0.03 (0.19) | 0.86 |
| *t*=fu | 1011 | 0.24 (0.06) | <0.001 | -0.01 (0.03) | 0.69 | 0.02 (0.08) | 0.81 | 1022 | 0.25 (0.09) | 0.009 | -0.02 (0.03) | 0.62 | 0.09 (0.12) | 0.44 | 0.42 (0.16) | 0.01 | -0.01 (0.03) | 0.66 | 0.07 (0.18) | 0.70 |
| **IN7** | | | | | | | | | | | | | | | | | | | | |
[truncated: 256,451 more chars]
