# Supplementary material for: Moderators of peer influence effects for adolescents’ smoking and vaping norms and outcomes in high and middle-income settings
Source: Front Psychol. 2025 Nov 3;16:1655761. doi: 10.3389/fpsyg.2025.1655761 (PMC12620383; doi:10.3389/fpsyg.2025.1655761)
Supplement: SUPPLEMENTARY FILE 3 — Marginal effects, regions of significance, and changes in R-squared values for models with significant interactions. [file Table_3.docx]

Supplementary File 3

Moderators of peer influence effects for adolescents’ smoking and vaping norms and outcomes in high and middle-income settings.

**Jennifer M. Murray*, Sharon C. Sánchez-Franco, Olga L. Sarmiento, Erik O. Kimbrough, Christopher Tate, Shannon C. Montgomery, Rajnish Kumar, Laura Dunne, Abhijit Ramalingam, Erin L. Krupka, Felipe Montes, Huiyu Zhou, Laurence Moore, Linda Bauld, Blanca Llorente, Frank Kee, Ruth F. Hunter***

*** Correspondence:** Corresponding Authors: [jmurray39@qub.ac.uk](mailto:jmurray39@qub.ac.uk), [ruth.hunter@qub.ac.uk](mailto:ruth.hunter@qub.ac.uk)

**This file includes:**

Supplementary Tables S3.1. to S3.6. (marginal effects, regions of significance, and changes in R-squared values for models with significant interactions).

**
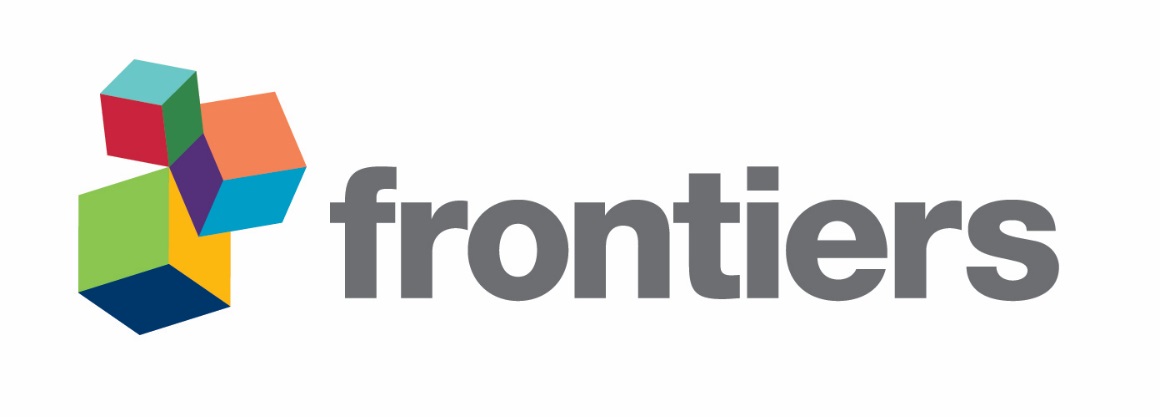
**

**Supplementary file 3: Marginal effects, regions of significance, and changes in R-squared values for models with significant interactions.**

**Table S3.1.** Marginal effects, regions of significance, and changes in R-squared values for models including dichotomous moderator variables (Setting, Intervention, and Gender).

| **Model details^a^** | | | **Moderator: Setting (0=NI, 1=Bogotá).** | | | | | | | | | | |
| --- | --- | --- | --- | --- | --- | --- | --- | --- | --- | --- | --- | --- | --- |
| **Dependent variable** | **Peer group (*-i*)** | **Time-point (*t*)** | **Marginal effects (NI)^b^** | | | | **Marginal effects (Bogotá)^c^** | | | | **Regions of significance^d^** | | **∆R^2^** |
|  |  |  | ***b* (SE)** | **p-value** | **95% CI** | **99% CI** | ***b* (SE)** | **p-value** | **95% CI** | **99% CI** | **95%** | **99%** |  |
| P2S2 (-1 to 1) | Class^i^ | fu | -0.48 (0.25) | 0.05 | -0.97, 0.002 | -1.12, 0.16 | 0.47 (0.14) | 0.001 | 0.18, 0.75 | 0.10, 0.84 | <-0.805 | <-0.828 | 0.0118 |
| P2S4 (-1 to 1) | Year | base | 0.21 (0.33) | 0.51 | -0.42, 0.85 | -0.62, 1.05 | 2.23 (0.67) | 0.001 | 0.91, 3.56 | 0.49, 3.97 | >-0.861 | >-0.854 | 0.0061 |
| P2S5 (-1 to 1) | Class | base | -0.01 (0.16) | 0.95 | -0.33, 0.31 | -0.43, 0.41 | 0.63 (0.15) | <0.001 | 0.34, 0.93 | 0.24, 1.03 | <-0.400 | None | 0.0066 |
|  | Class | fu | -0.16 (0.18) | 0.38 | -0.52, 0.20 | -0.64, 0.31 | 0.64 (0.11) | <0.001 | 0.43, 0.85 | 0.37, 0.91 | <-0.344  >-0.049 | <-0.386 | 0.0107 |
|  | Year | base | -0.19 (0.37) | 0.61 | -0.92, 0.54 | -1.15, 0.76 | 0.99 (0.24) | <0.001 | 0.52, 1.45 | 0.38, 1.60 | <-0.447 | None | 0.0051 |
| Donation to ASSIST/Dead Cool (0 to 10) | Year^l^ | base | -0.32 (0.24) | 0.18 | -0.79, 0.15 | -0.94, 0.30 | 1.93 (0.52) | <0.001 | 0.90, 2.96 | 0.58, 3.28 | <3.088  >3.835 | >3.896 | 0.0117 |
| IN1 (-2 to 2) | Friends | fu | -0.19 (0.09) | 0.04 | -0.36, -0.01 | -0.42, 0.04 | 0.17 (0.08) | 0.03 | 0.01, 0.32 | -0.03, 0.37 | <1.581 | None | 0.0057 |
|  | Year | fu | -0.84 (0.45) | 0.06 | -1.72, 0.04 | -2.00, 0.32 | 0.44 (0.26) | 0.08 | -0.06, 0.95 | -0.22, 1.10 | <1.657 | None | 0.0052 |
| DN1.1 (1 to 5) | Class | base | 0.41 (0.17) | 0.02 | 0.08, 0.75 | -0.03, 0.86 | -0.39 (0.18) | 0.03 | -0.74, -0.04 | -0.85, 0.07 | <4.830 | <4.792 | 0.0076 |
|  | Year | base | 1.12 (0.32) | 0.001 | 0.49, 1.75 | 0.29, 1.95 | -0.43 (0.55) | 0.43 | -1.51, 0.65 | -1.85, 0.99 | None | None | 0.0064 |
| DN1.3 (1 to 5) | Year | fu | 0.25 (0.18) | 0.18 | -0.11, 0.61 | -0.23, 0.72 | -0.83 (0.40) | 0.04 | -1.61, -0.05 | -1.86, 0.19 | <4.483 | <4.435 | 0.0022 |
| DN2.1 (1 to 5) | Year | fu | 0.25 (0.17) | 0.14 | -0.09, 0.59 | -0.19, 0.70 | -0.84 (0.38) | 0.03 | -1.59, -0.09 | -1.83, 0.15 | <4.590 | None | 0.0052 |
| Self-report descriptive norms 2 (Average DN2.1 to DN2.3; 1 to 5) | Year | fu | 0.46 (0.15) | 0.002 | 0.17, 0.75 | 0.08, 0.84 | -1.22 (0.48) | 0.01 | -2.17, -0.28 | -2.46, 0.02 | <4.644 | <4.627 | 0.0054 |
| Perceived physical risks (0 to 100%) | Year | fu | -0.31 (0.24) | 0.20 | -0.79, 0.16 | -0.94, 0.31 | -1.29 (0.42) | 0.003 | -2.12, -0.45 | -2.38, -0.19 | >62.445 | >63.373  <69.667 | 0.0039 |
| Perceived social risks (0 to 100%) | Class | base | -0.15 (0.13) | 0.22 | -0.40, 0.09 | -0.48, 0.17 | 0.27 (0.11) | 0.01 | 0.06, 0.49 | -0.01, 0.56 | <81.373 | <78.893 | 0.0032 |
| Perceived behavioral control (easy to quit; 1 to 5) | Class^l^ | fu | 0.40 (0.17) | 0.02 | 0.06, 0.74 | -0.04, 0.85 | -0.86 (0.26) | 0.001 | -1.37, -0.36 | -1.53, -0.20 | <3.525 | <3.435 | 0.0128 |
|  | Year^j^ | fu | -3.73 (0.89) | <0.001 | -5.48, -1.97 | -6.04, -1.42 | -1.17 (0.53) | 0.03 | -2.20, -0.13 | -2.53, 0.20 | All | All | 0.0058 |
| Perceived behavioral control (to avoid smoking; 1 to 5) | Friends | base | 0.27 (0.12) | 0.02 | 0.04, 0.51 | -0.04, 0.59 | -0.09 (0.09) | 0.28 | -0.26, 0.07 | -0.31, 0.13 | >3.854 | >3.976 | 0.0051 |
| Objectively measured smoking behavior (0 to 30 ppm) | Friends | fu | 0.74 (0.10) | <0.001 | 0.55, 0.93 | 0.49, 0.99 | 0.31 (0.09) | 0.001 | 0.12, 0.49 | 0.06, 0.55 | <3.423 | <3.217 | 0.0115 |
|  | Year^k,l^ | base | 0.86 (0.13) | <0.001 | 0.61, 1.12 | 0.52, 1.20 | -0.08 (0.14) | 0.58 | -0.36, 0.20 | -0.45, 0.29 | <2.710 | <2.526 | 0.0108 |
| **Dependent variable** | **Peer group (*-i*)** | **Time-point (*t*)** | **Moderator: Intervention (1=ASSIST, 2=Dead Cool).** | | | | | | | | | | |
|  |  |  | **Marginal effects (ASSIST)^b^** | | | | **Marginal effects (Dead Cool)^c^** | | | | **Regions of significance^d^** | | **∆R^2^** |
|  |  |  | ***b* (SE)** | **p-value** | **95% CI** | **99% CI** | ***b* (SE)** | **p-value** | **95% CI** | **99% CI** | **95%** | **99%** |  |
| P2S4 (-1 to 1) | Year | base | -0.43 (0.45) | 0.33 | -1.31, 0.45 | -1.59, 0.72 | 1.52 (0.47) | 0.001 | 0.60, 2.44 | 0.31, 2.73 | <-0.889 | None | 0.0079 |
| Donation to ASSIST/Dead Cool (0 to 10) | Friends | base | 0.30 (0.07) | <0.001 | 0.17, 0.44 | 0.12, 0.48 | -0.08 (0.09) | 0.40 | -0.26, 0.11 | -0.32, 0.16 | >4.360 | >5.298  <8.902 | 0.0098 |
|  | Year | base | 0.83 (0.27) | 0.002 | 0.30, 1.36 | 0.13, 1.53 | -0.41 (0.32) | 0.20 | -1.04, 0.21 | -1.24, 0.41 | >3.950 | None | 0.0077 |
| IN5 (-2 to 2) | Friends | fu | 0.18 (0.08) | 0.02 | 0.03, 0.33 | -0.02, 0.38 | -0.20 (0.09) | 0.02 | -0.38, -0.03 | -0.43, 0.03 | <1.205 | None | 0.0067 |
|  | Class | fu | 0.23 (0.14) | 0.10 | -0.04, 0.51 | -0.13, 0.60 | -0.36 (0.18) | 0.05 | -0.71, -0.003 | -0.82, 0.11 | None | None | 0.0039 |
| DN1.1 (1 to 5) | Friends | base | 0.37 (0.13) | 0.003 | 0.12, 0.61 | 0.04, 0.69 | -0.04 (0.10) | 0.67 | -0.24, 0.16 | -0.31, 0.22 | >2.605  <4.795 | None | 0.0045 |
|  | Class | base | 0.46 (0.17) | 0.008 | 0.12, 0.80 | 0.02, 0.91 | -0.40 (0.16) | 0.02 | -0.72, -0.08 | -0.82, 0.02 | <4.838 | <4.808 | 0.0071 |
| Attitudes (1 to 5) | Class | fu | -0.44 (0.19) | 0.02 | -0.81, -0.08 | -0.92, 0.04 | 0.18 (0.15) | 0.22 | -0.11, 0.47 | -0.20, 0.56 | >3.877 | >3.926  <4.465 | 0.0049 |
| Objectively measured smoking behavior (0 to 30 ppm) | Friends | base | 0.29 (0.07) | <0.001 | 0.16, 0.41 | 0.12, 0.46 | 0.47 (0.06) | <0.001 | 0.35, 0.60 | 0.31, 0.64 | >2.381  <5.823 | None | 0.0041 |
|  | Class | base | 0.26 (0.07) | <0.001 | 0.13, 0.39 | 0.09, 0.43 | 0.53 (0.06) | <0.001 | 0.41, 0.66 | 0.37, 0.70 | >2.550 | >3.092 | 0.0076 |
| **Dependent variable** | **Peer group (*-i*)** | **Time-point (*t*)** | **Moderator: Gender (0=boy, 1=girl/PNTS).** | | | | | | | | | | |
|  |  |  | **Marginal effects (boy)^b^** | | | | **Marginal effects (girl/PNTS)^c^** | | | | **Regions of significance^d^** | | **∆R^2^** |
|  |  |  | ***b* (SE)** | **p-value** | **95% CI** | **99% CI** | ***b* (SE)** | **p-value** | **95% CI** | **99% CI** | **95%** | **99%** |  |
| P2S9 (-1 to 1) | Class | fu | 0.08 (0.17) | 0.64 | -0.26, 0.42 | -0.36, 0.52 | 0.72 (0.13) | <0.001 | 0.46, 0.97 | 0.38, 1.05 | <-0.817 | None | 0.0090 |
| P3Q1 (-1 to 1) | Friends | fu | -0.11 (0.09) | 0.19 | -0.28, 0.05 | -0.34, 0.11 | 0.32 (0.08) | <0.001 | 0.16, 0.47 | 0.11, 0.52 | >-0.354 | >-0.291 | 0.0112 |
| P3Q2 (-1 to 1) | Friends | fu | -0.009 (0.09) | 0.92 | -0.19, 0.17 | -0.24, 0.23 | 0.33 (0.07) | <0.001 | 0.20, 0.46 | 0.16, 0.50 | >-0.197 | >-0.041  <0.551 | 0.0074 |
| Experiment Part 3: Descriptive norms (Average P3Q1 to P3Q2; -1 to 1) | Friends | fu | -0.08 (0.08) | 0.35 | -0.24, 0.09 | -0.29, 0.14 | 0.31 (0.07) | <0.001 | 0.17, 0.45 | 0.13, 0.49 | >-0.301 | >-0.233 | 0.0095 |
| IN1 (-2 to 2) | Year | base | 0.94 (0.28) | 0.001 | 0.39, 1.50 | 0.21, 1.67 | -0.18 (0.33) | 0.59 | -0.82, 0.46 | -1.02, 0.66 | >1.904 | None | 0.0049 |
| IN3 (-2 to 2) | Friends | base | 0.31 (0.11) | 0.006 | 0.09, 0.53 | 0.02, 0.61 | -0.008 (0.07) | 0.91 | -0.14, 0.12 | -0.18, 0.16 | None | None | 0.0050 |
| IN5 (-2 to 2) | Year | fu | 0.61 (0.31) | 0.05 | 0.005, 1.22 | -0.19, 1.41 | -0.33 (0.24) | 0.18 | -0.80, 0.15 | -0.95, 0.30 | None | None | 0.0039 |
| Self-report injunctive norms (Average IN1 to IN7; -2 to 2) | Year | base | 0.78 (0.24) | 0.001 | 0.31, 1.25 | 0.16, 1.40 | 0.02 (0.23) | 0.93 | -0.42, 0.47 | -0.56, 0.61 | None | None | 0.0040 |
|  | Year | fu | 0.65 (0.22) | 0.003 | 0.22, 1.07 | 0.09, 1.20 | -0.12 (0.20) | 0.54 | -0.51, 0.27 | -0.63, 0.39 | None | None | 0.0046 |
| DN2.2 (1 to 5) | Class | base | 0.07 (0.12) | 0.54 | -0.16, 0.30 | -0.23, 0.37 | 0.52 (0.14) | <0.001 | 0.25, 0.79 | 0.17, 0.88 | None | None | 0.0050 |
| DN2.3 (1 to 5) | Class | base | 0.36 (0.14) | 0.008 | 0.10, 0.63 | 0.01, 0.71 | 0.76 (0.12) | <0.001 | 0.54, 0.99 | 0.46, 1.06 | >4.105  <4.783 | None | 0.0043 |
|  | Class | fu | 0.20 (0.12) | 0.10 | -0.04, 0.44 | -0.11, 0.51 | 0.56 (0.10) | <0.001 | 0.36, 0.76 | 0.30, 0.82 | <4.755 | None | 0.0048 |
|  | Year | base | 0.33 (0.16) | 0.05 | 0.006, 0.65 | -0.10, 0.75 | 0.90 (0.17) | <0.001 | 0.57, 1.22 | 0.47, 1.32 | <4.792 | None | 0.0053 |
|  | Year | fu | 0.23 (0.15) | 0.12 | -0.06, 0.52 | -0.15, 0.62 | 0.78 (0.15) | <0.001 | 0.49, 1.07 | 0.40, 1.16 | <4.750 | None | 0.0069 |
| Self-report descriptive norms 2 (Average DN2.1 to DN2.3; 1 to 5) | Class | base | 0.12 (0.13) | 0.35 | -0.13, 0.37 | -0.21, 0.45 | 0.57 (0.13) | <0.001 | 0.32, 0.82 | 0.24, 0.90 | <4.627 | >4.433  <4.535 | 0.0057 |
| Intentions (1 to 6) | Friends | fu | 0.06 (0.08) | 0.43 | -0.09, 0.22 | -0.14, 0.27 | 0.46 (0.12) | <0.001 | 0.22, 0.70 | 0.14, 0.77 | None | None | 0.0106 |
| Perceived physical risks (0 to 100%) | Year | base | 0.50 (0.20) | 0.01 | 0.10, 0.89 | -0.02, 1.02 | -0.32 (0.18) | 0.07 | -0.66, 0.03 | -0.77, 0.13 | >61.443 | >63.600 | 0.0064 |
| Perceived addiction risks (0 to 100%) | Class | fu | 0.17 (0.12) | 0.15 | -0.06, 0.40 | -0.13, 0.47 | 0.56 (0.09) | <0.001 | 0.38, 0.74 | 0.32, 0.80 | >41.139 | None | 0.0059 |
| **Smoking susceptibility^e^** | | | **Moderator: Intervention (1=ASSIST, 2=Dead Cool).** | | | | | | | | | | |
| **Dependent variable** | **Peer group (*-i*)** | **Time-point (*t*)** | **Marginal effects (ASSIST)^f^** | | | | **Marginal effects (Dead Cool)^g^** | | | | **Regions of significance^h^** | | **∆R^2^** |
|  |  |  | **Pr (SE)** | **p-value** | **95% CI** | **99% CI** | **Pr (SE)** | **p-value** | **95% CI** | **99% CI** | **95%** | **99%** |  |
| Smoking susceptibility (1=yes; 0=no) | Year | base | 0.13 (0.04) | <0.001 | 0.06, 0.20 | 0.03, 0.22 | 0.02 (0.02) | 0.33 | -0.02, 0.05 | -0.03, 0.06 | >39.329% | None | 0.0044 |

*b*: unstandardized regression coefficient; SE: standard error; CI: confidence interval; ppm: parts per million; NI: Northern Ireland; PNTS: prefer not to say; Pr: probability.

^a^In each model the outcome variable is the focal participant’s (*i*) response to the relevant item at follow-up. The predictor variable is the average of the relevant peer group’s (*-i*) responses to the equivalent item at baseline (*t*=base) or follow-up (*t*=fu), where *–i*=focal participant's nominated friends; focal participant’s school class; focal participant’s school year group. The moderator, and interaction of the moderator with the predictor variable, were also included as independent variables in all models. All models include robust (Huber White) standard errors. The following baseline variables are included as covariates in all models: : gender (0=boy; 1=girl/prefer not to say), age (1=12 years or less; 2=13 years; 3=14 years or more), intervention (1=ASSIST; 2=Dead Cool), ethnicity (0=no ethnic minority; 1=ethnic minority), individuals' socio-economic status (NI: 1=NIMDM2017≤296.6; 2=296.6<NIMDM2017≤593.2; 3=NIMDM2017>593.2; Bogotá: 1=Informal settlement/Lowest/Low; 2=Middle-Low/Middle; 3=Middle-High/High), and baseline values of the outcome variable. The predictor variable and baseline values of the outcome variable were mean-centered.

^b^Marginal effects calculated using the Delta-method. Results are unstandardized regression coefficients representing the average change in the outcome variable for a one-unit increase in the predictor variable among participants who are at base values of the moderator variable (Setting [NI], Intervention [ASSIST], Gender [boy]).

^c^Marginal effects calculated using the Delta-method. Results are unstandardized regression coefficients representing the average change in the outcome variable for a one-unit increase in the predictor variable among participants at one unit above base values of the moderator variable (Setting [Bogotá], Intervention [Dead Cool], Gender [girl/PNTS]).

^d^Boundaries indicating regions of significance for dichotomous moderator variables (values of the predictor variable [non-centered] for which slopes are significantly different for different levels of the moderator variable: Bogotá versus NI, Dead Cool versus ASSIST, girl/PNTS versus boy) at the 95% and 99% confidence levels. "None" means there were no regions where the slopes differed significantly. "All" means the slopes differed significantly across the entire range of the predictor variable.

^e^Logistic regressions were run for models with focal participants' smoking susceptibility as the outcome variable, and robust (Huber White) standard errors. The predictor variable is the percentage of the relevant group (*-i*) classified as susceptible to commencing smoking at baseline (*t*=base) or follow-up (*t*=fu). The moderator, interaction of the moderator with the predictor variable, and baseline covariates, were also included as independent variables in all models. The predictor variable was mean-centered

^f^Marginal effects calculated using the Delta-method. Results are the average change in the predicted probability of being classified as susceptible to commencing smoking for a 10% increase in the number of nominated friends/pupils in the same school class/pupils in the same school year group classified as being susceptible to commencing smoking among participants who are at base values of the moderator variable (Setting [NI], Intervention [ASSIST], Gender [boy]).

^g^Marginal effects calculated using the Delta-method. Results are the average change in the predicted probability of being classified as susceptible to commencing smoking for a 10% increase in the number of nominated friends/pupils in the same school class/pupils in the same school year group classified as being susceptible to commencing smoking among participants at one unit above base values of the moderator variable (Setting [Bogotá], Intervention [Dead Cool], Gender [girl/PNTS]).

^h^Boundaries indicating regions of significance for dichotomous moderator variables (values of the predictor variable [non-centered] for which slopes are significantly different for different levels of the moderator variable: Bogotá versus NI, Dead Cool versus ASSIST, girl/PNTS versus boy) at the 95% and 99% confidence levels. "None" means there were no regions where the slopes differed significantly.

^i^For the model examining school class average, vif=4.32 for predictor, vif=2.10 for setting, vif=3.23 for interaction. Predictor coefficient is positive and significant without including setting or interaction.

^j^At least one IV has vif>100. Potentially problematic levels of multi-collinearity.

^k^At least one IV has variance inflation factor (vif)>20.

^l^Retained statistical significance at the 5% level after using the Holm-Bonferroni procedure to correct the p-values for multiple testing (p≤0.05; based on 276 tests of interaction effects carried out per moderator).

**Table S3.2.** Marginal effects, regions of significance, and changes in R-squared values for models including school socio-economic status as moderator variables.

| **Model details^a^** | | | **Moderator: School socio-economic status in all schools (1=lowest SES to 4=highest SES).** | | | | | | | | | | |
| --- | --- | --- | --- | --- | --- | --- | --- | --- | --- | --- | --- | --- | --- |
| **Dependent variable** | **Peer group (*-i*)** | **Time-point (*t*)** | **Marginal effects (low; SES=1.361)^b^** | | | | **Marginal effects (high; SES=3.090)^c^** | | | | **Regions of significance^d^** | | **∆R^2^** |
|  |  |  | ***b* (SE)** | **p-value** | **95% CI** | **99% CI** | ***b* (SE)** | **p-value** | **95% CI** | **99% CI** | **95%** | **99%** |  |
| P2S4 (-1 to 1) | Year | base | 0.47 (0.32) | 0.14 | -0.16, 1.10 | -0.35, 1.30 | 2.37 (0.61) | <0.001 | 1.16, 3.57 | 0.79, 3.94 | >1.498 | >1.691 | 0.0079 |
| P2S6 (-1 to 1) | Year | base | 0.88 (0.23) | <0.001 | 0.43, 1.33 | 0.29, 1.48 | -0.11 (0.27) | 0.67 | -0.65, 0.42 | -0.81, 0.59 | <2.260 | <2.078 | 0.0067 |
|  | Year | fu | 0.79 (0.19) | <0.001 | 0.41, 1.16 | 0.29, 1.28 | -0.61 (0.43) | 0.16 | -1.45, 0.24 | -1.72, 0.51 | <1.954 | <1.859 | 0.0049 |
| IN1 (-2 to 2) | Friends | base | -0.20 (0.07) | 0.006 | -0.35, -0.06 | -0.39, -0.01 | 0.30 (0.13) | 0.02 | 0.05, 0.56 | -0.03, 0.64 | <1.616  >2.767 | <1.436  >3.398 | 0.0095 |
|  | Friends | fu | -0.28 (0.09) | 0.001 | -0.45, -0.11 | -0.50, -0.06 | 0.29 (0.09) | 0.002 | 0.11, 0.47 | 0.05, 0.53 | <1.825  >2.629 | <1.658  >2.833 | 0.0132 |
|  | Class | fu | -0.49 (0.17) | 0.004 | -0.82, -0.15 | -0.93, -0.05 | 0.11 (0.15) | 0.47 | -0.19, 0.41 | -0.28, 0.50 | <2.085 | <1.723 | 0.0036 |
| IN4 (-2 to 2) | Class | base | 0.21 (0.14) | 0.12 | -0.05, 0.48 | -0.14, 0.57 | -0.32 (0.19) | 0.09 | -0.70, 0.05 | -0.82, 0.17 | <1.073  >3.539 | None | 0.0039 |
| IN5 (-2 to 2) | Friends | base | 0.19 (0.09) | 0.04 | 0.006, 0.37 | -0.05, 0.43 | -0.07 (0.07) | 0.34 | -0.21, 0.07 | -0.25, 0.12 | <1.456 | None | 0.0035 |
| Self-report injunctive norms (Average IN1 to IN7; -2 to 2) | Friends | fu | 0.02 (0.08) | 0.80 | -0.14, 0.18 | -0.19, 0.23 | 0.31 (0.07) | <0.001 | 0.17, 0.46 | 0.12, 0.51 | >1.934 | >2.106 | 0.0046 |
| DN1.1 (1 to 5) | Friends | base | 0.37 (0.13) | 0.006 | 0.11, 0.63 | 0.03, 0.71 | -0.22 (0.13) | 0.10 | -0.48, 0.04 | -0.56, 0.12 | <1.785  >3.266 | <1.487  >3.794 | 0.0160 |
|  | Class | base | 0.35 (0.17) | 0.04 | 0.03, 0.68 | -0.08, 0.79 | -0.22 (0.16) | 0.16 | -0.54, 0.09 | -0.63, 0.19 | <1.487  >3.581 | None | 0.0070 |
|  | Class | fu | 0.24 (0.14) | 0.08 | -0.03, 0.51 | -0.12, 0.60 | -0.46 (0.20) | 0.02 | -0.86, -0.06 | -0.99, 0.06 | <1.241  >2.790 | >3.656 | 0.0077 |
| DN1.5 (1 to 5) | Class | base | 0.47 (0.18) | 0.009 | 0.12, 0.83 | 0.005, 0.94 | -0.06 (0.09) | 0.53 | -0.24, 0.12 | -0.30, 0.18 | <2.189  >3.770 | <1.444 | 0.0045 |
|  | Class | fu | 0.34 (0.13) | 0.008 | 0.09, 0.60 | 0.009, 0.68 | -0.29 (0.14) | 0.04 | -0.57, -0.01 | -0.66, 0.08 | <1.712  >3.033 | <1.408  >3.595 | 0.0066 |
| Self-efficacy (Friends; 1 to 6) | Year | base | 0.16 (0.19) | 0.38 | -0.20, 0.53 | -0.31, 0.64 | 0.93 (0.31) | 0.002 | 0.33, 1.53 | 0.14, 1.72 | >1.769 | >2.094 | 0.0038 |
| **Dependent variable** | **Peer group (*-i*)** | **Time-point (*t*)** | **Moderator: School socio-economic status in NI schools (5.7=lowest SES [NIMDM2017=57] to 80.2=highest SES [NIMDM2017=802]).** | | | | | | | | | | |
|  |  |  | **Marginal effects (low; NIMDM2017=11.082)^b^** | | | | **Marginal effects (high; NIMDM2017=57.677)^c^** | | | | **Regions of significance^d^** | | **∆R^2^** |
|  |  |  | ***b* (SE)** | **p-value** | **95% CI** | **99% CI** | ***b* (SE)** | **p-value** | **95% CI** | **99% CI** | **95%** | **99%** |  |
| IN1 (-2 to 2) | Friends | base | -0.31 (0.12) | 0.01 | -0.54, -0.07 | -0.62, 0.001 | 0.35 (0.19) | 0.06 | -0.01, 0.72 | -0.13, 0.84 | <18.165  >59.816 | <10.899 | 0.0157 |
| IN5 (-2 to 2) | Friends | base | 0.32 (0.16) | 0.05 | 0.008, 0.63 | -0.09, 0.72 | -0.07 (0.11) | 0.54 | -0.27, 0.14 | -0.34, 0.21 | <13.207 | None | 0.0075 |
| DN1.1 (1 to 5) | Friends | base | 0.31 (0.19) | 0.11 | -0.07, 0.68 | -0.18, 0.79 | -0.32 (0.15) | 0.04 | -0.62, -0.02 | -0.72, 0.08 | >55.973 | >66.807 | 0.0110 |
| Self-report smoking behavior (1 to 4) | Friends | fu | 0.42 (0.17) | 0.01 | 0.09, 0.76 | -0.02, 0.87 | -0.07 (0.12) | 0.56 | -0.30, 0.16 | -0.37, 0.23 | <26.534 | None | 0.0136 |
| Self-efficacy (Emotional; 1 to 6) | Friends | fu | 0.61 (0.18) | 0.001 | 0.26, 0.96 | 0.15, 1.07 | -0.29 (0.20) | 0.14 | -0.68, 0.09 | -0.80, 0.21 | <32.316  >70.050 | <28.589 | 0.0142 |
| Self-efficacy (Friends; 1 to 6) | Friends | fu | 0.47 (0.18) | 0.007 | 0.13, 0.82 | 0.02, 0.93 | -0.50 (0.19) | 0.009 | -0.87, -0.12 | -0.99, -0.004 | <22.071  >46.527 | <13.551  >57.019 | 0.0188 |
| Self-efficacy (Opportunity; 1 to 6) | Friends | fu | 0.30 (0.17) | 0.07 | -0.03, 0.63 | -0.13, 0.73 | -0.51 (0.19) | 0.009 | -0.89, -0.13 | -1.01, -0.006 | <6.970  >41.008 | >55.437 | 0.0126 |
| Perceived physical risks (0 to 100%) | Friends | base | 0.36 (0.12) | 0.003 | 0.12, 0.60 | 0.05, 0.68 | 0.02 (0.08) | 0.78 | -0.14, 0.19 | -0.19, 0.24 | <39.627 | <29.032 | 0.0061 |
| **Dependent variable** | **Peer group (*-i*)** | **Time-point (*t*)** | **Moderator: School socio-economic status in Bogotá schools (1=lowest SES [Lower] to 4=highest SES [Higher]).** | | | | | | | | | | |
|  |  |  | **Marginal effects (low; SES=2.022)^b^** | | | | **Marginal effects (high; SES=3.022)^c^** | | | | **Regions of significance^d^** | | **∆R^2^** |
|  |  |  | ***b* (SE)** | **p-value** | **95% CI** | **99% CI** | ***b* (SE)** | **p-value** | **95% CI** | **99% CI** | **95%** | **99%** |  |
| IN1 (-2 to 2) | Friends | fu | -0.11 (0.11) | 0.31 | -0.31, 0.10 | -0.38, 0.17 | 0.34 (0.12) | 0.004 | 0.11, 0.57 | 0.03, 0.64 | >2.606 | >2.798 | 0.0096 |
| Self-report descriptive norms 1 (Average DN1.1 to DN1.5; 1 to 5) | Friends | base | -0.05 (0.07) | 0.52 | -0.19, 0.10 | -0.24, 0.14 | 0.27 (0.09) | 0.004 | 0.09, 0.45 | 0.03, 0.51 | >2.551 | >2.753 | 0.0058 |
| Objectively measured smoking behavior (0 to 30 ppm) | Friends | base | 0.20 (0.15) | 0.18 | -0.09, 0.48 | -0.18, 0.57 | -0.44 (0.16) | 0.007 | -0.76, -0.12 | -0.86, -0.02 | >2.726 | >2.945 | 0.0184 |
|  | Friends | fu | 0.15 (0.08) | 0.06 | -0.005, 0.30 | -0.05, 0.35 | 0.58 (0.10) | <0.001 | 0.38, 0.77 | 0.32, 0.83 | >2.031 | >2.107 | 0.0214 |

*b*: unstandardized regression coefficient; SE: standard error; CI: confidence interval; ppm: parts per million; SES: socio-economic status.

^a^In each model the outcome variable is the focal participant’s (*i*) response to the relevant item at follow-up. The predictor variable is the average of the relevant peer group’s (*-i*) responses to the equivalent item at baseline (*t*=base) or follow-up (*t*=fu), where *–i*=focal participant's nominated friends; focal participant’s school class; focal participant’s school year group. The moderator, and interaction of the moderator with the predictor variable, were also included as independent variables in all models. All models include robust (Huber White) standard errors. The following baseline variables are included as covariates in all models: : gender (0=boy; 1=girl/prefer not to say), age (1=12 years or less; 2=13 years; 3=14 years or more), intervention (1=ASSIST; 2=Dead Cool), ethnicity (0=no ethnic minority; 1=ethnic minority), and baseline values of the outcome variable. The predictor variable, baseline values of the outcome variable, and continuous moderator variables were mean-centered.

^b^Marginal effects calculated using the Delta-method. Results are unstandardized regression coefficients representing the average change in the outcome variable for a one-unit increase in the predictor variable among participants who are one standard deviation below the mean on the moderator variable ('low' values of continuous moderator variables).

^c^Marginal effects calculated using the Delta-method. Results are unstandardized regression coefficients representing the average change in the outcome variable for a one-unit increase in the predictor variable among participants who are one standard deviation above the mean on the moderator variable ('high' values of continuous moderator variables).

^d^Boundaries indicating regions of significance (values of the continuous moderator variable [non-centered] for which the conditional effect of the predictor variable on the outcome variable differs significantly from 0) at the 95% and 99% confidence levels, calculated using the Johnson-Neyman technique. "None" means there were no regions where the conditional effect differed significantly from 0. "All" means the conditional effect differed significantly from 0 across the entire range of the moderator variable.

**Table S3.3.** Marginal effects, regions of significance, and changes in R-squared values for models including norm sensitivities and related personality characteristics as moderator variables.

| **Model details^a^** | | | **Moderator: Norms sensitivities/Rule-following (0=least RF to 5=most RF).** | | | | | | | | | | |
| --- | --- | --- | --- | --- | --- | --- | --- | --- | --- | --- | --- | --- | --- |
| **Dependent variable** | **Peer group (*-i*)** | **Time-point (*t*)** | **Marginal effects (low; RF=1.299)^b^** | | | | **Marginal effects (high; RF=4.900)^c^** | | | | **Regions of significance^d^** | | **∆R^2^** |
|  |  |  | ***b* (SE)** | **p-value** | **95% CI** | **99% CI** | ***b* (SE)** | **p-value** | **95% CI** | **99% CI** | **95%** | **99%** |  |
| P2S8 (-1 to 1) | Class | base | 0.13 (0.11) | 0.27 | -0.10, 0.35 | -0.17, 0.42 | 0.51 (0.12) | <0.001 | 0.28, 0.74 | 0.21, 0.82 | >1.918 | >2.305 | 0.0045 |
| Perceived physical risks (0 to 100%) | Year | base | 0.49 (0.20) | 0.02 | 0.09, 0.89 | -0.04, 1.02 | -0.29 (0.18) | 0.11 | -0.64, 0.06 | -0.75, 0.17 | <2.032 | <0.820 | 0.0061 |
|  | Year | fu | 0.41 (0.27) | 0.13 | -0.12, 0.95 | -0.28, 1.11 | -0.47 (0.25) | 0.06 | -0.96, 0.01 | -1.11, 0.16 | <0.416  >4.968 | None | 0.0049 |
| Perceived behavioral control (easy to quit; 1 to 5) | Year | fu | 0.39 (0.14) | 0.004 | 0.12, 0.66 | 0.04, 0.75 | 0.83 (0.12) | <0.001 | 0.59, 1.06 | 0.52, 1.13 | >0.650 | >1.112 | 0.0058 |
| Perceived behavioral control (to avoid smoking; 1 to 5) | Class | base | 0.75 (0.20) | <0.001 | 0.36, 1.14 | 0.24, 1.27 | 0.04 (0.17) | 0.81 | -0.30, 0.38 | -0.40, 0.49 | <3.733 | <3.326 | 0.0067 |
| **Dependent variable** | **Peer group (*-i*)** | **Time-point (*t*)** | **Moderator: Pro-sociality (0=least pro-sociality to 10=most pro-sociality).** | | | | | | | | | | |
|  |  |  | **Marginal effects (low; PS=5.576)^b^** | | | | **Marginal effects (high; PS=9.842)^c^** | | | | **Regions of significance^d^** | | **∆R^2^** |
|  |  |  | ***b* (SE)** | **p-value** | **95% CI** | **99% CI** | ***b* (SE)** | **p-value** | **95% CI** | **99% CI** | **95%** | **99%** |  |
| P2S6 (-1 to 1) | Year | fu | 0.07 (0.25) | 0.79 | -0.42, 0.56 | -0.58, 0.71 | 0.84 (0.18) | <0.001 | 0.48, 1.20 | 0.37, 1.31 | >7.079 | >7.478 | 0.0049 |
| P2S7 (-1 to 1) | Year | fu | -0.22 (0.25) | 0.38 | -0.70, 0.26 | -0.85, 0.41 | 0.78 (0.20) | <0.001 | 0.38, 1.18 | 0.25, 1.30 | <3.364  >7.917 | >8.358 | 0.0083 |
| P2S9 (-1 to 1) | Friends | fu | 0.40 (0.09) | <0.001 | 0.22, 0.58 | 0.17, 0.63 | 0.08 (0.09) | 0.36 | -0.09, 0.25 | -0.14, 0.30 | <9.013 | <8.593 | 0.0071 |
| Experiment Part 2: Injunctive norms (Average P2S2 to P2S9; -1 to 1) | Year | base | 0.01 (0.22) | 0.96 | -0.42, 0.44 | -0.55, 0.57 | 0.81 (0.20) | <0.001 | 0.42, 1.20 | 0.30, 1.33 | >7.132 | >7.541 | 0.0049 |
|  | Year | fu | -0.08 (0.21) | 0.69 | -0.50, 0.33 | -0.63, 0.46 | 0.68 (0.19) | <0.001 | 0.31, 1.05 | 0.19, 1.17 | >7.598 | >8.062 | 0.0054 |
| IN4 (-2 to 2) | Class | fu | 0.53 (0.17) | 0.001 | 0.21, 0.86 | 0.10, 0.96 | -0.15 (0.15) | 0.31 | -0.44, 0.14 | -0.53, 0.23 | <7.474 | <6.844 | 0.0064 |
| DN2.1 (1 to 5) | Class | base | -0.08 (0.13) | 0.50 | -0.33, 0.16 | -0.41, 0.24 | 0.43 (0.16) | 0.007 | 0.12, 0.74 | 0.02, 0.84 | <2.110  >8.024 | >9.215 | 0.0052 |
| Self-report descriptive norms 2 (Average DN2.1 to DN2.3; 1 to 5) | Friends | fu | 0.003 (0.07) | 0.97 | -0.14, 0.14 | -0.18, 0.18 | 0.24 (0.08) | 0.002 | 0.09, 0.39 | 0.04, 0.44 | >7.562 | >8.351 | 0.0045 |
|  | Class | base | 0.13 (0.13) | 0.31 | -0.12, 0.38 | -0.20, 0.45 | 0.61 (0.14) | <0.001 | 0.33, 0.89 | 0.24, 0.98 | >6.309 | >6.735 | 0.0057 |
| Intentions (1 to 6) | Friends | base | 0.02 (0.10) | 0.86 | -0.18, 0.22 | -0.24, 0.28 | 0.34 (0.11) | 0.002 | 0.13, 0.56 | 0.06, 0.63 | >7.489 | >8.288 | 0.0044 |
| Self-efficacy (Opportunity; 1 to 6) | Year | fu | 0.65 (0.24) | 0.007 | 0.17, 1.12 | 0.02, 1.27 | -0.16 (0.21) | 0.45 | -0.58, 0.26 | -0.71, 0.39 | <7.117 | <5.952 | 0.0053 |
| Perceived physical risks (0 to 100%) | Friends | base | -0.05 (0.07) | 0.47 | -0.18, 0.08 | -0.22, 0.12 | 0.22 (0.07) | 0.001 | 0.09, 0.35 | 0.05, 0.39 | <3.149  >7.999 | >8.642 | 0.0065 |
| Perceived social risks (0 to 100%) | Class | fu | 0.05 (0.13) | 0.69 | -0.20, 0.30 | -0.28, 0.38 | 0.40 (0.12) | 0.001 | 0.17, 0.63 | 0.10, 0.70 | >7.146 | >7.686 | 0.0038 |
| Perceived addiction risks (0 to 100%) | Friends | base | 0.10 (0.07) | 0.16 | -0.04, 0.23 | -0.08, 0.28 | 0.37 (0.08) | <0.001 | 0.20, 0.53 | 0.15, 0.58 | >6.010 | >6.463 | 0.0052 |
| **Dependent variable** | **Peer group (*-i*)** | **Time-point (*t*)** | **Moderator: Fear of negative evaluation (1=least FNE to 5=most FNE).** | | | | | | | | | | |
|  |  |  | **Marginal effects (low; FNE=2.071)^b^** | | | | **Marginal effects (high; FNE=3.407)^c^** | | | | **Regions of significance^d^** | | **∆R^2^** |
|  |  |  | ***b* (SE)** | **p-value** | **95% CI** | **99% CI** | ***b* (SE)** | **p-value** | **95% CI** | **99% CI** | **95%** | **99%** |  |
| P2S6 (-1 to 1) | Friends | base | 0.005 (0.10) | 0.96 | -0.19, 0.20 | -0.25, 0.26 | 0.36 (0.09) | <0.001 | 0.18, 0.53 | 0.13, 0.59 | >2.570 | >2.699 | 0.0057 |
|  | Year | base | 0.004 (0.24) | 0.99 | -0.46, 0.47 | -0.61, 0.62 | 0.83 (0.24) | 0.001 | 0.35, 1.30 | 0.20, 1.45 | >2.672 | >2.871 | 0.0062 |
| DN1.3 (1 to 5) | Year | fu | -0.31 (0.19) | 0.11 | -0.68, 0.07 | -0.80, 0.19 | 0.23 (0.16) | 0.15 | -0.08, 0.55 | -0.18, 0.65 | <1.689  >3.774 | None | 0.0027 |
| Self-report descriptive norms 1 (Average DN1.1 to DN1.5; 1 to 5) | Class | base | -0.15 (0.11) | 0.16 | -0.37, 0.06 | -0.43, 0.13 | 0.19 (0.08) | 0.02 | 0.03, 0.36 | -0.02, 0.41 | <1.488  >3.241 | >3.598 | 0.0033 |
|  | Class | fu | -0.22 (0.11) | 0.06 | -0.44, 0.01 | -0.51, 0.08 | 0.18 (0.08) | 0.03 | 0.02, 0.34 | -0.03, 0.39 | <2.005  >3.329 | <1.076  >3.613 | 0.0041 |
| Self-report smoking behavior (1 to 4) | Friends | fu | 0.08 (0.06) | 0.21 | -0.04, 0.20 | -0.08, 0.23 | 0.46 (0.11) | <0.001 | 0.24, 0.67 | 0.17, 0.74 | >2.193 | >2.294 | 0.0098 |
| Self-efficacy (Emotional; 1 to 6) | Friends | fu | 0.13 (0.07) | 0.09 | -0.02, 0.27 | -0.07, 0.32 | 0.48 (0.12) | <0.001 | 0.24, 0.72 | 0.17, 0.79 | >2.128 | >2.247 | 0.0089 |
| Perceived behavioral control (easy to quit; 1 to 5) | Friends | base | 0.14 (0.08) | 0.06 | -0.008, 0.30 | -0.06, 0.34 | 0.38 (0.07) | <0.001 | 0.24, 0.52 | 0.20, 0.56 | >2.102 | >2.269 | 0.0046 |
| **Dependent variable** | **Peer group (*-i*)** | **Time-point (*t*)** | **Moderator: Need to belong (1=least NTB to 5=most NTB).** | | | | | | | | | | |
|  |  |  | **Marginal effects (low; NTB=2.326)^b^** | | | | **Marginal effects (high; NTB=3.580)^c^** | | | | **Regions of significance^d^** | | **∆R^2^** |
|  |  |  | ***b* (SE)** | **p-value** | **95% CI** | **99% CI** | ***b* (SE)** | **p-value** | **95% CI** | **99% CI** | **95%** | **99%** |  |
| P2S9 (-1 to 1) | Class | base | 0.86 (0.19) | <0.001 | 0.48, 1.23 | 0.36, 1.35 | 0.18 (0.17) | 0.30 | -0.16, 0.51 | -0.26, 0.62 | <3.383 | <3.271 | 0.0065 |
| IN7 (-2 to 2) | Friends | base | -0.01 (0.07) | 0.86 | -0.15, 0.13 | -0.20, 0.17 | 0.26 (0.09) | 0.003 | 0.09, 0.43 | 0.03, 0.48 | >2.918 | >3.170 | 0.0041 |
| DN1.2 (1 to 5) | Friends | base | -0.01 (0.07) | 0.86 | -0.15, 0.12 | -0.19, 0.17 | 0.26 (0.07) | <0.001 | 0.12, 0.39 | 0.08, 0.43 | >2.837 | >2.968 | 0.0038 |
| Self-report smoking behavior (1 to 4) | Friends | fu | 0.14 (0.07) | 0.04 | 0.006, 0.27 | -0.04, 0.31 | 0.45 (0.11) | <0.001 | 0.24, 0.67 | 0.17, 0.74 | >2.309 | >2.428 | 0.0069 |
| Self-efficacy (Emotional; 1 to 6) | Friends | fu | 0.12 (0.08) | 0.11 | -0.03, 0.27 | -0.08, 0.32 | 0.51 (0.11) | <0.001 | 0.29, 0.73 | 0.22, 0.80 | >2.398 | >2.510 | 0.0099 |
| Self-efficacy (Opportunity; 1 to 6) | Friends | fu | 0.05 (0.06) | 0.45 | -0.08, 0.17 | -0.12, 0.21 | 0.42 (0.13) | 0.001 | 0.17, 0.68 | 0.09, 0.76 | <1.119  >2.567 | >2.732 | 0.0096 |

*b*: unstandardized regression coefficient; SE: standard error; CI: confidence interval; ppm: parts per million; RF: Rule-following; PS: Pro-sociality; FNE: Fear of negative evaluation; NTB: Need to belong.

^a^In each model the outcome variable is the focal participant’s (*i*) response to the relevant item at follow-up. The predictor variable is the average of the relevant peer group’s (*-i*) responses to the equivalent item at baseline (*t*=base) or follow-up (*t*=fu), where *–i*=focal participant's nominated friends; focal participant’s school class; focal participant’s school year group. The moderator, and interaction of the moderator with the predictor variable, were also included as independent variables in all models. All models include robust (Huber White) standard errors. The following baseline variables are included as covariates in all models: : gender (0=boy; 1=girl/prefer not to say), age (1=12 years or less; 2=13 years; 3=14 years or more), intervention (1=ASSIST; 2=Dead Cool), ethnicity (0=no ethnic minority; 1=ethnic minority), individuals' socio-economic status (NI: 1=NIMDM2017≤296.6; 2=296.6<NIMDM2017≤593.2; 3=NIMDM2017>593.2; Bogotá: 1=Informal settlement/Lowest/Low; 2=Middle-Low/Middle; 3=Middle-High/High), and baseline values of the outcome variable. The predictor variable, baseline values of the outcome variable, and continuous moderator variables were mean-centered.

^b^Marginal effects calculated using the Delta-method. Results are unstandardized regression coefficients representing the average change in the outcome variable for a one-unit increase in the predictor variable among participants who are one standard deviation below the mean on the moderator variable ('low' values of continuous moderator variables).

^c^Marginal effects calculated using the Delta-method. Results are unstandardized regression coefficients representing the average change in the outcome variable for a one-unit increase in the predictor variable among participants who are one standard deviation above the mean on the moderator variable ('high' values of continuous moderator variables).

^d^Boundaries indicating regions of significance (values of the continuous moderator variable [non-centered] for which the conditional effect of the predictor variable on the outcome variable differs significantly from 0) at the 95% and 99% confidence levels, calculated using the Johnson-Neyman technique. "None" means there were no regions where the conditional effect differed significantly from 0. "All" means the conditional effect differed significantly from 0 across the entire range of the moderator variable.

**Table S3.4.** Marginal effects, regions of significance, and changes in R-squared values for models including the 'Big Five' personality traits as moderator variables.

| **Model details^a^** | | | **Moderator: Openness (0=least openness to 4=most openness).** | | | | | | | | | | |
| --- | --- | --- | --- | --- | --- | --- | --- | --- | --- | --- | --- | --- | --- |
| **Dependent variable** | **Peer group (*-i*)** | **Time-point (*t*)** | **Marginal effects (low; Openness=1.882)** | | | | **Marginal effects (high; Openness=3.253)** | | | | **Regions of significance** | | **∆R^2^** |
|  |  |  | ***b* (SE)** | **p-value** | **95% CI** | **99% CI** | ***b* (SE)** | **p-value** | **95% CI** | **99% CI** | **95%** | **99%** |  |
| P2S6 (-1 to 1) | Year | Base | 0.04 (0.26) | 0.86 | -0.46, 0.55 | -0.62, 0.71 | 0.86 (0.24) | <0.001 | 0.39, 1.34 | 0.24, 1.48 | >2.442 | >2.620 | 0.0057 |
|  | Year | fu | 0.06 (0.23) | 0.80 | -0.39, 0.51 | -0.53, 0.65 | 0.94 (0.19) | <0.001 | 0.57, 1.32 | 0.45, 1.44 | >2.307 | >2.426 | 0.0063 |
| P2S7 (-1 to 1) | Year | fu | -0.13 (0.24) | 0.59 | -0.61, 0.35 | -0.76, 0.50 | 0.69 (0.21) | 0.001 | 0.28, 1.10 | 0.15, 1.23 | >2.652 | >2.841 | 0.0060 |
| P2S8 (-1 to 1) | Friends | base | 0.16 (0.08) | 0.03 | 0.01, 0.32 | -0.03, 0.36 | 0.46 (0.08) | <0.001 | 0.31, 0.61 | 0.26, 0.65 | >1.840 | >1.977 | 0.0051 |
| Experiment Part 2: Injunctive norms (Average P2S2 to P2S9; -1 to 1) | Friends | base | 0.06 (0.08) | 0.50 | -0.11, 0.22 | -0.16, 0.27 | 0.40 (0.09) | <0.001 | 0.23, 0.57 | 0.18, 0.62 | >2.172 | >2.284 | 0.0060 |
|  | Year | base | 0.09 (0.21) | 0.67 | -0.32, 0.50 | -0.45, 0.63 | 0.85 (0.21) | <0.001 | 0.44, 1.26 | 0.32, 1.39 | >2.288 | >2.425 | 0.0045 |
| **Dependent variable** | **Peer group (*-i*)** | **Time-point (*t*)** | **Moderator: Extraversion (0=least extraverted to 4=most extraverted).** | | | | | | | | | | |
|  |  |  | **Marginal effects (low; Extraversion=1.886)** | | | | **Marginal effects (high; Extraversion=3.376)** | | | | **Regions of significance** | | **∆R^2^** |
|  |  |  | ***b* (SE)** | **p-value** | **95% CI** | **99% CI** | ***b* (SE)** | **p-value** | **95% CI** | **99% CI** | **95%** | **99%** |  |
| P2S5 (-1 to 1) | Friends | fu | 0.04 (0.08) | 0.61 | -0.12, 0.20 | -0.16, 0.25 | 0.34 (0.08) | <0.001 | 0.17, 0.50 | 0.12, 0.56 | >2.298 | >2.452 | 0.0060 |
|  | Class | base | 0.10 (0.14) | 0.49 | -0.18, 0.37 | -0.27, 0.46 | 0.59 (0.13) | <0.001 | 0.32, 0.85 | 0.24, 0.93 | >2.260 | >2.410 | 0.0055 |
|  | Class | fu | 0.20 (0.13) | 0.14 | -0.07, 0.46 | -0.15, 0.54 | 0.75 (0.12) | <0.001 | 0.50, 0.99 | 0.43, 1.07 | >2.007 | >2.139 | 0.0087 |
| P2S9 (-1 to 1) | Friends | base | -0.04 (0.10) | 0.69 | -0.24, 0.16 | -0.30, 0.22 | 0.34 (0.10) | 0.001 | 0.15, 0.53 | 0.09, 0.59 | >2.565 | >2.729 | 0.0068 |
|  | Class | base | 0.13 (0.17) | 0.43 | -0.20, 0.46 | -0.30, 0.56 | 0.86 (0.21) | <0.001 | 0.46, 1.26 | 0.33, 1.39 | >2.171 | >2.301 | 0.0076 |
|  | Year | base | -0.06 (0.30) | 0.83 | -0.65, 0.52 | -0.84, 0.71 | 1.21 (0.35) | 0.001 | 0.53, 1.89 | 0.31, 2.11 | <0.261  >2.476 | >2.647 | 0.0077 |
|  | Year | fu | -0.42 (0.28) | 0.14 | -0.98, 0.13 | -1.15, 0.31 | 0.74 (0.31) | 0.02 | 0.12, 1.35 | -0.07, 1.55 | <1.505  >3.068 | >3.766 | 0.0077 |
| Intentions (1 to 6) | Class | base | 0.89 (0.23) | <0.001 | 0.45, 1.34 | 0.31, 1.48 | 0.04 (0.17) | 0.81 | -0.28, 0.36 | -0.39, 0.47 | <2.972 | <2.828 | 0.0086 |
| **Dependent variable** | **Peer group (*-i*)** | **Time-point (*t*)** | **Moderator: Agreeableness (0=least agreeable to 4=most agreeable).** | | | | | | | | | | |
|  |  |  | **Marginal effects (low; Agreeableness=1.925)** | | | | **Marginal effects (high; Agreeableness=3.225)** | | | | **Regions of significance** | | **∆R^2^** |
|  |  |  | ***b* (SE)** | **p-value** | **95% CI** | **99% CI** | ***b* (SE)** | **p-value** | **95% CI** | **99% CI** | **95%** | **99%** |  |
| P2S9 (-1 to 1) | Friends | fu | 0.41 (0.11) | <0.001 | 0.21, 0.62 | 0.14, 0.68 | 0.09 (0.08) | 0.29 | -0.07, 0.25 | -0.13, 0.30 | <3.011 | <2.876 | 0.0062 |
| IN1 (-2 to 2) | Class | fu | -0.59 (0.21) | 0.004 | -0.99, -0.19 | -1.12, -0.06 | 0.17 (0.17) | 0.33 | -0.17, 0.51 | -0.28, 0.62 | <2.481  >3.944 | <2.226 | 0.0063 |
| Self-report smoking behavior (1 to 4) | Year | base | 0.95 (0.23) | <0.001 | 0.50, 1.40 | 0.36, 1.55 | 0.31 (0.17) | 0.07 | -0.02, 0.64 | -0.12, 0.74 | <3.193 | <3.029 | 0.0203 |
|  | Year | fu | 0.66 (0.16) | <0.001 | 0.34, 0.98 | 0.24, 1.08 | 0.22 (0.10) | 0.03 | 0.02, 0.42 | -0.04, 0.48 | <3.267 | <3.128 | 0.0028 |
| **Dependent variable** | **Peer group (*-i*)** | **Time-point (*t*)** | **Moderator: Conscientiousness (0=least conscientious to 4=most conscientious).** | | | | | | | | | | |
|  |  |  | **Marginal effects (low; Conscientiousness=1.683)** | | | | **Marginal effects (high; Conscientiousness=2.997)** | | | | **Regions of significance** | | **∆R^2^** |
|  |  |  | ***b* (SE)** | **p-value** | **95% CI** | **99% CI** | ***b* (SE)** | **p-value** | **95% CI** | **99% CI** | **95%** | **99%** |  |
| Self-report smoking behavior (1 to 4) | Year | fu | 0.68 (0.17) | <0.001 | 0.36, 1.01 | 0.25, 1.11 | 0.24 (0.09) | 0.009 | 0.06, 0.43 | 0.005, 0.48 | <3.143 | <3.008 | 0.0029 |
| Attitudes (1 to 5) | Year | fu | 0.79 (0.23) | 0.001 | 0.33, 1.24 | 0.19, 1.38 | -0.32 (0.24) | 0.17 | -0.78, 0.14 | -0.93, 0.29 | <2.200  >3.275 | <2.038  >3.796 | 0.0093 |
| Self-efficacy (Emotional; 1 to 6) | Class | fu | 0.44 (0.14) | 0.001 | 0.17, 0.72 | 0.09, 0.80 | 0.03 (0.10) | 0.74 | -0.16, 0.22 | -0.21, 0.28 | <2.547 | <2.319 | 0.0036 |
| Self-efficacy (Opportunity; 1 to 6) | Class | fu | 0.45 (0.16) | 0.004 | 0.15, 0.76 | 0.05, 0.86 | -0.00003 (0.10) | 1.00 | -0.20, 0.20 | -0.26, 0.26 | <2.407 | <2.071 | 0.0038 |
| **Dependent variable** | **Peer group (*-i*)** | **Time-point (*t*)** | **Moderator: Emotional stability (0=least stable to 4=most stable).** | | | | | | | | | | |
|  |  |  | **Marginal effects (low; Stability=1.231)** | | | | **Marginal effects (high; Stability=2.755)** | | | | **Regions of significance** | | **∆R^2^** |
|  |  |  | ***b* (SE)** | **p-value** | **95% CI** | **99% CI** | ***b* (SE)** | **p-value** | **95% CI** | **99% CI** | **95%** | **99%** |  |
| Attitudes (1 to 5) | Year | fu | 0.66 (0.21) | 0.001 | 0.25, 1.07 | 0.13, 1.20 | -0.30 (0.23) | 0.20 | -0.75, 0.16 | -0.89, 0.30 | <1.749  >3.186 | <1.541 | 0.0082 |
| **Smoking susceptibility^b^** | | | **Moderator: Extraversion (0=least extraverted to 4=most extraverted).** | | | | | | | | | | |
| **Dependent variable** | **Peer group (*-i*)** | **Time-point (*t*)** | **Marginal effects (low; Extraversion=1.886)^c^** | | | | **Marginal effects (high; Extraversion=3.376)^d^** | | | | **Regions of significance^e^** | | **∆R^2^** |
|  |  |  | **Pr (SE)** | **p-value** | **95% CI** | **99% CI** | **Pr (SE)** | **p-value** | **95% CI** | **99% CI** | **95%** | **99%** |  |
| Smoking susceptibility 1=yes; 0=no) | Year | base | 0.08 (0.03) | 0.002 | 0.03, 0.13 | 0.01, 0.14 | -0.007 (0.02) | 0.75 | -0.05, 0.04 | -0.06, 0.05 | <2.647 | <2.391 | 0.0049 |
|  | Year | fu | 0.08 (0.02) | <0.001 | 0.04, 0.11 | 0.03, 0.12 | 0.01 (0.02) | 0.60 | -0.03, 0.05 | -0.04, 0.06 | <2.953 | <2.794 | 0.0055 |

*b*: unstandardized regression coefficient; SE: standard error; CI: confidence interval; Pr: probability.

^a^See footnotes a-d of Table S3.3. for model details and interpretation of results.

^b^Logistic regressions were run for models including focal participants' smoking susceptibility as the outcome variable, with robust (Huber White) standard errors. The predictor variable is the percentage of the relevant group (*-i*) classified as susceptible to commencing smoking at baseline (*t*=base) or follow-up (*t*=fu). The moderator, interaction of the moderator with the predictor variable, and baseline covariates were also included as independent variables in all models. The predictor variable, and continuous moderator variables were mean-centered

^c^Marginal effects calculated using the Delta-method. Results are the average change in the predicted probability of being classified as susceptible to commencing smoking for a 10% increase in the number of nominated friends/pupils in the same school class/pupils in the same school year group classified as being susceptible to commencing smoking among participants who are one standard deviation below the mean on the moderator variable ('low' values of continuous moderator variables).

^d^Marginal effects calculated using the Delta-method. Results are the average change in the predicted probability of being classified as susceptible to commencing smoking for a 10% increase in the number of nominated friends/pupils in the same school class/pupils in the same school year group classified as being susceptible to commencing smoking among participants who are one standard deviation above the mean on the moderator variable ('high' values of continuous moderator variables).

^e^Boundaries indicating regions of significance (values of the continuous moderator variable [non-centered] for which the conditional effect of the predictor variable on the average predicted probability of being classified as susceptible to commencing smoking differs significantly from 0) at the 95% and 99% confidence levels, calculated using the Johnson-Neyman technique.

**Table S3.5.** Marginal effects, regions of significance, and changes in R-squared values for models including social network parameters as moderator variables.

| **Model details^a^** | | | **Moderator: Individuals' follow-up clustering coefficients (0=least interconnections between nominated friends to 10=most interconnections between nominated friends).** | | | | | | | | | | |
| --- | --- | --- | --- | --- | --- | --- | --- | --- | --- | --- | --- | --- | --- |
| **Dependent variable** | **Peer group (*-i*)** | **Time-point (*t*)** | **Marginal effects (low; follow-up clustering coefficient=1.129)** | | | | **Marginal effects (high; follow-up clustering coefficient=5.662)** | | | | **Regions of significance** | | **∆R^2^** |
|  |  |  | ***b* (SE)** | **p-value** | **95% CI** | **99% CI** | ***b* (SE)** | **p-value** | **95% CI** | **99% CI** | **95%** | **99%** |  |
| P2S3 (-1 to 1) | Friends | fu | -0.07 (0.10) | 0.46 | -0.27, 0.12 | -0.33, 0.18 | 0.26 (0.09) | 0.003 | 0.09, 0.43 | 0.03, 0.49 | >3.892 | >4.704 | 0.0065 |
| Donation to ASSIST/Dead Cool (0 to 10) | Friends | fu | 0.18 (0.09) | 0.04 | 0.007, 0.36 | -0.05, 0.41 | 0.44 (0.06) | <0.001 | 0.32, 0.55 | 0.28, 0.59 | >0.807 | >1.568 | 0.0054 |
| IN3 (-2 to 2) | Class | fu | -0.27 (0.15) | 0.08 | -0.56, 0.03 | -0.66, 0.12 | 0.21 (0.13) | 0.09 | -0.03, 0.46 | -0.11, 0.54 | <0.080  >6.479 | None | 0.0028 |
| DN1.1 (1 to 5) | Year | fu | 1.09 (0.31) | <0.001 | 0.50, 1.69 | 0.31, 1.88 | 0.15 (0.19) | 0.43 | -0.22, 0.51 | -0.33, 0.62 | <4.852 | <4.335 | 0.0080 |
| **Dependent variable** | **Peer group (*-i*)** | **Time-point (*t*)** | **Moderator: Individuals' baseline eigenvector centralities (0.008=least well-connected friends, i.e. least central to 3.13=most well-connected friends, i.e. most central).** | | | | | | | | | | |
|  |  |  | **Marginal effects (low; baseline EVC=0.141)** | | | | **Marginal effects (high; baseline EVC=1.305)** | | | | **Regions of significance** | | **∆R^2^** |
|  |  |  | ***b* (SE)** | **p-value** | **95% CI** | **99% CI** | ***b* (SE)** | **p-value** | **95% CI** | **99% CI** | **95%** | **99%** |  |
| P2S3 (-1 to 1) | Year | base | -0.68 (0.36) | 0.06 | -1.38, 0.02 | -1.61, 0.24 | 0.68 (0.36) | 0.06 | -0.02, 1.37 | -0.24, 1.59 | <0.111  >1.337 | >1.912 | 0.0067 |
| P2S5 (-1 to 1) | Class | base | 0.03 (0.13) | 0.79 | -0.22, 0.28 | -0.29, 0.36 | 0.63 (0.13) | <0.001 | 0.38, 0.89 | 0.29, 0.97 | >0.464 | >0.565 | 0.0084 |
|  | Year | base | 0.26 (0.18) | 0.14 | -0.09, 0.60 | -0.20, 0.71 | 0.84 (0.20) | <0.001 | 0.44, 1.23 | 0.32, 1.35 | >0.267 | >0.416 | 0.0044 |
| P3Q2 (-1 to 1) | Friends | base | 0.07 (0.07) | 0.34 | -0.07, 0.22 | -0.12, 0.26 | 0.29 (0.06) | <0.001 | 0.17, 0.42 | 0.13, 0.46 | >0.401 | >0.536 | 0.0036 |
|  | Year | base | 0.08 (0.14) | 0.60 | -0.20, 0.36 | -0.29, 0.44 | 0.58 (0.14) | <0.001 | 0.29, 0.86 | 0.21, 0.95 | >0.480 | >0.609 | 0.0047 |
| Experiment Part 3: Descriptive norms (Average P3Q1 to P3Q2; -1 to 1) | Friends | base | 0.03 (0.08) | 0.74 | -0.12, 0.17 | -0.17, 0.22 | 0.26 (0.07) | <0.001 | 0.12, 0.39 | 0.08, 0.44 | >0.581 | >0.731 | 0.0040 |
| IN3 (-2 to 2) | Year | base | -0.25 (0.24) | 0.30 | -0.71, 0.22 | -0.86, 0.36 | 0.70 (0.23) | 0.002 | 0.25, 1.14 | 0.11, 1.28 | >0.909 | >1.096 | 0.0073 |
| Perceived physical risks (0 to 100%) | Friends | base | -0.03 (0.07) | 0.61 | -0.16, 0.10 | -0.21, 0.14 | 0.20 (0.07) | 0.008 | 0.05, 0.34 | 0.007, 0.39 | >0.847 | >1.190 | 0.0037 |
| Perceived social risks (0 to 100%) | Friends^b^ | base | -0.01 (0.07) | 0.87 | -0.14, 0.12 | -0.18, 0.16 | 0.38 (0.08) | <0.001 | 0.23, 0.54 | 0.18, 0.59 | >0.476 | >0.563 | 0.0096 |
| Objectively measured smoking behavior (0 to 30 ppm) | Friends | base | 0.25 (0.07) | <0.001 | 0.11, 0.39 | 0.07, 0.43 | 0.48 (0.06) | <0.001 | 0.37, 0.60 | 0.33, 0.64 | All | All | 0.0071 |
|  | Class | base | 0.25 (0.07) | <0.001 | 0.12, 0.38 | 0.08, 0.42 | 0.51 (0.07) | <0.001 | 0.39, 0.64 | 0.35, 0.68 | All | All | 0.0078 |
| **Dependent variable** | **Peer group (*-i*)** | **Time-point (*t*)** | **Moderator: Individuals' baseline closeness centralities (2.24=longest distance to all other nodes, i.e. least central to 5.10=shortest distance to all other nodes, i.e. most central); Individuals' follow-up closeness centralities (2.15 to 4.94).** | | | | | | | | | | |
|  |  |  | **Marginal effects (low; baseline CC=3.208; follow-up CC=3.007)** | | | | **Marginal effects (high; baseline CC=4.097; follow-up CC=3.984)** | | | | **Regions of significance** | | **∆R^2^** |
|  |  |  | ***b* (SE)** | **p-value** | **95% CI** | **99% CI** | ***b* (SE)** | **p-value** | **95% CI** | **99% CI** | **95%** | **99%** |  |
| IN5 (-2 to 2) | Year | fu | 0.55 (0.27) | 0.04 | 0.02, 1.08 | -0.15, 1.25 | -0.86 (0.36) | 0.02 | -1.58, -0.15 | -1.80, 0.07 | <3.030  >3.761 | <2.645  >4.210 | 0.0056 |
| DN1.3 (1 to 5) | Year | fu | -0.21 (0.17) | 0.22 | -0.55, 0.13 | -0.65, 0.23 | 0.45 (0.21) | 0.03 | 0.04, 0.87 | -0.09, 1.00 | <2.562  >3.863 | None | 0.0032 |
| Self-report smoking behavior (1 to 4) | Class | base | -0.01 (0.13) | 0.94 | -0.26, 0.25 | -0.35, 0.33 | 0.50 (0.17) | 0.005 | 0.15, 0.84 | 0.05, 0.95 | >3.618 | >3.829 | 0.0035 |
|  | Year | base | 0.14 (0.21) | 0.51 | -0.28, 0.55 | -0.41, 0.68 | 1.04 (0.26) | <0.001 | 0.54, 1.54 | 0.38, 1.70 | >3.429 | >3.533 | 0.0043 |
| Intentions (1 to 6) | Year | fu | -0.17 (0.23) | 0.47 | -0.62, 0.28 | -0.77, 0.43 | 0.61 (0.21) | 0.003 | 0.21, 1.01 | 0.08, 1.14 | >3.588 | >3.744 | 0.0044 |
| Self-efficacy (Emotional; 1 to 6) | Friends | base | 0.02 (0.06) | 0.72 | -0.10, 0.15 | -0.14, 0.19 | 0.35 (0.14) | 0.009 | 0.09, 0.62 | 0.003, 0.70 | <2.528  >3.555 | >4.044 | 0.0056 |
|  | Class | base | 0.07 (0.13) | 0.61 | -0.19, 0.32 | -0.26, 0.39 | 0.74 (0.21) | <0.001 | 0.33, 1.15 | 0.20, 1.28 | <2.355  >3.421 | >3.527 | 0.0055 |
| Self-efficacy (Friends; 1 to 6) | Friends | base | -0.006 (0.06) | 0.92 | -0.12, 0.11 | -0.15, 0.14 | 0.27 (0.11) | 0.01 | 0.06, 0.48 | -0.007, 0.55 | <2.683  >3.682 | >4.203 | 0.0040 |
|  | Class | base | -0.07 (0.13) | 0.61 | -0.33, 0.20 | -0.41, 0.28 | 0.59 (0.20) | 0.004 | 0.19, 0.98 | 0.07, 1.11 | <2.557  >3.607 | >3.780 | 0.0054 |
| Perceived social risks (0 to 100%) | Friends | base | 0.04 (0.06) | 0.49 | -0.08, 0.16 | -0.11, 0.20 | 0.31 (0.07) | <0.001 | 0.16, 0.45 | 0.11, 0.50 | >3.412 | >3.506 | 0.0054 |
| Perceived addiction risks (0 to 100%) | Friends | base | 0.09 (0.07) | 0.20 | -0.05, 0.24 | -0.09, 0.28 | 0.38 (0.08) | <0.001 | 0.23, 0.53 | 0.18, 0.57 | >3.320 | >3.412 | 0.0059 |
| Objectively measured smoking behavior (0 to 30 ppm) | Friends^b^ | fu | 0.21 (0.10) | 0.03 | 0.02, 0.40 | -0.04, 0.46 | 0.67 (0.06) | <0.001 | 0.55, 0.78 | 0.51, 0.82 | >2.979 | >3.070 | 0.0313 |
|  | Class | base | 0.23 (0.07) | 0.001 | 0.10, 0.37 | 0.05, 0.41 | 0.51 (0.07) | <0.001 | 0.37, 0.65 | 0.32, 0.70 | >2.999 | >3.101 | 0.0072 |
| **Dependent variable** | **Peer group (*-i*)** | **Time-point (*t*)** | **Moderator: Individuals' follow-up betweenness centralities (0=node falls on the least proportion of shortest paths between two other nodes, i.e. least central to 5.49=node falls on the greatest proportion of shortest paths between two other nodes, i.e. most central).** | | | | | | | | | | |
|  |  |  | **Marginal effects (low; follow-up BC=0.000)** | | | | **Marginal effects (high; follow-up BC=0.767)** | | | | **Regions of significance** | | **∆R^2^** |
|  |  |  | ***b* (SE)** | **p-value** | **95% CI** | **99% CI** | ***b* (SE)** | **p-value** | **95% CI** | **99% CI** | **95%** | **99%** |  |
| Self-report descriptive norms 2 (Average DN2.1 to DN2.3; 1 to 5) | Friends | fu | 0.01 (0.07) | 0.83 | -0.12, 0.15 | -0.17, 0.20 | 0.27 (0.08) | 0.001 | 0.10, 0.43 | 0.05, 0.49 | >0.300 | >0.423 | 0.0046 |
| Objectively measured smoking behavior (0 to 30 ppm) | Friends^b^ | fu | 0.34 (0.09) | <0.001 | 0.16, 0.51 | 0.11, 0.56 | 0.76 (0.07) | <0.001 | 0.63, 0.90 | 0.58, 0.94 | All | All | 0.0260 |
|  | Year | fu | 0.72 (0.07) | <0.001 | 0.59, 0.86 | 0.54, 0.91 | 1.01 (0.09) | <0.001 | 0.84, 1.18 | 0.79, 1.23 | All | All | 0.0071 |
| **Dependent variable** | **Peer group (*-i*)** | **Time-point (*t*)** | **Moderator: Baseline school network Gini degree coefficients (1.70=least expected difference in degree between two randomly selected nodes, i.e. degree distribution is least heterogeneous to 2.67=greatest expected difference in degree between two randomly selected nodes, i.e. degree distribution is most heterogeneous); Follow-up school network Gini degree coefficients (1.91 to 2.91).** | | | | | | | | | | |
|  |  |  | **Marginal effects (low; baseline GDC=1.869; follow-up GDC=1.971)** | | | | **Marginal effects (high; baseline GDC=2.414; follow-up GDC=2.523)** | | | | **Regions of significance** | | **∆R^2^** |
|  |  |  | ***b* (SE)** | **p-value** | **95% CI** | **99% CI** | ***b* (SE)** | **p-value** | **95% CI** | **99% CI** | **95%** | **99%** |  |
| P2S4 (-1 to 1) | Year | base | 1.51 (0.53) | 0.004 | 0.47, 2.55 | 0.15, 2.88 | -0.69 (0.55) | 0.21 | -1.76, 0.38 | -2.10, 0.72 | <2.073 | <1.980 | 0.0066 |
|  | Year | fu | 0.88 (0.23) | <0.001 | 0.44, 1.33 | 0.30, 1.47 | -1.17 (0.54) | 0.03 | -2.22, -0.11 | -2.55, 0.22 | <2.102  >2.461 | <2.071  >2.746 | 0.0080 |
| P2S6 (-1 to 1) | Year | base | 1.16 (0.29) | <0.001 | 0.60, 1.72 | 0.42, 1.90 | 0.02 (0.24) | 0.94 | -0.45, 0.49 | -0.60, 0.64 | <2.245 | <2.192 | 0.0079 |
| Donation to ASSIST/Dead Cool (0 to 10) | Friends | base | -0.06 (0.08) | 0.44 | -0.23, 0.10 | -0.28, 0.15 | 0.37 (0.08) | <0.001 | 0.21, 0.52 | 0.16, 0.57 | >2.094 | >2.134 | 0.0120 |
|  | Year | base | -0.42 (0.30) | 0.17 | -1.01, 0.18 | -1.20, 0.36 | 0.91 (0.27) | 0.001 | 0.38, 1.44 | 0.22, 1.60 | <1.730  >2.205 | >2.267 | 0.0096 |
| Self-report injunctive norms (Average IN1 to IN7; -2 to 2) | Friends | base | 0.21 (0.07) | 0.004 | 0.07, 0.36 | 0.02, 0.40 | -0.08 (0.09) | 0.40 | -0.25, 0.10 | -0.31, 0.16 | <2.050 | <1.952 | 0.0032 |
| DN1.1 (1 to 5) | Year | base | 2.32 (0.64) | <0.001 | 1.06, 3.59 | 0.66, 3.99 | 0.69 (0.33) | 0.04 | 0.04, 1.33 | -0.16, 1.53 | <2.427 | <2.370 | 0.0051 |
| DN2.1 (1 to 5) | Year | fu | 0.58 (0.23) | 0.01 | 0.12, 1.04 | -0.02, 1.19 | -0.53 (0.31) | 0.09 | -1.13, 0.07 | -1.32, 0.26 | <2.075  >2.624 | <1.933 | 0.0038 |
| Perceived behavioral control (to avoid smoking; 1 to 5) | Friends | fu | 0.25 (0.09) | 0.006 | 0.07, 0.43 | 0.02, 0.49 | -0.18 (0.10) | 0.08 | -0.37, 0.02 | -0.44, 0.08 | <2.110  >2.573 | <2.012  >2.873 | 0.0103 |
|  | Class | fu | 0.32 (0.17) | 0.06 | -0.02, 0.66 | -0.13, 0.77 | -0.38 (0.21) | 0.08 | -0.80, 0.04 | -0.93, 0.18 | <1.940  >2.617 | None | 0.0063 |
| Objectively measured smoking behavior (0 to 30 ppm) | Friends | fu | 0.64 (0.07) | <0.001 | 0.50, 0.77 | 0.45, 0.82 | 0.42 (0.07) | <0.001 | 0.28, 0.57 | 0.24, 0.61 | All | <2.879 | 0.0145 |

*b*: unstandardized regression coefficient; SE: standard error; CI: confidence interval; ppm: parts per million; EVC: eigenvector centrality; CC: closeness centrality; BC: betweenness centrality; GDC: Gini degree coefficient.

^a^See footnotes a-d of Table S3.3. for model details and interpretation of results. For models examining social network betweenness centralities as moderators, 'low' values were capped at the minimum value.

^b^Retained statistical significance at the 5% level after using the Holm-Bonferroni procedure to correct the p-values for multiple testing (p≤0.05; based on 276 tests of interaction effects carried out per moderator).

**Table S3.6.** Marginal effects, regions of significance, and changes in R-squared values for models including the self-efficacy subscales as moderator variables.

| **Model details^a^** | | | **Moderator: Self-efficacy emotional subscale (1=least self-efficacy to 6=most self-efficacy).** | | | | | | | | | | |
| --- | --- | --- | --- | --- | --- | --- | --- | --- | --- | --- | --- | --- | --- |
| **Dependent variable** | **Peer group (*-i*)** | **Time-point (*t*)** | **Marginal effects (low; Self-efficacy=4.804)** | | | | **Marginal effects (high; Self-efficacy=6.000)** | | | | **Regions of significance** | | **∆R^2^** |
|  |  |  | ***b* (SE)** | **p-value** | **95% CI** | **99% CI** | ***b* (SE)** | **p-value** | **95% CI** | **99% CI** | **95%** | **99%** |  |
| Intentions (1 to 6) | Year | base | 1.63 (0.39) | <0.001 | 0.86, 2.40 | 0.62, 2.65 | 0.58 (0.25) | 0.02 | 0.09, 1.08 | -0.07, 1.24 | All | <5.938 | 0.0063 |
|  | Year | fu | 0.82 (0.24) | 0.001 | 0.36, 1.28 | 0.21, 1.43 | 0.17 (0.15) | 0.24 | -0.12, 0.47 | -0.21, 0.56 | <5.819 | <5.659 | 0.0068 |
| Self-report injunctive norms (Average IN1 to IN7; -2 to 2) | Friends | fu | 0.31 (0.08) | <0.001 | 0.16, 0.47 | 0.11, 0.52 | 0.09 (0.06) | 0.13 | -0.03, 0.21 | -0.07, 0.25 | <5.886 | <5.734 | 0.0052 |
| Objectively measured smoking behavior (0 to 30 ppm) | Class | base | 0.25 (0.07) | <0.001 | 0.12, 0.39 | 0.08, 0.43 | 0.43 (0.05) | <0.001 | 0.32, 0.53 | 0.29, 0.57 | >4.182 | >4.416 | 0.0055 |
|  | Year | base | 0.47 (0.08) | <0.001 | 0.31, 0.64 | 0.26, 0.69 | 0.63 (0.06) | <0.001 | 0.52, 0.74 | 0.48, 0.78 | >3.388 | >3.741 | 0.0036 |
| **Dependent variable** | **Peer group (*-i*)** | **Time-point (*t*)** | **Moderator: Self-efficacy friends subscale (1=least self-efficacy to 6=most self-efficacy).** | | | | | | | | | | |
|  |  |  | **Marginal effects (low; Self-efficacy=4.859)** | | | | **Marginal effects (high; Self-efficacy=6.000)** | | | | **Regions of significance** | | **∆R^2^** |
|  |  |  | ***b* (SE)** | **p-value** | **95% CI** | **99% CI** | ***b* (SE)** | **p-value** | **95% CI** | **99% CI** | **95%** | **99%** |  |
| Self-report smoking behavior (1 to 4) | Year | base | 1.22 (0.34) | <0.001 | 0.55, 1.88 | 0.35, 2.09 | 0.24 (0.15) | 0.11 | -0.06, 0.53 | -0.15, 0.62 | <5.943 | <5.838 | 0.0090 |
| Intentions (1 to 6) | Year | base | 1.73 (0.40) | <0.001 | 0.95, 2.51 | 0.70, 2.76 | 0.61 (0.26) | 0.02 | 0.11, 1.12 | -0.05, 1.28 | All | <5.959 | 0.0068 |
| Perceived physical risks (0 to 100%) | Friends | fu | 0.03 (0.06) | 0.62 | -0.09, 0.15 | -0.13, 0.19 | 0.18 (0.06) | 0.003 | 0.06, 0.30 | 0.02, 0.34 | <1.854  >5.416 | >5.702 | 0.0040 |
| Objectively measured smoking behavior (0 to 30 ppm) | Friends | base | 0.28 (0.07) | <0.001 | 0.15, 0.40 | 0.11, 0.45 | 0.41 (0.05) | <0.001 | 0.30, 0.51 | 0.27, 0.54 | >3.982 | >4.268 | 0.0039 |
|  | Class | base | 0.24 (0.06) | <0.001 | 0.12, 0.37 | 0.08, 0.41 | 0.44 (0.05) | <0.001 | 0.33, 0.54 | 0.30, 0.58 | <1.413  >4.307 | >4.506 | 0.0080 |
|  | Year | base | 0.44 (0.08) | <0.001 | 0.29, 0.60 | 0.24, 0.65 | 0.64 (0.06) | <0.001 | 0.53, 0.76 | 0.49, 0.79 | >3.736 | >4.004 | 0.0060 |
| **Dependent variable** | **Peer group (*-i*)** | **Time-point (*t*)** | **Moderator: Self-efficacy opportunity subscale (1=least self-efficacy to 6=most self-efficacy).** | | | | | | | | | | |
|  |  |  | **Marginal effects (low; Self-efficacy=5.196)** | | | | **Marginal effects (high; Self-efficacy=6.000)** | | | | **Regions of significance** | | **∆R^2^** |
|  |  |  | ***b* (SE)** | **p-value** | **95% CI** | **99% CI** | ***b* (SE)** | **p-value** | **95% CI** | **99% CI** | **95%** | **99%** |  |
| P2S3 (-1 to 1) | Year | fu | -0.73 (0.41) | 0.08 | -1.54, 0.08 | -1.79, 0.33 | 0.34 (0.26) | 0.18 | -0.16, 0.85 | -0.32, 1.01 | <5.066 | <3.908 | 0.0063 |
| Self-report injunctive norms (Average IN1 to IN7; -2 to 2) | Friends | fu | 0.34 (0.08) | <0.001 | 0.18, 0.50 | 0.13, 0.55 | 0.12 (0.06) | 0.03 | 0.01, 0.23 | -0.02, 0.27 | All | <5.928 | 0.0062 |
| DN2.3 (1 to 5) | Friends | base | 0.12 (0.09) | 0.18 | -0.05, 0.29 | -0.11, 0.35 | 0.32 (0.08) | <0.001 | 0.17, 0.48 | 0.12, 0.53 | <1.995  >5.358 | >5.510 | 0.0055 |
| Intentions (1 to 6) | Year | base | 1.70 (0.39) | <0.001 | 0.93, 2.47 | 0.69, 2.72 | 0.70 (0.25) | 0.005 | 0.21, 1.19 | 0.05, 1.34 | All | All | 0.0061 |
| Perceived physical risks (0 to 100%) | Friends | fu | 0.02 (0.06) | 0.72 | -0.10, 0.15 | -0.14, 0.18 | 0.17 (0.06) | 0.005 | 0.05, 0.29 | 0.01, 0.32 | <2.629  >5.656 | >5.881 | 0.0042 |
| Objectively measured smoking behavior (0 to 30 ppm) | Friends^b^ | base | 0.24 (0.06) | <0.001 | 0.12, 0.36 | 0.08, 0.40 | 0.41 (0.05) | <0.001 | 0.30, 0.52 | 0.27, 0.55 | <2.429  >4.762 | <1.143  >4.914 | 0.0070 |
|  | Class^b^ | base | 0.24 (0.07) | <0.001 | 0.11, 0.36 | 0.07, 0.40 | 0.42 (0.05) | <0.001 | 0.32, 0.53 | 0.29, 0.56 | <2.641  >4.838 | <1.505  >4.985 | 0.0079 |
|  | Year^b^ | base | 0.43 (0.08) | <0.001 | 0.29, 0.58 | 0.24, 0.63 | 0.63 (0.06) | <0.001 | 0.52, 0.75 | 0.48, 0.78 | >4.370 | >4.555 | 0.0070 |

*b*: unstandardized regression coefficient; SE: standard error; CI: confidence interval; ppm: parts per million.

^a^See footnotes a-d of Table S3.3. for model details and interpretation of results. For models examining self-efficacy subscales as moderator variables, 'high' values were capped at the maximum value of the scale.

^b^Retained statistical significance at the 5% level after using the Holm-Bonferroni procedure to correct the p-values for multiple testing (p≤0.05; based on 258 tests of interaction effects carried out per moderator).
