# Supplementary material for: Moderators of peer influence effects for adolescents’ smoking and vaping norms and outcomes in high and middle-income settings
Source: Front Psychol. 2025 Nov 3;16:1655761. doi: 10.3389/fpsyg.2025.1655761 (PMC12620383; doi:10.3389/fpsyg.2025.1655761)
Supplement: SUPPLEMENTARY FILE 4 — Figures showing conditional effects of peer influence for smoking and vaping outcomes by varying levels of the moderators with bounds indicating regions of significance. [file Table_4.pdf]

Supplementary File 4

Moderators of peer influence effects for adolescents' smoking and vaping norms and outcomes in high and middle-income settings.

Jennifer M. Murray*, Sharon C. Sánchez-Franco, Olga L. Sarmiento, Erik O. Kimbrough, Christopher Tate, Shannon C. Montgomery, Rajnish Kumar, Laura Dunne, Abhijit Ramalingam, Erin L. Krupka, Felipe Montes, Huiyu Zhou, Laurence Moore, Linda Bauld, Blanca Llorente, Frank Kee, Ruth F. Hunter*

*** Correspondence:** Corresponding Authors: jmurray39@qub.ac.uk, ruth.hunter@qub.ac.uk

This file includes:

Supplementary Figures S4.1. to S4.195. (figures showing conditional effects of peer influence for smoking and vaping outcomes by varying levels of the moderators with bounds indicating regions of significance).

Supplementary file 4: Figures showing conditional effects of peer influence for smoking and vaping outcomes by varying levels of the moderators with bounds indicating regions of significance.

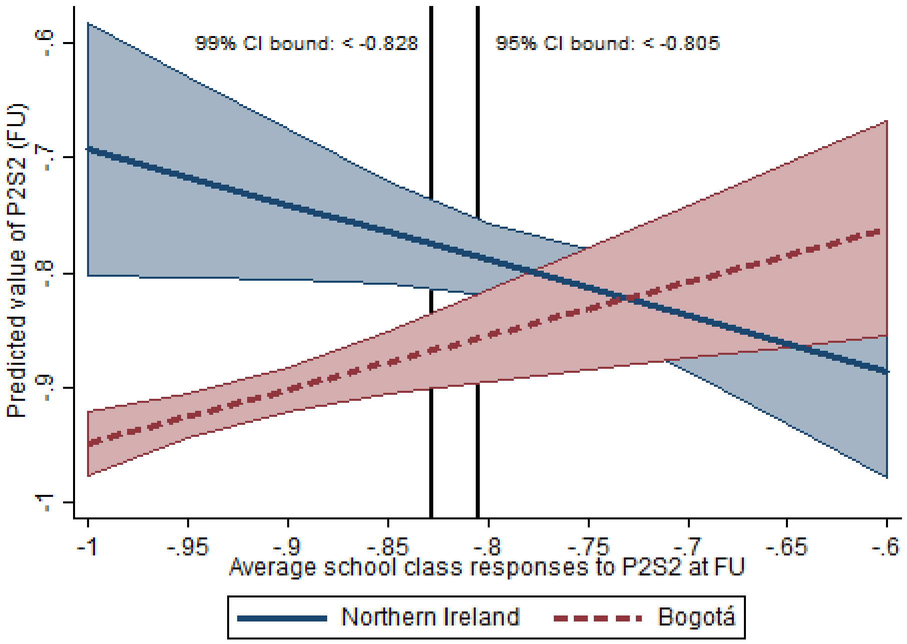

Figure S4.1. The conditional effects of peer influence from average school class responses for P2S2 at follow-up (predictor) on focal participants' values of P2S2 at follow-up (outcome) for participants in NI and Bogotá (moderator: setting) with 95% CI limits for each slope, and bounds indicating regions of significance at the 95% and 99% levels (indicating values of the predictor for which the slopes differ significantly for NI and Bogotá).

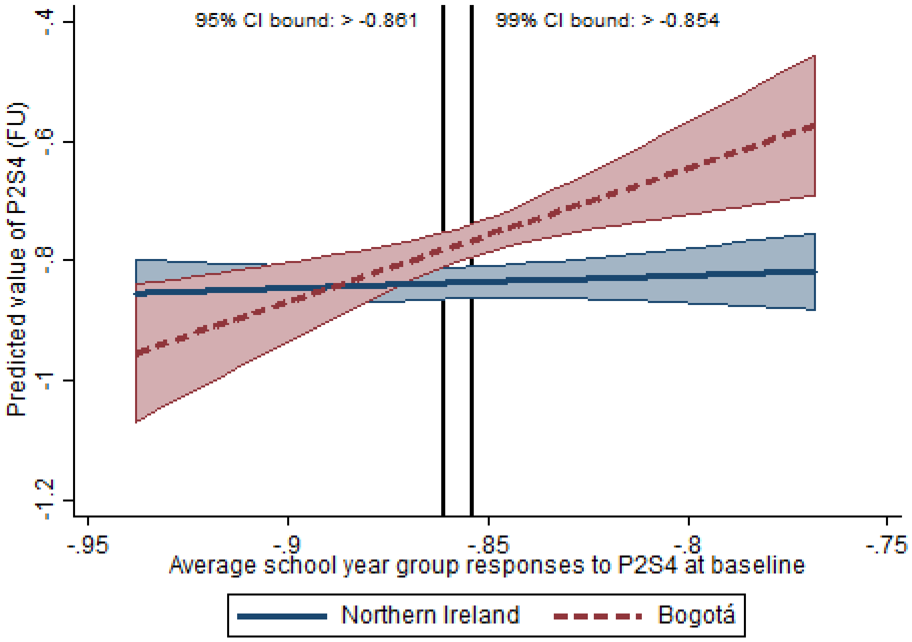

Figure S4.2. The conditional effects of peer influence from average school year group responses for P2S4 at baseline (predictor) on focal participants' values of P2S4 at follow-up (outcome) for participants in NI and Bogotá (moderator: setting) with 95% CI limits for each slope, and bounds indicating regions of significance at the 95% and 99% levels (indicating values of the predictor for which the slopes differ significantly for NI and Bogotá).

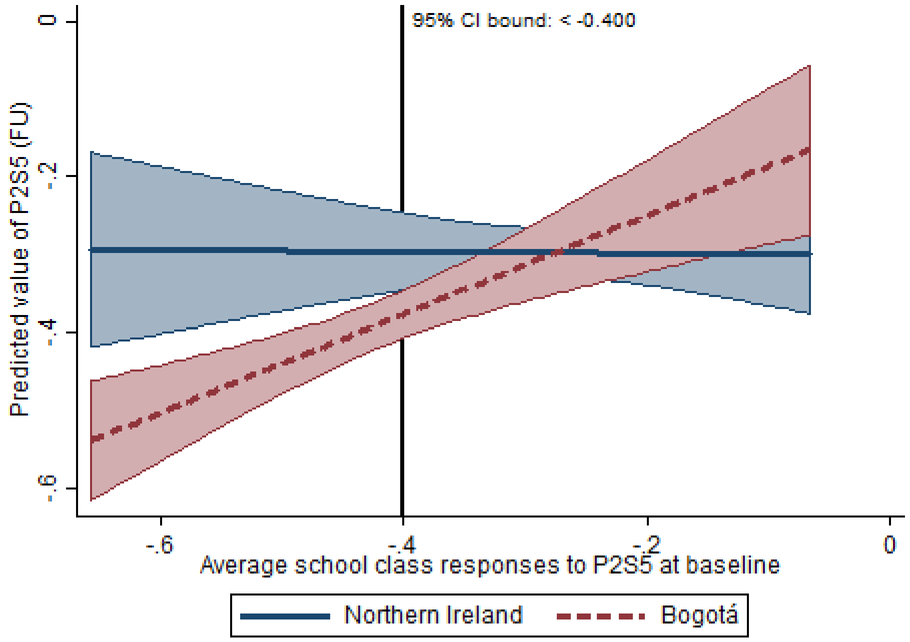

Figure S4.3. The conditional effects of peer influence from average school class responses for P2S5 at baseline (predictor) on focal participants' values of P2S5 at follow-up (outcome) for participants in NI and Bogotá (moderator: setting) with 95% CI limits for each slope, and bounds indicating regions of significance at the 95% level (indicating values of the predictor for which the slopes differ significantly for NI and Bogotá).

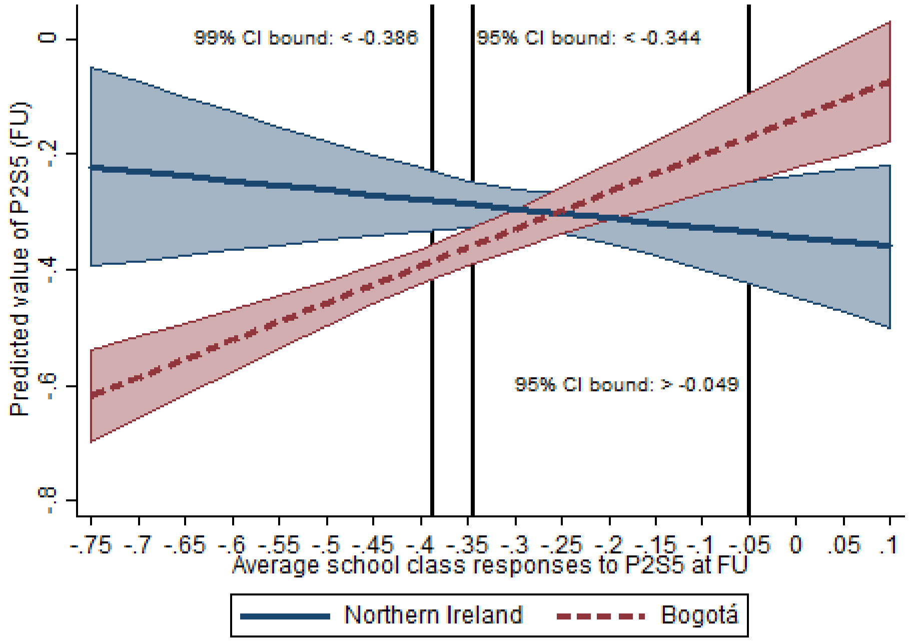

Figure S4.4. The conditional effects of peer influence from average school class responses for P2S5 at follow-up (predictor) on focal participants' values of P2S5 at follow-up (outcome) for participants in NI and Bogotá (moderator: setting) with 95% CI limits for each slope, and bounds indicating regions of significance at the 95% and 99% levels (indicating values of the predictor for which the slopes differ significantly for NI and Bogotá).

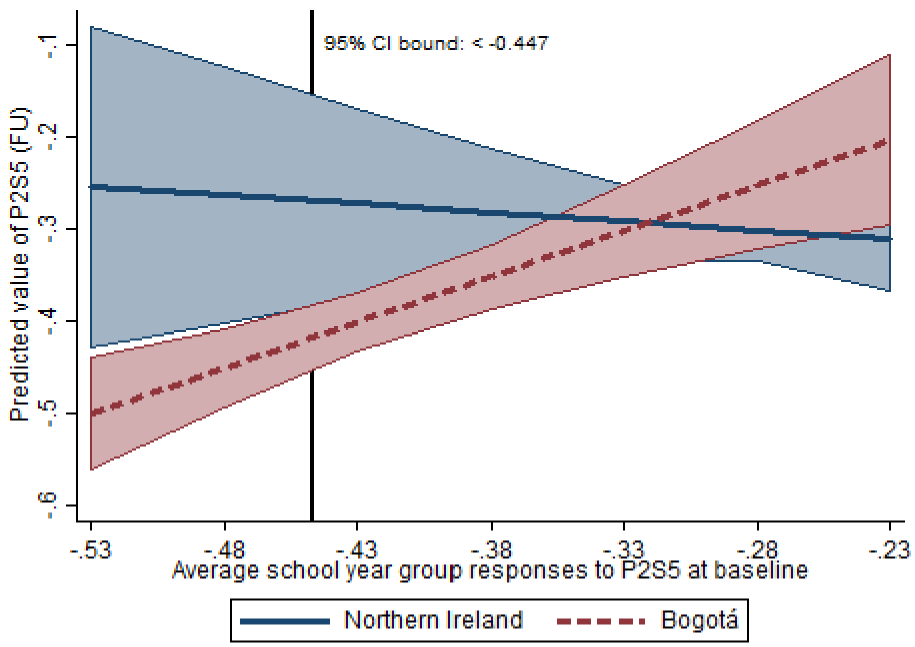

Figure S4.5. The conditional effects of peer influence from average school year group responses for P2S5 at baseline (predictor) on focal participants' values of P2S5 at follow-up (outcome) for participants in NI and Bogotá (moderator: setting) with 95% CI limits for each slope, and bounds indicating regions of significance at the 95% level (indicating values of the predictor for which the slopes differ significantly for NI and Bogotá).

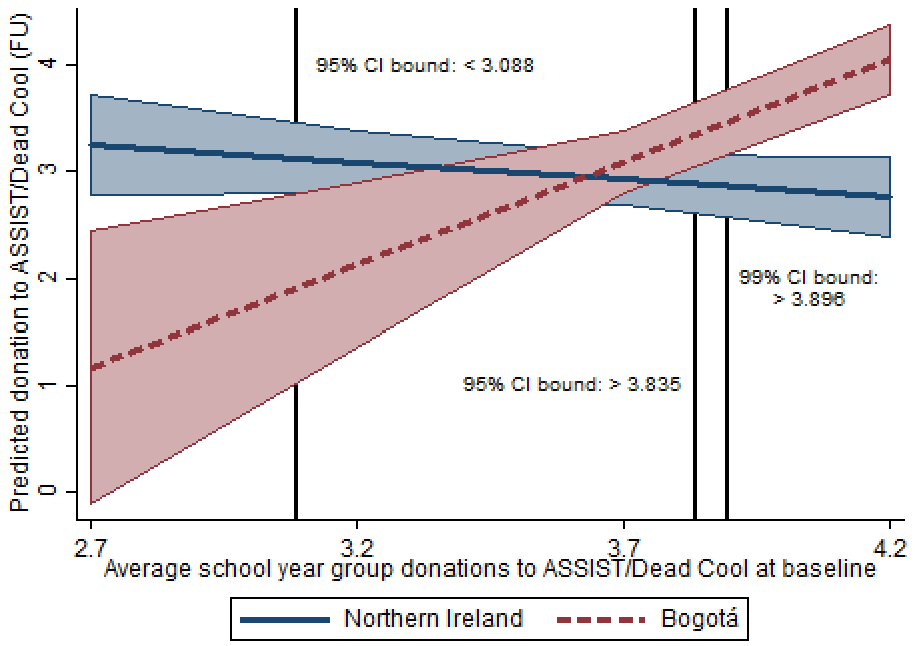

Figure S4.6. The conditional effects of peer influence from average school year group donations to ASSIST/Dead Cool at baseline (predictor) on focal participants' donations to ASSIST/Dead Cool at follow-up (outcome) for participants in NI and Bogotá (moderator: setting) with 95% CI limits for each slope, and bounds indicating regions of significance at the 95% and 99% levels (indicating values of the predictor for which the slopes differ significantly for NI and Bogotá).

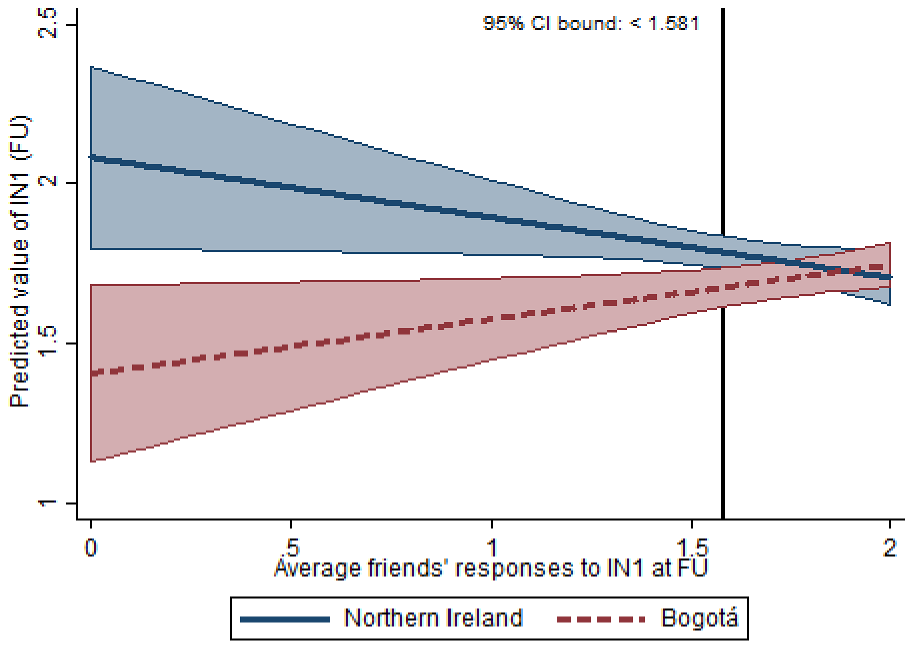

Figure S4.7. The conditional effects of peer influence from average friends' responses to IN1 at follow-up (predictor) on focal participants' values of IN1 at follow-up (outcome) for participants in NI and Bogotá (moderator: setting) with 95% CI limits for each slope, and bounds indicating regions of significance at the 95% level (indicating values of the predictor for which the slopes differ significantly for NI and Bogotá).

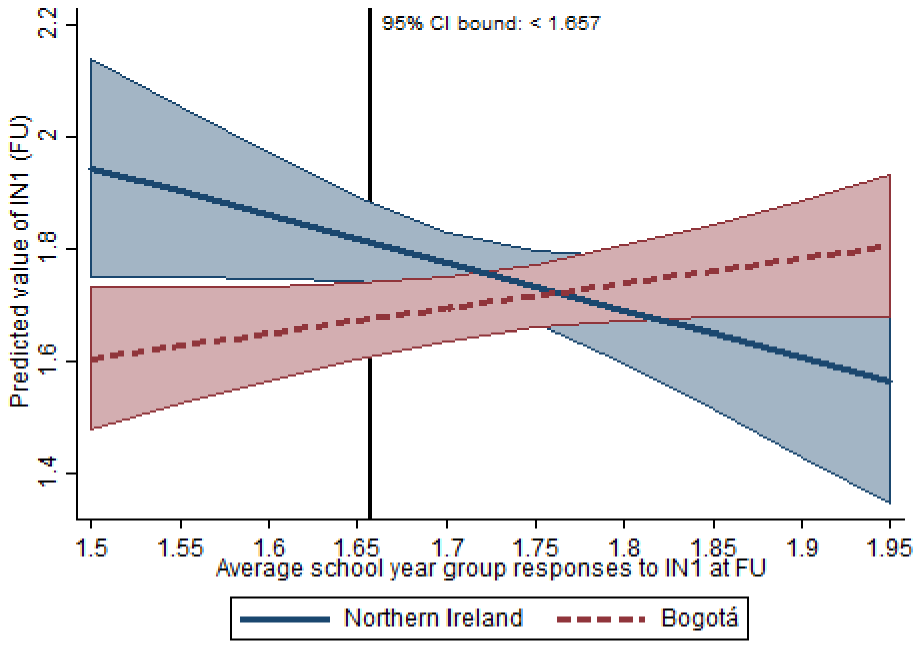

Figure S4.8. The conditional effects of peer influence from average school year group responses to IN1 at follow-up (predictor) on focal participants' values of IN1 at follow-up (outcome) for participants in NI and Bogotá (moderator: setting) with 95% CI limits for each slope, and bounds indicating regions of significance at the 95% level (indicating values of the predictor for which the slopes differ significantly for NI and Bogotá).

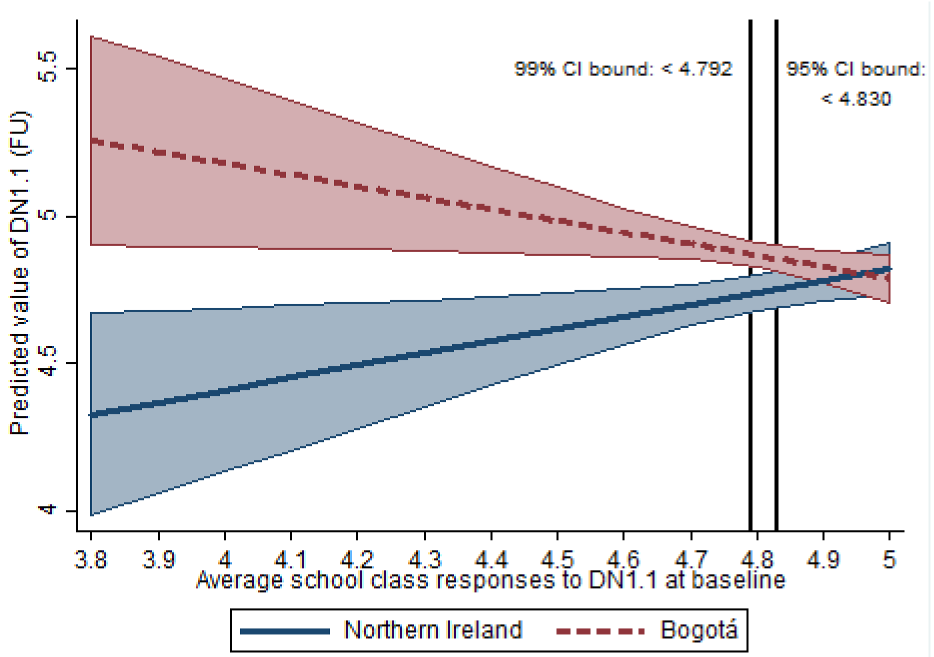

Figure S4.9. The conditional effects of peer influence from average school class responses to DN1.1 at baseline (predictor) on focal participants' values of DN1.1 at follow-up (outcome) for participants in NI and Bogotá (moderator: setting) with 95% CI limits for each slope, and bounds indicating regions of significance at the 95% and 99% levels (indicating values of the predictor for which the slopes differ significantly for NI and Bogotá).

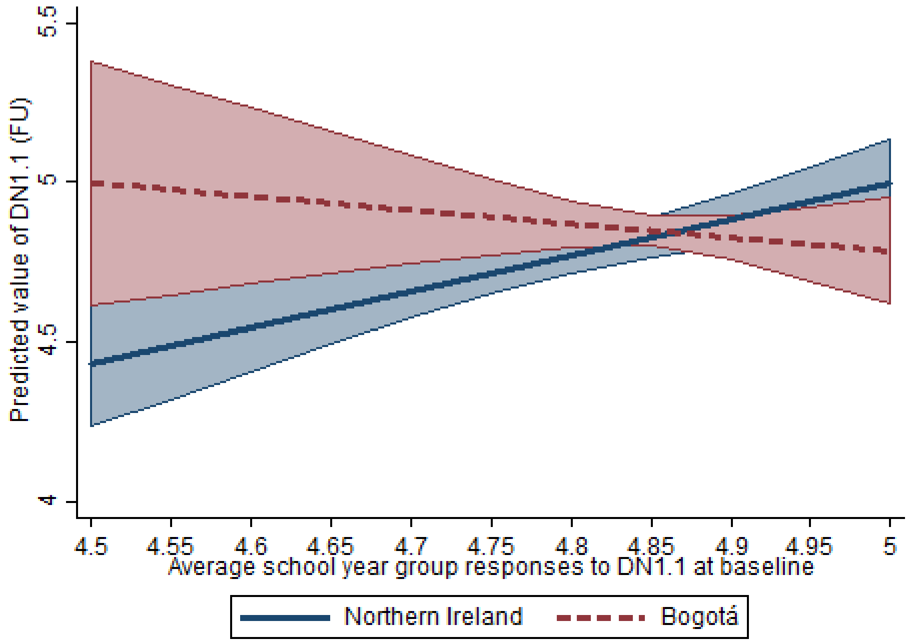

Figure S4.10. The conditional effects of peer influence from average school year group responses to DN1.1 at baseline (predictor) on focal participants' values of DN1.1 at follow-up (outcome) for participants in NI and Bogotá (moderator: setting) with 95% CI limits for each slope.

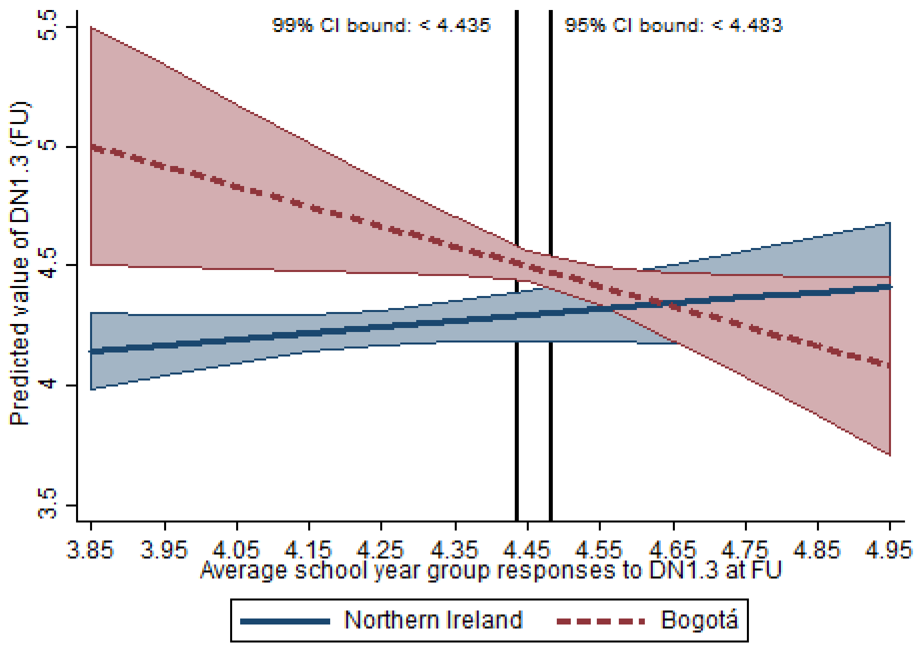

Figure S4.11. The conditional effects of peer influence from average school year group responses to DN1.3 at follow-up (predictor) on focal participants' values of DN1.3 at follow-up (outcome) for participants in NI and Bogotá (moderator: setting) with 95% CI limits for each slope, and bounds indicating regions of significance at the 95% and 99% levels (indicating values of the predictor for which the slopes differ significantly for NI and Bogotá).

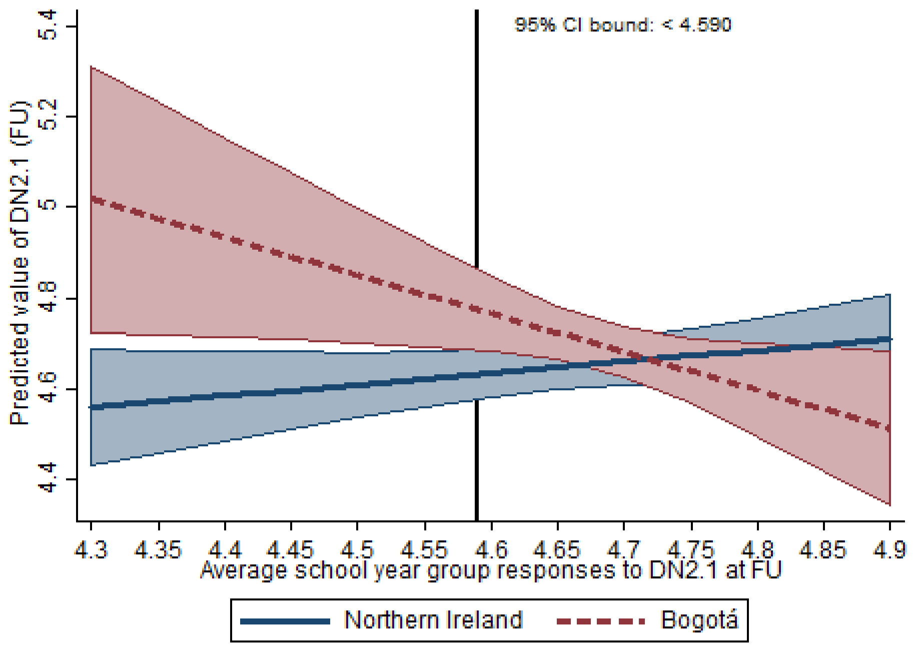

Figure S4.12. The conditional effects of peer influence from average school year group responses to DN2.1 at follow-up (predictor) on focal participants' values of DN2.1 at follow-up (outcome) for participants in NI and Bogotá (moderator: setting) with 95% CI limits for each slope, and bounds indicating regions of significance at the 95% level (indicating values of the predictor for which the slopes differ significantly for NI and Bogotá).

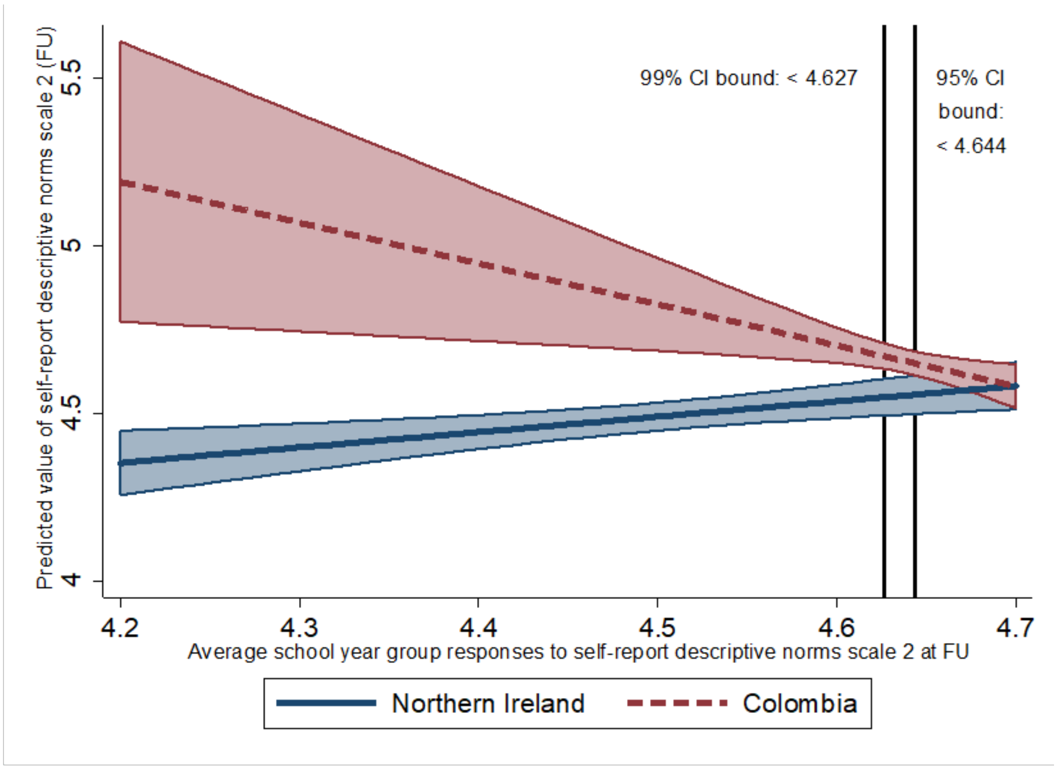

Figure S4.13. The conditional effects of peer influence from average school year group responses to self-report descriptive norms scale 2 (average DN2.1 to DN2.3) at follow-up (predictor) on focal participants' values of self-report descriptive norms scale 2 at follow-up (outcome) for participants in NI and Bogotá (moderator: setting) with 95% CI limits for each slope, and bounds indicating regions of significance at the 95% and 99% levels (indicating values of the predictor for which the slopes differ significantly for NI and Bogotá).

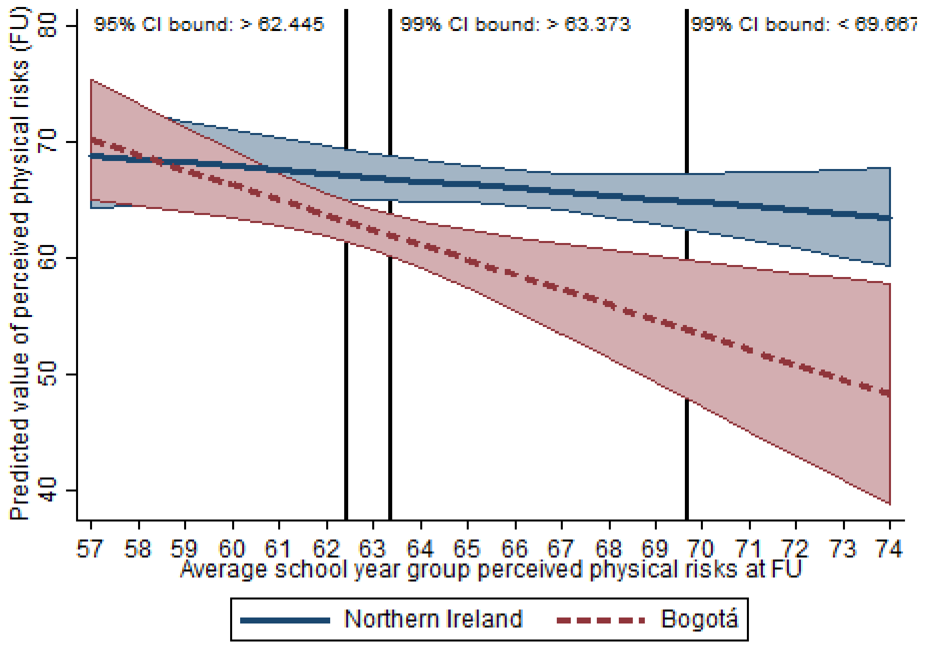

Figure S4.14. The conditional effects of peer influence from average school year group perceived physical risks at follow-up (predictor) on focal participants' perceived physical risks at follow-up (outcome) for participants in NI and Bogotá (moderator: setting) with 95% CI limits for each slope, and bounds indicating regions of significance at the 95% and 99% levels (indicating values of the predictor for which the slopes differ significantly for NI and Bogotá).

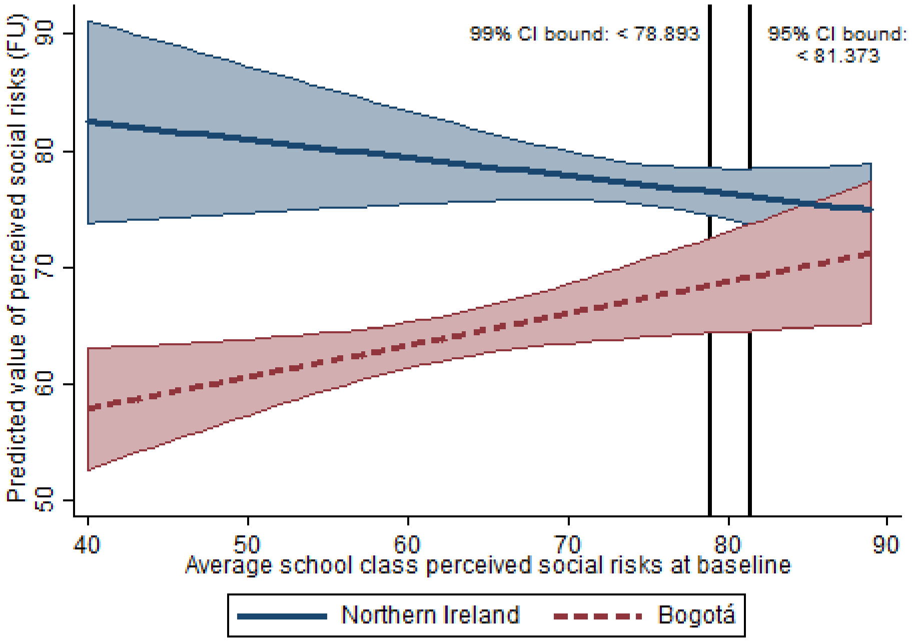

Figure S4.15. The conditional effects of peer influence from average school class perceived social risks at baseline (predictor) on focal participants' perceived social risks at follow-up (outcome) for participants in NI and Bogotá (moderator: setting) with 95% CI limits for each slope, and bounds indicating regions of significance at the 95% and 99% levels (indicating values of the predictor for which the slopes differ significantly for NI and Bogotá).

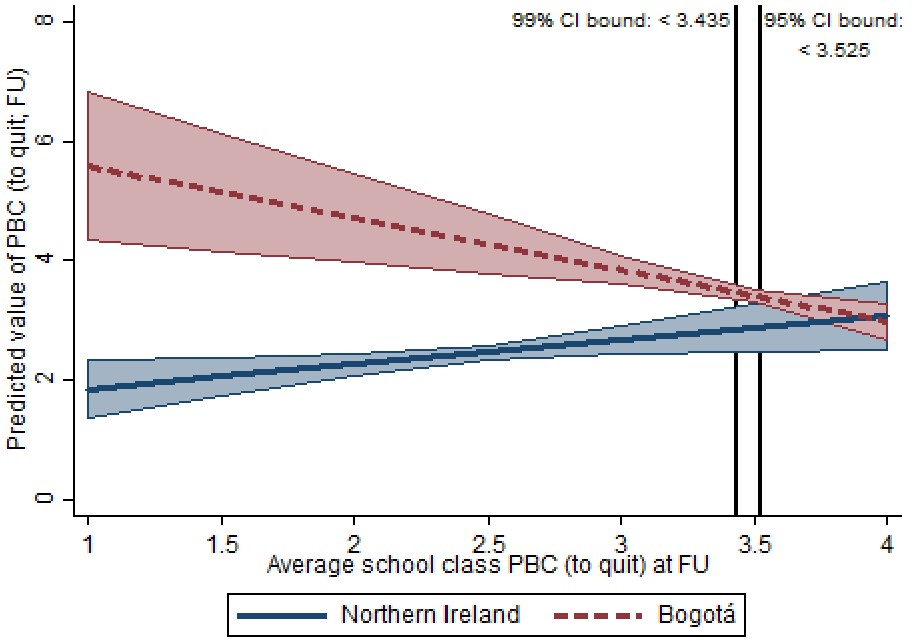

Figure S4.16. The conditional effects of peer influence from average school class perceived behavioral control (easy to quit) at follow-up (predictor) on focal participants' perceived behavioral control (easy to quit) at follow-up (outcome) for participants in NI and Bogotá (moderator: setting) with 95% CI limits for each slope, and bounds indicating regions of significance at the 95% and 99% levels (indicating values of the predictor for which the slopes differ significantly for NI and Bogotá).

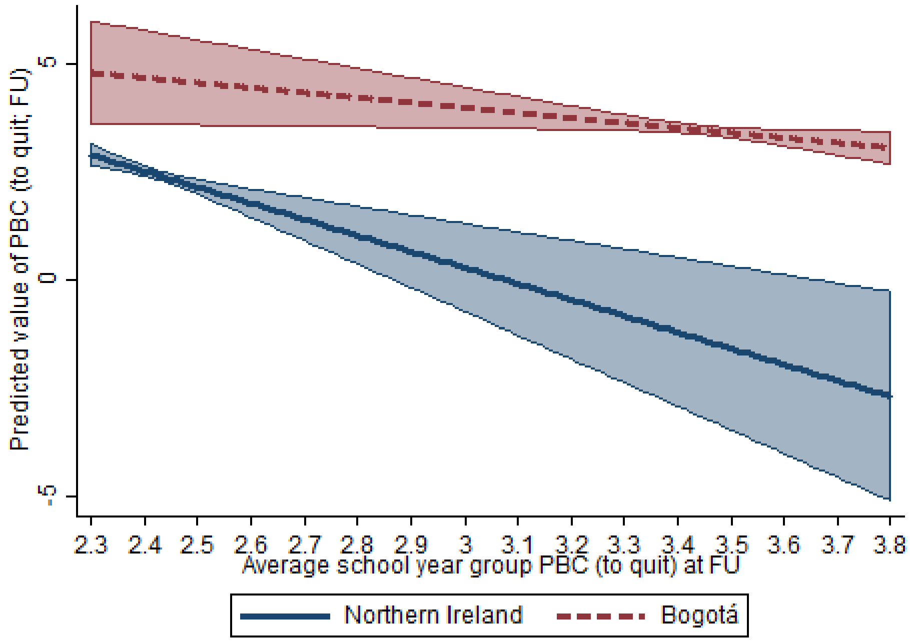

Figure S4.17. The conditional effects of peer influence from average school year group perceived behavioral control (easy to quit) at follow-up (predictor) on focal participants' perceived behavioral control (easy to quit) at follow-up (outcome) for participants in NI and Bogotá (moderator: setting) with 95% CI limits for each slope.

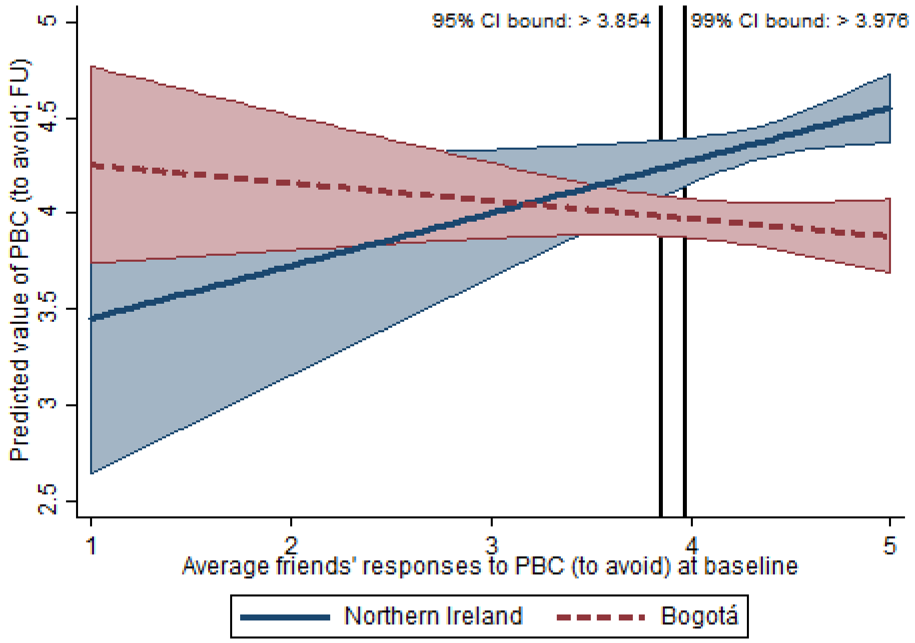

Figure S4.18. The conditional effects of peer influence from average friends' perceived behavioral control (easy to avoid) at baseline (predictor) on focal participants' perceived behavioral control (easy to avoid) at follow-up (outcome) for participants in NI and Bogotá (moderator: setting) with 95% CI limits for each slope, and bounds indicating regions of significance at the 95% and 99% levels (indicating values of the predictor for which the slopes differ significantly for NI and Bogotá).

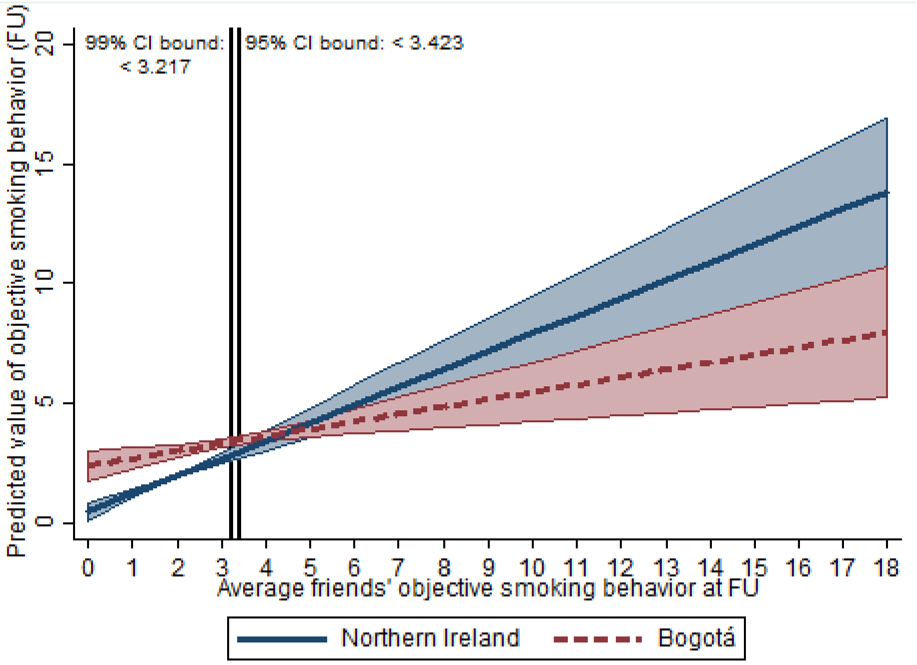

Figure S4.19. The conditional effects of peer influence from average friends' objectively measured smoking behavior at follow-up (predictor) on focal participants' objectively measured smoking behavior at follow-up (outcome) for participants in NI and Bogotá (moderator: setting) with 95% CI limits for each slope, and bounds indicating regions of significance at the 95% and 99% levels (indicating values of the predictor for which the slopes differ significantly for NI and Bogotá).

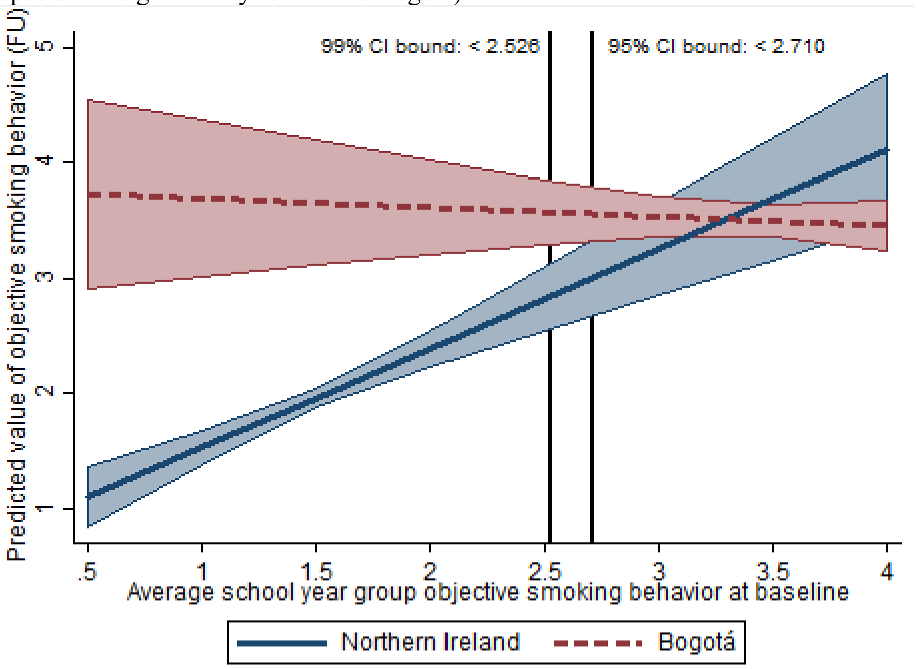

Figure S4.20. The conditional effects of peer influence from average school year group objectively measured smoking behavior at baseline (predictor) on focal participants' objectively measured smoking behavior at follow-up (outcome) for participants in NI and Bogotá (moderator: setting) with 95% CI limits for each slope, and bounds indicating regions of significance at the 95% and 99% levels (indicating values of the predictor for which the slopes differ significantly for NI and Bogotá).

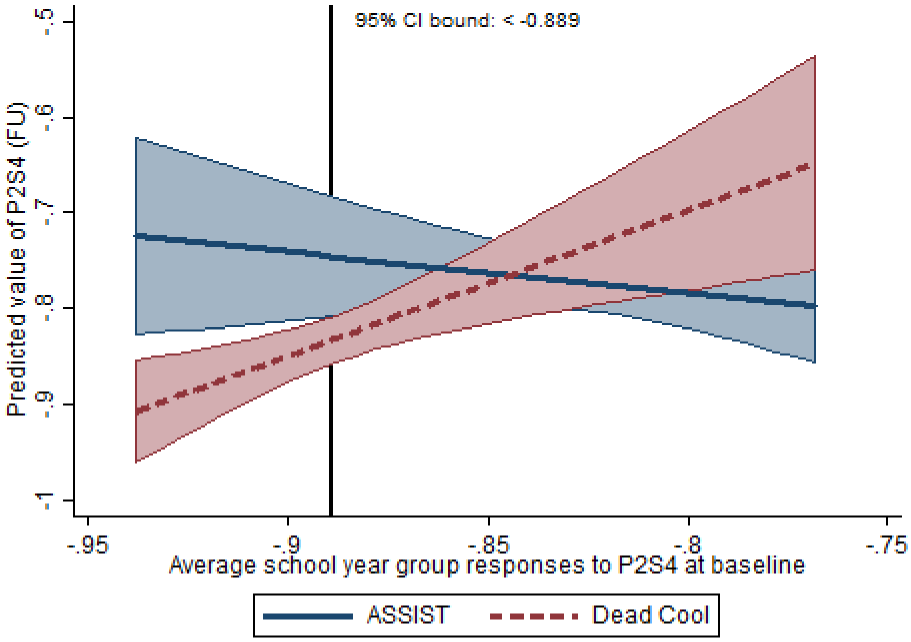

Figure S4.21. The conditional effects of peer influence from average school year group responses for P2S4 at baseline (predictor) on focal participants' values of P2S4 at follow-up (outcome) for participants in ASSIST and Dead Cool schools (moderator: intervention) with 95% CI limits for each slope, and bounds indicating regions of significance at the 95% level (indicating values of the predictor for which the slopes differ significantly for ASSIST and Dead Cool).

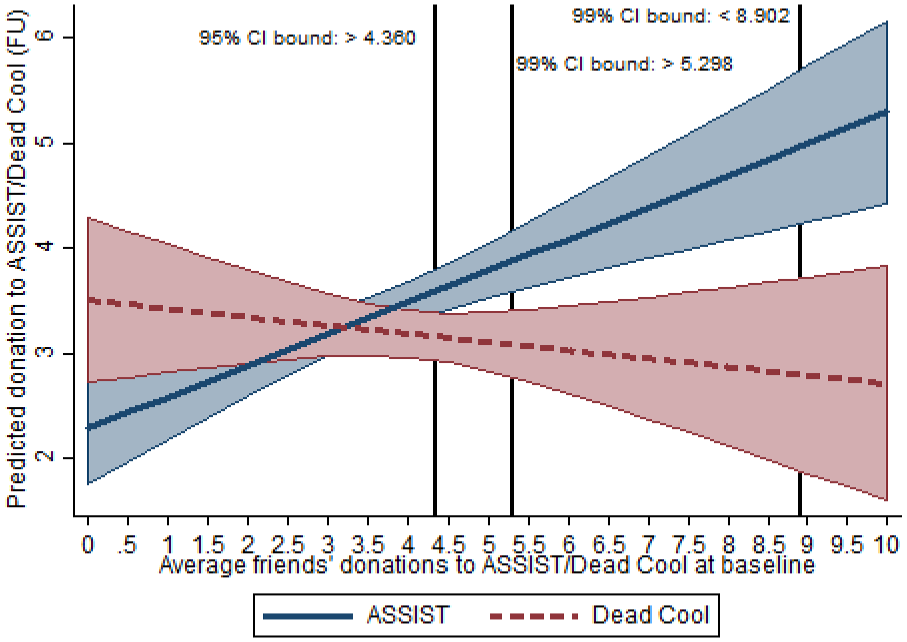

Figure S4.22. The conditional effects of peer influence from average friends' donations to ASSIST/Dead Cool at baseline (predictor) on focal participants' donations to ASSIST/Dead Cool at follow-up (outcome) for participants in ASSIST and Dead Cool schools (moderator: intervention) with 95% CI limits for each slope, and bounds indicating regions of significance at the 95% and 99% levels (indicating values of the predictor for which the slopes differ significantly for ASSIST and Dead Cool).

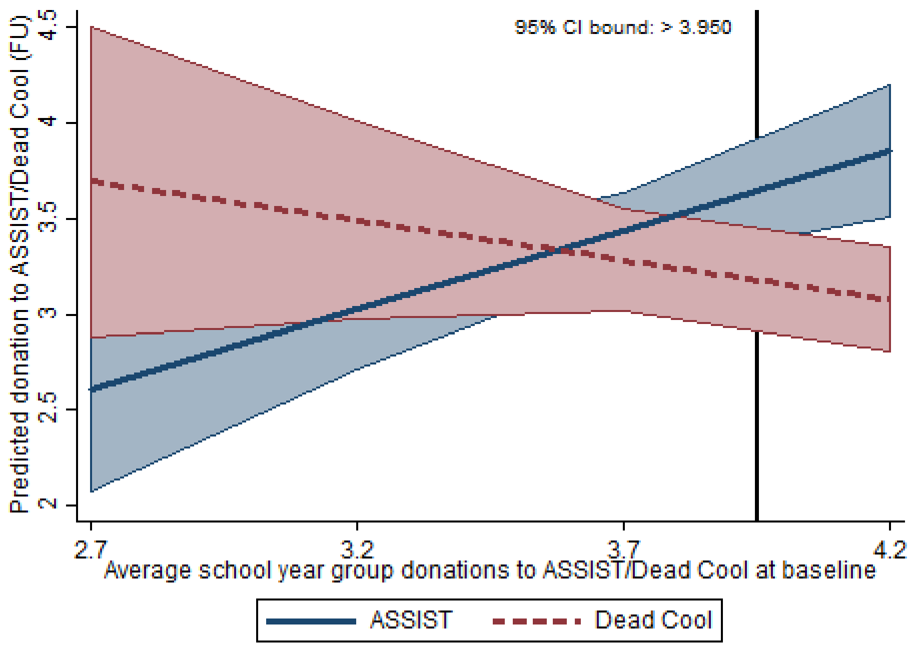

Figure S4.23. The conditional effects of peer influence from average school year group donations to ASSIST/Dead Cool at baseline (predictor) on focal participants' donations to ASSIST/Dead Cool at follow-up (outcome) for participants in ASSIST and Dead Cool schools (moderator: intervention) with 95% CI limits for each slope, and bounds indicating regions of significance at the 95% level (indicating values of the predictor for which the slopes differ significantly for ASSIST and Dead Cool).

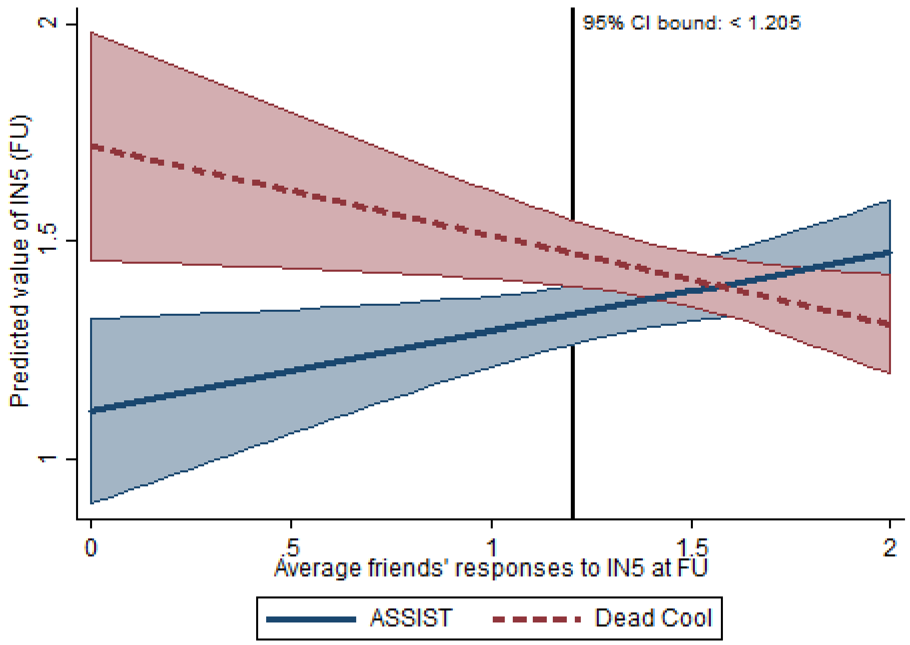

Figure S4.24. The conditional effects of peer influence from average friends' responses to IN5 at follow-up (predictor) on focal participants' values of IN5 at follow-up (outcome) for participants in ASSIST and Dead Cool schools (moderator: intervention) with 95% CI limits for each slope, and bounds indicating regions of significance at the 95% level (indicating values of the predictor for which the slopes differ significantly for ASSIT and Dead Cool).

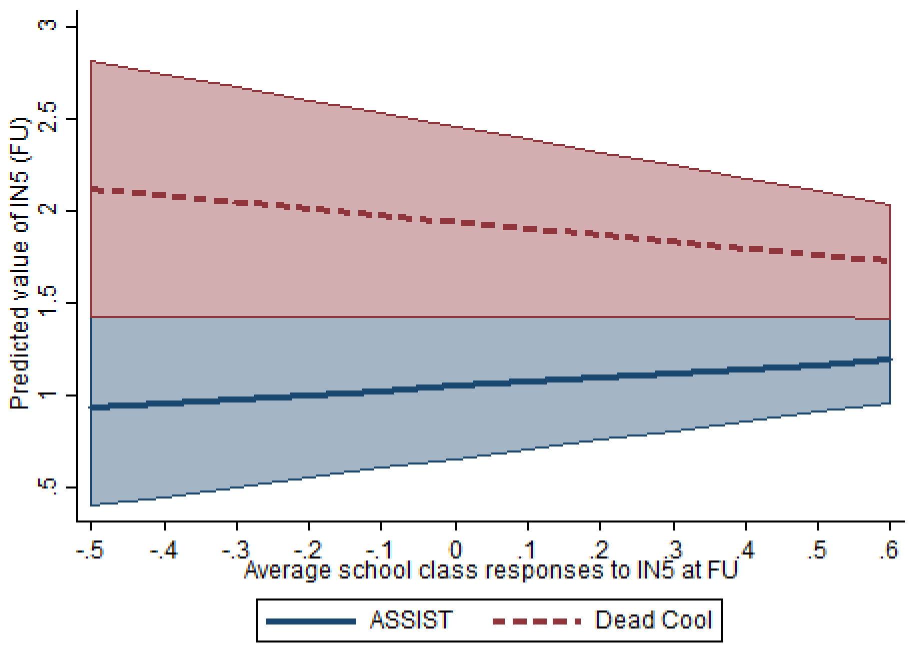

Figure S4.25. The conditional effects of peer influence from average school class responses to IN5 at follow-up (predictor) on focal participants' values of IN5 at follow-up (outcome) for participants in ASSIST and Dead Cool schools (moderator: intervention) with 95% CI limits for each slope.

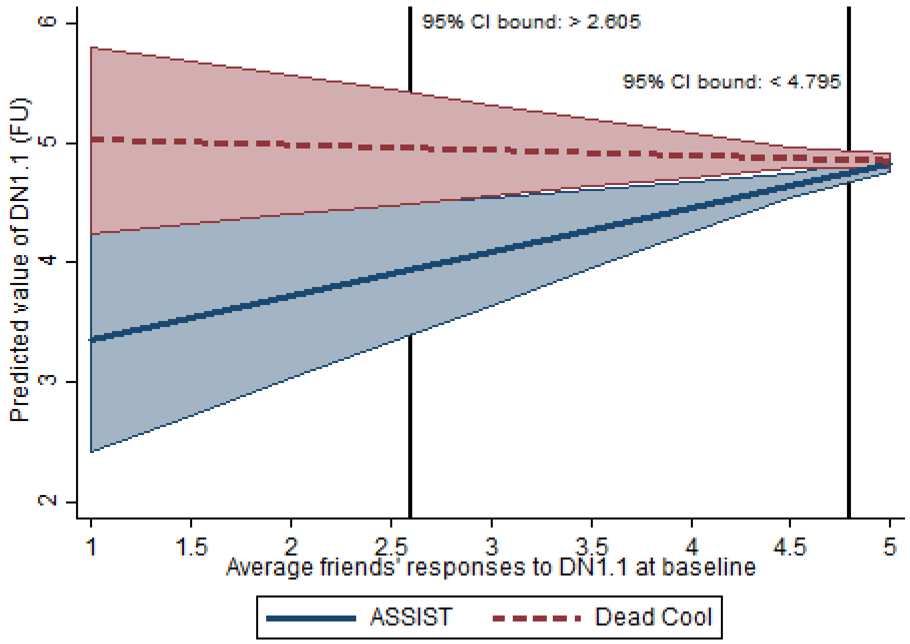

Figure S4.26. The conditional effects of peer influence from average friends' responses to DN1.1 at baseline (predictor) on focal participants' values of DN1.1 at follow-up (outcome) for participants in ASSIST and Dead Cool schools (moderator: intervention) with 95% CI limits for each slope, and bounds indicating regions of significance at the 95% level (indicating values of the predictor for which the slopes differ significantly for ASSIST and Dead Cool).

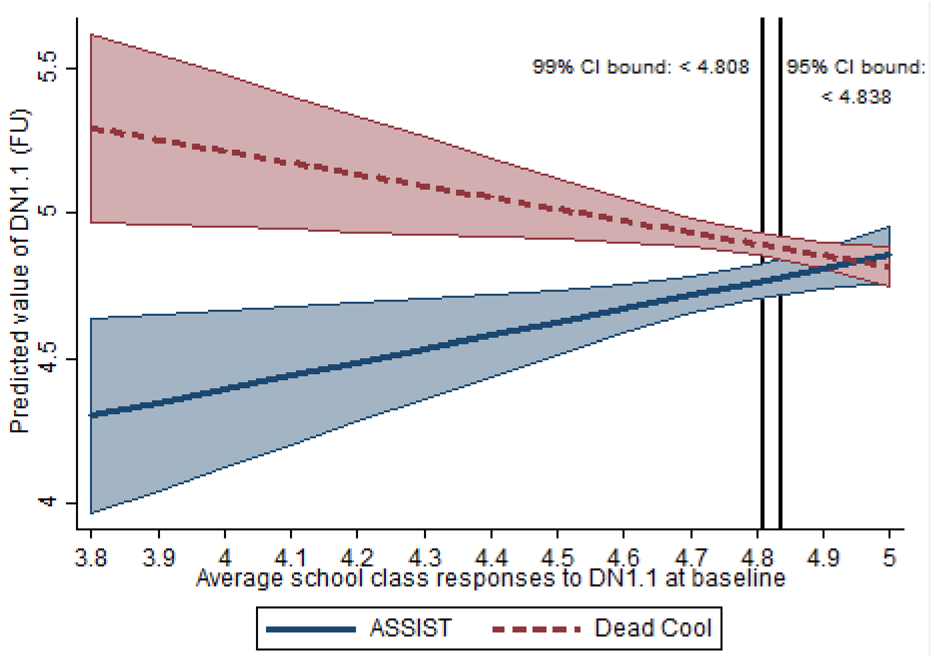

Figure S4.27. The conditional effects of peer influence from average school class responses to DN1.1 at baseline (predictor) on focal participants' values of DN1.1 at follow-up (outcome) for participants in ASSIST and Dead Cool schools (moderator: intervention) with 95% CI limits for each slope, and bounds indicating regions of significance at the 95% and 99% levels (indicating values of the predictor for which the slopes differ significantly for ASSIST and Dead Cool).

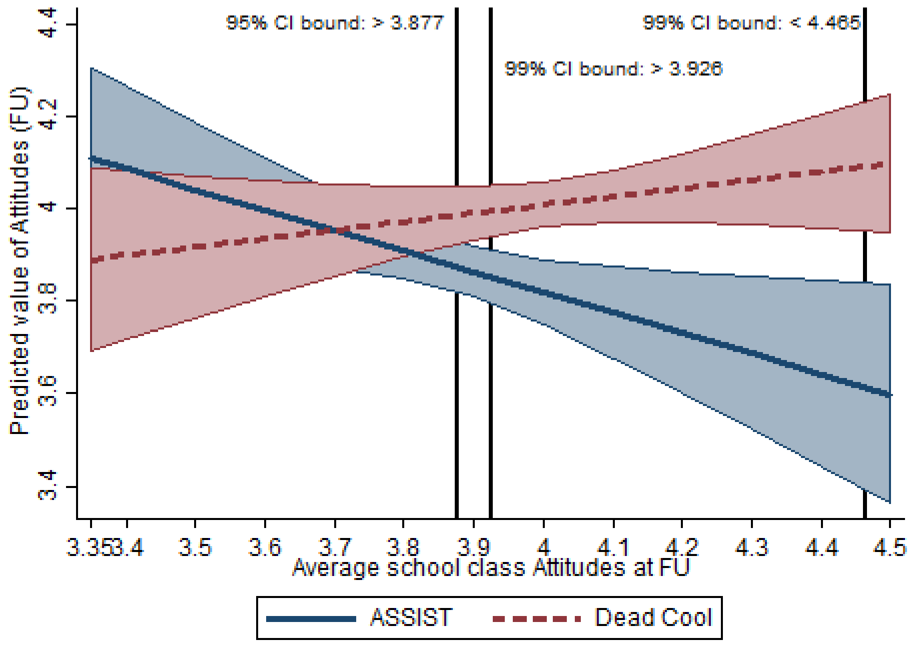

Figure S4.28. The conditional effects of peer influence from average school class attitudes at follow-up (predictor) on focal participants' attitudes at follow-up (outcome) for participants in ASSIST and Dead Cool schools (moderator: intervention) with 95% CI limits for each slope, and bounds indicating regions of significance at the 95% and 99% levels (indicating values of the predictor for which the slopes differ significantly for ASSIST and Dead Cool).

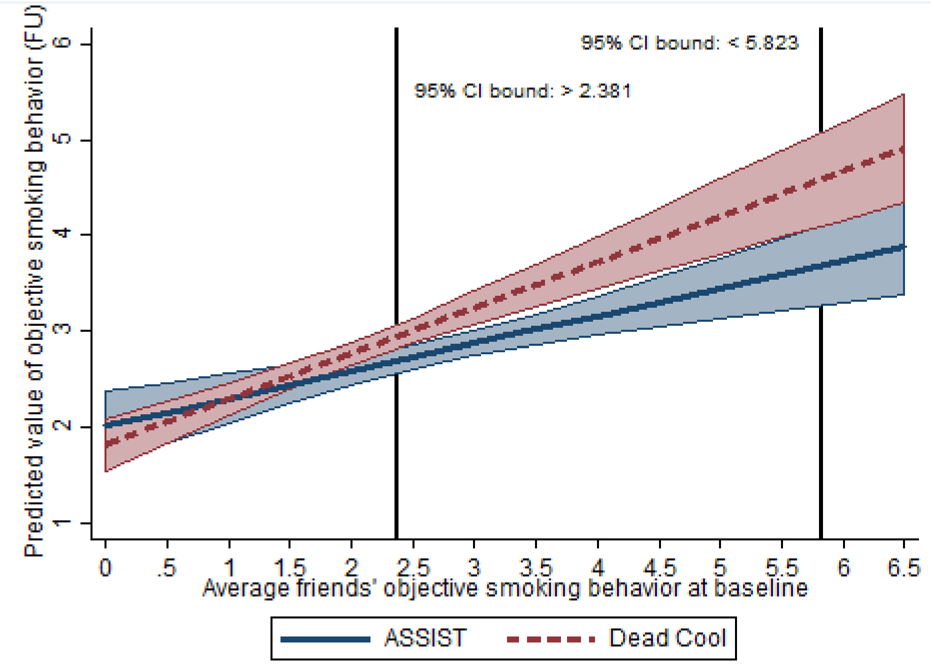

Figure S4.29. The conditional effects of peer influence from average friends' objectively measured smoking behavior at baseline (predictor) on focal participants' objectively measured smoking behavior at follow-up (outcome) for participants in ASSIST and Dead Cool schools (moderator: intervention) with 95% CI limits for each slope, and bounds indicating regions of significance at the 95% level (indicating values of the predictor for which the slopes differ significantly for ASSIST and Dead Cool).

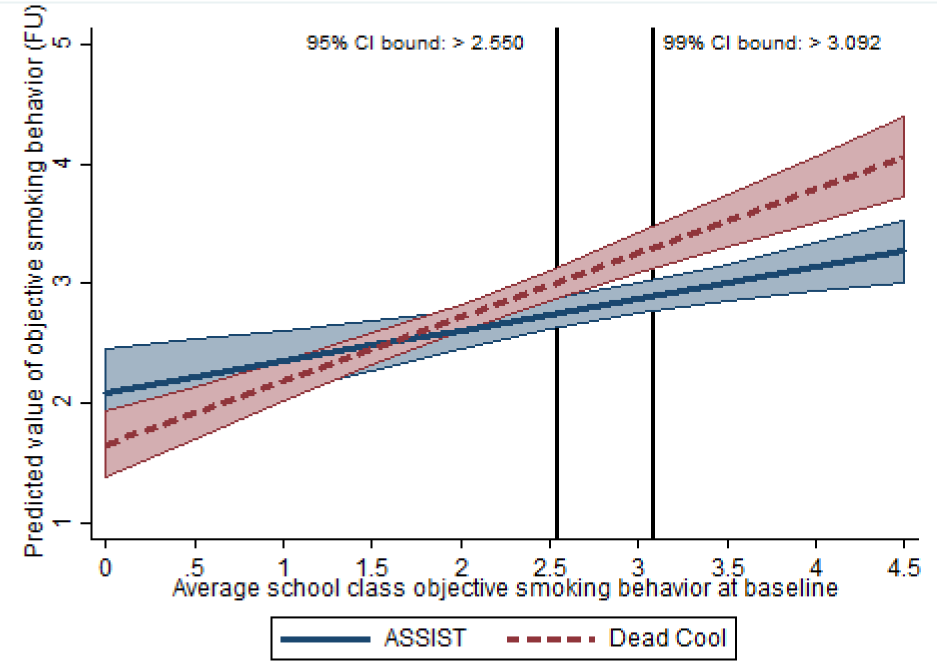

Figure S4.30. The conditional effects of peer influence from average school class objectively measured smoking behavior at baseline (predictor) on focal participants' objectively measured smoking behavior at follow-up (outcome) for participants in ASSIST and Dead Cool schools (moderator: intervention) with 95% CI limits for each slope, and bounds indicating regions of significance at the 95% and 99% levels (indicating values of the predictor for which the slopes differ significantly for ASSIST and Dead Cool).

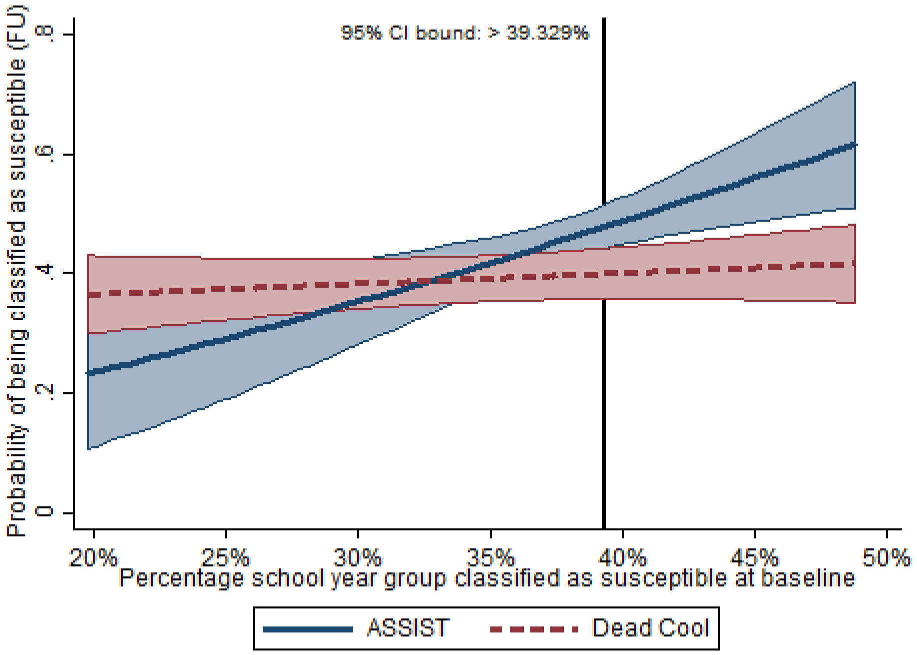

Figure S4.31. The conditional effects of peer influence from percentage school year group classified as susceptible to commencing smoking at baseline (predictor) on focal participants' probability of being classified as susceptible to commencing smoking at follow-up (outcome) for participants in ASSIST and Dead Cool schools (moderator: intervention) with 95% CI limits for each slope, and bounds indicating regions of significance at the 95% level (indicating values of the predictor for which the slopes differ significantly for ASSIST and Dead Cool).

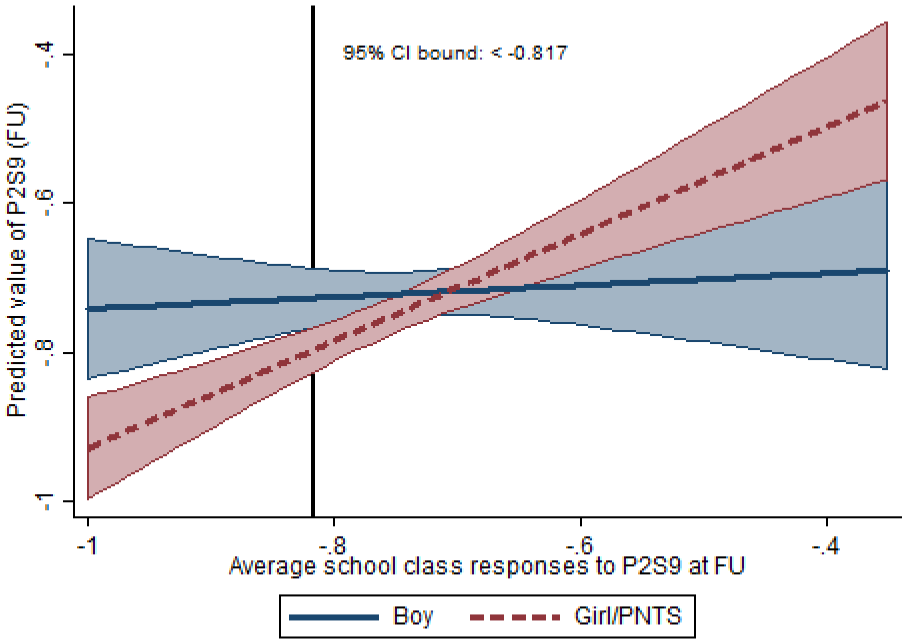

Figure S4.32. The conditional effects of peer influence from average school class responses for P2S9 at follow-up (predictor) on focal participants' values of P2S9 at follow-up (outcome) for boys and girls/PNTS (moderator: gender) with 95% CI limits for each slope, and bounds indicating regions of significance at the 95% level (indicating values of the predictor for which the slopes differ significantly for boys and girls/PNTS).

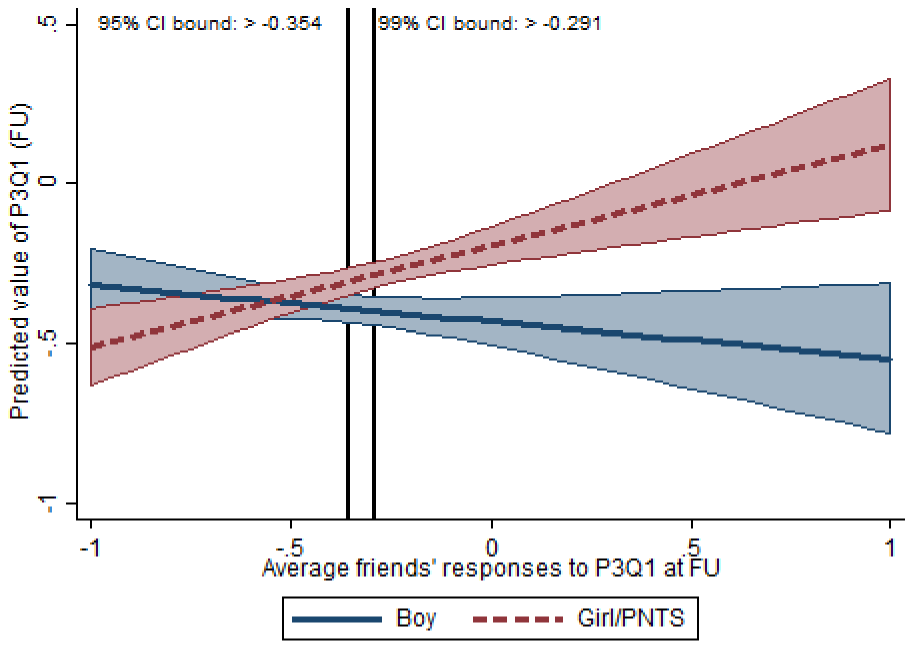

Figure S4.33. The conditional effects of peer influence from average friends' responses for P3Q1 at follow-up (predictor) on focal participants' values of P3Q1 at follow-up (outcome) for boys and girls/PNTS (moderator: gender) with 95% CI limits for each slope, and bounds indicating regions of significance at the 95% and 99% levels (indicating values of the predictor for which the slopes differ significantly for boys and girls/PNTS).

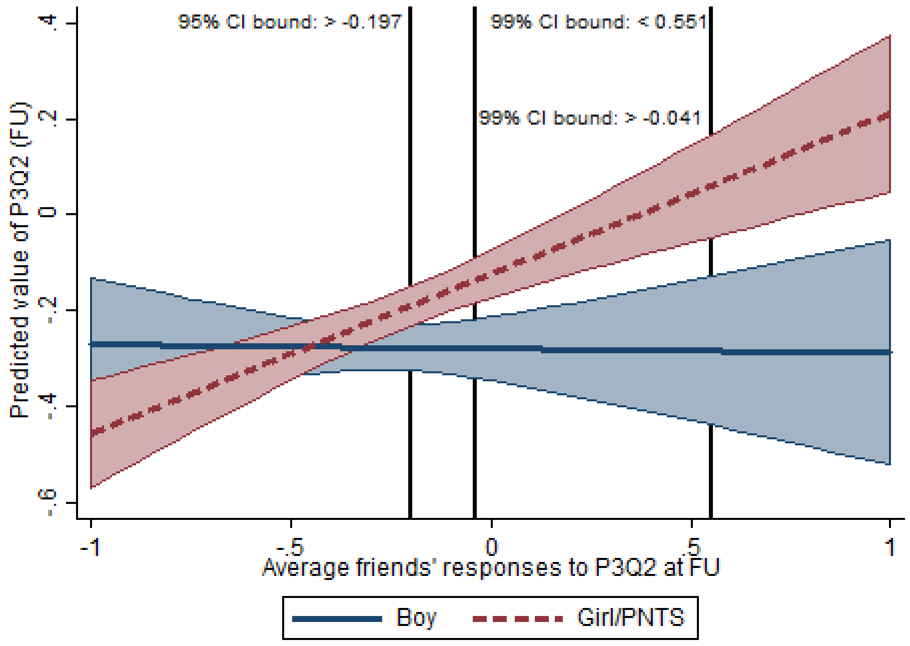

Figure S4.34. The conditional effects of peer influence from average friends' responses for P3Q2 at follow-up (predictor) on focal participants' values of P3Q2 at follow-up (outcome) for boys and girls/PNTS (moderator: gender) with 95% CI limits for each slope, and bounds indicating regions of significance at the 95% and 99% levels (indicating values of the predictor for which the slopes differ significantly for boys and girls/PNTS).

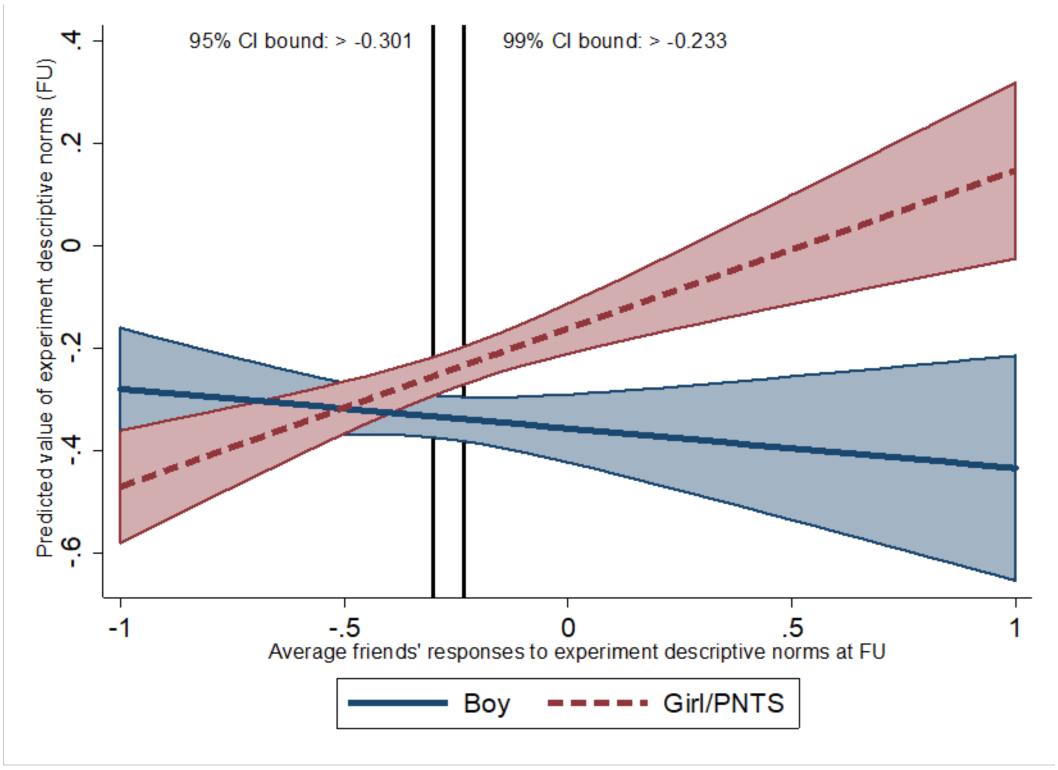

Figure S4.35. The conditional effects of peer influence from average friends' responses for experiment part 3 (descriptive norms, average P3Q1 to P3Q2) at follow-up (predictor) on focal participants' values of experiment part 3 at follow-up (outcome) for boys and girls/PNTS (moderator: gender) with 95% CI limits for each slope, and bounds indicating regions of significance at the 95% and 99% levels (indicating values of the predictor for which the slopes differ significantly for boys and girls/PNTS).

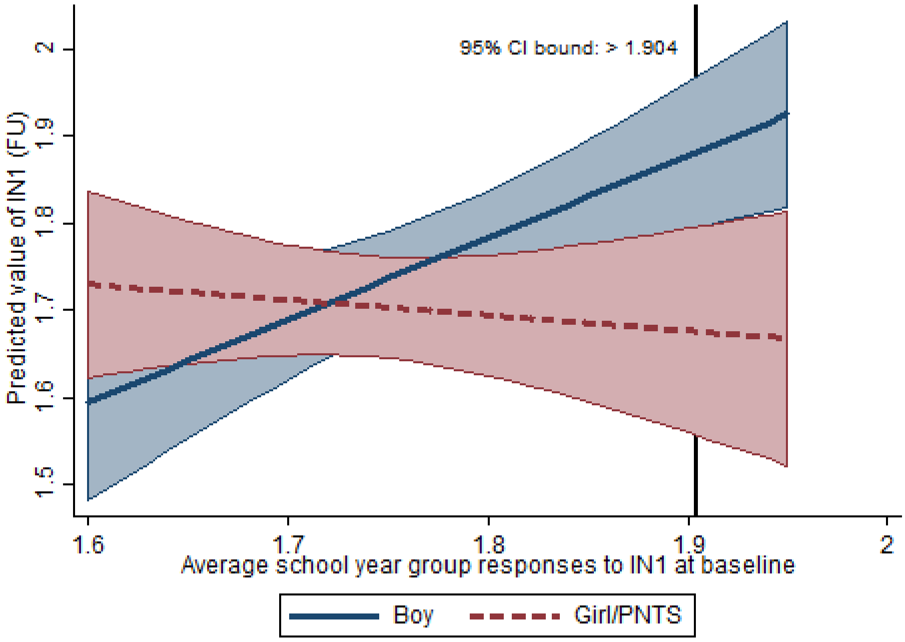

Figure S4.36. The conditional effects of peer influence from average school year responses for IN1 at baseline (predictor) on focal participants' values of IN1 at follow-up (outcome) for boys and girls/PNTS (moderator: gender) with 95% CI limits for each slope, and bounds indicating regions of significance at the 95% level (indicating values of the predictor for which the slopes differ significantly for boys and girls/PNTS).

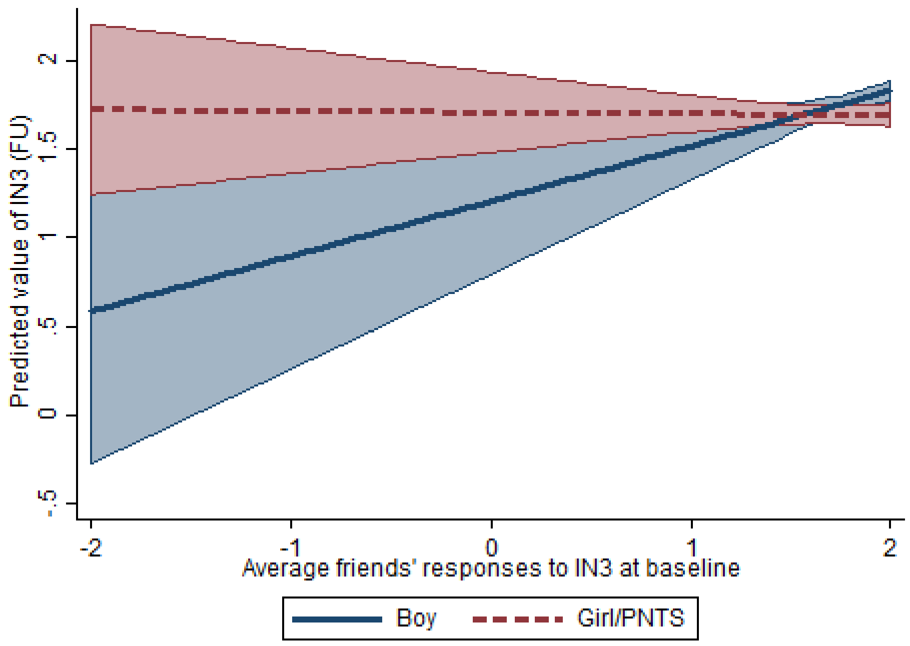

Figure S4.37. The conditional effects of peer influence from average friends' responses for IN3 at baseline (predictor) on focal participants' values of IN3 at follow-up (outcome) for boys and girls/PNTS (moderator: gender) with 95% CI limits for each slope.

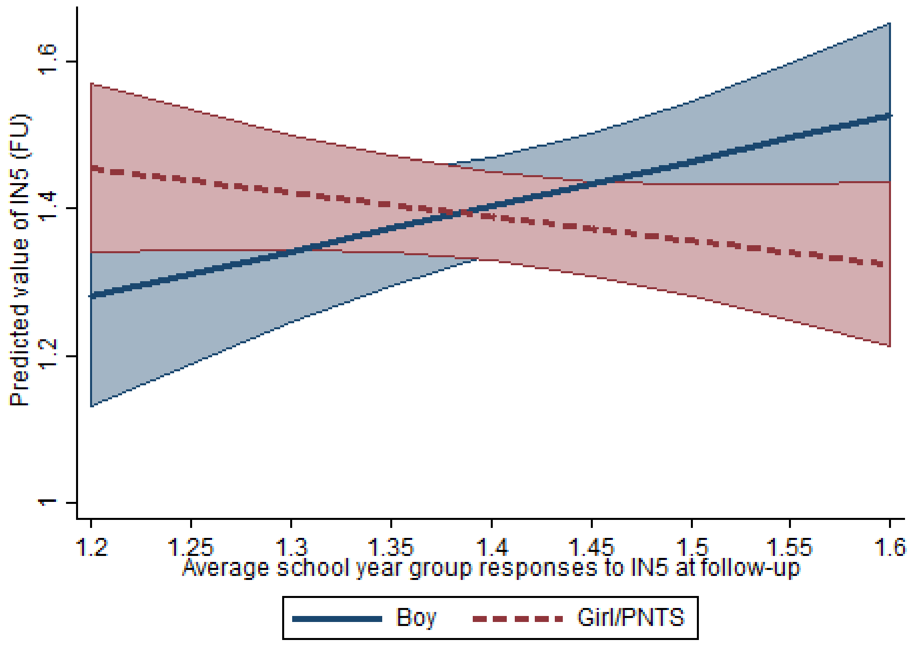

Figure S4.38. The conditional effects of peer influence from average school year group responses for IN5 at follow-up (predictor) on focal participants' values of IN5 at follow-up (outcome) for boys and girls/PNTS (moderator: gender) with 95% CI limits for each slope.

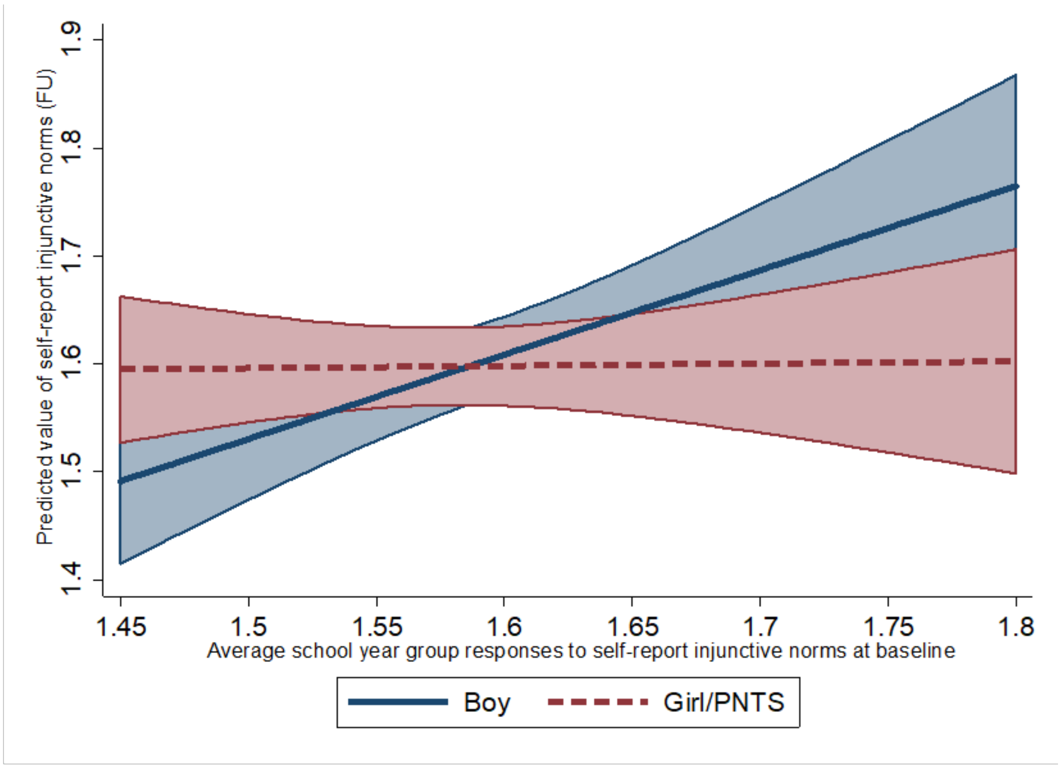

Figure S4.39. The conditional effects of peer influence from average school year group responses for self-report injunctive norms (average IN1 to IN7) at baseline (predictor) on focal participants' values of self-report injunctive norms at follow-up (outcome) for boys and girls/PNTS (moderator: gender) with 95% CI limits for each slope.

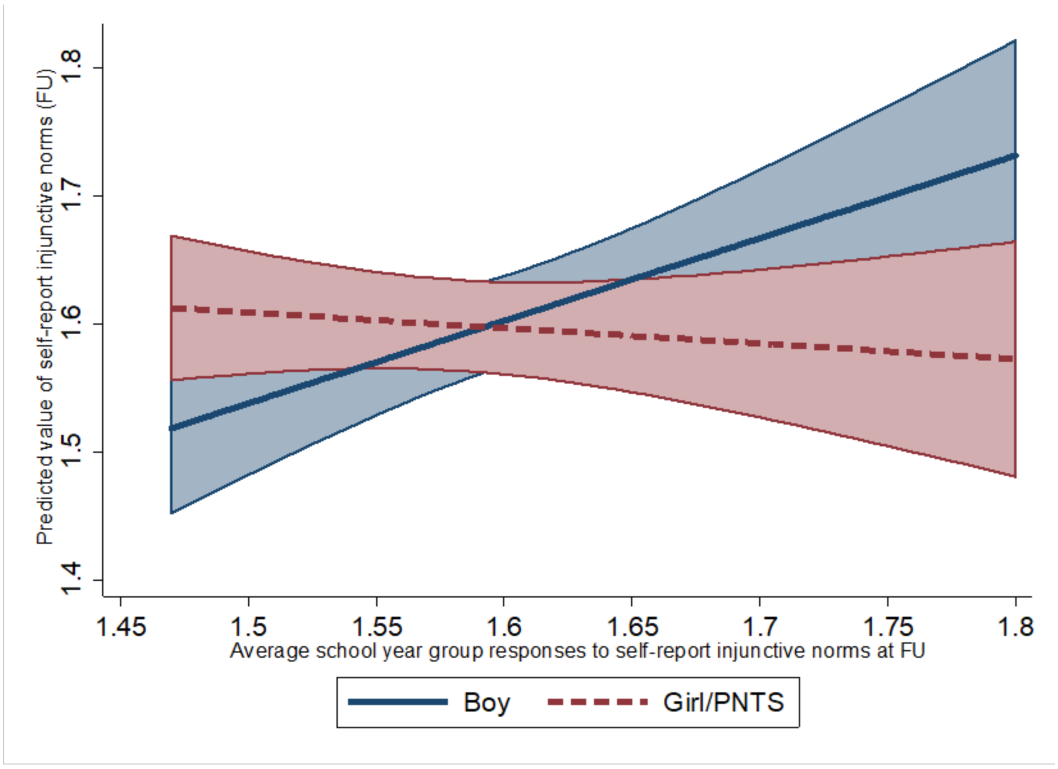

Figure S4.40. The conditional effects of peer influence from average school year group responses for self-report injunctive norms (average IN1 to IN7) at follow-up (predictor) on focal participants' values of self-report injunctive norms at follow-up (outcome) for boys and girls/PNTS (moderator: gender) with 95% CI limits for each slope.

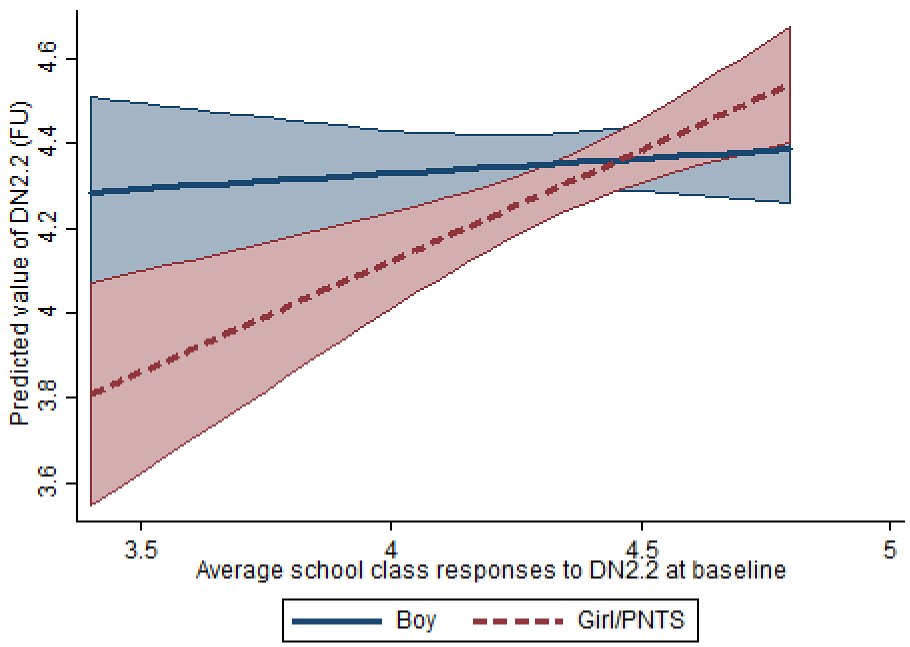

Figure S4.41. The conditional effects of peer influence from average school class responses for DN2.2 at baseline (predictor) on focal participants' values of DN2.2 at follow-up (outcome) for boys and girls/PNTS (moderator: gender) with 95% CI limits for each slope.

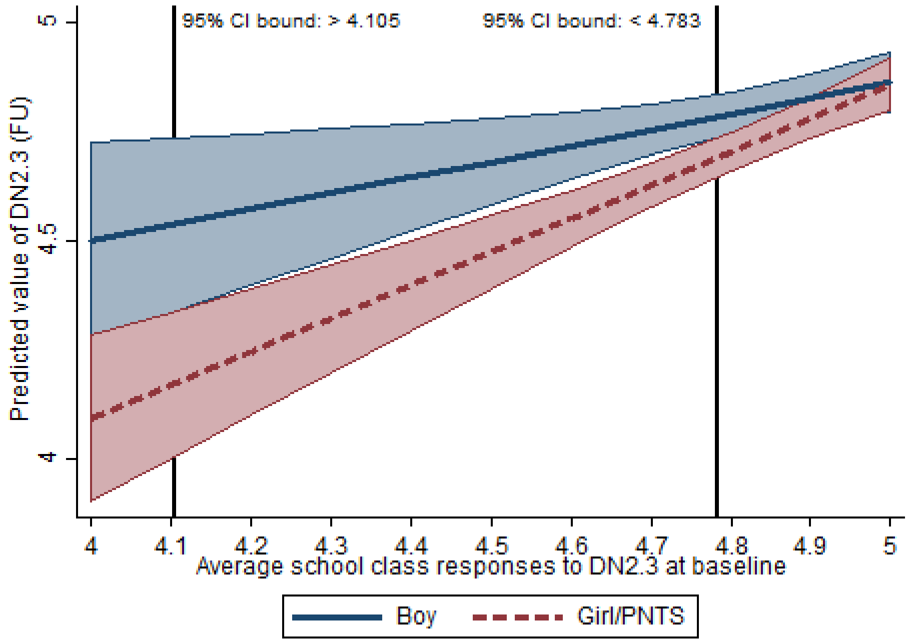

Figure S4.42. The conditional effects of peer influence from average school class responses for DN2.3 at baseline (predictor) on focal participants' values of DN2.3 at follow-up (outcome) for boys and girls/PNTS (moderator: gender) with 95% CI limits for each slope, and bounds indicating regions of significance at the 95% level (indicating values of the predictor for which the slopes differ significantly for boys and girls/PNTS).

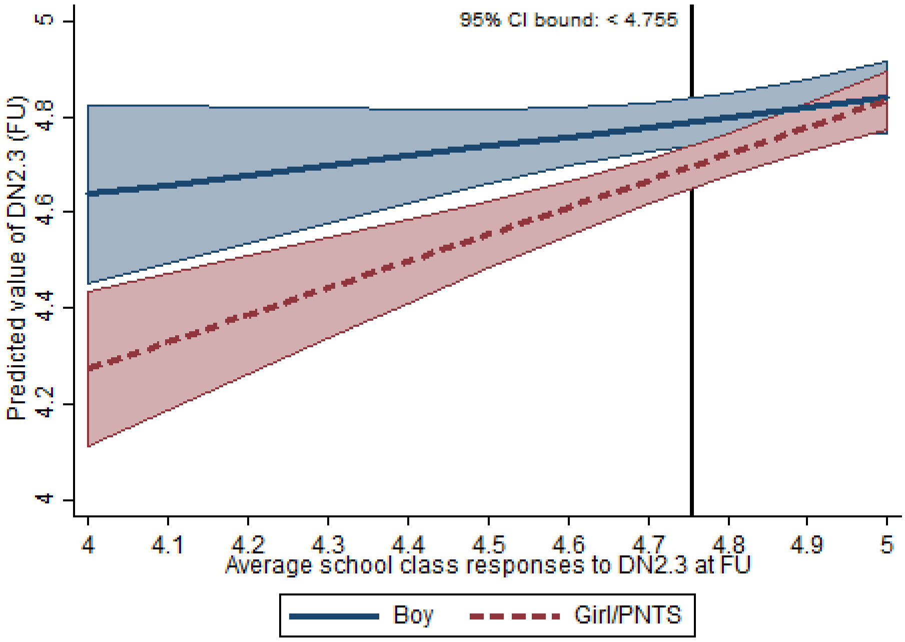

Figure S4.43. The conditional effects of peer influence from average school class responses for DN2.3 at follow-up (predictor) on focal participants' values of DN2.3 at follow-up (outcome) for boys and girls/PNTS (moderator: gender) with 95% CI limits for each slope, and bounds indicating regions of significance at the 95% level (indicating values of the predictor for which the slopes differ significantly for boys and girls/PNTS).

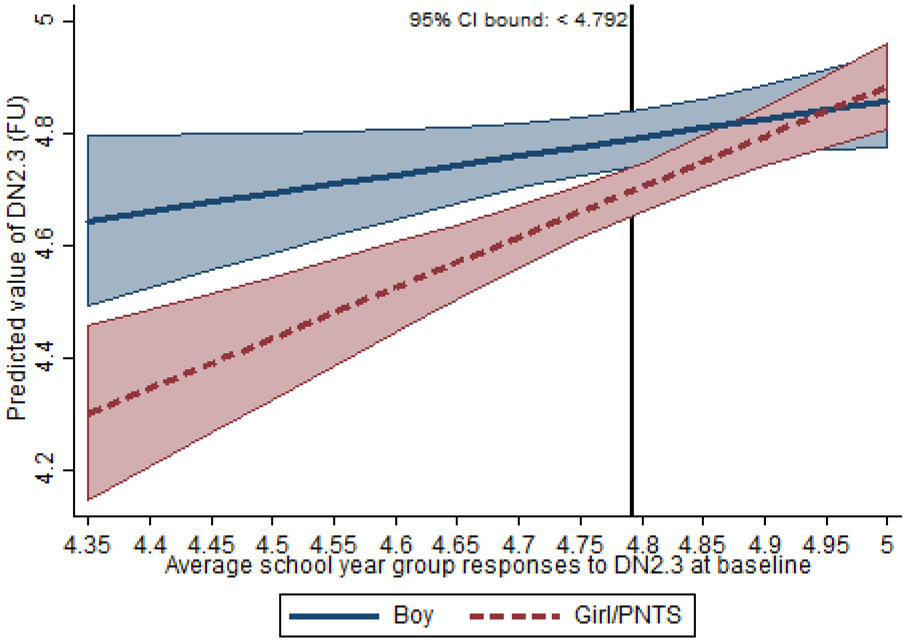

Figure S4.44. The conditional effects of peer influence from average school year group responses for DN2.3 at baseline (predictor) on focal participants' values of DN2.3 at follow-up (outcome) for boys and girls/PNTS (moderator: gender) with 95% CI limits for each slope, and bounds indicating regions of significance at the 95% level (indicating values of the predictor for which the slopes differ significantly for boys and girls/PNTS).

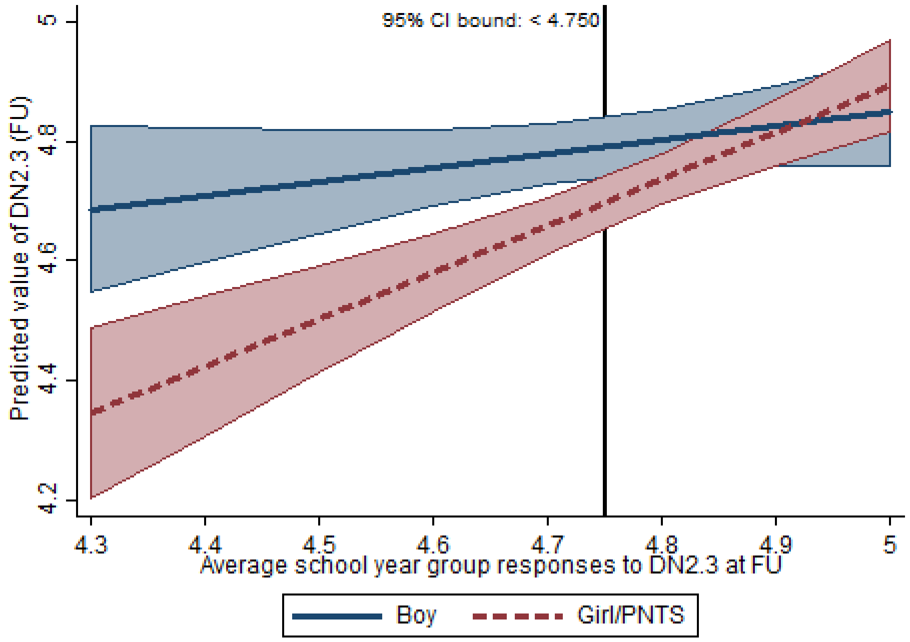

Figure S4.45. The conditional effects of peer influence from average school year group responses for DN2.3 at follow-up (predictor) on focal participants' values of DN2.3 at follow-up (outcome) for boys and girls/PNTS (moderator: gender) with 95% CI limits for each slope, and bounds indicating regions of significance at the 95% level (indicating values of the predictor for which the slopes differ significantly for boys and girls/PNTS).

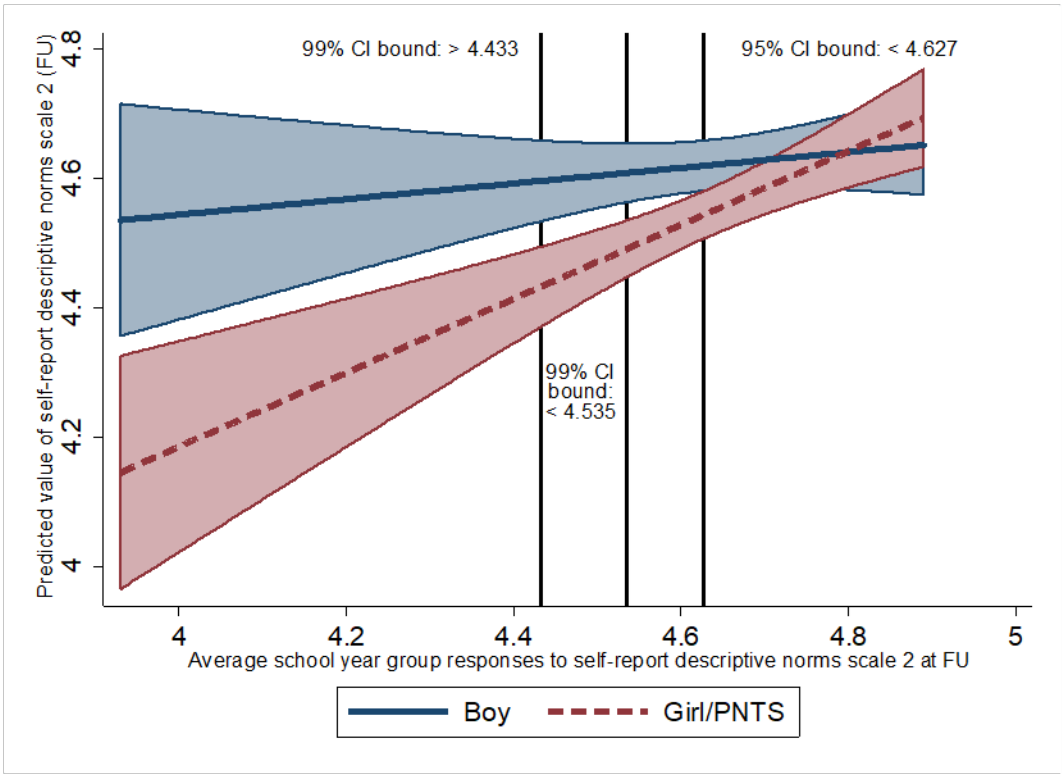

Figure S4.46. The conditional effects of peer influence from average school class responses for self-report descriptive norms scale 2 (average DN2.1 to DN2.3) at baseline (predictor) on focal participants' values of self-report descriptive norms scale 2 at follow-up (outcome) for boys and girls/PNTS (moderator: gender) with 95% CI limits for each slope, and bounds indicating regions of significance at the 95% and 99% levels (indicating values of the predictor for which the slopes differ significantly for boys and girls/PNTS).

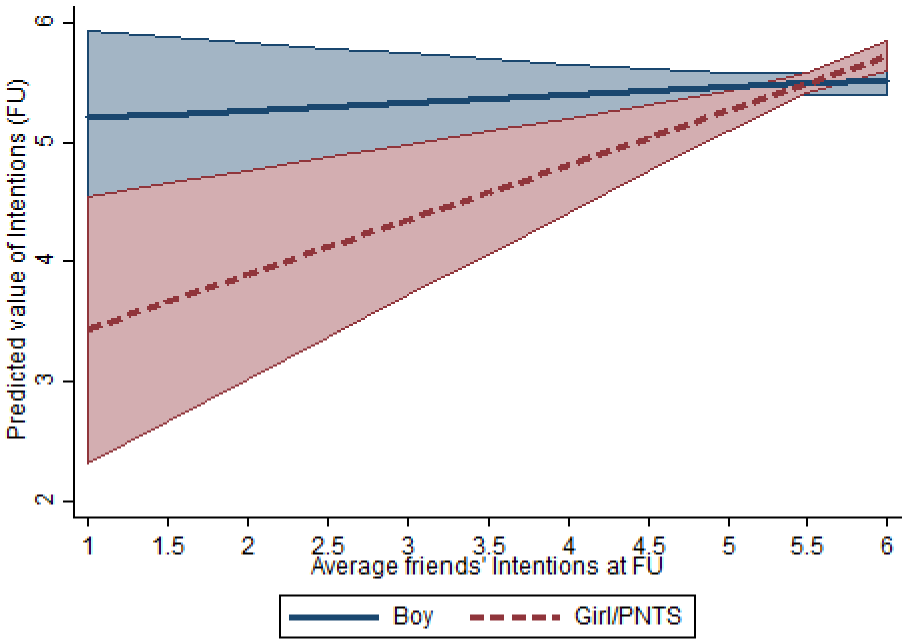

Figure S4.47. The conditional effects of peer influence from average friends' intentions at follow-up (predictor) on focal participants' intentions at follow-up (outcome) for boys and girls/PNTS (moderator: gender) with 95% CI limits for each slope.

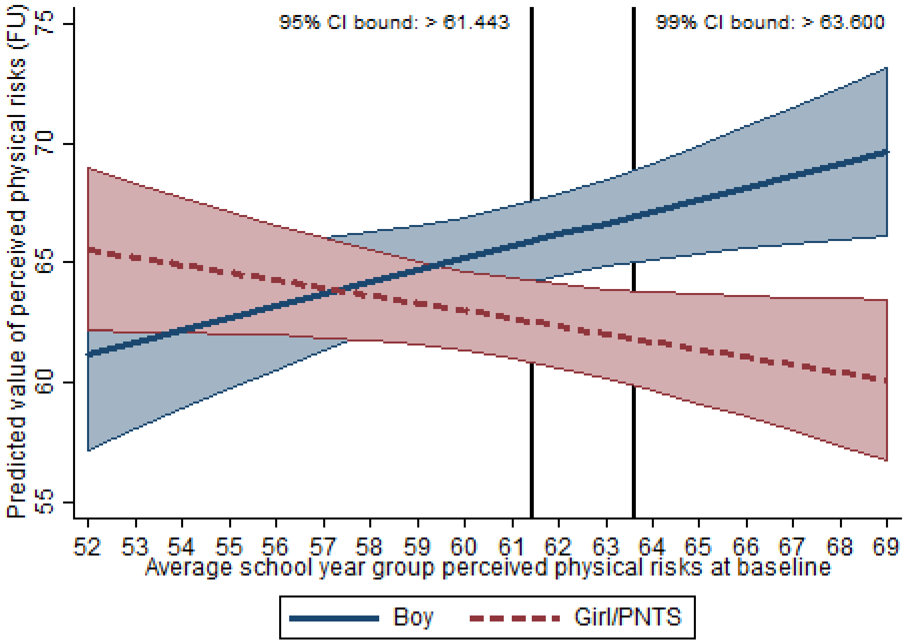

Figure S4.48. The conditional effects of peer influence from average school year group perceived physical risks at baseline (predictor) on focal participants' perceived physical risks at follow-up (outcome) for boys and girls/PNTS (moderator: gender) with 95% CI limits for each slope, and bounds indicating regions of significance at the 95% and 99% levels (indicating values of the predictor for which the slopes differ significantly for boys and girls/PNTS).

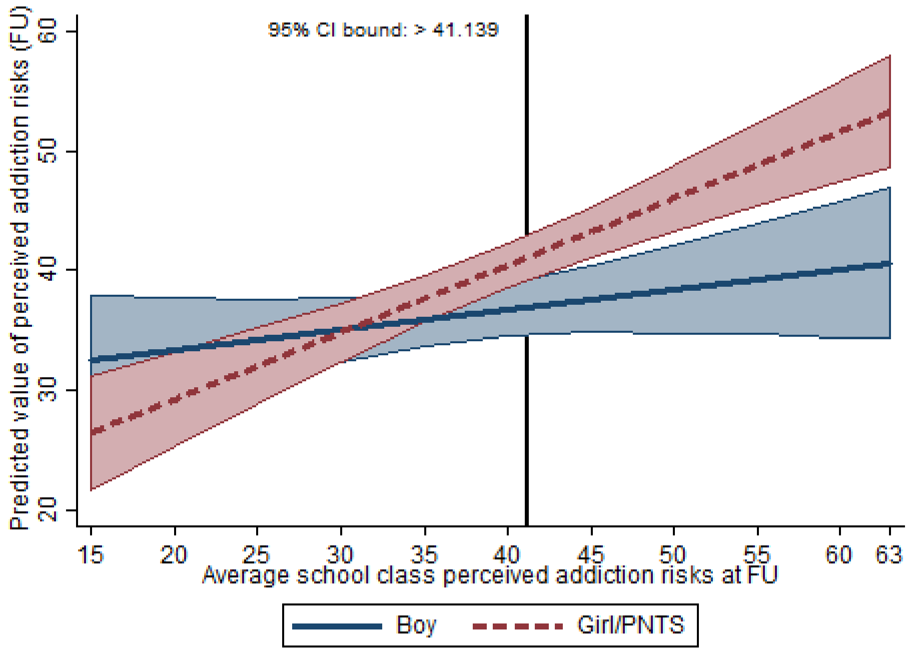

Figure S4.49. The conditional effects of peer influence from average school class perceived addiction risks at follow-up (predictor) on focal participants' perceived addiction risks at follow-up (outcome) for boys and girls/PNTS (moderator: gender) with 95% CI limits for each slope, and bounds indicating regions of significance at the 95% level (indicating values of the predictor for which the slopes differ significantly for boys and girls/PNTS).

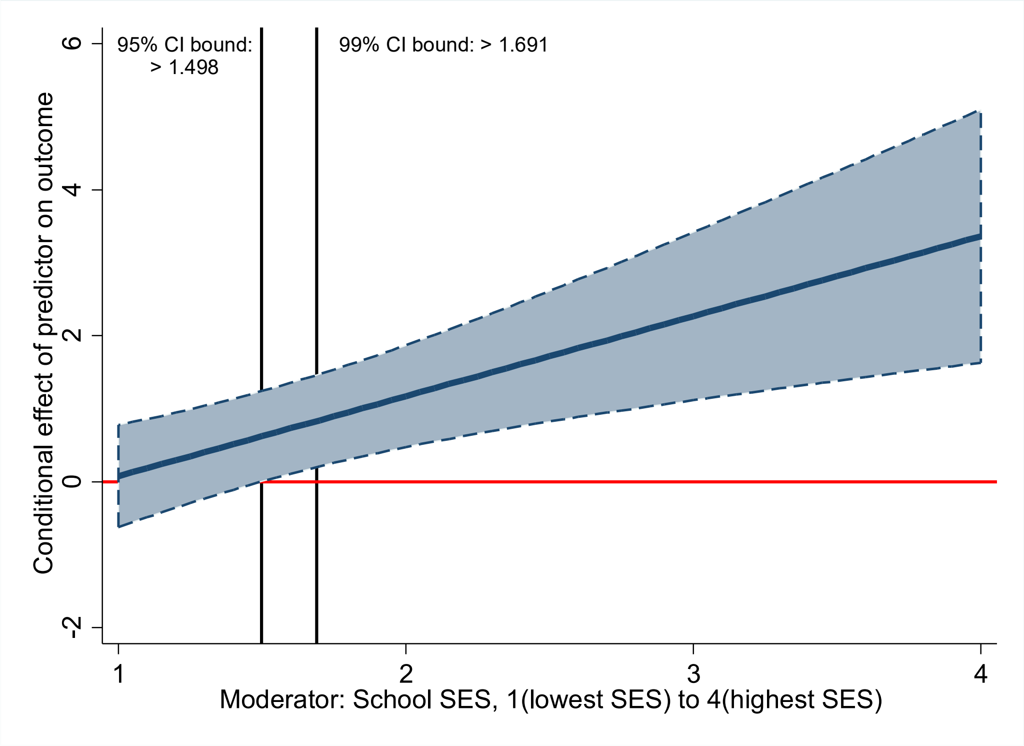

Figure S4.50. The conditional effects of peer influence from average school year group responses for P2S4 at baseline (predictor) on focal participants' values of P2S4 at follow-up (outcome) by school socio-economic status in all schools (moderator) with 95% CI limits for conditional effects, and bounds indicating regions of significance at the 95% and 99% levels (indicating values of the moderator for which conditional effects differ significantly from 0).

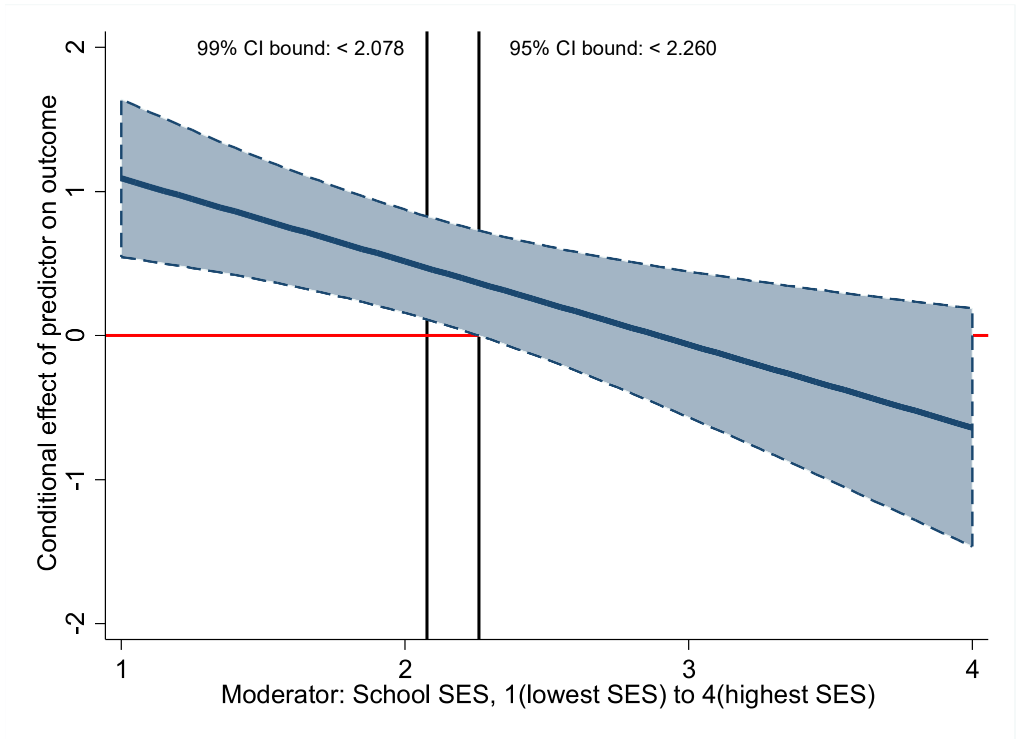

Figure S4.51. The conditional effects of peer influence from average school year group responses for P2S6 at baseline (predictor) on focal participants' values of P2S6 at follow-up (outcome) by school socio-economic status in all schools (moderator) with 95% CI limits for conditional effects, and bounds indicating regions of significance at the 95% and 99% levels (indicating values of the moderator for which conditional effects differ significantly from 0).

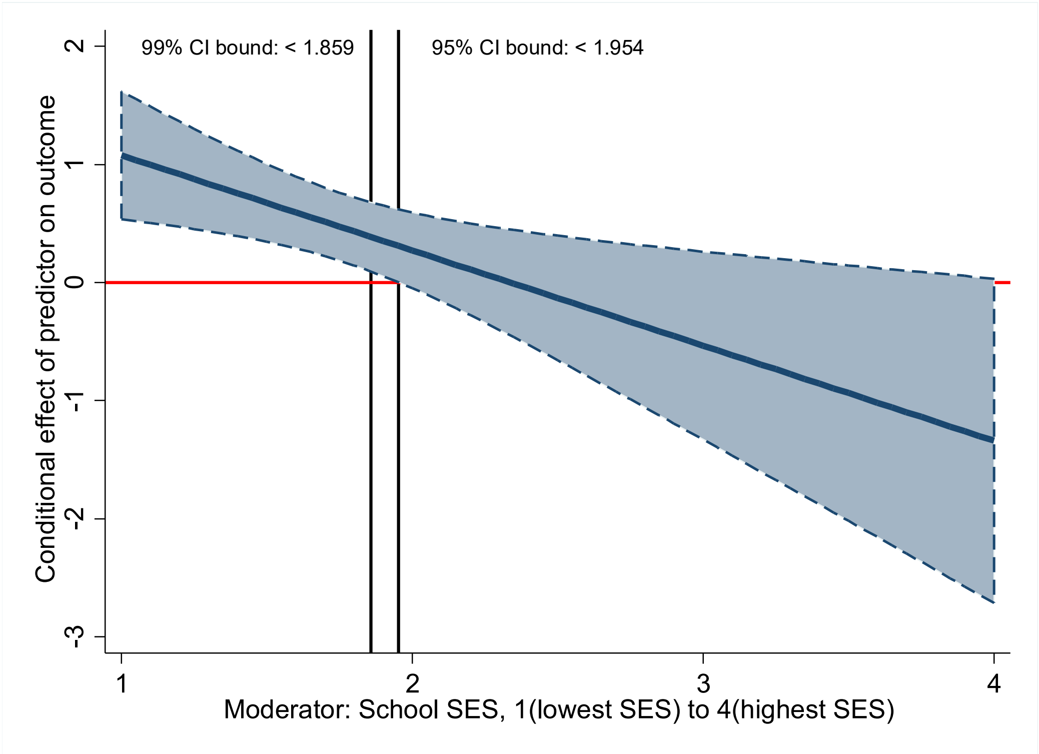

Figure S4.52. The conditional effects of peer influence from average school year group responses for P2S6 at follow-up (predictor) on focal participants' values of P2S6 at follow-up (outcome) by school socio-economic status in all schools (moderator) with 95% CI limits for conditional effects, and bounds indicating regions of significance at the 95% and 99% levels (indicating values of the moderator for which conditional effects differ significantly from 0).

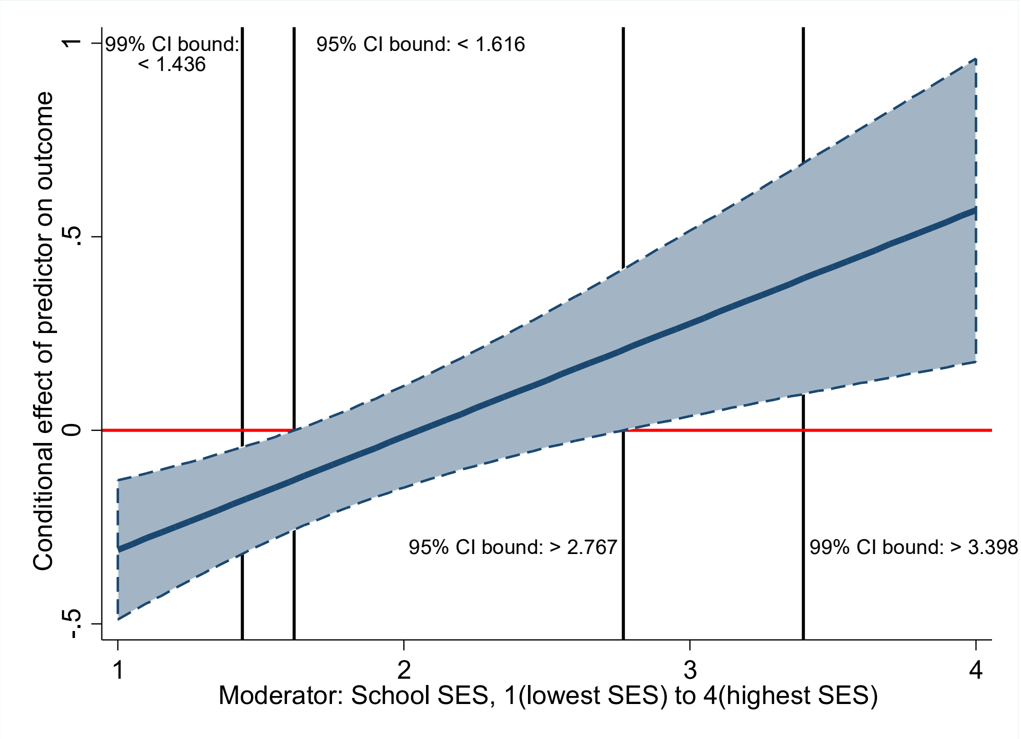

Figure S4.53. The conditional effects of peer influence from average friends' responses for IN1 at baseline (predictor) on focal participants' values of IN1 at follow-up (outcome) by school socio-economic status in all schools (moderator) with 95% CI limits for conditional effects, and bounds indicating regions of significance at the 95% and 99% levels (indicating values of the moderator for which conditional effects differ significantly from 0).

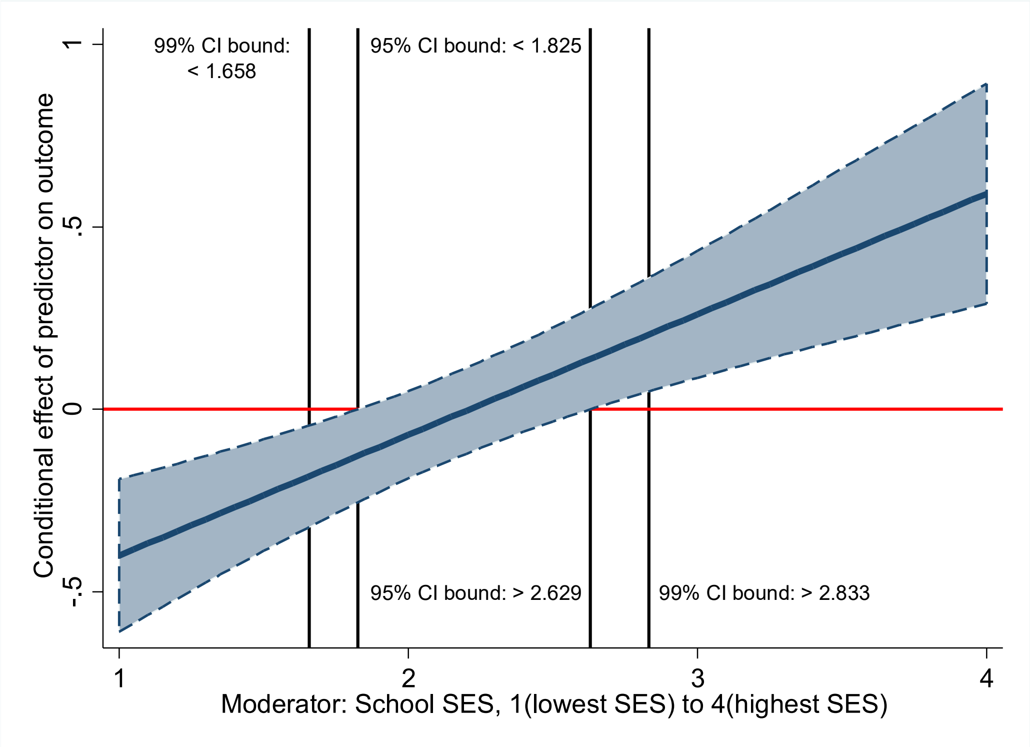

Figure S4.54. The conditional effects of peer influence from average friends' responses for IN1 at follow-up (predictor) on focal participants' values of IN1 at follow-up (outcome) by school socio-economic status in all schools (moderator) with 95% CI limits for conditional effects, and bounds indicating regions of significance at the 95% and 99% levels (indicating values of the moderator for which conditional effects differ significantly from 0).

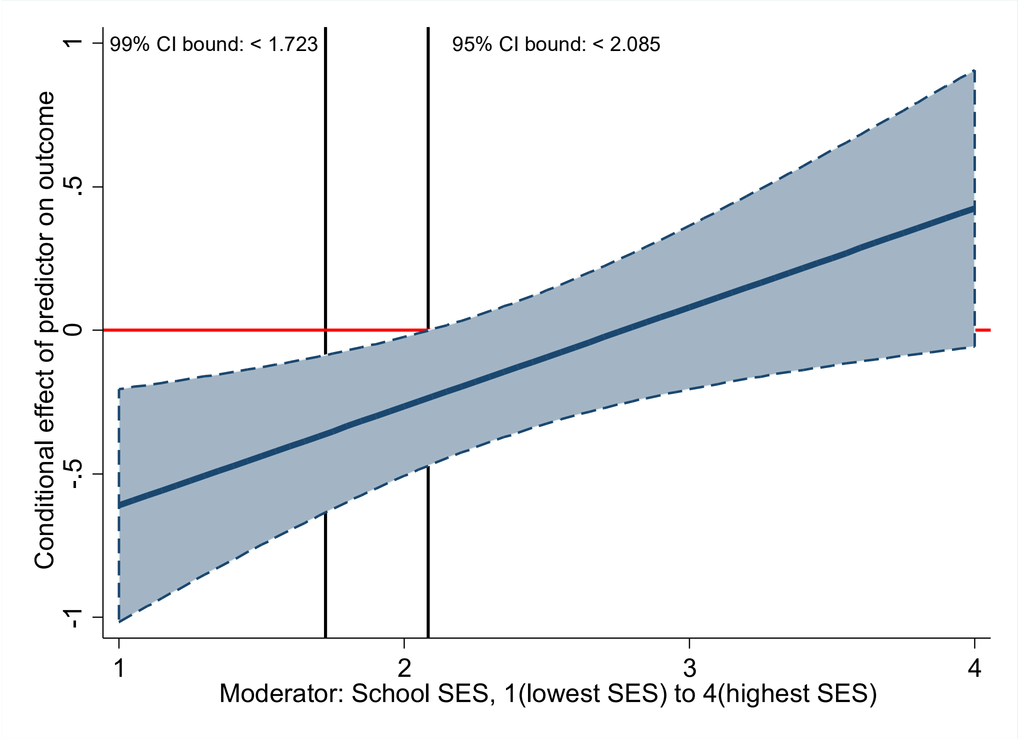

Figure S4.55. The conditional effects of peer influence from average school class responses for IN1 at follow-up (predictor) on focal participants' values of IN1 at follow-up (outcome) by school socio-economic status in all schools (moderator) with 95% CI limits for conditional effects, and bounds indicating regions of significance at the 95% and 99% levels (indicating values of the moderator for which conditional effects differ significantly from 0).

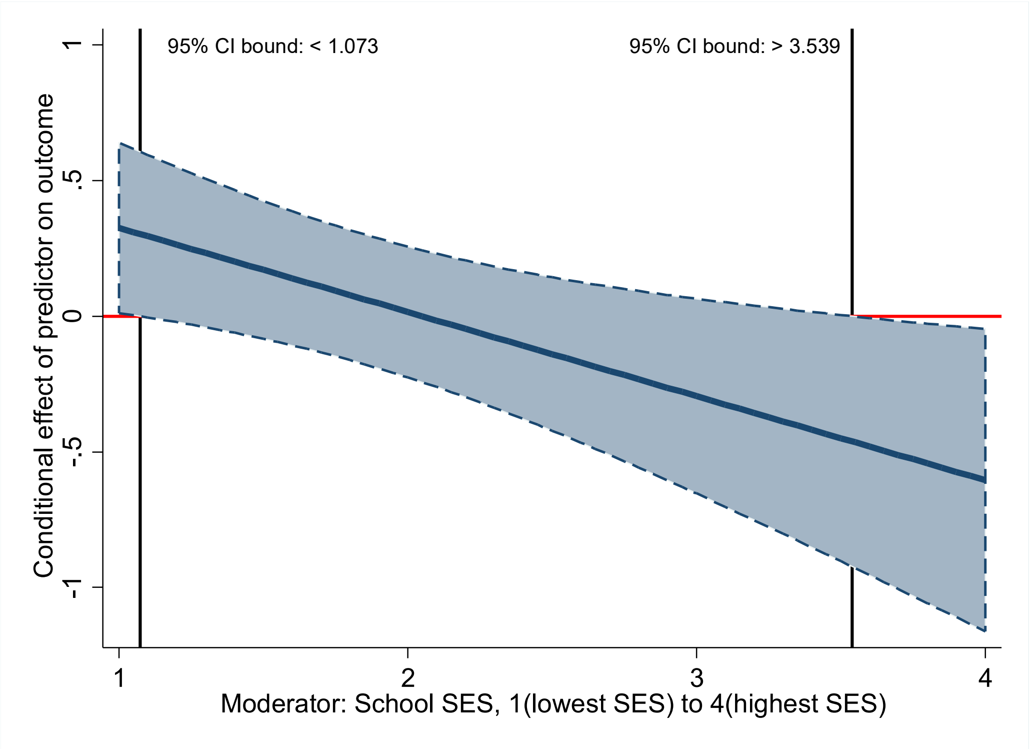

Figure S4.56. The conditional effects of peer influence from average school class responses for IN4 at baseline (predictor) on focal participants' values of IN4 at follow-up (outcome) by school socio-economic status in all schools (moderator) with 95% CI limits for conditional effects, and bounds indicating regions of significance at the 95% and 99% levels (indicating values of the moderator for which conditional effects differ significantly from 0).

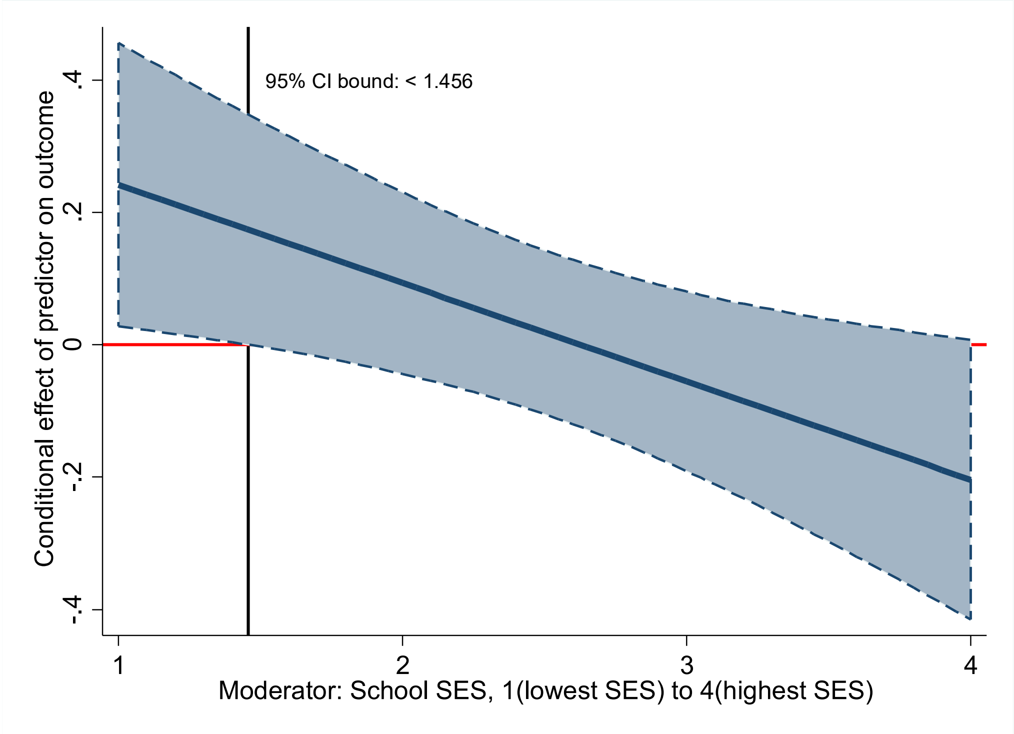

Figure S4.57. The conditional effects of peer influence from average friends' responses for IN5 at baseline (predictor) on focal participants' values of IN5 at follow-up (outcome) by school socio-economic status in all schools (moderator) with 95% CI limits for conditional effects, and bounds indicating regions of significance at the 95% and 99% levels (indicating values of the moderator for which conditional effects differ significantly from 0).

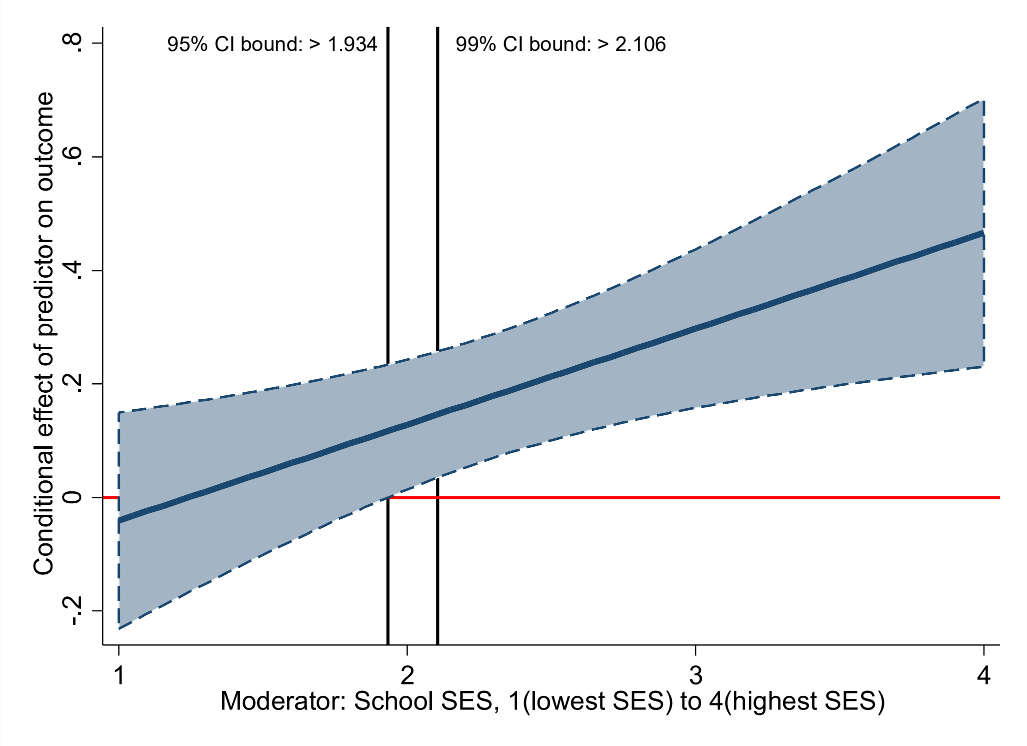

Figure S4.58. The conditional effects of peer influence from average friends' self-report injunctive norms (average IN1 to IN7) at follow-up (predictor) on focal participants' self-report injunctive norms at follow-up (outcome) by school socio-economic status in all schools (moderator) with 95% CI limits for conditional effects, and bounds indicating regions of significance at the 95% and 99% levels (indicating values of the moderator for which conditional effects differ significantly from 0).

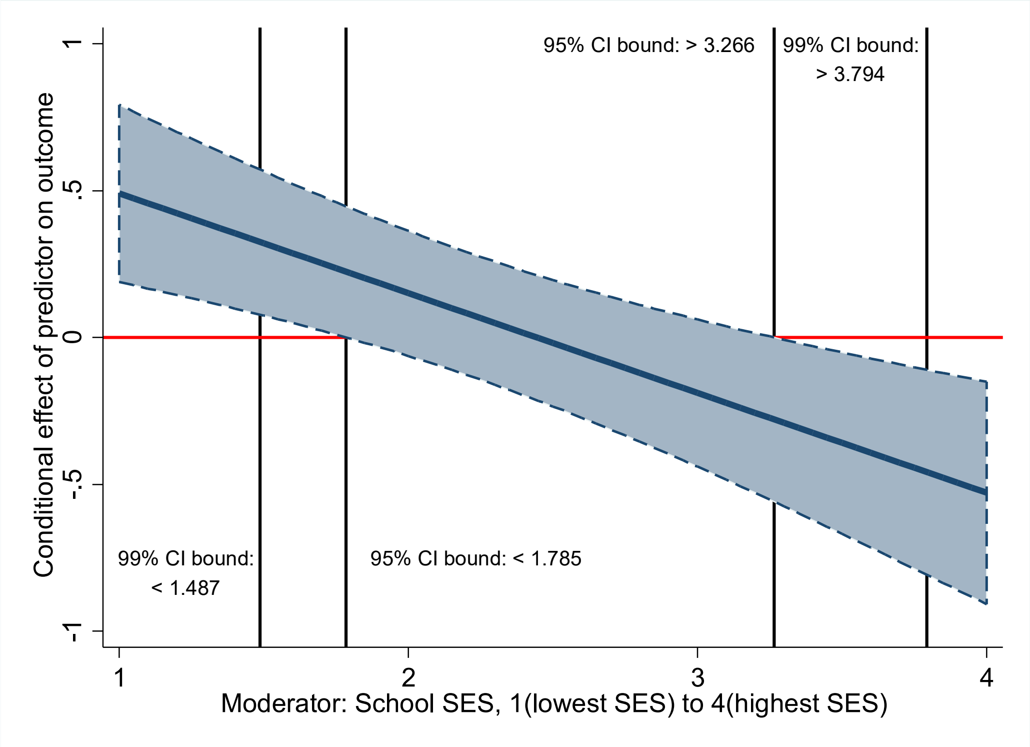

Figure S4.59. The conditional effects of peer influence from average friends' responses for DN1.1 at baseline (predictor) on focal participants' values of DN1.1 at follow-up (outcome) by school socio-economic status in all schools (moderator) with 95% CI limits for conditional effects, and bounds indicating regions of significance at the 95% and 99% levels (indicating values of the moderator for which conditional effects differ significantly from 0).

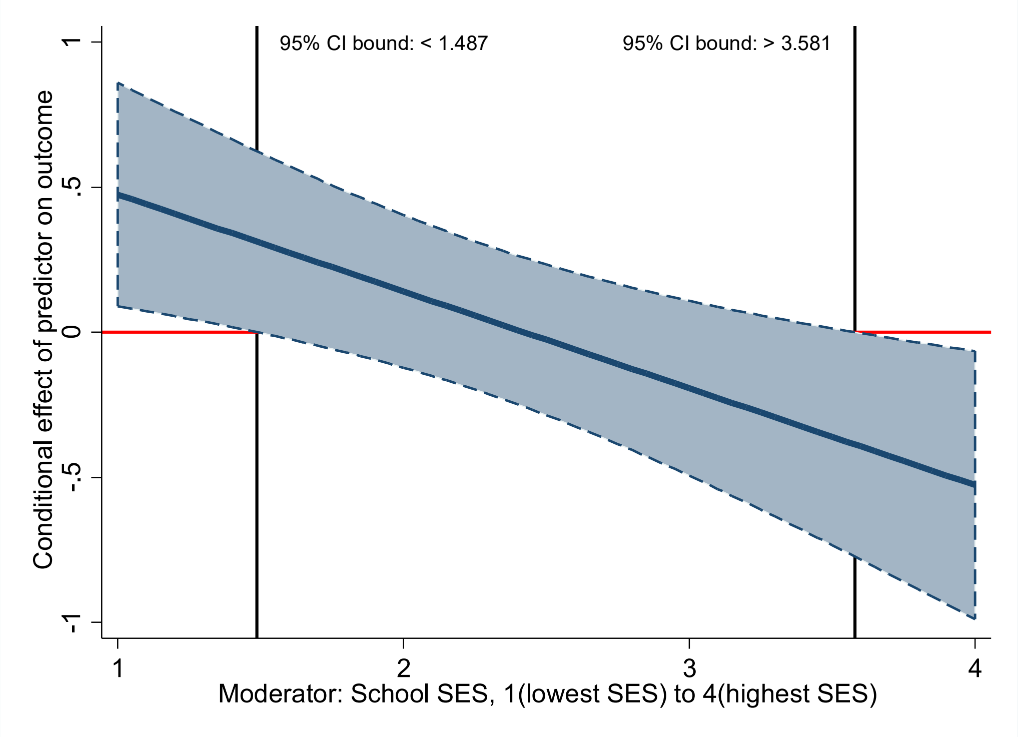

Figure S4.60. The conditional effects of peer influence from average school class responses for DN1.1 at baseline (predictor) on focal participants' values of DN1.1 at follow-up (outcome) by school socio-economic status in all schools (moderator) with 95% CI limits for conditional effects, and bounds indicating regions of significance at the 95% and 99% levels (indicating values of the moderator for which conditional effects differ significantly from 0).

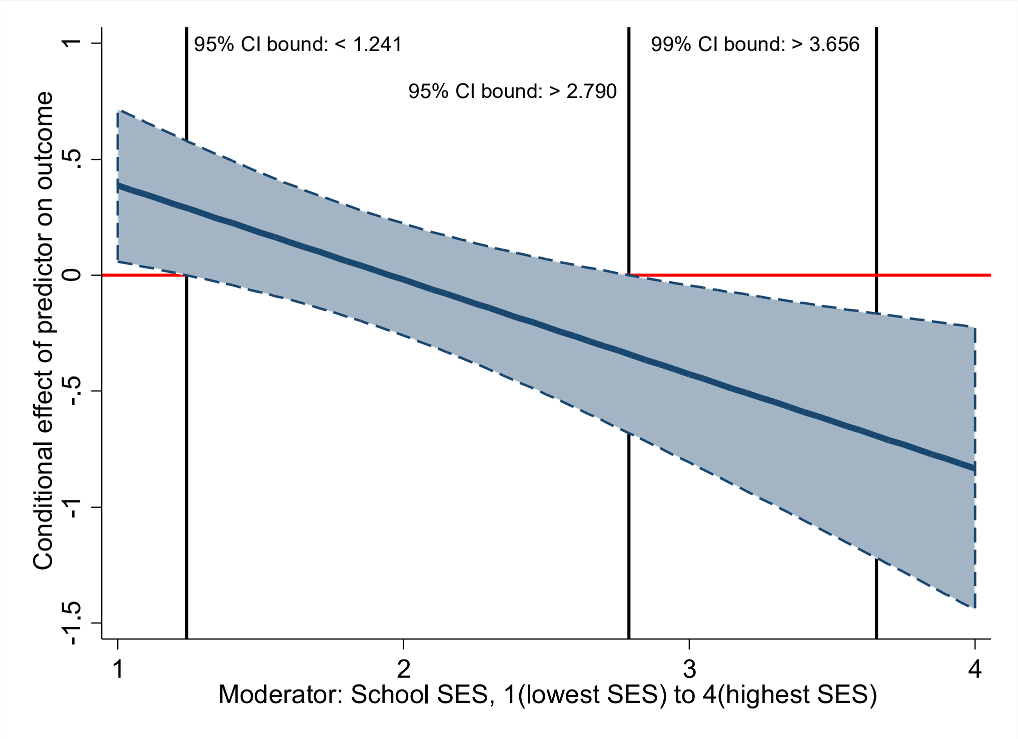

Figure S4.61. The conditional effects of peer influence from average school class responses for DN1.1 at follow-up (predictor) on focal participants' values of DN1.1 at follow-up (outcome) by school socio-economic status in all schools (moderator) with 95% CI limits for conditional effects, and bounds indicating regions of significance at the 95% and 99% levels (indicating values of the moderator for which conditional effects differ significantly from 0).

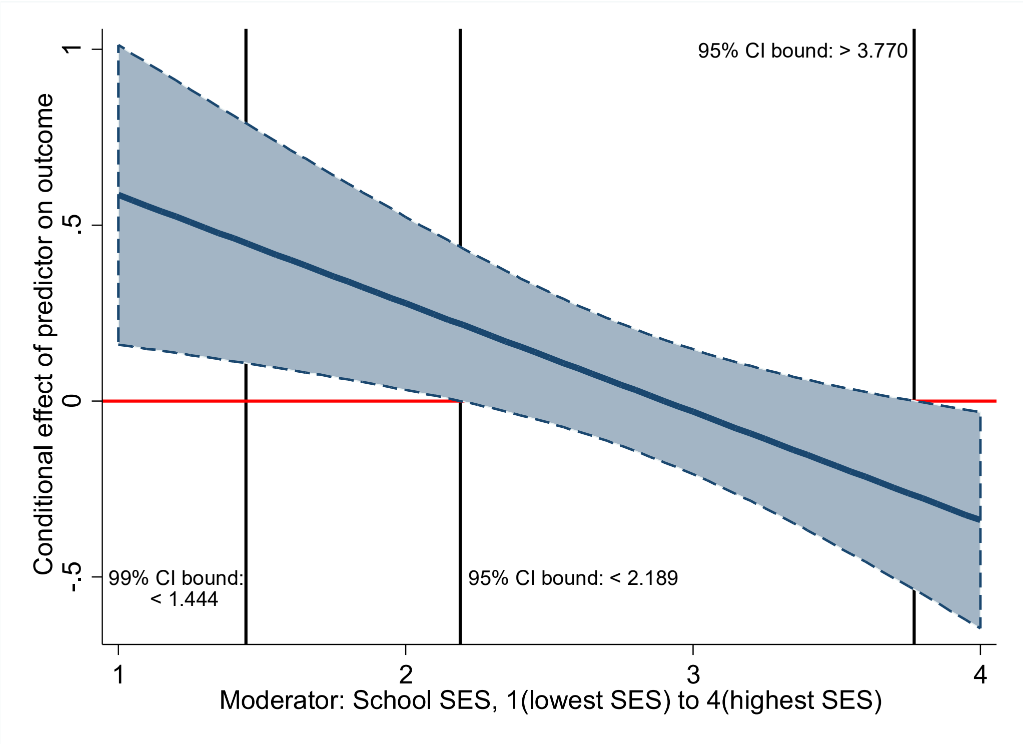

Figure S4.62. The conditional effects of peer influence from average school class responses for DN1.5 at baseline (predictor) on focal participants' values of DN1.5 at follow-up (outcome) by school socio-economic status in all schools (moderator) with 95% CI limits for conditional effects, and bounds indicating regions of significance at the 95% and 99% levels (indicating values of the moderator for which conditional effects differ significantly from 0).

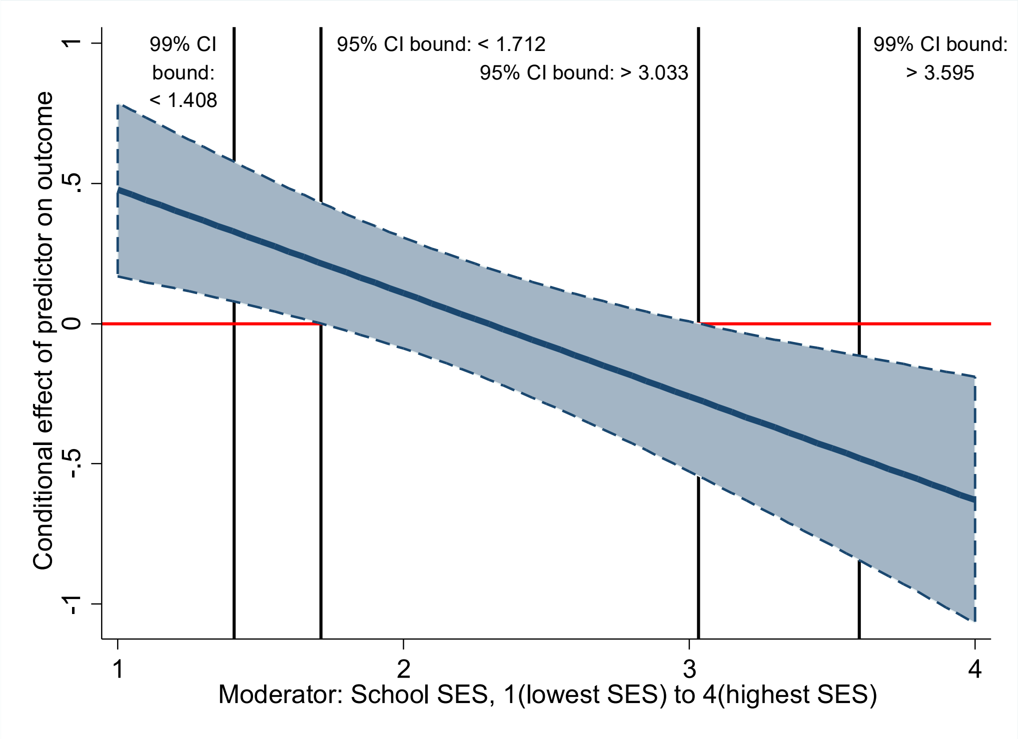

Figure S4.63. The conditional effects of peer influence from average school class responses for DN1.5 at follow-up (predictor) on focal participants' values of DN1.5 at follow-up (outcome) by school socio-economic status in all schools (moderator) with 95% CI limits for conditional effects, and bounds indicating regions of significance at the 95% and 99% levels (indicating values of the moderator for which conditional effects differ significantly from 0).

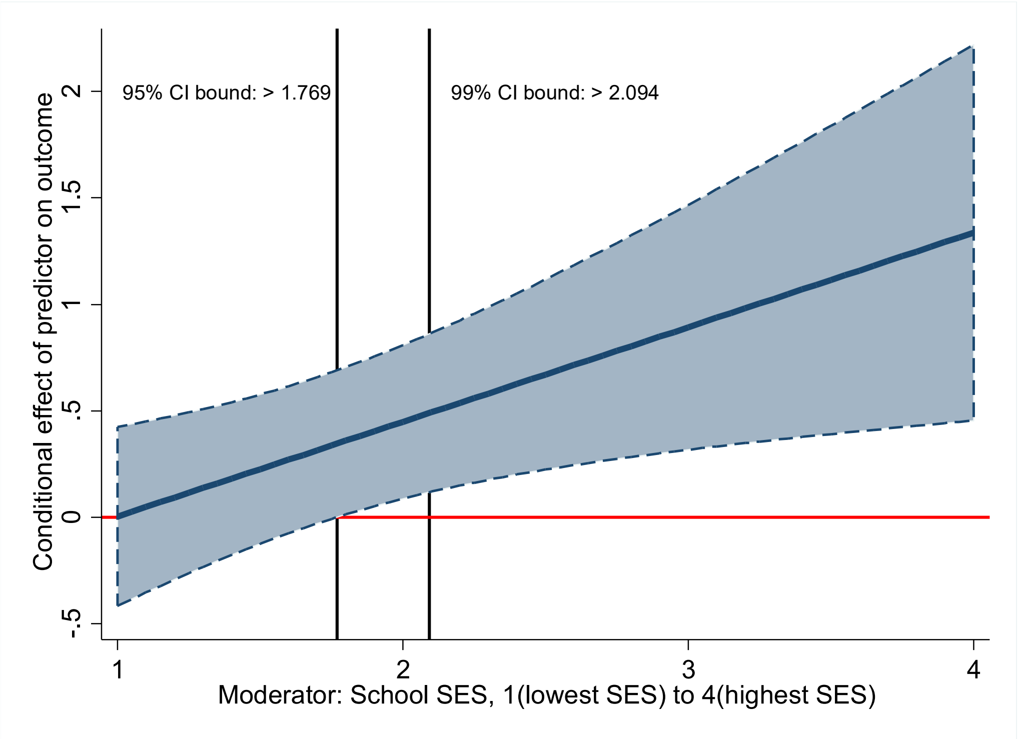

Figure S4.64. The conditional effects of peer influence from average school year group responses for self-efficacy (friends) at baseline (predictor) on focal participants' values of self-efficacy (friends) at follow-up (outcome) by school socio-economic status in all schools (moderator) with 95% CI limits for conditional effects, and bounds indicating regions of significance at the 95% and 99% levels (indicating values of the moderator for which conditional effects differ significantly from 0).

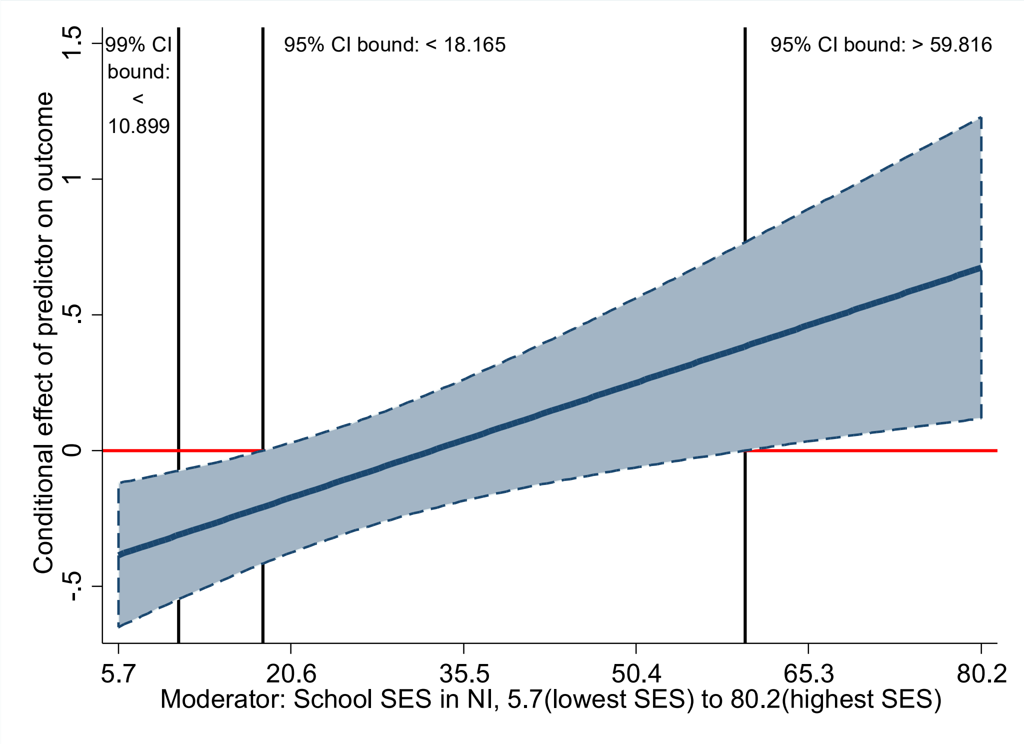

Figure S4.65. The conditional effects of peer influence from average friends' responses for IN1 at baseline (predictor) on focal participants' values of IN1 at follow-up (outcome) by school socio-economic status in NI schools (moderator) with 95% CI limits for conditional effects, and bounds indicating regions of significance at the 95% and 99% levels (indicating values of the moderator for which conditional effects differ significantly from 0).

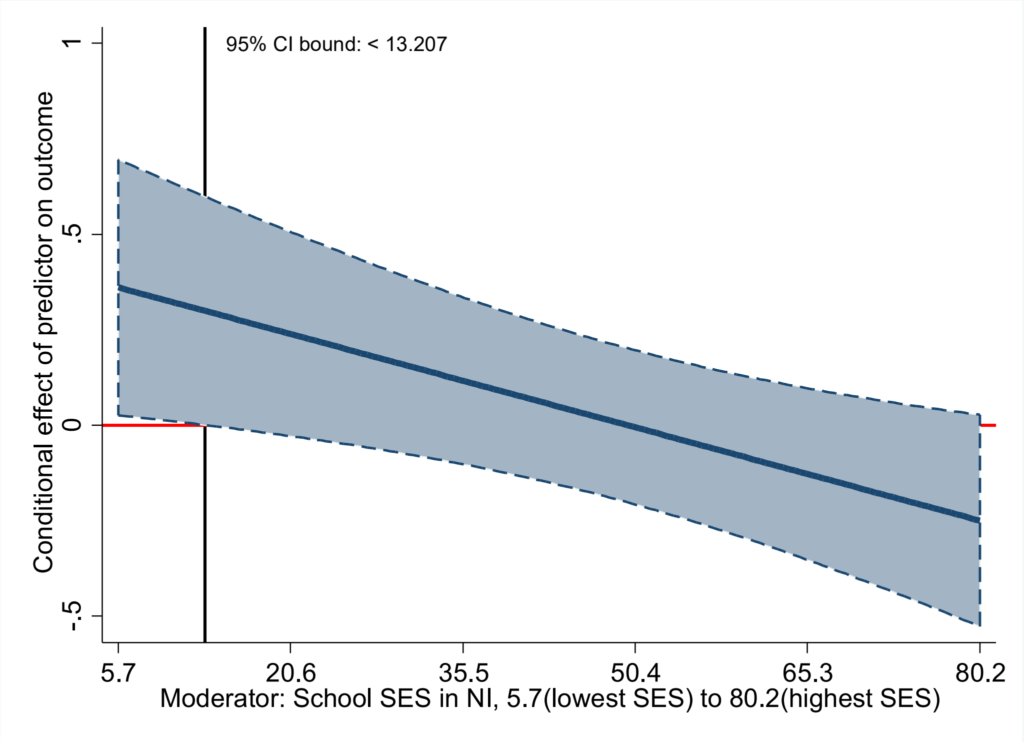

Figure S4.66. The conditional effects of peer influence from average friends' responses for IN5 at baseline (predictor) on focal participants' values of IN5 at follow-up (outcome) by school socio-economic status in NI schools (moderator) with 95% CI limits for conditional effects, and bounds indicating regions of significance at the 95% and 99% levels (indicating values of the moderator for which conditional effects differ significantly from 0).

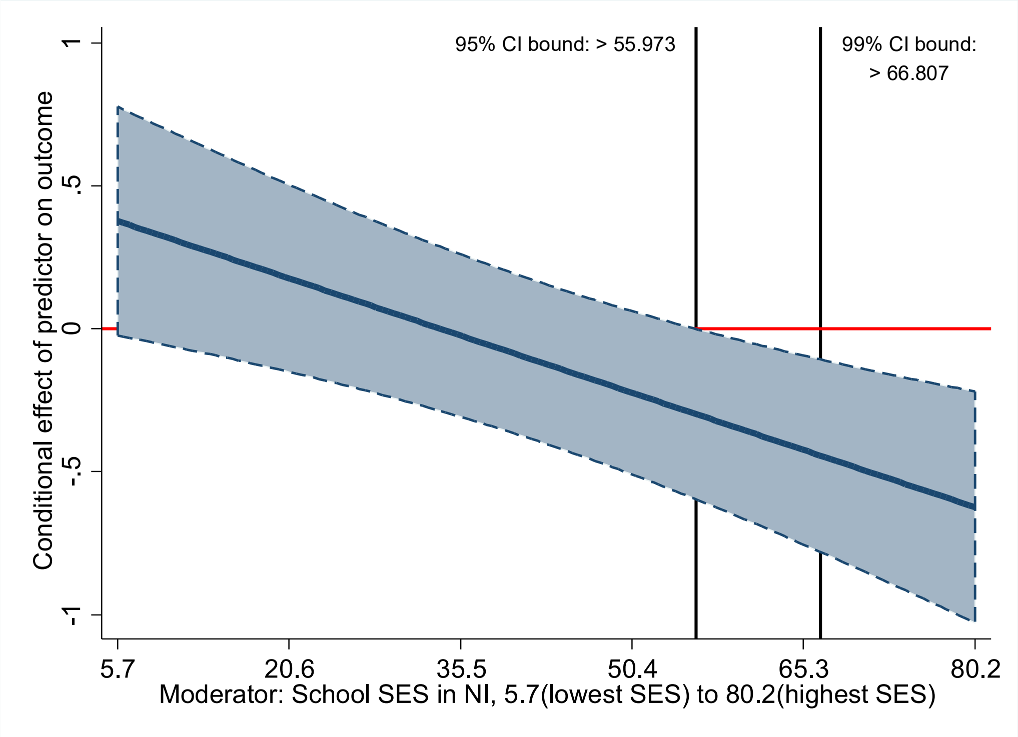

Figure S4.67. The conditional effects of peer influence from average friends' responses for DN1.1 at baseline (predictor) on focal participants' values of DN1.1 at follow-up (outcome) by school socio-economic status in NI schools (moderator) with 95% CI limits for conditional effects, and bounds indicating regions of significance at the 95% and 99% levels (indicating values of the moderator for which conditional effects differ significantly from 0).

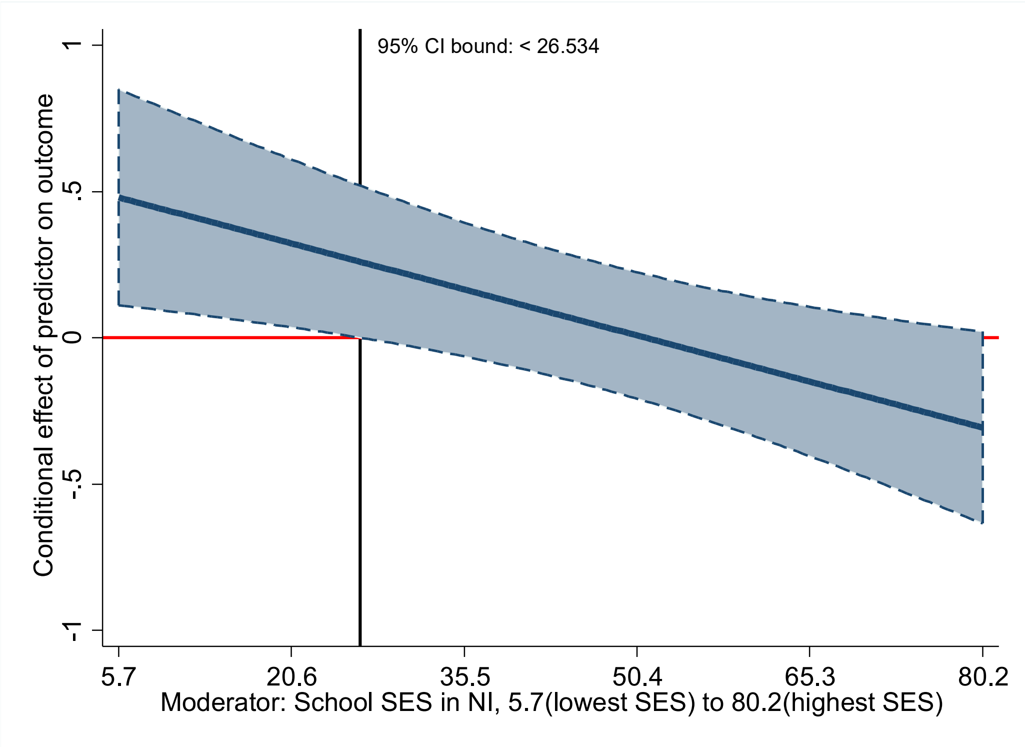

Figure S4.68. The conditional effects of peer influence from average friends' self-report smoking behavior at follow-up (predictor) on focal participants' self-report smoking behavior at follow-up (outcome) by school socio-economic status in NI schools (moderator) with 95% CI limits for conditional effects, and bounds indicating regions of significance at the 95% and 99% levels (indicating values of the moderator for which conditional effects differ significantly from 0).

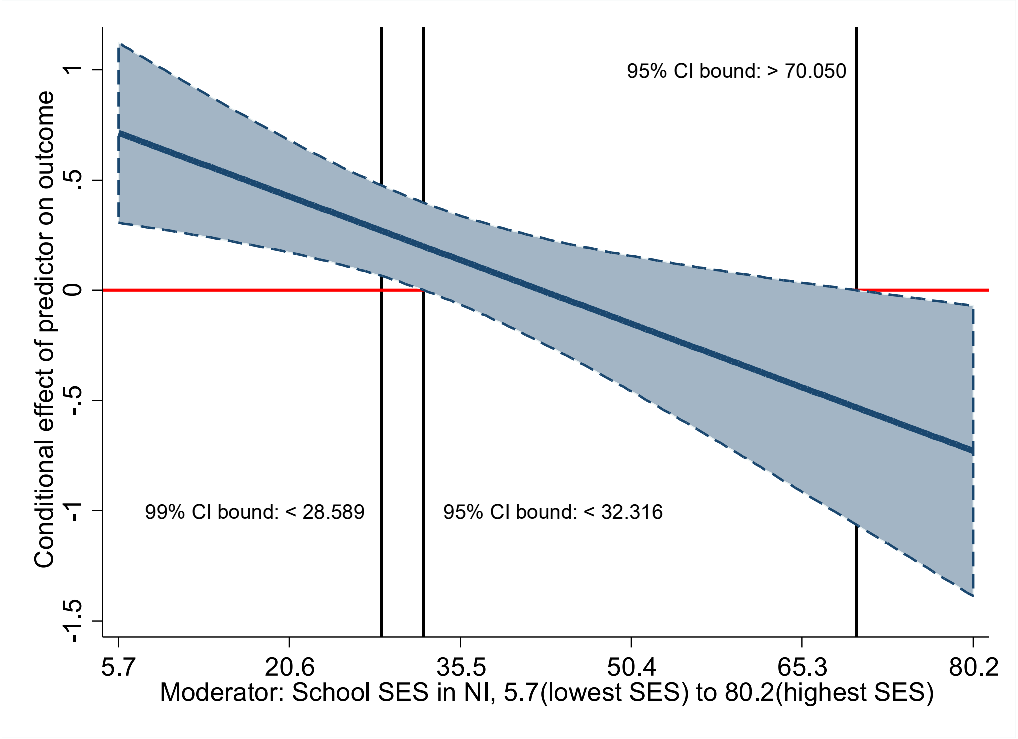

Figure S4.69. The conditional effects of peer influence from average friends' responses for self-efficacy (emotional) at follow-up (predictor) on focal participants' values of self-efficacy (emotional) at follow-up (outcome) by school socio-economic status in NI schools (moderator) with 95% CI limits for conditional effects, and bounds indicating regions of significance at the 95% and 99% levels (indicating values of the moderator for which conditional effects differ significantly from 0).

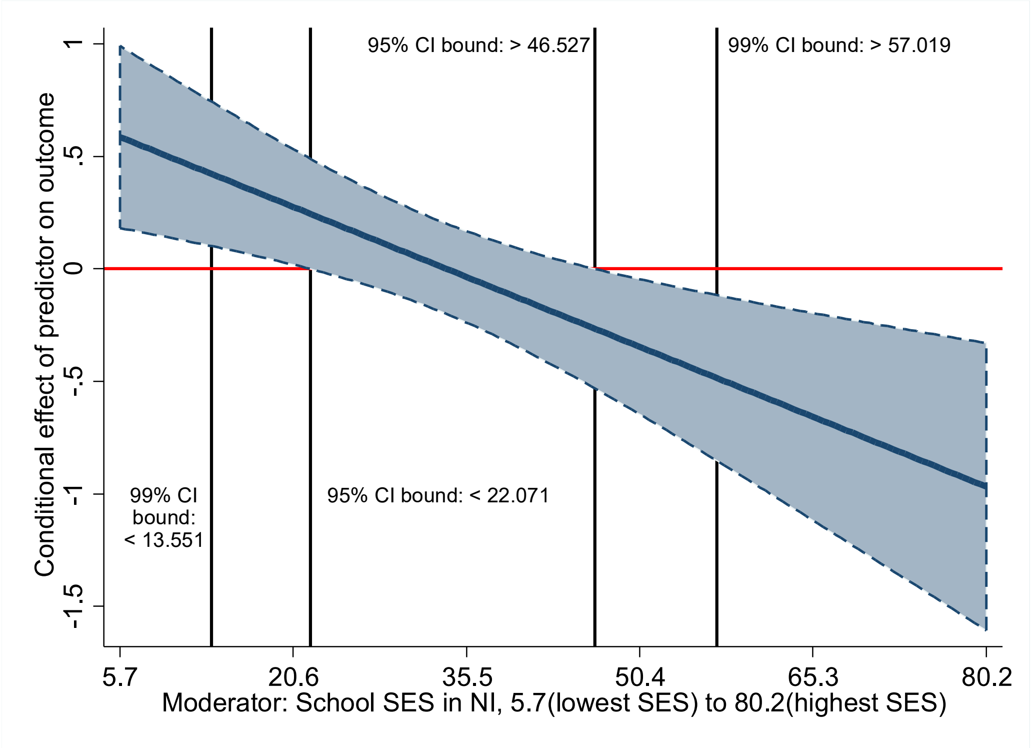

Figure S4.70. The conditional effects of peer influence from average friends' responses for self-efficacy (friends) at follow-up (predictor) on focal participants' values of self-efficacy (friends) at follow-up (outcome) by school socio-economic status in NI schools (moderator) with 95% CI limits for conditional effects, and bounds indicating regions of significance at the 95% and 99% levels (indicating values of the moderator for which conditional effects differ significantly from 0).

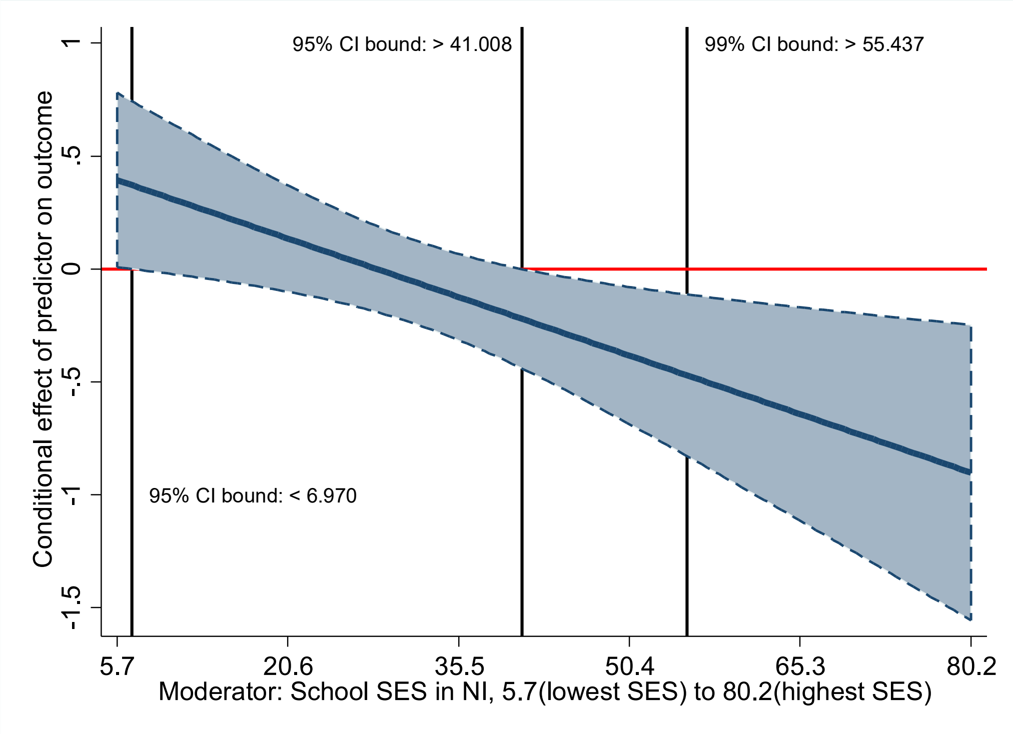

Figure S4.71. The conditional effects of peer influence from average friends' responses for self-efficacy (opportunity) at follow-up (predictor) on focal participants' values of self-efficacy (opportunity) at follow-up (outcome) by school socio-economic status in NI schools (moderator) with 95% CI limits for conditional effects, and bounds indicating regions of significance at the 95% and 99% levels (indicating values of the moderator for which conditional effects differ significantly from 0).

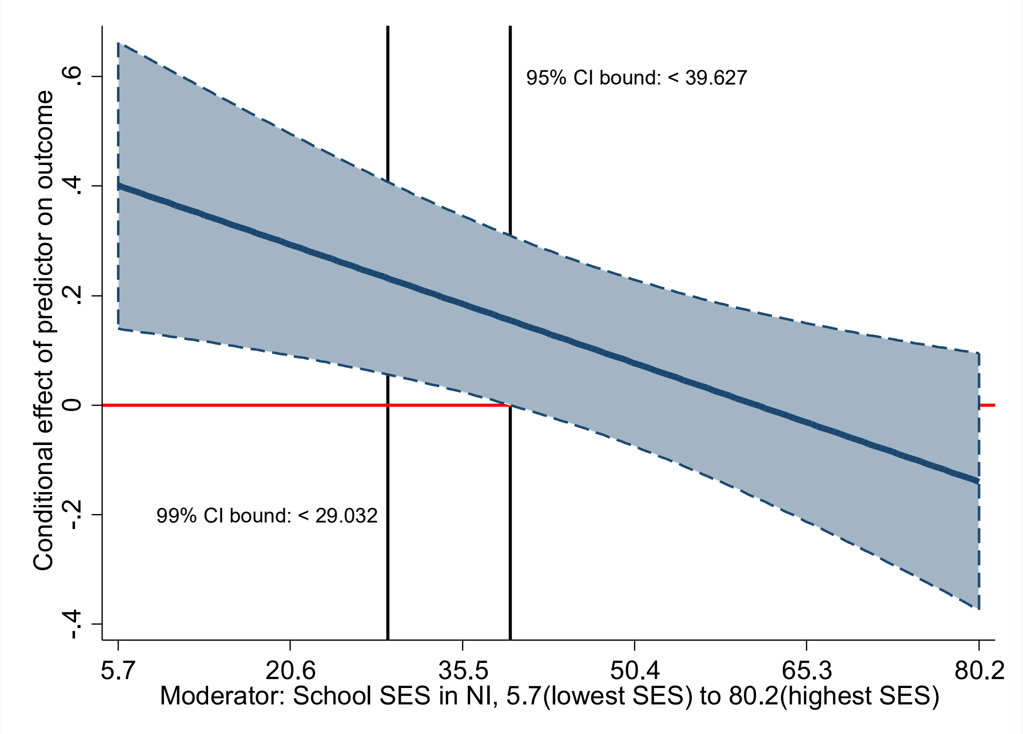

Figure S4.72. The conditional effects of peer influence from average friends' perceived physical risks at baseline (predictor) on focal participants' perceived physical risks at follow-up (outcome) by school socio-economic status in NI schools (moderator) with 95% CI limits for conditional effects, and bounds indicating regions of significance at the 95% and 99% levels (indicating values of the moderator for which conditional effects differ significantly from 0).

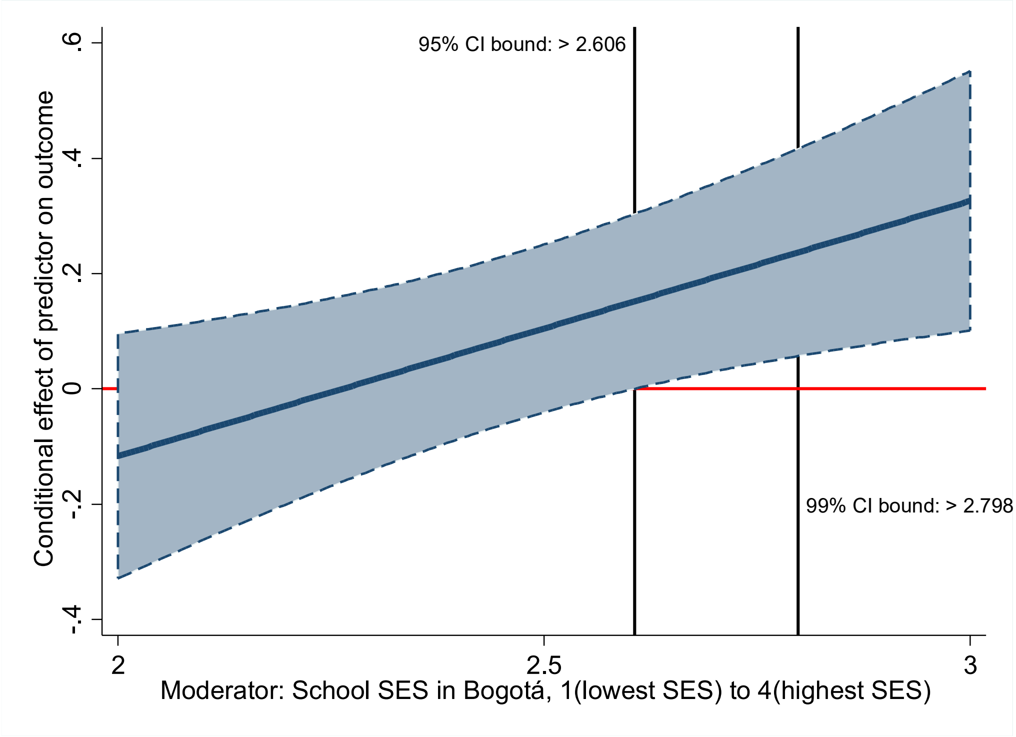

Figure S4.73. The conditional effects of peer influence from average friends' responses for IN1 at follow-up (predictor) on focal participants' values of IN1 at follow-up (outcome) by school socio-economic status in Bogotá schools (moderator) with 95% CI limits for conditional effects, and bounds indicating regions of significance at the 95% and 99% levels (indicating values of the moderator for which conditional effects differ significantly from 0).

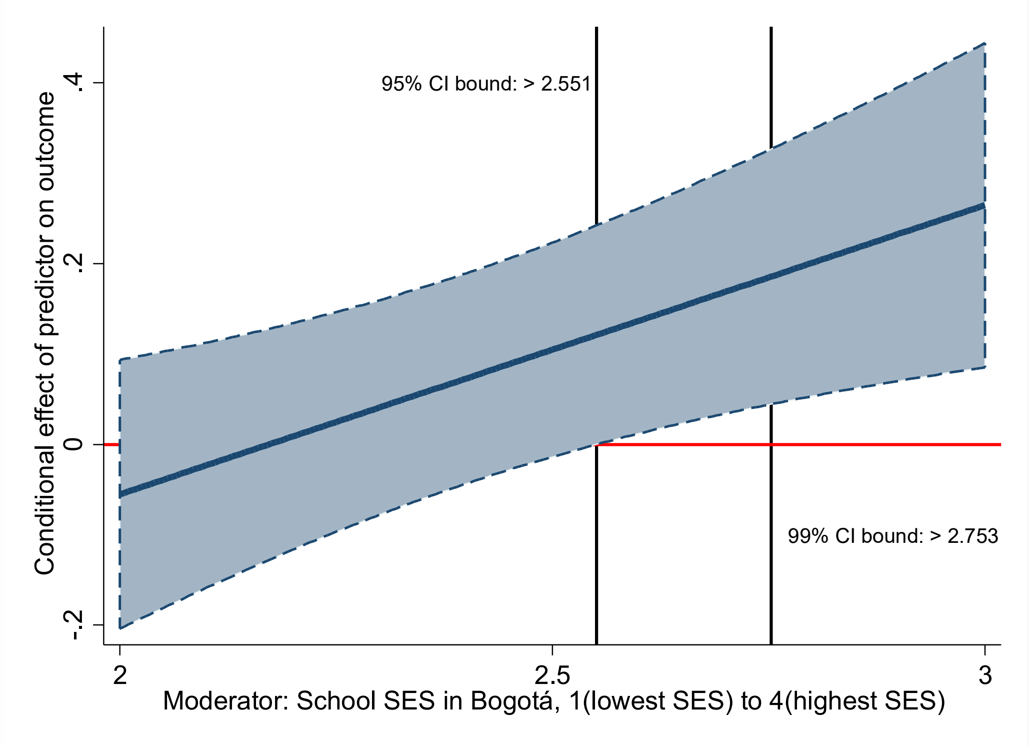

Figure S4.74. The conditional effects of peer influence from average friends' self-report descriptive norms scale 1 (average DN1.1 to DN1.5) at baseline (predictor) on focal participants' self-report descriptive norms scale 1 at follow-up (outcome) by school socio-economic status in Bogotá schools (moderator) with 95% CI limits for conditional effects, and bounds indicating regions of significance at the 95% and 99% levels (indicating values of the moderator for which conditional effects differ significantly from 0).

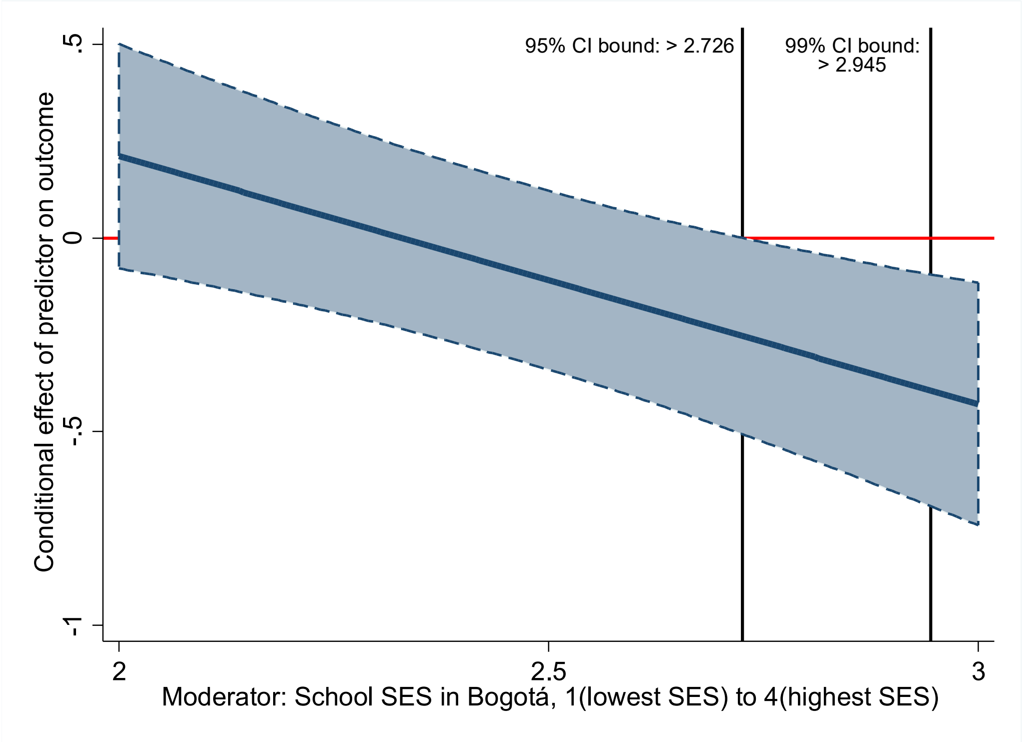

Figure S4.75. The conditional effects of peer influence from average friends' objectively measured smoking behavior at baseline (predictor) on focal participants' objectively measured smoking behavior at follow-up (outcome) by school socio-economic status in Bogotá schools (moderator) with 95% CI limits for conditional effects, and bounds indicating regions of significance at the 95% and 99% levels (indicating values of the moderator for which conditional effects differ significantly from 0).

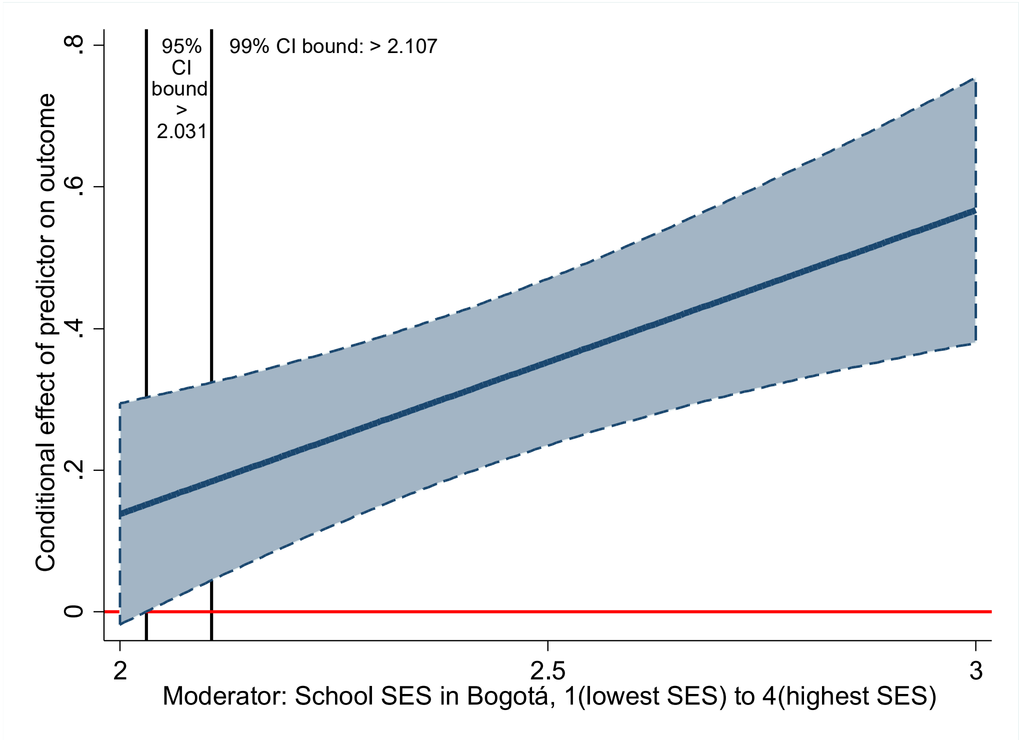

Figure S4.76. The conditional effects of peer influence from average friends' objectively measured smoking behavior at follow-up (predictor) on focal participants' objectively measured smoking behavior at follow-up (outcome) by school socio-economic status in Bogotá schools (moderator) with 95% CI limits for conditional effects, and bounds indicating regions of significance at the 95% and 99% levels (indicating values of the moderator for which conditional effects differ significantly from 0).

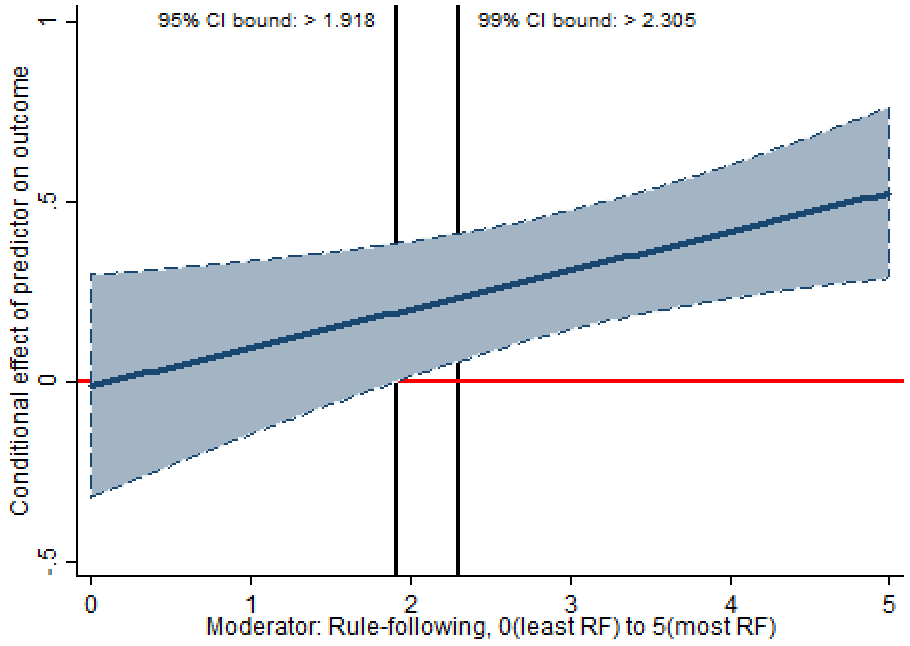

Figure S4.77. The conditional effects of peer influence from average school class responses for P2S8 at baseline (predictor) on focal participants' values of P2S8 at follow-up (outcome) by rule-following (moderator) with 95% CI limits for conditional effects, and bounds indicating regions of significance at the 95% and 99% levels (indicating values of the moderator for which conditional effects differ significantly from 0).

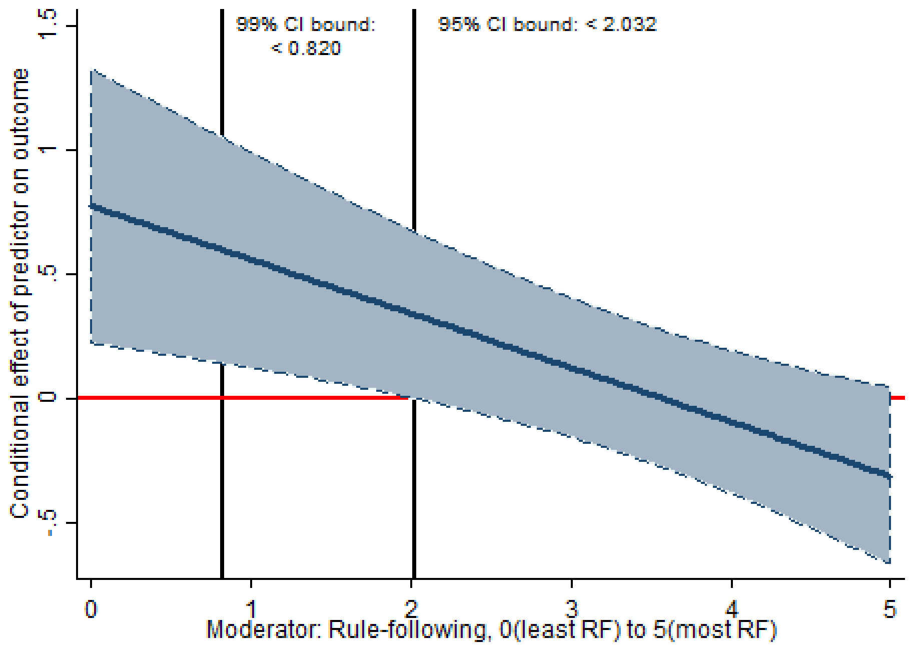

Figure S4.78. The conditional effects of peer influence from average school year group perceived physical risks at baseline (predictor) on focal participants' perceived physical risks at follow-up (outcome) by rule-following (moderator) with 95% CI limits for conditional effects, and bounds indicating regions of significance at the 95% and 99% levels (indicating values of the moderator for which conditional effects differ significantly from 0).

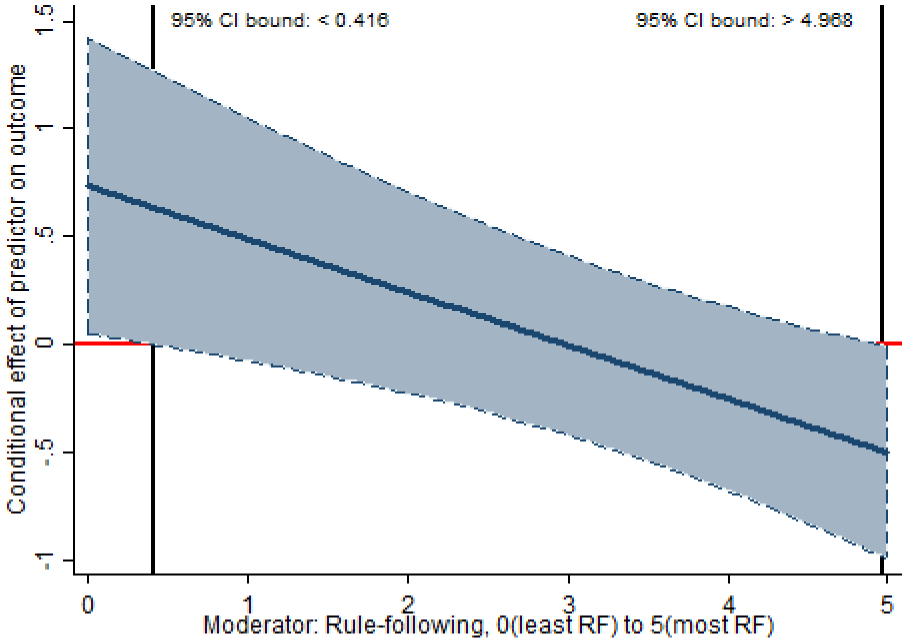

Figure S4.79. The conditional effects of peer influence from average school year group perceived physical risks at follow-up (predictor) on focal participants' perceived physical risks at follow-up (outcome) by rule-following (moderator) with 95% CI limits for conditional effects, and bounds indicating regions of significance at the 95% level (indicating values of the moderator for which conditional effects differ significantly from 0).

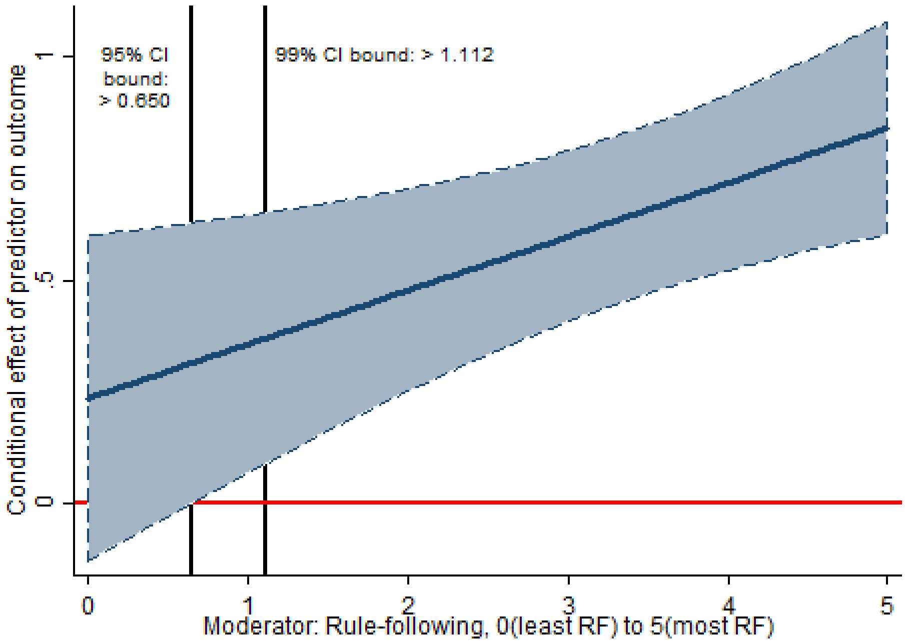

Figure S4.80. The conditional effects of peer influence from average school year group perceived behavioral control (easy to quit) at follow-up (predictor) on focal participants' perceived behavioral control (easy to quit) at follow-up (outcome) by rule-following (moderator) with 95% CI limits for conditional effects, and bounds indicating regions of significance at the 95% and 99% levels (indicating values of the moderator for which conditional effects differ significantly from 0).

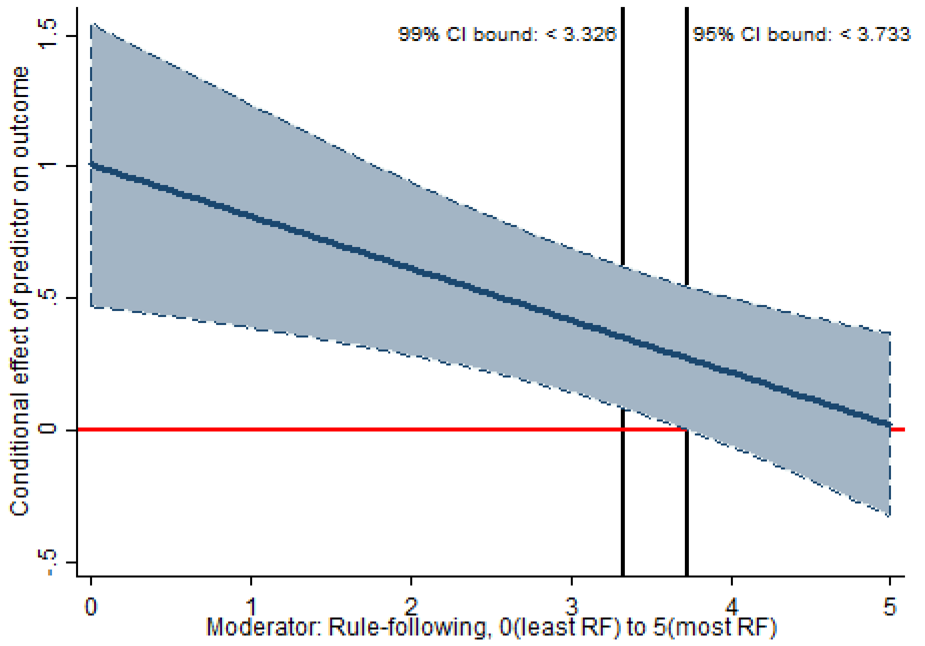

Figure S4.81. The conditional effects of peer influence from average school class perceived behavioral control (easy to avoid) at baseline (predictor) on focal participants' perceived behavioral control (easy to avoid) at follow-up (outcome) by rule-following (moderator) with 95% CI limits for conditional effects, and bounds indicating regions of significance at the 95% and 99% levels (indicating values of the moderator for which conditional effects differ significantly from 0).

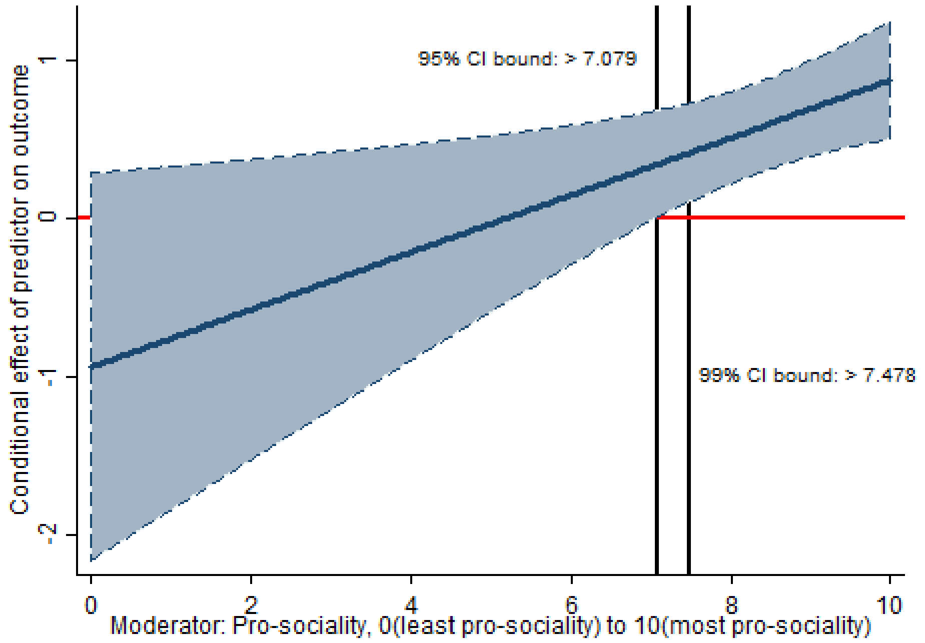

Figure S4.82. The conditional effects of peer influence from average school year group responses for P2S6 at follow-up (predictor) on focal participants' values of P2S6 at follow-up (outcome) by pro-sociality (moderator) with 95% CI limits for conditional effects, and bounds indicating regions of significance at the 95% and 99% levels (indicating values of the moderator for which conditional effects differ significantly from 0).

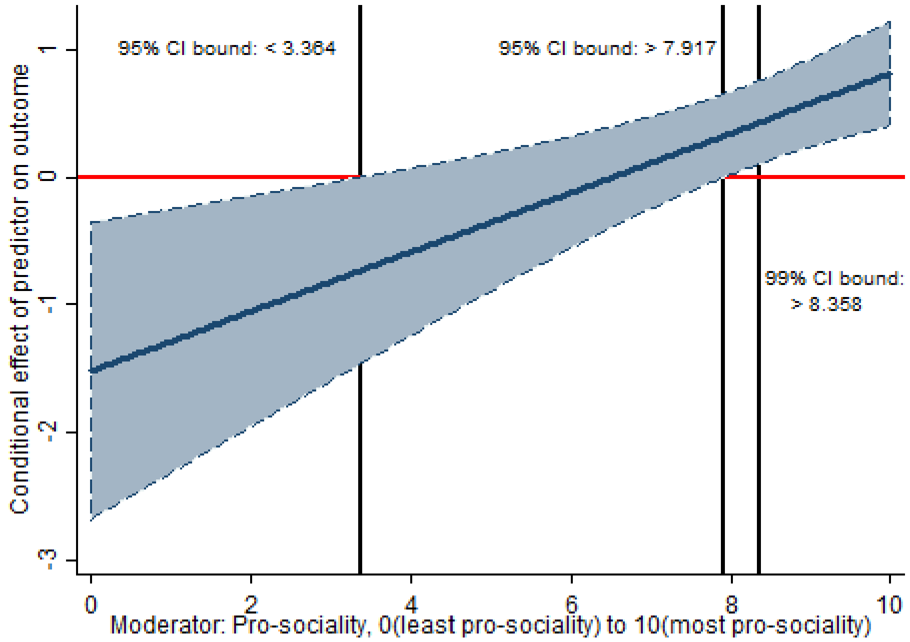

Figure S4.83. The conditional effects of peer influence from average school year group responses for P2S7 at follow-up (predictor) on focal participants' values of P2S7 at follow-up (outcome) by pro-sociality (moderator) with 95% CI limits for conditional effects, and bounds indicating regions of significance at the 95% and 99% levels (indicating values of the moderator for which conditional effects differ significantly from 0).

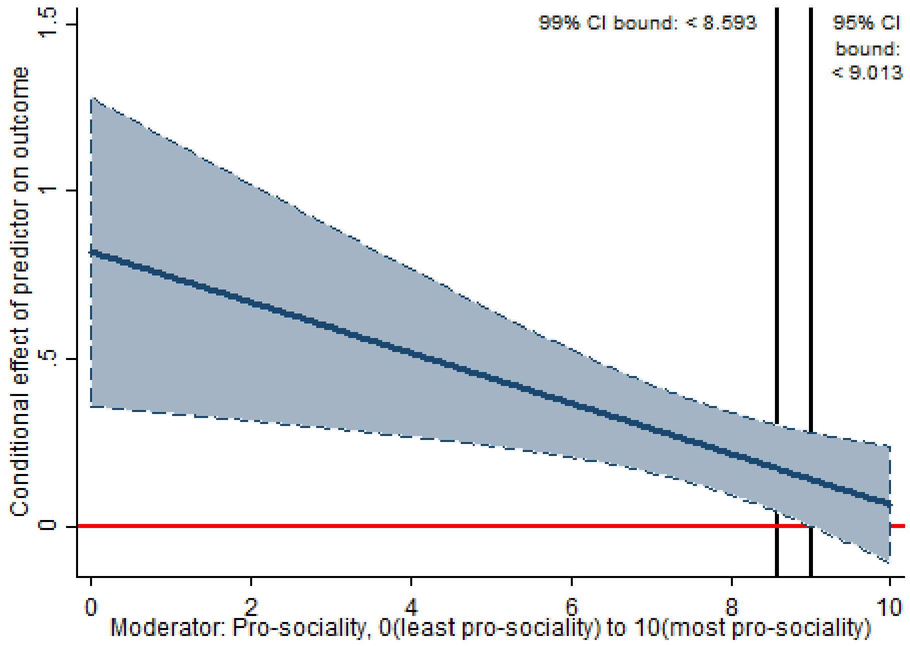

Figure S4.84. The conditional effects of peer influence from average friends' responses for P2S9 at follow-up (predictor) on focal participants' values of P2S9 at follow-up (outcome) by pro-sociality (moderator) with 95% CI limits for conditional effects, and bounds indicating regions of significance at the 95% and 99% levels (indicating values of the moderator for which conditional effects differ significantly from 0).

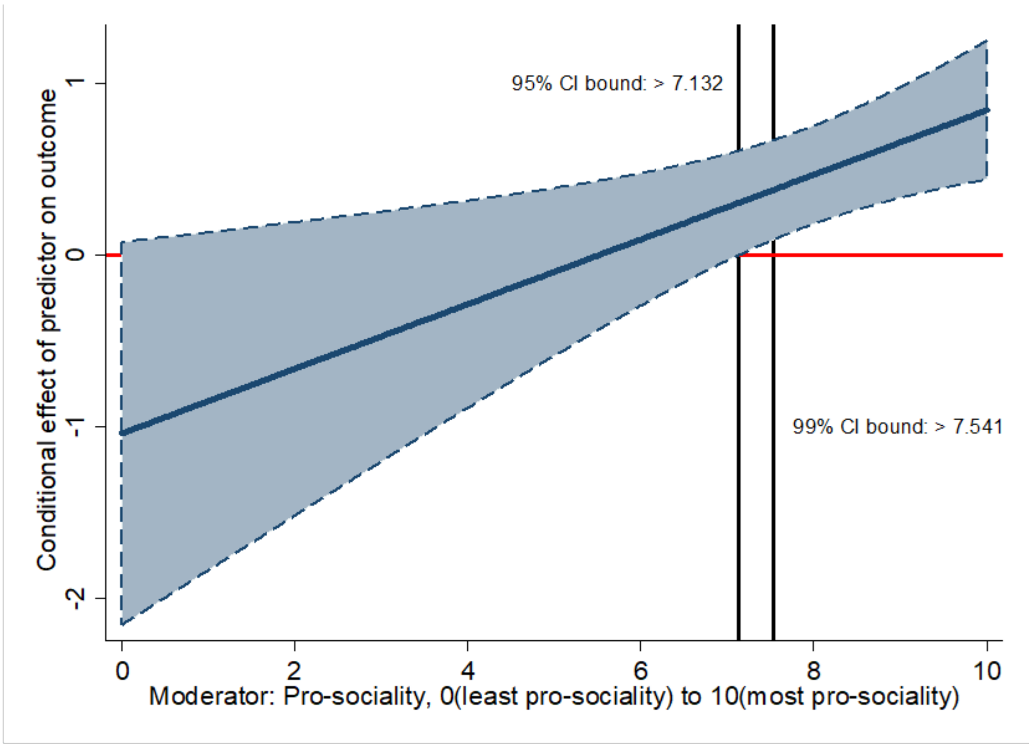

Figure S4.85. The conditional effects of peer influence from average school year group responses for experiment part 2 (injunctive norms, average P2S2 to P2S9) at baseline (predictor) on focal participants' values of experiment part 2 at follow-up (outcome) by pro-sociality (moderator) with 95% CI limits for conditional effects, and bounds indicating regions of significance at the 95% and 99% levels (indicating values of the moderator for which conditional effects differ significantly from 0).

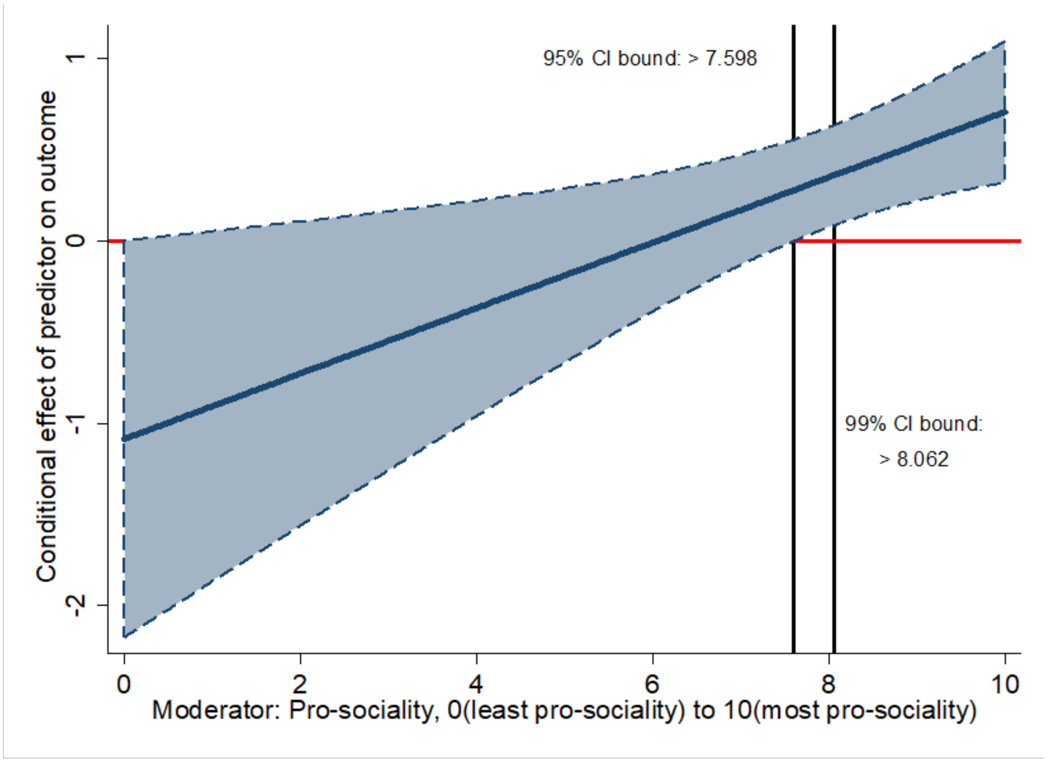

Figure S4.86. The conditional effects of peer influence from average school year group responses for experiment part 2 (injunctive norms, average P2S2 to P2S9) at follow-up (predictor) on focal participants' values of experiment part 2 at follow-up (outcome) by pro-sociality (moderator) with 95% CI limits for conditional effects, and bounds indicating regions of significance at the 95% and 99% levels (indicating values of the moderator for which conditional effects differ significantly from 0).

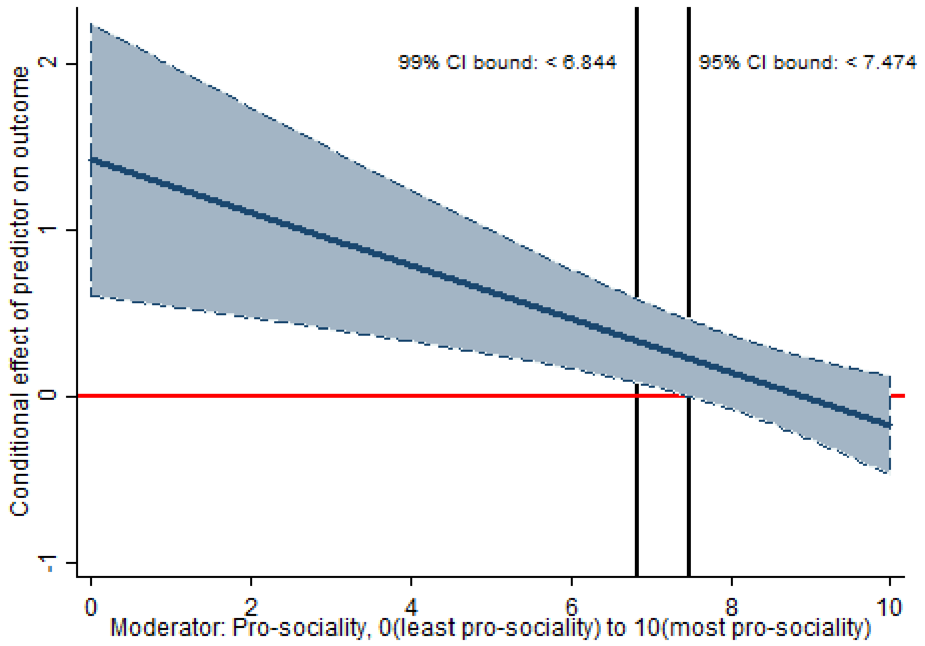

Figure S4.87. The conditional effects of peer influence from average school class responses to IN4 at follow-up (predictor) on focal participants' values of IN4 at follow-up (outcome) by pro-sociality (moderator) with 95% CI limits for conditional effects, and bounds indicating regions of significance at the 95% and 99% levels (indicating values of the moderator for which conditional effects differ significantly from 0).

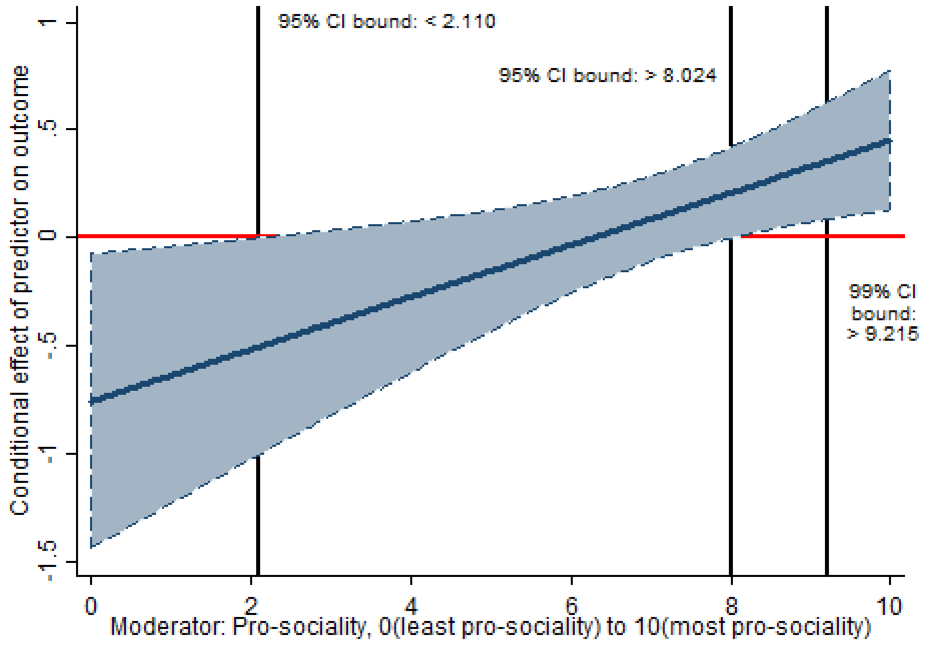

Figure S4.88. The conditional effects of peer influence from average school class responses to DN2.1 at baseline (predictor) on focal participants' values of DN2.1 at follow-up (outcome) by pro-sociality (moderator) with 95% CI limits for conditional effects, and bounds indicating regions of significance at the 95% and 99% levels (indicating values of the moderator for which conditional effects differ significantly from 0).

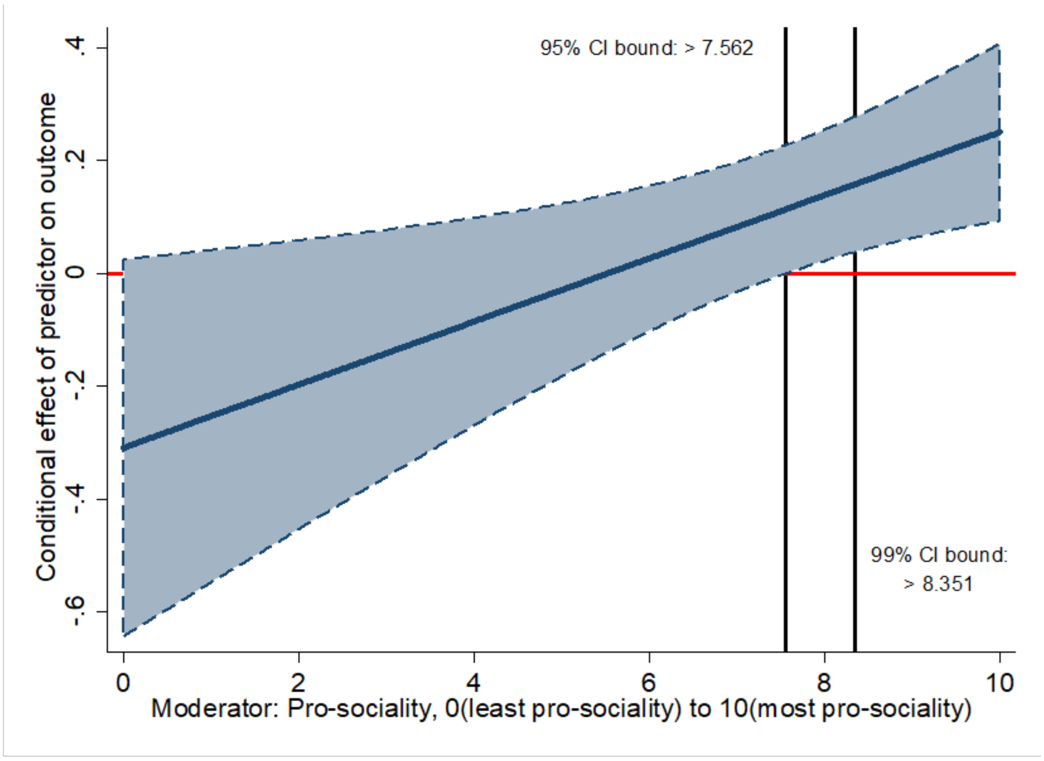

Figure S4.89. The conditional effects of peer influence from average friends' responses to self-report descriptive norms scale 2 (average DN2.1 to DN2.3) at follow-up (predictor) on focal participants' values of descriptive norms scale 2 at follow-up (outcome) by pro-sociality (moderator) with 95% CI limits for conditional effects, and bounds indicating regions of significance at the 95% and 99% levels (indicating values of the moderator for which conditional effects differ significantly from 0).

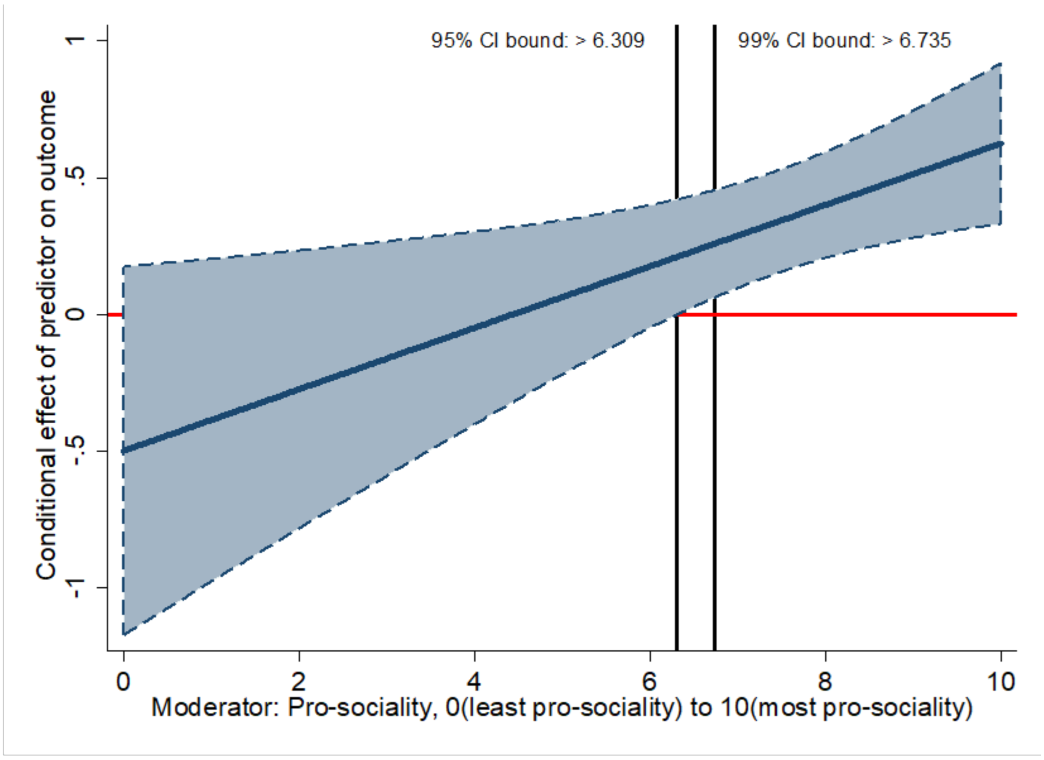

Figure S4.90. The conditional effects of peer influence from average school class responses to self-report descriptive norms scale 2 (average DN2.1 to DN2.3) at baseline (predictor) on focal participants' values of descriptive norms scale 2 at follow-up (outcome) by pro-sociality (moderator) with 95% CI limits for conditional effects, and bounds indicating regions of significance at the 95% and 99% levels (indicating values of the moderator for which conditional effects differ significantly from 0).

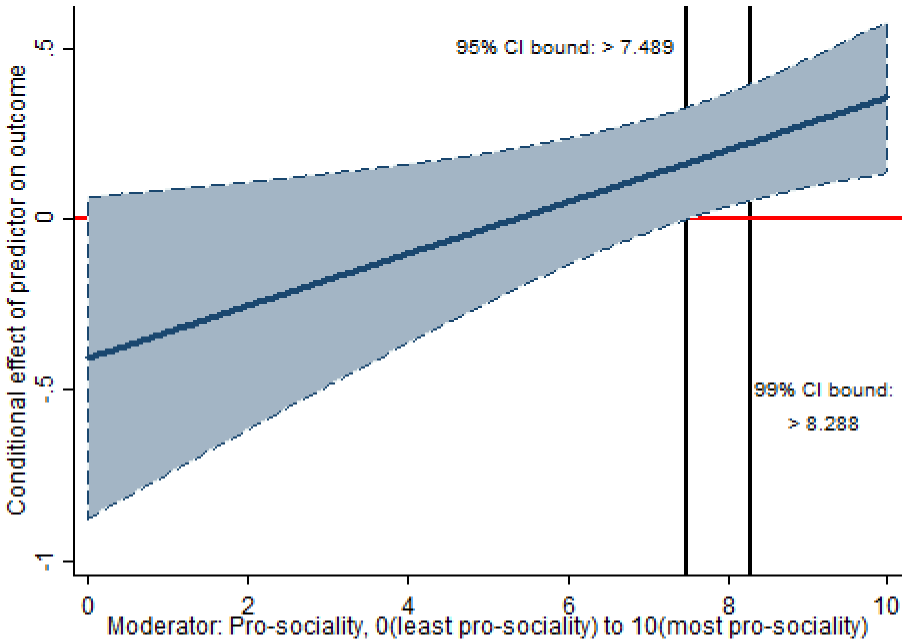

Figure S4.91. The conditional effects of peer influence from average friends' intentions at baseline (predictor) on focal participants' intentions at follow-up (outcome) by pro-sociality (moderator) with 95% CI limits for conditional effects, and bounds indicating regions of significance at the 95% and 99% levels (indicating values of the moderator for which conditional effects differ significantly from 0).

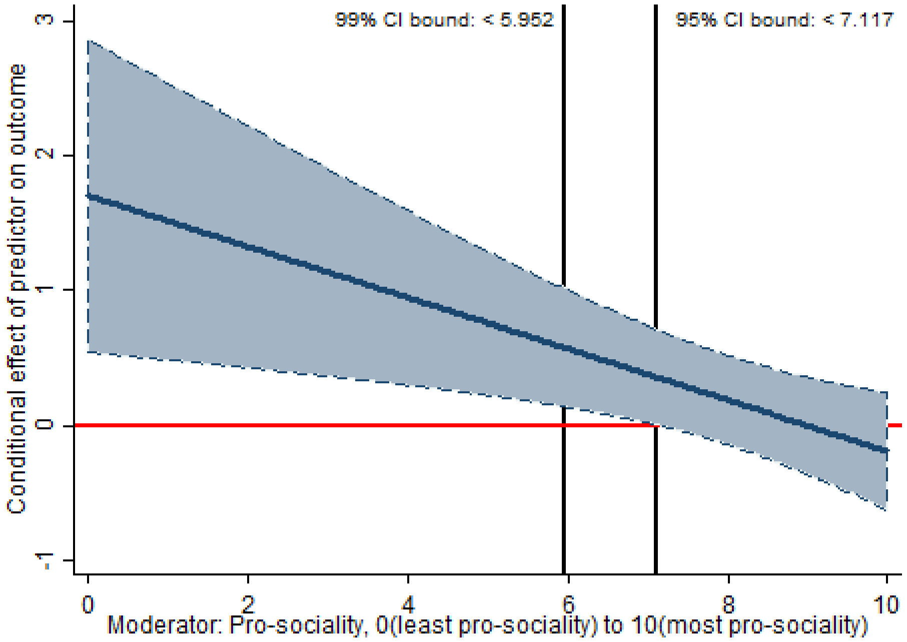

Figure S4.92. The conditional effects of peer influence from average school year group self-efficacy (opportunity) at follow-up (predictor) on focal participants' self-efficacy (opportunity) at follow-up (outcome) by pro-sociality (moderator) with 95% CI limits for conditional effects, and bounds indicating regions of significance at the 95% and 99% levels (indicating values of the moderator for which conditional effects differ significantly from 0).

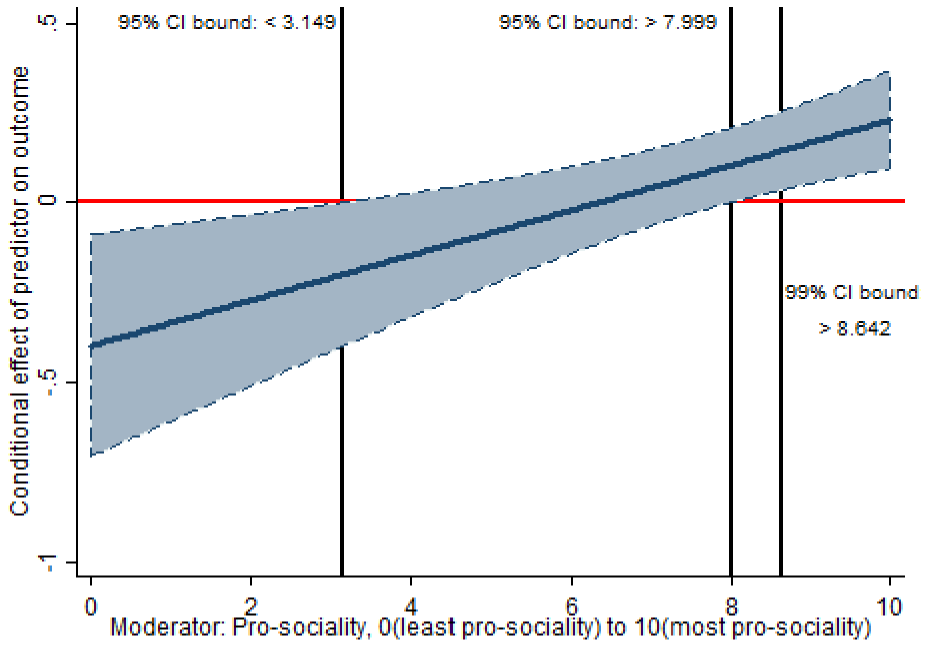

Figure S4.93. The conditional effects of peer influence from average friends' perceived physical risks at baseline (predictor) on focal participants' perceived physical risks at follow-up (outcome) by pro-sociality (moderator) with 95% CI limits for conditional effects, and bounds indicating regions of significance at the 95% and 99% levels (indicating values of the moderator for which conditional effects differ significantly from 0).

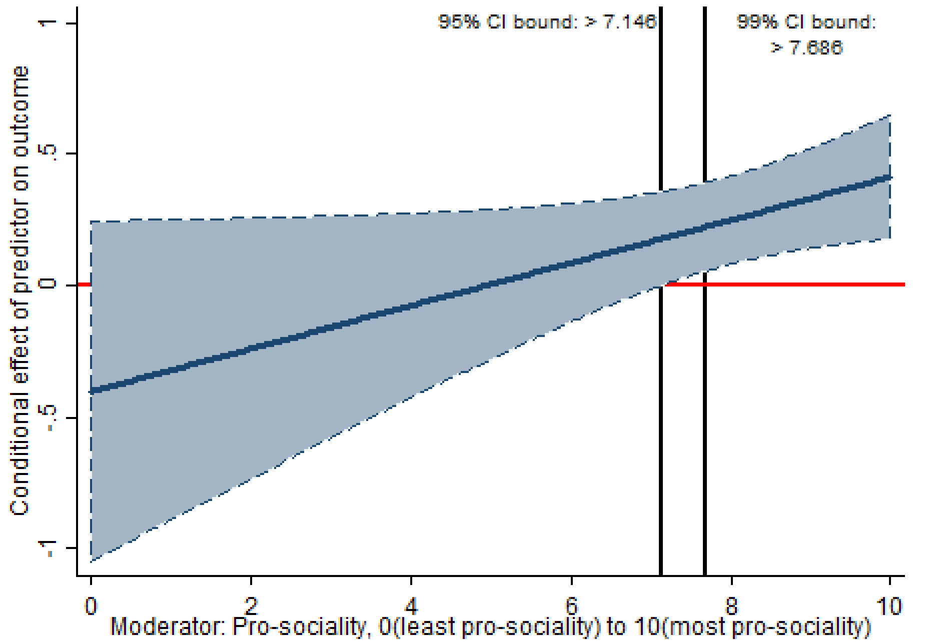

Figure S4.94. The conditional effects of peer influence from average school class perceived social risks at follow-up (predictor) on focal participants' perceived social risks at follow-up (outcome) by pro-sociality (moderator) with 95% CI limits for conditional effects, and bounds indicating regions of significance at the 95% and 99% levels (indicating values of the moderator for which conditional effects differ significantly from 0).

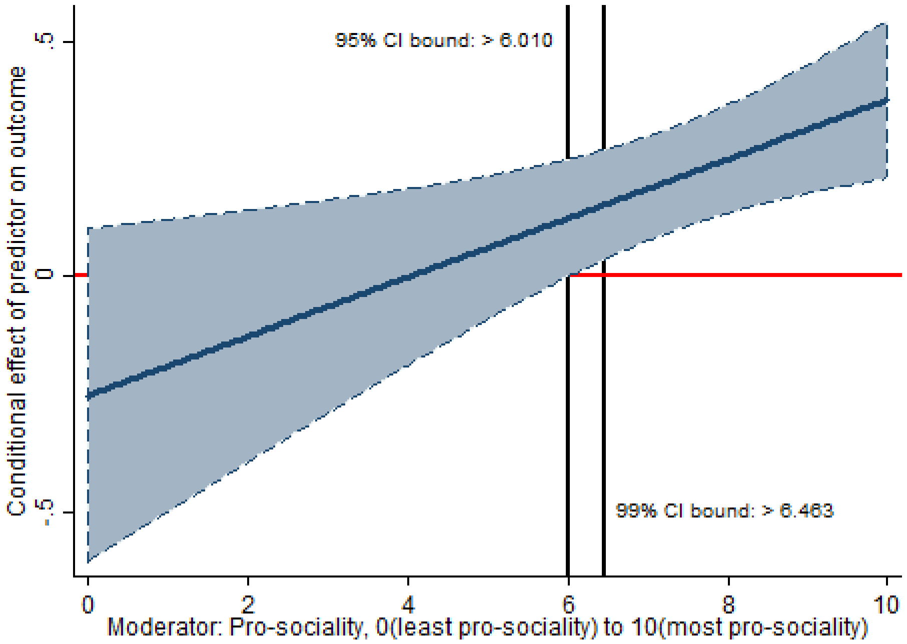

Figure S4.95. The conditional effects of peer influence from average friends' perceived addiction risks at baseline (predictor) on focal participants' perceived addiction risks at follow-up (outcome) by pro-sociality (moderator) with 95% CI limits for conditional effects, and bounds indicating regions of significance at the 95% and 99% levels (indicating values of the moderator for which conditional effects differ significantly from 0).

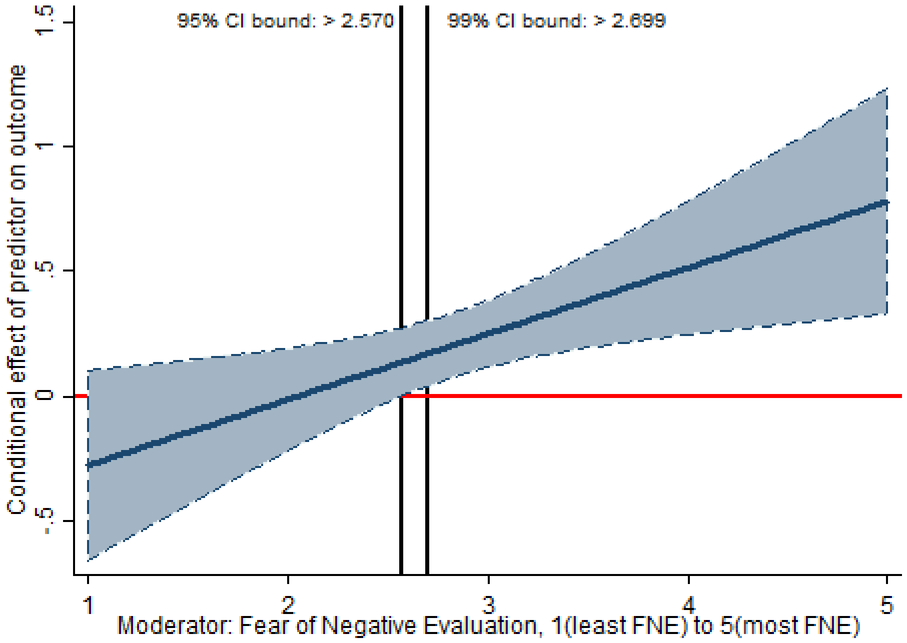

Figure S4.96. The conditional effects of peer influence from average friends' responses for P2S6 at baseline (predictor) on focal participants' values of P2S6 at follow-up (outcome) by fear of negative evaluation (moderator) with 95% CI limits for conditional effects, and bounds indicating regions of significance at the 95% and 99% levels (indicating values of the moderator for which conditional effects differ significantly from 0).

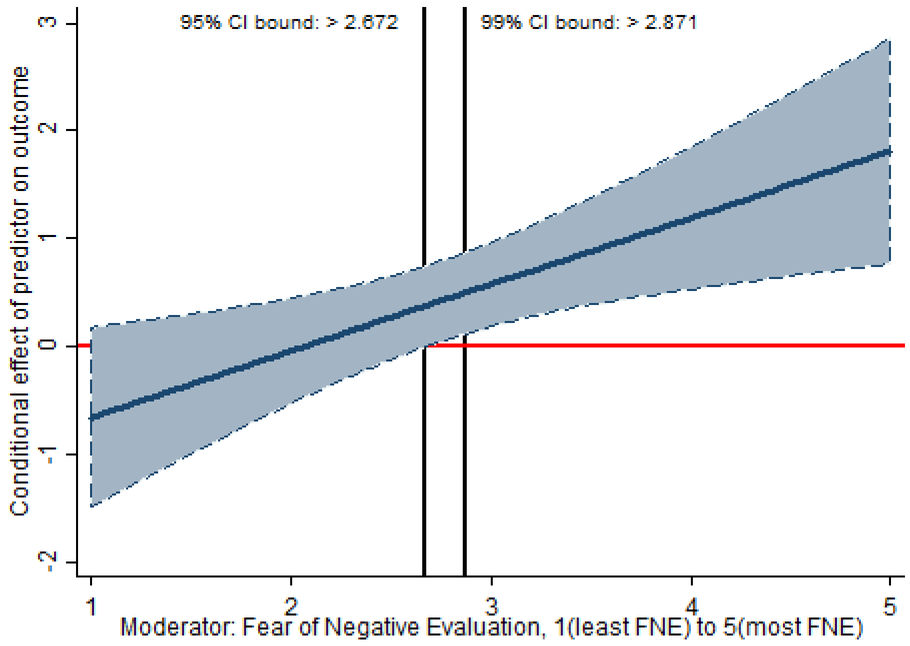

Figure S4.97. The conditional effects of peer influence from average school year group responses for P2S6 at baseline (predictor) on focal participants' values of P2S6 at follow-up (outcome) by fear of negative evaluation (moderator) with 95% CI limits for conditional effects, and bounds indicating regions of significance at the 95% and 99% levels (indicating values of the moderator for which conditional effects differ significantly from 0).

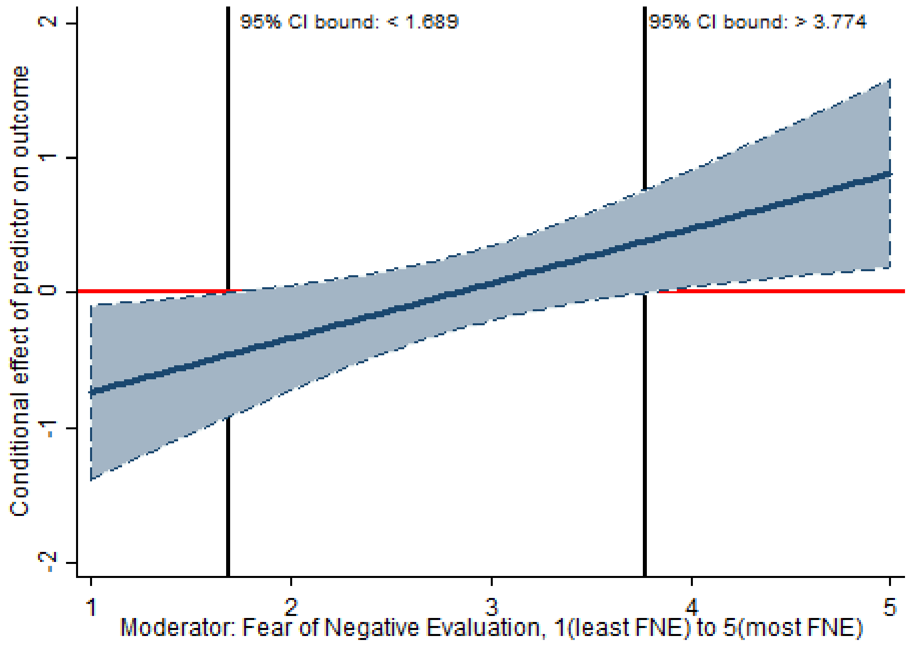

Figure S4.98. The conditional effects of peer influence from average school year group responses to DN1.3 at follow-up (predictor) on focal participants' values of DN1.3 at follow-up (outcome) by fear of negative evaluation (moderator) with 95% CI limits for conditional effects, and bounds indicating regions of significance at the 95% level (indicating values of the moderator for which conditional effects differ significantly from 0).

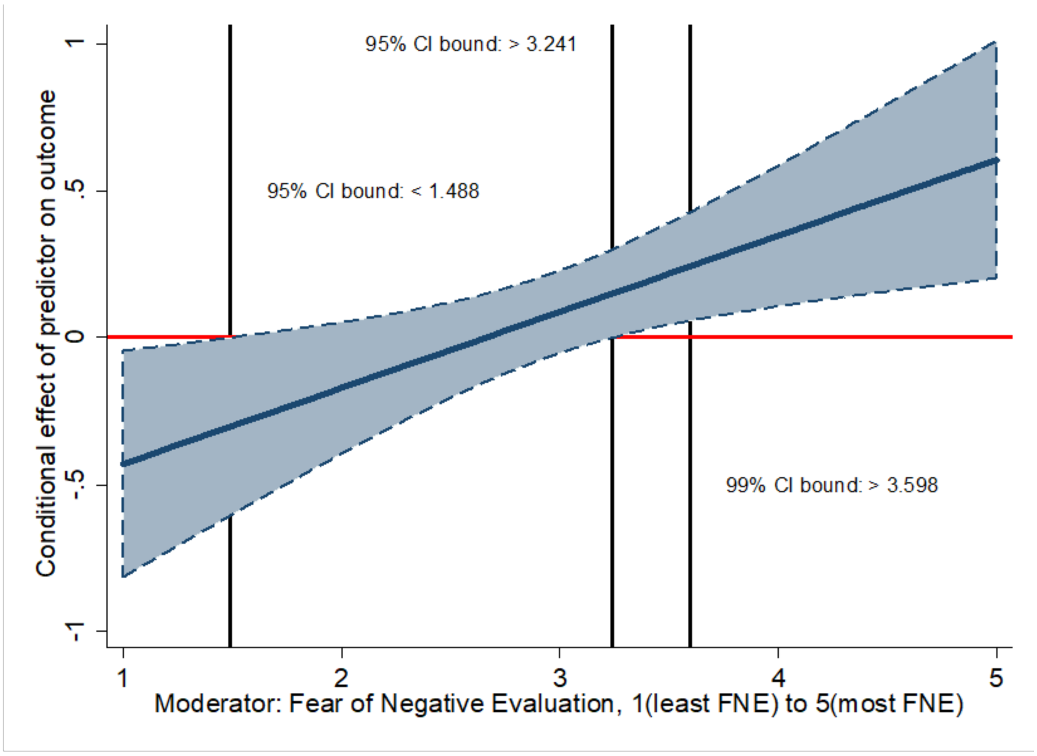

Figure S4.99. The conditional effects of peer influence from average school class responses to self-report descriptive norms scale 1 (average DN1.1 to DN1.5) at baseline (predictor) on focal participants' values of self-report descriptive norms scale 1 at follow-up (outcome) by fear of negative evaluation (moderator) with 95% CI limits for conditional effects, and bounds indicating regions of significance at the 95% and 99% levels (indicating values of the moderator for which conditional effects differ significantly from 0).

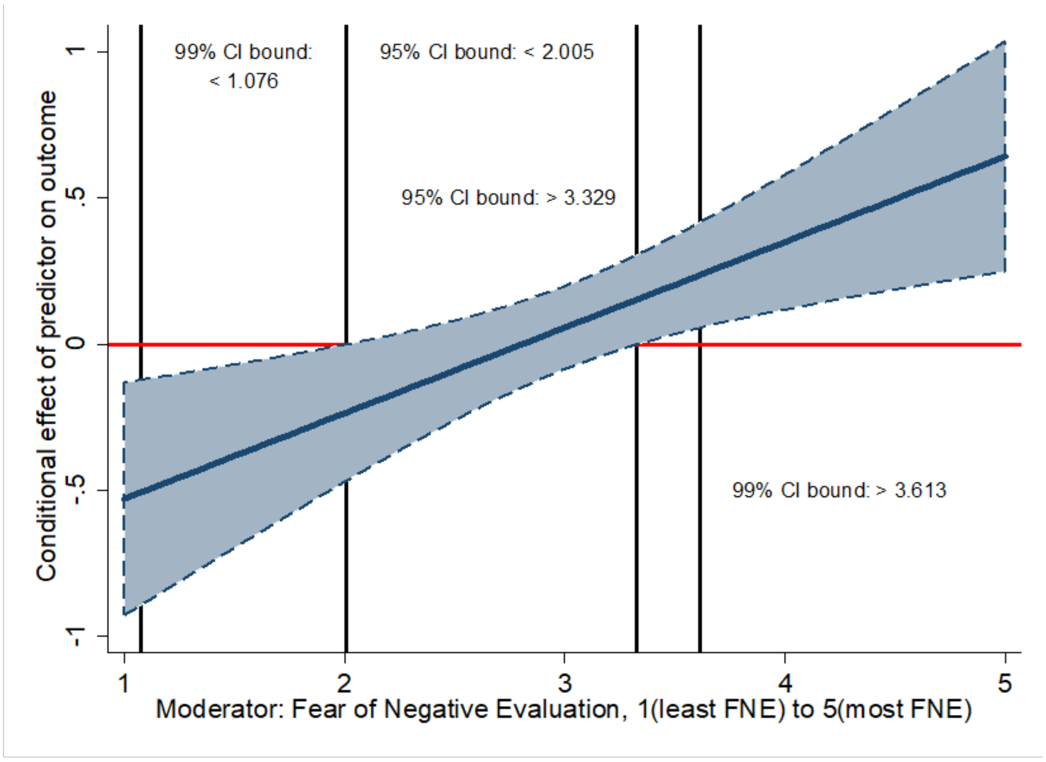

Figure S4.100. The conditional effects of peer influence from average school class responses to self-report descriptive norms scale 1 (average DN1.1 to DN1.5) at follow-up (predictor) on focal participants' values of self-report descriptive norms scale 1 at follow-up (outcome) by fear of negative evaluation (moderator) with 95% CI limits for conditional effects, and bounds indicating regions of significance at the 95% and 99% levels (indicating values of the moderator for which conditional effects differ significantly from 0).

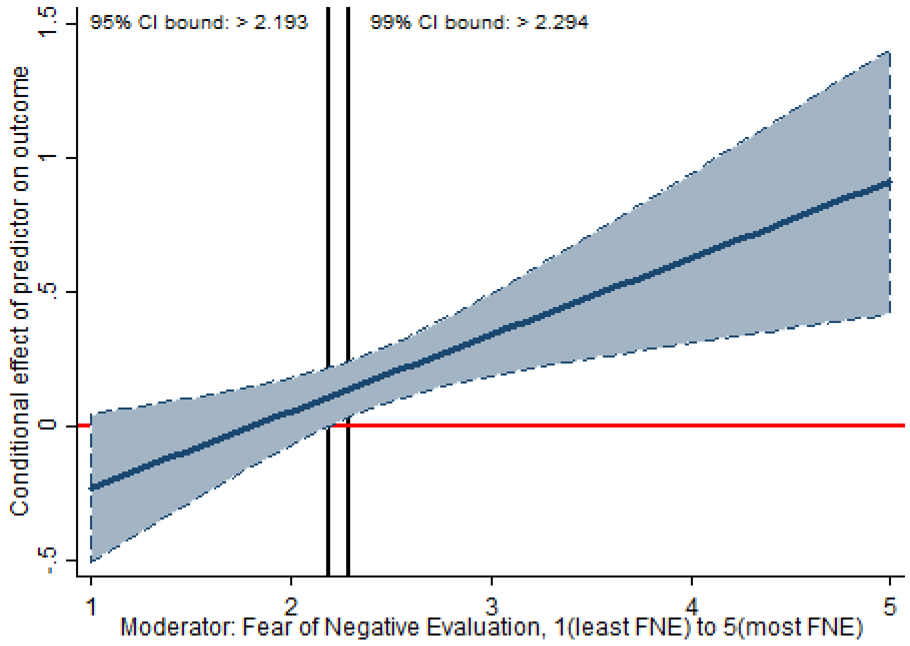

Figure S4.101. The conditional effects of peer influence from average friends' self-report smoking behavior at follow-up (predictor) on focal participants' self-report smoking behavior at follow-up (outcome) by fear of negative evaluation (moderator) with 95% CI limits for conditional effects, and bounds indicating regions of significance at the 95% and 99% levels (indicating values of the moderator for which conditional effects differ significantly from 0).

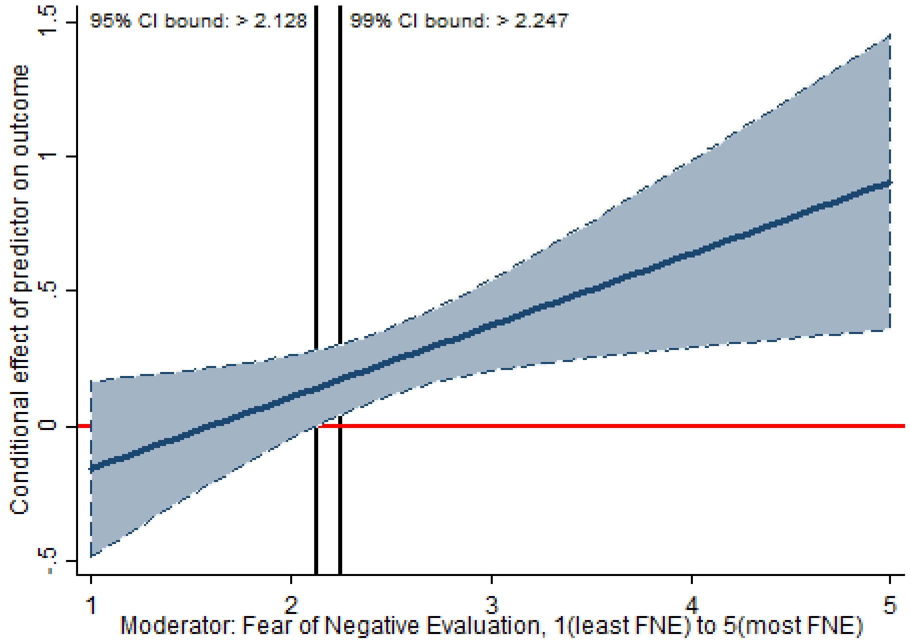

Figure S4.102. The conditional effects of peer influence from average friends' self-efficacy (emotional) at follow-up (predictor) on focal participants' self-efficacy (emotional) at follow-up (outcome) by fear of negative evaluation (moderator) with 95% CI limits for conditional effects, and bounds indicating regions of significance at the 95% and 99% levels (indicating values of the moderator for which conditional effects differ significantly from 0).

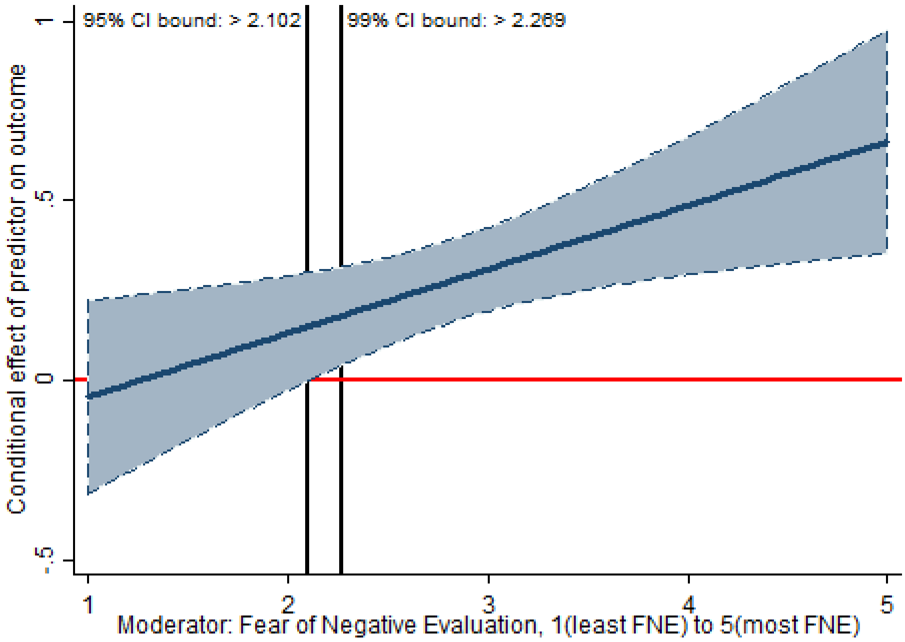

Figure S4.103. The conditional effects of peer influence from average friends' perceived behavioral control (easy to quit) at baseline (predictor) on focal participants' perceived behavioral control (easy to quit) at follow-up (outcome) by fear of negative evaluation (moderator) with 95% CI limits for conditional effects, and bounds indicating regions of significance at the 95% and 99% levels (indicating values of the moderator for which conditional effects differ significantly from 0).

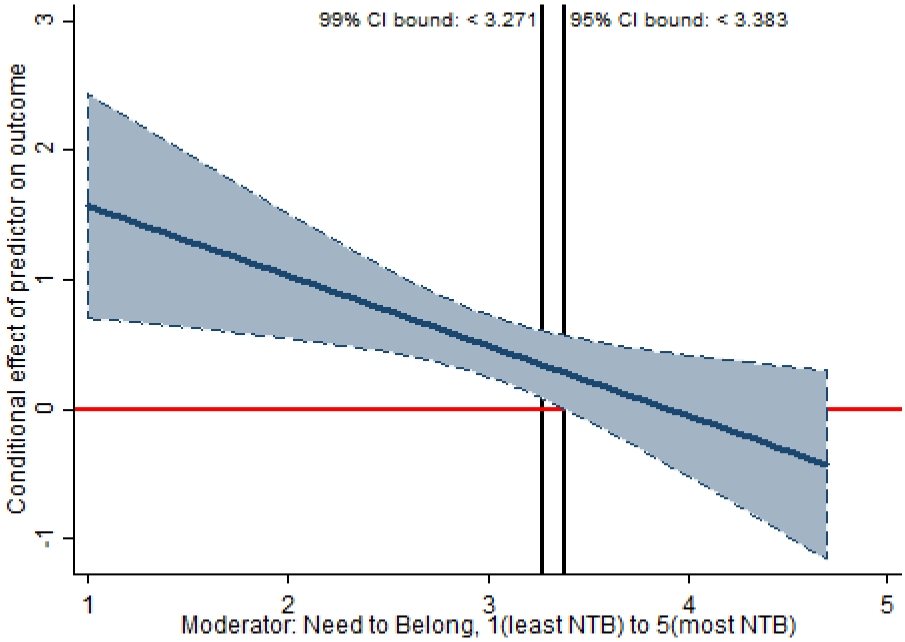

Figure S4.104. The conditional effects of peer influence from average school class responses for P2S9 at baseline (predictor) on focal participants' values of P2S9 at follow-up (outcome) by need to belong (moderator) with 95% CI limits for conditional effects, and bounds indicating regions of significance at the 95% and 99% levels (indicating values of the moderator for which conditional effects differ significantly from 0).

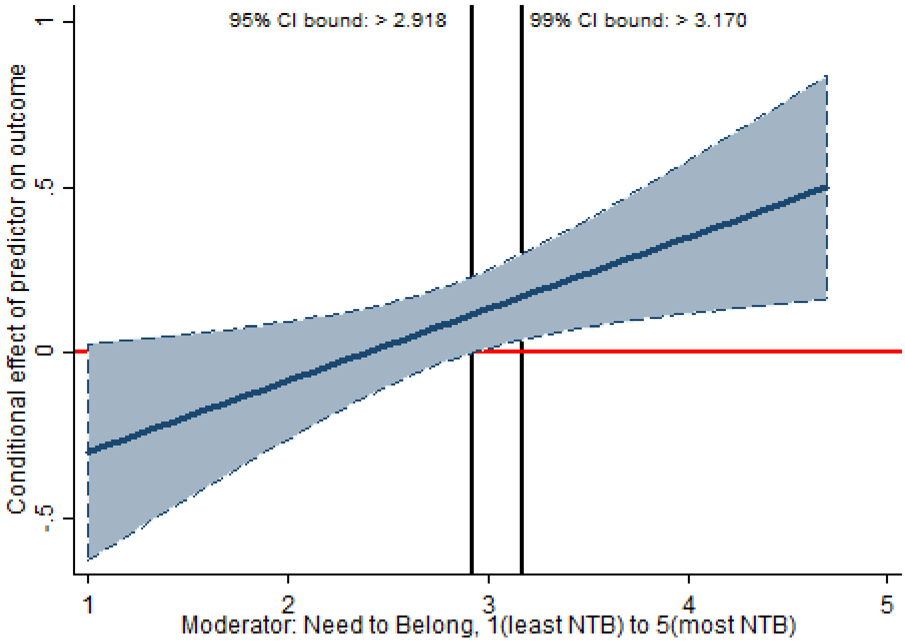

Figure S4.105. The conditional effects of peer influence from average friends' responses to IN7 at baseline (predictor) on focal participants' values of IN7 at follow-up (outcome) by need to belong (moderator) with 95% CI limits for conditional effects, and bounds indicating regions of significance at the 95% and 99% levels (indicating values of the moderator for which conditional effects differ significantly from 0).

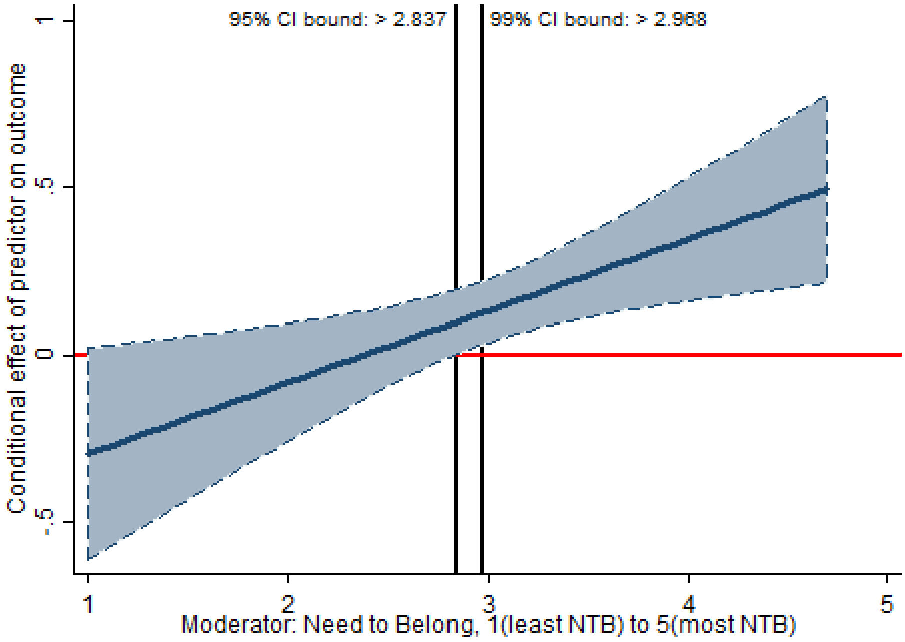

Figure S4.106. The conditional effects of peer influence from average friends' responses to DN1.2 at baseline (predictor) on focal participants' values of DN1.2 at follow-up (outcome) by need to belong (moderator) with 95% CI limits for conditional effects, and bounds indicating regions of significance at the 95% and 99% levels (indicating values of the moderator for which conditional effects differ significantly from 0).

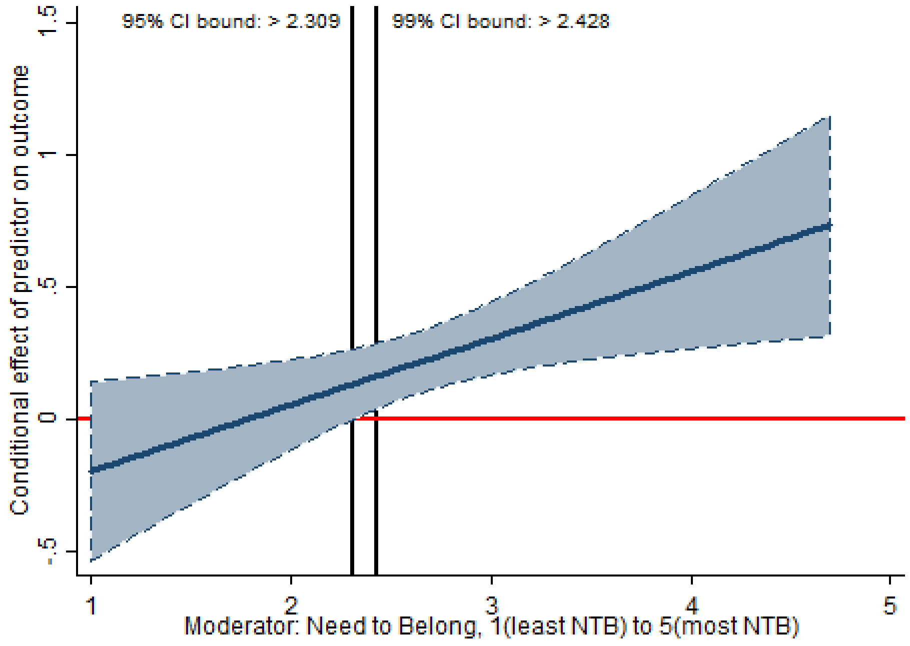

Figure S4.107. The conditional effects of peer influence from average friends' self-report smoking behavior at follow-up (predictor) on focal participants' self-report smoking behavior at follow-up (outcome) by need to belong (moderator) with 95% CI limits for conditional effects, and bounds indicating regions of significance at the 95% and 99% levels (indicating values of the moderator for which conditional effects differ significantly from 0).

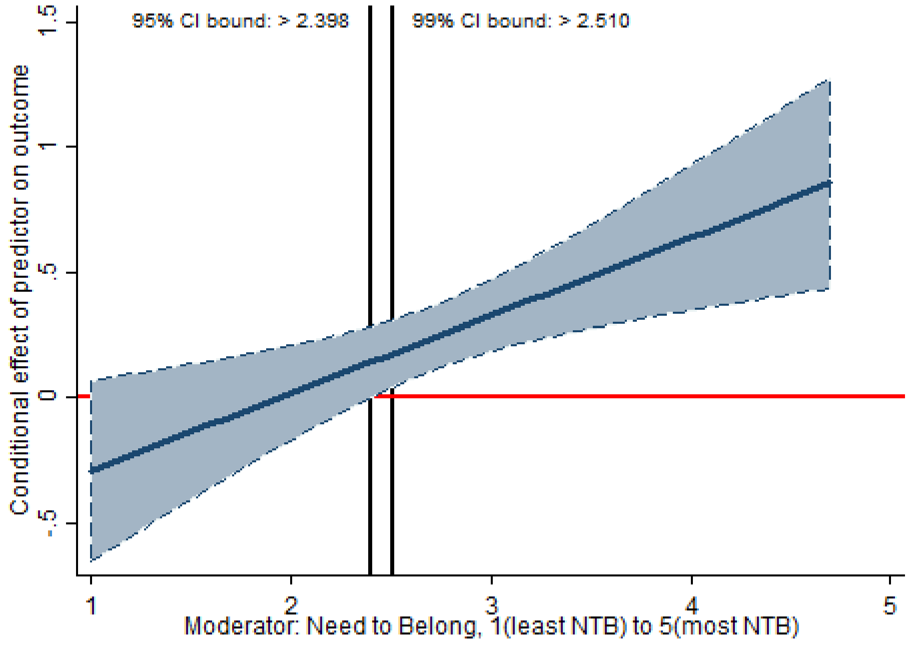

Figure S4.108. The conditional effects of peer influence from average friends' self-efficacy (emotional) at follow-up (predictor) on focal participants' self-efficacy (emotional) at follow-up (outcome) by need to belong (moderator) with 95% CI limits for conditional effects, and bounds indicating regions of significance at the 95% and 99% levels (indicating values of the moderator for which conditional effects differ significantly from 0).

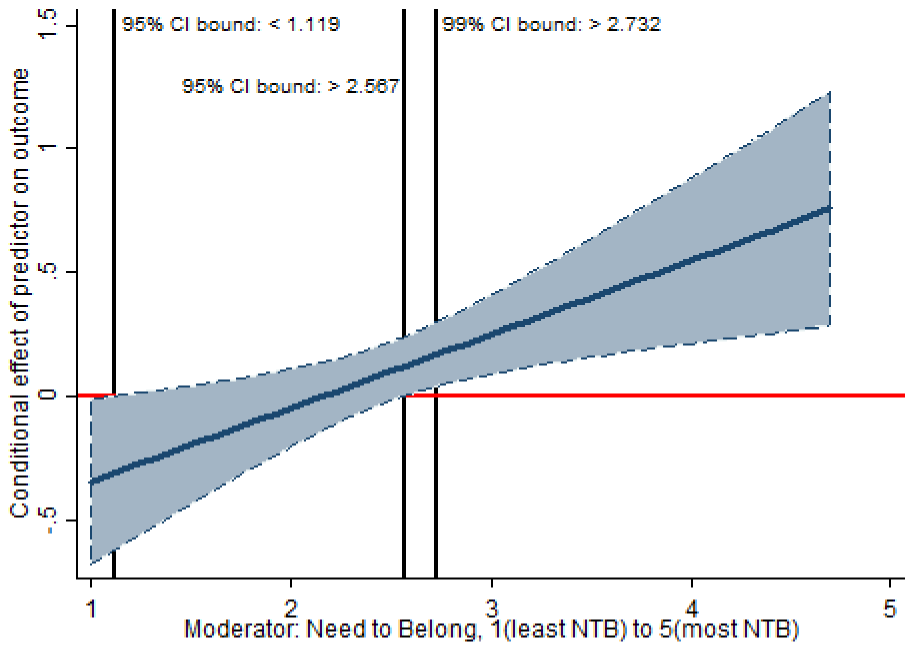

Figure S4.109. The conditional effects of peer influence from average friends' self-efficacy (opportunity) at follow-up (predictor) on focal participants' self-efficacy (opportunity) at follow-up (outcome) by need to belong (moderator) with 95% CI limits for conditional effects, and bounds indicating regions of significance at the 95% and 99% levels (indicating values of the moderator for which conditional effects differ significantly from 0).

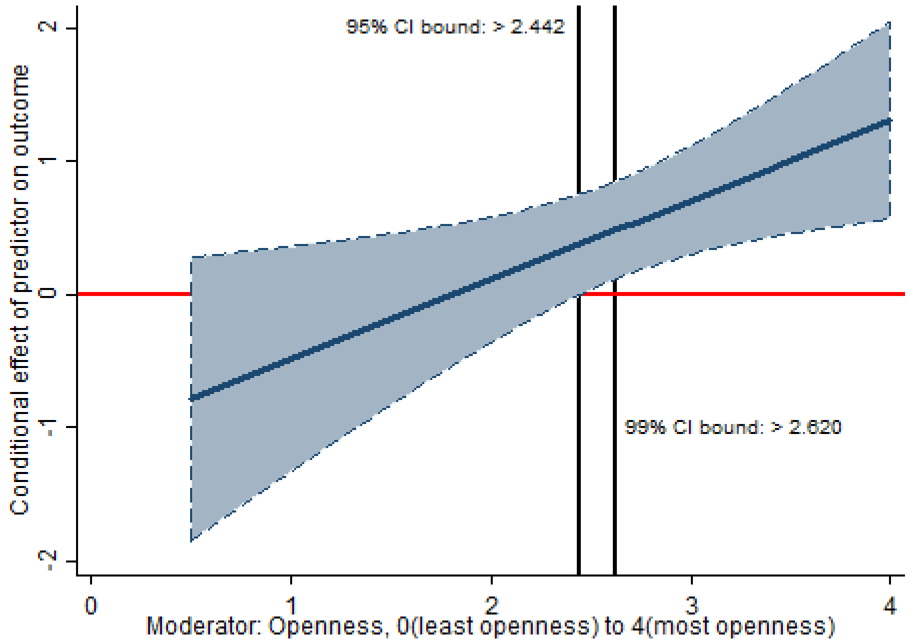

Figure S4.110. The conditional effects of peer influence from average school year group responses for P2S6 at baseline (predictor) on focal participants' values of P2S6 at follow-up (outcome) by openness (moderator) with 95% CI limits for conditional effects, and bounds indicating regions of significance at the 95% and 99% levels (indicating values of the moderator for which conditional effects differ significantly from 0).

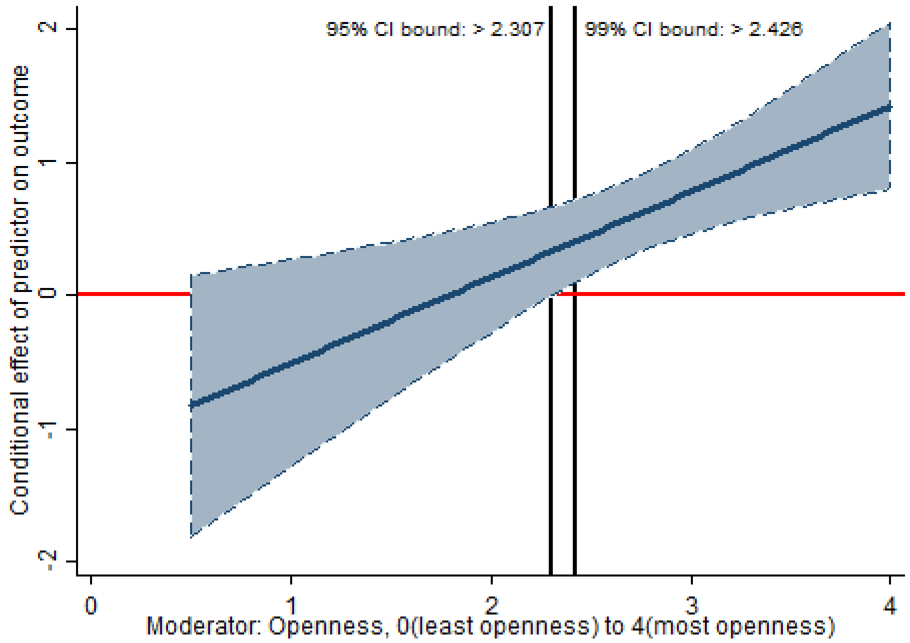

Figure S4.111. The conditional effects of peer influence from average school year group responses for P2S6 at follow-up (predictor) on focal participants' values of P2S6 at follow-up (outcome) by openness (moderator) with 95% CI limits for conditional effects, and bounds indicating regions of significance at the 95% and 99% levels (indicating values of the moderator for which conditional effects differ significantly from 0).

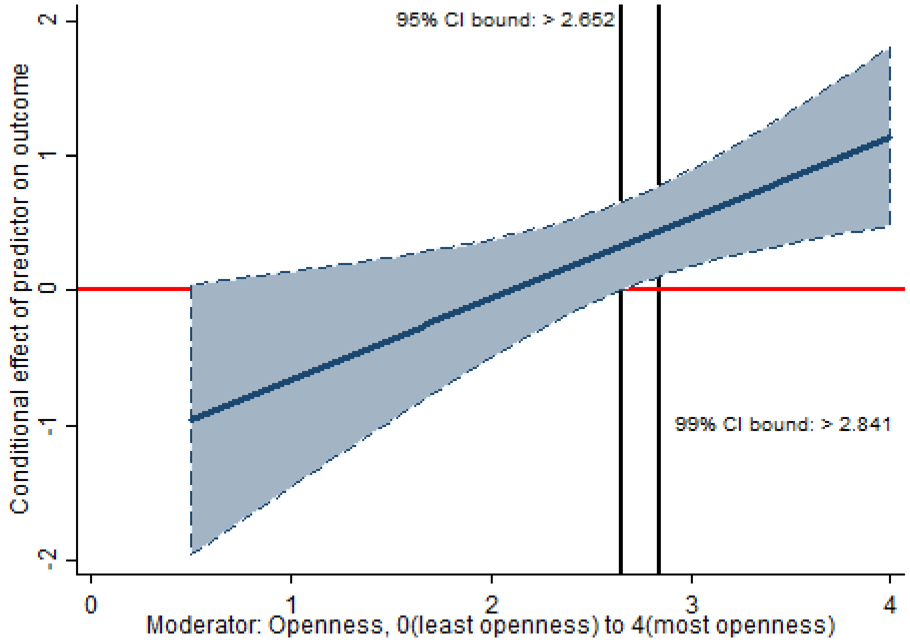

Figure S4.112. The conditional effects of peer influence from average school year group responses for P2S7 at follow-up (predictor) on focal participants' values of P2S7 at follow-up (outcome) by openness (moderator) with 95% CI limits for conditional effects, and bounds indicating regions of significance at the 95% and 99% levels (indicating values of the moderator for which conditional effects differ significantly from 0).

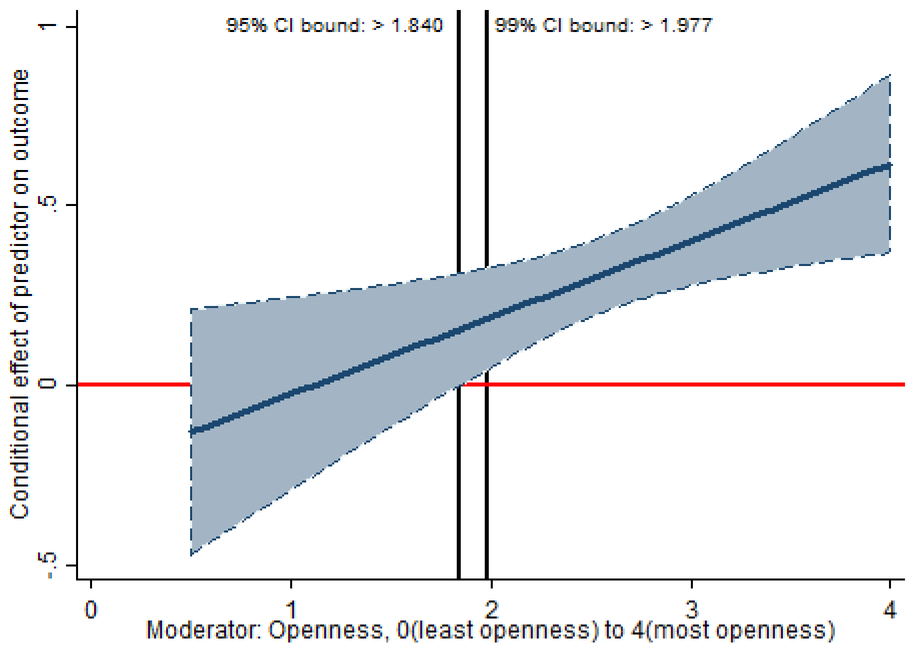

Figure S4.113. The conditional effects of peer influence from average friends' responses for P2S8 at baseline (predictor) on focal participants' values of P2S8 at follow-up (outcome) by openness (moderator) with 95% CI limits for conditional effects, and bounds indicating regions of significance at the 95% and 99% levels (indicating values of the moderator for which conditional effects differ significantly from 0).

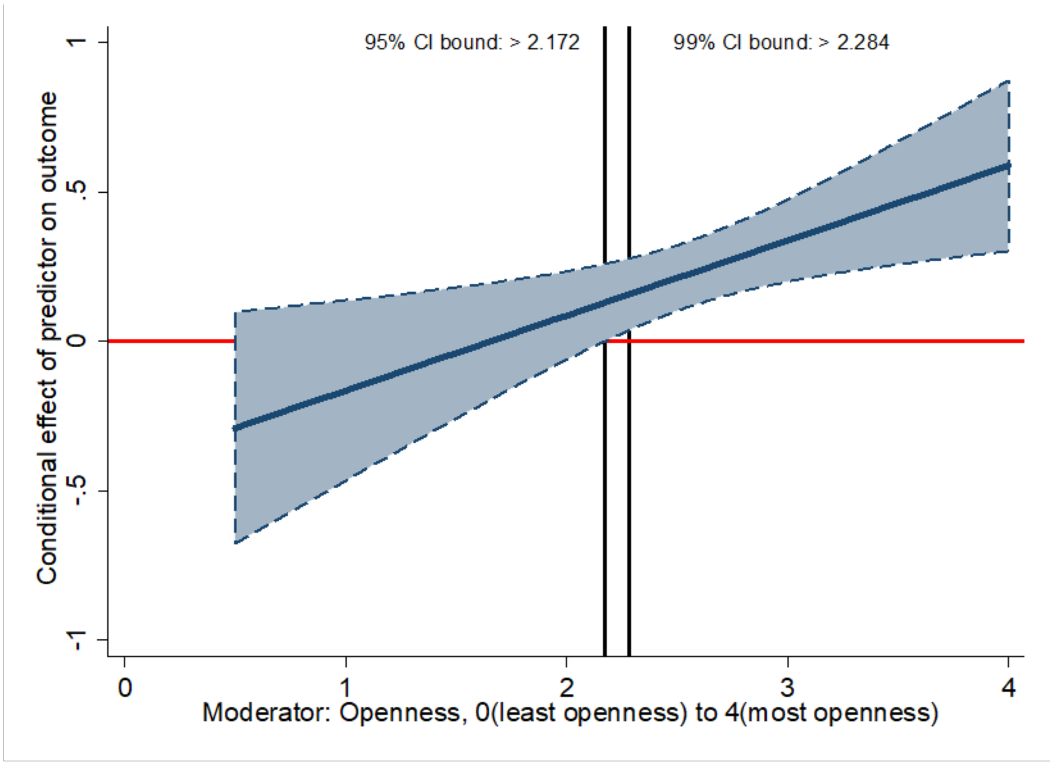

Figure S4.114. The conditional effects of peer influence from average friends' responses for experiment part 2 (injunctive norms, average P2S2 to P2S9) at baseline (predictor) on focal participants' values of experiment part 2 at follow-up (outcome) by openness (moderator) with 95% CI limits for conditional effects, and bounds indicating regions of significance at the 95% and 99% levels (indicating values of the moderator for which conditional effects differ significantly from 0).

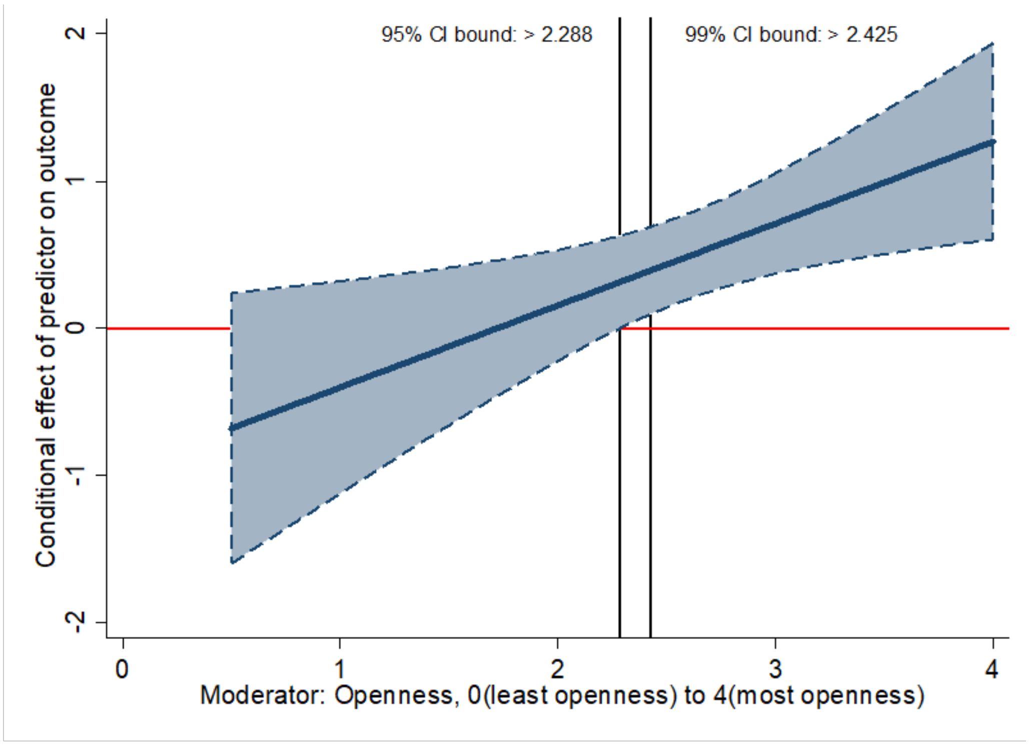

Figure S4.115. The conditional effects of peer influence from average school year group responses for experiment part 2 (injunctive norms, average P2S2 to P2S9) at baseline (predictor) on focal participants' values of experiment part 2 at follow-up (outcome) by openness (moderator) with 95% CI limits for conditional effects, and bounds indicating regions of significance at the 95% and 99% levels (indicating values of the moderator for which conditional effects differ significantly from 0).

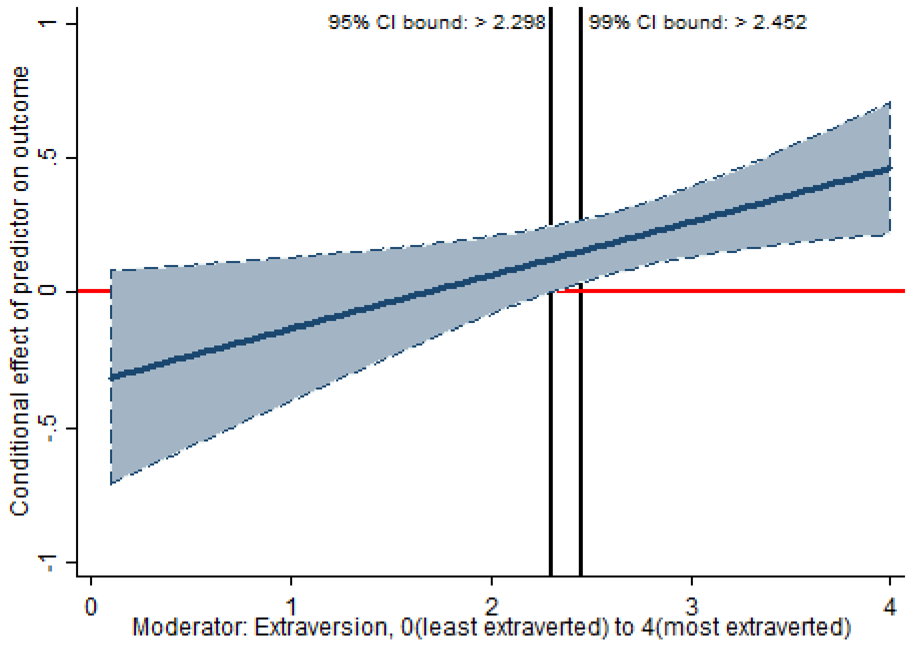

Figure S4.116. The conditional effects of peer influence from average friends' responses for P2S5 at follow-up (predictor) on focal participants' values of P2S5 at follow-up (outcome) by extraversion (moderator) with 95% CI limits for conditional effects, and bounds indicating regions of significance at the 95% and 99% levels (indicating values of the moderator for which conditional effects differ significantly from 0).

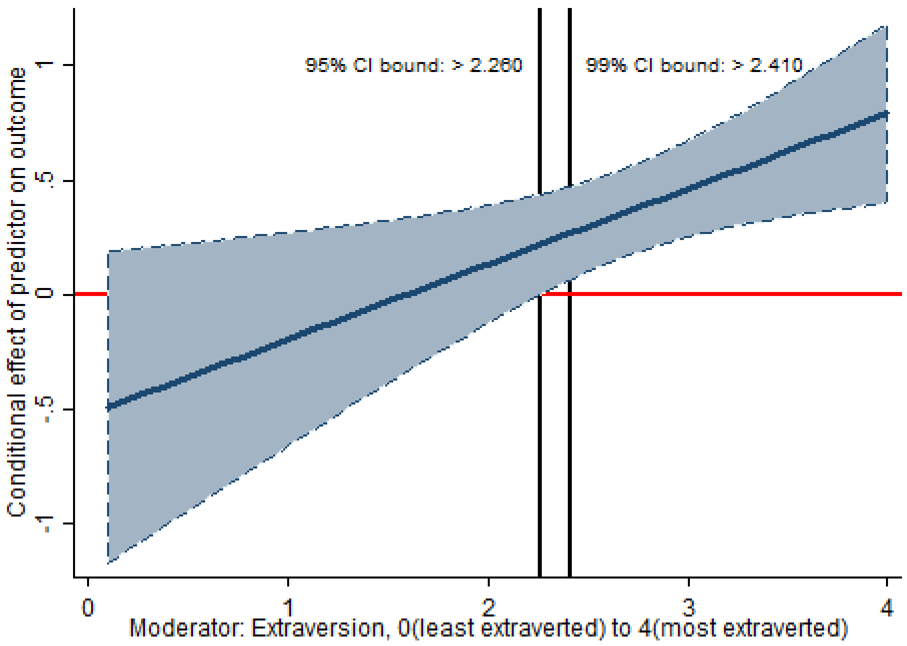

Figure S4.117. The conditional effects of peer influence from average school class responses for P2S5 at baseline (predictor) on focal participants' values of P2S5 at follow-up (outcome) by extraversion (moderator) with 95% CI limits for conditional effects, and bounds indicating regions of significance at the 95% and 99% levels (indicating values of the moderator for which conditional effects differ significantly from 0).

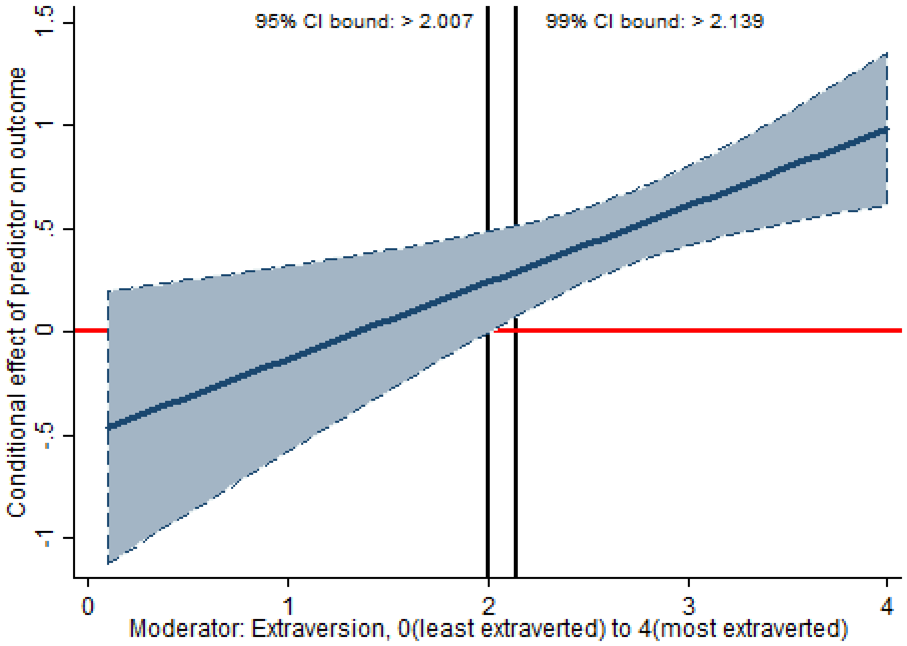

Figure S4.118. The conditional effects of peer influence from average school class responses for P2S5 at follow-up (predictor) on focal participants' values of P2S5 at follow-up (outcome) by extraversion (moderator) with 95% CI limits for conditional effects, and bounds indicating regions of significance at the 95% and 99% levels (indicating values of the moderator for which conditional effects differ significantly from 0).

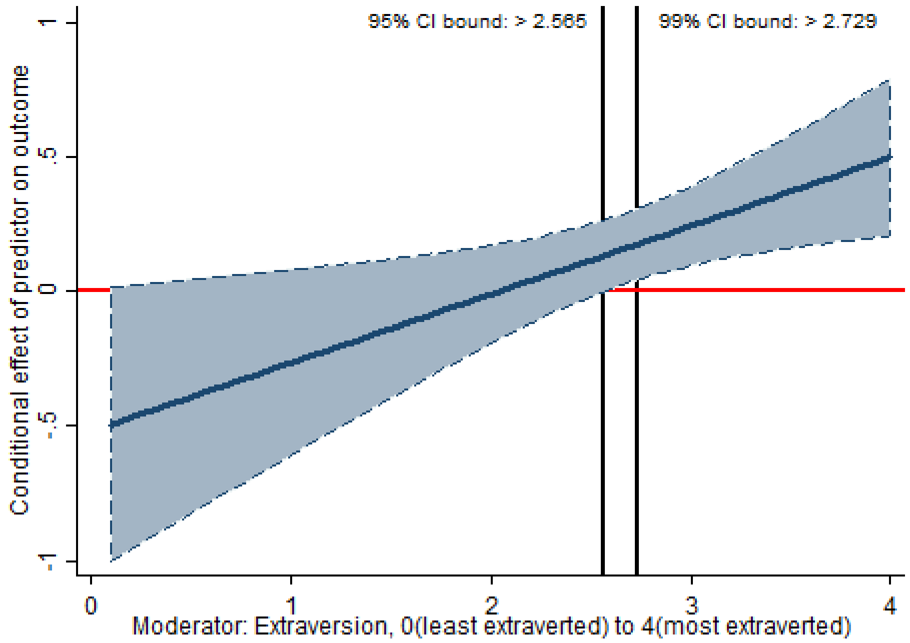

Figure S4.119. The conditional effects of peer influence from average friends' responses for P2S9 at baseline (predictor) on focal participants' values of P2S9 at follow-up (outcome) by extraversion (moderator) with 95% CI limits for conditional effects, and bounds indicating regions of significance at the 95% and 99% levels (indicating values of the moderator for which conditional effects differ significantly from 0).

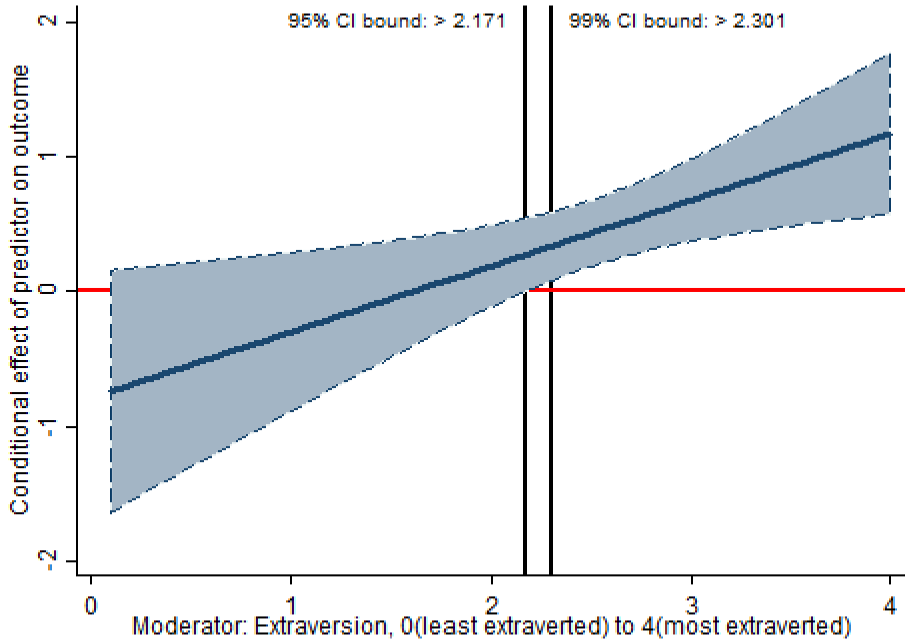

Figure S4.120. The conditional effects of peer influence from average school class responses for P2S9 at baseline (predictor) on focal participants' values of P2S9 at follow-up (outcome) by extraversion (moderator) with 95% CI limits for conditional effects, and bounds indicating regions of significance at the 95% and 99% levels (indicating values of the moderator for which conditional effects differ significantly from 0).

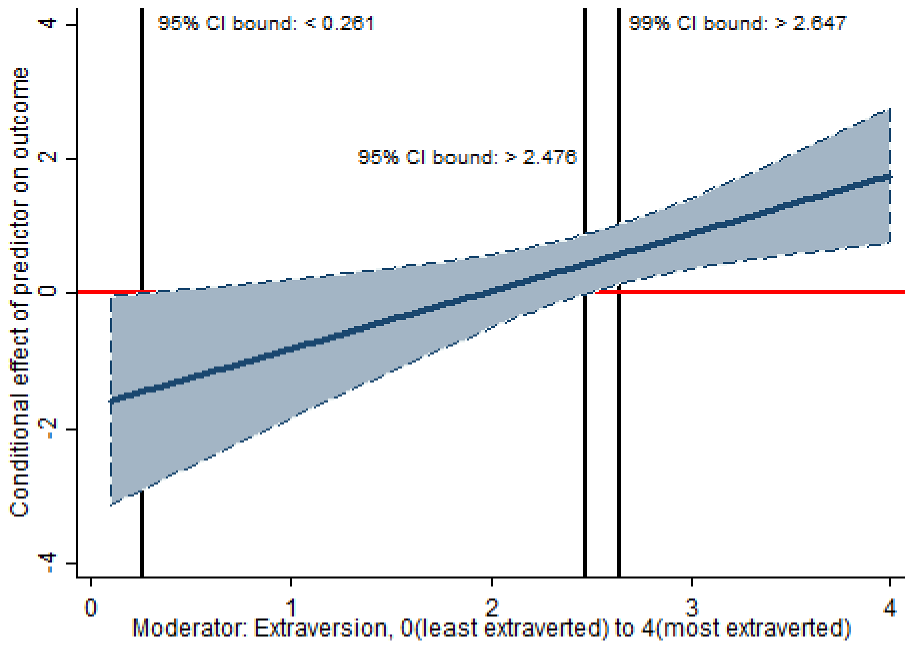

Figure S4.121. The conditional effects of peer influence from average school year group responses for P2S9 at baseline (predictor) on focal participants' values of P2S9 at follow-up (outcome) by extraversion (moderator) with 95% CI limits for conditional effects, and bounds indicating regions of significance at the 95% and 99% levels (indicating values of the moderator for which conditional effects differ significantly from 0).

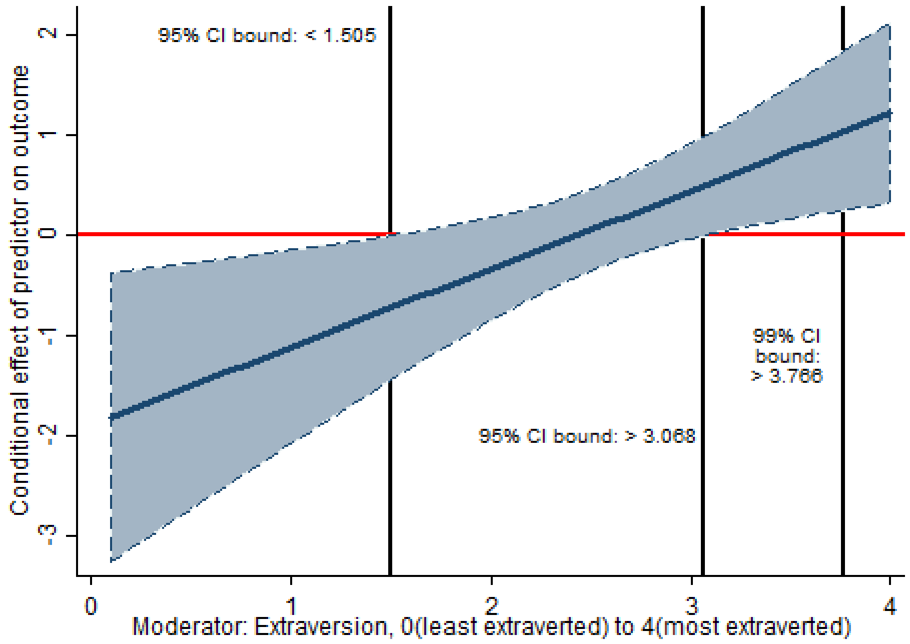

Figure S4.122. The conditional effects of peer influence from average school year group responses for P2S9 at follow-up (predictor) on focal participants' values of P2S9 at follow-up (outcome) by extraversion (moderator) with 95% CI limits for conditional effects, and bounds indicating regions of significance at the 95% and 99% levels (indicating values of the moderator for which conditional effects differ significantly from 0).

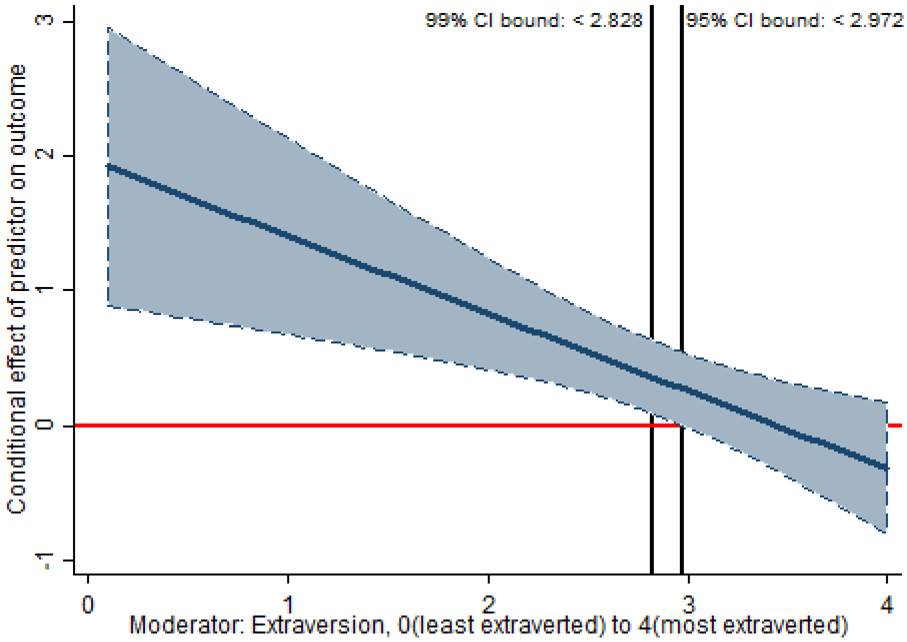

Figure S4.123. The conditional effects of peer influence from average school class intentions at baseline (predictor) on focal participants' intentions at follow-up (outcome) by extraversion (moderator) with 95% CI limits for conditional effects, and bounds indicating regions of significance at the 95% and 99% levels (indicating values of the moderator for which conditional effects differ significantly from 0).

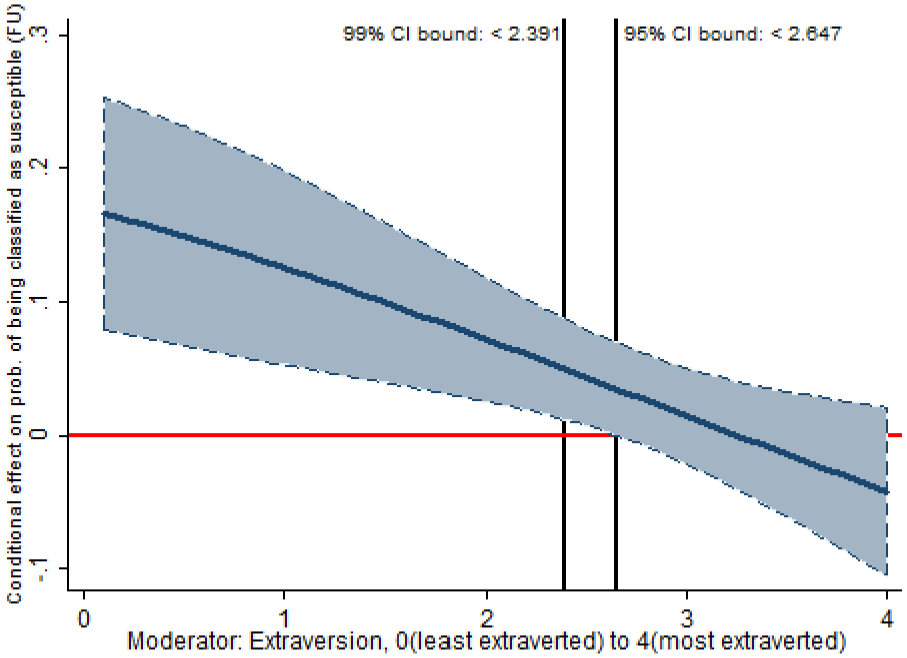

Figure S4.124. The conditional effects of peer influence from percentage school year group classified as susceptible to commencing smoking at baseline (predictor) on focal participants' probability of being classified as susceptible to commencing smoking at follow-up (outcome) by extraversion (moderator) with 95% CI limits for conditional effects, and bounds indicating regions of significance at the 95% and 99% levels (indicating values of the moderator for which conditional effects differ significantly from 0).

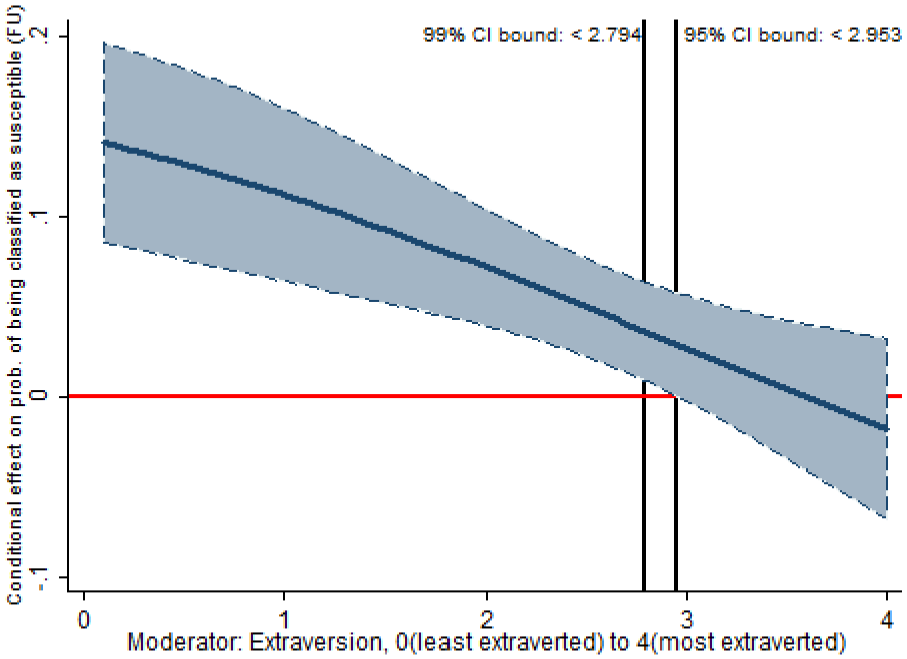

Figure S4.125. The conditional effects of peer influence from percentage school year group classified as susceptible to commencing smoking at follow-up (predictor) on focal participants' probability of being classified as susceptible to commencing smoking at follow-up (outcome) by extraversion (moderator) with 95% CI limits for conditional effects, and bounds indicating regions of significance at the 95% and 99% levels (indicating values of the moderator for which conditional effects differ significantly from 0).

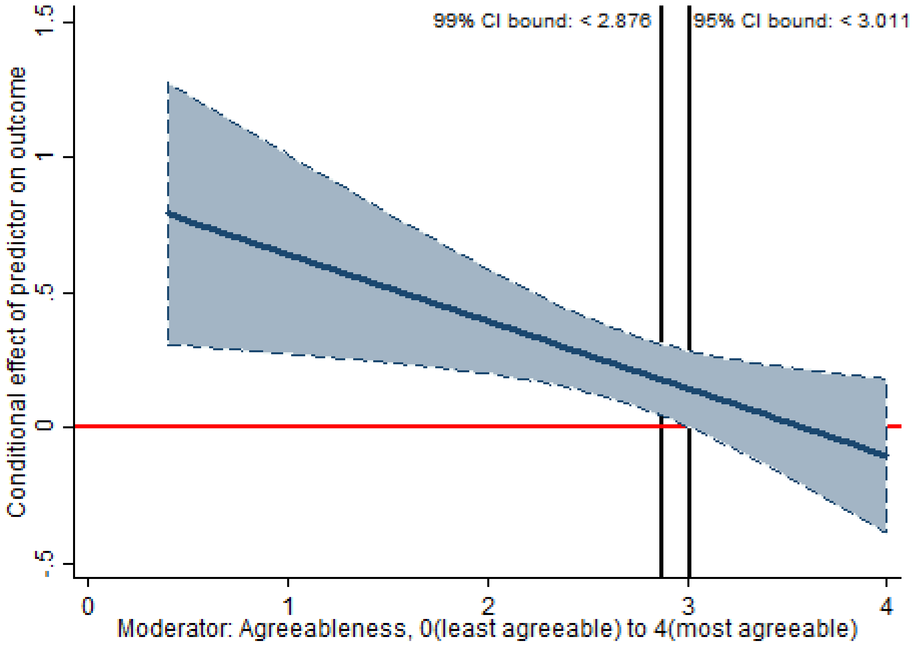

Figure S4.126. The conditional effects of peer influence from average friends’ responses for P2S9 at follow-up (predictor) on focal participants' values of P2S9 at follow-up (outcome) by agreeableness (moderator) with 95% CI limits for conditional effects, and bounds indicating regions of significance at the 95% and 99% levels (indicating values of the moderator for which conditional effects differ significantly from 0).

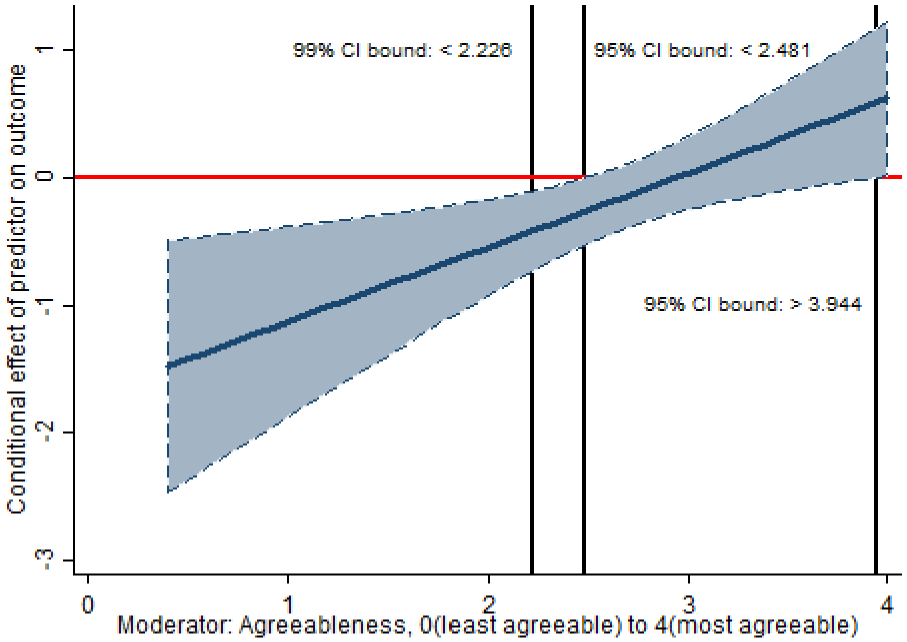

Figure S4.127. The conditional effects of peer influence from average school class responses to IN1 at follow-up (predictor) on focal participants' values of IN1 at follow-up (outcome) by agreeableness (moderator) with 95% CI limits for conditional effects, and bounds indicating regions of significance at the 95% and 99% levels (indicating values of the moderator for which conditional effects differ significantly from 0).

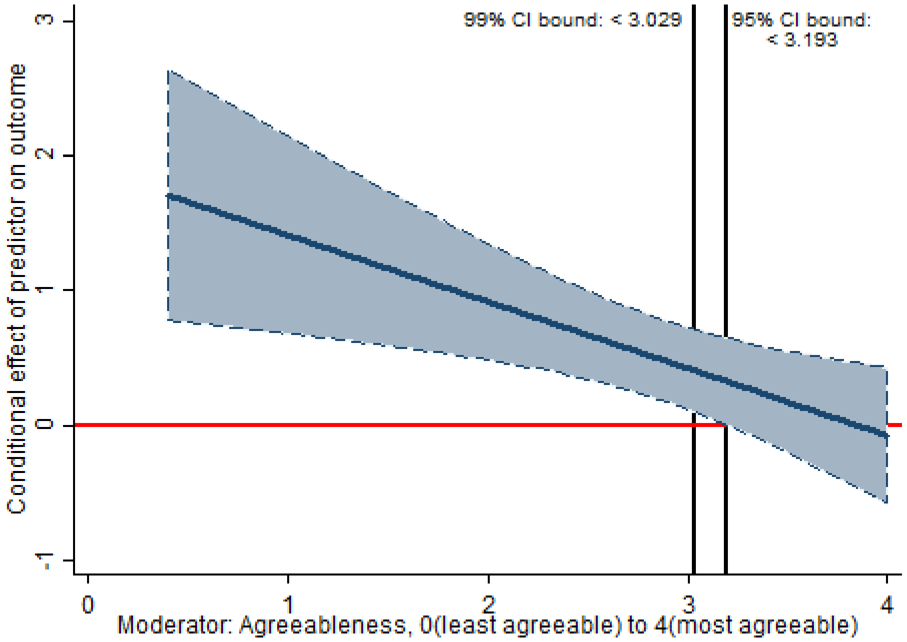

Figure S4.128. The conditional effects of peer influence from average school year group self-report smoking behavior at baseline (predictor) on focal participants' self-report smoking behavior at follow-up (outcome) by agreeableness (moderator) with 95% CI limits for conditional effects, and bounds indicating regions of significance at the 95% and 99% levels (indicating values of the moderator for which conditional effects differ significantly from 0).

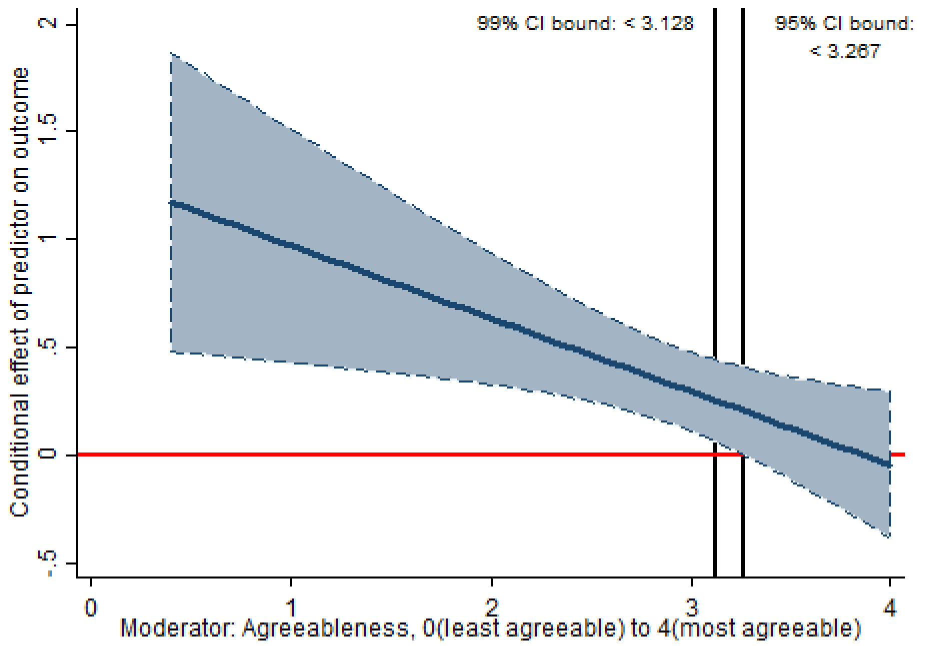

Figure S4.129. The conditional effects of peer influence from average school year group self-report smoking behavior at follow-up (predictor) on focal participants' self-report smoking behavior at follow-up (outcome) by agreeableness (moderator) with 95% CI limits for conditional effects, and bounds indicating regions of significance at the 95% and 99% levels (indicating values of the moderator for which conditional effects differ significantly from 0).

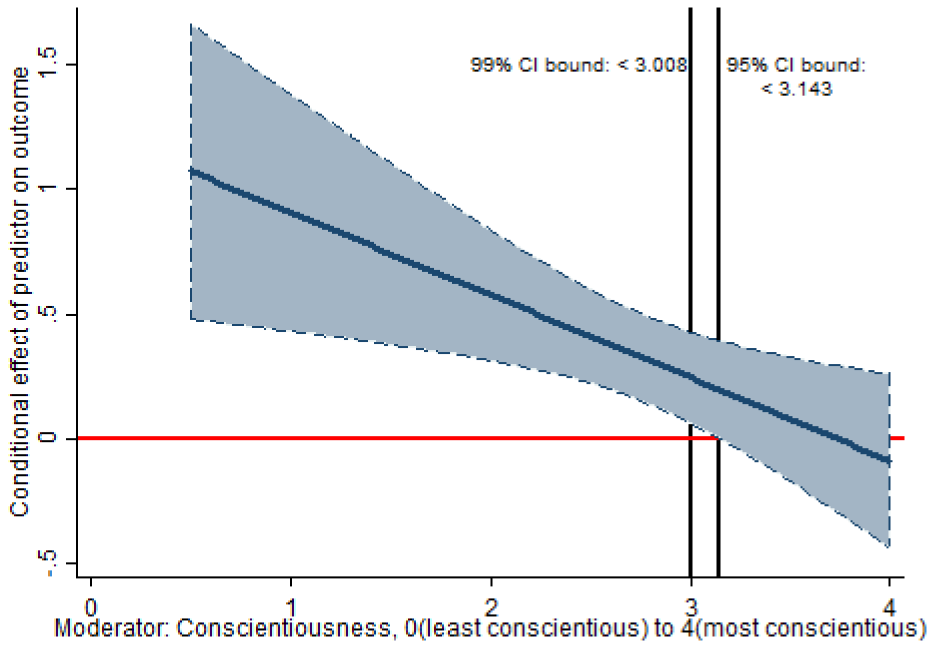

Figure S4.130. The conditional effects of peer influence from average school year group self-report smoking behavior at follow-up (predictor) on focal participants' self-report smoking behavior at follow-up (outcome) by conscientiousness (moderator) with 95% CI limits for conditional effects, and bounds indicating regions of significance at the 95% and 99% levels (indicating values of the moderator for which conditional effects differ significantly from 0).

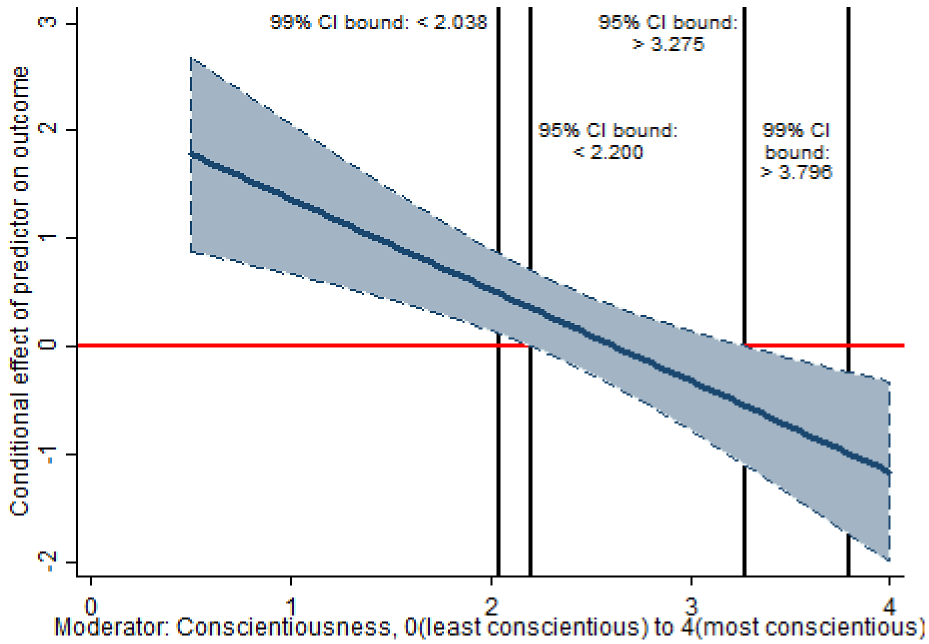

Figure S4.131. The conditional effects of peer influence from average school year group attitudes at follow-up (predictor) on focal participants' attitudes at follow-up (outcome) by conscientiousness (moderator) with 95% CI limits for conditional effects, and bounds indicating regions of significance at the 95% and 99% levels (indicating values of the moderator for which conditional effects differ significantly from 0).

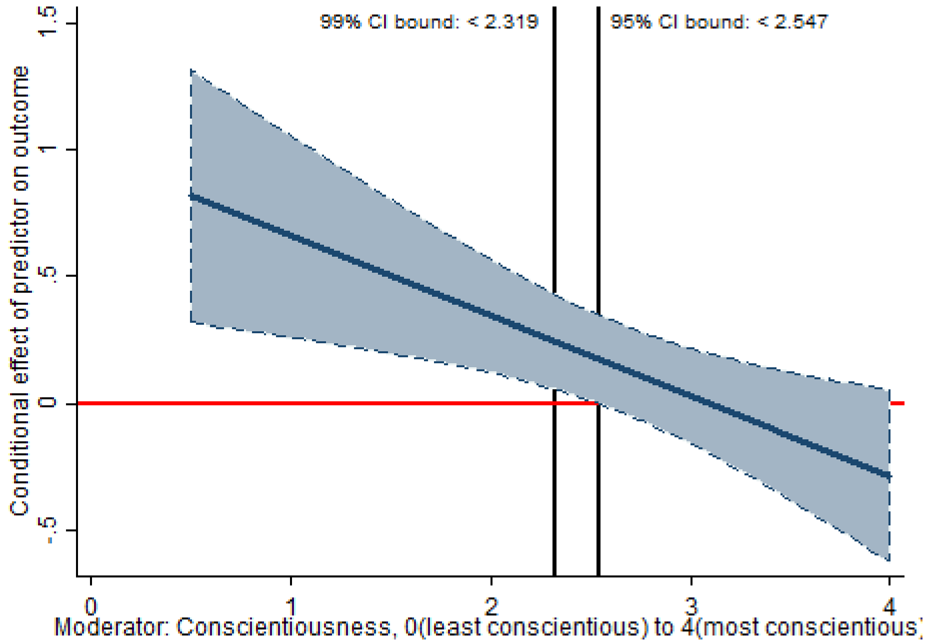

Figure S4.132. The conditional effects of peer influence from average school class self-efficacy (emotional) at follow-up (predictor) on focal participants' self-efficacy (emotional) at follow-up (outcome) by conscientiousness (moderator) with 95% CI limits for conditional effects, and bounds indicating regions of significance at the 95% and 99% levels (indicating values of the moderator for which conditional effects differ significantly from 0).

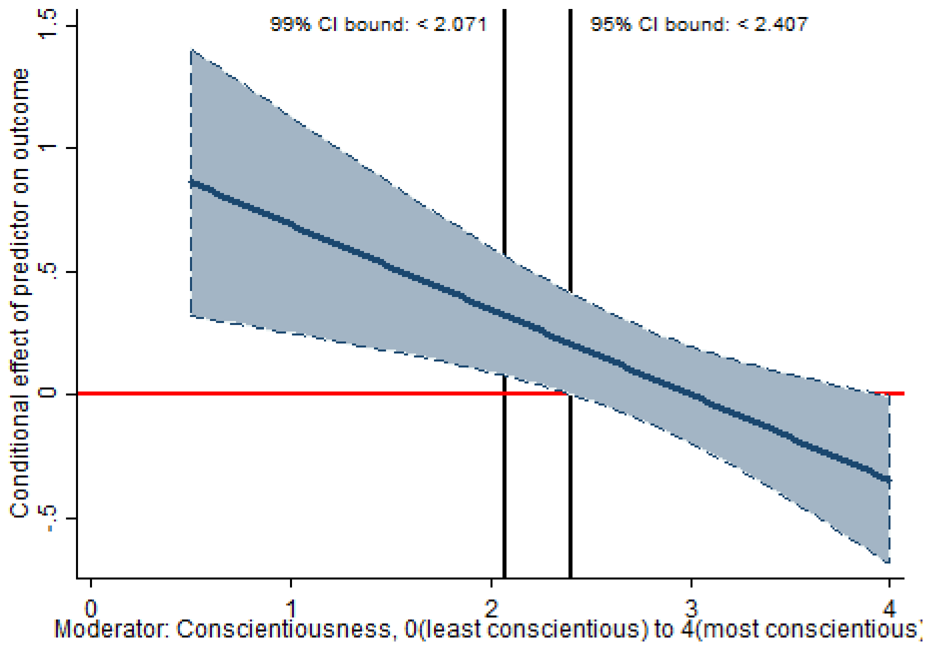

Figure S4.133. The conditional effects of peer influence from average school class self-efficacy (opportunity) at follow-up (predictor) on focal participants' self-efficacy (opportunity) at follow-up (outcome) by conscientiousness (moderator) with 95% CI limits for conditional effects, and bounds indicating regions of significance at the 95% and 99% levels (indicating values of the moderator for which conditional effects differ significantly from 0).

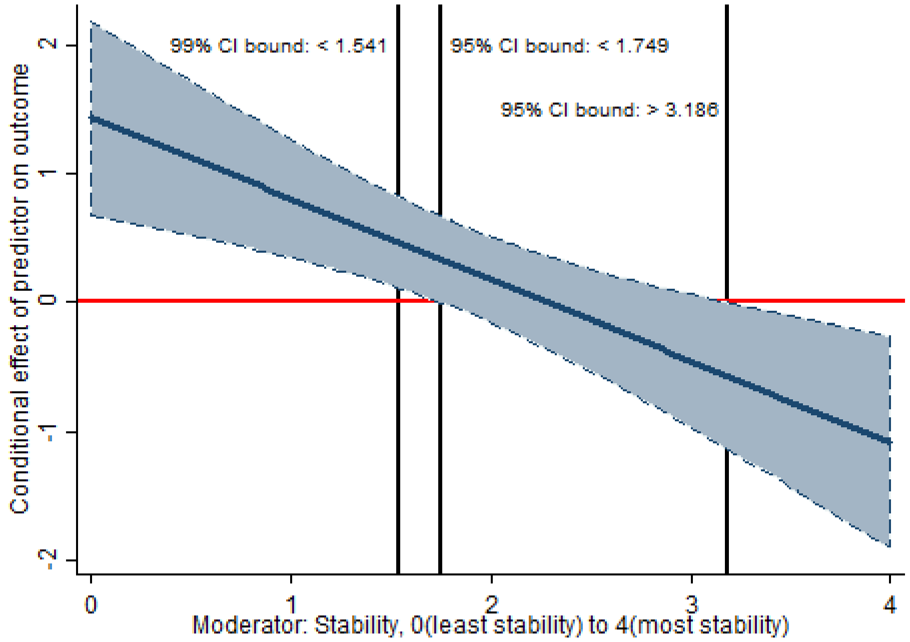

Figure S4.134. The conditional effects of peer influence from average school year group attitudes at follow-up (predictor) on focal participants' attitudes at follow-up (outcome) by emotional stability (moderator) with 95% CI limits for conditional effects, and bounds indicating regions of significance at the 95% and 99% levels (indicating values of the moderator for which conditional effects differ significantly from 0).

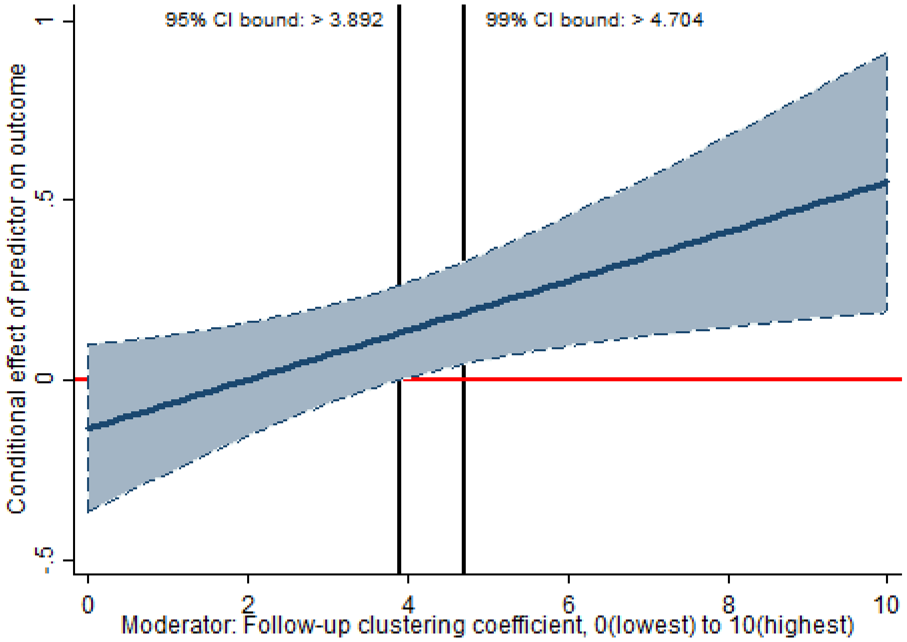

Figure S4.135. The conditional effects of peer influence from average friends' responses for P2S3 at follow-up (predictor) on focal participants' values of P2S3 at follow-up (outcome) by social network clustering coefficients at follow-up (moderator) with 95% CI limits for conditional effects, and bounds indicating regions of significance at the 95% and 99% levels (indicating values of the moderator for which conditional effects differ significantly from 0).

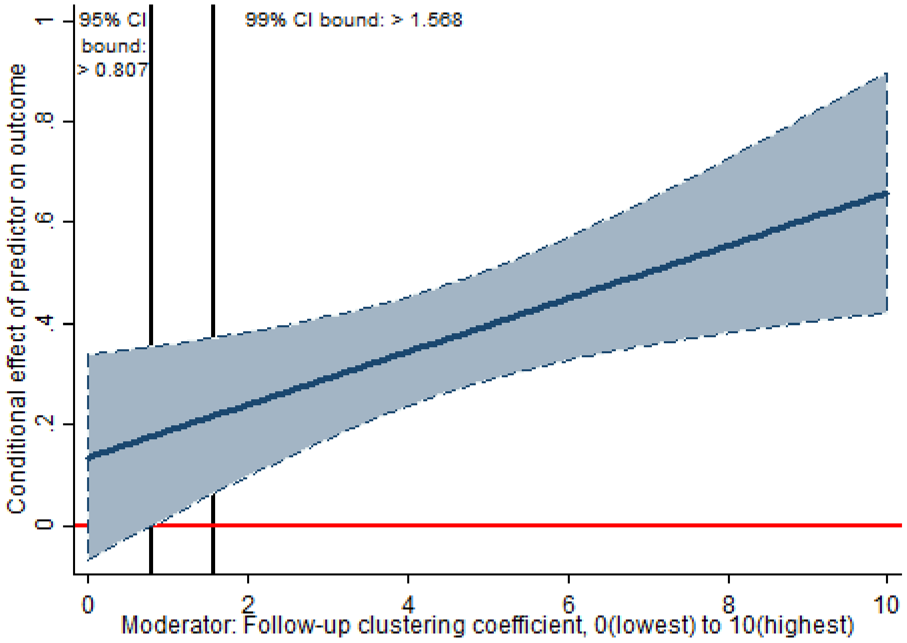

Figure S4.136. The conditional effects of peer influence from average friends' donations to ASSIST/Dead Cool at follow-up (predictor) on focal participants' donations to ASSIST/Dead Cool at follow-up (outcome) by social network clustering coefficients at follow-up (moderator) with 95% CI limits for conditional effects, and bounds indicating regions of significance at the 95% and 99% levels (indicating values of the moderator for which conditional effects differ significantly from 0).

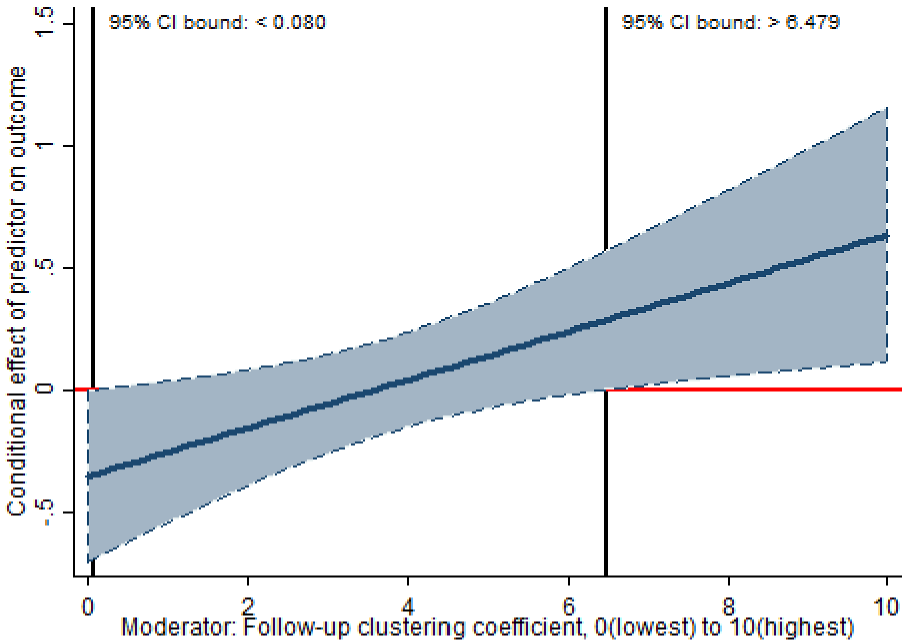

Figure S4.137. The conditional effects of peer influence from average school class responses to IN3 at follow-up (predictor) on focal participants' values of IN3 at follow-up (outcome) by social network clustering coefficients at follow-up (moderator) with 95% CI limits for conditional effects, and bounds indicating regions of significance at the 95% level (indicating values of the moderator for which conditional effects differ significantly from 0).

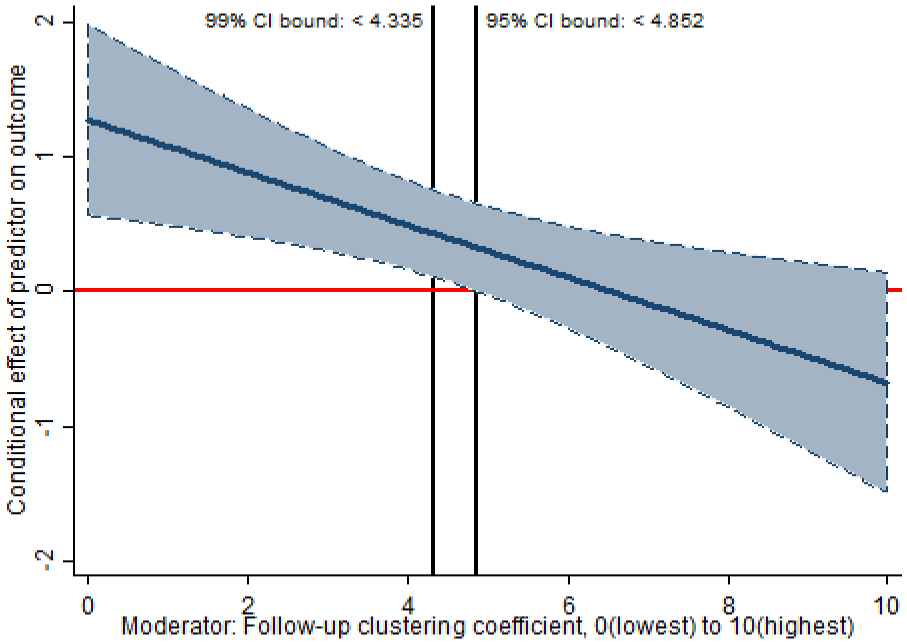

Figure S4.138. The conditional effects of peer influence from average school year group responses to DN1.1 at follow-up (predictor) on focal participants' values of DN1.1 at follow-up (outcome) by social network clustering coefficients at follow-up (moderator) with 95% CI limits for conditional effects, and bounds indicating regions of significance at the 95% and 99% levels (indicating values of the moderator for which conditional effects differ significantly from 0).

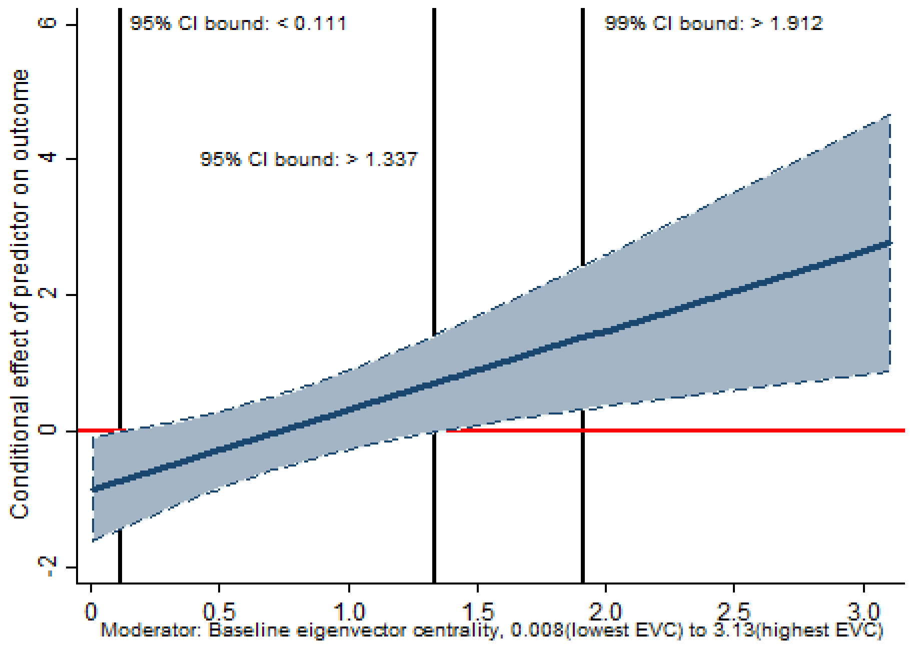

Figure S4.139. The conditional effects of peer influence from average school year group responses for P2S3 at baseline (predictor) on focal participants' values of P2S3 at follow-up (outcome) by social network eigenvector centralities at baseline (moderator) with 95% CI limits for conditional effects, and bounds indicating regions of significance at the 95% and 99% levels (indicating values of the moderator for which conditional effects differ significantly from 0).

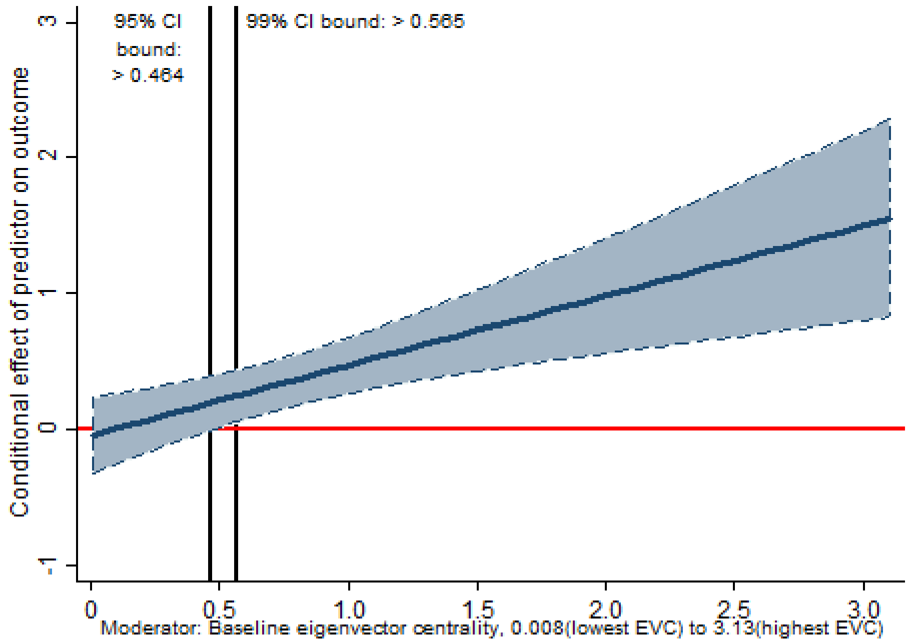

Figure S4.140. The conditional effects of peer influence from average school class responses for P2S5 at baseline (predictor) on focal participants' values of P2S5 at follow-up (outcome) by social network eigenvector centralities at baseline (moderator) with 95% CI limits for conditional effects, and bounds indicating regions of significance at the 95% and 99% levels (indicating values of the moderator for which conditional effects differ significantly from 0).

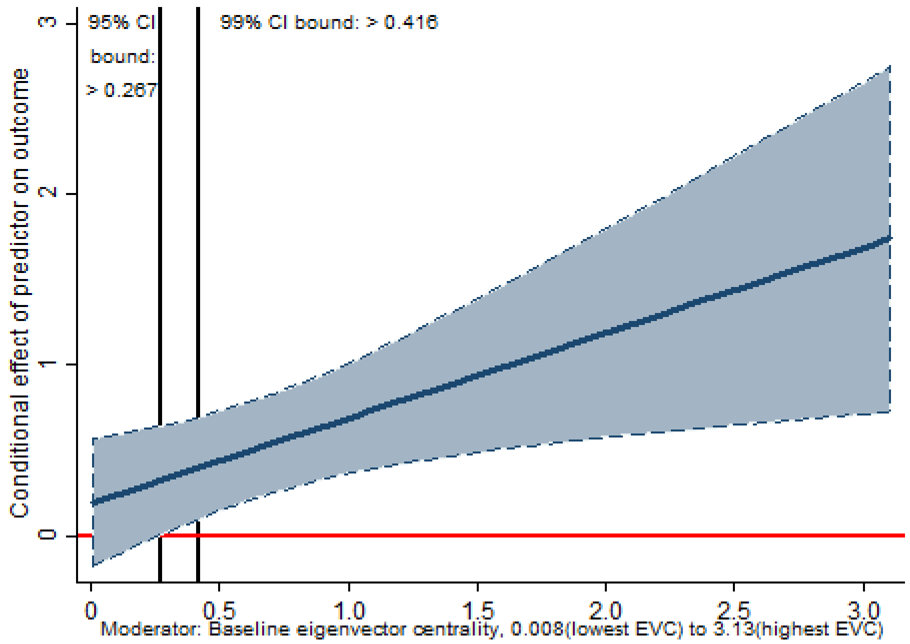

Figure S4.141. The conditional effects of peer influence from average school year group responses for P2S5 at baseline (predictor) on focal participants' values of P2S5 at follow-up (outcome) by social network eigenvector centralities at baseline (moderator) with 95% CI limits for conditional effects, and bounds indicating regions of significance at the 95% and 99% levels (indicating values of the moderator for which conditional effects differ significantly from 0).

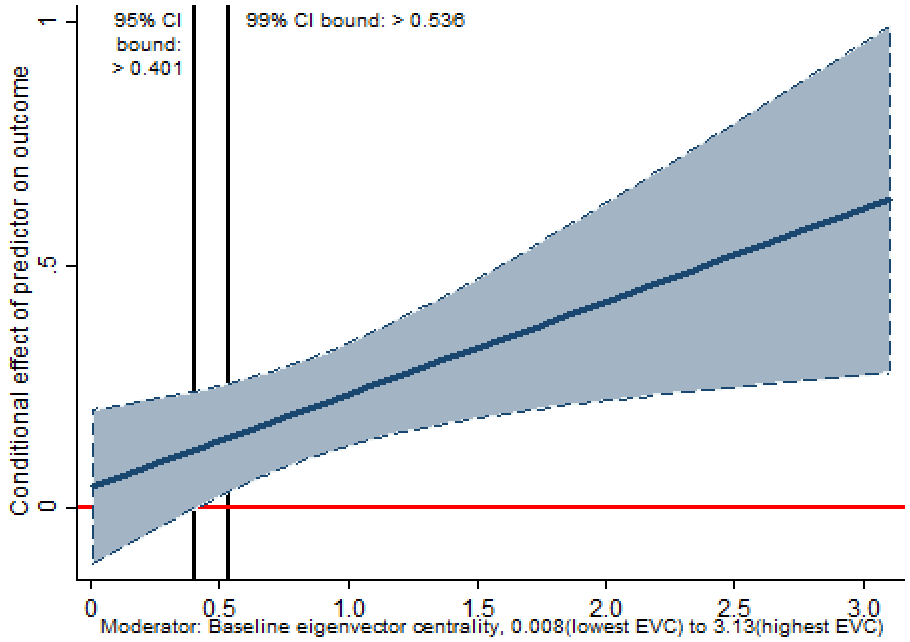

Figure S4.142. The conditional effects of peer influence from average friends' responses for P3Q2 at baseline (predictor) on focal participants' values of P3Q2 at follow-up (outcome) by social network eigenvector centralities at baseline (moderator) with 95% CI limits for conditional effects, and bounds indicating regions of significance at the 95% and 99% levels (indicating values of the moderator for which conditional effects differ significantly from 0).

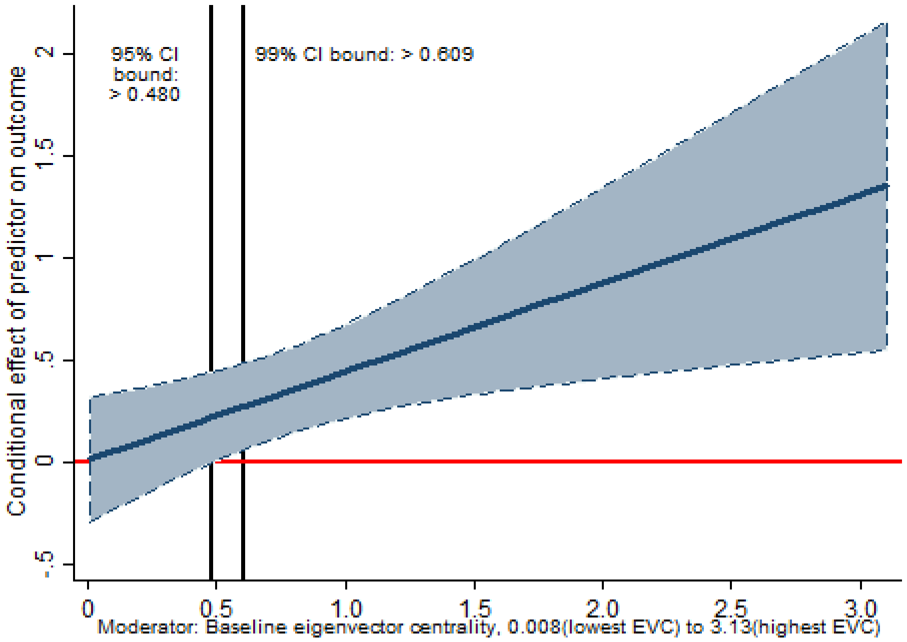

Figure S4.143. The conditional effects of peer influence from average school year group responses for P3Q2 at baseline (predictor) on focal participants' values of P3Q2 at follow-up (outcome) by social network eigenvector centralities at baseline (moderator) with 95% CI limits for conditional effects, and bounds indicating regions of significance at the 95% and 99% levels (indicating values of the moderator for which conditional effects differ significantly from 0).

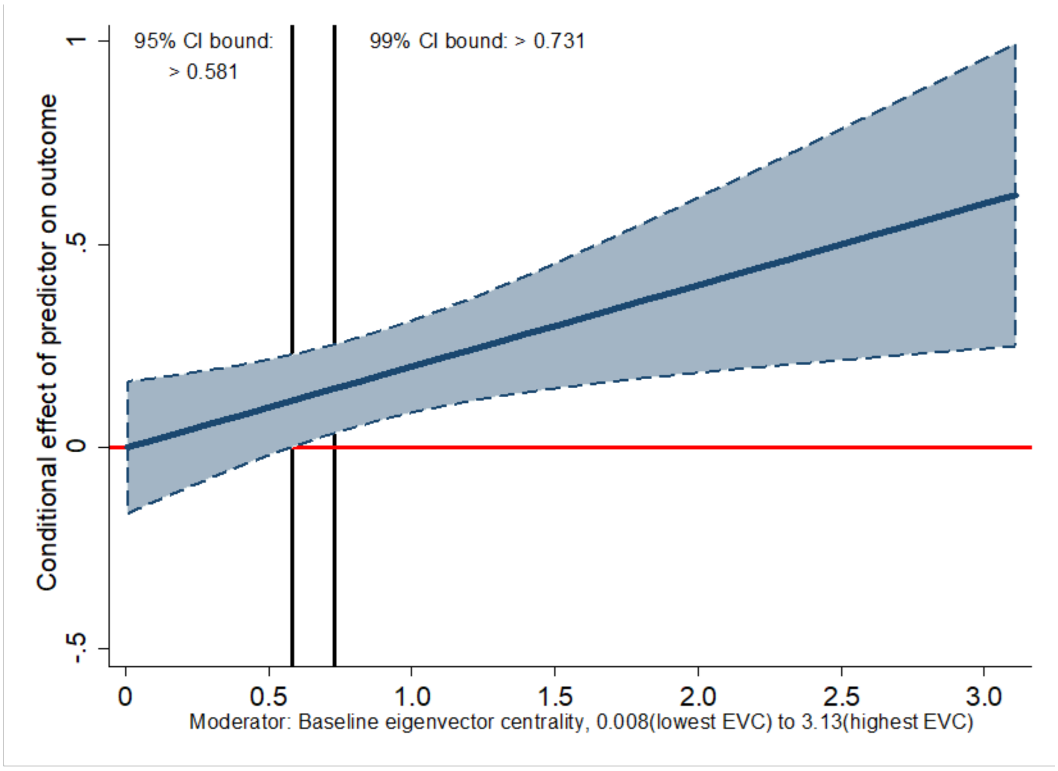

Figure S4.144. The conditional effects of peer influence from average friends' responses for experiment part 3 (descriptive norms, average P3Q1 to P3Q2) at baseline (predictor) on focal participants' values of experiment part 3 at follow-up (outcome) by social network eigenvector centralities at baseline (moderator) with 95% CI limits for conditional effects, and bounds indicating regions of significance at the 95% and 99% levels (indicating values of the moderator for which conditional effects differ significantly from 0).

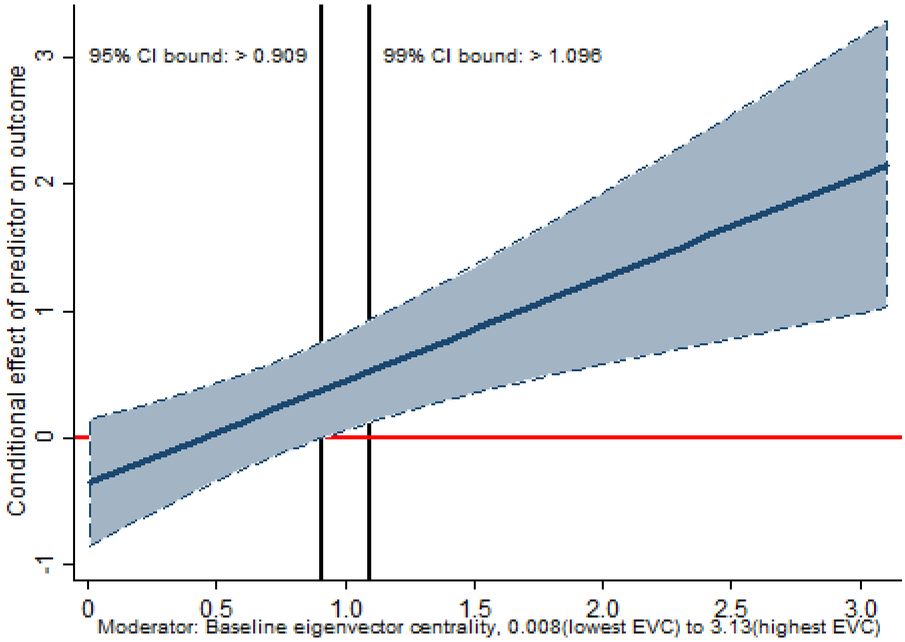

Figure S4.145. The conditional effects of peer influence from average school year group responses to IN3 at baseline (predictor) on focal participants' values of IN3 at follow-up (outcome) by social network eigenvector centralities at baseline (moderator) with 95% CI limits for conditional effects, and bounds indicating regions of significance at the 95% and 99% levels (indicating values of the moderator for which conditional effects differ significantly from 0).

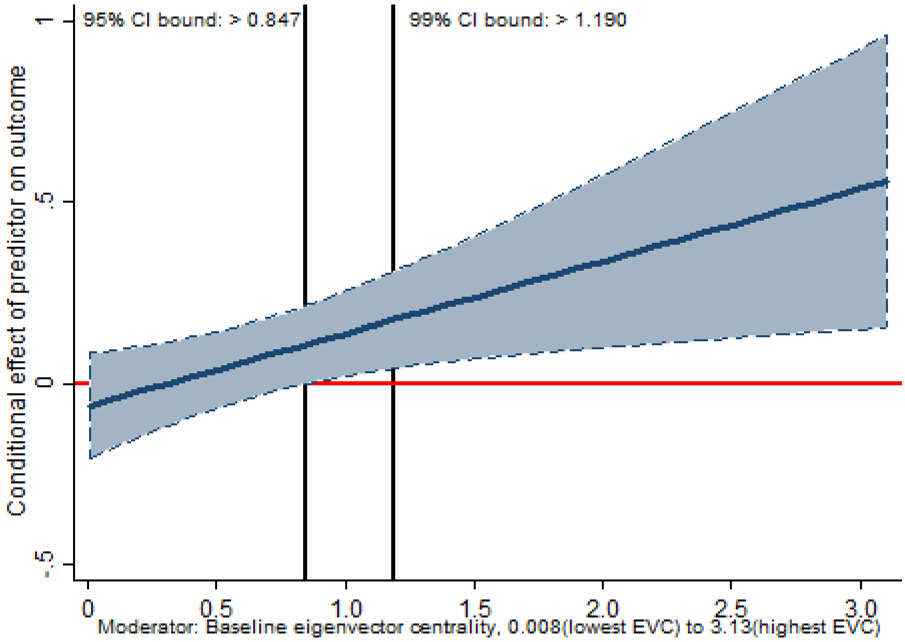

Figure S4.146. The conditional effects of peer influence from average friends' perceived physical risks at baseline (predictor) on focal participants' perceived physical risks at follow-up (outcome) by social network eigenvector centralities at baseline (moderator) with 95% CI limits for conditional effects, and bounds indicating regions of significance at the 95% and 99% levels (indicating values of the moderator for which conditional effects differ significantly from 0).

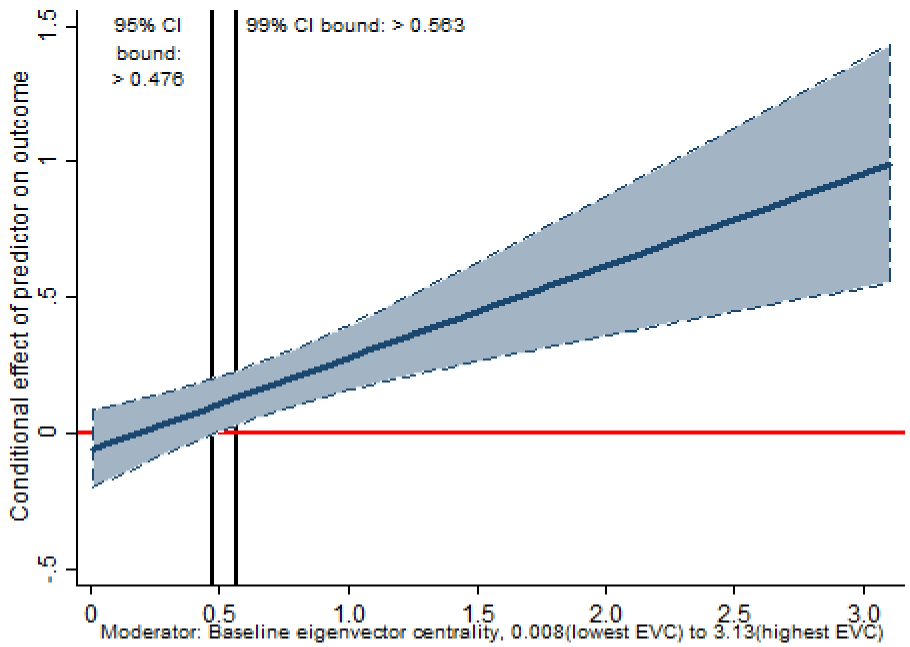

Figure S4.147. The conditional effects of peer influence from average friends' perceived social risks at baseline (predictor) on focal participants' perceived social risks at follow-up (outcome) by social network eigenvector centralities at baseline (moderator) with 95% CI limits for conditional effects, and bounds indicating regions of significance at the 95% and 99% levels (indicating values of the moderator for which conditional effects differ significantly from 0).

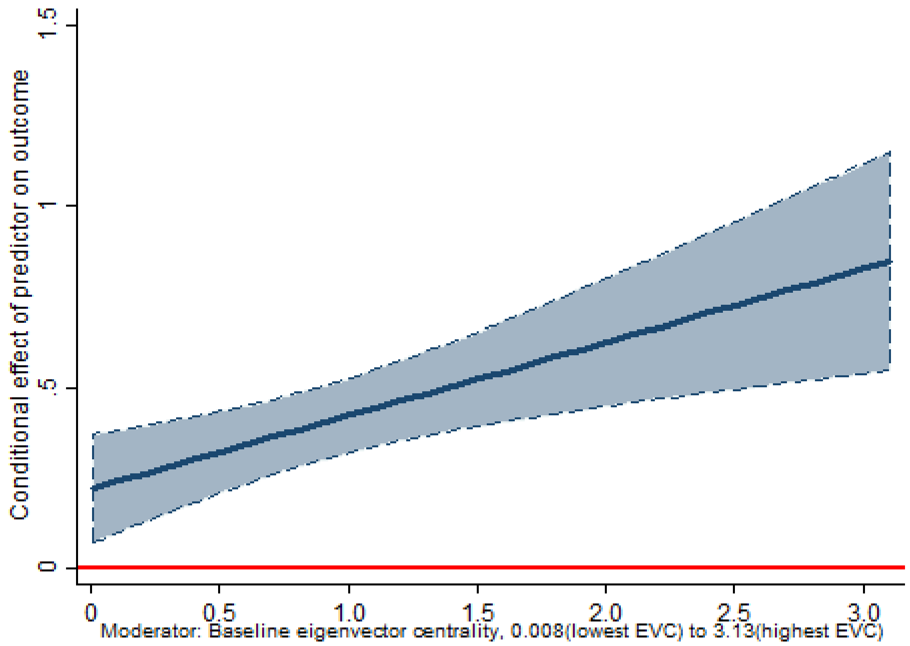

Figure S4.148. The conditional effects of peer influence from average friends' objectively measured smoking behavior at baseline (predictor) on focal participants' objectively measured smoking behavior at follow-up (outcome) by social network eigenvector centralities at baseline (moderator) with 95% CI limits for conditional effects.

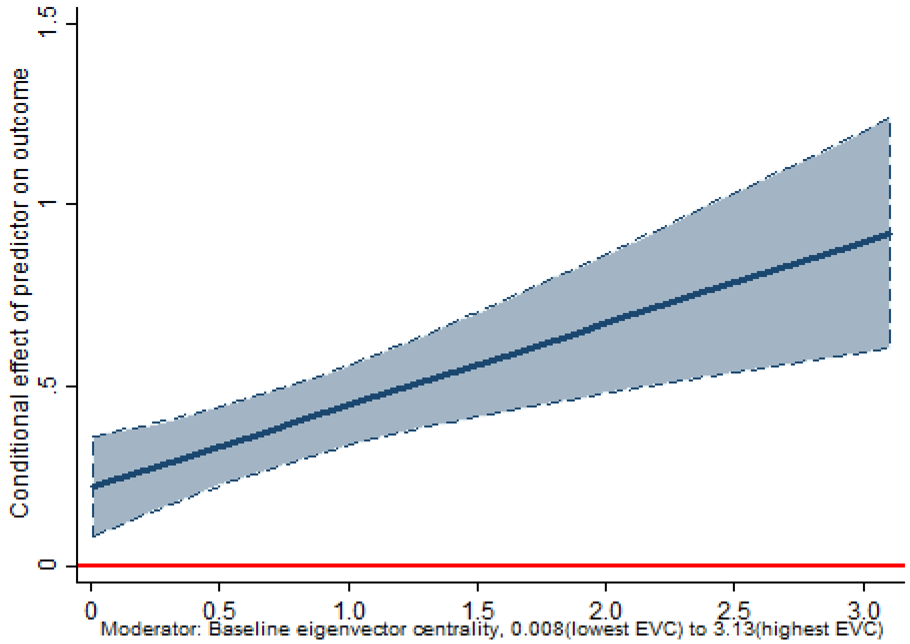

Figure S4.149. The conditional effects of peer influence from average school class objectively measured smoking behavior at baseline (predictor) on focal participants' objectively measured smoking behavior at follow-up (outcome) by social network eigenvector centralities at baseline (moderator) with 95% CI limits for conditional effects.

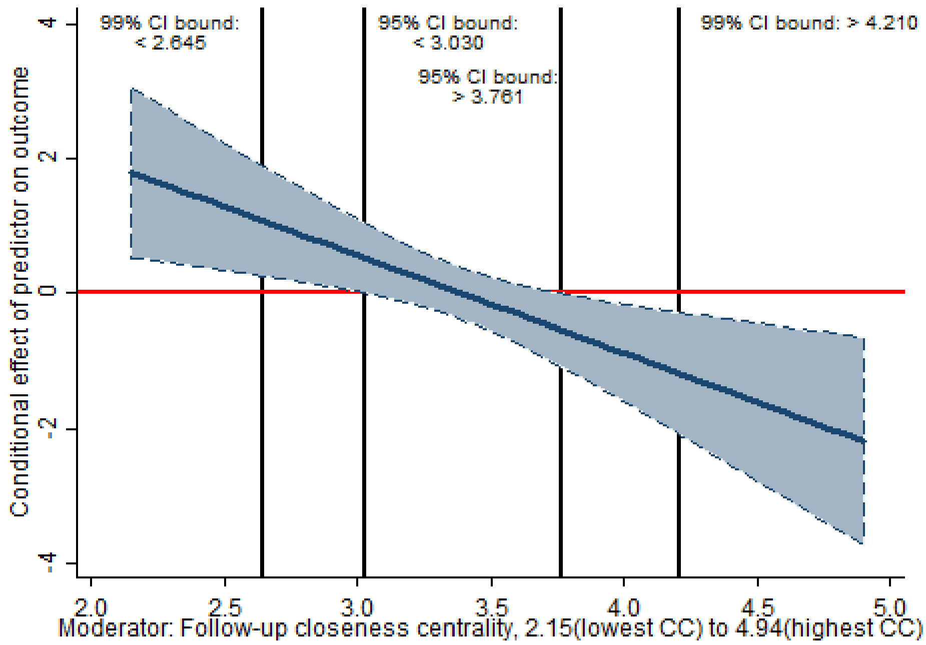

Figure S4.150. The conditional effects of peer influence from average school year group responses to IN5 at follow-up (predictor) on focal participants' values of IN5 at follow-up (outcome) by social network closeness centralities at follow-up (moderator) with 95% CI limits for conditional effects, and bounds indicating regions of significance at the 95% and 99% levels (indicating values of the moderator for which conditional effects differ significantly from 0).

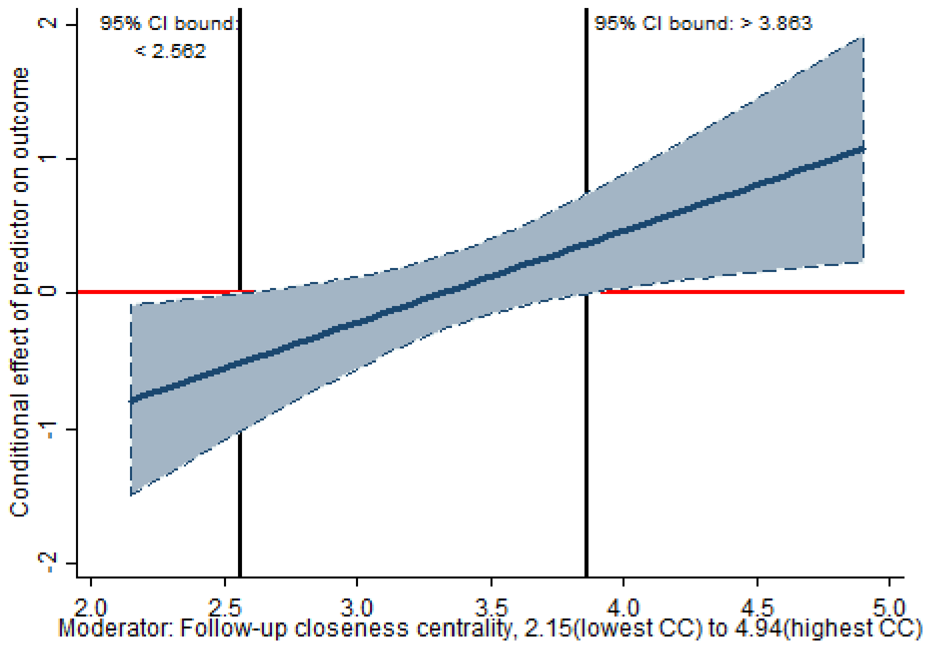

Figure S4.151. The conditional effects of peer influence from average school year group responses to DN1.3 at follow-up (predictor) on focal participants' values of DN1.3 at follow-up (outcome) by social network closeness centralities at follow-up (moderator) with 95% CI limits for conditional effects, and bounds indicating regions of significance at the 95% level (indicating values of the moderator for which conditional effects differ significantly from 0).

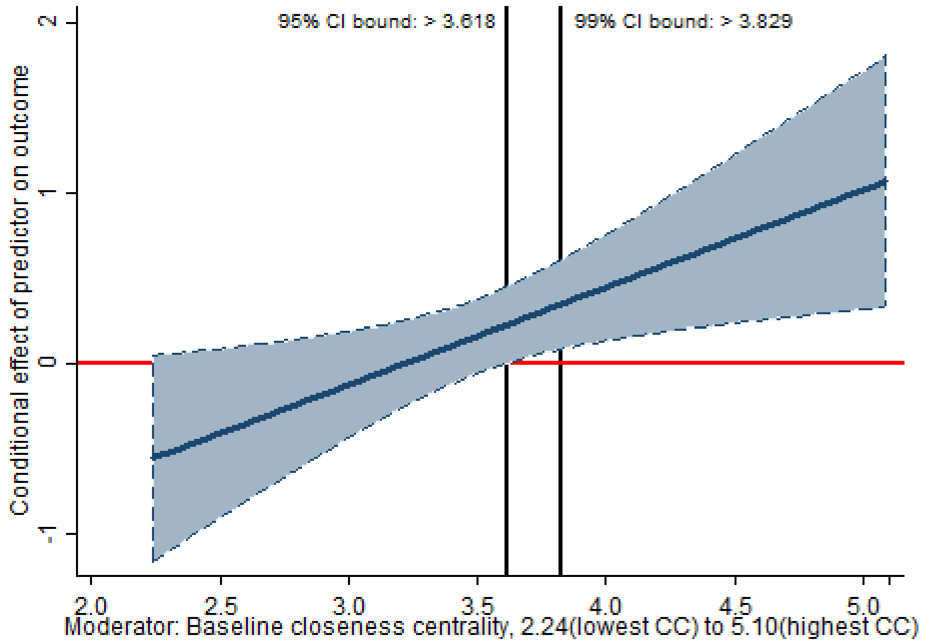

Figure S4.152. The conditional effects of peer influence from average school class self-report smoking behavior at baseline (predictor) on focal participants' self-report smoking behavior at follow-up (outcome) by social network closeness centralities at baseline (moderator) with 95% CI limits for conditional effects, and bounds indicating regions of significance at the 95% and 99% levels (indicating values of the moderator for which conditional effects differ significantly from 0).

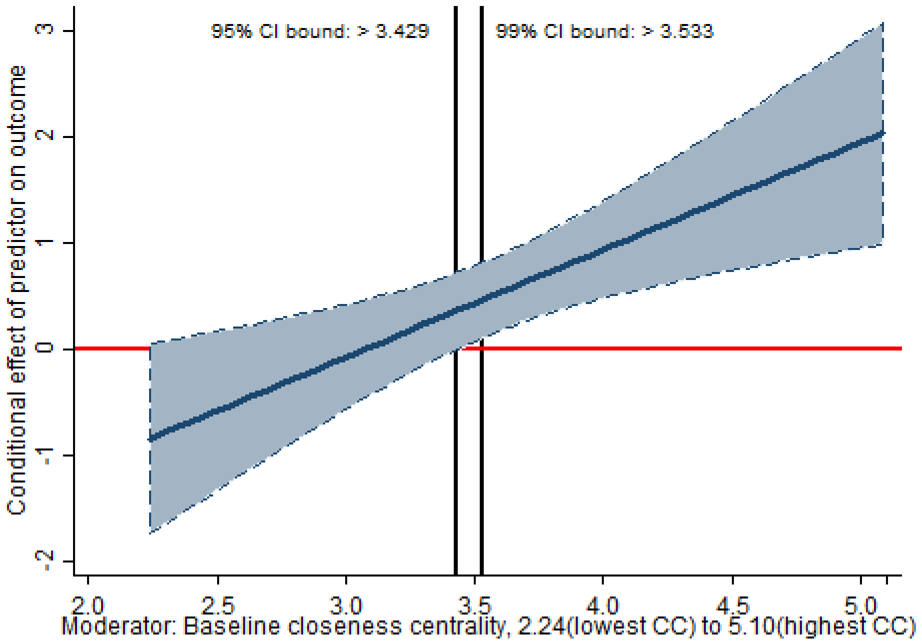

Figure S4.153. The conditional effects of peer influence from average school year group self-report smoking behavior at baseline (predictor) on focal participants' self-report smoking behavior at follow-up (outcome) by social network closeness centralities at baseline (moderator) with 95% CI limits for conditional effects, and bounds indicating regions of significance at the 95% and 99% levels (indicating values of the moderator for which conditional effects differ significantly from 0).

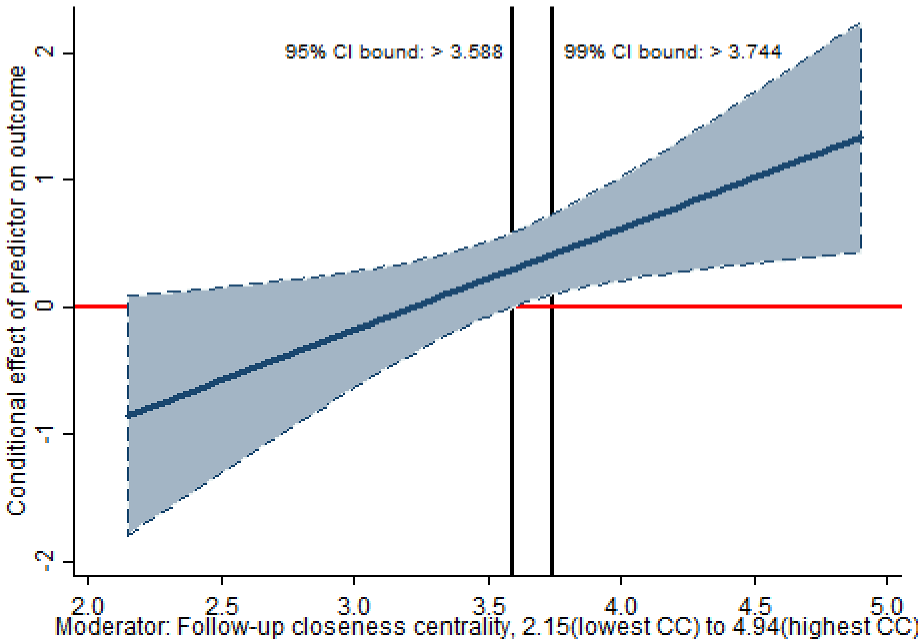

Figure S4.154. The conditional effects of peer influence from average school year group intentions at follow-up (predictor) on focal participants' intentions at follow-up (outcome) by social network closeness centralities at follow-up (moderator) with 95% CI limits for conditional effects, and bounds indicating regions of significance at the 95% and 99% levels (indicating values of the moderator for which conditional effects differ significantly from 0).

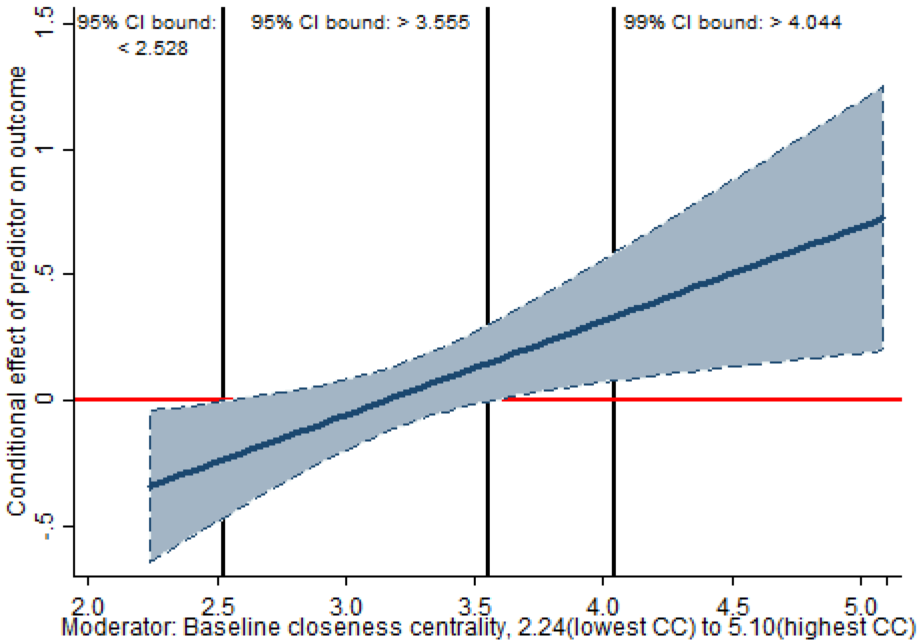

Figure S4.155. The conditional effects of peer influence from average friends' self-efficacy (emotional) at baseline (predictor) on focal participants' self-efficacy (emotional) at follow-up (outcome) by social network closeness centralities at baseline (moderator) with 95% CI limits for conditional effects, and bounds indicating regions of significance at the 95% and 99% levels (indicating values of the moderator for which conditional effects differ significantly from 0).

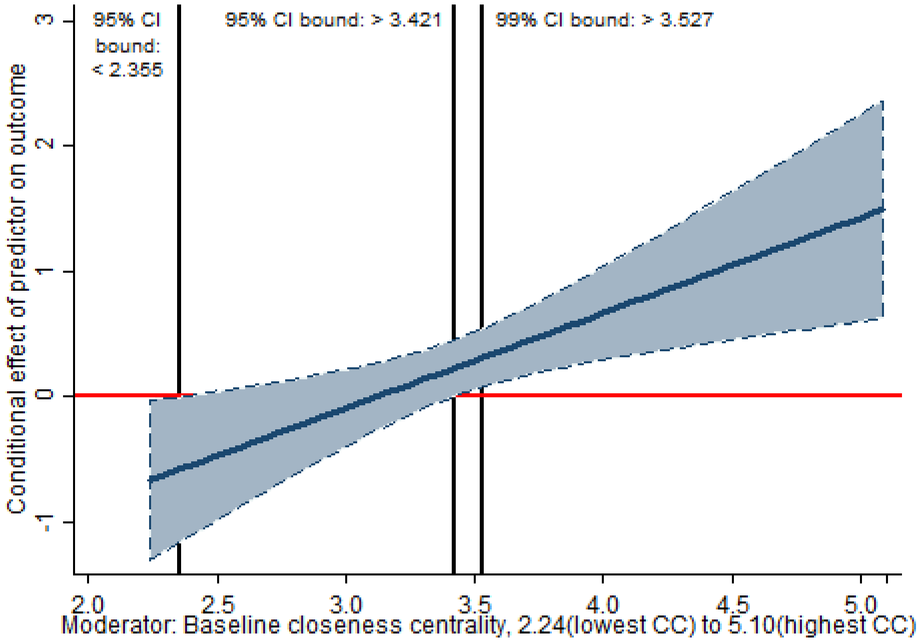

Figure S4.156. The conditional effects of peer influence from average school class self-efficacy (emotional) at baseline (predictor) on focal participants' self-efficacy (emotional) at follow-up (outcome) by social network closeness centralities at baseline (moderator) with 95% CI limits for conditional effects, and bounds indicating regions of significance at the 95% and 99% levels (indicating values of the moderator for which conditional effects differ significantly from 0).

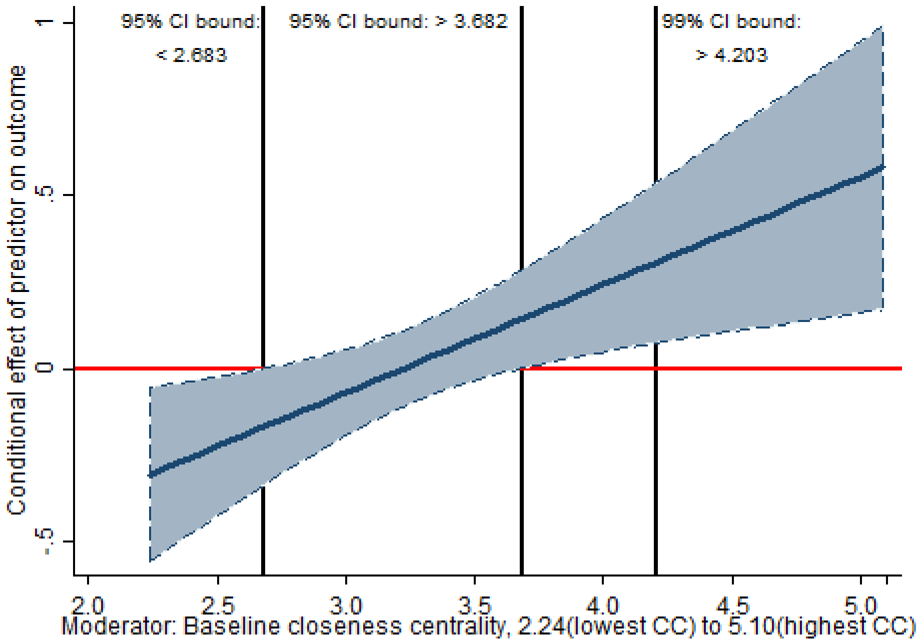

Figure S4.157. The conditional effects of peer influence from average friends' self-efficacy (friends) at baseline (predictor) on focal participants' self-efficacy (friends) at follow-up (outcome) by social network closeness centralities at baseline (moderator) with 95% CI limits for conditional effects, and bounds indicating regions of significance at the 95% and 99% levels (indicating values of the moderator for which conditional effects differ significantly from 0).

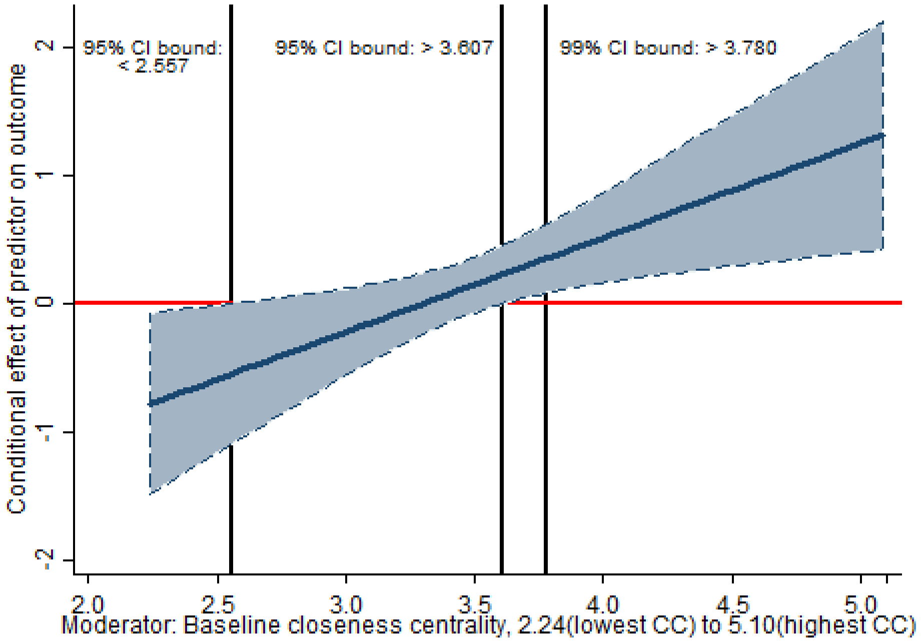

Figure S4.158. The conditional effects of peer influence from average school class self-efficacy (friends) at baseline (predictor) on focal participants' self-efficacy (friends) at follow-up (outcome) by social network closeness centralities at baseline (moderator) with 95% CI limits for conditional effects, and bounds indicating regions of significance at the 95% and 99% levels (indicating values of the moderator for which conditional effects differ significantly from 0).

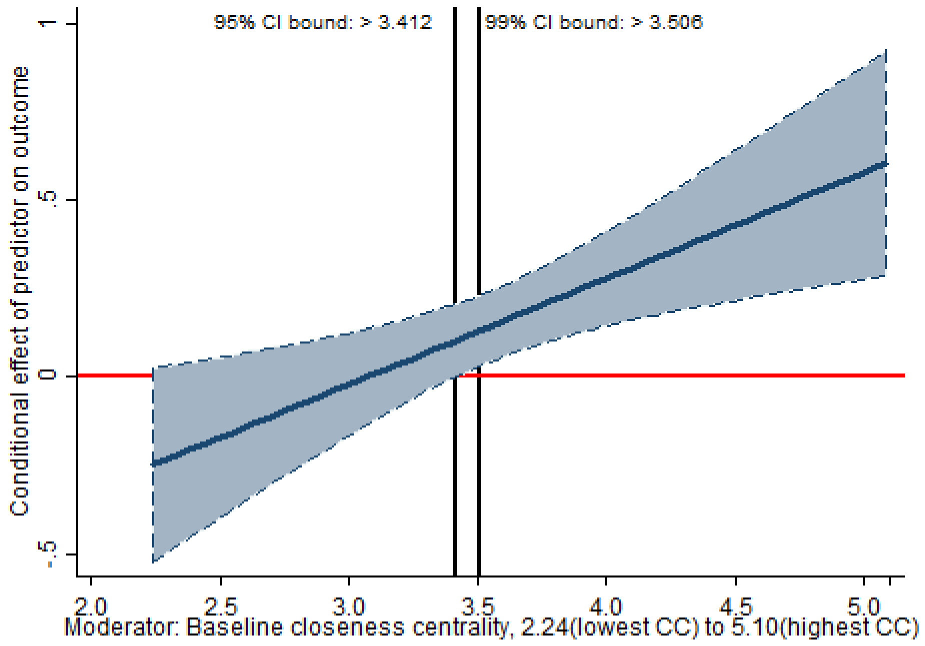

Figure S4.159. The conditional effects of peer influence from average friends' perceived social risks at baseline (predictor) on focal participants' perceived social risks at follow-up (outcome) by social network closeness centralities at baseline (moderator) with 95% CI limits for conditional effects, and bounds indicating regions of significance at the 95% and 99% levels (indicating values of the moderator for which conditional effects differ significantly from 0).

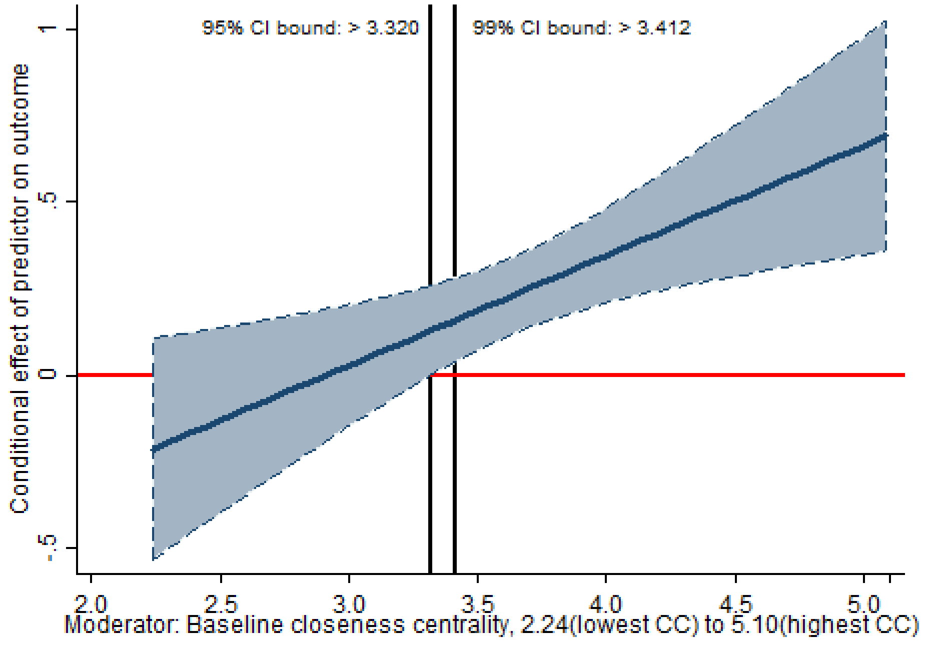

Figure S4.160. The conditional effects of peer influence from average friends' perceived addiction risks at baseline (predictor) on focal participants' perceived addiction risks at follow-up (outcome) by social network closeness centralities at baseline (moderator) with 95% CI limits for conditional effects, and bounds indicating regions of significance at the 95% and 99% levels (indicating values of the moderator for which conditional effects differ significantly from 0).

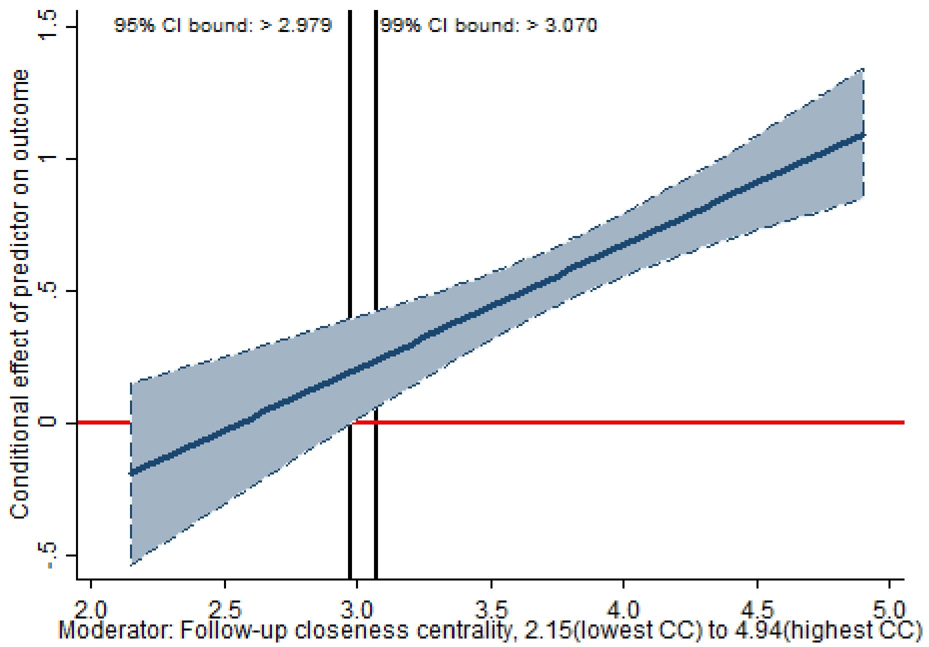

Figure S4.161. The conditional effects of peer influence from average friends' objectively measured smoking behavior at follow-up (predictor) on focal participants' objectively measured smoking behavior at follow-up (outcome) by social network closeness centralities at follow-up (moderator) with 95% CI limits for conditional effects, and bounds indicating regions of significance at the 95% and 99% levels (indicating values of the moderator for which conditional effects differ significantly from 0).

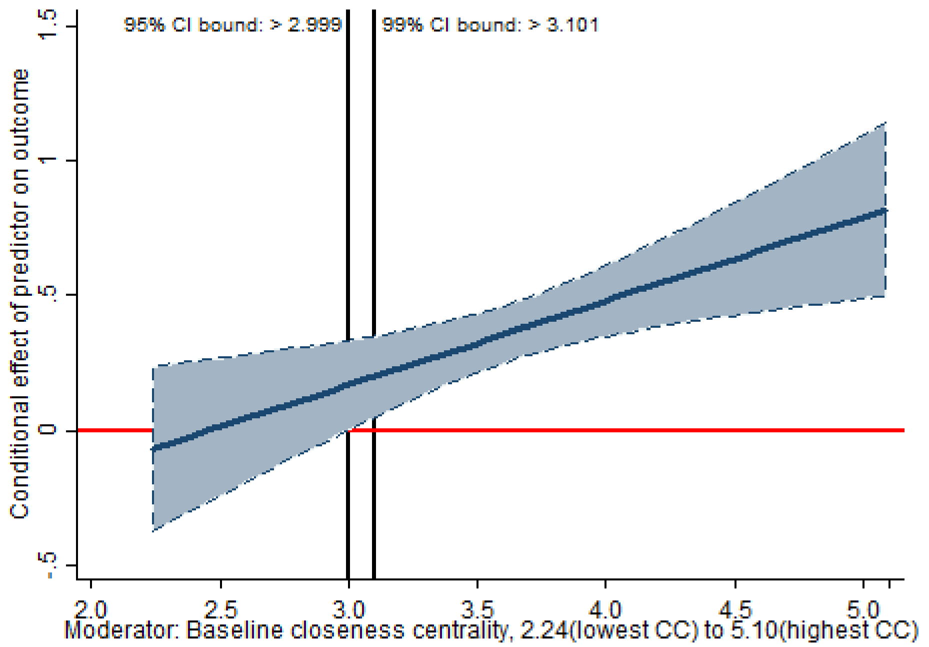

Figure S4.162. The conditional effects of peer influence from average school class objectively measured smoking behavior at baseline (predictor) on focal participants' objectively measured smoking behavior at follow-up (outcome) by social network closeness centralities at baseline (moderator) with 95% CI limits for conditional effects, and bounds indicating regions of significance at the 95% and 99% levels (indicating values of the moderator for which conditional effects differ significantly from 0).

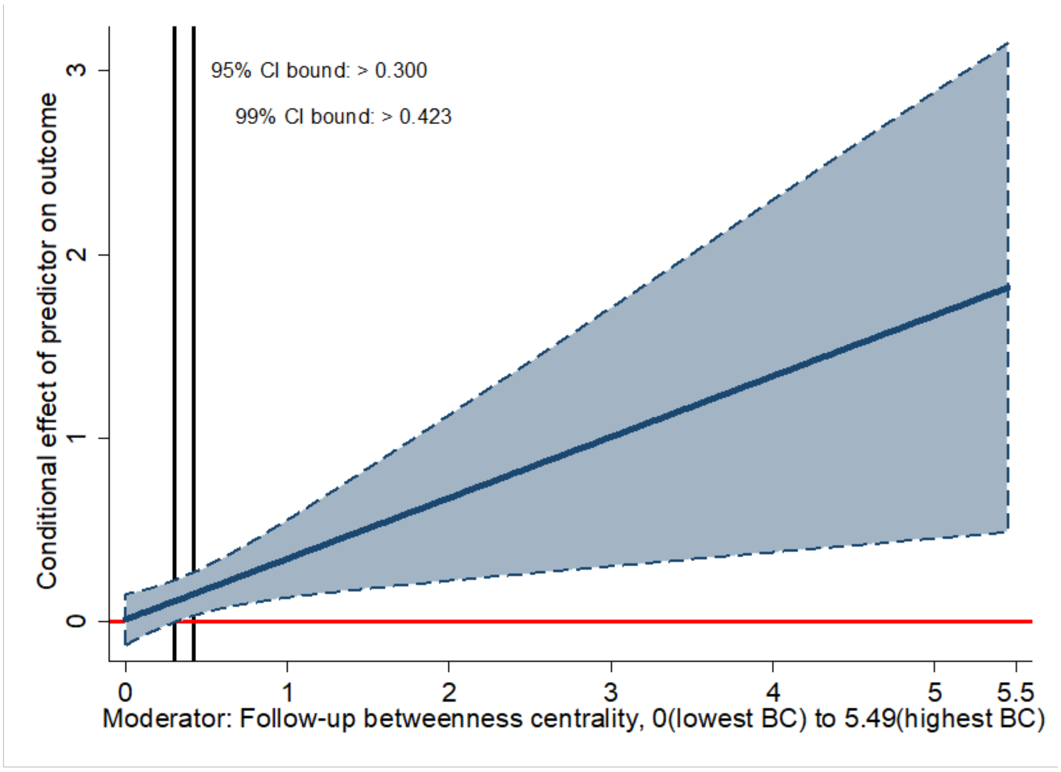

Figure S4.163. The conditional effects of peer influence from average friends' responses to self-report descriptive norms scale 2 (average DN2.1 to DN2.3) at follow-up (predictor) on focal participants' values of descriptive norms scale 2 at follow-up (outcome) by social network betweenness centralities at follow-up (moderator) with 95% CI limits for conditional effects, and bounds indicating regions of significance at the 95% and 99% levels (indicating values of the moderator for which conditional effects differ significantly from 0).

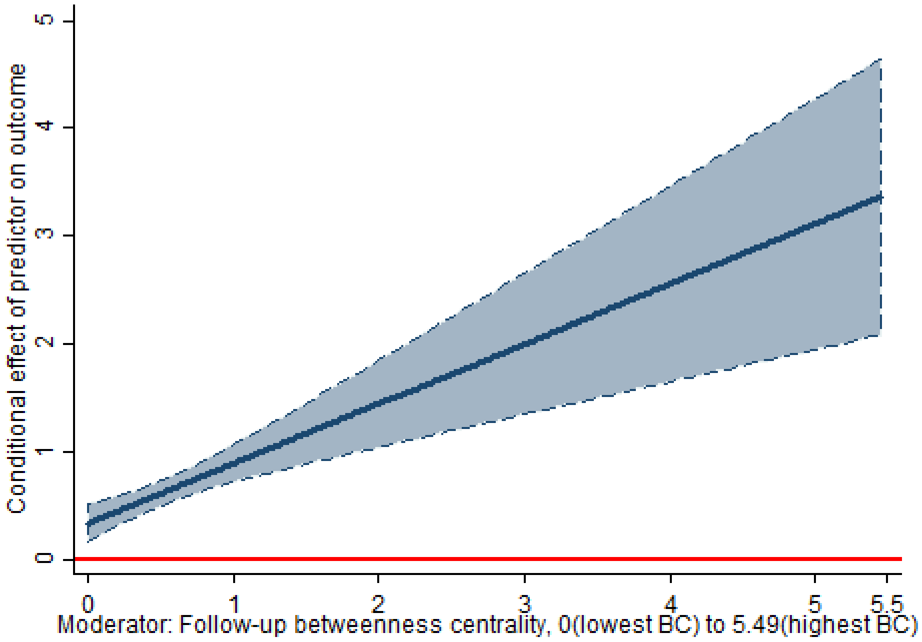

Figure S4.164. The conditional effects of peer influence from average friends' objectively measured smoking behavior at follow-up (predictor) on focal participants' objectively measured smoking behavior at follow-up (outcome) by social network betweenness centralities at follow-up (moderator) with 95% CI limits for conditional effects.

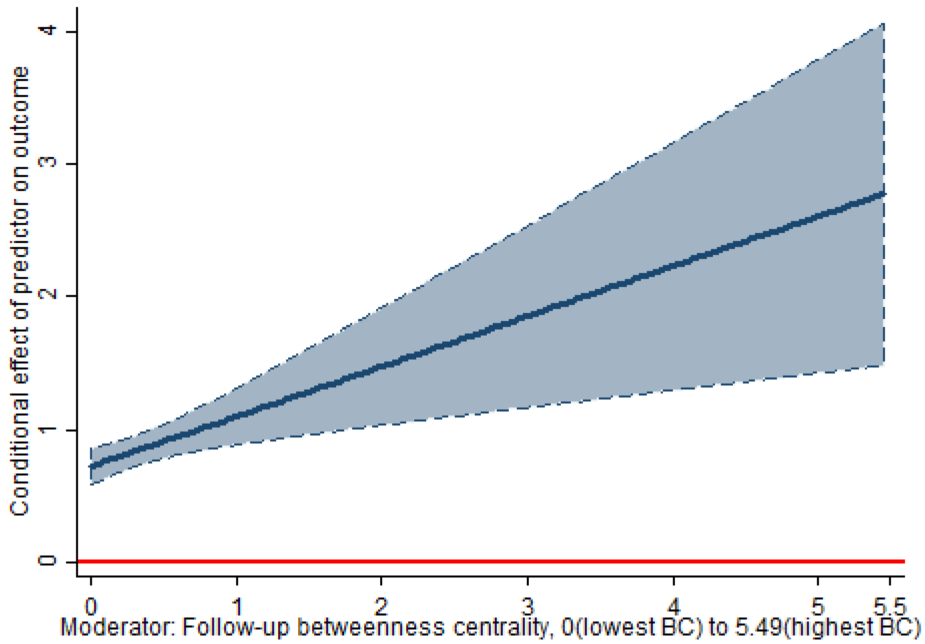

Figure S4.165. The conditional effects of peer influence from average school year group objectively measured smoking behavior at follow-up (predictor) on focal participants' objectively measured smoking behavior at follow-up (outcome) by social network betweenness centralities at follow-up (moderator) with 95% CI limits for conditional effects.

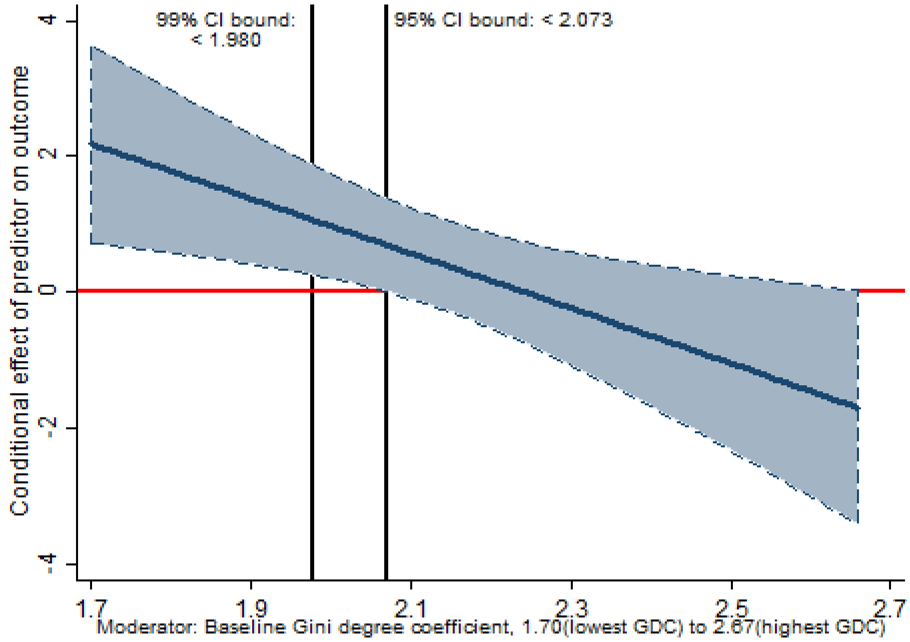

Figure S4.166. The conditional effects of peer influence from average school year group responses for P2S4 at baseline (predictor) on focal participants' values of P2S4 at follow-up (outcome) by social network Gini degree coefficients at baseline (moderator) with 95% CI limits for conditional effects, and bounds indicating regions of significance at the 95% and 99% levels (indicating values of the moderator for which conditional effects differ significantly from 0).

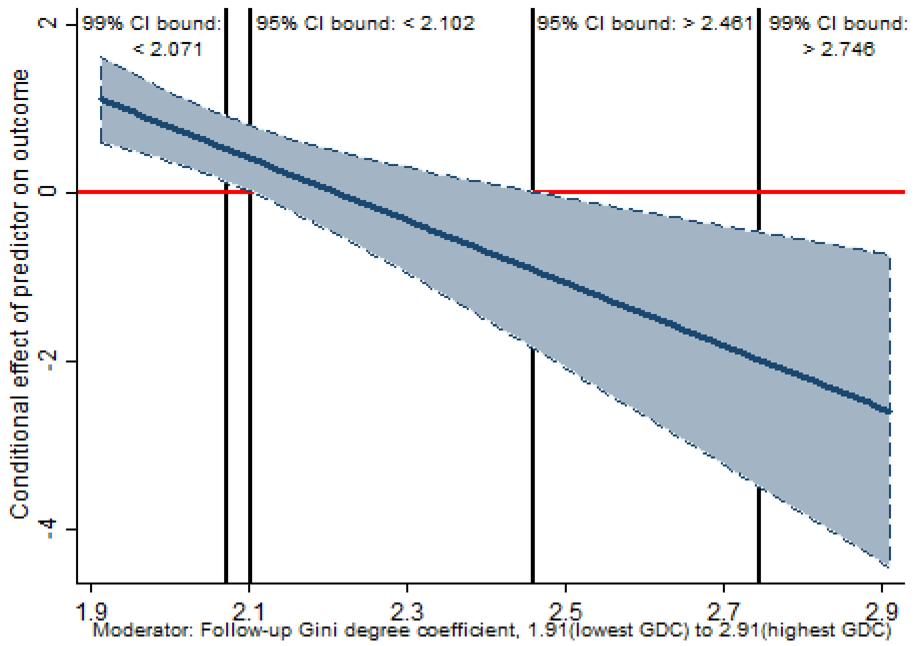

Figure S4.167. The conditional effects of peer influence from average school year group responses for P2S4 at follow-up (predictor) on focal participants' values of P2S4 at follow-up (outcome) by social network Gini degree coefficients at follow-up (moderator) with 95% CI limits for conditional effects, and bounds indicating regions of significance at the 95% and 99% levels (indicating values of the moderator for which conditional effects differ significantly from 0).

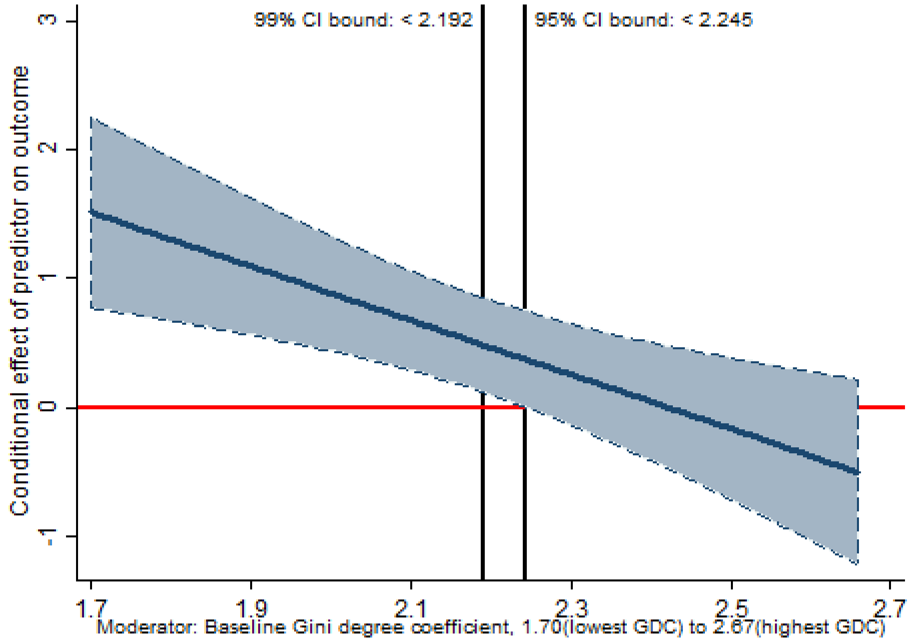

Figure S4.168. The conditional effects of peer influence from average school year group responses for P2S6 at baseline (predictor) on focal participants' values of P2S6 at follow-up (outcome) by social network Gini degree coefficients at baseline (moderator) with 95% CI limits for conditional effects, and bounds indicating regions of significance at the 95% and 99% levels (indicating values of the moderator for which conditional effects differ significantly from 0).

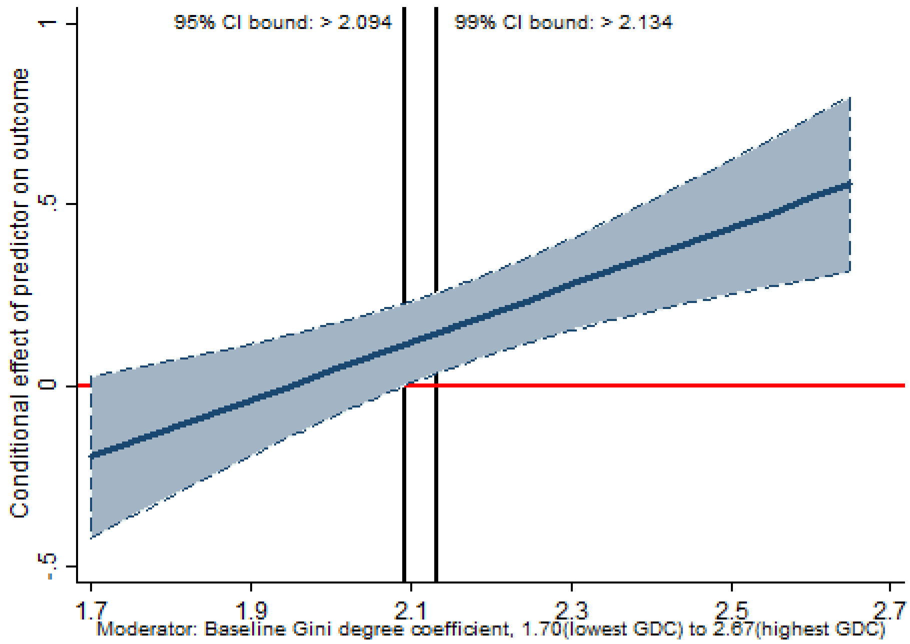

Figure S4.169. The conditional effects of peer influence from average friends' donations to ASSIST/Dead Cool at baseline (predictor) on focal participants' donations to ASSIST/Dead Cool at follow-up (outcome) by social network Gini degree coefficients at baseline (moderator) with 95% CI limits for conditional effects, and bounds indicating regions of significance at the 95% and 99% levels (indicating values of the moderator for which conditional effects differ significantly from 0).

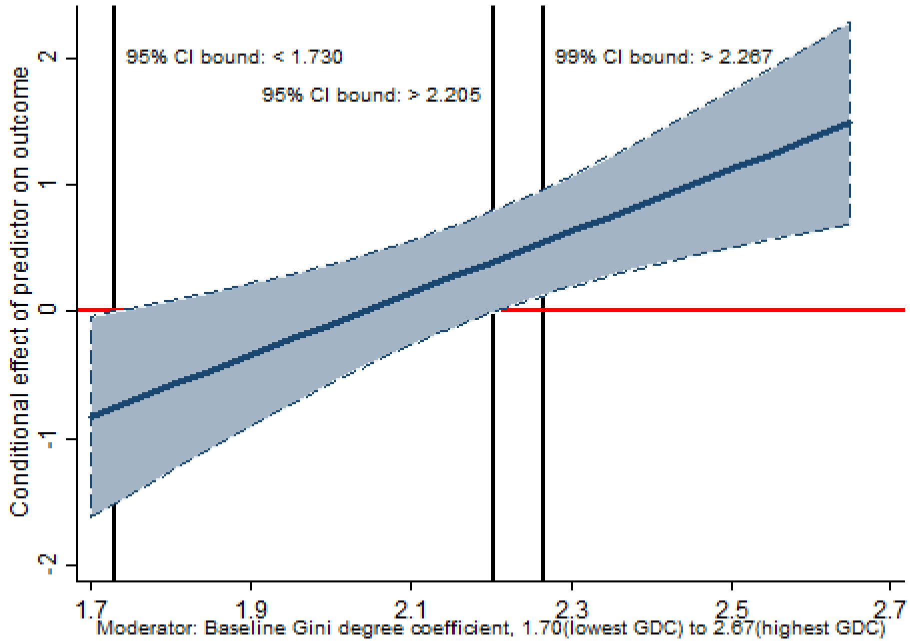

Figure S4.170. The conditional effects of peer influence from average school year group donations to ASSIST/Dead Cool at baseline (predictor) on focal participants' donations to ASSIST/Dead Cool at follow-up (outcome) by social network Gini degree coefficients at baseline (moderator) with 95% CI limits for conditional effects, and bounds indicating regions of significance at the 95% and 99% levels (indicating values of the moderator for which conditional effects differ significantly from 0).

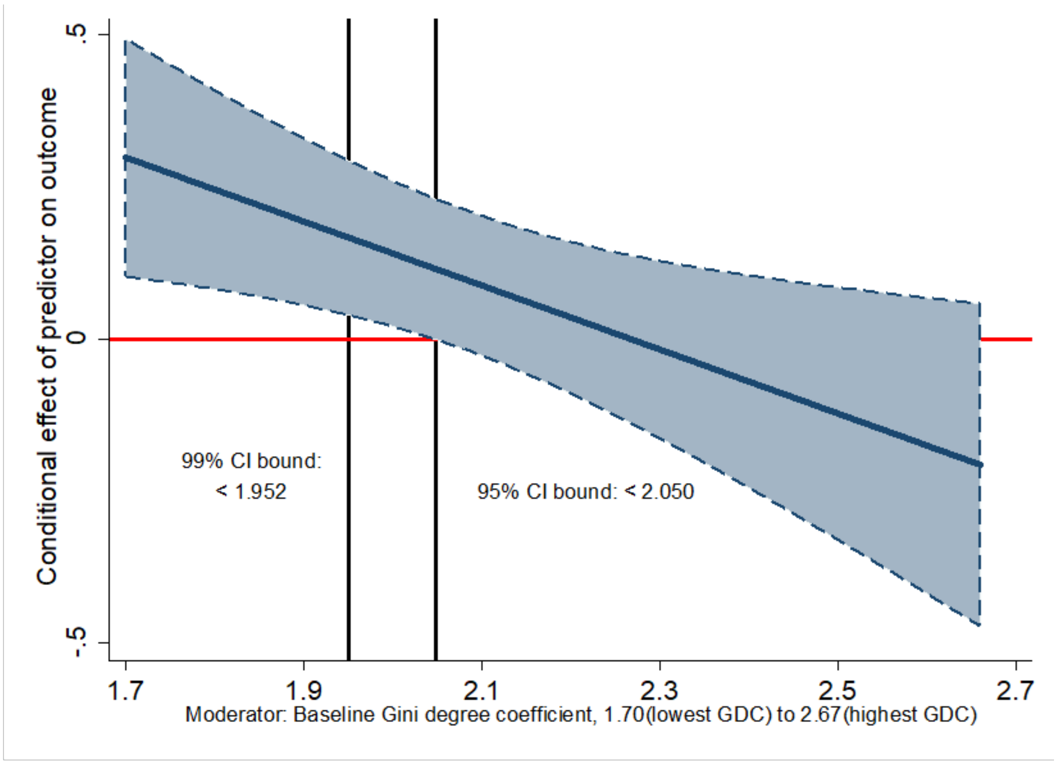

Figure S4.171. The conditional effects of peer influence from average friends' responses to self-report injunctive norms (average IN1 to IN7) at baseline (predictor) on focal participants' values of self-report injunctive norms at follow-up (outcome) by social network Gini degree coefficients at baseline (moderator) with 95% CI limits for conditional effects, and bounds indicating regions of significance at the 95% and 99% levels (indicating values of the moderator for which conditional effects differ significantly from 0).

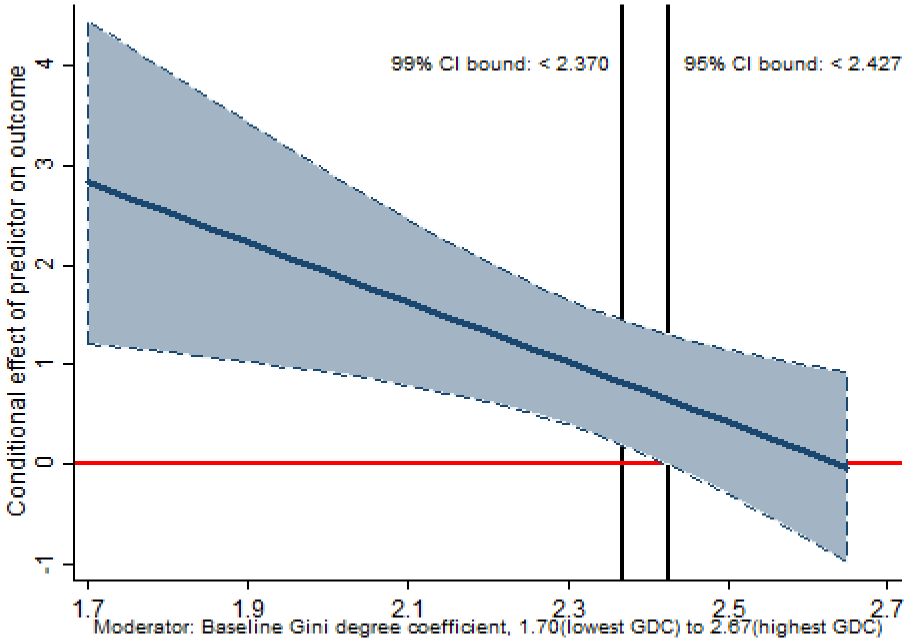

Figure S4.172. The conditional effects of peer influence from average school year group responses to DN1.1 at baseline (predictor) on focal participants' values of DN1.1 at follow-up (outcome) by social network Gini degree coefficients at baseline (moderator) with 95% CI limits for conditional effects, and bounds indicating regions of significance at the 95% and 99% levels (indicating values of the moderator for which conditional effects differ significantly from 0).

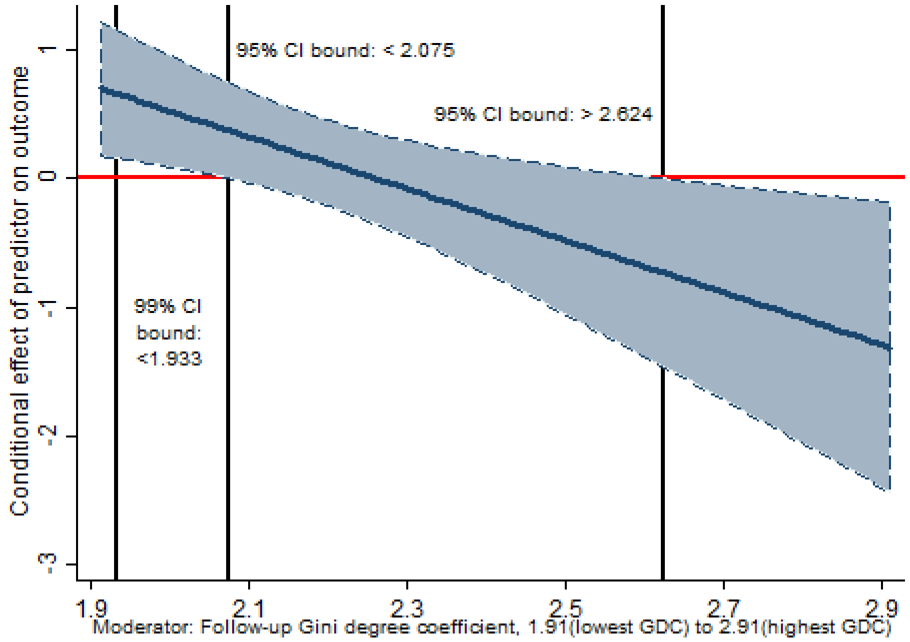

Figure S4.173. The conditional effects of peer influence from average school year group responses to DN2.1 at follow-up (predictor) on focal participants' values of DN2.1 at follow-up (outcome) by social network Gini degree coefficients at follow-up (moderator) with 95% CI limits for conditional effects, and bounds indicating regions of significance at the 95% and 99% levels (indicating values of the moderator for which conditional effects differ significantly from 0).

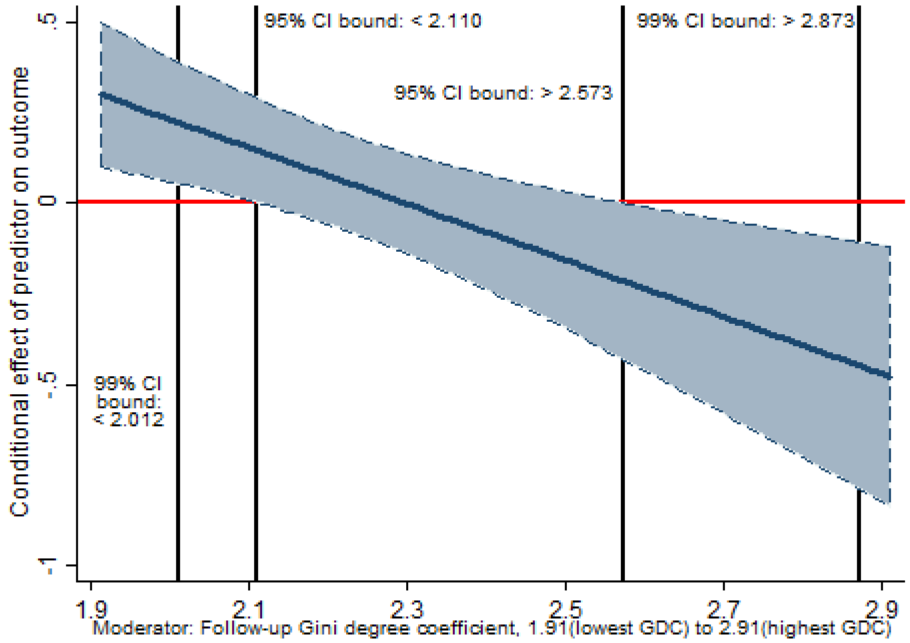

Figure S4.174. The conditional effects of peer influence from average friends' perceived behavioral control (easy to avoid) at follow-up (predictor) on focal participants' perceived behavioral control (easy to avoid) at follow-up (outcome) by social network Gini degree coefficients at follow-up (moderator) with 95% CI limits for conditional effects, and bounds indicating regions of significance at the 95% and 99% levels (indicating values of the moderator for which conditional effects differ significantly from 0).

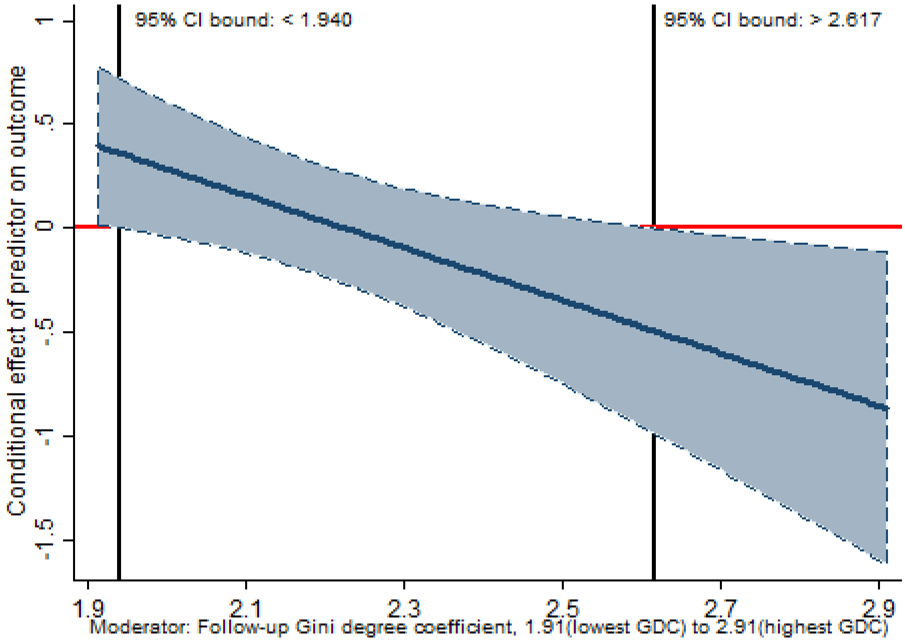

Figure S4.175. The conditional effects of peer influence from average school class perceived behavioral control (easy to avoid) at follow-up (predictor) on focal participants' perceived behavioral control (easy to avoid) at follow-up (outcome) by social network Gini degree coefficients at follow-up (moderator) with 95% CI limits for conditional effects, and bounds indicating regions of significance at the 95% and 99% levels (indicating values of the moderator for which conditional effects differ significantly from 0).

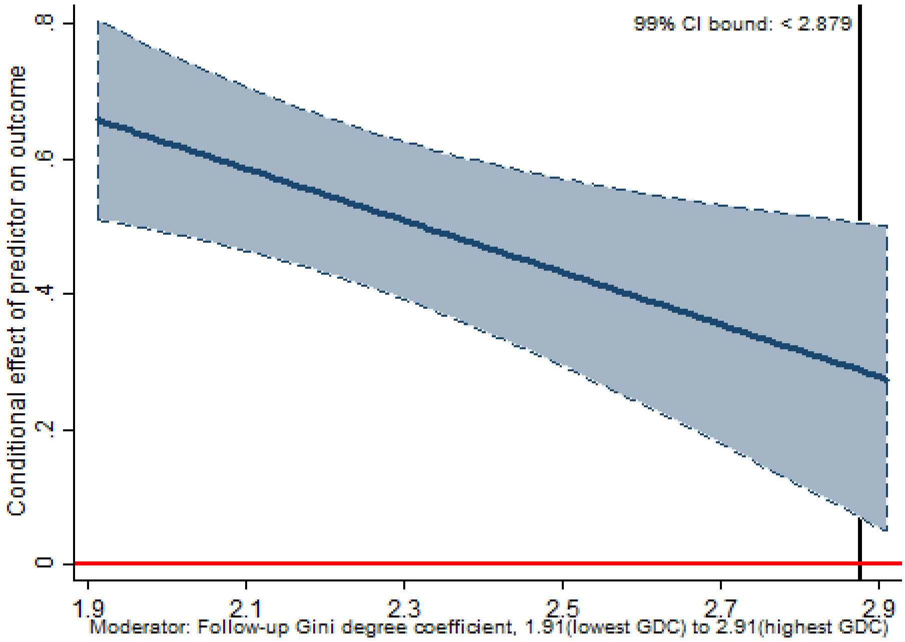

Figure S4.176. The conditional effects of peer influence from average friends' objectively measured smoking behavior at follow-up (predictor) on focal participants' objectively measured smoking behavior at follow-up (outcome) by social network Gini degree coefficients at follow-up (moderator) with 95% CI limits for conditional effects, and bounds indicating regions of significance at the 99% level (indicating values of the moderator for which conditional effects differ significantly from 0).

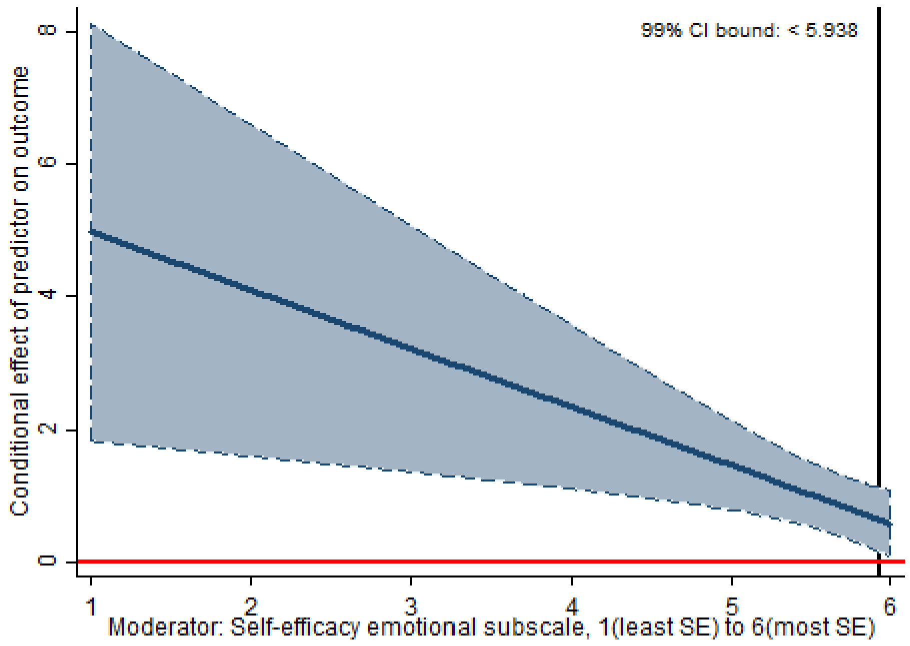

Figure S4.177. The conditional effects of peer influence from average school year group intentions at baseline (predictor) on focal participants' intentions at follow-up (outcome) by self-efficacy emotional subscale (moderator) with 95% CI limits for conditional effects, and bounds indicating regions of significance at the 99% level (indicating values of the moderator for which conditional effects differ significantly from 0).

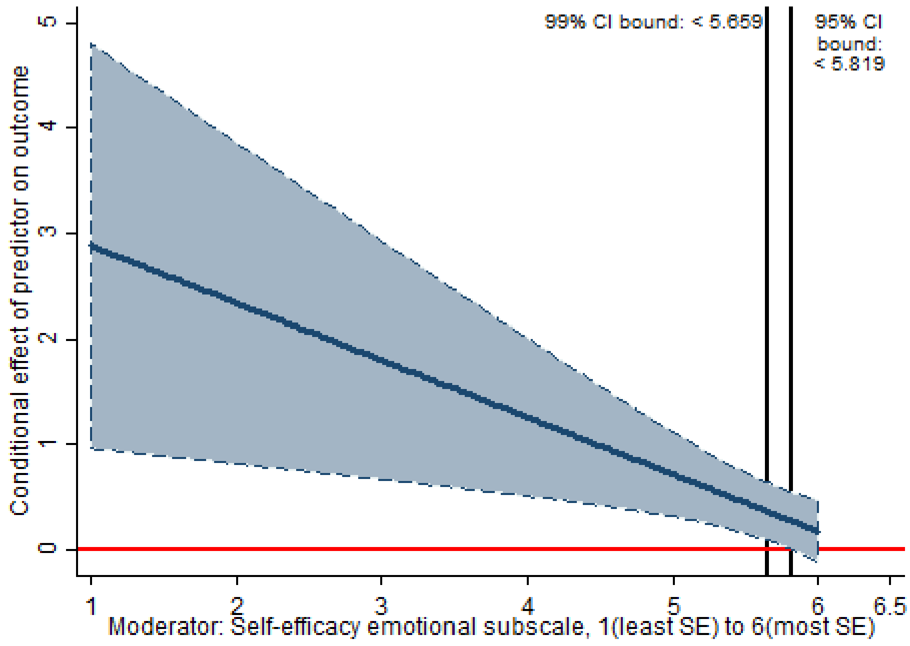

Figure S4.178. The conditional effects of peer influence from average school year group intentions at follow-up (predictor) on focal participants' intentions at follow-up (outcome) by self-efficacy emotional subscale (moderator) with 95% CI limits for conditional effects, and bounds indicating regions of significance at the 95% and 99% levels (indicating values of the moderator for which conditional effects differ significantly from 0).

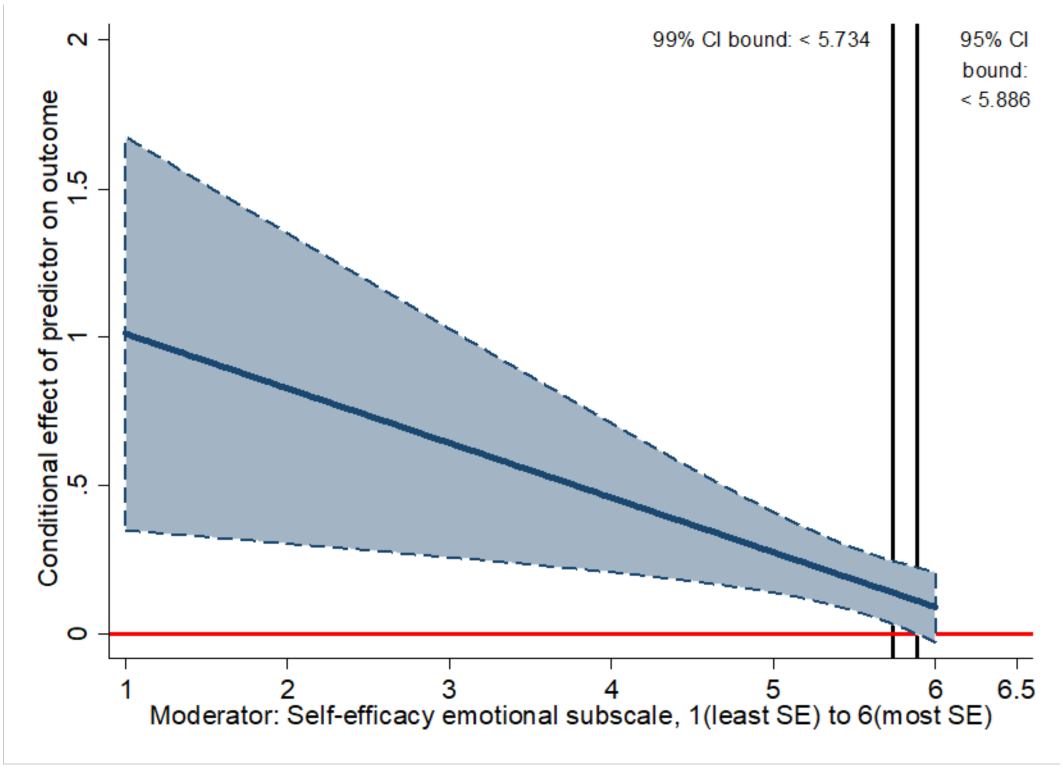

Figure S4.179. The conditional effects of peer influence from average friends' responses to self-report injunctive norms (average IN1 to IN7) at follow-up (predictor) on focal participants' values of self-report injunctive norms at follow-up (outcome) by self-efficacy emotional subscale (moderator) with 95% CI limits for conditional effects, and bounds indicating regions of significance at the 95% and 99% levels (indicating values of the moderator for which conditional effects differ significantly from 0).

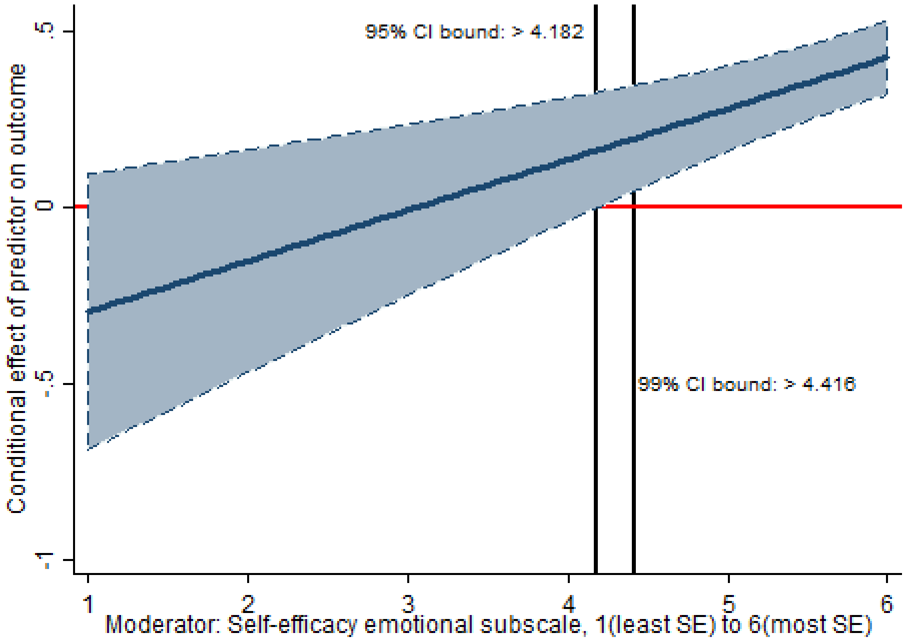

Figure S4.180. The conditional effects of peer influence from average school class objectively measured smoking behavior at baseline (predictor) on focal participants' objectively measured smoking behavior at follow-up (outcome) by self-efficacy emotional subscale (moderator) with 95% CI limits for conditional effects, and bounds indicating regions of significance at the 95% and 99% levels (indicating values of the moderator for which conditional effects differ significantly from 0).

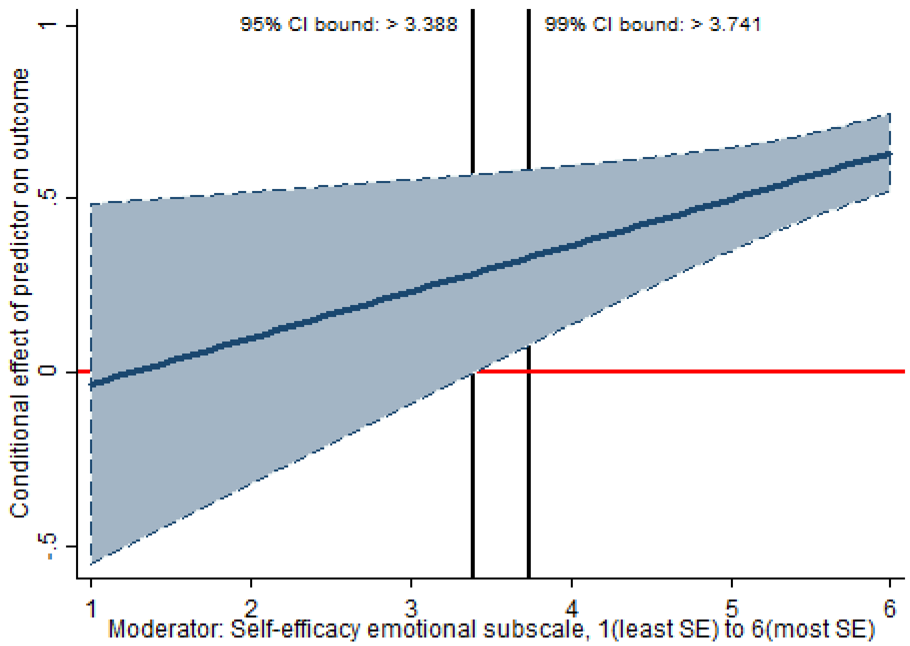

Figure S4.181. The conditional effects of peer influence from average school year group objectively measured smoking behavior at baseline (predictor) on focal participants' objectively measured smoking behavior at follow-up (outcome) by self-efficacy emotional subscale (moderator) with 95% CI limits for conditional effects, and bounds indicating regions of significance at the 95% and 99% levels (indicating values of the moderator for which conditional effects differ significantly from 0).

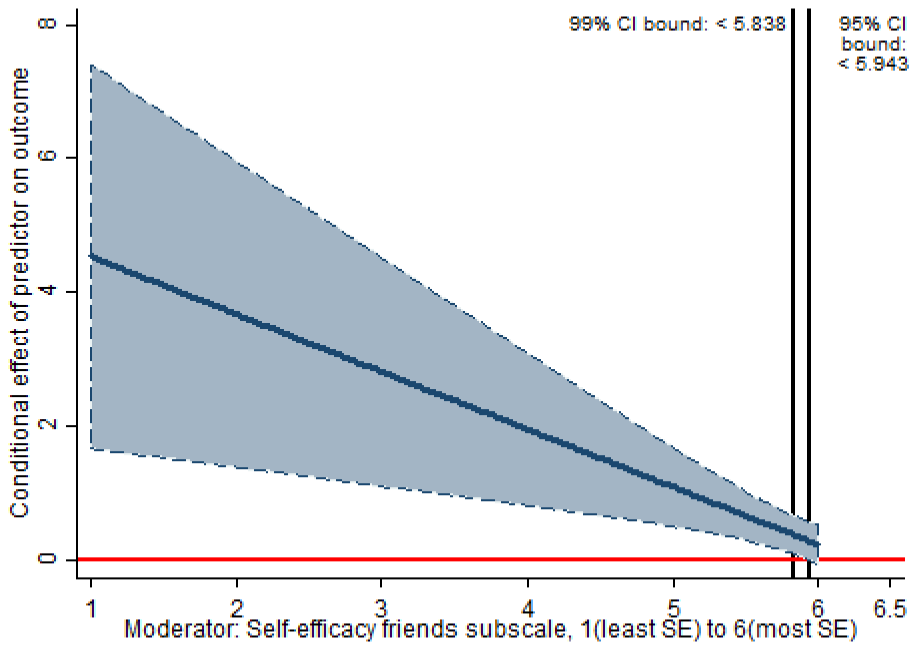

Figure S4.182. The conditional effects of peer influence from average school year group self-report smoking behavior at baseline (predictor) on focal participants' self-report smoking behavior at follow-up (outcome) by self-efficacy friends subscale (moderator) with 95% CI limits for conditional effects, and bounds indicating regions of significance at the 95% and 99% levels (indicating values of the moderator for which conditional effects differ significantly from 0).

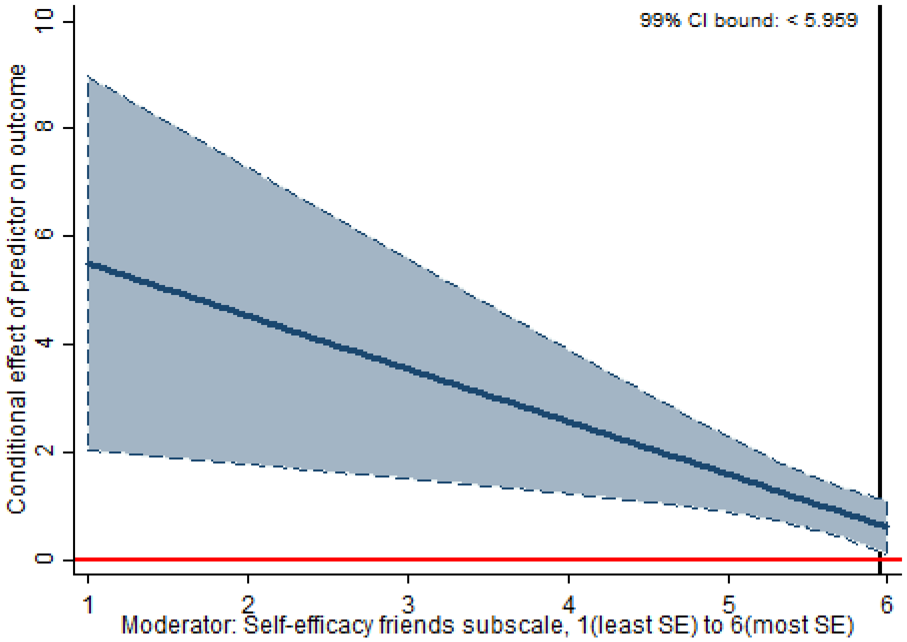

Figure S4.183. The conditional effects of peer influence from average school year group intentions at baseline (predictor) on focal participants' intentions at follow-up (outcome) by self-efficacy friends subscale (moderator) with 95% CI limits for conditional effects, and bounds indicating regions of significance at the 99% level (indicating values of the moderator for which conditional effects differ significantly from 0).

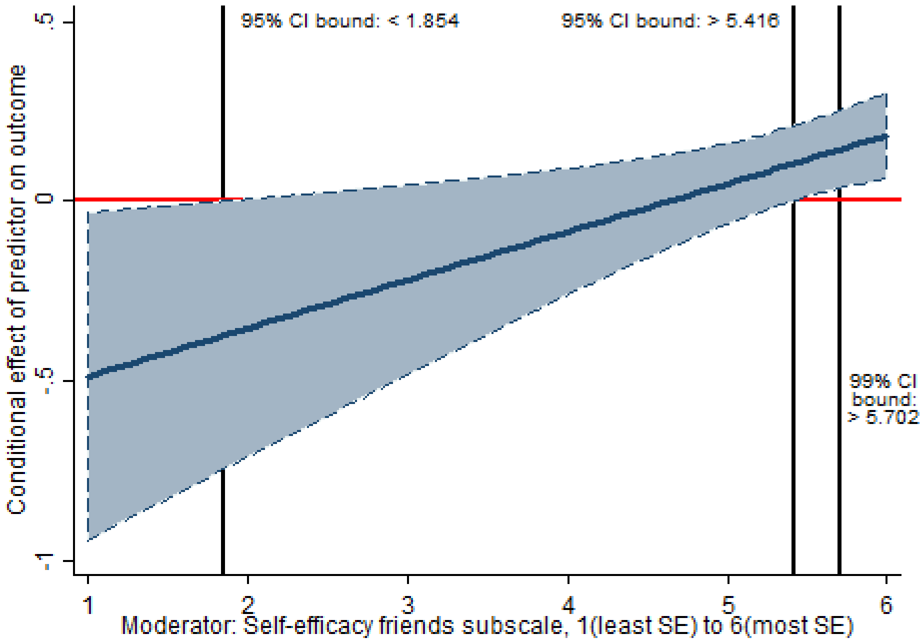

Figure S4.184. The conditional effects of peer influence from average friends' perceived physical risks at follow-up (predictor) on focal participants' perceived physical risks at follow-up (outcome) by self-efficacy friends subscale (moderator) with 95% CI limits for conditional effects, and bounds indicating regions of significance at the 95% and 99% levels (indicating values of the moderator for which conditional effects differ significantly from 0).

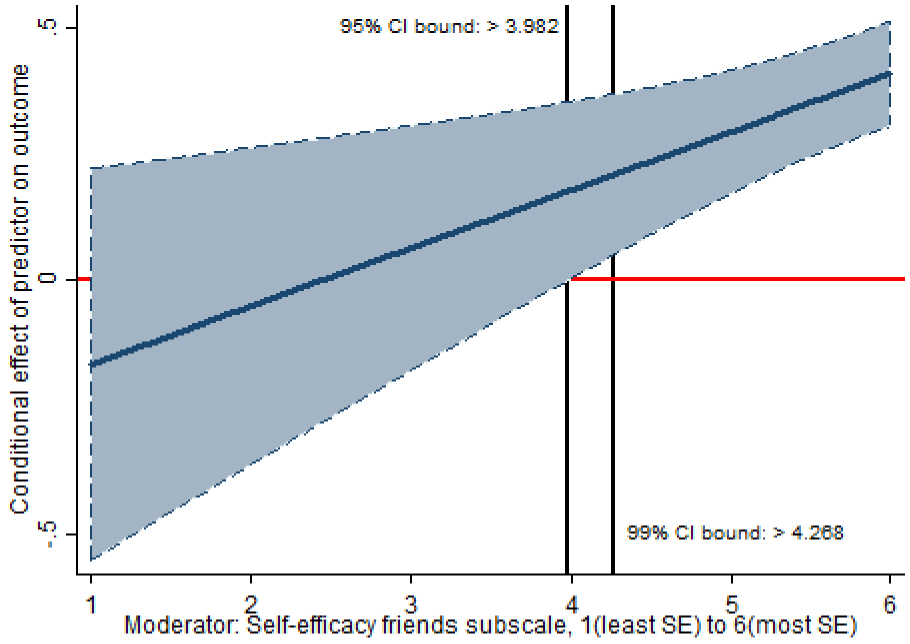

Figure S4.185. The conditional effects of peer influence from average friends' objectively measured smoking behavior at baseline (predictor) on focal participants' objectively measured smoking behavior at follow-up (outcome) by self-efficacy friends subscale (moderator) with 95% CI limits for conditional effects, and bounds indicating regions of significance at the 95% and 99% levels (indicating values of the moderator for which conditional effects differ significantly from 0).

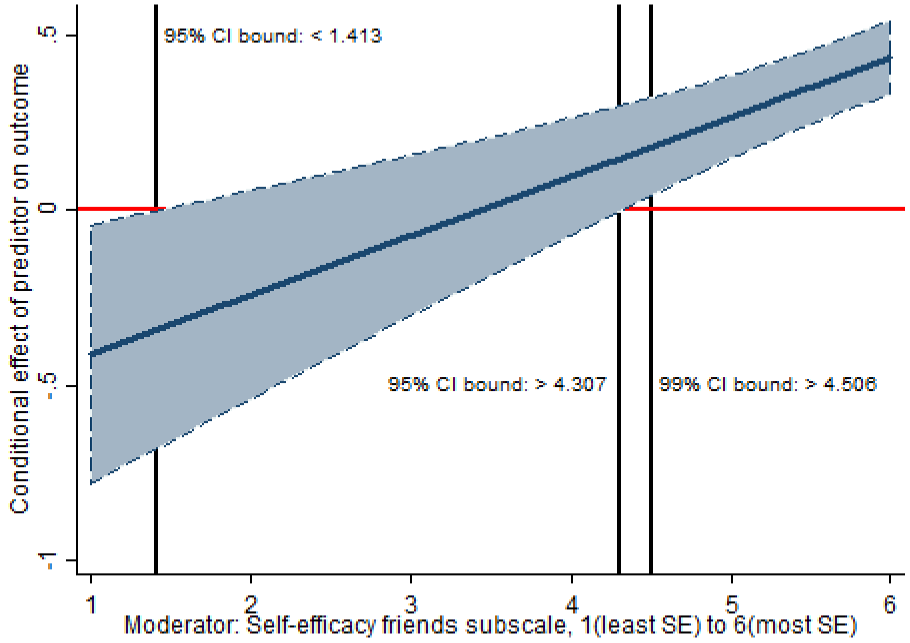

Figure S4.186. The conditional effects of peer influence from average school class objectively measured smoking behavior at baseline (predictor) on focal participants' objectively measured smoking behavior at follow-up (outcome) by self-efficacy friends subscale (moderator) with 95% CI limits for conditional effects, and bounds indicating regions of significance at the 95% and 99% levels (indicating values of the moderator for which conditional effects differ significantly from 0).

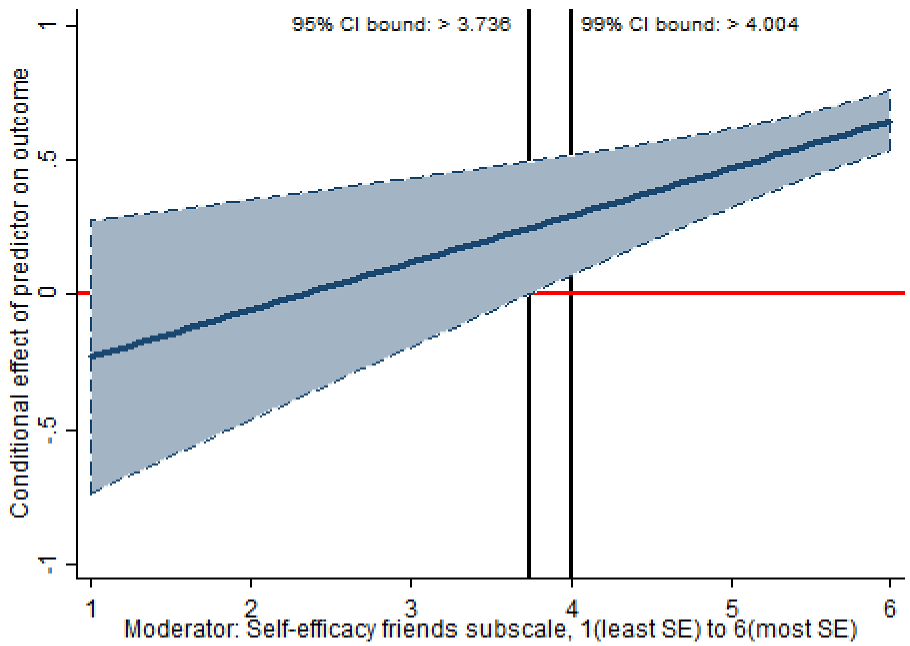

Figure S4.187. The conditional effects of peer influence from average school year group objectively measured smoking behavior at baseline (predictor) on focal participants' objectively measured smoking behavior at follow-up (outcome) by self-efficacy friends subscale (moderator) with 95% CI limits for conditional effects, and bounds indicating regions of significance at the 95% and 99% levels (indicating values of the moderator for which conditional effects differ significantly from 0).

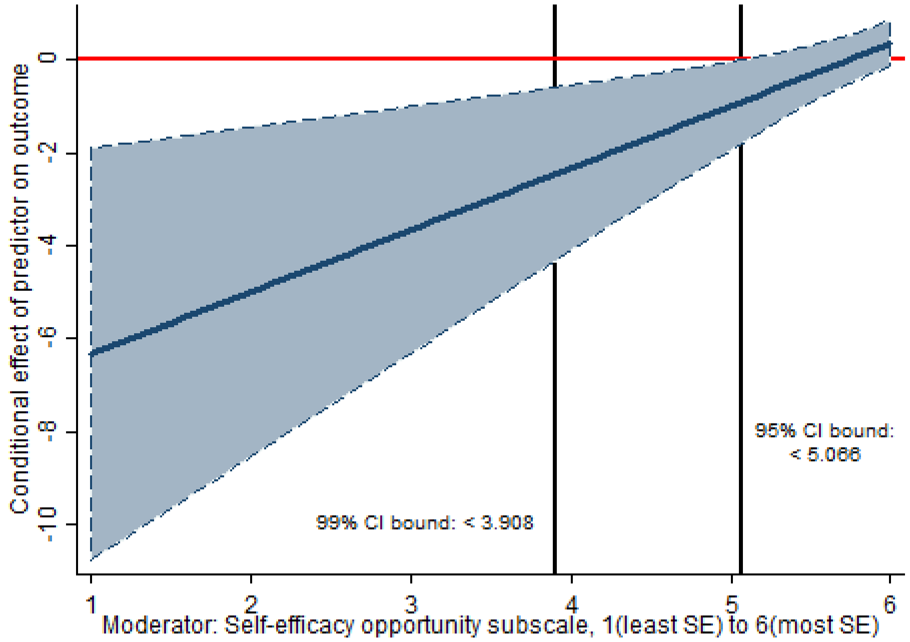

Figure S4.188. The conditional effects of peer influence from average school year group responses for P2S3 at follow-up (predictor) on focal participants' values of P2S3 at follow-up (outcome) by self-efficacy opportunity subscale (moderator) with 95% CI limits for conditional effects, and bounds indicating regions of significance at the 95% and 99% levels (indicating values of the moderator for which conditional effects differ significantly from 0).

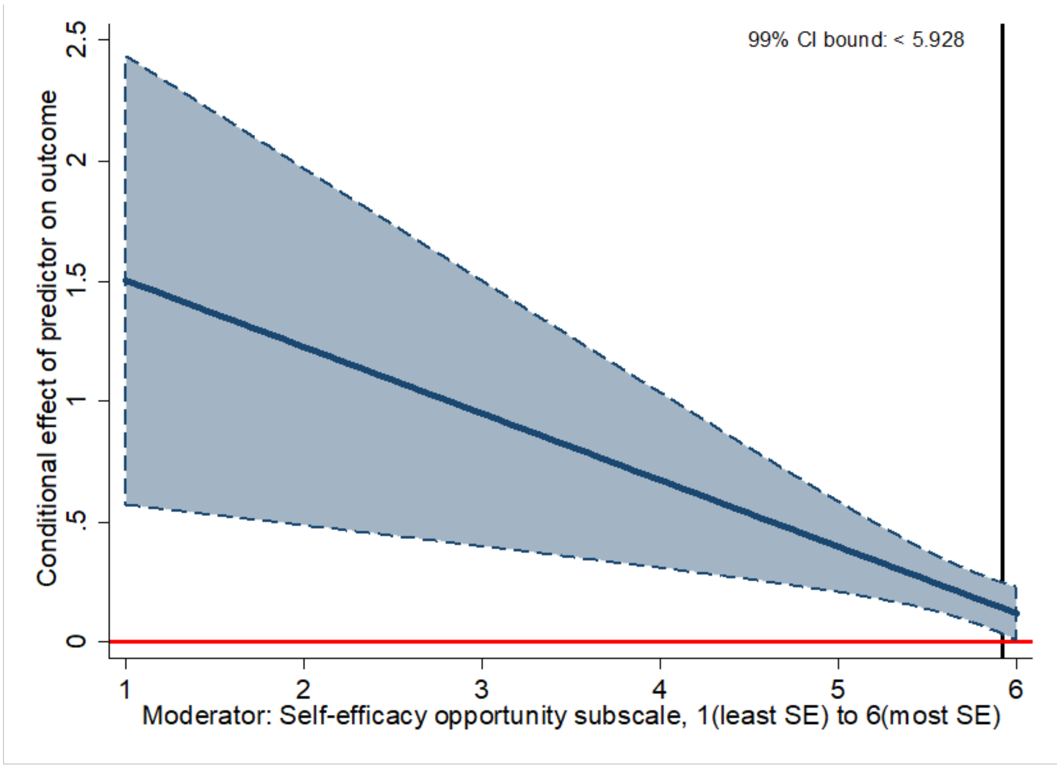

Figure S4.189. The conditional effects of peer influence from average friends' responses to self-report injunctive norms (average IN1 to IN7) at follow-up (predictor) on focal participants' values of self-report injunctive norms at follow-up (outcome) by self-efficacy opportunity subscale (moderator) with 95% CI limits for conditional effects, and bounds indicating regions of significance at the 95% and 99% levels (indicating values of the moderator for which conditional effects differ significantly from 0).

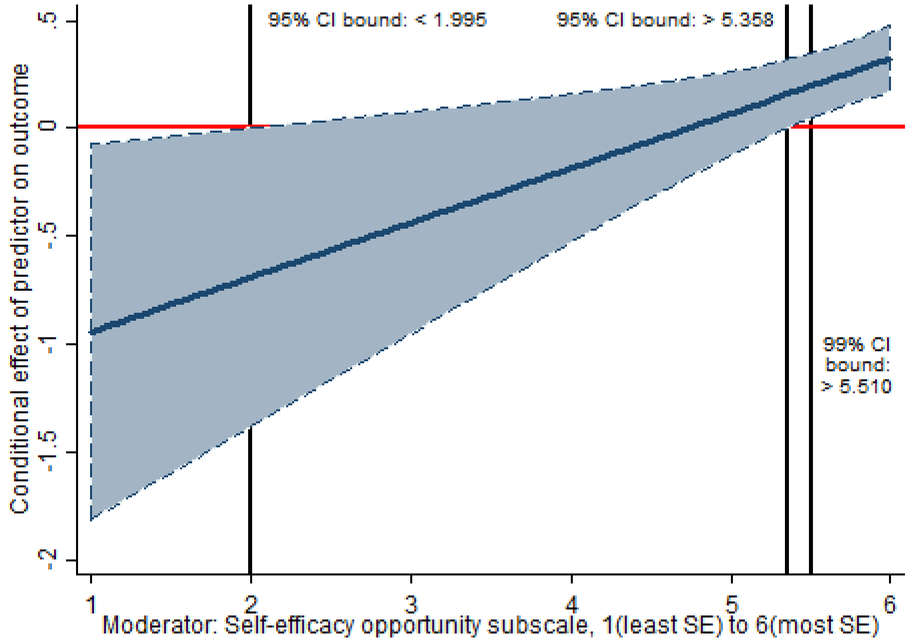

Figure S4.190. The conditional effects of peer influence from average friends' responses to DN2.3 at baseline (predictor) on focal participants' values of DN2.3 at follow-up (outcome) by self-efficacy opportunity subscale (moderator) with 95% CI limits for conditional effects, and bounds indicating regions of significance at the 95% and 99% levels (indicating values of the moderator for which conditional effects differ significantly from 0).

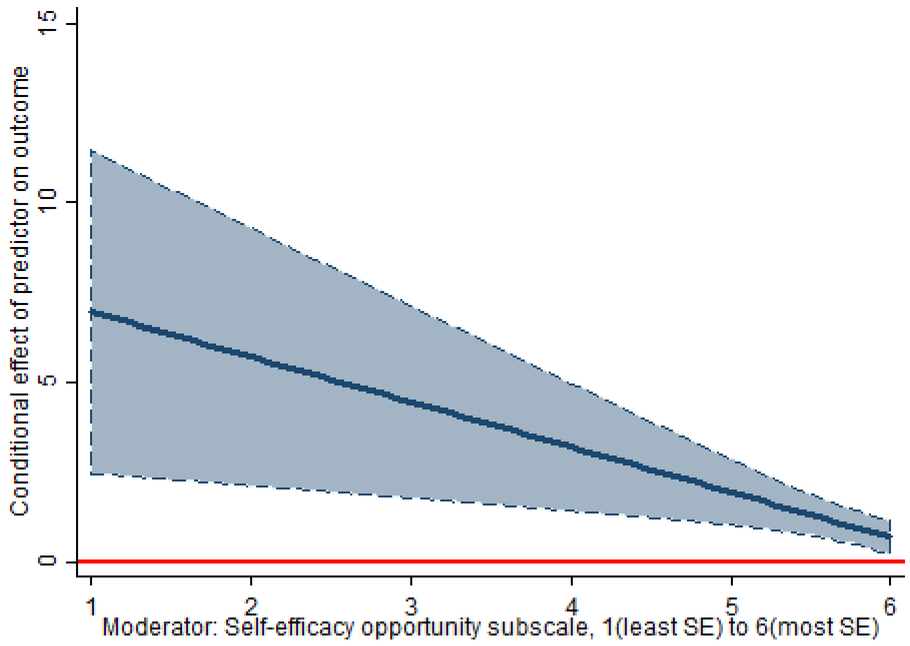

Figure S4.191. The conditional effects of peer influence from average school year group intentions at baseline (predictor) on focal participants' intentions at follow-up (outcome) by self-efficacy opportunity subscale (moderator) with 95% CI limits for conditional effects.

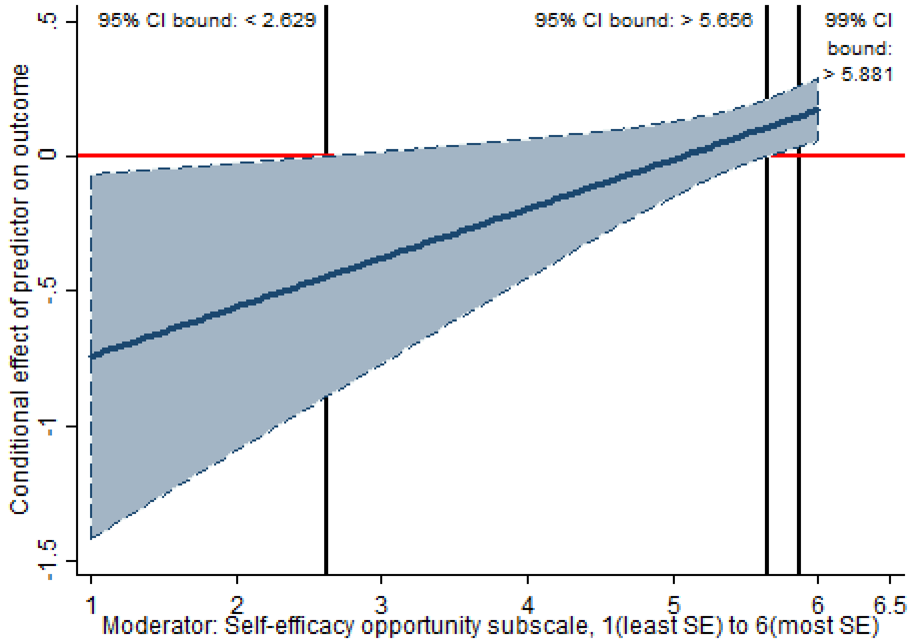

Figure S4.192. The conditional effects of peer influence from average friends' perceived physical risks at follow-up (predictor) on focal participants' perceived physical risks at follow-up (outcome) by self-efficacy opportunity subscale (moderator) with 95% CI limits for conditional effects, and bounds indicating regions of significance at the 95% and 99% levels (indicating values of the moderator for which conditional effects differ significantly from 0).

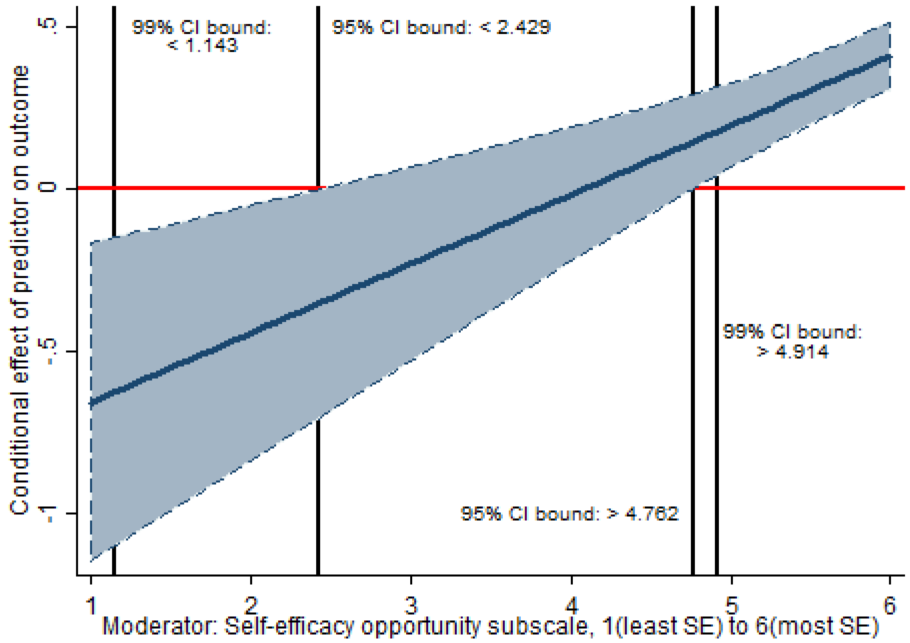

Figure S4.193. The conditional effects of peer influence from average friends' objectively measured smoking behavior at baseline (predictor) on focal participants' objectively measured smoking behavior at follow-up (outcome) by self-efficacy opportunity subscale (moderator) with 95% CI limits for conditional effects, and bounds indicating regions of significance at the 95% and 99% levels (indicating values of the moderator for which conditional effects differ significantly from 0).

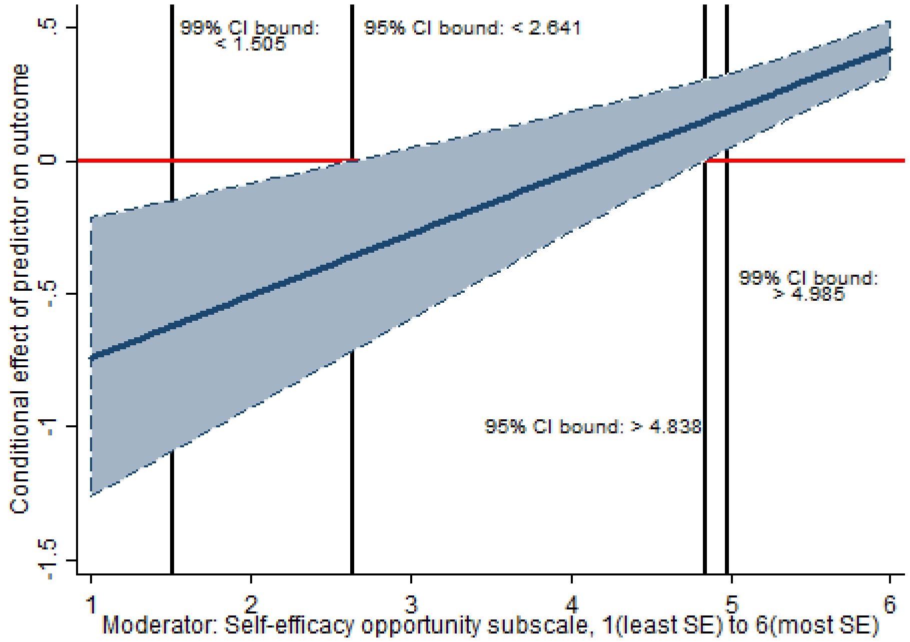

Figure S4.194. The conditional effects of peer influence from average school class objectively measured smoking behavior at baseline (predictor) on focal participants' objectively measured smoking behavior at follow-up (outcome) by self-efficacy opportunity subscale (moderator) with 95% CI limits for conditional effects, and bounds indicating regions of significance at the 95% and 99% levels (indicating values of the moderator for which conditional effects differ significantly from 0).

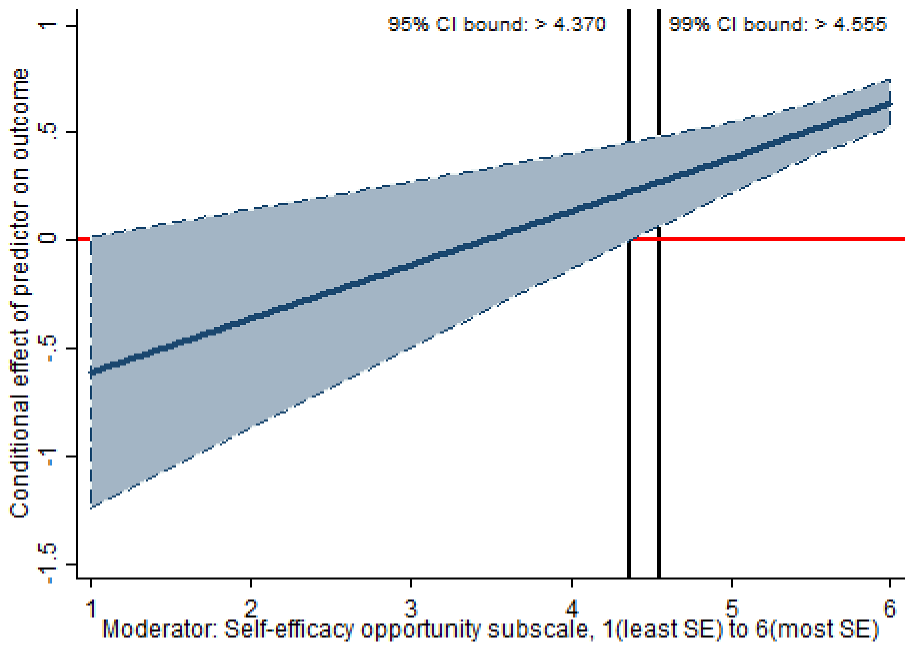

Figure S4.195. The conditional effects of peer influence from average school year group objectively measured smoking behavior at baseline (predictor) on focal participants' objectively measured smoking behavior at follow-up (outcome) by self-efficacy opportunity subscale (moderator) with 95% CI limits for conditional effects, and bounds indicating regions of significance at the 95% and 99% levels (indicating values of the moderator for which conditional effects differ significantly from 0).
