# Supplementary material for: Moderators of peer influence effects for adolescents’ smoking and vaping norms and outcomes in high and middle-income settings
Source: Front Psychol. 2025 Nov 3;16:1655761. doi: 10.3389/fpsyg.2025.1655761 (PMC12620383; doi:10.3389/fpsyg.2025.1655761)
Supplement: SUPPLEMENTARY FILE 5 — Multiverse figures. [file Table_5.docx]

Supplementary File 5

Moderators of peer influence effects for adolescents’ smoking and vaping norms and outcomes in high and middle-income settings.

**Jennifer M. Murray*, Sharon C. Sánchez-Franco, Olga L. Sarmiento, Erik O. Kimbrough, Christopher Tate, Shannon C. Montgomery, Rajnish Kumar, Laura Dunne, Abhijit Ramalingam, Erin L. Krupka, Felipe Montes, Huiyu Zhou, Laurence Moore, Linda Bauld, Blanca Llorente, Frank Kee, Ruth F. Hunter***

*** Correspondence:** Corresponding Authors: [jmurray39@qub.ac.uk](mailto:jmurray39@qub.ac.uk), [ruth.hunter@qub.ac.uk](mailto:ruth.hunter@qub.ac.uk)

**This file includes:**

Supplementary Tables S5.1. to S5.92. (multiverse figures).

**
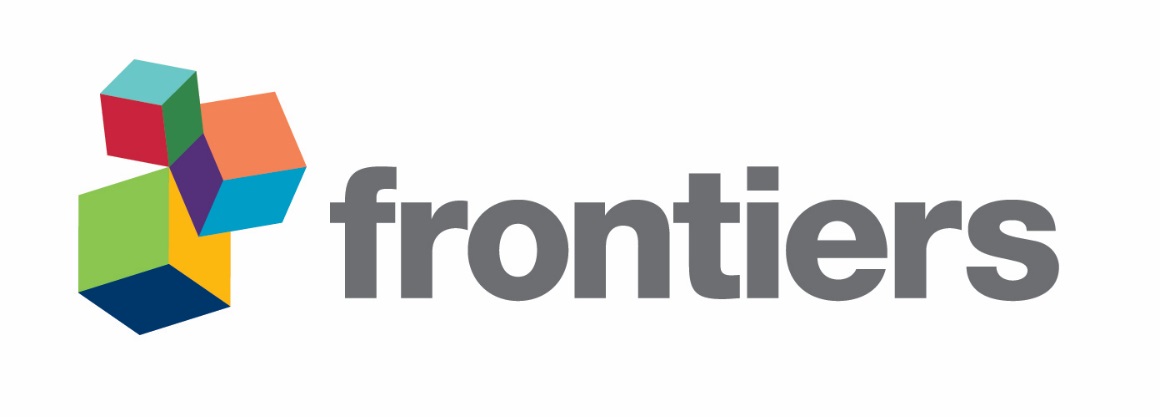
**

**Supplementary file 5: Multiverse figures.**

**Figure S5.1.** Histogram showing the distribution of p-values for interaction effects across the 276 models with setting as the moderator (mean=0.43, median=0.40, 20 [7.2%] at p≤0.01, 35 [11.6%] at p≤0.05).

**Figure S5.2.** **Volcano plot for the 270 models with setting (0=Northern Ireland; 1=Bogotá) as the moderator.** The negative logs of the p-values for the interaction effects are shown on the y-axis (larger values on the y-axis correspond to smaller p-values). Standardized regression coefficients for the interaction effects are shown on the x-axis (the estimated difference in standard deviations of the outcome variable between two cases that differ by one standard deviation on the ‘peer group average’ predictor variable, for pupils in Bogotá compared to pupils in Northern Ireland). The red dashed lines show the cut-off points where p=0.015 and p=0.055, with observations lying above the lines attaining statistical significance at the p≤0.01 and p≤0.05 levels respectively. The grey dashed lines show the 1^st^, 50^th^, and 99^th^ percentiles of the distributions of p-values and the standardized regression coefficients (mean=-0.003, median=-0.001, 1^st^ percentile=-0.47, 99^th^ percentile=0.33). The results of logistic regression models including smoking susceptibility as the outcome variable are not included in the plot.

**Figure S5.3.** **Visualization of the multiverse of standardized regression coefficients for interaction effects in the 270 models with setting (0=Northern Ireland; 1=Bogotá) as the moderator.** Standardized regression coefficients can be interpreted as the estimated difference in standard deviations of the outcome variable between two cases that differ by one standard deviation on the ‘peer group average’ predictor variable, for pupils in Bogotá compared to pupils in Northern Ireland. Logistic regression models including smoking susceptibility as the outcome variable are not included. Cells highlighted in gold indicate p≤0.01. Cells highlighted in red indicate p≤0.05.

For example, the result "0.04" in the top lefthand side of the figure indicates that the estimated difference in standard deviations of the outcome P2S2 at follow-up (outcome variable) between two pupils who differ by one standard deviation on the average of their nominated friends' P2S2 scores at baseline (predictor variable) was 0.04 standard deviations higher for pupils in Bogotá compared to Northern Ireland.

**Figure S5.4.** **Visualization of the multiverse of p-values for interaction effects in the 276 models with setting (0=Northern Ireland; 1=Bogotá) as the moderator.** Logistic regression models including smoking susceptibility as the outcome variable are included. Cells highlighted in gold indicate p≤0.01. Cells highlighted in red indicate p≤0.05.

**Figure S5.5.** Histogram showing the distribution of p-values for interaction effects across the 276 models with intervention as the moderator (mean=0.44, median=0.40, 11 [4.0%] at p≤0.01, 21 [7.6%] at p≤0.05).

**Figure S5.6.** **Volcano plot for the 270 models with intervention (1=ASSIST; 2=Dead Cool) as the moderator.** The negative logs of the p-values for the interaction effects are shown on the y-axis (larger values on the y-axis correspond to smaller p-values). Standardized regression coefficients for the interaction effects are shown on the x-axis (the estimated difference in standard deviations of the outcome variable between two cases that differ by one standard deviation on the ‘peer group average’ predictor variable, for pupils in Dead Cool schools compared to pupils in ASSIST schools). The red dashed lines show the cut-off points where p=0.015 and p=0.055, with observations lying above the lines attaining statistical significance at the p≤0.01 and p≤0.05 levels respectively. The grey dashed lines show the 1^st^, 50^th^, and 99^th^ percentiles of the distributions of p-values and the standardized regression coefficients (mean=0.00008, median=0.002, 1^st^ percentile=-0.21, 99^th^ percentile=0.18). The results of logistic regression models including smoking susceptibility as the outcome variable are not included in the plot.

**Figure S5.7.** **Visualization of the multiverse of standardized regression coefficients for interaction effects in the 270 models with intervention (1=ASSIST; 2=Dead Cool) as the moderator.** Standardized regression coefficients can be interpreted as the estimated difference in standard deviations of the outcome variable between two cases that differ by one standard deviation on the ‘peer group average’ predictor variable, for pupils in Dead Cool schools compared to pupils in ASSIST schools. Logistic regression models including smoking susceptibility as the outcome variable are not included. Cells highlighted in gold indicate p≤0.01. Cells highlighted in red indicate p≤0.05.

For example, the result "0.08" in the top lefthand side of the figure indicates that the estimated difference in standard deviations of the outcome P2S2 at follow-up (outcome variable) between two pupils who differ by one standard deviation on the average of their nominated friends' P2S2 scores at baseline (predictor variable) was 0.08 standard deviations higher for pupils in Dead Cool schools compared to ASSIST schools.

**Figure S5.8.** **Visualization of the multiverse of p-values for interaction effects in the 276 models with intervention (1=ASSIST; 2=Dead Cool) as the moderator.** Logistic regression models including smoking susceptibility as the outcome variable are included. Cells highlighted in gold indicate p≤0.01. Cells highlighted in red indicate p≤0.05.

**Figure S5.9.** Histogram showing the distribution of p-values for interaction effects across the 276 models with gender as the moderator (mean=0.43, median=0.41, 18 [6.5%] at p≤0.01, 36 [13.0%] at p≤0.05).

**Figure S5.10.** **Volcano plot for the 270 models with gender (0=boy; 1=girl/prefer not to say) as the moderator.** The negative logs of the p-values for the interaction effects are shown on the y-axis (larger values on the y-axis correspond to smaller p-values). Standardized regression coefficients for the interaction effects are shown on the x-axis (the estimated difference in standard deviations of the outcome variable between two cases that differ by one standard deviation on the ‘peer group average’ predictor variable, for boys compared to girls/prefer not to say). The red dashed lines show the cut-off points where p=0.015 and p=0.055, with observations lying above the lines attaining statistical significance at the p≤0.01 and p≤0.05 levels respectively. The grey dashed lines show the 1^st^, 50^th^, and 99^th^ percentiles of the distributions of p-values and the standardized regression coefficients (mean=0.02, median=0.01, 1^st^ percentile=-0.14, 99^th^ percentile=0.21). The results of logistic regression models including smoking susceptibility as the outcome variable are not included in the plot.

**Figure S5.11.** **Visualization of the multiverse of standardized regression coefficients for interaction effects in the 270 models with gender (0=boy; 1=girl/prefer not to say) as the moderator.** Standardized regression coefficients can be interpreted as the estimated difference in standard deviations of the outcome variable between two cases that differ by one standard deviation on the ‘peer group average’ predictor variable, for boys compared to girls/prefer not to say. Logistic regression models including smoking susceptibility as the outcome variable are not included. Cells highlighted in gold indicate p≤0.01. Cells highlighted in red indicate p≤0.05.

For example, the result "0.00" in the top lefthand side of the figure indicates that the estimated difference in standard deviations of the outcome P2S2 at follow-up (outcome variable) between two pupils who differ by one standard deviation on the average of their nominated friends' P2S2 scores at baseline (predictor variable) was 0.00 standard deviations higher for girls/prefer not to say compared to boys.

**Figure S5.12.** **Visualization of the multiverse of p-values for interaction effects in the 276 models with gender (0=boy; 1=girl/prefer not to say) as the moderator.** Logistic regression models including smoking susceptibility as the outcome variable are included. Cells highlighted in gold indicate p≤0.01. Cells highlighted in red indicate p≤0.05.

**Figure S5.13.** Histogram showing the distribution of p-values for interaction effects across the 276 models with school socio-economic status (all schools) as the moderator (mean=0.44, median=0.41, 15 [5.4%] at p≤0.01, 23 [8.3%] at p≤0.05).

**Figure S5.14.** **Volcano plot for the 270 models with school socio-economic status in all schools (1 [lowest SES] to 4 [highest SES]) as the moderator.** The negative logs of the p-values for the interaction effects are shown on the y-axis (larger values on the y-axis correspond to smaller p-values). Standardized regression coefficients for the interaction effects are shown on the x-axis (the estimated difference in standard deviations of the outcome variable between two cases that differ by one standard deviation on the ‘peer group average’ predictor variable, as school socio-economic status changes by one standard deviation). The red dashed lines show the cut-off points where p=0.015 and p=0.055, with observations lying above the lines attaining statistical significance at the p≤0.01 and p≤0.05 levels respectively. The grey dashed lines show the 1^st^, 50^th^, and 99^th^ percentiles of the distributions of p-values and the standardized regression coefficients (mean=-0.003, median=-0.002, 1^st^ percentile=-0.13, 99^th^ percentile=0.10). The results of logistic regression models including smoking susceptibility as the outcome variable are not included in the plot.

**Figure S5.15.** **Visualization of the multiverse of standardized regression coefficients for interaction effects in the 270 models with school socio-economic status in all schools (1 [lowest SES] to 4 [highest SES]) as the moderator.** Standardized regression coefficients can be interpreted as the estimated difference in standard deviations of the outcome variable between two cases that differ by one standard deviation on the ‘peer group average’ predictor variable, as school socio-economic status changes by one standard deviation. Logistic regression models including smoking susceptibility as the outcome variable are not included. Cells highlighted in gold indicate p≤0.01. Cells highlighted in red indicate p≤0.05.

For example, the result "-0.01" in the top lefthand side of the figure indicates that the estimated difference in standard deviations of the outcome P2S2 at follow-up (outcome variable) between two pupils who differ by one standard deviation on the average of their nominated friends' P2S2 scores at baseline (predictor variable) decreased by 0.01 standard deviations as school socio-economic status increased by one standard deviation.

**Figure S5.16.** **Visualization of the multiverse of p-values for interaction effects in the 276 models with school socio-economic status in all schools (1 [lowest SES] to 4 [highest SES]) as the moderator.** Logistic regression models including smoking susceptibility as the outcome variable are included. Cells highlighted in gold indicate p≤0.01. Cells highlighted in red indicate p≤0.05.

**Figure S5.17.** Histogram showing the distribution of p-values for interaction effects across the 92 models with school socio-economic status (NI schools) as the moderator (mean=0.41, median=0.39, 8 [8.7%] at p≤0.01, 14 [15.2%] at p≤0.05).

**Figure S5.18.** **Volcano plot for the 90 models with school socio-economic status in NI schools (5.7 [lowest SES; NIMDM2017=57] to 80.2 [highest SES; NIMDM2017=802]) as the moderator.** The negative logs of the p-values for the interaction effects are shown on the y-axis (larger values on the y-axis correspond to smaller p-values). Standardized regression coefficients for the interaction effects are shown on the x-axis (the estimated difference in standard deviations of the outcome variable between two cases that differ by one standard deviation on the ‘peer group average’ predictor variable, as school socio-economic status changes by one standard deviation). The red dashed lines show the cut-off points where p=0.015 and p=0.055, with observations lying above the lines attaining statistical significance at the p≤0.01 and p≤0.05 levels respectively. The grey dashed lines show the 1^st^, 50^th^, and 99^th^ percentiles of the distributions of p-values and the standardized regression coefficients (mean=-0.02, median=-0.02, 1^st^ percentile=-0.24, 99^th^ percentile=0.14). The results of logistic regression models including smoking susceptibility as the outcome variable are not included in the plot.

**Figure S5.19.** **Visualization of the multiverse of standardized regression coefficients for interaction effects in the 90 models with school socio-economic status in NI schools (5.7 [lowest SES; NIMDM2017=57] to 80.2 [highest SES; NIMDM2017=802]) as the moderator.** Standardized regression coefficients can be interpreted as the estimated difference in standard deviations of the outcome variable between two cases that differ by one standard deviation on the ‘peer group average’ predictor variable, as school socio-economic status changes by one standard deviation. Logistic regression models including smoking susceptibility as the outcome variable are not included. Cells highlighted in gold indicate p≤0.01. Cells highlighted in red indicate p≤0.05.

For example, the result "-0.05" in the top lefthand side of the figure indicates that the estimated difference in standard deviations of the outcome P2S2 at follow-up (outcome variable) between two pupils who differ by one standard deviation on the average of their nominated friends' P2S2 scores at baseline (predictor variable) decreased by 0.05 standard deviations as school socio-economic status increased by one standard deviation.

**Figure S5.20.** **Visualization of the multiverse of p-values for interaction effects in the 92 models with school socio-economic status in NI schools (5.7 [lowest SES; NIMDM2017=57] to 80.2 [highest SES; NIMDM2017=802]) as the moderator.** Logistic regression models including smoking susceptibility as the outcome variable are included. Cells highlighted in gold indicate p≤0.01. Cells highlighted in red indicate p≤0.05.

**Figure S5.21.** Histogram showing the distribution of p-values for interaction effects across the 92 models with school socio-economic status (Bogotá schools) as the moderator (mean=0.49, median=0.49, 4 [4.3%] at p≤0.01, 9 [9.8%] at p≤0.05).

**Figure S5.22.** **Volcano plot for the 90 models with school socio-economic status in Bogotá schools (1 [lowest SES; Lower] to 4 [highest SES; Higher]) as the moderator.** The negative logs of the p-values for the interaction effects are shown on the y-axis (larger values on the y-axis correspond to smaller p-values). Standardized regression coefficients for the interaction effects are shown on the x-axis (the estimated difference in standard deviations of the outcome variable between two cases that differ by one standard deviation on the ‘peer group average’ predictor variable, as school socio-economic status changes by one standard deviation). The red dashed lines show the cut-off points where p=0.015 and p=0.055, with observations lying above the lines attaining statistical significance at the p≤0.01 and p≤0.05 levels respectively. The grey dashed lines show the 1^st^, 50^th^, and 99^th^ percentiles of the distributions of p-values and the standardized regression coefficients (mean=0.02, median=0.02, 1^st^ percentile=-0.15, 99^th^ percentile=0.16). The results of logistic regression models including smoking susceptibility as the outcome variable are not included in the plot.

**Figure S5.23.** **Visualization of the multiverse of standardized regression coefficients for interaction effects in the 90 models with school socio-economic status in Bogotá schools (1 [lowest SES; Lower] to 4 [highest SES; Higher]) as the moderator.** Standardized regression coefficients can be interpreted as the estimated difference in standard deviations of the outcome variable between two cases that differ by one standard deviation on the ‘peer group average’ predictor variable, as school socio-economic status changes by one standard deviation. Logistic regression models including smoking susceptibility as the outcome variable are not included. Cells highlighted in gold indicate p≤0.01. Cells highlighted in red indicate p≤0.05.

For example, the result "0.02" in the top lefthand side of the figure indicates that the estimated difference in standard deviations of the outcome P2S2 at follow-up (outcome variable) between two pupils who differ by one standard deviation on the average of their nominated friends' P2S2 scores at baseline (predictor variable) increased by 0.02 standard deviations as school socio-economic status increased by one standard deviation.

**Figure S5.24.** **Visualization of the multiverse of p-values for interaction effects in the 92 models with school socio-economic status in Bogotá schools (1 [lowest SES; Lower] to 4 [highest SES; Higher]) as the moderator.** Logistic regression models including smoking susceptibility as the outcome variable are included. Cells highlighted in gold indicate p≤0.01. Cells highlighted in red indicate p≤0.05.

**Figure S5.25.** Histogram showing the distribution of p-values for interaction effects across the 276 models with rule-following as the moderator (mean=0.45, median=0.42, 5 [1.8%] at p≤0.01, 14 [5.1%] at p≤0.05).

**Figure S5.26.** **Volcano plot for the 270 models with norm sensitivities/rule-following (0 [least RF] to 5 [most RF]) as the moderator.** The negative logs of the p-values for the interaction effects are shown on the y-axis (larger values on the y-axis correspond to smaller p-values). Standardized regression coefficients for the interaction effects are shown on the x-axis (the estimated difference in standard deviations of the outcome variable between two cases that differ by one standard deviation on the ‘peer group average’ predictor variable, as rule-following changes by one standard deviation). The red dashed lines show the cut-off points where p=0.015 and p=0.055, with observations lying above the lines attaining statistical significance at the p≤0.01 and p≤0.05 levels respectively. The grey dashed lines show the 1^st^, 50^th^, and 99^th^ percentiles of the distributions of p-values and the standardized regression coefficients (mean=0.004, median=0.004, 1^st^ percentile=-0.07, 99^th^ percentile=0.07). The results of logistic regression models including smoking susceptibility as the outcome variable are not included in the plot.

**Figure S5.27.** **Visualization of the multiverse of standardized regression coefficients for interaction effects in the 270 models with norm sensitivities/rule-following (0 [least RF] to 5 [most RF]) as the moderator.** Standardized regression coefficients can be interpreted as the estimated difference in standard deviations of the outcome variable between two cases that differ by one standard deviation on the ‘peer group average’ predictor variable, as rule-following changes by one standard deviation. Logistic regression models including smoking susceptibility as the outcome variable are not included. Cells highlighted in gold indicate p≤0.01. Cells highlighted in red indicate p≤0.05.

For example, the result "0.02" in the top lefthand side of the figure indicates that the estimated difference in standard deviations of the outcome P2S2 at follow-up (outcome variable) between two pupils who differ by one standard deviation on the average of their nominated friends' P2S2 scores at baseline (predictor variable) increased by 0.02 standard deviations as norm sensitivities/rule-following increased by one standard deviation.

**Figure S5.28.** **Visualization of the multiverse of p-values for interaction effects in the 276 models with norm sensitivities/rule-following (0 [least RF] to 5 [most RF]) as the moderator.** Logistic regression models including smoking susceptibility as the outcome variable are included. Cells highlighted in gold indicate p≤0.01. Cells highlighted in red indicate p≤0.05.

**Figure S5.29.** Histogram showing the distribution of p-values for interaction effects across the 276 models with pro-sociality as the moderator (mean=0.43, median=0.41, 14 [5.1%] at p≤0.01, 35 [12.7%] at p≤0.05).

**Figure S5.30.** **Volcano plot for the 270 models with pro-sociality (0 [least pro-sociality] to 10 [most pro-sociality]) as the moderator.** The negative logs of the p-values for the interaction effects are shown on the y-axis (larger values on the y-axis correspond to smaller p-values). Standardized regression coefficients for the interaction effects are shown on the x-axis (the estimated difference in standard deviations of the outcome variable between two cases that differ by one standard deviation on the ‘peer group average’ predictor variable, as pro-sociality changes by one standard deviation). The red dashed lines show the cut-off points where p=0.015 and p=0.055, with observations lying above the lines attaining statistical significance at the p≤0.01 and p≤0.05 levels respectively. The grey dashed lines show the 1^st^, 50^th^, and 99^th^ percentiles of the distributions of p-values and the standardized regression coefficients (mean=0.009, median=0.01, 1^st^ percentile=-0.07, 99^th^ percentile=0.08). The results of logistic regression models including smoking susceptibility as the outcome variable are not included in the plot.

**Figure S5.31.** **Visualization of the multiverse of standardized regression coefficients for interaction effects in the 270 models with pro-sociality (0 [least pro-sociality] to 10 [most pro-sociality]) as the moderator.** Standardized regression coefficients can be interpreted as the estimated difference in standard deviations of the outcome variable between two cases that differ by one standard deviation on the ‘peer group average’ predictor variable, as pro-sociality changes by one standard deviation. Logistic regression models including smoking susceptibility as the outcome variable are not included. Cells highlighted in gold indicate p≤0.01. Cells highlighted in red indicate p≤0.05.

For example, the result "-0.02" in the top lefthand side of the figure indicates that the estimated difference in standard deviations of the outcome P2S2 at follow-up (outcome variable) between two pupils who differ by one standard deviation on the average of their nominated friends' P2S2 scores at baseline (predictor variable) decreased by 0.02 standard deviations as pro-sociality increased by one standard deviation.

**Figure S5.32.** **Visualization of the multiverse of p-values for interaction effects in the 276 models with pro-sociality (0 [least pro-sociality] to 10 [most pro-sociality]) as the moderator.** Logistic regression models including smoking susceptibility as the outcome variable are included. Cells highlighted in gold indicate p≤0.01. Cells highlighted in red indicate p≤0.05.

**Figure S5.33.** Histogram showing the distribution of p-values for interaction effects across the 276 models with fear of negative evaluation as the moderator (mean=0.43, median=0.40, 8 [2.9%] at p≤0.01, 45 [16.3%] at p≤0.05).

**Figure S5.34.** **Volcano plot for the 270 models with fear of negative evaluation (1 [least FNE] to 5 [most FNE]) as the moderator.** The negative logs of the p-values for the interaction effects are shown on the y-axis (larger values on the y-axis correspond to smaller p-values). Standardized regression coefficients for the interaction effects are shown on the x-axis (the estimated difference in standard deviations of the outcome variable between two cases that differ by one standard deviation on the ‘peer group average’ predictor variable, as FNE changes by one standard deviation). The red dashed lines show the cut-off points where p=0.015 and p=0.055, with observations lying above the lines attaining statistical significance at the p≤0.01 and p≤0.05 levels respectively. The grey dashed lines show the 1^st^, 50^th^, and 99^th^ percentiles of the distributions of p-values and the standardized regression coefficients (mean=0.01, median=0.01, 1^st^ percentile=-0.06, 99^th^ percentile=0.09). The results of logistic regression models including smoking susceptibility as the outcome variable are not included in the plot.

**Figure S5.35.** **Visualization of the multiverse of standardized regression coefficients for interaction effects in the 270 models with fear of negative evaluation (1 [least FNE] to 5 [most FNE]) as the moderator.** Standardized regression coefficients can be interpreted as the estimated difference in standard deviations of the outcome variable between two cases that differ by one standard deviation on the ‘peer group average’ predictor variable, as FNE changes by one standard deviation. Logistic regression models including smoking susceptibility as the outcome variable are not included. Cells highlighted in gold indicate p≤0.01. Cells highlighted in red indicate p≤0.05.

For example, the result "0.06" in the top lefthand side of the figure indicates that the estimated difference in standard deviations of the outcome P2S2 at follow-up (outcome variable) between two pupils who differ by one standard deviation on the average of their nominated friends' P2S2 scores at baseline (predictor variable) increased by 0.06 standard deviations as fear of negative evaluation increased by one standard deviation.

**Figure S5.36.** **Visualization of the multiverse of p-values for interaction effects in the 276 models with fear of negative evaluation (1 [least FNE] to 5 [most FNE]) as the moderator.** Logistic regression models including smoking susceptibility as the outcome variable are included. Cells highlighted in gold indicate p≤0.01. Cells highlighted in red indicate p≤0.05.

**Figure S5.37.** Histogram showing the distribution of p-values for interaction effects across the 276 models with need to belong as the moderator (mean=0.49, median=0.48, 6 [2.2%] at p≤0.01, 19 [6.9%] at p≤0.05).

**Figure S5.38.** **Volcano plot for the 270 models with need to belong (1 [least NTB] to 5 [most NTB]) as the moderator.** The negative logs of the p-values for the interaction effects are shown on the y-axis (larger values on the y-axis correspond to smaller p-values). Standardized regression coefficients for the interaction effects are shown on the x-axis (the estimated difference in standard deviations of the outcome variable between two cases that differ by one standard deviation on the ‘peer group average’ predictor variable, as NTB changes by one standard deviation). The red dashed lines show the cut-off points where p=0.015 and p=0.055, with observations lying above the lines attaining statistical significance at the p≤0.01 and p≤0.05 levels respectively. The grey dashed lines show the 1^st^, 50^th^, and 99^th^ percentiles of the distributions of p-values and the standardized regression coefficients (mean=0.01, median=0.009, 1^st^ percentile=-0.08, 99^th^ percentile=0.09). The results of logistic regression models including smoking susceptibility as the outcome variable are not included in the plot.

**Figure S5.39.** **Visualization of the multiverse of standardized regression coefficients for interaction effects in the 270 models with need to belong (1 [least NTB] to 5 [most NTB]) as the moderator.** Standardized regression coefficients can be interpreted as the estimated difference in standard deviations of the outcome variable between two cases that differ by one standard deviation on the ‘peer group average’ predictor variable, as NTB changes by one standard deviation. Logistic regression models including smoking susceptibility as the outcome variable are not included. Cells highlighted in gold indicate p≤0.01. Cells highlighted in red indicate p≤0.05.

For example, the result "0.00" in the top lefthand side of the figure indicates that the estimated difference in standard deviations of the outcome P2S2 at follow-up (outcome variable) between two pupils who differ by one standard deviation on the average of their nominated friends' P2S2 scores at baseline (predictor variable) increased by 0.00 standard deviations as need to belong increased by one standard deviation.

**Figure S5.40.** **Visualization of the multiverse of p-values for interaction effects in the 276 models with need to belong (1 [least NTB] to 5 [most NTB]) as the moderator.** Logistic regression models including smoking susceptibility as the outcome variable are included. Cells highlighted in gold indicate p≤0.01. Cells highlighted in red indicate p≤0.05.

**Figure S5.41.** Histogram showing the distribution of p-values for interaction effects across the 276 models with openness as the moderator (mean=0.48, median=0.48, 6 [2.2%] at p≤0.01, 16 [5.8%] at p≤0.05).

**Figure S5.42.** **Volcano plot for the 270 models with openness (0 [least openness] to 4 [most openness]) as the moderator.** The negative logs of the p-values for the interaction effects are shown on the y-axis (larger values on the y-axis correspond to smaller p-values). Standardized regression coefficients for the interaction effects are shown on the x-axis (the estimated difference in standard deviations of the outcome variable between two cases that differ by one standard deviation on the ‘peer group average’ predictor variable, as openness changes by one standard deviation). The red dashed lines show the cut-off points where p=0.015 and p=0.055, with observations lying above the lines attaining statistical significance at the p≤0.01 and p≤0.05 levels respectively. The grey dashed lines show the 1^st^, 50^th^, and 99^th^ percentiles of the distributions of p-values and the standardized regression coefficients (mean=0.006, median=0.005, 1^st^ percentile=-0.06, 99^th^ percentile=0.09). The results of logistic regression models including smoking susceptibility as the outcome variable are not included in the plot.

**Figure S5.43.** **Visualization of the multiverse of standardized regression coefficients for interaction effects in the 270 models with openness (0 [least openness] to 4 [most openness]) as the moderator.** Standardized regression coefficients can be interpreted as the estimated difference in standard deviations of the outcome variable between two cases that differ by one standard deviation on the ‘peer group average’ predictor variable, as openness changes by one standard deviation. Logistic regression models including smoking susceptibility as the outcome variable are not included. Cells highlighted in gold indicate p≤0.01. Cells highlighted in red indicate p≤0.05.

For example, the result "-0.01" in the top lefthand side of the figure indicates that the estimated difference in standard deviations of the outcome P2S2 at follow-up (outcome variable) between two pupils who differ by one standard deviation on the average of their nominated friends' P2S2 scores at baseline (predictor variable) decreased by 0.01 standard deviations as openness increased by one standard deviation.

**Figure S5.44.** **Visualization of the multiverse of p-values for interaction effects in the 276 models with openness (0 [least openness] to 4 [most openness]) as the moderator.** Logistic regression models including smoking susceptibility as the outcome variable are included. Cells highlighted in gold indicate p≤0.01. Cells highlighted in red indicate p≤0.05.

**Figure S5.45.** Histogram showing the distribution of p-values for interaction effects across the 276 models with extraversion as the moderator (mean=0.49, median=0.49, 10 [3.6%] at p≤0.01, 24 [8.7%] at p≤0.05).

**Figure S5.46.** **Volcano plot for the 270 models with extraversion (0 [least extraverted] to 4 [most extraverted]) as the moderator.** The negative logs of the p-values for the interaction effects are shown on the y-axis (larger values on the y-axis correspond to smaller p-values). Standardized regression coefficients for the interaction effects are shown on the x-axis (the estimated difference in standard deviations of the outcome variable between two cases that differ by one standard deviation on the ‘peer group average’ predictor variable, as extraversion changes by one standard deviation). The red dashed lines show the cut-off points where p=0.015 and p=0.055, with observations lying above the lines attaining statistical significance at the p≤0.01 and p≤0.05 levels respectively. The grey dashed lines show the 1^st^, 50^th^, and 99^th^ percentiles of the distributions of p-values and the standardized regression coefficients (mean=-0.0001, median=-0.001, 1^st^ percentile=-0.08, 99^th^ percentile=0.09). The results of logistic regression models including smoking susceptibility as the outcome variable are not included in the plot.

**Figure S5.47.** **Visualization of the multiverse of standardized regression coefficients for interaction effects in the 270 models with extraversion (0 [least extraverted] to 4 [most extraverted]) as the moderator.** Standardized regression coefficients can be interpreted as the estimated difference in standard deviations of the outcome variable between two cases that differ by one standard deviation on the ‘peer group average’ predictor variable, as extraversion changes by one standard deviation. Logistic regression models including smoking susceptibility as the outcome variable are not included. Cells highlighted in gold indicate p≤0.01. Cells highlighted in red indicate p≤0.05.

For example, the result "-0.03" in the top lefthand side of the figure indicates that the estimated difference in standard deviations of the outcome P2S2 at follow-up (outcome variable) between two pupils who differ by one standard deviation on the average of their nominated friends' P2S2 scores at baseline (predictor variable) decreased by 0.03 standard deviations as extraversion increased by one standard deviation.

**Figure S5.48.** **Visualization of the multiverse of p-values for interaction effects in the 276 models with extraversion (0 [least extraverted] to 4 [most extraverted]) as the moderator.** Logistic regression models including smoking susceptibility as the outcome variable are included. Cells highlighted in gold indicate p≤0.01. Cells highlighted in red indicate p≤0.05.

**Figure S5.49.** Histogram showing the distribution of p-values for interaction effects across the 276 models with agreeableness as the moderator (mean=0.50, median=0.50, 4 [1.4%] at p≤0.01, 11 [4.0%] at p≤0.05).

**Figure S5.50.** **Volcano plot for the 270 models with agreeableness (0 [least agreeable] to 4 [most agreeable]) as the moderator.** The negative logs of the p-values for the interaction effects are shown on the y-axis (larger values on the y-axis correspond to smaller p-values). Standardized regression coefficients for the interaction effects are shown on the x-axis (the estimated difference in standard deviations of the outcome variable between two cases that differ by one standard deviation on the ‘peer group average’ predictor variable, as agreeableness changes by one standard deviation). The red dashed lines show the cut-off points where p=0.015 and p=0.055, with observations lying above the lines attaining statistical significance at the p≤0.01 and p≤0.05 levels respectively. The grey dashed lines show the 1^st^, 50^th^, and 99^th^ percentiles of the distributions of p-values and the standardized regression coefficients (mean=-0.007, median=-0.005, 1^st^ percentile=-0.08, 99^th^ percentile=0.06). The results of logistic regression models including smoking susceptibility as the outcome variable are not included in the plot.

**Figure S5.51.** **Visualization of the multiverse of standardized regression coefficients for interaction effects in the 270 models with agreeableness (0 [least agreeable] to 4 [most agreeable]) as the moderator.** Standardized regression coefficients can be interpreted as the estimated difference in standard deviations of the outcome variable between two cases that differ by one standard deviation on the ‘peer group average’ predictor variable, as agreeableness changes by one standard deviation. Logistic regression models including smoking susceptibility as the outcome variable are not included. Cells highlighted in gold indicate p≤0.01. Cells highlighted in red indicate p≤0.05.

For example, the result "-0.04" in the top lefthand side of the figure indicates that the estimated difference in standard deviations of the outcome P2S2 at follow-up (outcome variable) between two pupils who differ by one standard deviation on the average of their nominated friends' P2S2 scores at baseline (predictor variable) decreased by 0.04 standard deviations as agreeableness increased by one standard deviation.

**Figure S5.52.** **Visualization of the multiverse of p-values for interaction effects in the 276 models with agreeableness (0 [least agreeable] to 4 [most agreeable]) as the moderator.** Logistic regression models including smoking susceptibility as the outcome variable are included. Cells highlighted in gold indicate p≤0.01. Cells highlighted in red indicate p≤0.05.

**Figure S5.53.** Histogram showing the distribution of p-values for interaction effects across the 276 models with conscientiousness as the moderator (mean=0.46, median=0.44, 4 [1.4%] at p≤0.01, 22 [8.0%] at p≤0.05).

**Figure S5.54.** **Volcano plot for the 270 models with conscientiousness (0 [least conscientious] to 4 [most conscientious]) as the moderator.** The negative logs of the p-values for the interaction effects are shown on the y-axis (larger values on the y-axis correspond to smaller p-values). Standardized regression coefficients for the interaction effects are shown on the x-axis (the estimated difference in standard deviations of the outcome variable between two cases that differ by one standard deviation on the ‘peer group average’ predictor variable, as conscientiousness changes by one standard deviation). The red dashed lines show the cut-off points where p=0.015 and p=0.055, with observations lying above the lines attaining statistical significance at the p≤0.01 and p≤0.05 levels respectively. The grey dashed lines show the 1^st^, 50^th^, and 99^th^ percentiles of the distributions of p-values and the standardized regression coefficients (mean=-0.007, median=-0.008, 1^st^ percentile=-0.07, 99^th^ percentile=0.06). The results of logistic regression models including smoking susceptibility as the outcome variable are not included in the plot.

**Figure S5.55.** **Visualization of the multiverse of standardized regression coefficients for interaction effects in the 270 models with conscientiousness (0 [least conscientious] to 4 [most conscientious]) as the moderator.** Standardized regression coefficients can be interpreted as the estimated difference in standard deviations of the outcome variable between two cases that differ by one standard deviation on the ‘peer group average’ predictor variable, as conscientiousness changes by one standard deviation. Logistic regression models including smoking susceptibility as the outcome variable are not included. Cells highlighted in gold indicate p≤0.01. Cells highlighted in red indicate p≤0.05.

For example, the result "-0.01" in the top lefthand side of the figure indicates that the estimated difference in standard deviations of the outcome P2S2 at follow-up (outcome variable) between two pupils who differ by one standard deviation on the average of their nominated friends' P2S2 scores at baseline (predictor variable) decreased by 0.01 standard deviations as conscientiousness increased by one standard deviation.

**Figure S5.56.** **Visualization of the multiverse of p-values for interaction effects in the 276 models with conscientiousness (0 [least conscientious] to 4 [most conscientious]) as the moderator.** Logistic regression models including smoking susceptibility as the outcome variable are included. Cells highlighted in gold indicate p≤0.01. Cells highlighted in red indicate p≤0.05.

**Figure S5.57.** Histogram showing the distribution of p-values for interaction effects across the 276 models with emotional stability as the moderator (mean=0.50, median=0.48, 1 [0.4%] at p≤0.01, 9 [3.3%] at p≤0.05).

**Figure S5.58.** **Volcano plot for the 270 models with emotional stability (0 [least stability] to 4 [most stability]) as the moderator.** The negative logs of the p-values for the interaction effects are shown on the y-axis (larger values on the y-axis correspond to smaller p-values). Standardized regression coefficients for the interaction effects are shown on the x-axis (the estimated difference in standard deviations of the outcome variable between two cases that differ by one standard deviation on the ‘peer group average’ predictor variable, as stability changes by one standard deviation). The red dashed lines show the cut-off points where p=0.015 and p=0.055, with observations lying above the lines attaining statistical significance at the p≤0.01 and p≤0.05 levels respectively. The grey dashed lines show the 1^st^, 50^th^, and 99^th^ percentiles of the distributions of p-values and the standardized regression coefficients (mean=-0.007, median=-0.008, 1^st^ percentile=-0.08, 99^th^ percentile=0.06). The results of logistic regression models including smoking susceptibility as the outcome variable are not included in the plot.

**Figure S5.59.** **Visualization of the multiverse of standardized regression coefficients for interaction effects in the 270 models with emotional stability (0 [least stability] to 4 [most stability]) as the moderator.** Standardized regression coefficients can be interpreted as the estimated difference in standard deviations of the outcome variable between two cases that differ by one standard deviation on the ‘peer group average’ predictor variable, as stability changes by one standard deviation. Logistic regression models including smoking susceptibility as the outcome variable are not included. Cells highlighted in gold indicate p≤0.01. Cells highlighted in red indicate p≤0.05.

For example, the result "-0.08" in the top lefthand side of the figure indicates that the estimated difference in standard deviations of the outcome P2S2 at follow-up (outcome variable) between two pupils who differ by one standard deviation on the average of their nominated friends' P2S2 scores at baseline (predictor variable) decreased by 0.08 standard deviations as emotional stability increased by one standard deviation.

**Figure S5.60.** **Visualization of the multiverse of p-values for interaction effects in the 276 models with emotional stability (0 [least stability] to 4 [most stability]) as the moderator.** Logistic regression models including smoking susceptibility as the outcome variable are included. Cells highlighted in gold indicate p≤0.01. Cells highlighted in red indicate p≤0.05.

**Figure S5.61.** Histogram showing the distribution of p-values for interaction effects across the 276 models with social network clustering coefficients as the moderator (mean=0.51, median=0.53, 4 [1.4%] at p≤0.01, 13 [4.7%] at p≤0.05).

**Figure S5.62.** **Volcano plot for the 270 models with clustering coefficients (0 [least interconnections between nominated friends] to 10 [most interconnections between nominated friends]) as the moderator.** The negative logs of the p-values for the interaction effects are shown on the y-axis (larger values on the y-axis correspond to smaller p-values). Standardized regression coefficients for the interaction effects are shown on the x-axis (the estimated difference in standard deviations of the outcome variable between two cases that differ by one standard deviation on the ‘peer group average’ predictor variable, as clustering coefficients change by one standard deviation). The red dashed lines show the cut-off points where p=0.015 and p=0.055, with observations lying above the lines attaining statistical significance at the p≤0.01 and p≤0.05 levels respectively. The grey dashed lines show the 1^st^, 50^th^, and 99^th^ percentiles of the distributions of p-values and the standardized regression coefficients (mean=-0.0004, median=0.0003, 1^st^ percentile=-0.07, 99^th^ percentile=0.07). The results of logistic regression models including smoking susceptibility as the outcome variable are not included in the plot.

**Figure S5.63.** **Visualization of the multiverse of standardized regression coefficients for interaction effects in the 270 models with clustering coefficients (0 [least interconnections between nominated friends] to 10 [most interconnections between nominated friends]) as the moderator.** Standardized regression coefficients can be interpreted as the estimated difference in standard deviations of the outcome variable between two cases that differ by one standard deviation on the ‘peer group average’ predictor variable, as clustering coefficients change by one standard deviation. Logistic regression models including smoking susceptibility as the outcome variable are not included. Cells highlighted in gold indicate p≤0.01. Cells highlighted in red indicate p≤0.05.

For example, the result "-0.01" in the top lefthand side of the figure indicates that the estimated difference in standard deviations of the outcome P2S2 at follow-up (outcome variable) between two pupils who differ by one standard deviation on the average of their nominated friends' P2S2 scores at baseline (predictor variable) decreased by 0.01 standard deviations as network clustering coefficients at baseline increased by one standard deviation.

**Figure S5.64.** **Visualization of the multiverse of p-values for interaction effects in the 276 models with clustering coefficients (0 [least interconnections between nominated friends] to 10 [most interconnections between nominated friends]) as the moderator.** Logistic regression models including smoking susceptibility as the outcome variable are included. Cells highlighted in gold indicate p≤0.01. Cells highlighted in red indicate p≤0.05.

**Figure S5.65.** Histogram showing the distribution of p-values for interaction effects across the 276 models with social network eigenvector centralities as the moderator (mean=0.43, median=0.40, 11 [4.0%] at p≤0.01, 33 [12.0%] at p≤0.05).

**Figure S5.66.** **Volcano plot for the 270 models with eigenvector centralities (baseline: 0.008 [least well-connected friends, i.e. least central] to 3.13 [most well-connected friends, i.e. most central]; follow-up: 0.003 to 3.04) as the moderator.** The negative logs of the p-values for the interaction effects are shown on the y-axis (larger values on the y-axis correspond to smaller p-values). Standardized regression coefficients for the interaction effects are shown on the x-axis (the estimated difference in standard deviations of the outcome variable between two cases that differ by one standard deviation on the ‘peer group average’ predictor variable, as eigenvector centralities change by one standard deviation). The red dashed lines show the cut-off points where p=0.015 and p=0.055, with observations lying above the lines attaining statistical significance at the p≤0.01 and p≤0.05 levels respectively. The grey dashed lines show the 1^st^, 50^th^, and 99^th^ percentiles of the distributions of p-values and the standardized regression coefficients (mean=0.009, median=0.008, 1^st^ percentile=-0.08, 99^th^ percentile=0.10). The results of logistic regression models including smoking susceptibility as the outcome variable are not included in the plot.

**Figure S5.67.** **Visualization of the multiverse of standardized regression coefficients for interaction effects in the 270 models with eigenvector centralities (baseline: 0.008 [least well-connected friends, i.e. least central] to 3.13 [most well-connected friends, i.e. most central]; follow-up: 0.003 to 3.04) as the moderator.** Standardized regression coefficients can be interpreted as the estimated difference in standard deviations of the outcome variable between two cases that differ by one standard deviation on the ‘peer group average’ predictor variable, as eigenvector centralities change by one standard deviation. Logistic regression models including smoking susceptibility as the outcome variable are not included. Cells highlighted in gold indicate p≤0.01. Cells highlighted in red indicate p≤0.05.

For example, the result "0.01" in the top lefthand side of the figure indicates that the estimated difference in standard deviations of the outcome P2S2 at follow-up (outcome variable) between two pupils who differ by one standard deviation on the average of their nominated friends' P2S2 scores at baseline (predictor variable) increased by 0.01 standard deviations as network eigenvector centralities at baseline increased by one standard deviation.

**Figure S5.68.** **Visualization of the multiverse of p-values for interaction effects in the 276 models with eigenvector centralities (baseline: 0.008 [least well-connected friends, i.e. least central] to 3.13 [most well-connected friends, i.e. most central]; follow-up: 0.003 to 3.04) as the moderator.** Logistic regression models including smoking susceptibility as the outcome variable are included. Cells highlighted in gold indicate p≤0.01. Cells highlighted in red indicate p≤0.05.

**Figure S5.69.** Histogram showing the distribution of p-values for interaction effects across the 276 models with social network closeness centralities as the moderator (mean=0.40, median=0.33, 13 [4.7%] at p≤0.01, 30 [10.9%] at p≤0.05).

**Figure S5.70.** **Volcano plot for the 270 models with closeness centralities (baseline: 2.24 [longest distance to all other nodes, i.e. least central] to 5.10 [shortest distance to all other nodes, i.e. most central]; follow-up: 2.15 to 4.94) as the moderator.** The negative logs of the p-values for the interaction effects are shown on the y-axis (larger values on the y-axis correspond to smaller p-values). Standardized regression coefficients for the interaction effects are shown on the x-axis (the estimated difference in standard deviations of the outcome variable between two cases that differ by one standard deviation on the ‘peer group average’ predictor variable, as closeness centralities change by one standard deviation). The red dashed lines show the cut-off points where p=0.015 and p=0.055, with observations lying above the lines attaining statistical significance at the p≤0.01 and p≤0.05 levels respectively. The grey dashed lines show the 1^st^, 50^th^, and 99^th^ percentiles of the distributions of p-values and the standardized regression coefficients (mean=0.02, median=0.02, 1^st^ percentile=-0.07, 99^th^ percentile=0.11). The results of logistic regression models including smoking susceptibility as the outcome variable are not included in the plot.

**Figure S5.71.** **Visualization of the multiverse of standardized regression coefficients for interaction effects in the 270 models with closeness centralities (baseline: 2.24 [longest distance to all other nodes, i.e. least central] to 5.10 [shortest distance to all other nodes, i.e. most central]; follow-up: 2.15 to 4.94) as the moderator.** Standardized regression coefficients can be interpreted as the estimated difference in standard deviations of the outcome variable between two cases that differ by one standard deviation on the ‘peer group average’ predictor variable, as closeness centralities change by one standard deviation. Logistic regression models including smoking susceptibility as the outcome variable are not included. Cells highlighted in gold indicate p≤0.01. Cells highlighted in red indicate p≤0.05.

For example, the result "-0.02" in the top lefthand side of the figure indicates that the estimated difference in standard deviations of the outcome P2S2 at follow-up (outcome variable) between two pupils who differ by one standard deviation on the average of their nominated friends' P2S2 scores at baseline (predictor variable) decreased by 0.02 standard deviations as network closeness centralities at baseline increased by one standard deviation.

**Figure S5.72.** **Visualization of the multiverse of p-values for interaction effects in the 276 models with closeness centralities (baseline: 2.24 [longest distance to all other nodes, i.e. least central] to 5.10 [shortest distance to all other nodes, i.e. most central]; follow-up: 2.15 to 4.94) as the moderator.** Logistic regression models including smoking susceptibility as the outcome variable are included. Cells highlighted in gold indicate p≤0.01. Cells highlighted in red indicate p≤0.05.

**Figure S5.73.** Histogram showing the distribution of p-values for interaction effects across the 276 models with social network betweenness centralities as the moderator (mean=0.49, median=0.49, 3 [1.1%] at p≤0.01, 15 [5.4%] at p≤0.05).

**Figure S5.74.** **Volcano plot for the 270 models with betweenness centralities (baseline: 0 [node falls on the least proportion of shortest paths between two other nodes, i.e. least central] to 2.83 [node falls on the greatest proportion of shortest paths between two other nodes, i.e. most central]; follow-up: 0 to 5.49) as the moderator.** The negative logs of the p-values for the interaction effects are shown on the y-axis (larger values on the y-axis correspond to smaller p-values). Standardized regression coefficients for the interaction effects are shown on the x-axis (the estimated difference in standard deviations of the outcome variable between two cases that differ by one standard deviation on the ‘peer group average’ predictor variable, as betweenness centralities change by one standard deviation). The red dashed lines show the cut-off points where p=0.015 and p=0.055, with observations lying above the lines attaining statistical significance at the p≤0.01 and p≤0.05 levels respectively. The grey dashed lines show the 1^st^, 50^th^, and 99^th^ percentiles of the distributions of p-values and the standardized regression coefficients (mean=0.01, median=0.01, 1^st^ percentile=-0.07, 99^th^ percentile=0.16). The results of logistic regression models including smoking susceptibility as the outcome variable are not included in the plot.

**Figure S5.75.** **Visualization of the multiverse of standardized regression coefficients for interaction effects in the 270 models with betweenness centralities (baseline: 0 [node falls on the least proportion of shortest paths between two other nodes, i.e. least central] to 2.83 [node falls on the greatest proportion of shortest paths between two other nodes, i.e. most central]; follow-up: 0 to 5.49) as the moderator.** Standardized regression coefficients can be interpreted as the estimated difference in standard deviations of the outcome variable between two cases that differ by one standard deviation on the ‘peer group average’ predictor variable, as betweenness centralities change by one standard deviation. Logistic regression models including smoking susceptibility as the outcome variable are not included. Cells highlighted in gold indicate p≤0.01. Cells highlighted in red indicate p≤0.05.

For example, the result "-0.03" in the top lefthand side of the figure indicates that the estimated difference in standard deviations of the outcome P2S2 at follow-up (outcome variable) between two pupils who differ by one standard deviation on the average of their nominated friends' P2S2 scores at baseline (predictor variable) decreased by 0.03 standard deviations as network betweenness centralities at baseline increased by one standard deviation.

**Figure S5.76.** **Visualization of the multiverse of p-values for interaction effects in the 276 models with betweenness centralities (baseline: 0 [node falls on the least proportion of shortest paths between two other nodes, i.e. least central] to 2.83 [node falls on the greatest proportion of shortest paths between two other nodes, i.e. most central]; follow-up: 0 to 5.49) as the moderator.** Logistic regression models including smoking susceptibility as the outcome variable are included. Cells highlighted in gold indicate p≤0.01. Cells highlighted in red indicate p≤0.05.

**Figure S5.77.** Histogram showing the distribution of p-values for interaction effects across the 276 models with social network Gini degree coefficients as the moderator (mean=0.47, median=0.45, 11 [4.0%] at p≤0.01, 21 [7.6%] at p≤0.05).

**Figure S5.78.** **Volcano plot for the 270 models with Gini degree coefficients (baseline: 1.70 [least expected difference in degree between two randomly selected nodes, i.e. degree distribution is least heterogeneous] to 2.67 [greatest expected difference in degree between two randomly selected nodes, i.e. degree distribution is most heterogeneous]; follow-up: 1.91 to 2.91) as the moderator.** The negative logs of the p-values for the interaction effects are shown on the y-axis (larger values on the y-axis correspond to smaller p-values). Standardized regression coefficients for the interaction effects are shown on the x-axis (the estimated difference in standard deviations of the outcome variable between two cases that differ by one standard deviation on the ‘peer group average’ predictor variable, as Gini degree coefficients change by one standard deviation). The red dashed lines show the cut-off points where p=0.015 and p=0.055, with observations lying above the lines attaining statistical significance at the p≤0.01 and p≤0.05 levels respectively. The grey dashed lines show the 1^st^, 50^th^, and 99^th^ percentiles of the distributions of p-values and the standardized regression coefficients (mean=-0.02, median=-0.005, 1^st^ percentile=-0.31, 99^th^ percentile=0.12). The results of logistic regression models including smoking susceptibility as the outcome variable are not included in the plot.

**Figure S5.79.** **Visualization of the multiverse of standardized regression coefficients for interaction effects in the 270 models with Gini degree coefficients (baseline: 1.70 [least expected difference in degree between two randomly selected nodes, i.e. degree distribution is least heterogeneous] to 2.67 [greatest expected difference in degree between two randomly selected nodes, i.e. degree distribution is most heterogeneous]; follow-up: 1.91 to 2.91) as the moderator.** Standardized regression coefficients can be interpreted as the estimated difference in standard deviations of the outcome variable between two cases that differ by one standard deviation on the ‘peer group average’ predictor variable, as Gini degree coefficients change by one standard deviation. Logistic regression models including smoking susceptibility as the outcome variable are not included. Cells highlighted in gold indicate p≤0.01. Cells highlighted in red indicate p≤0.05.

For example, the result "0.03" in the top lefthand side of the figure indicates that the estimated difference in standard deviations of the outcome P2S2 at follow-up (outcome variable) between two pupils who differ by one standard deviation on the average of their nominated friends' P2S2 scores at baseline (predictor variable) increased by 0.03 standard deviations as network Gini degree coefficients at baseline increased by one standard deviation.

**Figure S5.80.** **Visualization of the multiverse of p-values for interaction effects in the 276 models with Gini degree coefficients (baseline: 1.70 [least expected difference in degree between two randomly selected nodes, i.e. degree distribution is least heterogeneous] to 2.67 [greatest expected difference in degree between two randomly selected nodes, i.e. degree distribution is most heterogeneous]; follow-up: 1.91 to 2.91) as the moderator.** Logistic regression models including smoking susceptibility as the outcome variable are included. Cells highlighted in gold indicate p≤0.01. Cells highlighted in red indicate p≤0.05.

**Figure S5.81.** Histogram showing the distribution of p-values for interaction effects across the 258 models with self-efficacy (emotional) as the moderator (mean=0.49, median=0.48, 5 [1.9%] at p≤0.01, 9 [3.5%] at p≤0.05).

**Figure S5.82.** **Volcano plot for the 252 models with self-efficacy (emotional; 1 [lowest self-efficacy to resist smoking] to 6 [greatest self-efficacy to resist smoking]) as the moderator.** The negative logs of the p-values for the interaction effects are shown on the y-axis (larger values on the y-axis correspond to smaller p-values). Standardized regression coefficients for the interaction effects are shown on the x-axis (the estimated difference in standard deviations of the outcome variable between two cases that differ by one standard deviation on the ‘peer group average’ predictor variable, as self-efficacy changes by one standard deviation). The red dashed lines show the cut-off points where p=0.015 and p=0.055, with observations lying above the lines attaining statistical significance at the p≤0.01 and p≤0.05 levels respectively. The grey dashed lines show the 1^st^, 50^th^, and 99^th^ percentiles of the distributions of p-values and the standardized regression coefficients (mean=-0.0004, median=0.004, 1^st^ percentile=-0.09, 99^th^ percentile=0.07). The results of logistic regression models including smoking susceptibility as the outcome variable are not included in the plot.

**Figure S5.83.** **Visualization of the multiverse of standardized regression coefficients for interaction effects in the 252 models with self-efficacy (emotional; 1 [lowest self-efficacy to resist smoking] to 6 [greatest self-efficacy to resist smoking]) as the moderator.** Standardized regression coefficients can be interpreted as the estimated difference in standard deviations of the outcome variable between two cases that differ by one standard deviation on the ‘peer group average’ predictor variable, as self-efficacy changes by one standard deviation. Logistic regression models including smoking susceptibility as the outcome variable are not included. Cells highlighted in gold indicate p≤0.01. Cells highlighted in red indicate p≤0.05.

For example, the result "-0.04" in the top lefthand side of the figure indicates that the estimated difference in standard deviations of the outcome P2S2 at follow-up (outcome variable) between two pupils who differ by one standard deviation on the average of their nominated friends' P2S2 scores at baseline (predictor variable) decreased by 0.04 standard deviations as self-efficacy (emotional) increased by one standard deviation.

**Figure S5.84.** **Visualization of the multiverse of p-values for interaction effects in the 258 models with self-efficacy (emotional; 1 [lowest self-efficacy to resist smoking] to 6 [greatest self-efficacy to resist smoking]) as the moderator.** Logistic regression models including smoking susceptibility as the outcome variable are included. Cells highlighted in gold indicate p≤0.01. Cells highlighted in red indicate p≤0.05.

**Figure S5.85.** Histogram showing the distribution of p-values for interaction effects across the 258 models with self-efficacy (friends) as the moderator (mean=0.48, median=0.48, 6 [2.3%] at p≤0.01, 14 [5.4%] at p≤0.05).

**Figure S5.86.** **Volcano plot for the 252 models with self-efficacy (friends; 1 [lowest self-efficacy to resist smoking] to 6 [greatest self-efficacy to resist smoking]) as the moderator.** The negative logs of the p-values for the interaction effects are shown on the y-axis (larger values on the y-axis correspond to smaller p-values). Standardized regression coefficients for the interaction effects are shown on the x-axis (the estimated difference in standard deviations of the outcome variable between two cases that differ by one standard deviation on the ‘peer group average’ predictor variable, as self-efficacy changes by one standard deviation). The red dashed lines show the cut-off points where p=0.015 and p=0.055, with observations lying above the lines attaining statistical significance at the p≤0.01 and p≤0.05 levels respectively. The grey dashed lines show the 1^st^, 50^th^, and 99^th^ percentiles of the distributions of p-values and the standardized regression coefficients (mean=-0.005, median=-0.005, 1^st^ percentile=-0.09, 99^th^ percentile=0.07). The results of logistic regression models including smoking susceptibility as the outcome variable are not included in the plot.

**Figure S5.87.** **Visualization of the multiverse of standardized regression coefficients for interaction effects in the 252 models with self-efficacy (friends; 1 [lowest self-efficacy to resist smoking] to 6 [greatest self-efficacy to resist smoking]) as the moderator.** Standardized regression coefficients can be interpreted as the estimated difference in standard deviations of the outcome variable between two cases that differ by one standard deviation on the ‘peer group average’ predictor variable, as self-efficacy changes by one standard deviation. Logistic regression models including smoking susceptibility as the outcome variable are not included. Cells highlighted in gold indicate p≤0.01. Cells highlighted in red indicate p≤0.05.

For example, the result "-0.04" in the top lefthand side of the figure indicates that the estimated difference in standard deviations of the outcome P2S2 at follow-up (outcome variable) between two pupils who differ by one standard deviation on the average of their nominated friends' P2S2 scores at baseline (predictor variable) decreased by 0.04 standard deviations as self-efficacy (friends) increased by one standard deviation.

**Figure S5.88.** **Visualization of the multiverse of p-values for interaction effects in the 258 models with self-efficacy (friends; 1 [lowest self-efficacy to resist smoking] to 6 [greatest self-efficacy to resist smoking]) as the moderator.** Logistic regression models including smoking susceptibility as the outcome variable are included. Cells highlighted in gold indicate p≤0.01. Cells highlighted in red indicate p≤0.05.

**Figure S5.89.** Histogram showing the distribution of p-values for interaction effects across the 258 models with self-efficacy (opportunity) as the moderator (mean=0.48, median=0.48, 8 [3.1%] at p≤0.01, 21 [8.1%] at p≤0.05).

**Figure S5.90.** **Volcano plot for the 252 models with self-efficacy (opportunity; 1 [lowest self-efficacy to resist smoking] to 6 [greatest self-efficacy to resist smoking]) as the moderator.** The negative logs of the p-values for the interaction effects are shown on the y-axis (larger values on the y-axis correspond to smaller p-values). Standardized regression coefficients for the interaction effects are shown on the x-axis (the estimated difference in standard deviations of the outcome variable between two cases that differ by one standard deviation on the ‘peer group average’ predictor variable, as self-efficacy changes by one standard deviation). The red dashed lines show the cut-off points where p=0.015 and p=0.055, with observations lying above the lines attaining statistical significance at the p≤0.01 and p≤0.05 levels respectively. The grey dashed lines show the 1^st^, 50^th^, and 99^th^ percentiles of the distributions of p-values and the standardized regression coefficients (mean=-0.0007, median=-0.002, 1^st^ percentile=-0.12, 99^th^ percentile=0.10). The results of logistic regression models including smoking susceptibility as the outcome variable are not included in the plot.

**Figure S5.91.** **Visualization of the multiverse of standardized regression coefficients for interaction effects in the 252 models with self-efficacy (opportunity; 1 [lowest self-efficacy to resist smoking] to 6 [greatest self-efficacy to resist smoking]) as the moderator.** Standardized regression coefficients can be interpreted as the estimated difference in standard deviations of the outcome variable between two cases that differ by one standard deviation on the ‘peer group average’ predictor variable, as self-efficacy changes by one standard deviation. Logistic regression models including smoking susceptibility as the outcome variable are not included. Cells highlighted in gold indicate p≤0.01. Cells highlighted in red indicate p≤0.05.

For example, the result "-0.03" in the top lefthand side of the figure indicates that the estimated difference in standard deviations of the outcome P2S2 at follow-up (outcome variable) between two pupils who differ by one standard deviation on the average of their nominated friends' P2S2 scores at baseline (predictor variable) decreased by 0.03 standard deviations as self-efficacy (opportunity) increased by one standard deviation.

**Figure S5.92.** **Visualization of the multiverse of p-values for interaction effects in the 258 models with self-efficacy (opportunity; 1 [lowest self-efficacy to resist smoking] to 6 [greatest self-efficacy to resist smoking]) as the moderator.** Logistic regression models including smoking susceptibility as the outcome variable are included. Cells highlighted in gold indicate p≤0.01. Cells highlighted in red indicate p≤0.05.
